# Sansanmycin natural product analogues as potent and selective anti-mycobacterials that inhibit lipid I biosynthesis

**DOI:** 10.1038/ncomms14414

**Published:** 2017-03-01

**Authors:** Anh T. Tran, Emma E. Watson, Venugopal Pujari, Trent Conroy, Luke J. Dowman, Andrew M. Giltrap, Angel Pang, Weng Ruh Wong, Roger G. Linington, Sebabrata Mahapatra, Jessica Saunders, Susan A. Charman, Nicholas P. West, Timothy D. H. Bugg, Julie Tod, Christopher G. Dowson, David I. Roper, Dean C. Crick, Warwick J. Britton, Richard J. Payne

**Affiliations:** 1 School of Chemistry, The University of Sydney, Sydney, New South Wales 2006, Australia; 2 Mycobacteria Research Laboratories, Department of Microbiology, Immunology and Pathology, Colorado State University, Fort Collins, Colorado 80523, USA; 3 Centenary Institute and Sydney Medical School, The University of Sydney, Sydney, New South Wales 2006, Australia; 4 Department of Chemistry and Biochemistry, University of California, Santa Cruz, California 95064, USA; 5 Department of Chemistry, Simon Fraser University, Burnaby, British Columbia, Canada V5A 1S6; 6 Centre for Drug Candidate Optimisation, Monash University, Melbourne, Victoria 3052, Australia; 7 School of Chemistry and Molecular Biosciences, University of Queensland, Brisbane, Queensland 4067, Australia; 8 Department of Chemistry, University of Warwick, Coventry CV4 7AL, UK; 9 School of Life Sciences, University of Warwick, Coventry CV4 7AL, UK

## Abstract

Tuberculosis (TB) is responsible for enormous global morbidity and mortality, and current treatment regimens rely on the use of drugs that have been in use for more than 40 years. Owing to widespread resistance to these therapies, new drugs are desperately needed to control the TB disease burden. Herein, we describe the rapid synthesis of analogues of the sansanmycin uridylpeptide natural products that represent promising new TB drug leads. The compounds exhibit potent and selective inhibition of *Mycobacterium tuberculosis*, the etiological agent of TB, both *in vitro* and intracellularly. The natural product analogues are nanomolar inhibitors of *Mtb* phospho-MurNAc-pentapeptide translocase, the enzyme responsible for the synthesis of lipid I in mycobacteria. This work lays the foundation for the development of uridylpeptide natural product analogues as new TB drug candidates that operate through the inhibition of peptidoglycan biosynthesis.

## Abstract (web summary)

Drug resistant tuberculosis (TB) infections are emerging at a high rate, with only few therapeutic options currently available. Here, the authors report synthetic analogues of the natural product sansanmycin that target mycobacterial cell wall biosynthesis and represent potent leads for improved anti-TB treatments.

Tuberculosis (TB) is caused by infection with the bacterium *Mycobacterium tuberculosis* (*Mtb*) and, according to the World Health Organization, was responsible for 1.5 million deaths and the emergence of 9.6 million new cases of the disease in 2014 (ref. 1). Current treatment for TB relies on a 6-month quadruple therapy comprising rifampicin, isoniazid, ethambutol and pyrazinamide^2^. Despite achieving a cure rate of >95% for drug-sensitive TB, this regimen is not effective against multi-drug resistant (MDR) and extensively drug resistant (XDR) TB infections, which are emerging at an alarming rate globally. While MDR infections can be treated with second-line antibiotics for extended periods, XDR infections are virtually untreatable as exemplified by a 98% mortality rate in a recent outbreak in South Africa^3^. To effectively combat these drug resistant cases, new TB drugs with novel modes of action are desperately needed. Two compounds, bedaquiline and delamanid, were recently approved for drug resistant TB; however these are only used as drugs of last resort owing to reported toxicity issues^4^.

It is well established that natural products provide a rich resource for the discovery of new drug leads^5^,^6^. Approximately 65% of antibacterials approved for use between 1981 and 2010 were natural products or natural product derivatives^5^,^6^, including currently employed TB drugs for example, rifampicin and the aminoglycosides. The sansanmycins belong to the uridylpeptide natural product family, a sub-class of the structurally diverse nucleoside antibiotics^7^,^8^ that include the liposidomycins^9^, caprazamycins^10^, capuramycins^11^,^12^ and muraymycins^13^. The sansanmycins are produced by the soil bacterium *Streptomyces sp.*^14^,^15^,^16^ and have a number of interesting structural features that are unique to the uridylpeptide natural product family. These include a 3′-deoxyuridine motif appended to *N*-methyl-2,3-diaminobutyric acid (DABA) via an unusual *cis*-4′,5′-enamide linkage. The DABA moiety is found within a ‘*pseudo*-tetrapeptide' chain which possesses the sites of variation in the family^7^. In addition to these fascinating structural features, the natural products have been shown to possess significant activity against *Mtb*, including MDR strains^14^,^15^. For these reasons we became interested in the sansanmycin scaffold as a privileged starting point for the discovery of novel TB drug leads.

In this manuscript we describe the rapid synthesis of a library of sansanmycin analogues that exhibit potent and selective activity against the virulent H37Rv strain of *Mtb*. We show that these natural product analogues disrupt the activity of *Mtb* phospho-MurNAc-pentapeptide translocase (variously designated as MurX in mycobacteria, MraY or translocase I in the literature), the integral membrane enzyme responsible for the biosynthesis of lipid I, a key intermediate in mycobacterial peptidoglycan synthesis.

## Results

### Anti-mycobacterial activity of dihydrosansanmycin A–C

**Figure 1:**
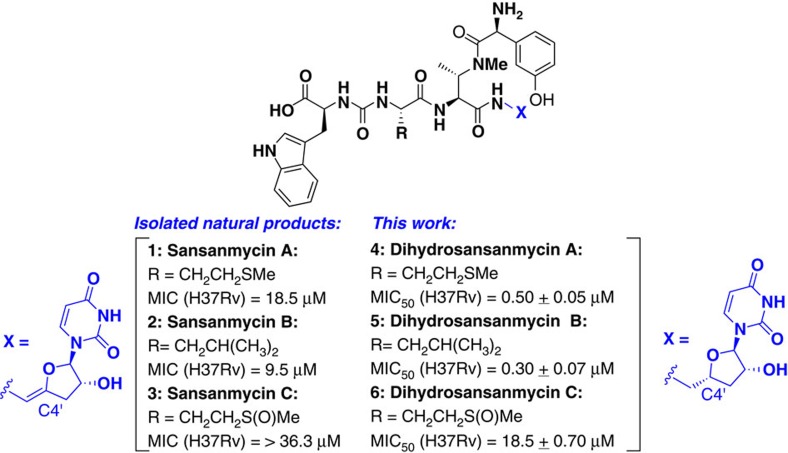
Structures of sansanmycins A-C (1–3), synthetic dihydrosansanmycins A–C (4–6) and inhibitory activity against *Mtb* H37Rv. MIC values of isolated sansanmycins A–C (**1–3**) were previously reported by Xie *et al*.^14^,^15^ MIC_50_ values of **4–6** represent the average of two independent experiments, each performed in triplicate; positive controls RIF: MIC_50_=0.006±0.002 μM; INH: MIC_50_=0.025±0.005 μM; MIC_50_ (HEK293) for **4–6**>200 μM.

Our initial goal focused on gaining access to structural analogues of the sansanmycin uridylpeptide natural product family (for example, analogues of sansanmycin A–C **1–3** (refs 14, 15), Fig. 1). To date, several derivatives of uridylpeptide natural products have been generated through engineering of the organisms that produce the natural products or through semi-synthetic approaches and, as such, feature limited structural variation^17^,^18^,^19^,^20^,^21^,^22^,^23^,^24^,^25^. A number of synthetic studies have also been reported on uridylpeptide natural products and analogues^26^,^27^,^28^,^29^,^30^,^31^,^32^,^33^, some of which have been shown to possess antimicrobial activity^7^,^8^. The focus of the present study was to develop a rapid and divergent synthetic strategy to access a diverse library of sansanmycin analogues that would enable the determination of key structure-activity relationships specifically against *Mtb.* In previous studies it has been shown that dihydropacidamycin analogues, lacking the enamide linkage maintained activity against *Pseudomonas aeruginosa*^23^. Given the potential metabolic lability of the enamide, we chose to target dihydrosansanmycin analogues in this study. Following the preparation of the first targets (Supplementary Methods) dihydrosansanmycins A–C (**4**–**6**), screening against the virulent H37Rv strain of *Mtb* was conducted using a resazurin assay^34^ (Fig. 1). These compounds were also counter-screened against HEK293 cells to gauge selectivity (Supplementary Methods). We were pleased to find that sansanmycin analogues **4–6** exhibited significant inhibitory activity against *Mtb*, with dihydrosansanmycin A (**4**) and B (**5**) possessing nanomolar MIC_50_ values (Fig. 1). Crucially, the stereochemistry at C-4′ of the ribose moiety was shown to be essential for anti-mycobacterial activity, with *S*-configuration in a dihydrosansanmycin B analogue leading to complete loss of activity (Supplementary Fig. 30). Interestingly, the importance of stereochemistry at C-4′ has also been reported for dihydropacidamycin, whereby analogues with *S*-stereochemistry at this position exhibited reduced activity against *P. aeruginosa* compared with the corresponding *R*-epimer^23^.

### Synthesis and activity of dihydrosansanmycin analogues

**Figure 2:**
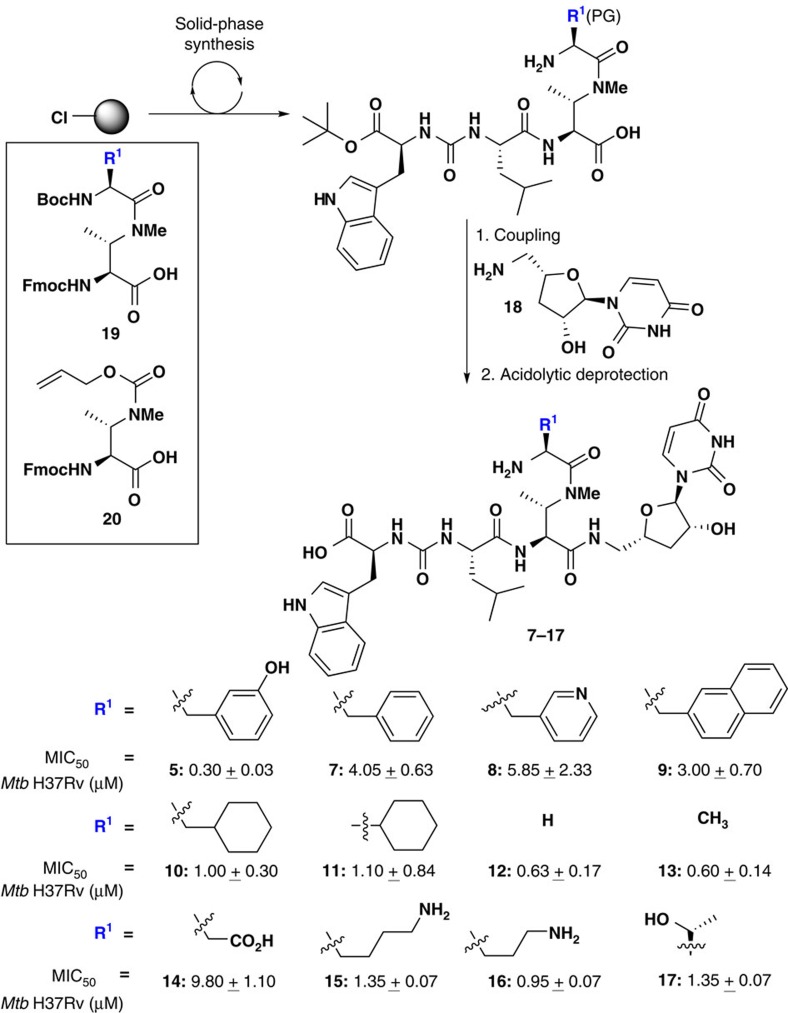
Synthesis of 1st generation sansanmycin analogues 7–17 with inhibitory activities against *Mtb* H37Rv. PG=side chain protection necessary for the synthesis of **5**, **14**, **15** and **16**. MIC_50_ values represent an average of two independent experiments each performed in triplicate; positive controls RIF: 0.006±0.002 μM; INH: MIC_50_=0.025±0.005 μM; MIC_50_ (HEK293) for **7–17**>200 μM.

Encouraged by the promising anti-mycobacterial activity of dihydrosansanmycin B (**5**), we next focused on the preparation of a targeted library of analogues of **5** to delineate the functional importance of the non-proteinogenic amino acid *m*-tyrosine (*m*-Tyr). As such, analogues **7–17**, retaining the DABA unit (due to the previously established importance of the *N*- and β-methyl moieties for activity^28^,^32^,^33^) but possessing a variety of functionalities in place of the *m*-Tyr moiety, were proposed (Fig. 2). Here, we took the opportunity to develop a robust divergent route to rapidly access the analogues. Preparation of **7–17** began with the synthesis of fully protected isopeptide fragments via Fmoc-strategy solid-phase peptide synthesis on 2-Cl Trt Cl resin. This was followed by late stage coupling of 4′-deoxyuridine fragment **18** which was synthesized from uridine in 12 steps using modifications to a method reported by Boojamra *et al*.^23^ (Fig. 2, Supplementary Methods). Two approaches were employed to access members of the library. The first involved coupling of suitably protected preformed isopeptide building blocks with a variety of amino acids appended to the DABA unit (general structure **19**). The second strategy involved the installation of allyloxycarbonyl (Alloc)-protected DABA fragment **20** onto the solid phase. This enabled late stage divergent access to the desired *pseudo*peptidic structures through *en bloc* Pd(0) deprotection followed by coupling of a variety of amino acids (R^1^ in Fig. 2, Supplementary Methods).

Following synthesis and purification by reverse-phase HPLC, analogues **7–17** were assessed against *Mtb* (H37Rv). We were pleased to find that most of the analogues retained significant activity against *Mtb*, albeit with a relatively flat structure-activity profile, with MIC_50_ values ranging from 0.6 to 9.80 μM for the series (Fig. 2). The incorporation of alternative aromatic substituents in compounds **7–9** proved to be detrimental with over an order of magnitude drop in activity. Incorporation of cyclohexyl-Ala (**10**), cyclohexyl Gly (**11**), Lys (**15**), Orn (**16**) or Thr (**17**) residues in place of *m*-Tyr did not lead to an appreciable decrease in activity (MIC_50_=0.95–1.35 μM), with the exception of the negatively charged Asp residue in **14** which led to a significant drop in potency. Interestingly, the replacement of *m*-Tyr with amino acids with smaller side chains for example, Gly in **12** (MIC_50_=0.63 μM) and Ala in **13** (MIC_50_=0.60 μM), provided anti-mycobacterials that were more potent than other substitutions at this position. As with dihydrosansanmycin A–C (**4–6**), compounds **7–17** were not cytotoxic to HEK293 cells up to a concentration of 200 μM. Given the promising activity of the natural product analogues against *Mtb*, we were next interested to assess the antimicrobial activity against other pathogenic bacteria to gauge whether there was any selectivity in activity. Towards this end, we counter-screened **4–17** against a panel of 15 pathogenic Gram-negative and Gram-positive bacterial strains using a high throughput screen, including several ‘ESKAPE' pathogens of clinical relevance^35^. The organisms in the screen included: *Bacillus subtilis*, *Staphylococcus aureus,* methicillin-resistant *S. aureus* (MRSA), *Staphylococcus epidermidis, Listeria ivanovii, Enterococcus faecium*, *Escherichia coli, Vibrio cholerae, Salmonella typhimurium, Pseudomonas aeruginosa, Yersinia pseudotuberculosis, Providencia alcalifaciens, Ochrobactrum anthropi, Enterobacter aerogenes, Acinetobacter baumannii*. Remarkably, while a small number of compounds exhibited modest activity against *E. coli* and *P. aeruginosa* (MIC_50_s: 12.5–100 μM), most did not inhibit the growth of other strains up to concentrations of 100 μM (Supplementary Tables 1 and 2). This selective anti-mycobacterial activity is an important criterion for the development of a TB drug lead and, as such, was a particularly encouraging observation.

### Second-generation dihydrosansanmycin analogues

**Figure 3:**
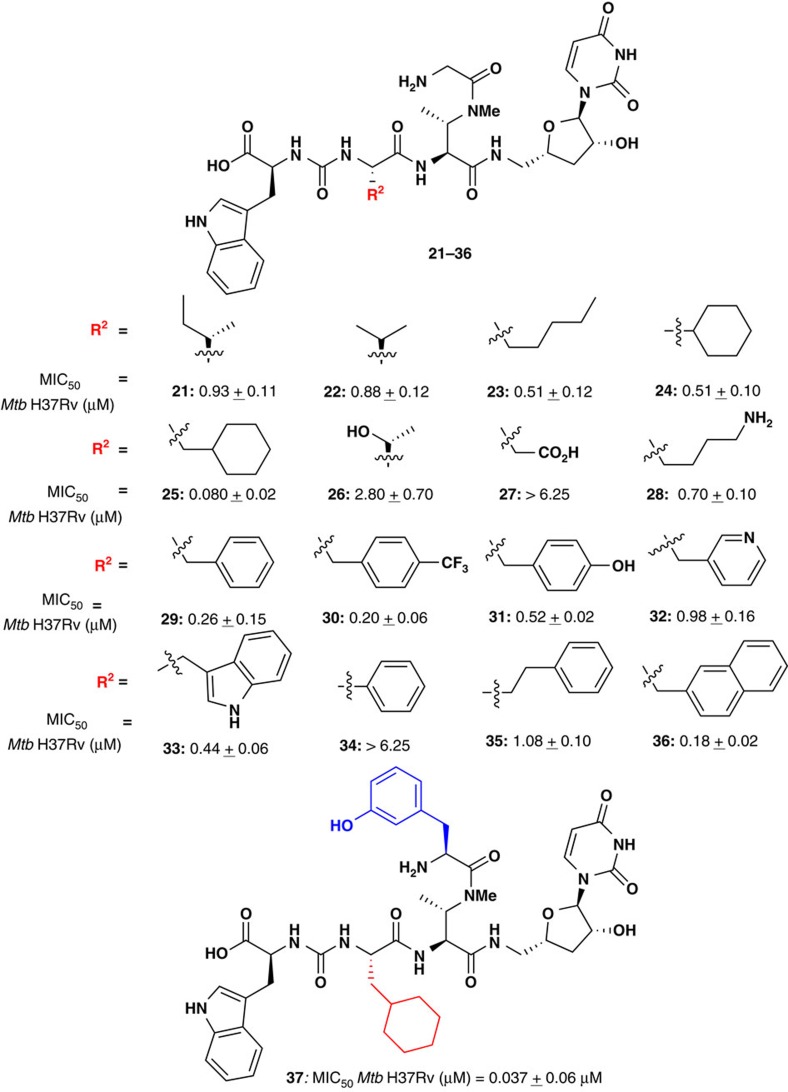
Synthesis of 2nd generation sansanmycin analogues 21–37 with inhibitory activities against *Mtb* H37Rv. MIC_50_ values represent average of two independent experiments, each performed in triplicate; positive controls RIF: MIC_50_=0.006±0.002 μM; INH: MIC_50_=0.025±0.005 μM; MIC_50_ (HEK293) for **21–37**>200 μM.

Because substitution of the synthetically challenging *m*-Tyr residue in **5** with a Gly residue in **12** did not lead to a substantial drop in anti-mycobacterial activity, we chose to maintain Gly at this position, and modify the Leu residue within the peptide chain (R in Fig. 1), in a second generation compound library. Specifically, a number of alkyl, heteroalkyl, aryl and heteroaryl substituents were proposed in analogues **21–36** with a view to interrogating further SARs. Dihydrosansanmycin analogues **21–36** were synthesized using the divergent solid-phase strategy described above (Supplementary Methods) and assessed for anti-mycobacterial activity against *Mtb* H37Rv (Fig. 3). Substitution of the isopropyl sidechain in **12** with different alkyl side chains in **21–24** did not lead to substantial differences in activity with MIC_50_ values ranging from 510 to 930 nM. Interestingly, extension of the cyclohexyl Gly side chain in **24** with a cyclohexyl Ala moiety led to a substantial increase in potency with analogue **25** exhibiting an MIC_50_ of 80 nM against *Mtb*. The introduction of more polar substituents, for example, a Thr side chain in **26** or an Asp residue in **27** led to a significant drop in activity (MIC_50_=2.80 μM and >6.25 μM, respectively) when compared with original lead **12**. A similar drop in activity was not observed when a Lys residue was introduced in **28** which was equipotent to **12** (MIC_50_=700 nM). Incorporation of aryl and heteroaryl groups in **29–36** in place of the Leu residue in **12** generally gave rise to improved anti-mycobacterial activity. Indeed, introduction of a Phe residue in **29** or a *p*-CF_3_ Phe residue in **30** led to a two-fold improvement in activity compared with **12**. Substitution with Tyr (**31**), 3-pyridyl Ala (**32**) and Trp (**33**) residues led to a modest drop in activity against *Mtb* compared with **29** (MIC_50_=0.44–0.98 μM). Shortening the side chain of Phe derivative **29** with a phenyl Gly side chain in **34** resulted in a dramatic drop in activity (MIC_50_>6.25 μM). This is in line with the reduced activity observed when shortening the side chain in the homologous analogues **24** and **25** with saturated cyclohexyl functionalities. Extending the Phe side chain in **29** by a further methylene unit in homo-Phe derivative **35** led to a four-fold loss in activity, while the incorporation of a naphthyl Ala side chain in **36** provided the most potent compound in the aryl substituted series with an MIC_50_ of 180 nM against *Mtb*. As with the first generation series of dihydrosansanmycin analogues, **21–36** did not show any activity (up to 200 μM) against HEK293 cells, and exhibited poor or no activity against the 15 pathogenic Gram-positive and Gram-negative bacterial strains (Supplementary Tables 1 and 2).

On the basis of the first generation (**7–17**, Fig. 2) analogue library, the optimal isopeptide substitution on the DABA unit was *m*-Tyr (found natively in the sansanmycins), whilst in the second generation library (**21–36**, Fig. 3), a cyclohexyl Ala residue provided the most potent anti-mycobacterial activity. With this in mind, analogue **37** was designed and synthesized that incorporated both of these functionalities (Fig. 3, Supplementary Methods). Gratifyingly, this analogue possessed the most potent activity against *Mtb* of all the natural product analogues synthesized with an MIC_50_ of 37 nM.

### Mechanism of action of sansanmycin analogues

**Figure 4:**
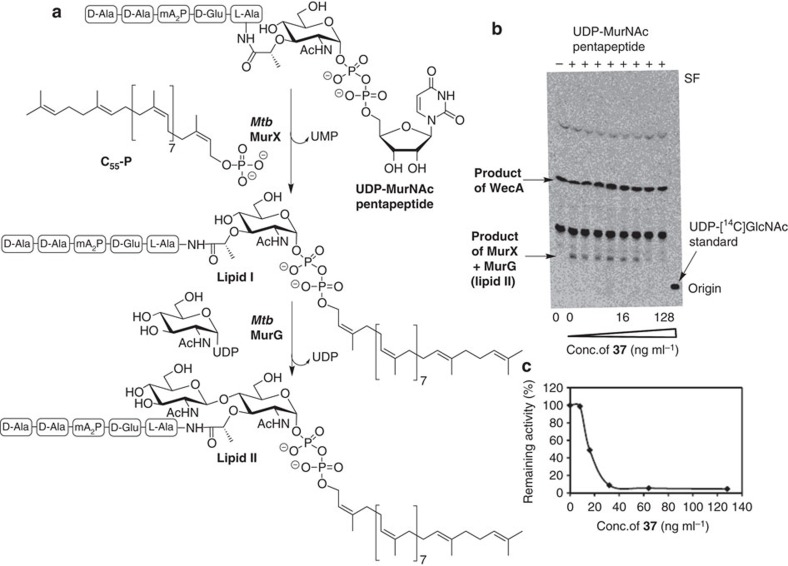
Inhibition studies of the synthetic sansanmycin analogues against *Mtb* MurX. (**a**) Reactions catalysed by *Mtb* MurX to generate lipid I and *Mtb* MurG to generate lipid II. (**b**) Exemplar TLC assay from *Mtb* mc^2^ 6230 membranes for the inhibition of *Mtb* MurX–MurG by dihydrosansanmycin analogue **37** (IC_50_=41 nM). Positive control=pacidamycin D^19^ and tunicamycin. Ala, alanine; Glu, glutamate; mA_2_P, *meso*-diaminopimelic acid. Product of MurX=decaprenyldiphosphoryl-MurNAc-pentapeptide (lipid I), product of MurG=decaprenyldiphosphoryl-MurNAc-pentapeptide-GlcNAc (lipid II), product of WecA=decaprenyldiphosphoryl-GlcNAc. (**c**) Inhibition assay of *Mtb* MurX with dihydrosansanmycin analogue **37** (IC_50_=16 nM) using dansylated UDP-MurNAc pentapeptide as substrate. *K*_m(app) UDP-MurNAc-pentapeptide_=51±4 μM and *V*_max(app)_=69±1.9 μM min^−1^; *K*_m(app) polyisoprenylphosphate_=56±9 μg ml^−1^ (Supplementary Figs 40 and 41). Positive control tunicamycin: IC_50_=189 nM (Supplementary Fig. 46).

It has been hypothesized that the antibacterial mechanism of action of the nucleoside antibiotic family is via inhibition of translocase I (MraY), an integral membrane protein that transfers UDP-MurNAc pentapeptide to polyisoprenylphosphate phosphate to afford lipid I, thereby catalysing the first membrane-associated step of peptidoglycan biosynthesis (Fig. 4a)^7^. Whilst inhibition of MraY has been unequivocally determined for some members of the family^8^,^25^,^28^,^36^,^37^,^38^, including a recently published structure of a complex of muraymycin D2 with the *Aquifex aeolicus* enzyme^25^, the antibacterial activity of other nucleoside antibiotics, including the sansanmycins, have only been predicted to be caused by inhibition of this enzyme. Furthermore, structural analogues of the nucleoside antibiotics have, on several occasions, been shown not to inhibit MraY, but to target other essential enzymes, suggesting that structural changes to the natural product can attenuate and even switch activity to other bacterial enzymes^39^,^40^. We were therefore interested in assessing the inhibitory activity of dihydrosansanmycin analogues **7–17** and **21–37** against *Mtb* translocase I. It is important to note that while the translocase I enzyme is dubbed MraY in most organisms, in mycobacteria this enzyme is encoded by the *murX* gene and is therefore referred to as MurX. An assay was developed to assess the inhibitory activity of the natural product analogues against *Mtb* MurX in an initial screen. Consistent with prior work on MraY orthologues of *Mtb* MurX (ref. 41), we were unable to overexpress the enzyme and therefore opted to assay the native enzyme in mycobacterial membranes. Briefly, *Mtb* mc^2^ 6230 membrane protein preparations were generated that contained MurX, along with other membrane proteins (including MurG, the enzyme that generates lipid II from lipid I, Fig. 4a). The inhibition of MurX was assessed in a MurX–MurG coupled assay whereby a decrease in lipid II formation was monitored by radiochemical means. Because the nucleoside antibiotics have only been demonstrated to inhibit MurX, we made the assumption that, despite the coupled nature of the assay, it would serve to effectively measure the inhibition of MurX. The assay was initially performed by the addition of a 200 nM concentration of a given sansanmycin analogue together with UDP-[^14^C]GlcNAc and UDP-MurNAc pentapeptide (Park's nucleotide). Following quenching of the enzymatic reactions and an extraction-based work up, thin layer chromatography (TLC) and phosphorimaging were used to measure the degree of inhibition of MurX by measuring a decrease in the formation of lipid II that incorporated the [^14^C] label on the GlcNAc moiety (Fig. 4b). We were pleased to observe that all the natural products inhibited MurX to varying degrees in this assay (9–100% MurX inhibition at 200 nM, Supplementary Tables 3–5). Importantly, the most potent analogues against *Mtb* growth were also the most active MurX inhibitors in this screen (Supplementary Figs 38 and 39). In a recent report by Ishizaki *et al*., CPZEN-45, an analogue of the caprazamycin family of nucleoside antibiotics was shown not to inhibit MurX in *M. smegmatis*, but rather inhibited WecA, an enzyme involved in arabinogalactan biosynthesis in mycobacteria^40^. WecA also uses UDP-GlcNAc as a substrate but, unlike MurX, does not utilize Park's nucleotide. As such, we also assessed inhibition of WecA by the sansanmycin analogues in our assay^40^. Interestingly, we did not observe inhibition of WecA by any of the compounds and, as such, the natural product analogues appear to be selective inhibitors of lipid I formation in peptidoglycan biosynthesis.

Having established inhibition of *Mtb* MurX by the dihydrosansanmycins at a single concentration, we next focused on three analogues which showed the most potent inhibition of *Mtb* and *Mtb* MurX *in vitro* (compounds **25**, **36** and **37**). The TLC-based assay developed above was employed using a range of concentrations of a given analogue and IC_50_ values of 54, 48 and 41 nM were determined for **25**, **36** and **37**, respectively (see Fig. 4b for assay plate for compound **37**). Importantly, there was significant correlation between the IC_50_ against *Mtb* MurX and the activity against *Mtb* H37Rv *in vitro*. To unequivocally determine that our lead sansanmycin analogues were inhibiting *Mtb* MurX exclusively (and not a combination of MurX and MurG), we established a complementary fluorescence-enhancement assay which measured the incorporation of an *N*-dansylated UDP-MurNAc pentapeptide (dansylated on the diaminopimelic acid moiety) using a modification to an assay reported for the *E. coli* orthologue by Bugg and co-workers^42^. Briefly, *Mtb* mc^2^ 6230 membranes were incubated with one of the lead analogues (compounds **25**, **36** or **37**) and the *N*-dansylated UDP-MurNAc pentapeptide substrate and formation of lipid I monitored by excitation at 340 nm and detection at 530 nm. Gratifyingly, the fluorescence assay of **25** (IC_50_=30 nM), **36** (IC_50_=14 nM) or **37** (IC_50_=16 nM) demonstrated similar inhibitory potencies to those determined via the radiochemical assay. Importantly, these data verify that the compounds are selective inhibitors of *Mtb* MurX and that this is likely the mechanism of anti-mycobacterial activity. Furthermore, the development of this fluorescence assay now provides a higher throughput means to screen future *Mtb* MurX inhibitors.

### Intracellular anti-mycobacterial activity of lead analogues

Having established that the three lead analogues were potent inhibitors of *Mtb* MurX, they were next assessed for their anti-mycobacterial activity in an intracellular assay. Specifically, THP-1 macrophages were infected with *Mtb* and the inhibition of mycobacterial growth measured in the presence of a range of concentrations of **25**, **36** or **37**. Gratifyingly, all of the natural product analogues maintained anti-mycobacterial activity intracellularly with IC_50_s of 1.57, 4.33 and 0.11 μM for **25**, **36** and **37**, respectively (Supplementary Figs 31–33). This data provides encouragement that these compounds may serve as bonafide leads for future TB drug discovery efforts.

### Stability studies

We finally assessed the stability of **25**, **36** and **37** in mouse and human plasma and mouse and human liver microsomes with each of the compounds showing excellent stability, with degradation half-lives of >7 h for human and mouse plasma and >160 min for human and mouse liver microsomes (Supplementary Tables 6 and 7). Based on the potent inhibition of *Mtb* (both *in vitro* and intracellularly) and the promising stability data, these compounds now serve as exciting leads for the development of a further generation of sansanmycin derivatives with a view to developing a TB drug lead which operates through the inhibition of lipid I synthesis in *Mtb.*

## Discussion

In summary, we have described the development of a robust divergent solid-phase synthetic strategy to rapidly access a library of dihydrosansanmycin natural product analogues. A number of potent inhibitors of *Mtb* were elucidated that possess activity both *in vitro* and in macrophages infected with the organism. The compounds were shown to potently inhibit the enzyme MurX, responsible for lipid I synthesis, a key intermediate en route to peptidoglycan in *Mtb*. Despite this mechanism of action, the natural product analogues possess selective activity against *Mtb*, with little to no activity against other pathogenic bacteria that also synthesize peptidoglycan. This selectivity provides a practical advantage for the potential use of these compounds as TB drugs as well as MurX as a TB drug target. Future work in our laboratories will focus on understanding the mechanism of this selectivity. Finally, the compounds were stable in plasma and against the action of liver microsomes which, together with the potent and selective activity against *Mtb*, lays the foundation for the development of TB drug leads based around this natural product scaffold in the future.

## Methods

### Synthesis of sansanmycin analogues

For materials, methods and complete details of synthetic procedures, see Supplementary Methods. ^1^H, ^13^C and 2D NMR spectra of compounds described in this manuscript can be found as Supplementary Figs 47–148.

### *In vitro* inhibition assays *Mtb*

*Mtb* H37Rv (ATCC 27294) was grown in Middlebrook 7H9 broth medium supplemented with OADC (Difco Laboratories, Detroit, MI, USA), 0.05% glycerol and 0.05% Tween-80. Freshly seeded cultures were grown at 37 °C, for ∼14 days, to mid-exponential phase (OD_600_ 0.4–0.8) for use in the inhibition assays. The effect of dihydrosansanmycin analogues against the growth of *Mtb* H37Rv was measured by a resazurin reduction microplate assay, using the procedure previously described by Taneja and Tyagi^34^. *Mtb* grown to mid-exponential phase (OD_600_ 0.4–0.8) was diluted to OD_600_ 0.002 in 7H9S medium (Middlebrook 7H9 with 10% albumin, dextrose, catalase (ADC, Moregate Biotech), 0.05% glycerol, 0.05% Tween-80, 1% tryptone; 96-well microtiter plates were set up with 100 μl inhibitors, serially diluted into 7H9S. Diluted *Mtb* (100 μl, representing ∼2 × 10^4^ CFU ml^−1^) was added to each well. Plates were incubated for 5 days at 37 °C in a humidified incubator before the addition of a 0.02% resazurin solution (30 μl) and 20% Tween-80 (12.5 μl) to each well. Sample fluorescence was measured after 24 h on a BMG Labtech Polarstar Omega instrument with an excitation wavelength of 530 nm and emission at 590 nm. Changes in fluorescence relative to positive control wells (*Mtb* H37Rv with no inhibitor) minus negative control wells (no *Mtb* H37Rv) were plotted for determination of MIC_50_ values. Rifampicin (R3501) and isoniazid (I3377) were purchased from Sigma-Aldrich.

### *Mtb* inhibition using infected macrophage culture

*Mtb* H37Ra strain (ATCC 25177) was grown in Middlebrook 7H9 broth medium supplemented with OADC (Difco Laboratories), 0.05% glycerol and 0.05% Tween-80. Freshly seeded cultures were grown at 37 °C, for ∼14 days, to mid-exponential phase (OD_600_ 0.4–0.8) for use in the inhibition assays and used to infect a human macrophage-like cell line (THP-1; ATCC TIB-202). THP-1 stocks were maintained at a culture density between 1 × 10^5^ and 1 × 10^6^ cells ml^−1^ in RPMI-1640 medium (with phenol red, 25 mM HEPES and 2 mM L-glutamine) supplemented with 10% FBS (FBS-500) and 0.05 mM β-mercaptoethanol. THP-1 cells were plated in 96-well tissue culture plates (Costar 3903; Corning) at a density of 1 × 10^5^ cells/well with phorbol myristic acetate (PMA; 100 nM) added. THP-1 cells were left to differentiate for 48 h at 37 °C at 5% CO_2_. A cell suspension of sonicated *Mtb* H37Ra in RPMI-1640 cell culture medium was used to infect differentiated THP-1 cells at a multiplicity of infection of 5 for 4 h at 37 °C at 5% CO_2_. Supernatant was then removed from all wells, THP-1 cells were washed with 200 μl phosphate buffered saline (PBS) three times and were subsequently replenished with fresh RPMI-1640 cell culture medium and incubated for a further 24 h at 37 °C and 5% CO_2_. The sansanmycin analogues were diluted in fresh RPMI-1640 cell culture medium and added to corresponding wells. Positive controls were dissolved in 100% dimethyl sulfoxide (DMSO) and diluted in 7H9 broth (Difco Becton Dickinson) with 10% ADC, 0.05% glycerol and 0.05% Tween 80 before adding to the wells. After 72 h of incubation at 37 °C at 5% CO_2_, tissue culture medium containing the test compound was removed from the wells; the cells were washed with 200 μl PBS, and then lysed with sterile water containing 0.1% Triton X. Cell lysates were serially diluted, 1:10, and plated on Middlebrook 7H11/OADC (283010; Difco) agar through to 1:10,000 dilution. Agar plates were then incubated at 37 °C for 3–4 weeks, after which the bacteria colonies were counted and CFU ml^−1^ of cell lysates were determined.

### Screen against a panel of bacterial strains

The effect of dihydrosansanmycin analogues against 15 clinically relevant Gram-positive and Gram-negative bacteria strains was evaluated using a procedure by Wong *et al*.^35^. Specifically, the screening panel consisted of six Gram-positive strains (BSL1: *Bacillus subtilis* 168, *Staphylococcus epidermidis* [ATCC 14990], *Enterococcus faecium* [ATCC 6569], *Listeria ivanovii* [BAA-139]); BSL2: *S. aureus* [ATCC 29213], methicillin-resistant *S. aureus* (MRSA) [BAA-44]) and nine Gram-negative strains (BSL1: *Escherichia coli* K12 [BW 25113], *Acinetobacter baumanii* [NCIMB 12457], *Enterobacter aerogenes* [ATCC 35029], *Ochrobactrum anthropi* [ATCC 49687], *Providencia alcalifaciens* [ATCC 9886]; BSL2: *Yersinia pseudotuberculosis* [IP2666 pIBI], *Pseudomonas aeruginosa* [ATCC 27835], *Salmonella typhimurium* LT2, *Vibrio cholerae* O1 [biotype El Tor A1552]). All staphylococcal strains, *L. ivanovii* and *E. faecium* were cultured in 10 ml of tryptic soy broth (17 g tryptone, 3 g soytone, 2.5 g dextrose, 5 g NaCl and 2.5 g dipotassium phosphate in 1 l distilled water; pH 7.5). *P. alcalifaciens*, *O. anthropi*, *E. aerogenes* and *A. baumanii* were grown in nutrient broth (Difco, USA), while *B. subtilis*, *E. coli*, *V. cholerae*, *S. typhimurium*, *P. aeruginosa* and *Y. pseudotuberculosis* cultures were grown in Luria Broth (10 g tryptone, 5 g yeast extract and 10 g NaCl in 1 l distilled water; pH 7.5). All three media were autoclaved at 121 °C for 30 min. Inoculated cultures were grown overnight in a shaker (200 r.p.m.; 30 °C).

Overnight saturated cell cultures of pathogenic strains were diluted 1:1,000 with fresh media and 30 μl of culture dispensed into each well of sterile clear 384-well plates. A total of 300 nl of DMSO prefraction stock solutions were pinned into screening plates using a Perkin Elmer Janus MDT robot. After inoculation, screening plates were stacked in a plate reader/shaker (Perkin Elmer EnVision) and OD_600_ readings taken once per hour for 24 h. Computer generated growth curves for serially diluted pure compounds (with the top final screening concentration of 100 μM) were used to determine MIC values by correlating the OD_600_ reading at the pre-exponential phase of the bacteria to the concentrations in individual wells.

### MurX enzyme inhibition assays

Materials: UDP-*N*-acetyl-D-glucosamine [glucosamine-^14^C(U)] ([^14^C]UDP-GlcNAc, Specific activity 300 mCi/mmol) was obtained from American Radio Chemicals, UDP-MurNAc-pentapeptide was obtained from BacWAN, University of Warwick, Coventry, UK (www.warwick.ac.uk/bacwan). Dansyl-labelled UDP-MurNAc-pentapeptide was synthesized from UDP-MurNAc-pentapeptide as described below. TLC Silica gel 60 F_254_ plates were procured from Merck (Germany). All other chemicals used were at least analytical grade and were obtained from Sigma-Aldrich. *Mtb* mc^2^ 6230 was a generous gift from Dr William Jacobs, Albert Einstein College of Medicine, New York.

### Membrane preparation from *Mtb* mc^2^ 6230

*Mtb* mc^2^ 6230 was grown in 7H9 medium (supplemented with 0.5% (v/v) oleic acid, 0.5% (w/v) albumin, 0.2% (w/v) dextrose, 24 μg ml^−1^ D-pantothenate and 0.2% casamino acids). Washed cells were resuspended in Buffer A (50 mM MOPS pH 7.9, 5 mM MgCl_2_, 5 mM DTT, 10% glycerol (v/v)), at 2 ml g^−1^ of cells, and disrupted by probe sonication on ice with a Sanyo Soniprep 150 (10 cycles of 60 s on and 90 s off). The whole cell lysates were centrifuged at 5,000*g* for 20 min at 4 °C. The supernatant was further centrifuged at 100,000*g* (for 1 h at 4 °C) in an Optima TLX Ultracentrifuge (Beckman). The membrane-enriched pellets were washed in Buffer A followed by ultracentrifugation at 100,000*g*. The washed pellets were resuspended in Buffer A, divided into aliquots and frozen at −80 °C. The protein concentration of the membrane-enriched fraction was estimated using a BCA protein assay kit (Pierce).

### Preparation of dansylated UDP-MurNAc pentapeptide

Synthesis and purification UDP-MurNAc-pentapeptide was achieved by chemoenzymatic recapitulation of the cytoplasmic synthetic pathway *in vitro*^43^. Desalted UDP-MurNAc-pentapeptide in sterile water was mixed with an equal volume of acetone and allowed to react overnight with a 42-fold molar ratio of dansyl chloride with stirring. The reaction was quenched with a 10-fold molar excess of Tris-Cl pH 9 to dansyl chloride before rotary evaporation to remove solvents. Dried products were resuspended in 1 ml sterile water and purified by size exclusion chromatography on a Superdex peptide 10/300 column (GE Healthcare) pre-equilibrated with 1.5 CV 0.1 M ammonium bicarbonate. Dansylated UDP-MurNAc-pentapeptide is the first peak to elute in this procedure with a characteristic absorbance at 340 and 280 nm. Fractions containing the required product were freeze dried 4 times and resuspended in a small volume of sterile water. Quantification and purity were confirmed by absorbance and mass spectrometry, respectively.

### Radiochemical *Mtb* MurX inhibition assay

Assay mixtures (200 μl) contained 50 mM MOPS pH 7.9, 5 mM MgCl_2_, 5 mM DTT, 10% glycerol (v/v), 0.1% CHAPS, 100 μM ATP, 25 μM UDP-MurNAc-pentapeptide, 0.5 μM [^14^C] UDP-GlcNAc, and varying concentrations of inhibitor (initial screening was carried out at a single concentration of 200 nM and the most potent compounds were screened at a range of concentrations to determine IC_50_ values, Supplementary Figs 34–37). Reactions were initiated by the addition of 400–500 μg of *Mtb* mc^2^ 6230 membrane protein and incubated at 37 °C for 1 h. Reactions were stopped by the addition of 6 ml of chloroform/MeOH (2:1), followed by low speed centrifugation and the organic extract was moved to a second tube. Extracts were back washed twice [once with water (800 μl) and then with chloroform/MeOH/water (3:47:48)], evaporated to dryness under a nitrogen stream, and dissolved in chloroform/MeOH (2:1 v/v). An aliquot was subjected to liquid scintillation counting (LS 6500, Beckman Coulter); a second aliquot was subjected to TLC (Silica gel 60 F_254_) developed in chloroform/MeOH/water/ammonium hydroxide (88:48:10:1). Distribution of radioactivity was detected by phosphorimaging (Typhoon TRIO, Amersham Biosciences) and quantified with ImageQuant TL v2005 software (Amersham Biosciences). IC_50_ values were calculated by using GraFit Software (Version 5.0.13).

### Fluorescence-based *Mtb* MurX inhibition assay

Continuous fluorescence MurX assays (100 μl in volume) were carried out *in vitro* at 25 °C in an assay buffer consisting of 83 mM Tris pH 7.5, 21 mM MgCl_2_, 6% glycerol, 0.1% TritonX-100, 15 μM dansylated UDP-MurNAc-pentapeptide, 40 μg ml^−1^ polyisoprenyl phosphate and varying concentrations of inhibitor^42^. Reactions were initiated by the addition of 60–70 μg of *Mtb* mc^2^ 6230 membrane protein and fluorescence was monitored at 340 and 530 nm for excitation and emission, respectively. Assays were carried out in duplicate and IC_50_ values were calculated using GraFit Software (Version 5.0.13, Supplementary Figs 42–44). A further inhibition assay was carried out for analogue **37** at an increased concentration of polyisoprenylphosphate (160 μg ml^−1^, Supplementary Fig. 45). Assays were also performed at a fixed concentration of UDP-MurNAc-pentapeptide (15 μM) and a range of exogenous polyisoprenylphosphate concentrations. This enabled the determination of the apparent *K*_m_ and *V*_max_ values with respect to the polyisoprenylphosphate substrate. The endogenous polyisoprenylphosphate concentration in the extracted membrane was calculated to be 0.28 μg mg^−1^ of membrane protein.

### Stability studies in human and mouse plasma

Human blood from nonidentifiable volunteer donors was sourced from the Australian Red Cross Blood Service under a supply agreement approved by the Monash Human Research Ethics Committee. Mouse blood was collected in-house under tissue harvesting protocols approved by the Monash Institute of Pharmaceutical Sciences Animal Ethics Committee. Pooled human and Swiss outbred mouse plasma samples were thawed and spiked with test compound solutions prepared in DMSO/acetonitrile/water (20:40:40) to provide a final compound concentration of 1,000 ng ml^−1^ and final DMSO and acetonitrile concentrations of 0.2% and 0.4% (v/v), respectively. Plasma was vortex mixed and aliquots (50 μl) were transferred to fresh microcentrifuge tubes and incubated at 37 °C. At various time points over the 6 h incubation period, duplicate plasma samples were removed and immediately snap-frozen in dry ice. All samples were stored frozen at −80 °C until analysis by LC–MS. Samples were processed by protein precipitation using a two-fold excess of acetonitrile followed by centrifugation. Analysis of the supernate was conducted using a Waters (Milford, MA) Acquity UPLC coupled to a Waters Micromass Quattro Premier mass spectrometer operated in positive electrospray ionization mode with multiple reaction monitoring. The cone voltage was 45 V, collision energy was 30, 30 and 40 V for **25**, **36** and **37**, respectively, and *m*/*z* transitions were 782.30>126.06 (**25**), 826.32>170.16 (**36**) and 888.39>126.06 (**37**). Processed samples (3 μl) were injected onto a Supelco Ascentis Express RP Amide column (50 × 2.1 mm, 2.7 μm) and analytes eluted using a water/acetonitrile (each containing 0.05% formic acid) gradient over 4 min with a flow rate was 0.4 ml min^−1^. Sample concentrations were quantitated by comparison to a calibration curve prepared in blank human or mouse plasma.

### Stability studies in human and mouse liver microsomes

The metabolic stability was assessed by incubating each test compound (0.5 μM) in duplicate with human and mouse liver microsomes (XenoTech, Kansas City, KS) at 37 °C and 0.4 mg ml^−1^ microsomal protein. The metabolic reaction was initiated by the addition of an NADPH-regenerating system and quenched at various time points over a 60 min incubation period by the addition of acetonitrile containing diazepam as internal standard. Control samples (containing no NADPH) were included and quenched at 2, 30 and 60 min to monitor for potential degradation in the absence of cofactor. Samples were centrifuged and the supernatant analysed by LCMS. Analysis was conducted using a Waters Acquity UPLC coupled to a Waters Xevo G2 QTOF mass spectrometer operated in positive electrospray ionization MS^E^ mode with a cone voltage of 30 V. Samples (5 μl) were injected onto an Ascentis Express Amide column (50 × 2.1 mm, 2.7 μm) and eluted using a water/acetonitrile (both containing 0.05% formic acid) gradient over 4 min at a flow rate of 0.4 ml min^−1^. Degradation rate half-lives and *in vitro* intrinsic clearance values were determined from the first order degradation profiles.

### Data availability

Data supporting the findings of this study are available within the article and its Supplementary Information files and from the corresponding author upon reasonable request.

## Additional information

**How to cite this article:** Tran, A. T. *et al*. Sansanmycin natural product analogues as potent and selective anti-mycobacterials that inhibit lipid I biosynthesis. *Nat. Commun.* **8,** 14414 doi: 10.1038/ncomms14414 (2017).

**Publisher's note**: Springer Nature remains neutral with regard to jurisdictional claims in published maps and institutional affiliations.

## Supplementary Material

Supplementary Information Supplementary Figures, Supplementary Tables, Supplementary Methods, Supplementary References
