# Supplementary material for: Sansanmycin natural product analogues as potent and selective anti-mycobacterials that inhibit lipid I biosynthesis
Source: Nat Commun. 2017 Mar 1;8:14414. doi: 10.1038/ncomms14414 (PMC5337940; doi:10.1038/ncomms14414)
Supplement: Supplementary Information — Supplementary Figures, Supplementary Tables, Supplementary Methods, Supplementary References [file ncomms14414-s1.pdf]

SUPPLEMENTARY FIGURES

Raw inhibition data of *Mtb* H37Rv by dihydrosansanmycin analogues

The data below represent exemplar data from a single experiment performed in triplicate

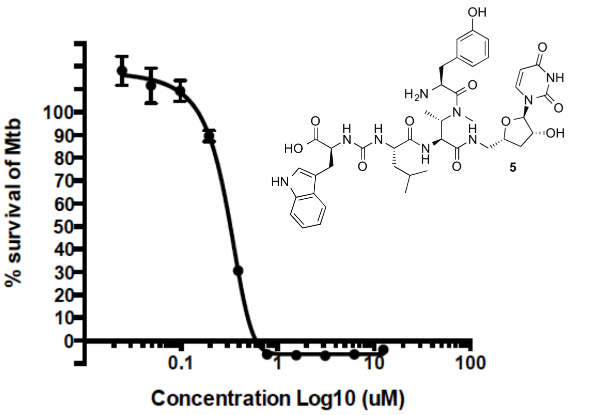

Supplementary Figure 1. Inhibition of *Mtb* H37Rv by dihydrosansanmycin analogue **5**.

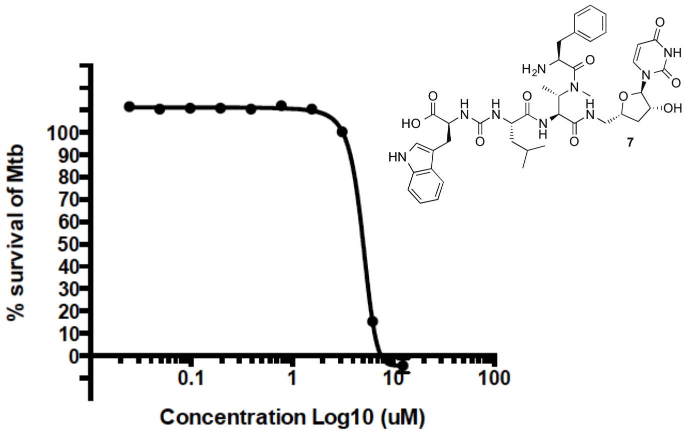

Supplementary Figure 2. Inhibition of *Mtb* H37Rv by dihydrosansanmycin analogue **7**.

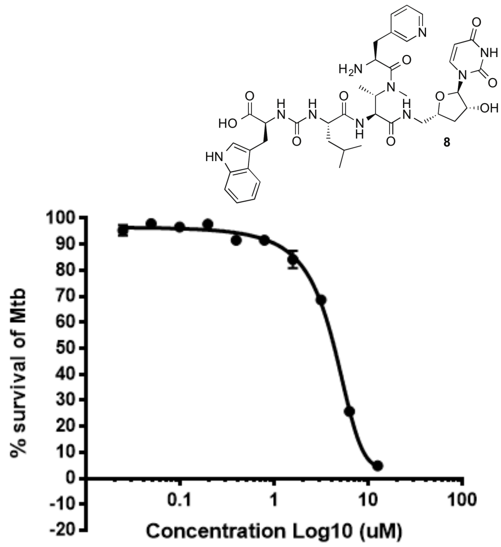

Supplementary Figure 3. Inhibition of *Mtb* H37Rv by dihydrosansanmycin analogue **8**.

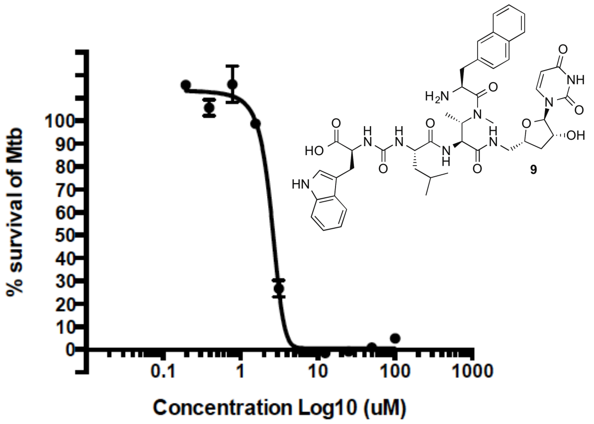

Supplementary Figure 4. Inhibition of *Mtb* H37Rv by dihydrosansanmycin analogue **9**.

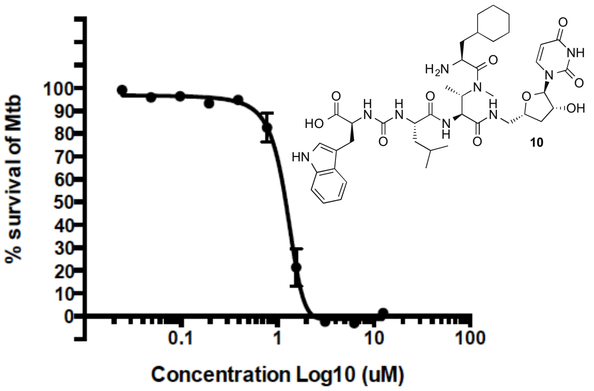

Supplementary Figure 5. Inhibition of *Mtb* H37Rv by dihydrosansanmycin analogue **10**.

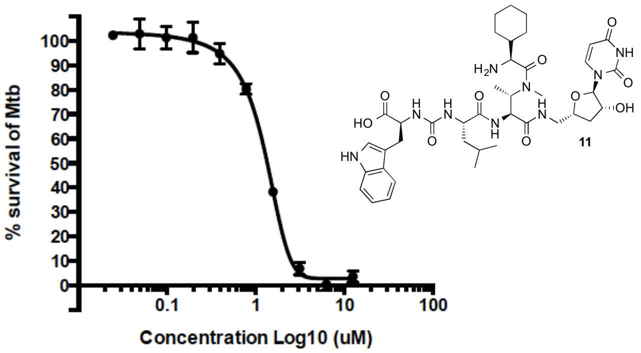

Supplementary Figure 6. Inhibition of *Mtb* H37Rv by dihydrosansanmycin analogue **11**.

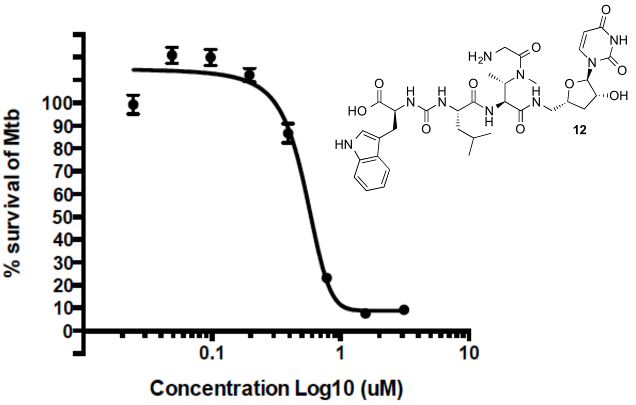

Supplementary Figure 7. Inhibition of *Mtb* H37Rv by dihydrosansanmycin analogue **12**.

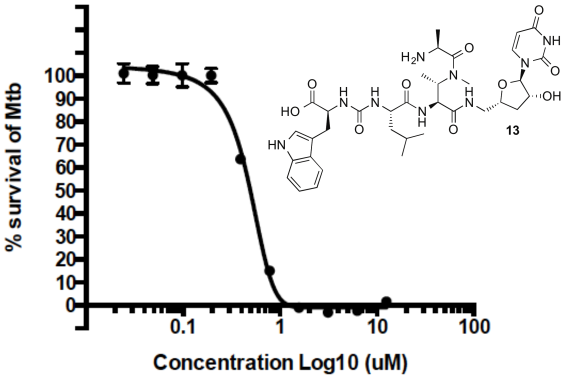

Supplementary Figure 8. Inhibition of *Mtb* H37Rv by dihydrosansanmycin analogue **13**.

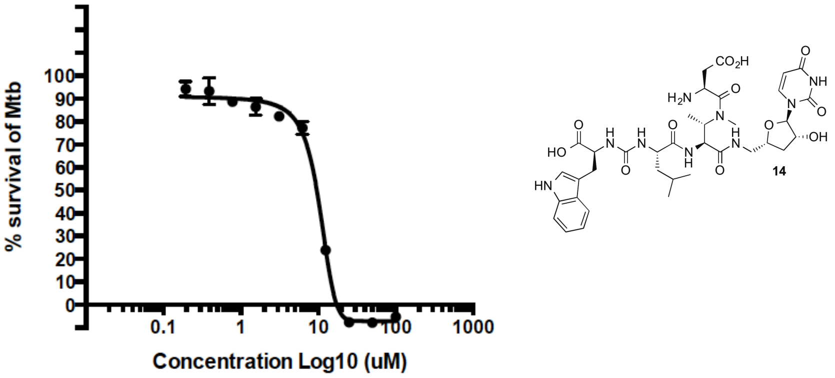

Supplementary Figure 9. Inhibition of *Mtb* H37Rv by dihydrosansanmycin analogue **14**.

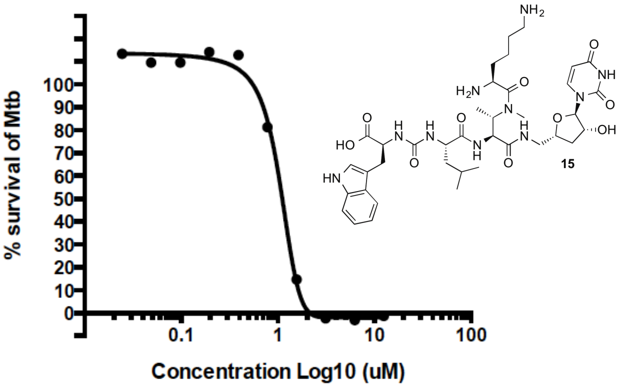

Supplementary Figure 10. Inhibition of *Mtb* H37Rv by dihydrosansanmycin analogue **15**.

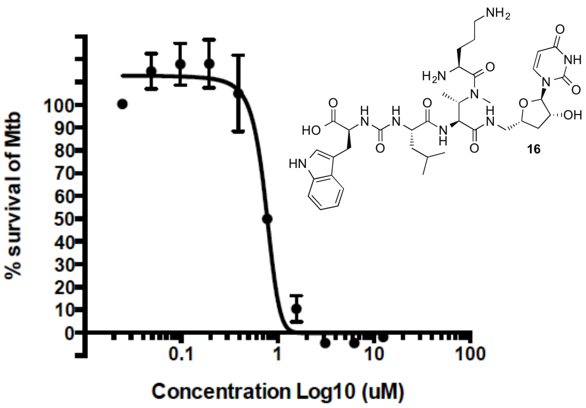

Supplementary Figure 11. Inhibition of *Mtb* H37Rv by dihydrosansanmycin analogue **16**.

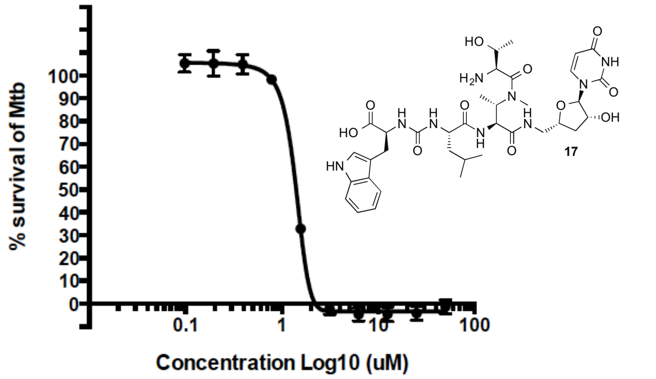

Supplementary Figure 12. Inhibition of *Mtb* H37Rv by dihydrosansanmycin analogue **17**.

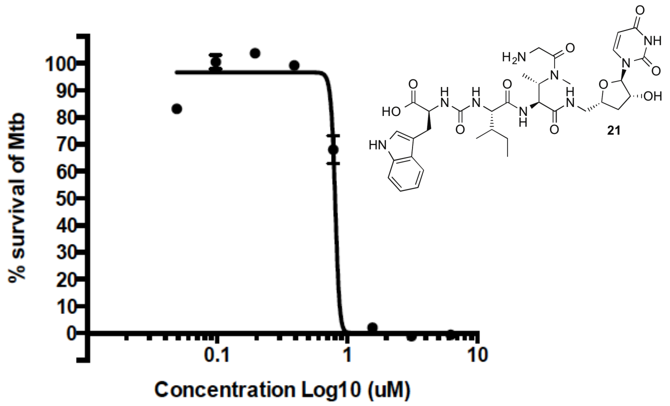

Supplementary Figure 13. Inhibition of *Mtb* H37Rv by dihydrosansanmycin analogue **21**.

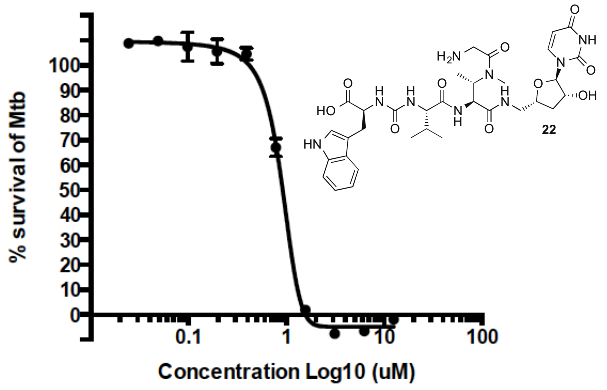

Supplementary Figure 14. Inhibition of *Mtb* H37Rv by dihydrosansanmycin analogue **22**.

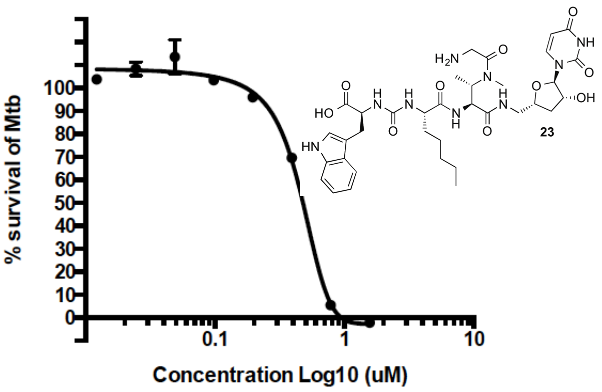

Supplementary Figure 15. Inhibition of *Mtb* H37Rv by dihydrosansanmycin analogue **23**.

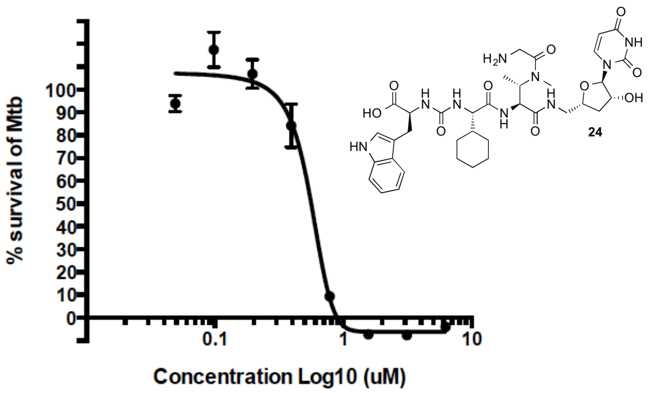

Supplementary Figure 16. Inhibition of *Mtb* H37Rv by dihydrosansanmycin analogue **24**.

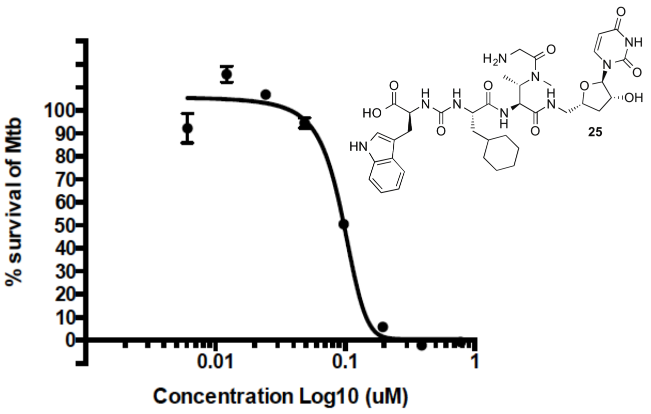

Supplementary Figure 17. Inhibition of *Mtb* H37Rv by dihydrosansanmycin analogue **25**.

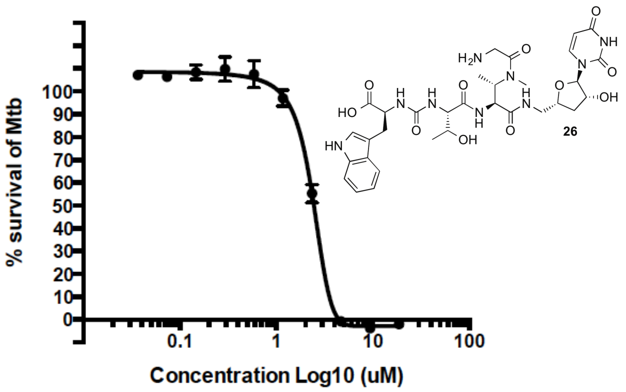

Supplementary Figure 18. Inhibition of *Mtb* H37Rv by dihydrosansanmycin analogue **26**.

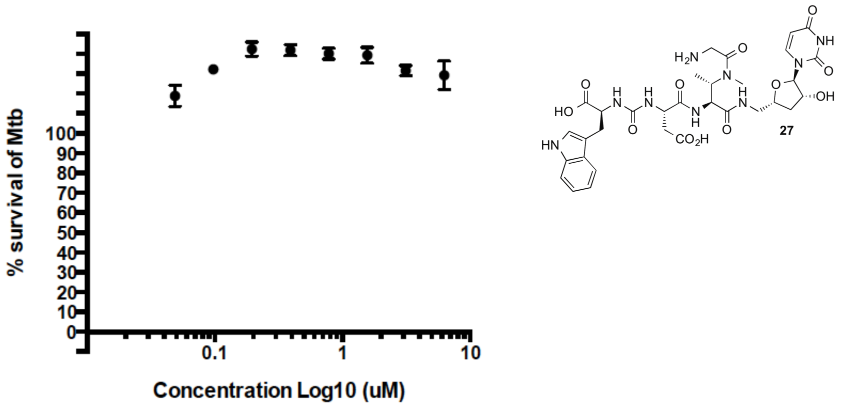

Supplementary Figure 19. Inhibition of *Mtb* H37Rv by dihydrosansanmycin analogue **27**.

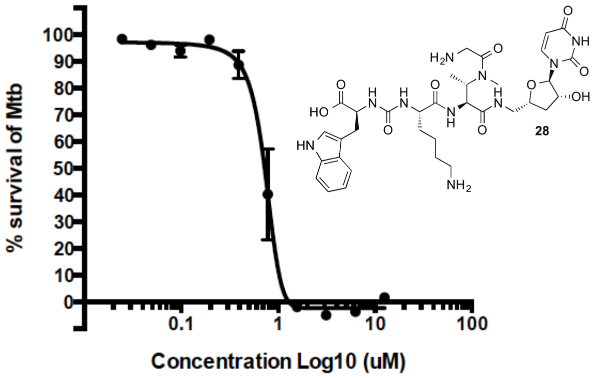

Supplementary Figure 20. Inhibition of *Mtb* H37Rv by dihydrosansanmycin analogue **28**.

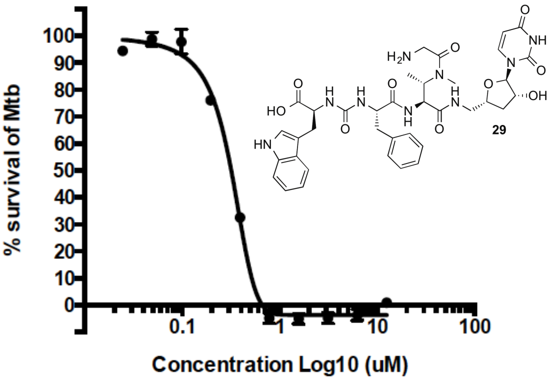

Supplementary Figure 21. Inhibition of *Mtb* H37Rv by dihydrosansanmycin analogue **29**.

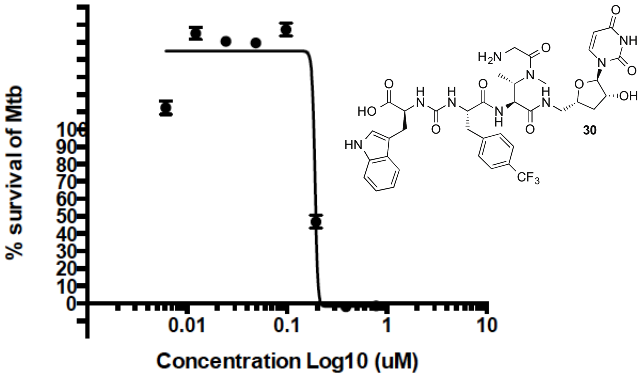

Supplementary Figure 22. Inhibition of *Mtb* H37Rv by dihydrosansanmycin analogue **30**.

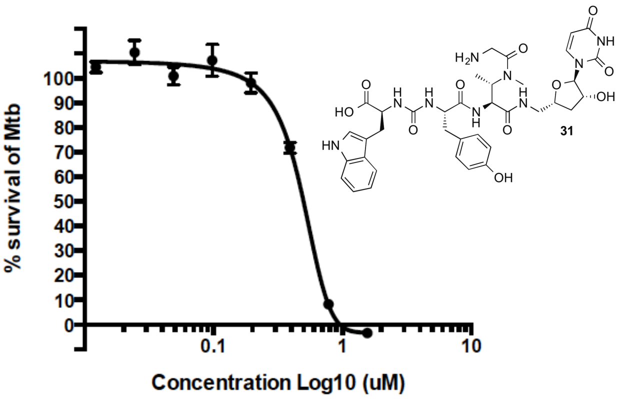

Supplementary Figure 23. Inhibition of *Mtb* H37Rv by dihydrosansanmycin analogue **31**.

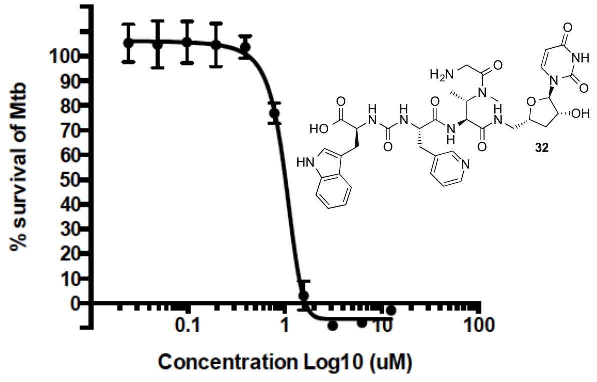

Supplementary Figure 24. Inhibition of *Mtb* H37Rv by dihydrosansanmycin analogue **32**.

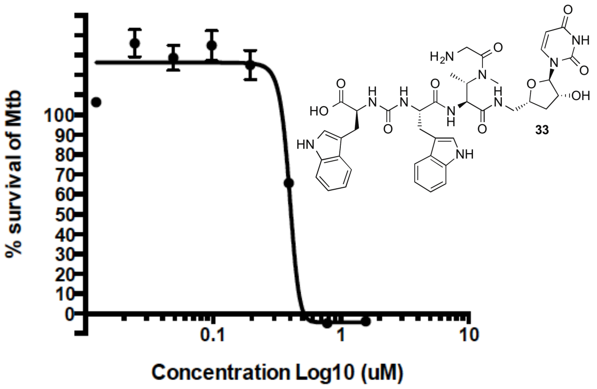

Supplementary Figure 25. Inhibition of *Mtb* H37Rv by dihydrosansanmycin analogue **33**.

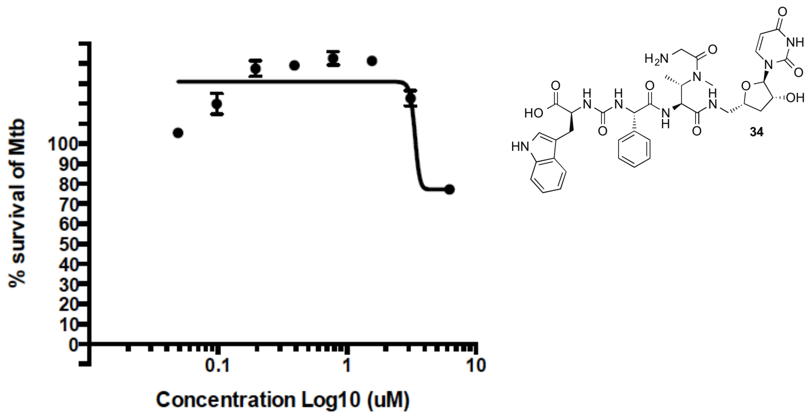

Supplementary Figure 26. Inhibition of *Mtb* H37Rv by dihydrosansanmycin analogue **34**.

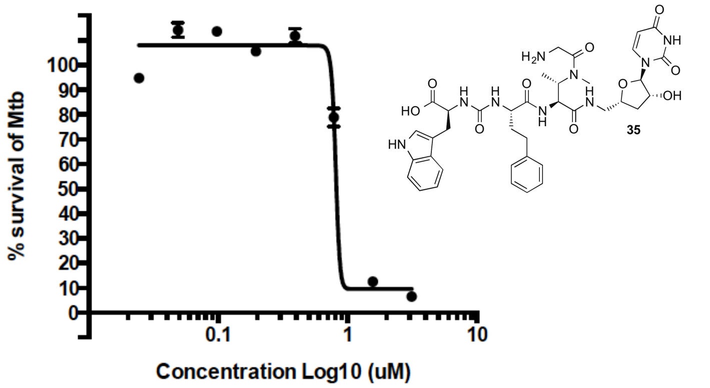

Supplementary Figure 27. Inhibition of *Mtb* H37Rv by dihydrosansanmycin analogue **35**.

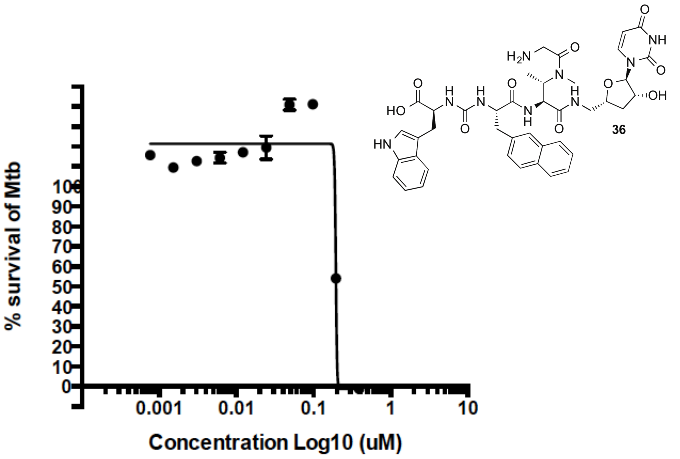

Supplementary Figure 28. Inhibition of *Mtb* H37Rv by dihydrosansanmycin analogue **36**.

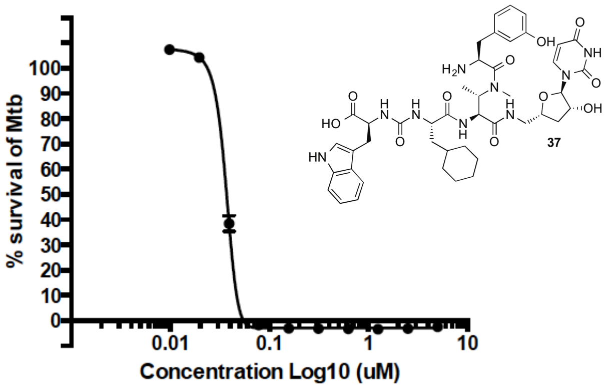

Supplementary Figure 29. Inhibition of *Mtb* H37Rv by dihydrosansanmycin analogue **37**.

Raw inhibition data of *Mtb* H37Rv by dihydrosansanmycin analogues: Effect of C-4’ stereochemistry on anti-mycobacterial activity

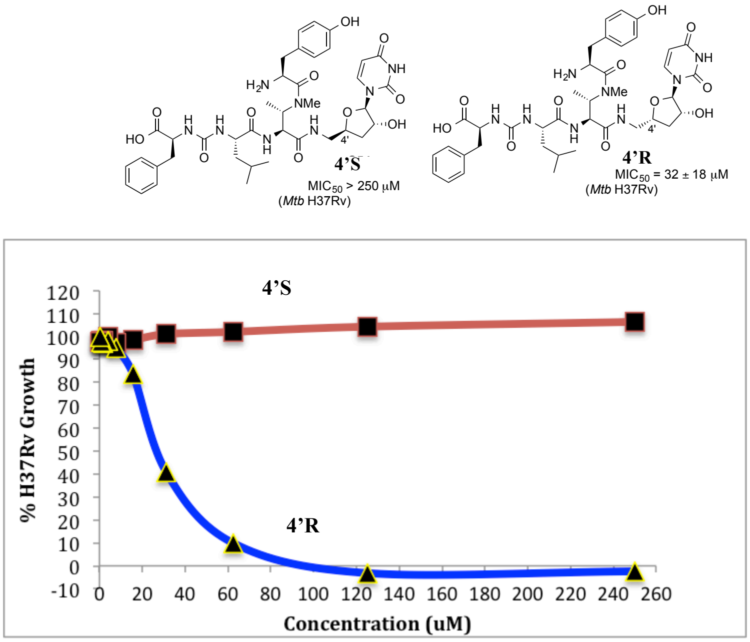

Supplementary Figure 30. Inhibition of *Mtb* H37Rv by dihydrosansanmycin analogues with differing 4’ stereochemistry

Intracellular anti-mycobacterial activity of dihydrosansanmycin analogues 25, 36 and 37

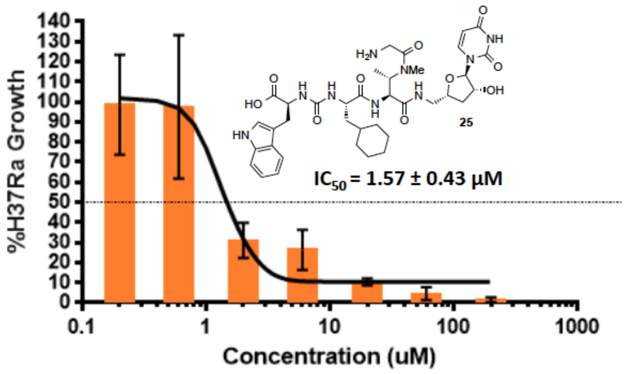

Supplementary Figure 31. Inhibition of *Mtb* H37Ra in THP-1 cells by dihydrosansanmycin analogue **25**. IC_{50} represents average of 2 independent experiments, each performed in triplicate.

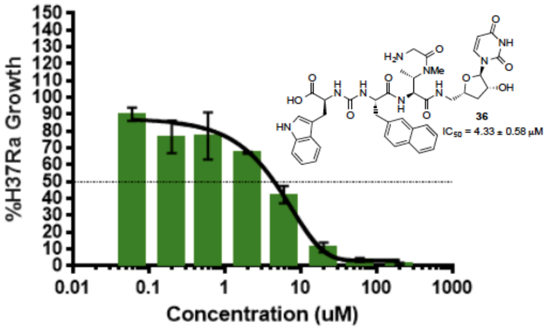

Supplementary Figure 32. Inhibition of *Mtb* H37Ra in THP-1 cells by dihydrosansanmycin analogue **36**. IC_{50} represents average of 2 independent experiments, each performed in triplicate.

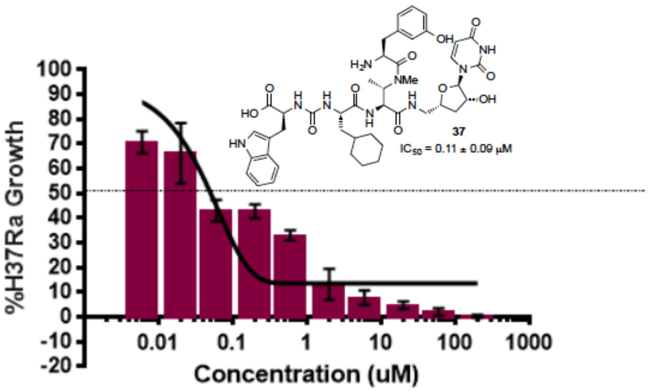

Supplementary Figure 33. Inhibition of *Mtb* H37Ra in THP-1 cells by dihydrosansanmycin analogue **37**. IC_{50} represents average of 2 independent experiments, each performed in triplicate.

MurX enzyme inhibition assays

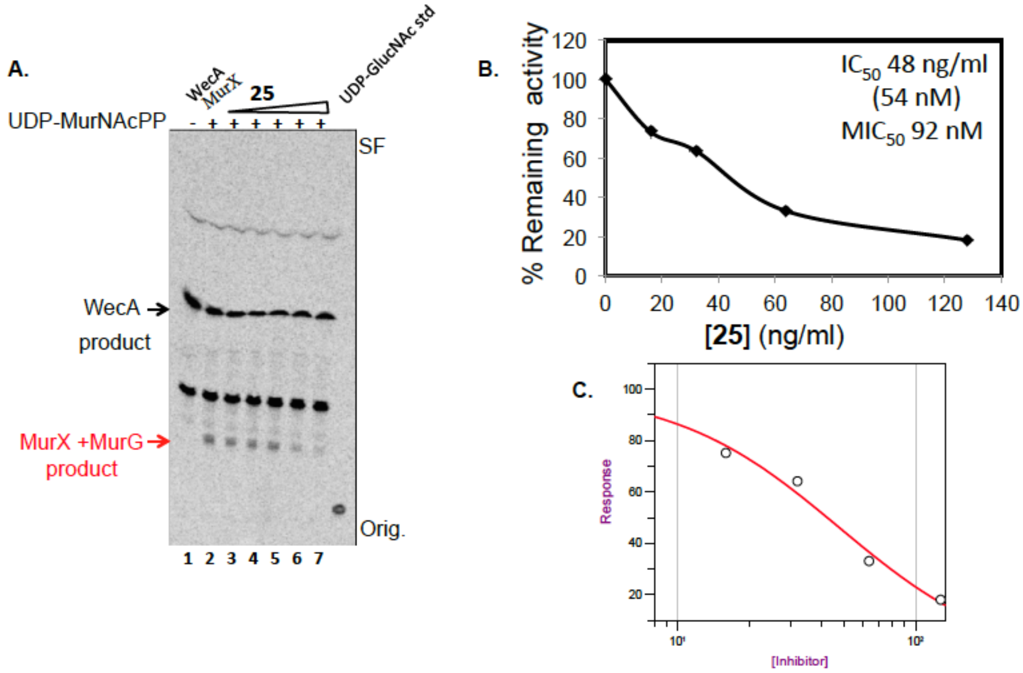

Supplementary Figure 34. TLC assay from *Mtb* mc[2] 6230 membranes for the inhibition of MurX by dihydrosansanmycin analogue **25**. **A**: TLC; **B**: Raw dose-response curve for inhibition of MurX by **25**; **C**: Log-transformed dose-response curve for inhibition of MurX by **25**

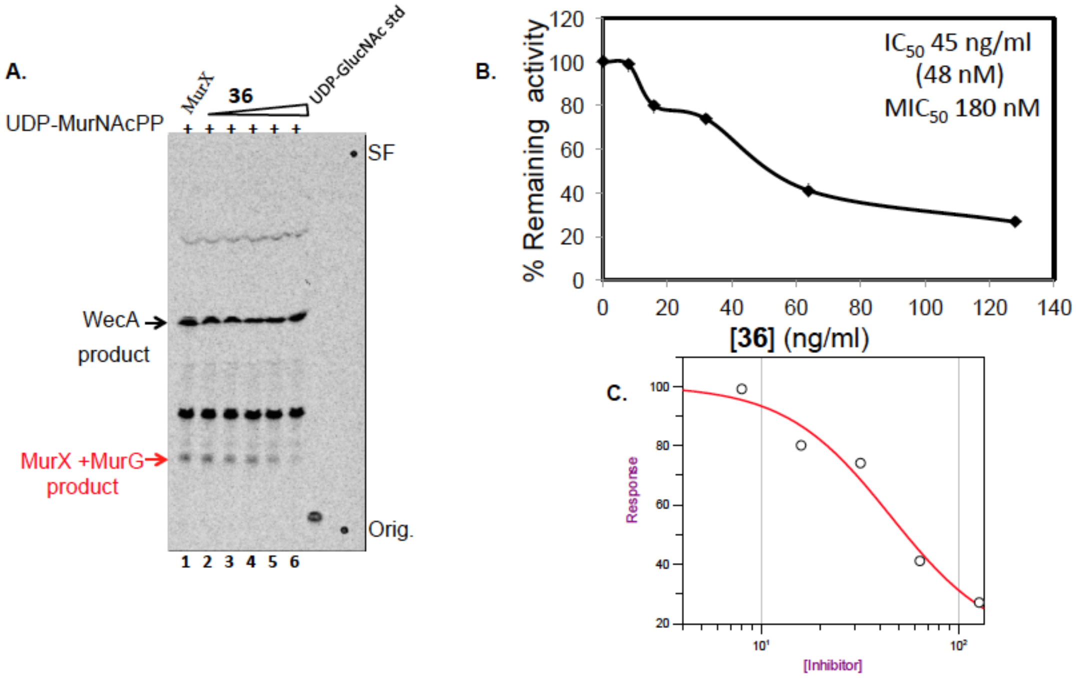

Supplementary Figure 35. TLC assay from *Mtb* mc[2] 6230 membranes for the inhibition of MurX by dihydrosansanmycin analogue **36**. **A**: TLC; **B**: Raw dose-response curve for inhibition of MurX by **36**; **C**: Log-transformed dose-response curve for inhibition of MurX by **36**

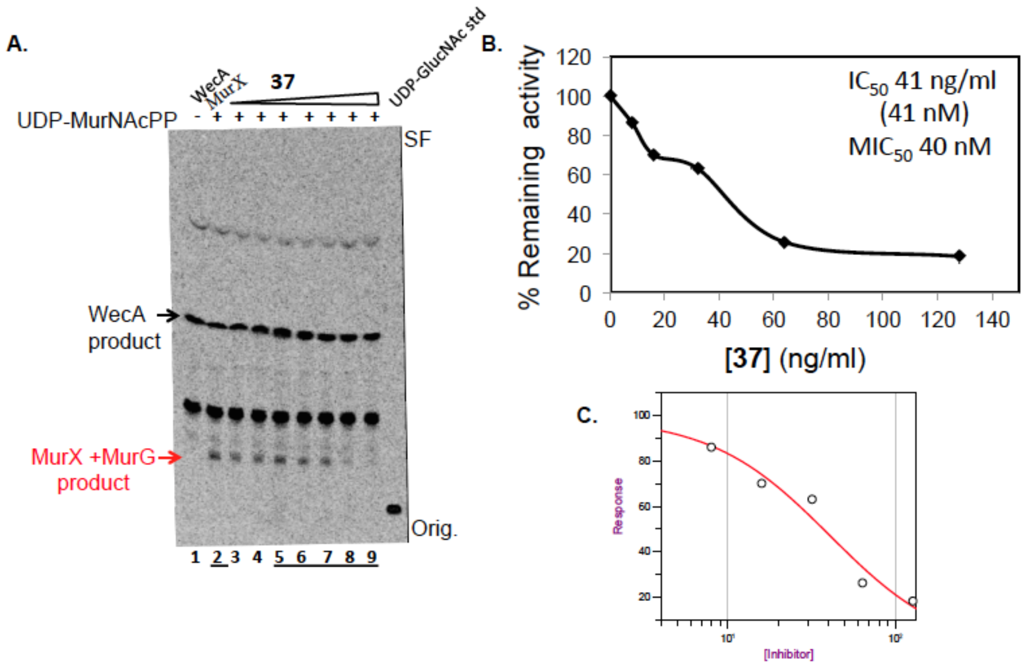

Supplementary Figure 36. TLC assay from *Mtb* mc[2] 6230 membranes for the inhibition of MurX by dihydrosansanmycin analogue **37**. **A**: TLC; **B**: Raw dose-response curve for inhibition of MurX by **37**; **C**: Log-transformed dose-response curve for inhibition of MurX by **37**

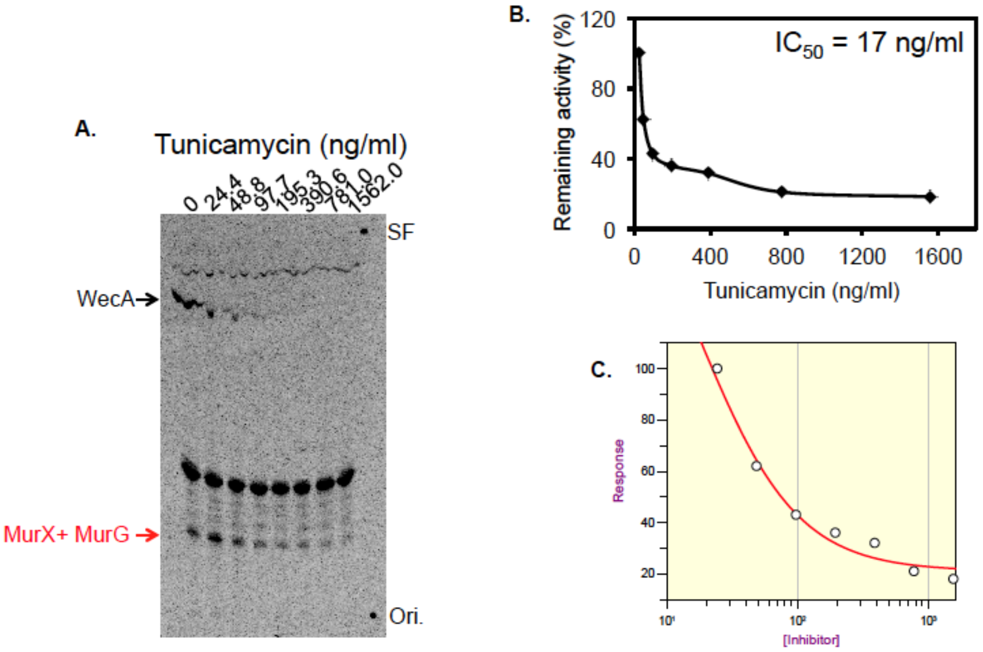

Supplementary Figure 37. TLC assay from *Mtb* mc[2] 6230 membranes for the inhibition of MurX by dihydrosansanmycin analogue tunicamycin. **A**: TLC; **B**: Raw dose-response curve for inhibition of MurX by tunicamycin; **C**: Log-transformed dose-response curve for inhibition of MurX by tunicamycin

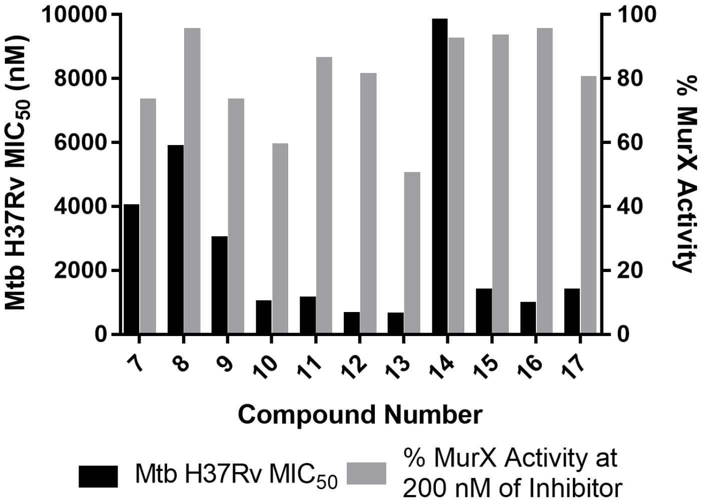

Supplementary Figure 38. Histogram for compounds **7-17** showing the relationship between % MurX activity (at 200 nM of inhibitor) and *Mtb* H37Rv growth inhibition

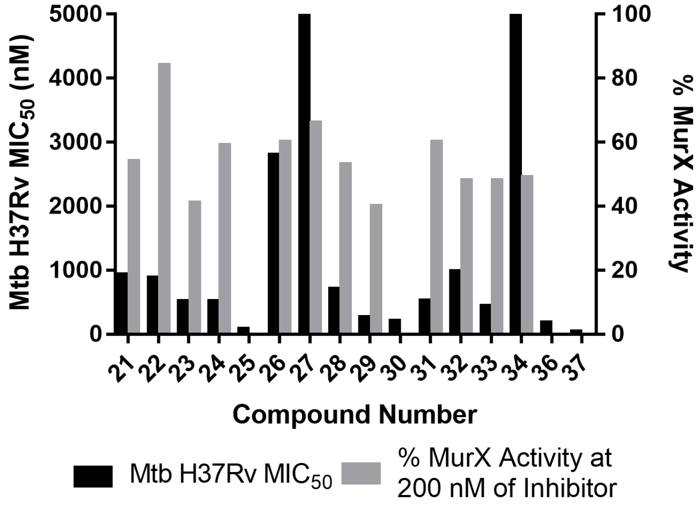

Supplementary Figure 39. Histogram for compounds **21-37** showing the relationship between % MurX activity (at 200 nM of inhibitor) and *Mtb* H37Rv growth inhibition

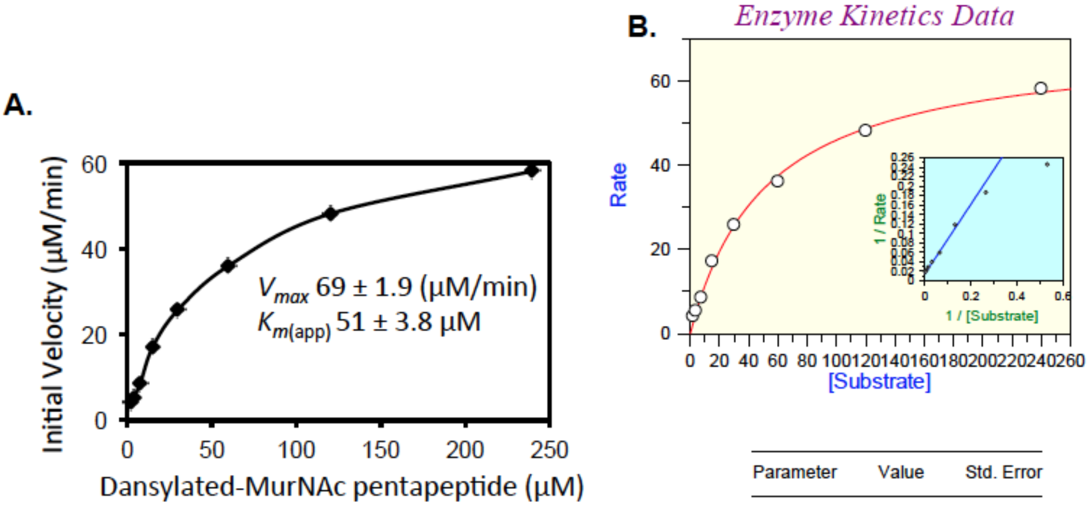

Parameter	Value	Std. Error
Vmax	69.4452	1.9135
Km	51.1991	3.8015

Supplementary Figure 40. Evaluation of kinetic parameters of MurX with varied UDP-MurNAc pentapeptide concentrations. **A**: Michaelis-Menten plot; **B**: Raw enzyme kinetics data. Data represents average of two independent experiments.

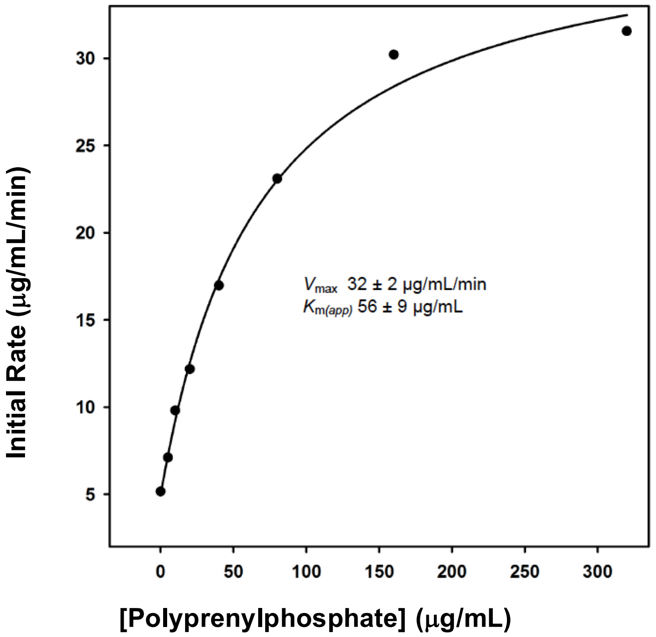

Supplementary Figure 41. Evaluation of kinetic parameters of MurX with respect to varied polyprenylphosphate concentration. Data represents average of two independent experiments.

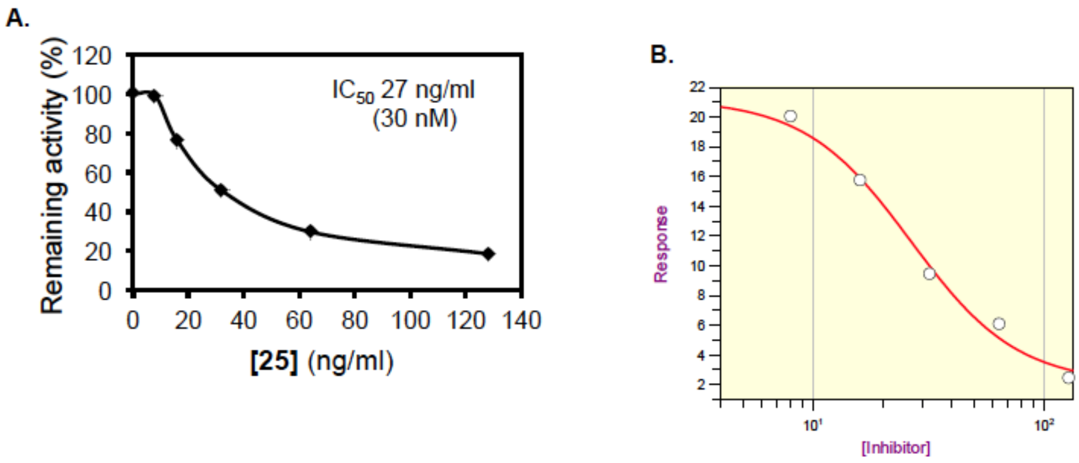

Parameter	Value	Std. Error
Y Range	19.1237	2.0396
IC 50	26.5987	3.9570
Slope factor	1.8870	0.4685
Background	2.0735	1.5716

Supplementary Figure 42. Fluorescence assay from *Mtb* mc^2 6230 membranes for the inhibition of MurX by dihydrosansanmycin analogue **25**. **A**: Raw dose-response curve for inhibition of MurX by **25**; **B**: Log-transformed dose-response curve for inhibition of MurX by **25**. Data presented is the average of two independent experiments.

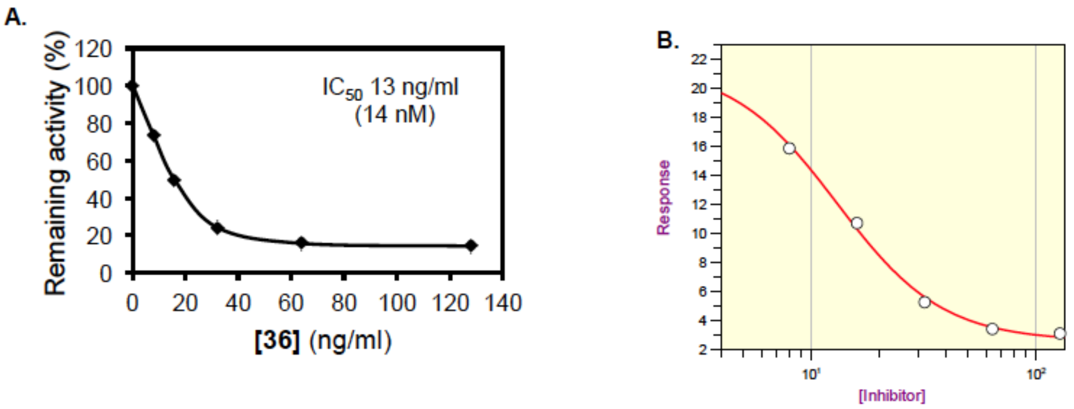

Parameter	Value	Std. Error
Y Range	19.0215	0.6946
IC 50	13.0274	0.7798
Slope factor	1.8497	0.1915
Background	2.5656	0.4792

Supplementary Figure 43. Fluorescence assay from *Mtb* mc^2 6230 membranes for the inhibition of MurX by dihydrosansanmycin analogue **36**. **A**: Raw dose-response curve for inhibition of MurX by **36**; **B**: Log-transformed dose-response curve for inhibition of MurX by **36**. Data presented is the average of two independent experiments.

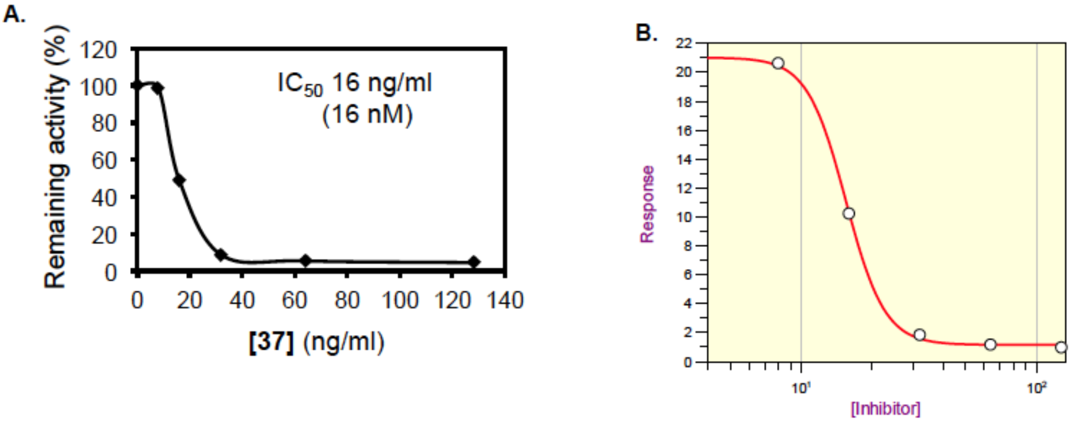

Parameter	Value	Std. Error
Y Range	19.8801	0.3463
IC 50	15.4966	0.2084
Slope factor	5.2664	0.7346
Background	1.1476	0.1828

Supplementary Figure 44. Fluorescence assay from *Mtb* mc[2] 6230 membranes for the inhibition of MurX by dihydrosansanmycin analogue **37**. **A**: Raw dose-response curve for inhibition of MurX by **37**; **B**: Log-transformed dose-response curve for inhibition of MurX by **37**. Data presented is the average of two independent experiments.

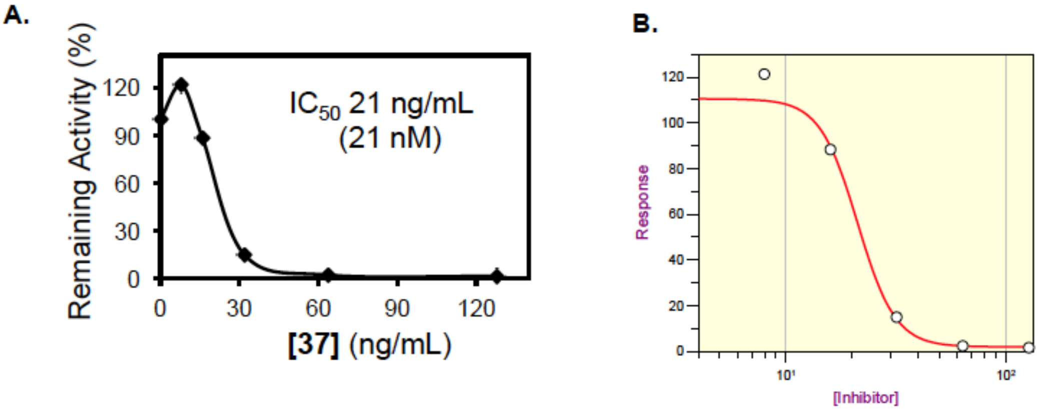

Parameter	Value	Std. Error
Y Range	108.6626	11.7443
IC 50	21.1669	2.9579
Slope factor	5.0584	2.0043
Background	1.8615	8.1196

Supplementary Figure 45. Fluorescence assay from *Mtb* mc[2] 6230 membranes for the inhibition of MurX by dihydrosansanmycin analogue **37** (polyprenylphosphate concentration = 160 µg/mL). **A**: Raw dose-response curve for inhibition of MurX by **37**; **B**: Log-transformed dose-response curve for inhibition of MurX by **37**. Data presented is the average of two independent experiments.

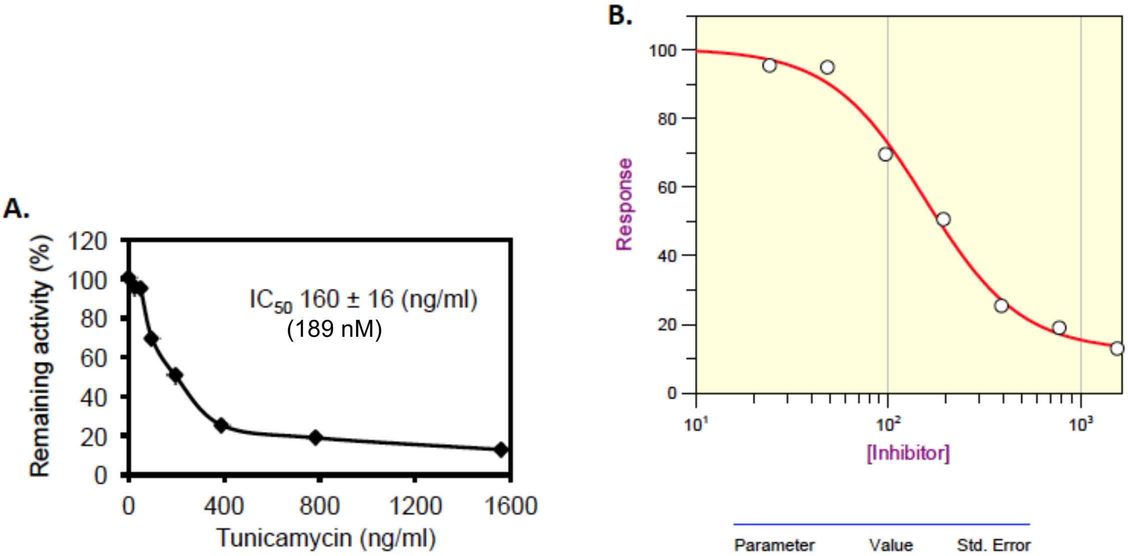

Parameter	Value	Std. Error
Y Range	88.1355	5.4493
IC 50	156.6416	16.0159
Slope factor	1.7489	0.2786
Background	12.2185	3.7857

Supplementary Figure 46. Fluorescence assay from *Mtb* mc^2 6230 membranes for the inhibition of MurX by tunicamycin (positive control). **A**: Raw dose-response curve for inhibition of MurX by tunicamycin; **B**: Log-transformed dose-response curve for inhibition of MurX by tunicamycin. Data presented is the average of two independent experiments.

NMR Spectra

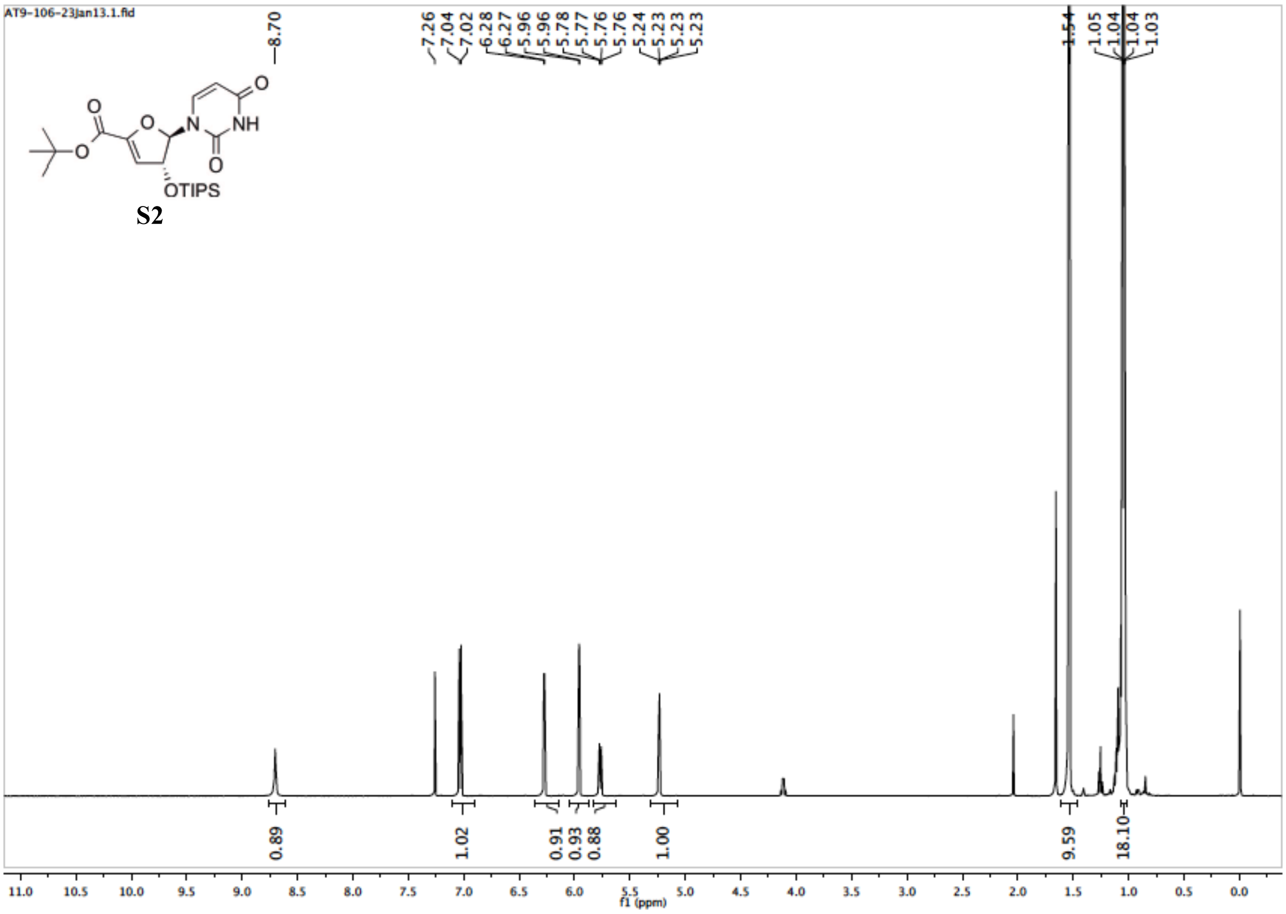

Supplementary Figure 47. ^{1}H NMR spectrum of compound **S2**.

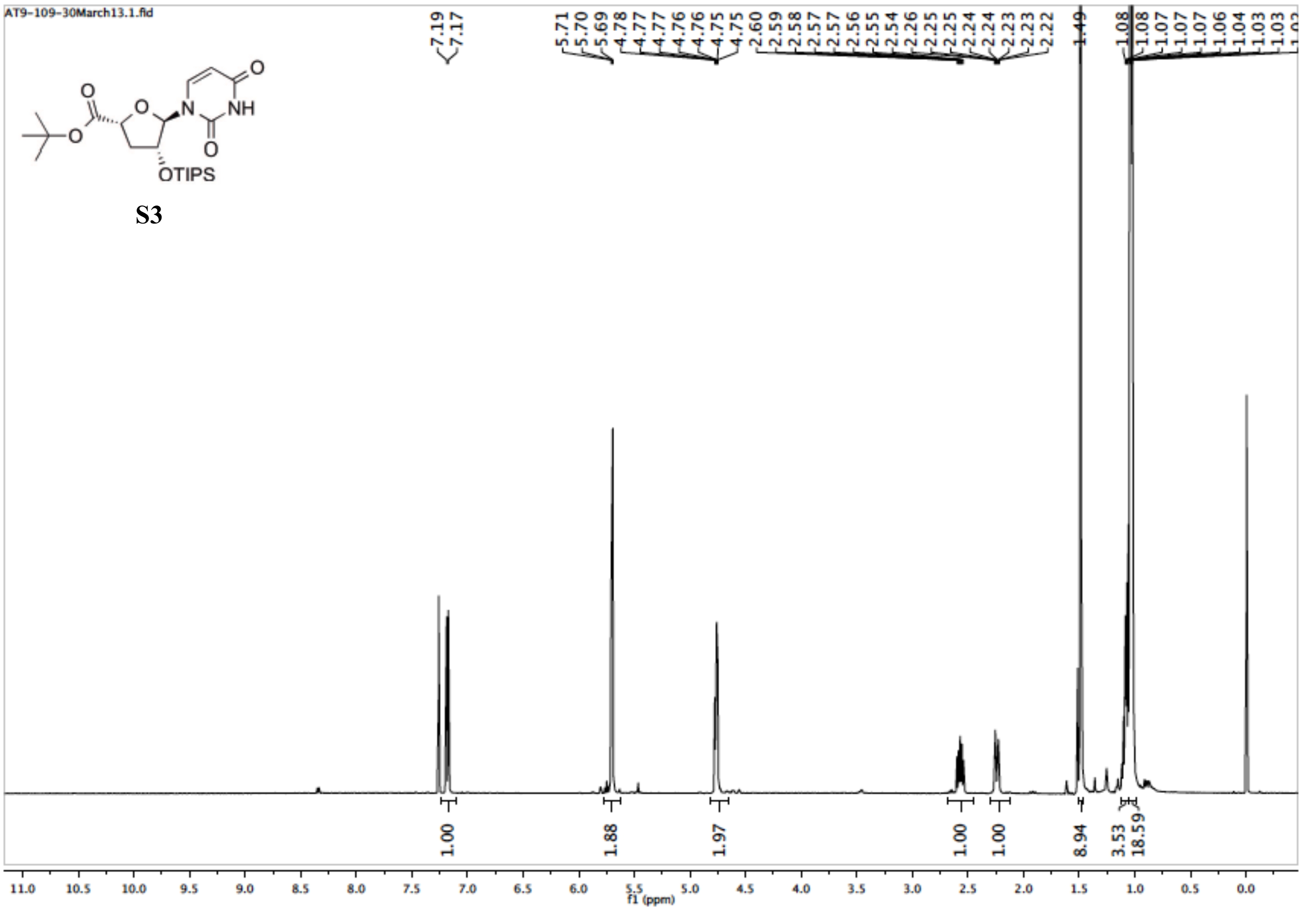

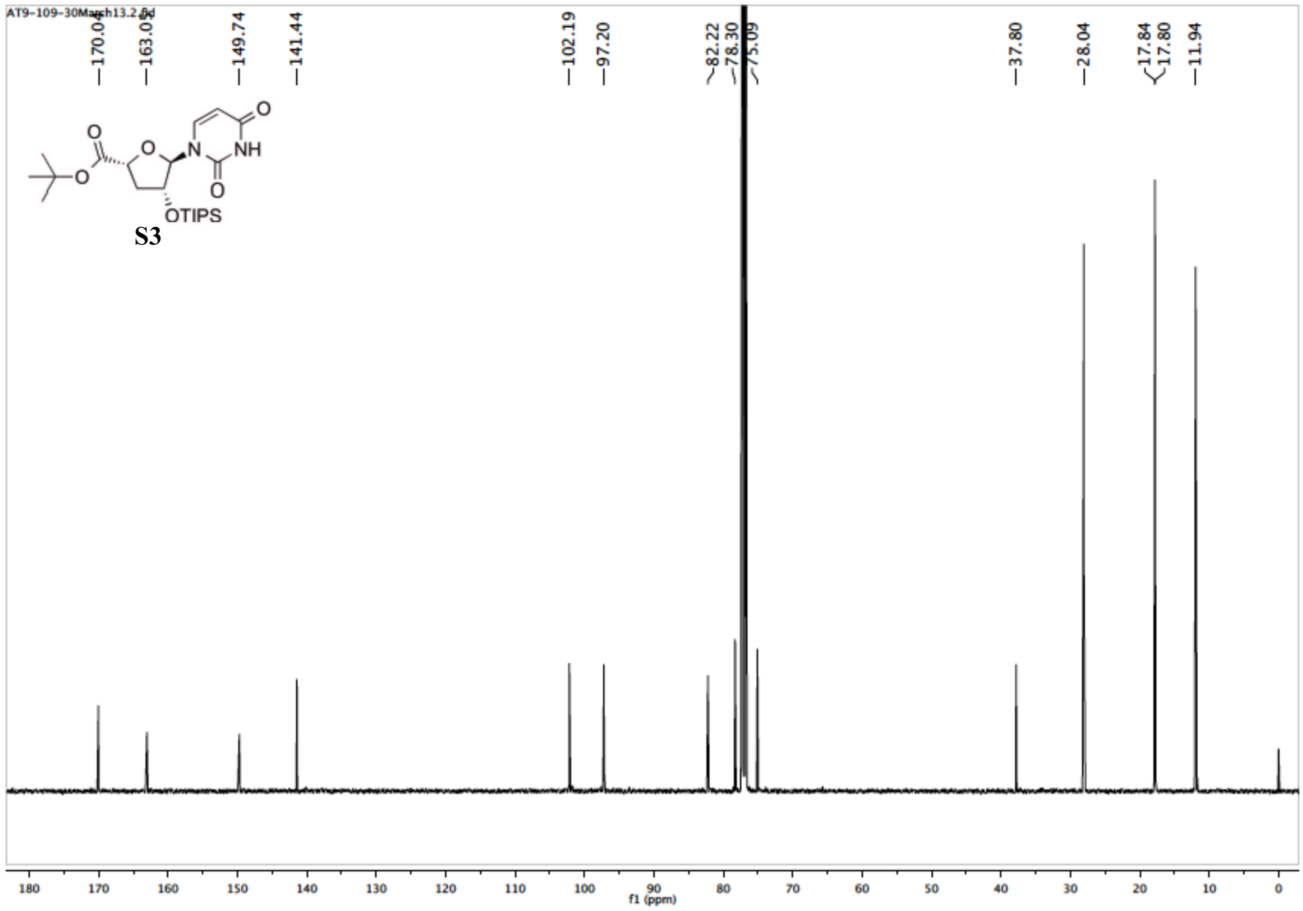

Supplementary Figure 48. ^{1}H (top) and $^{13}C\{^{1}H\}$ (bottom) NMR spectra of compound **S3**.

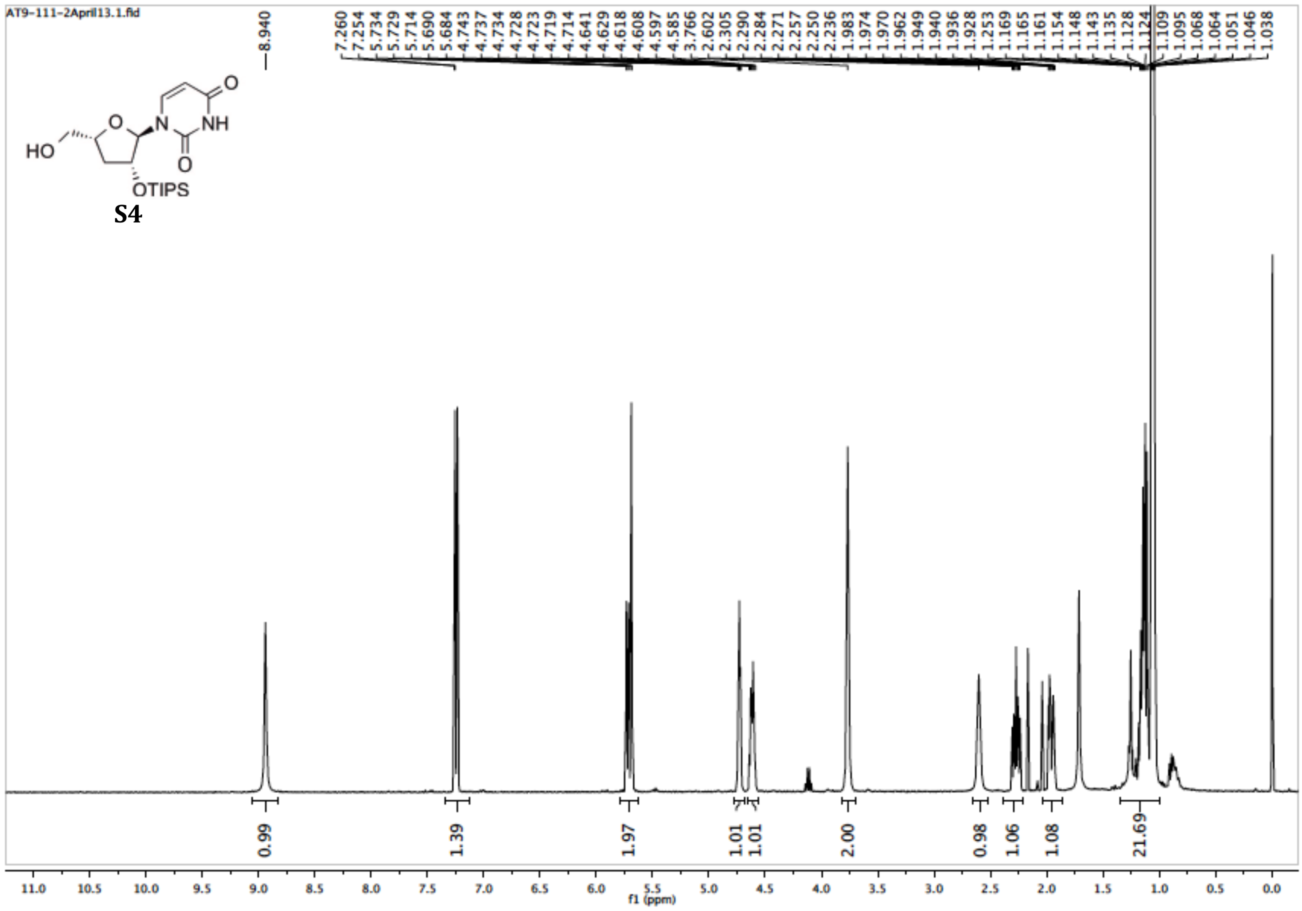

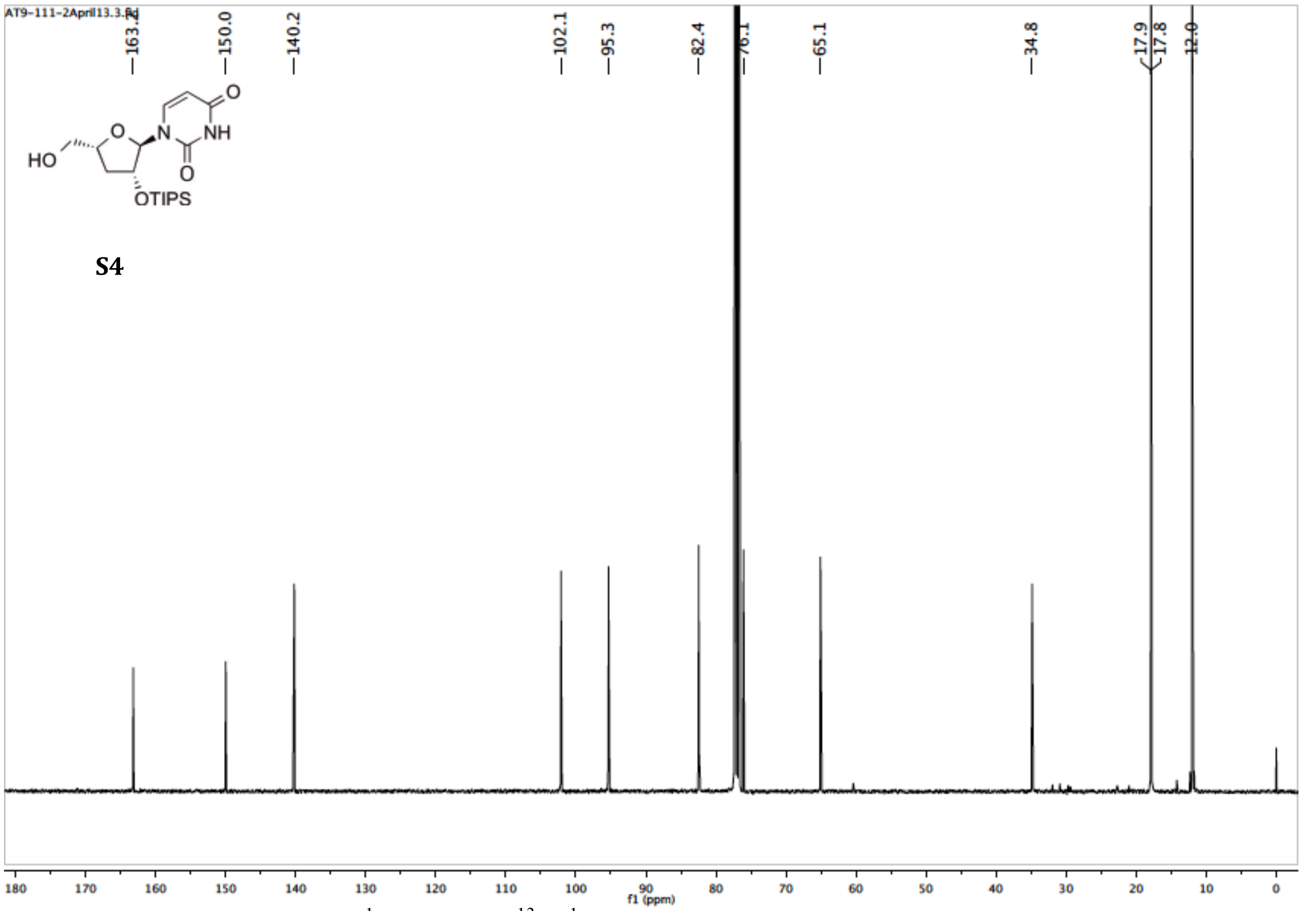

Supplementary Figure 49. ^{1}H (top) and $^{13}C\{^{1}H\}$ (bottom) NMR spectra of compound **S4**.

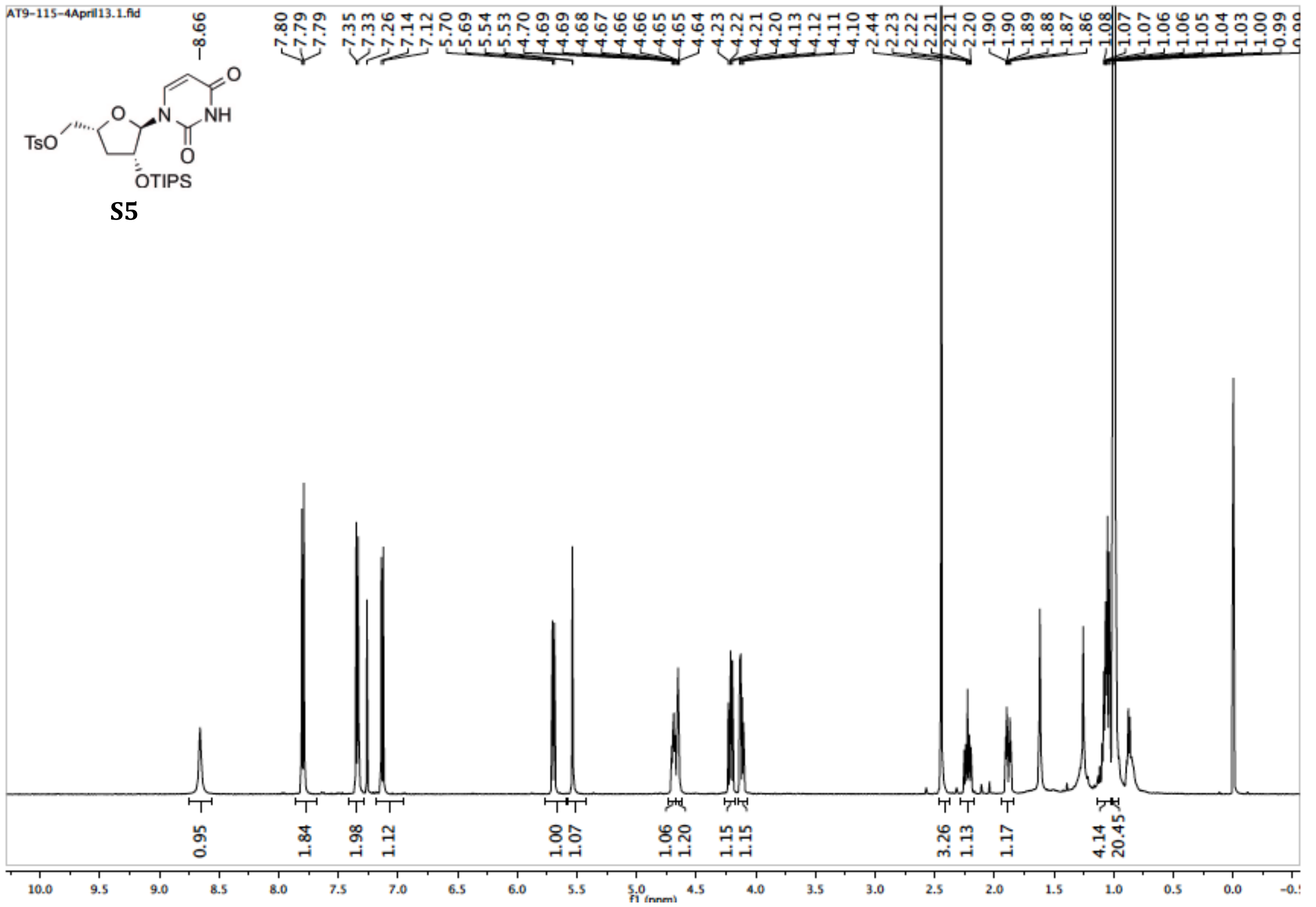

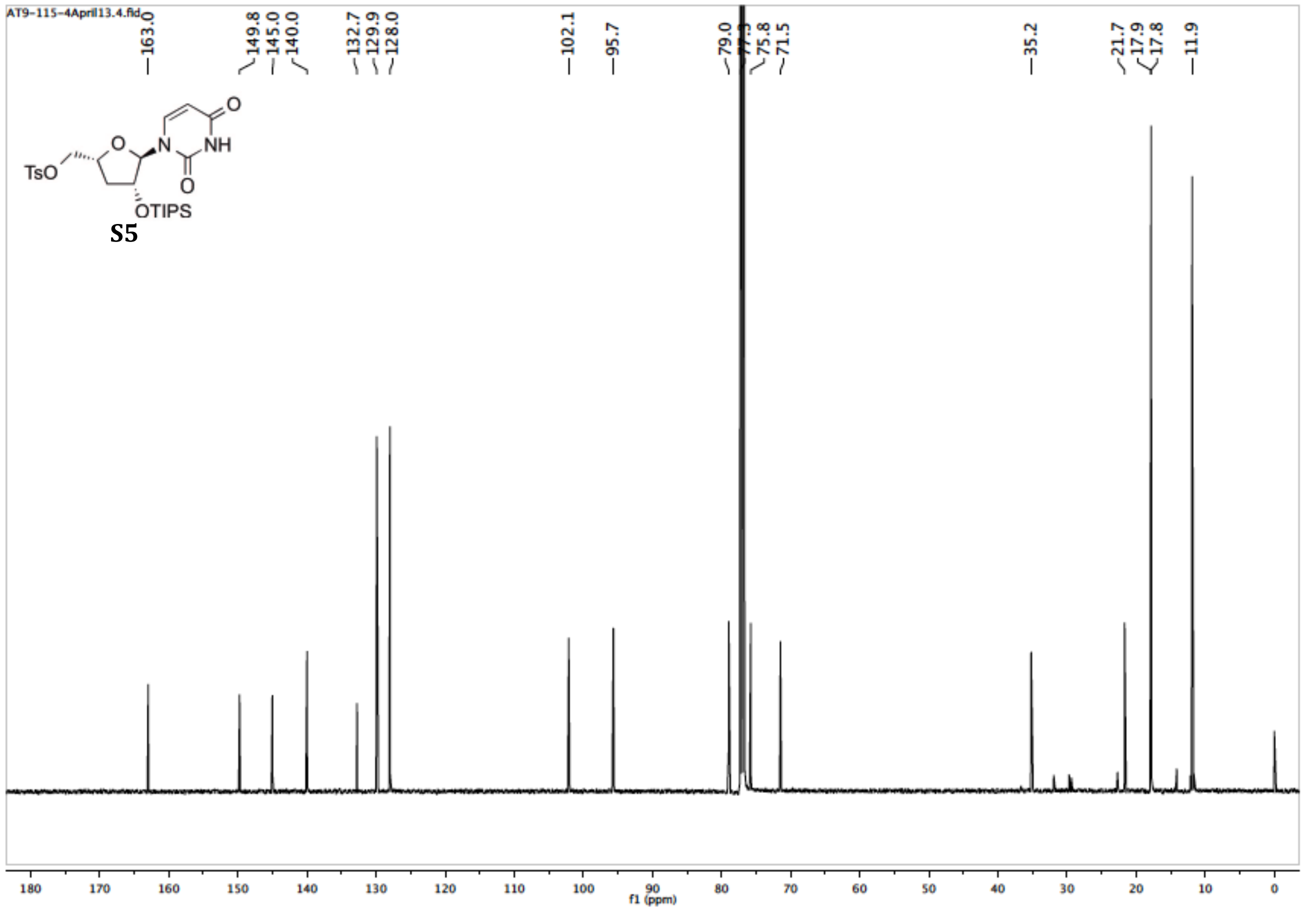

Supplementary Figure 50. ^{1}H (top) and $^{13}C\{^{1}H\}$ (bottom) NMR spectra of compound **S5**.

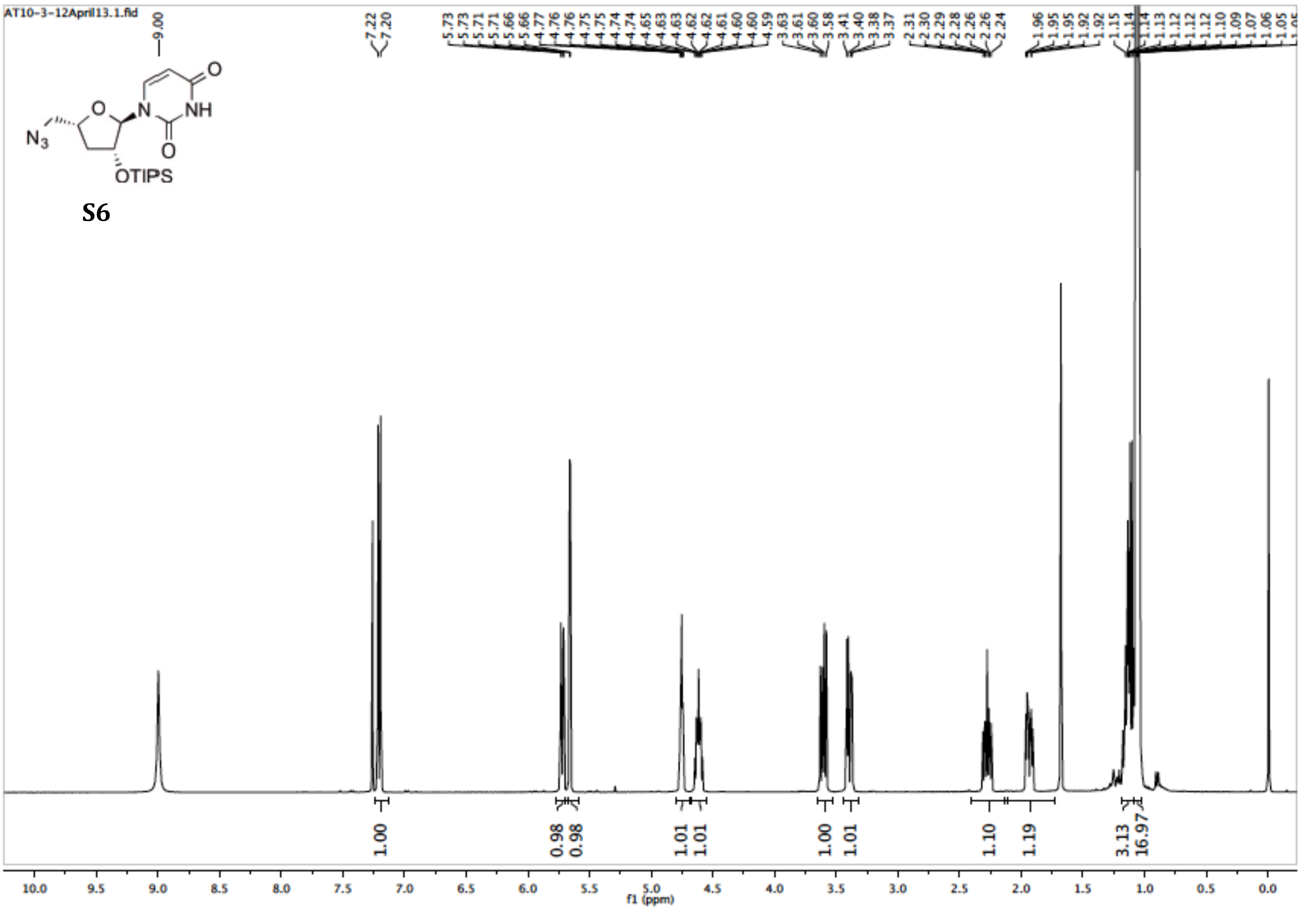

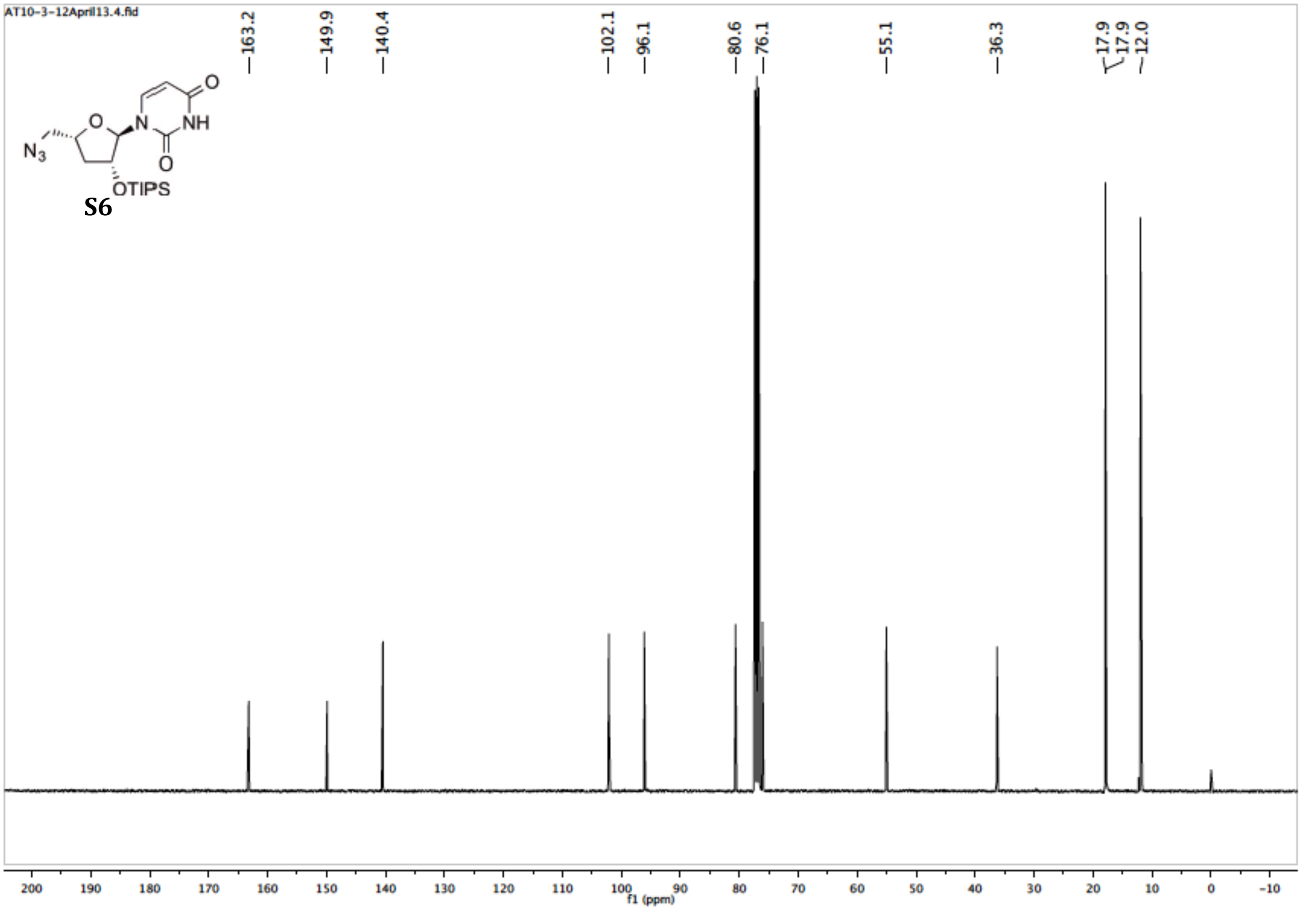

Supplementary Figure 51. ^{1}H (top) and $^{13}C\{^{1}H\}$ (bottom) NMR spectra of compound **S6**.

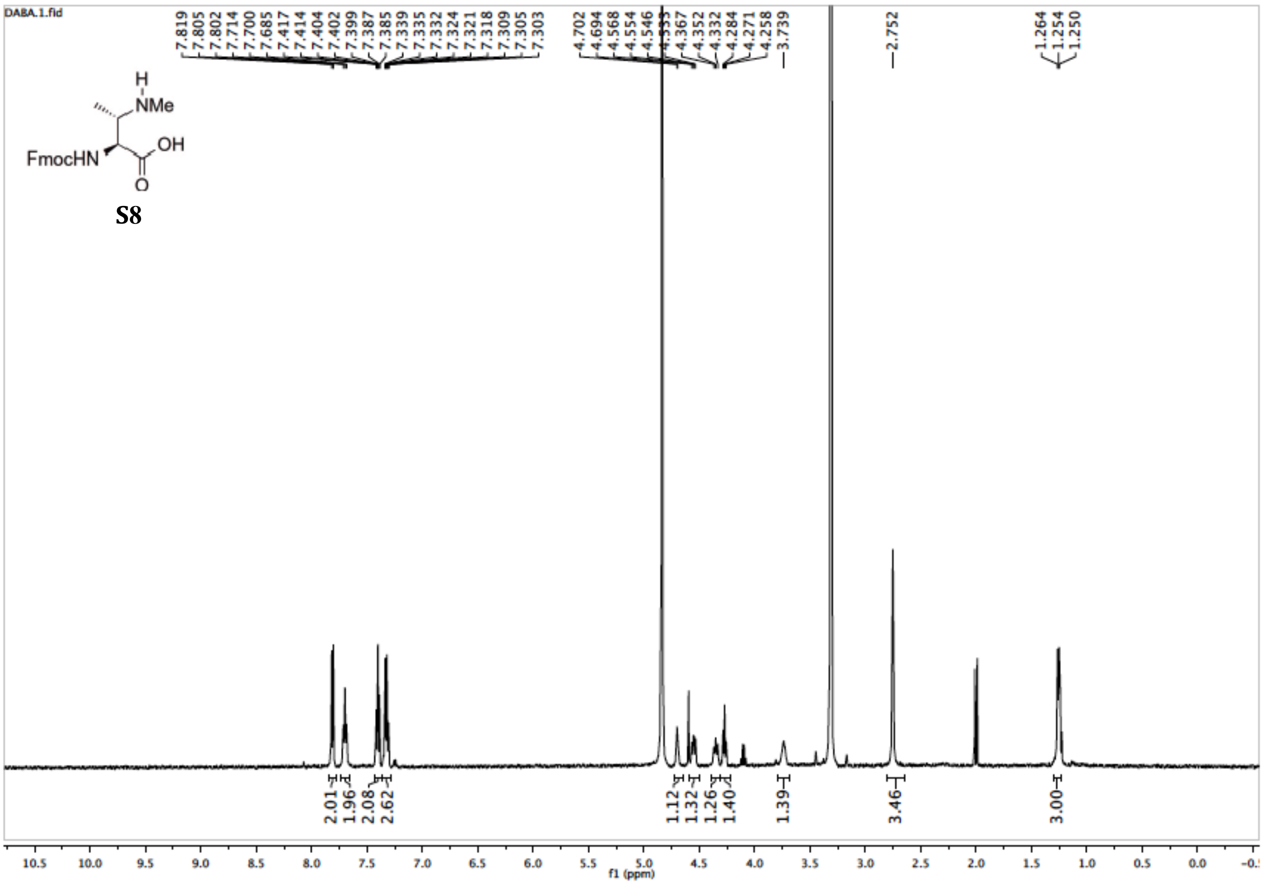

Supplementary Figure 52. ^{1}H NMR spectrum of compound **S8**.

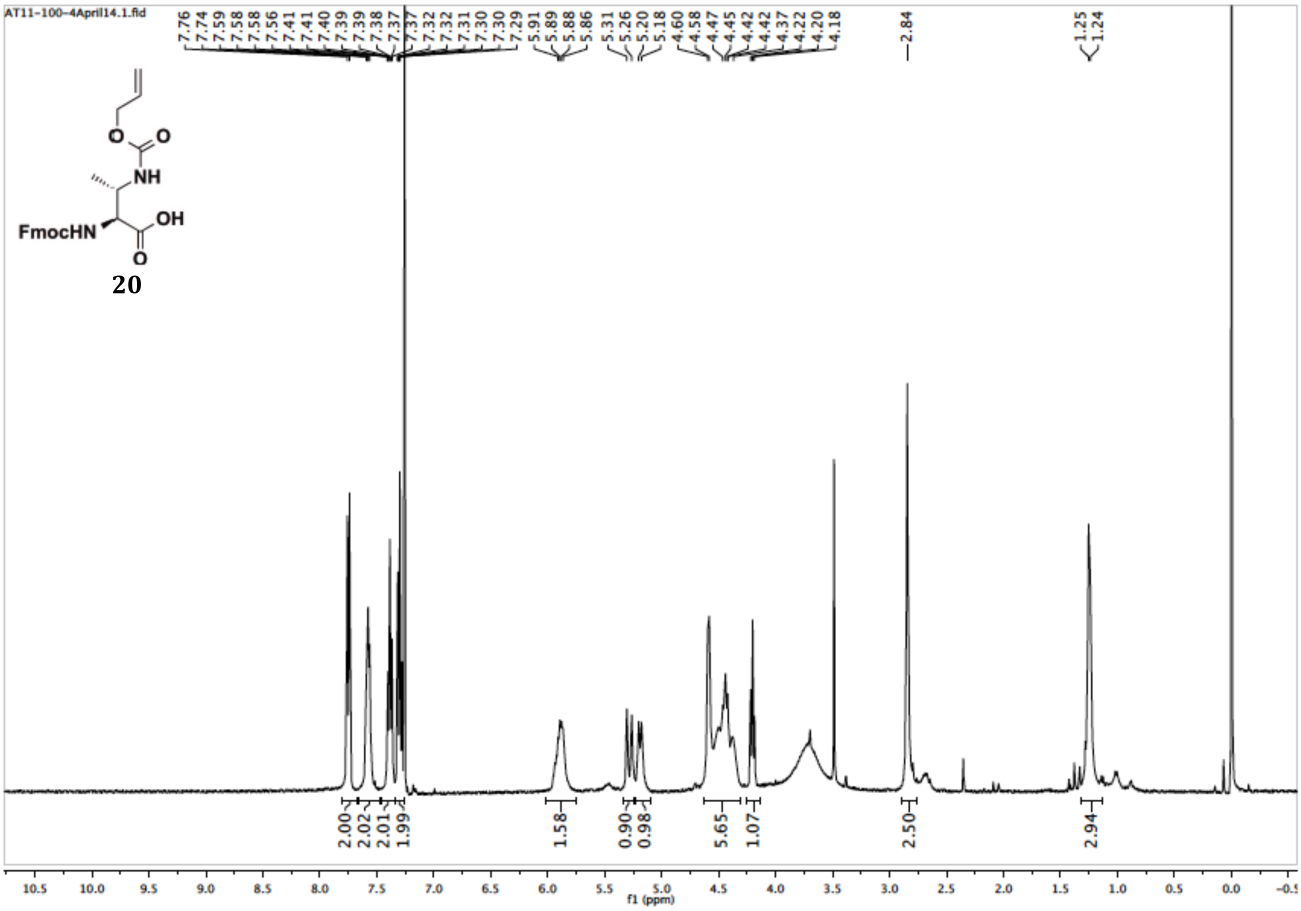

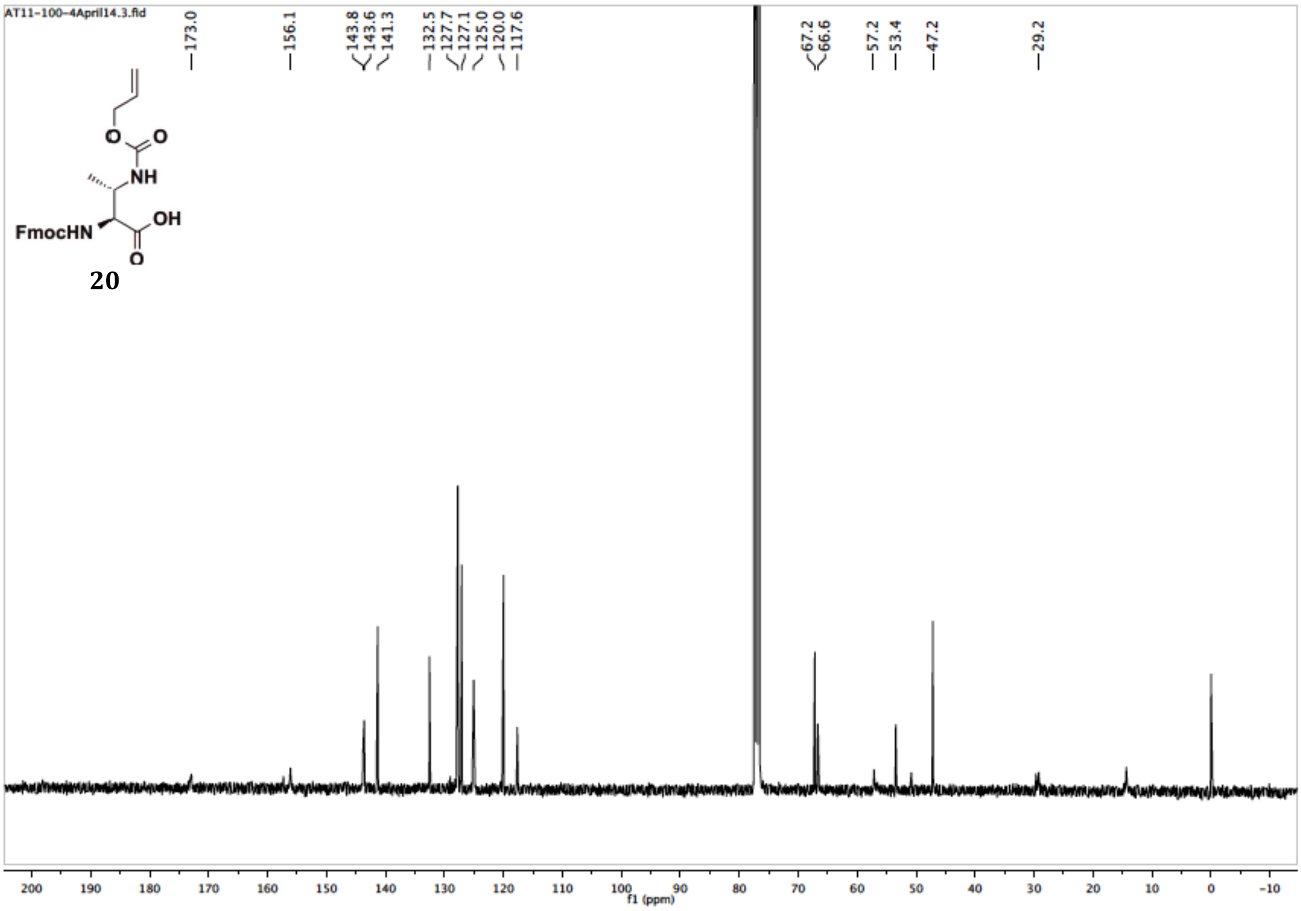

Supplementary Figure 53. ^{1}H (top) and ^{13}C{^{1}H} (bottom) NMR spectra of compound **20**.

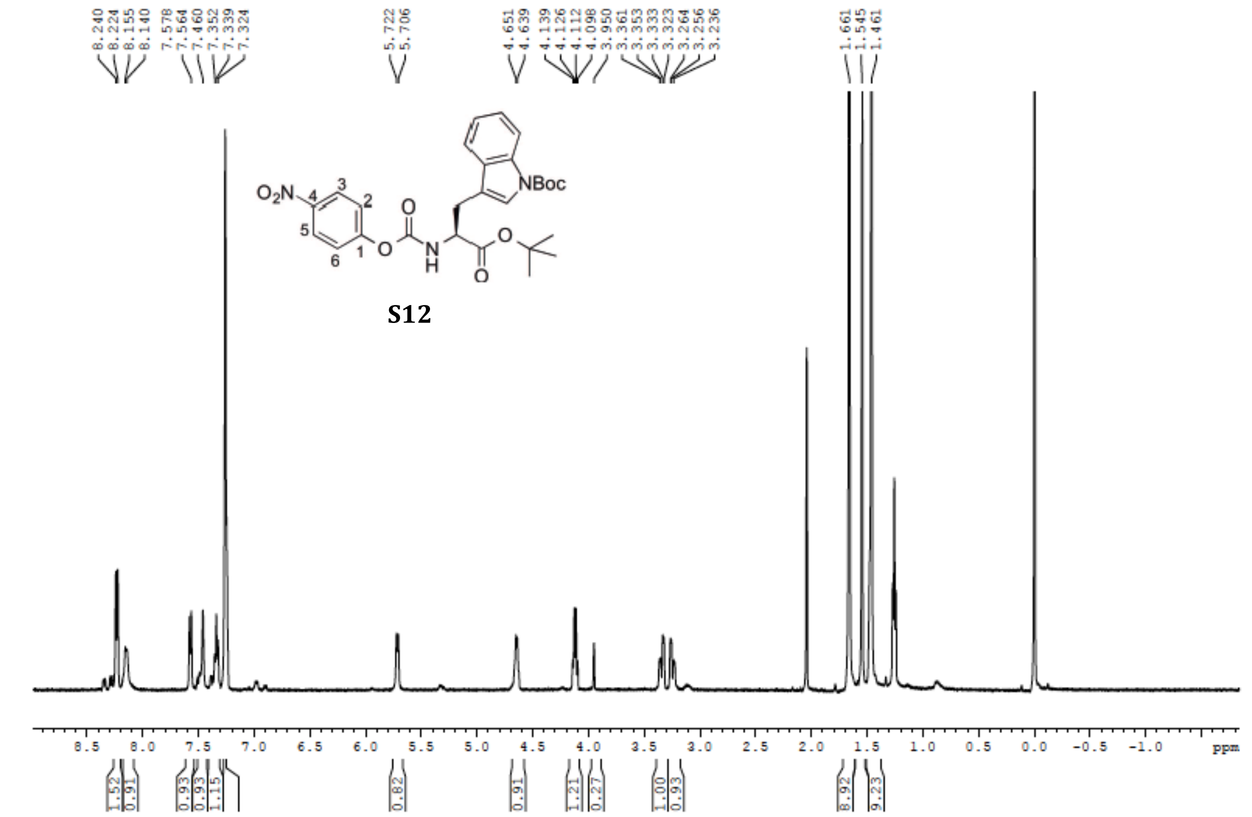

Supplementary Figure 54. ^{1}H NMR spectrum of compound **S12**.

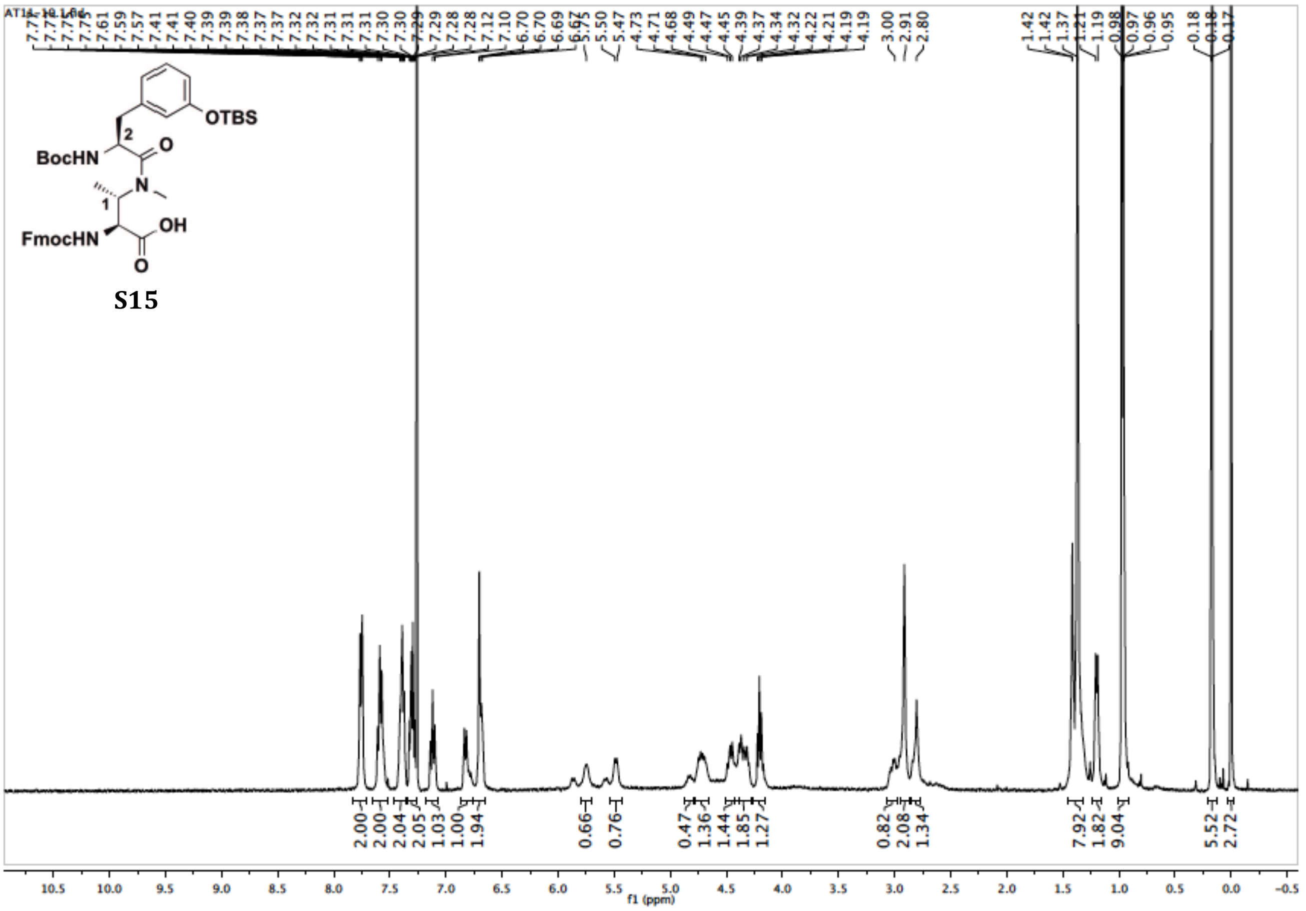

Supplementary Figure 55. ^{1}H NMR spectrum of compound **S15**.

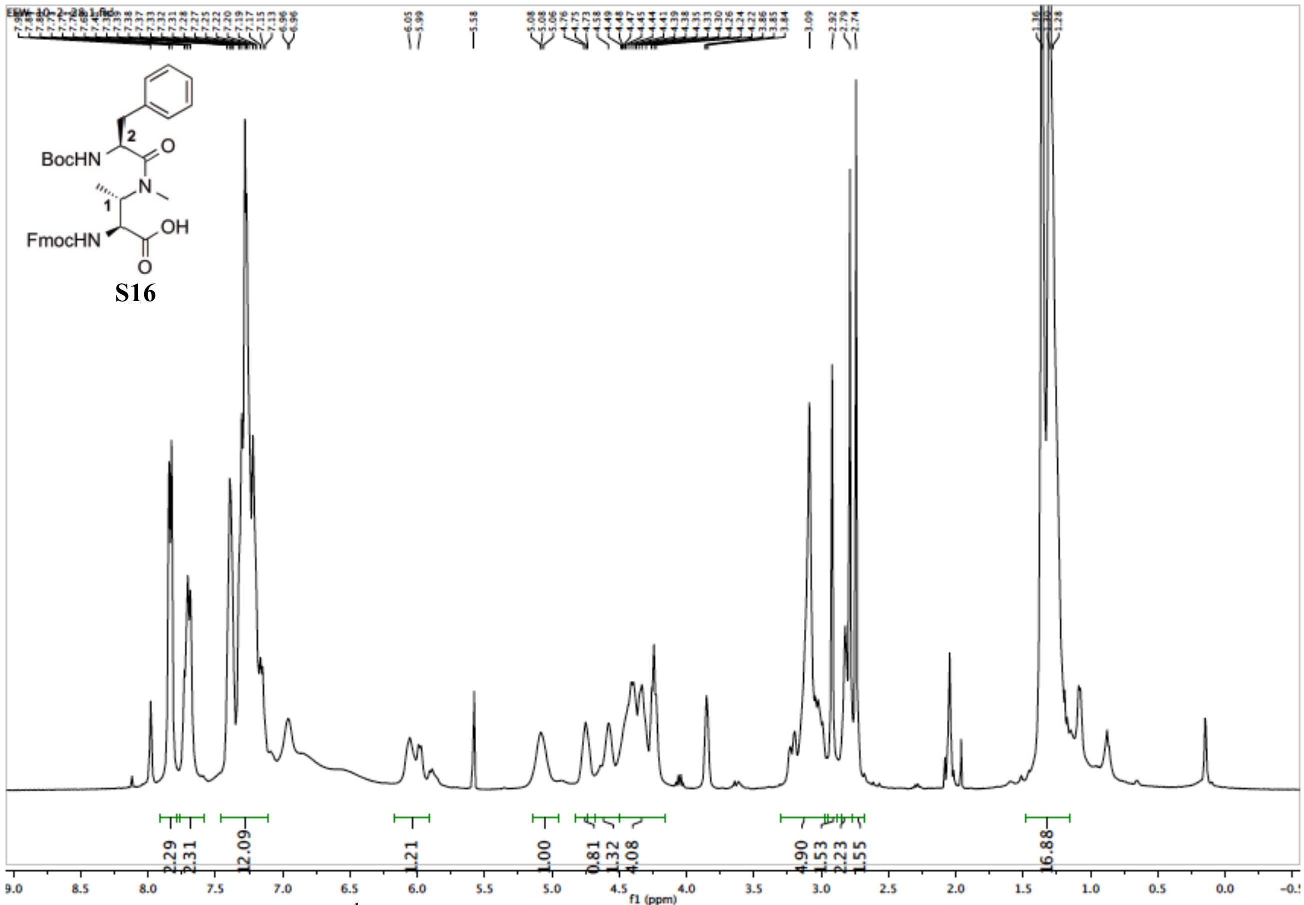

Supplementary Figure 56. ^{1}H NMR spectrum of compound **S16**.

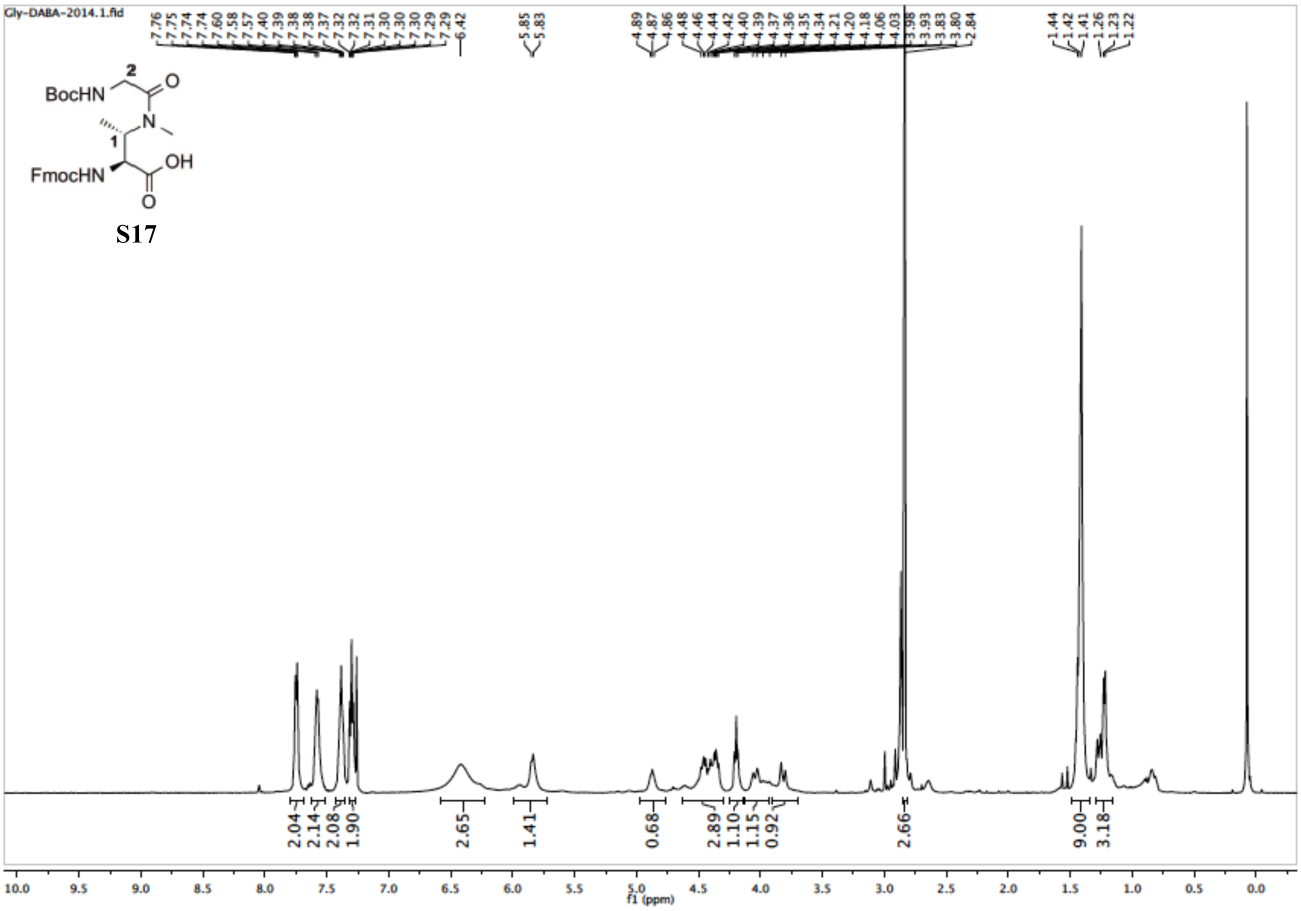

Supplementary Figure 57. ^{1}H NMR spectrum of compound **S17**.

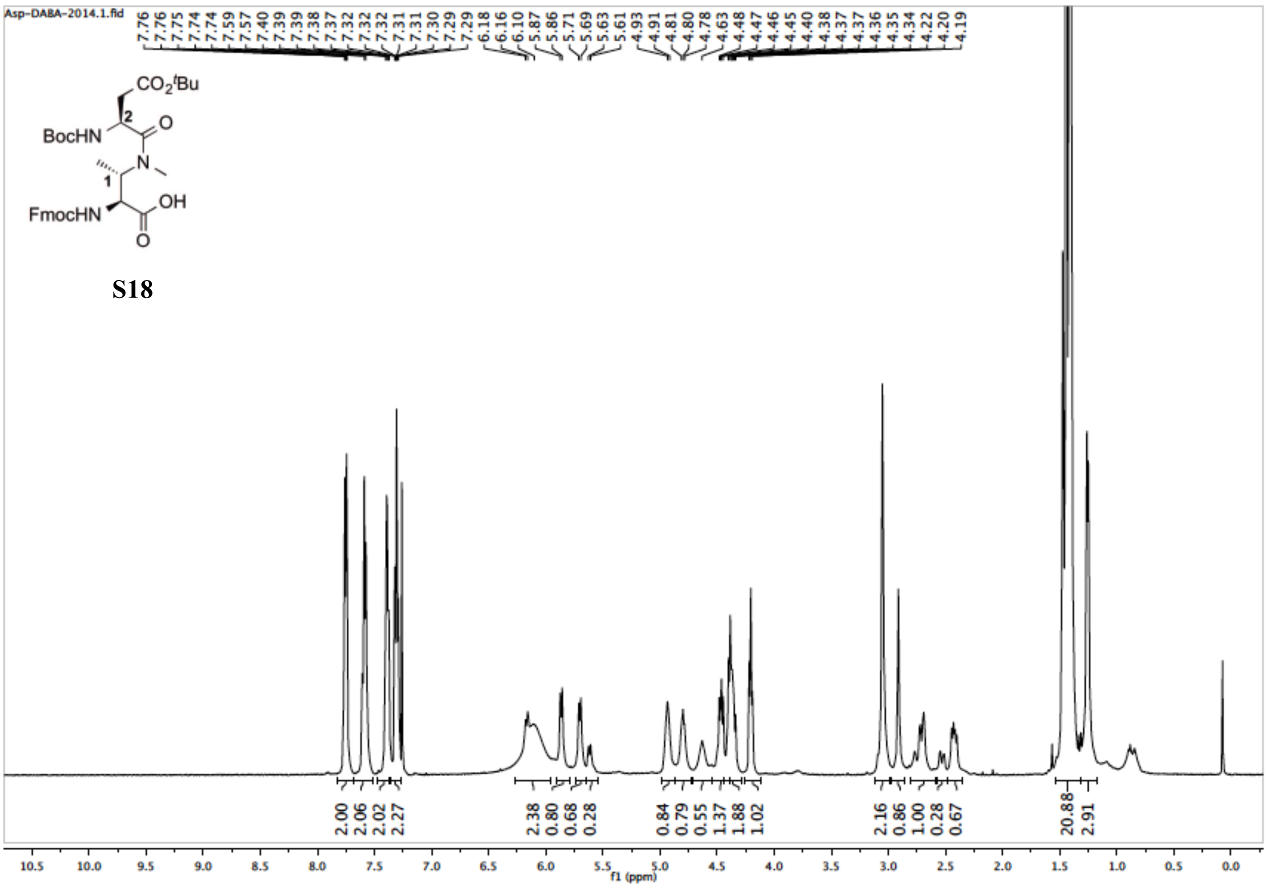

Supplementary Figure 58. ^{1}H NMR spectrum of compound **S18**.

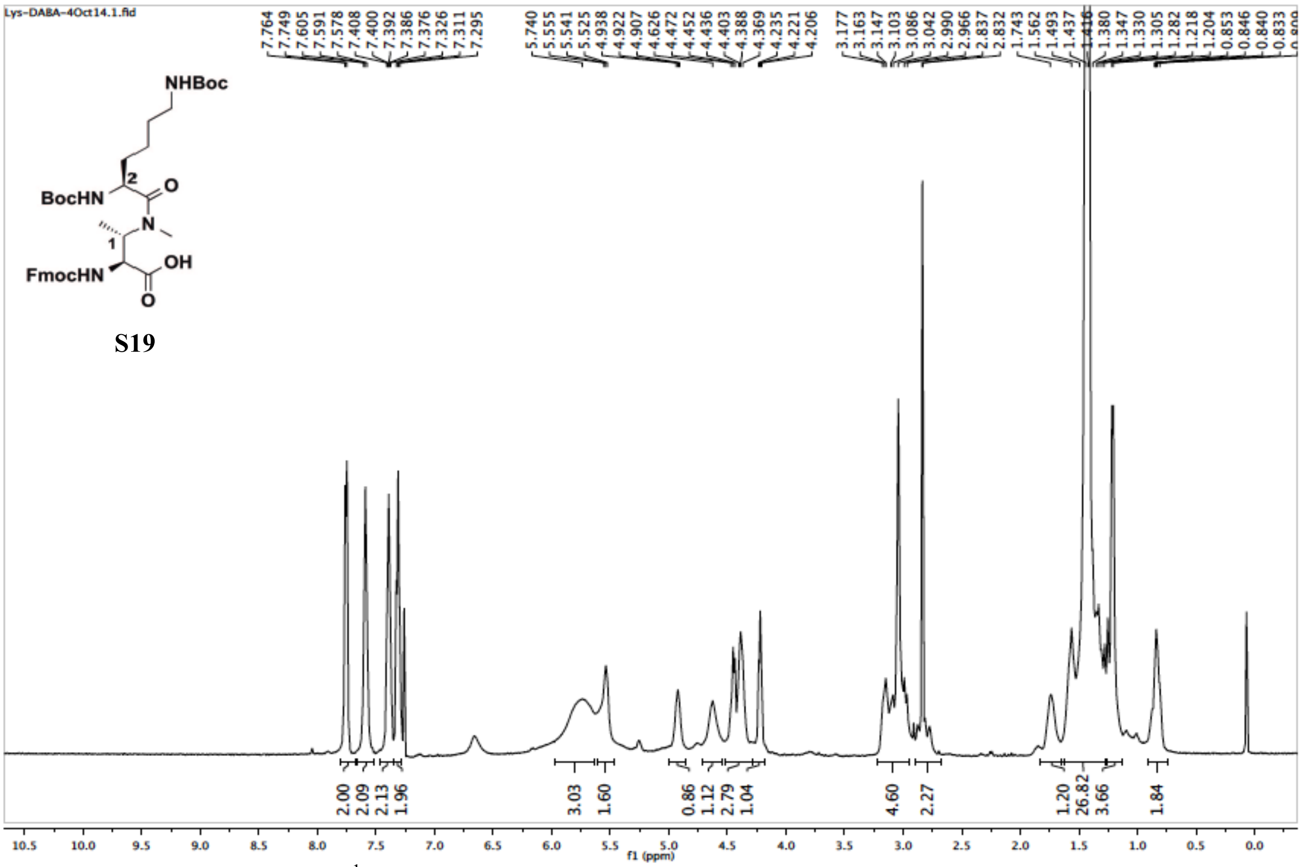

Supplementary Figure 59. ^{1}H NMR spectrum of compound **S19**.

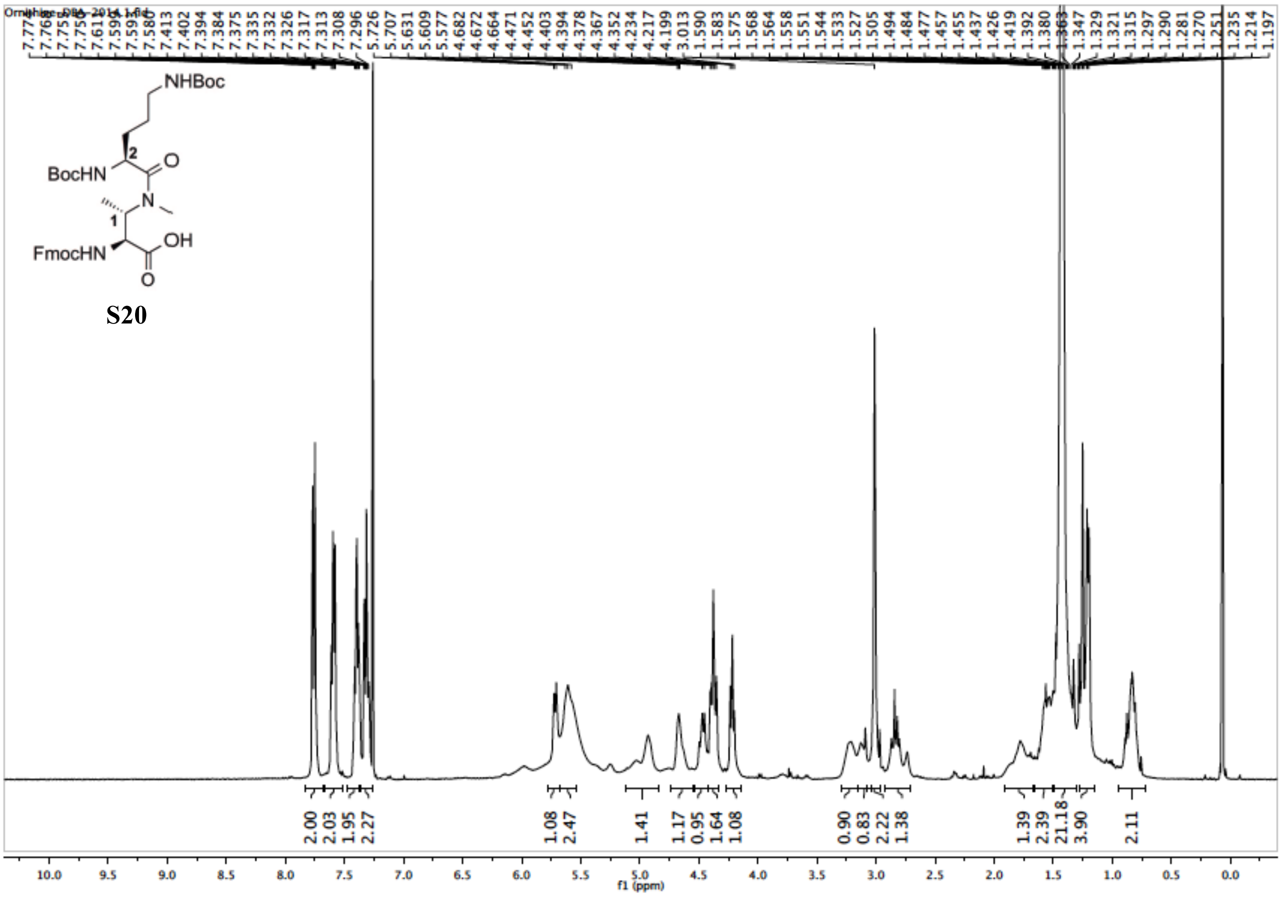

Supplementary Figure 60. ^{1}H NMR spectrum of compound **S20**.

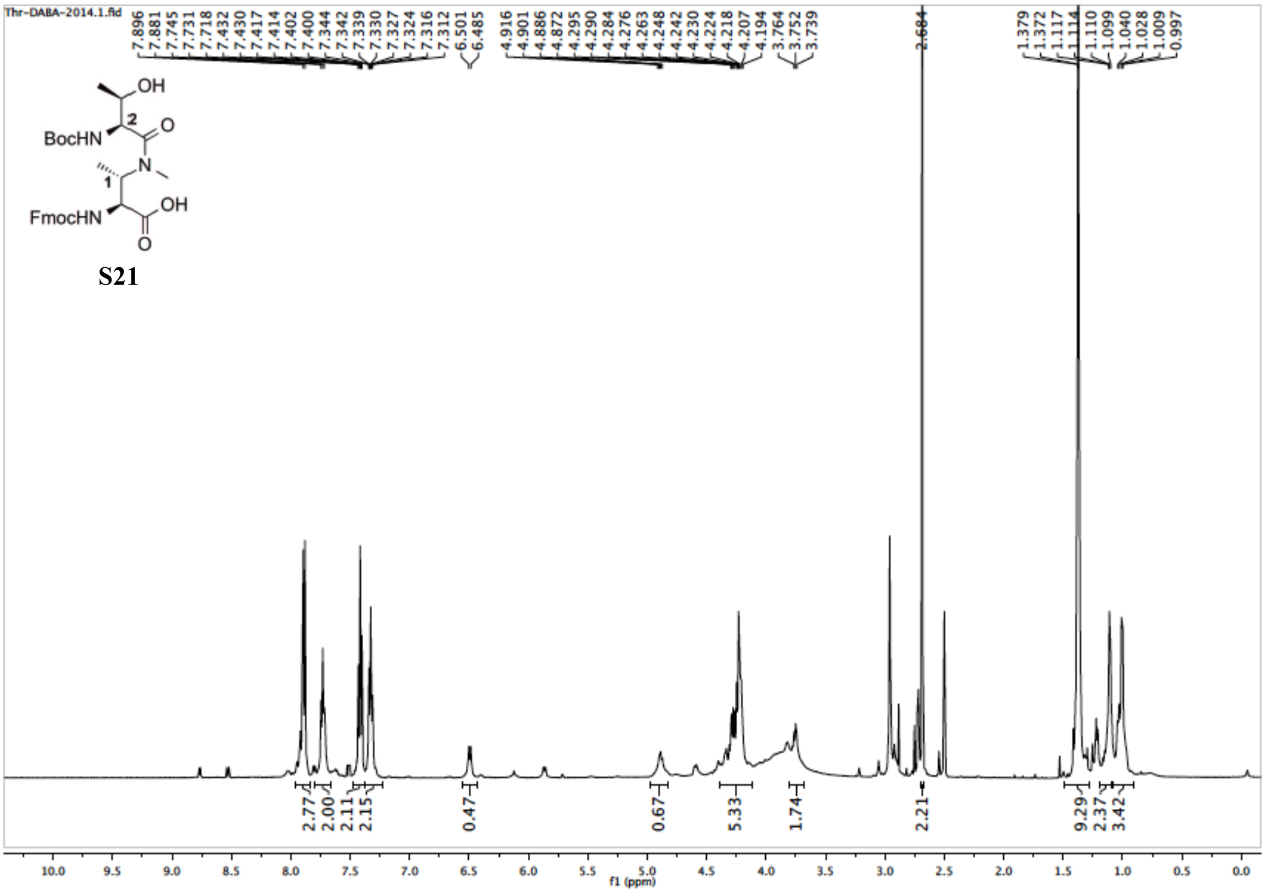

Supplementary Figure 61. ^{1}H NMR spectrum of compound **S21**.

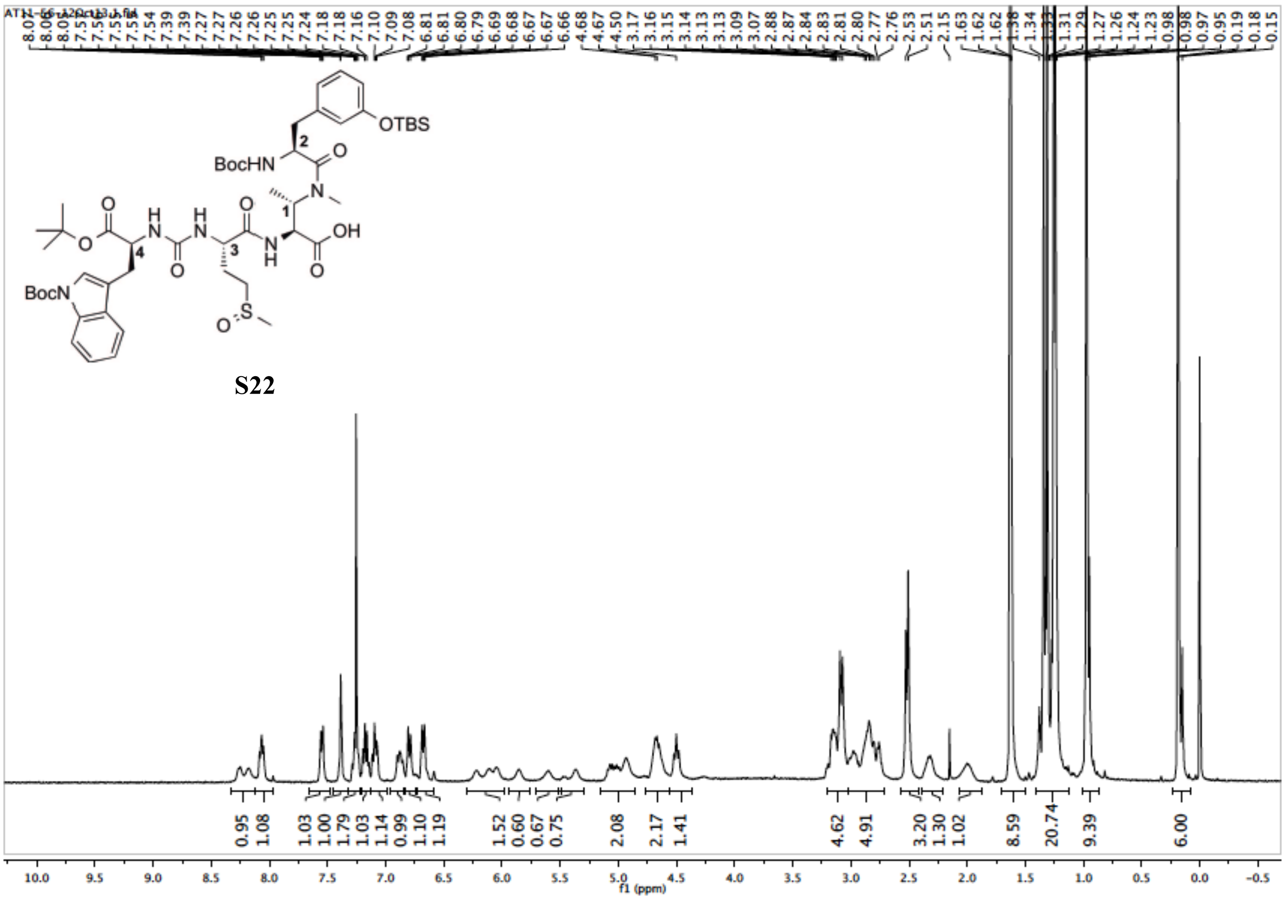

Supplementary Figure 62. ^{1}H NMR spectrum of compound **S22**.

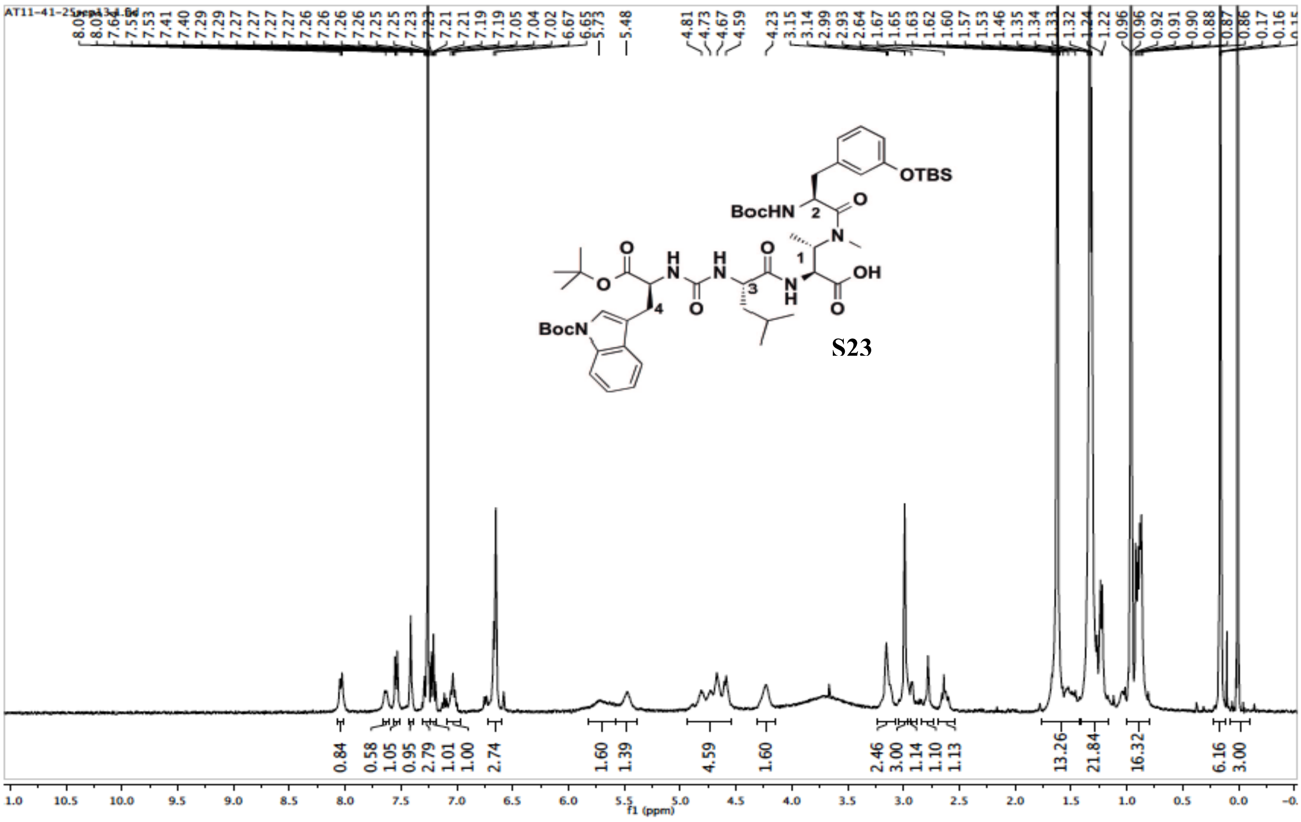

Supplementary Figure 63. ^{1}H NMR spectrum of compound **S23**.

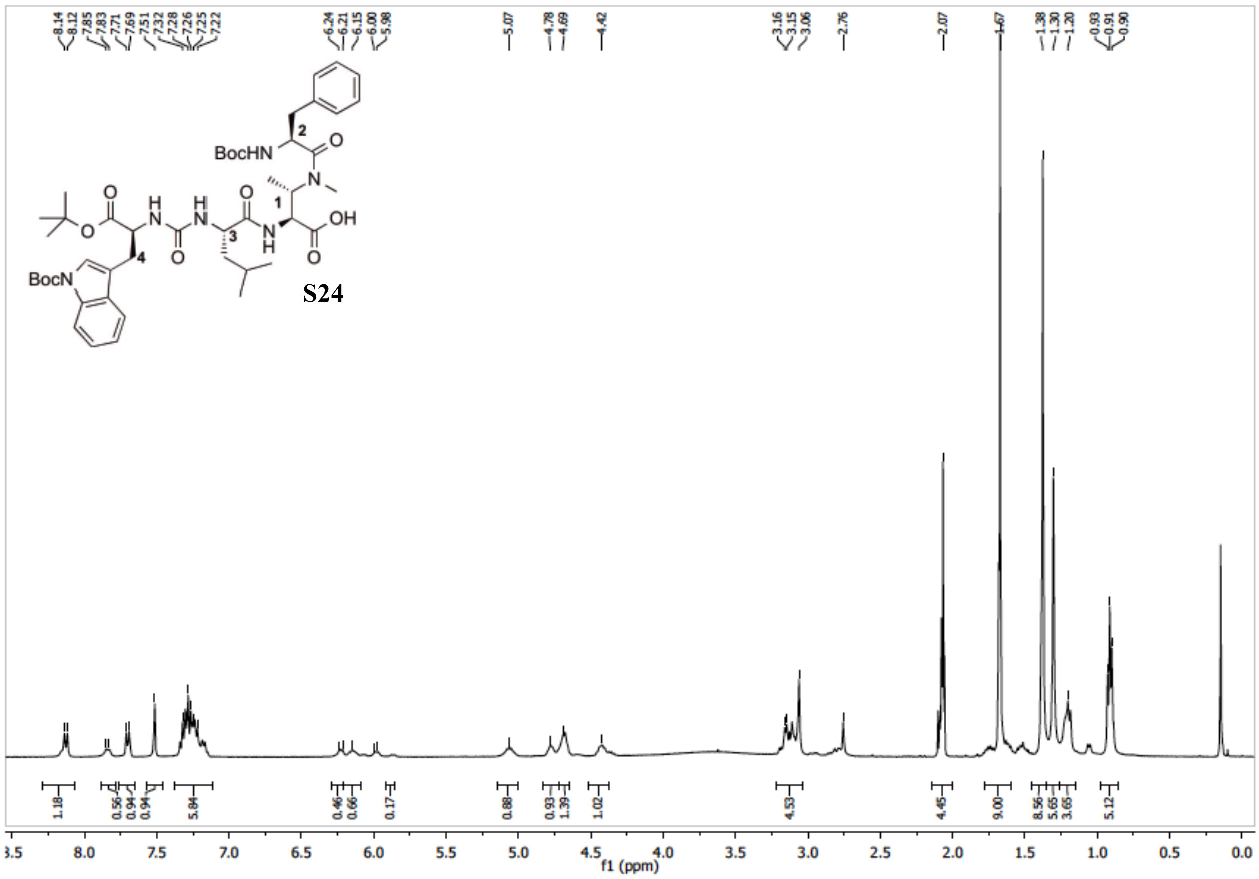

Supplementary Figure 64. ^{1}H NMR spectrum of compound **S24**.

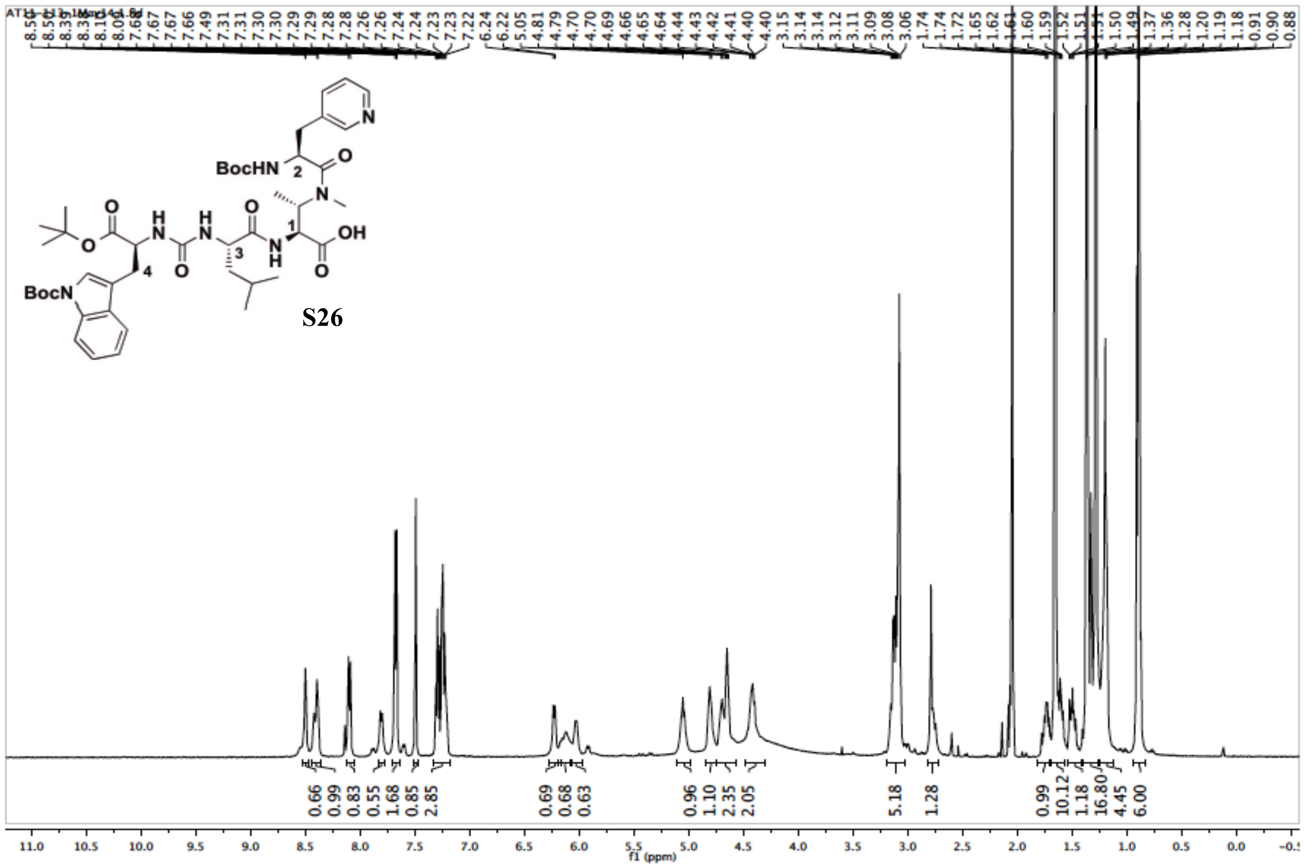

Supplementary Figure 65. ^{1}H NMR spectrum of compound **S26**.

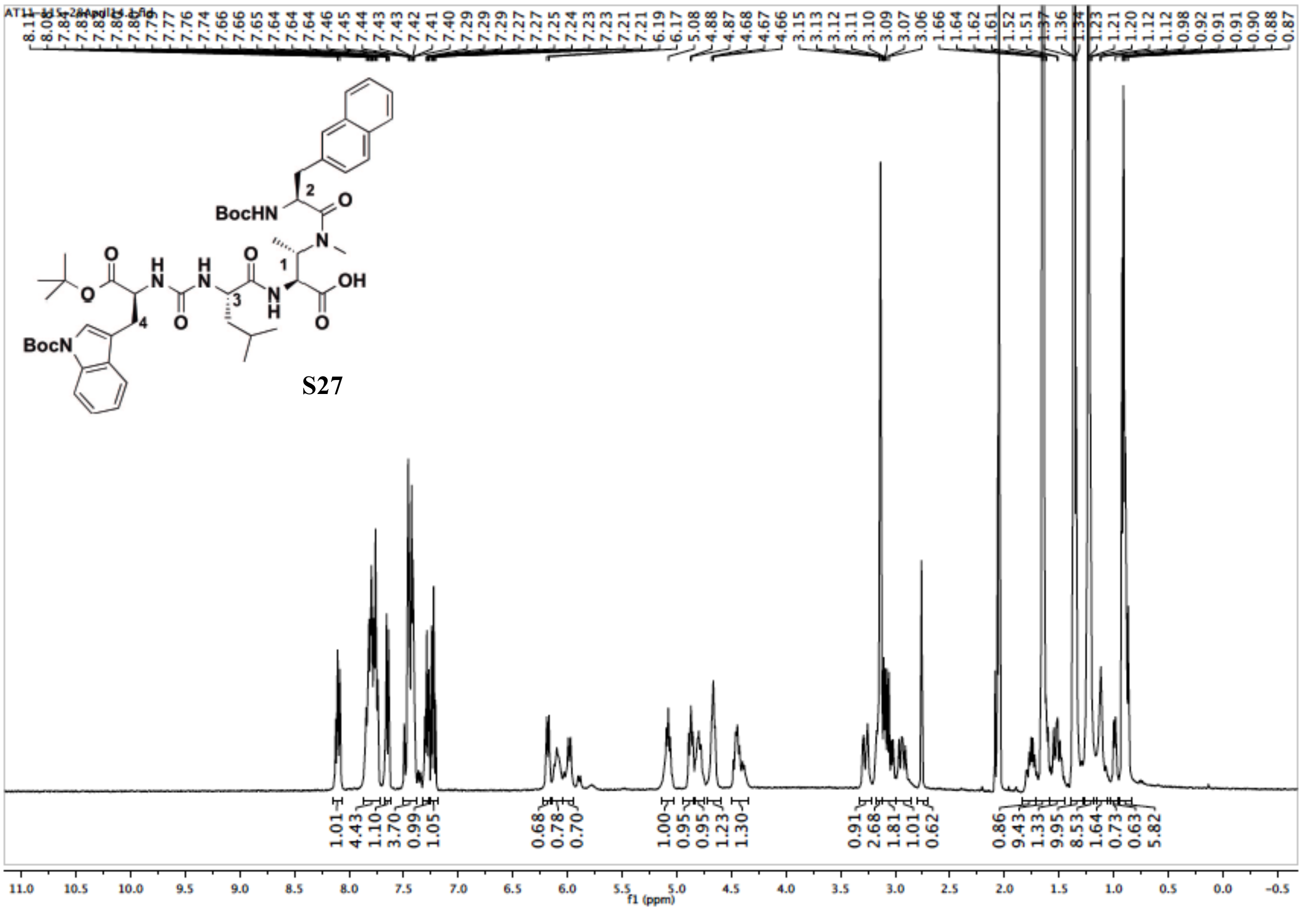

Supplementary Figure 66. ^{1}H NMR spectrum of compound **S27**.

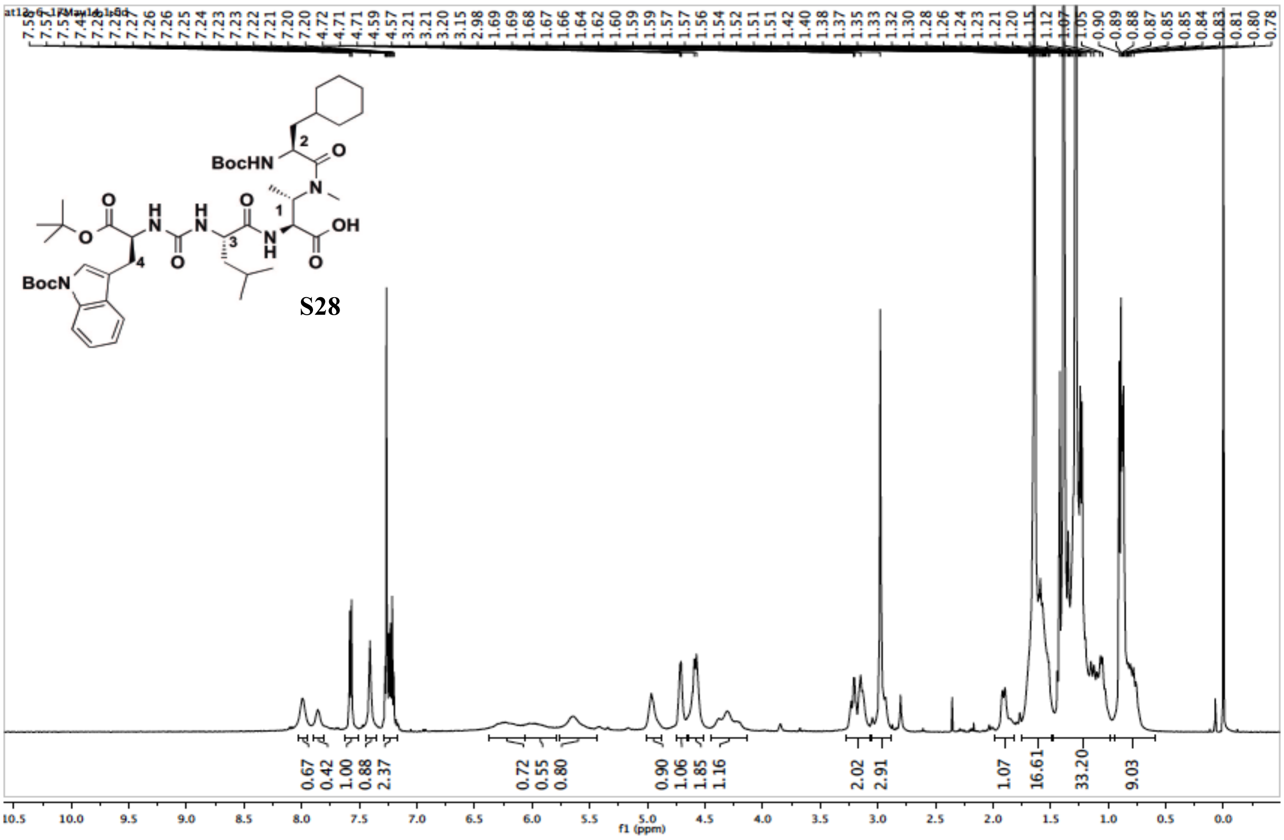

Supplementary Figure 67. ^{1}H NMR spectrum of compound **S28**.

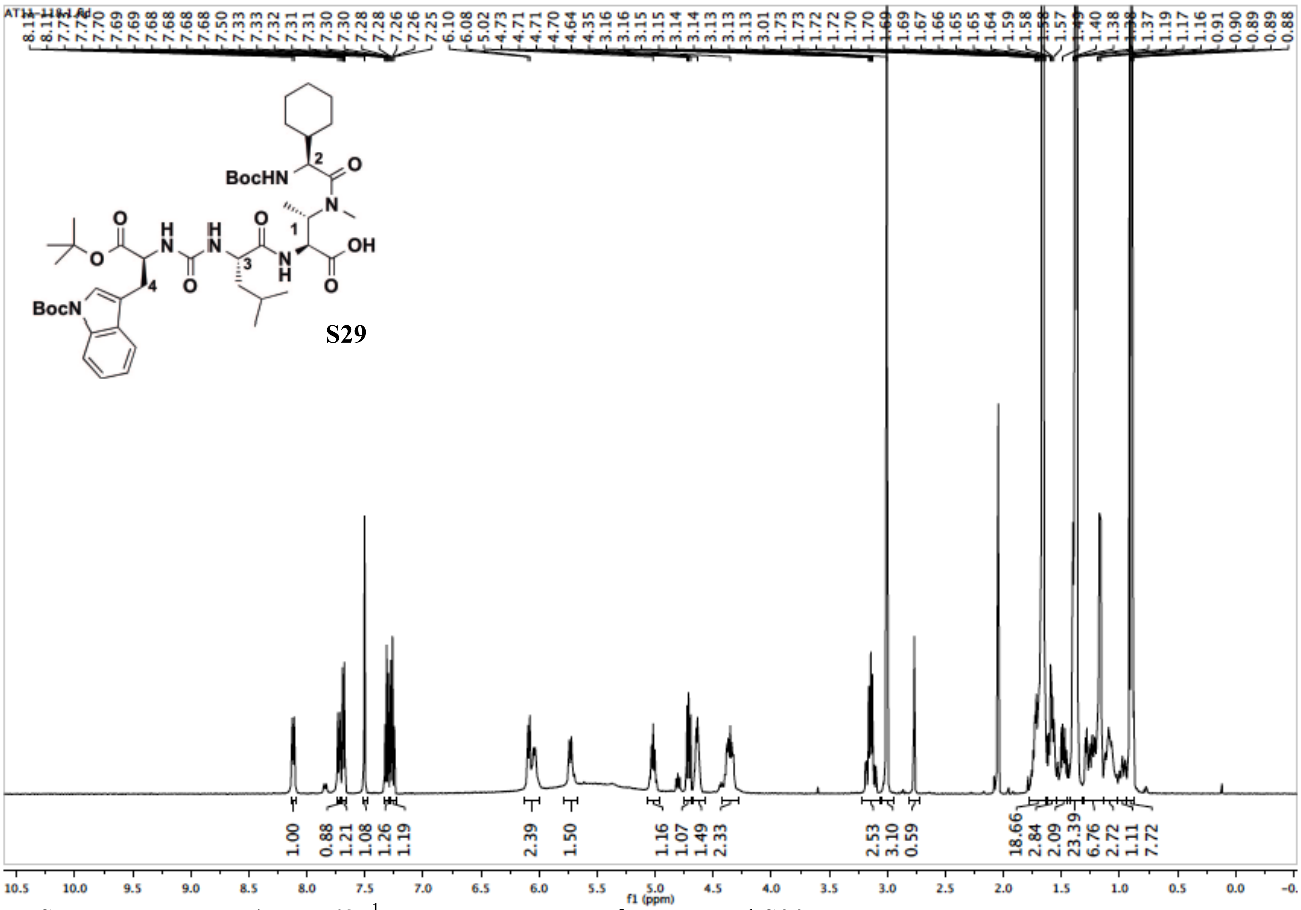

Supplementary Figure 68. ^{1}H NMR spectrum of compound **S29**.

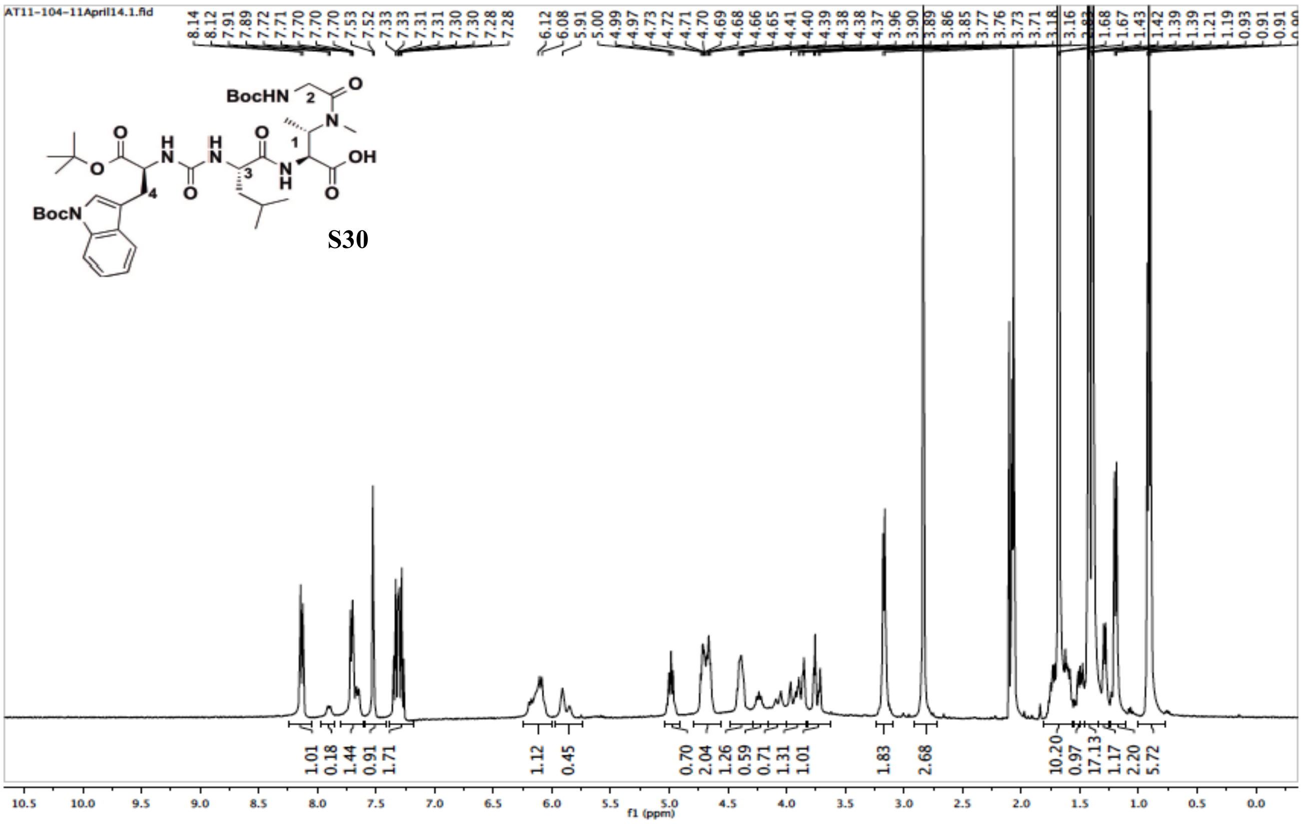

Supplementary Figure 69. ^{1}H NMR spectrum of compound **S30**.

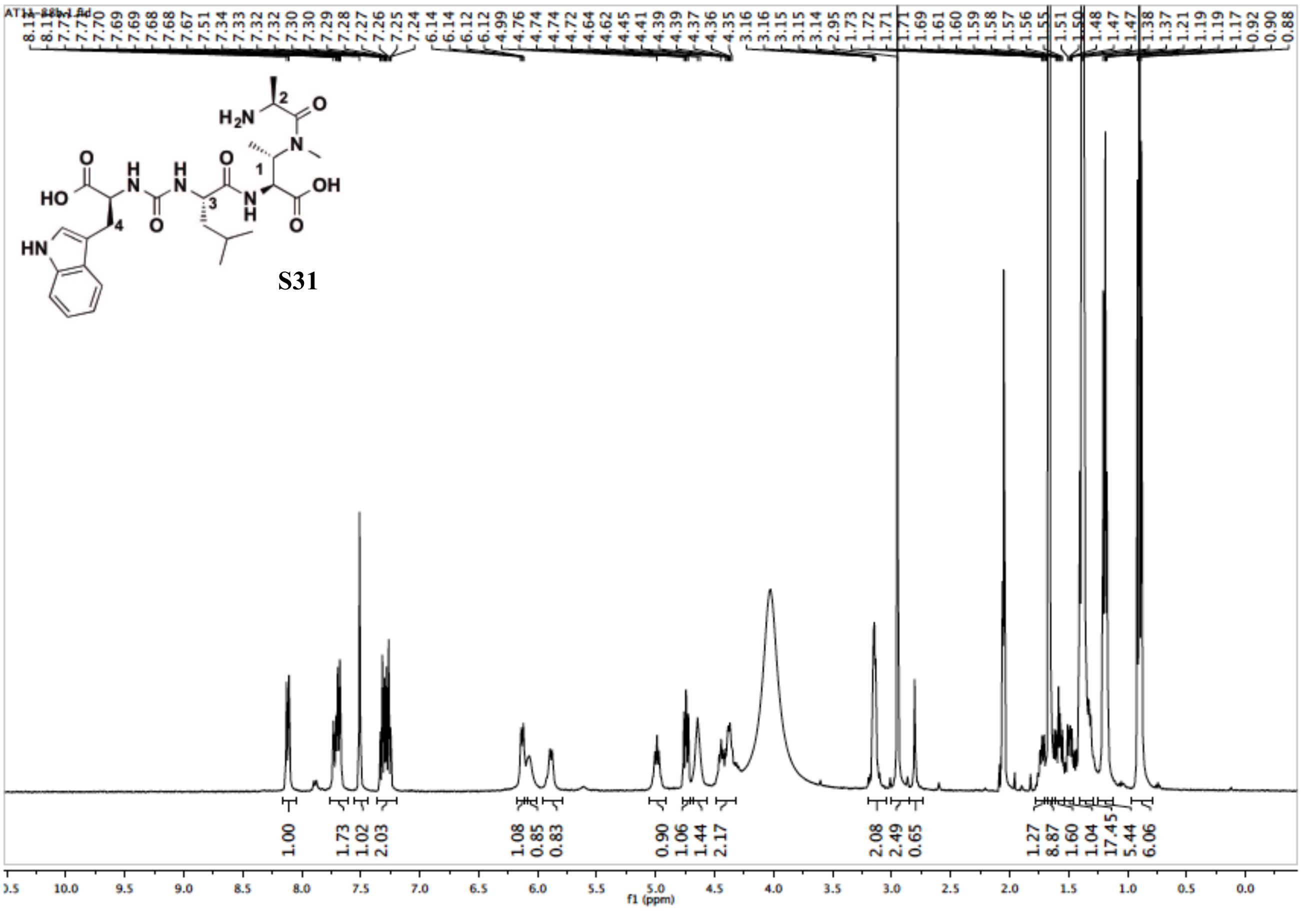

Supplementary Figure 70. ^{1}H NMR spectrum of compound **S31**.

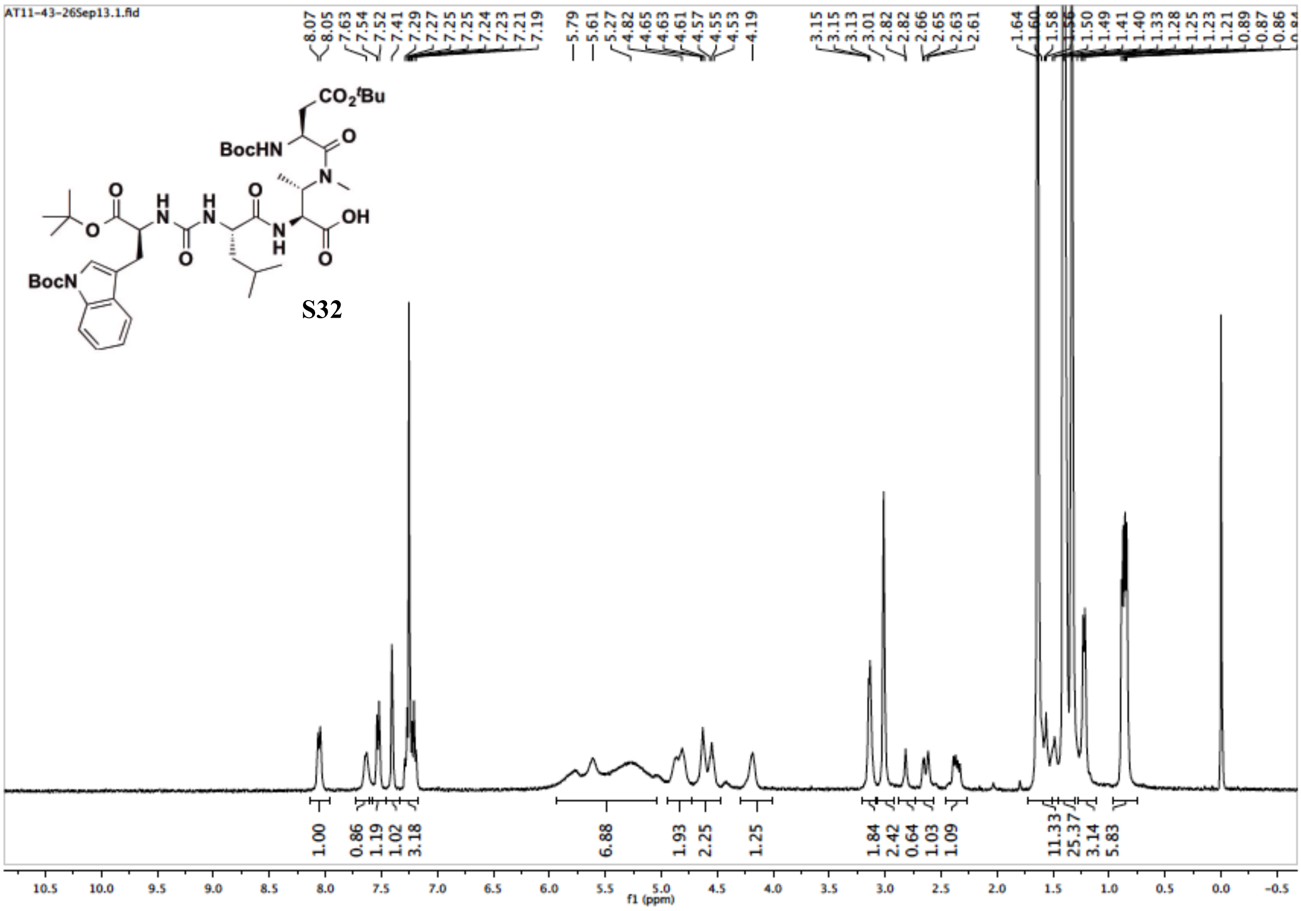

Supplementary Figure 71. ^{1}H NMR spectrum of compound **S32**.

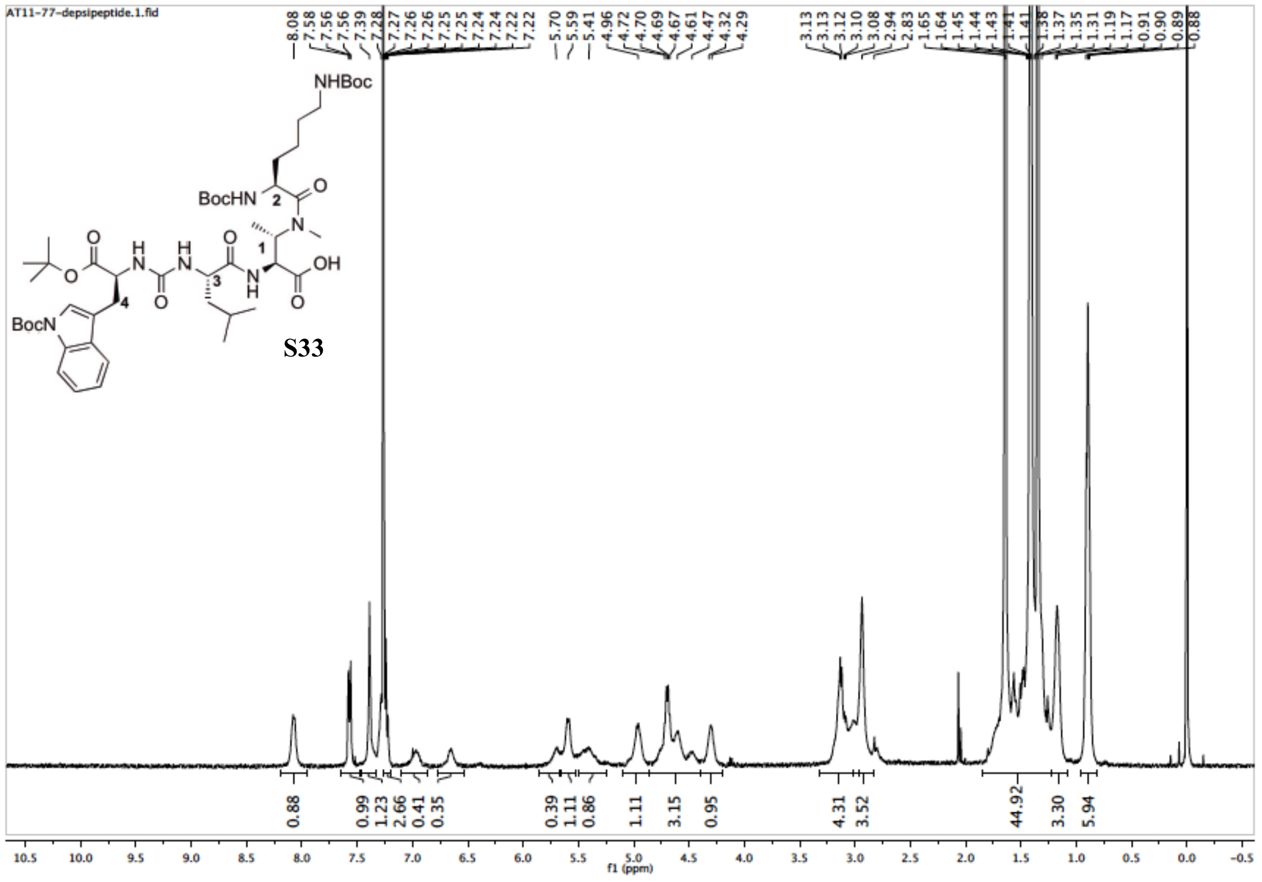

Supplementary Figure 72. ^{1}H NMR spectrum of compound **S33**.

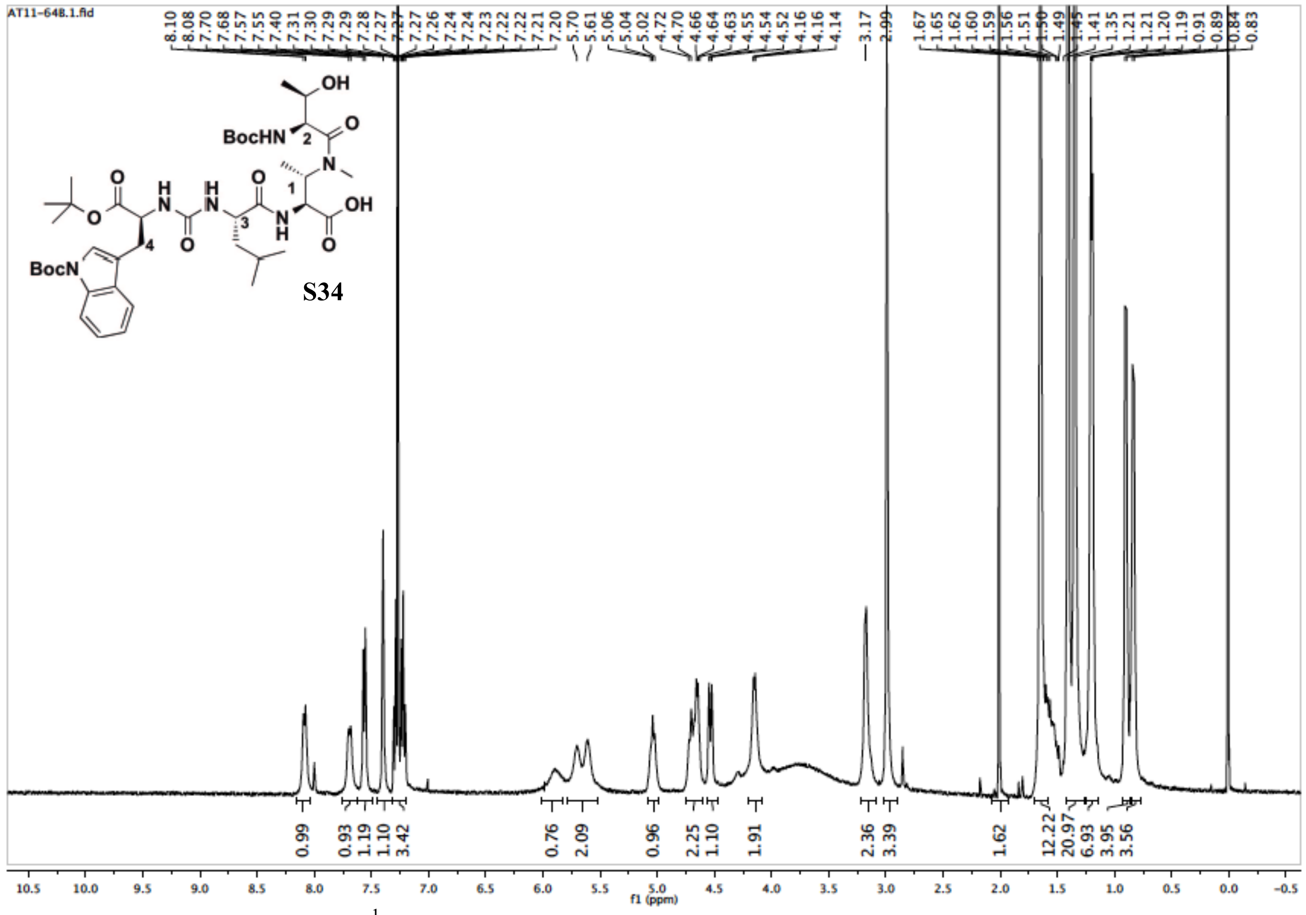

Supplementary Figure 73. ^{1}H NMR spectrum of compound **S34**.

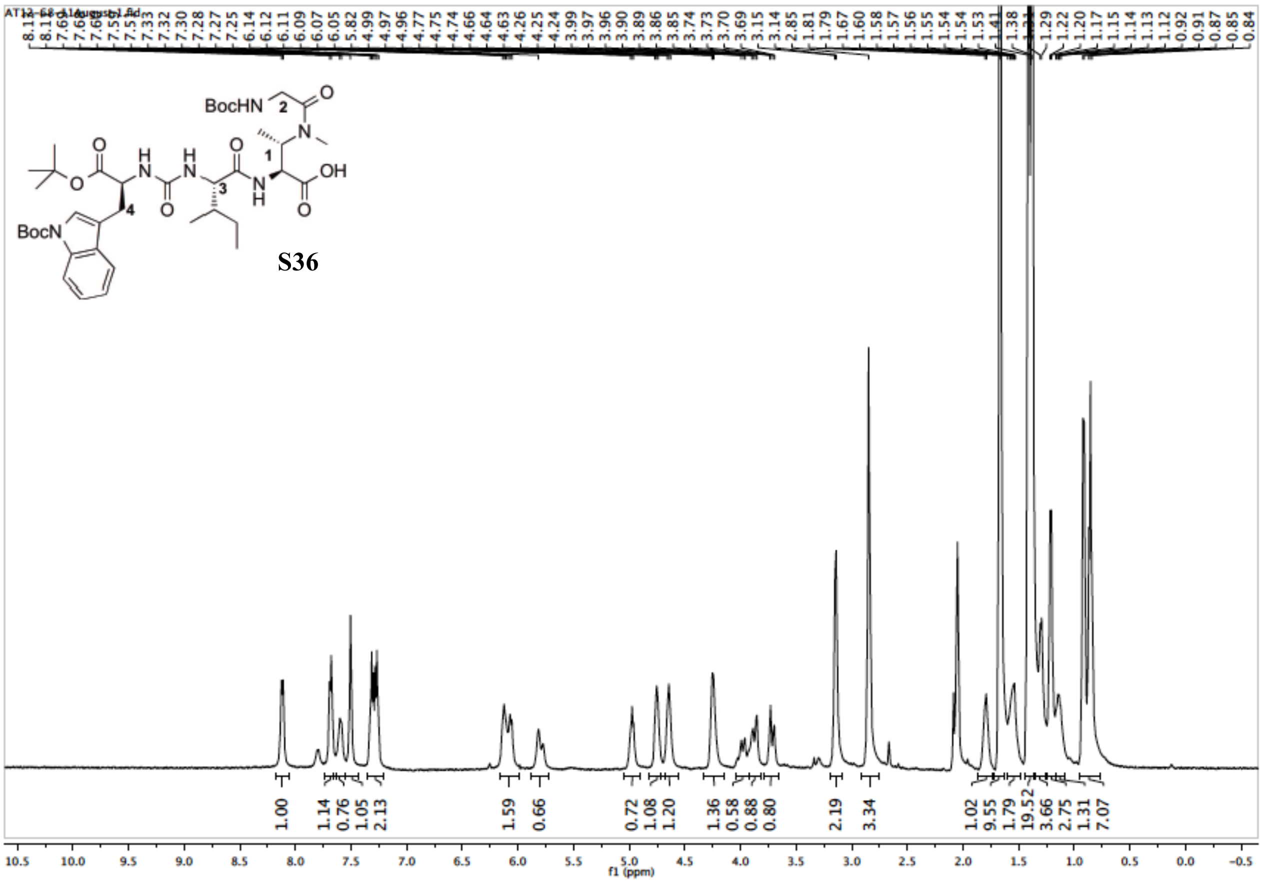

Supplementary Figure 74. ^{1}H NMR spectrum of compound **S36**.

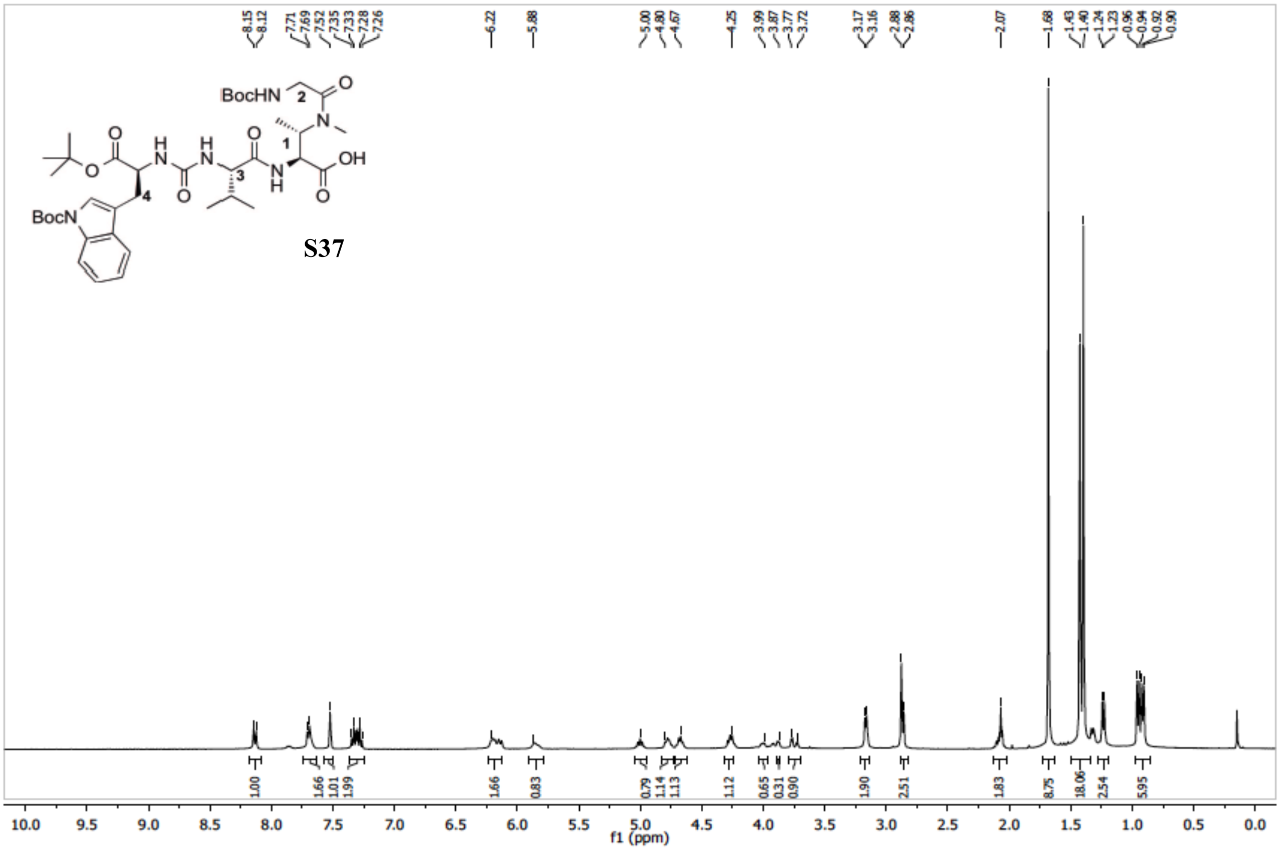

Supplementary Figure 75. ^{1}H NMR spectrum of compound **S37**.

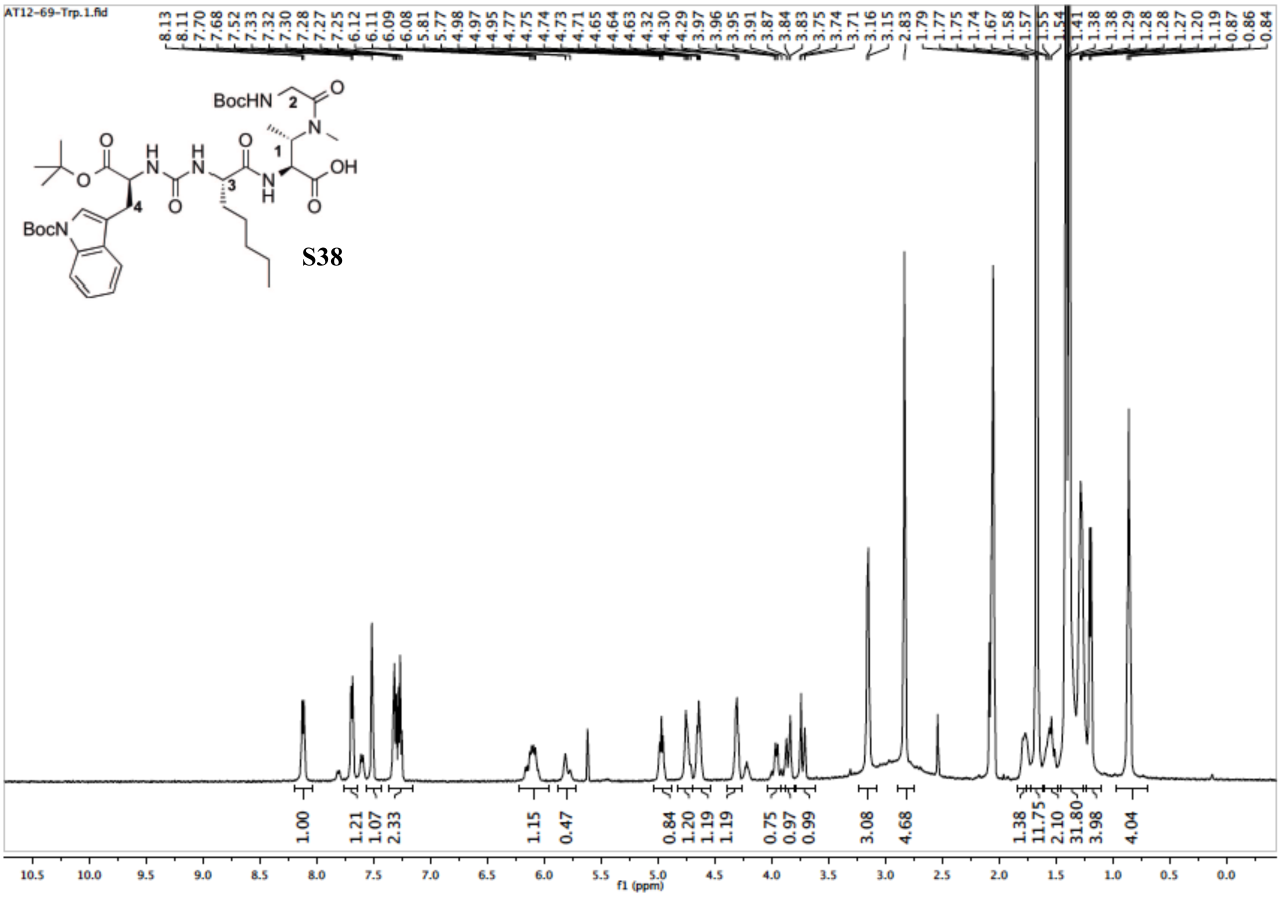

Supplementary Figure 76. ^{1}H NMR spectrum of compound **S38**.

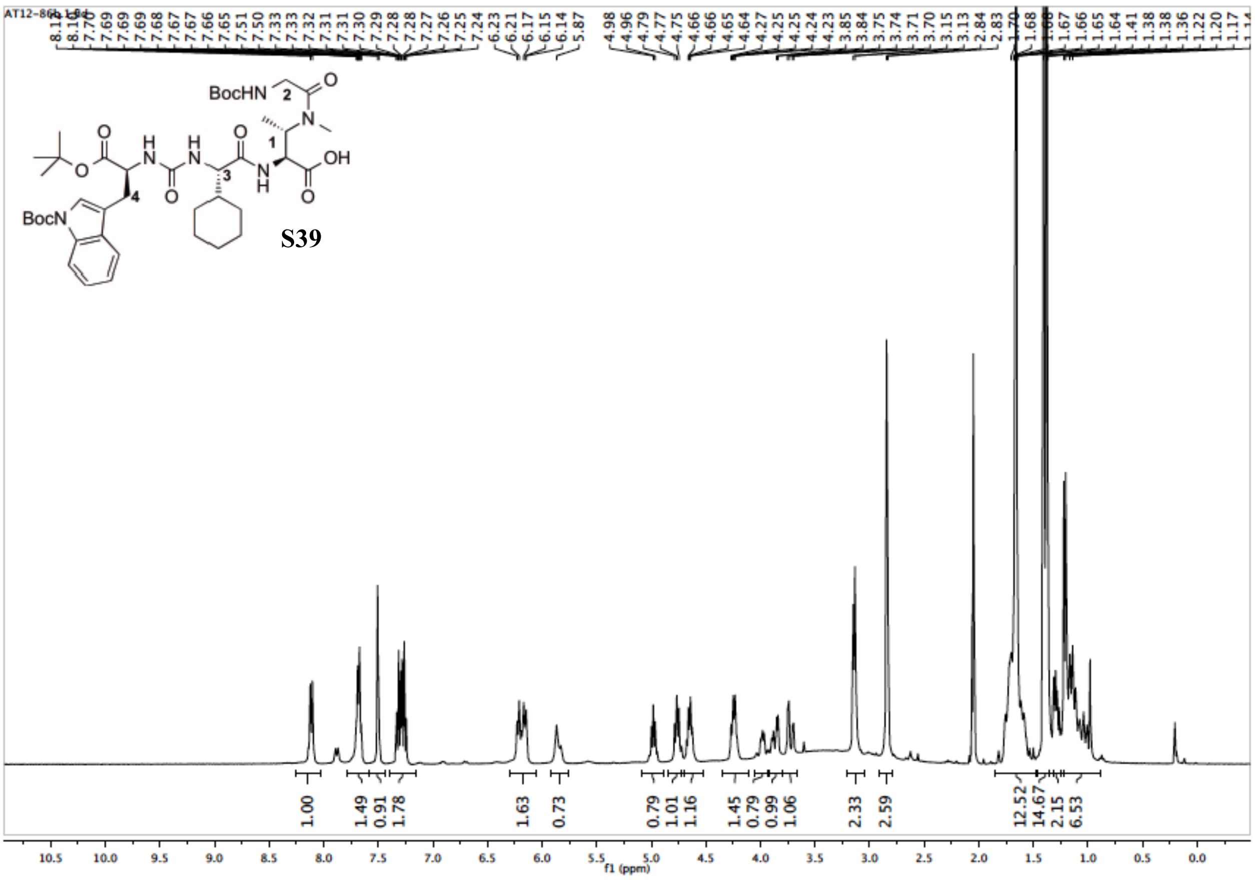

Supplementary Figure 77. ^{1}H NMR spectrum of compound **S39**.

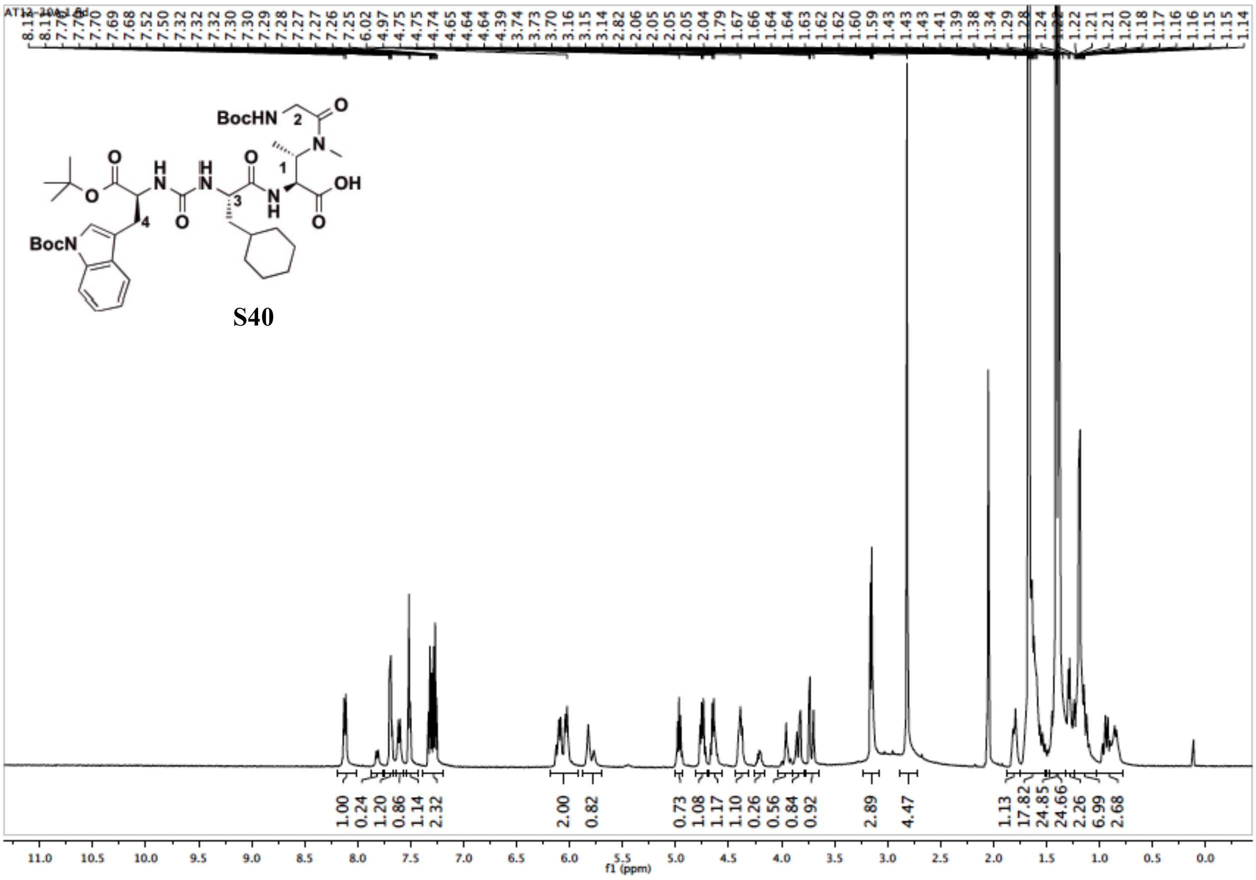

Supplementary Figure 78. ^{1}H NMR spectrum of compound **S40**.

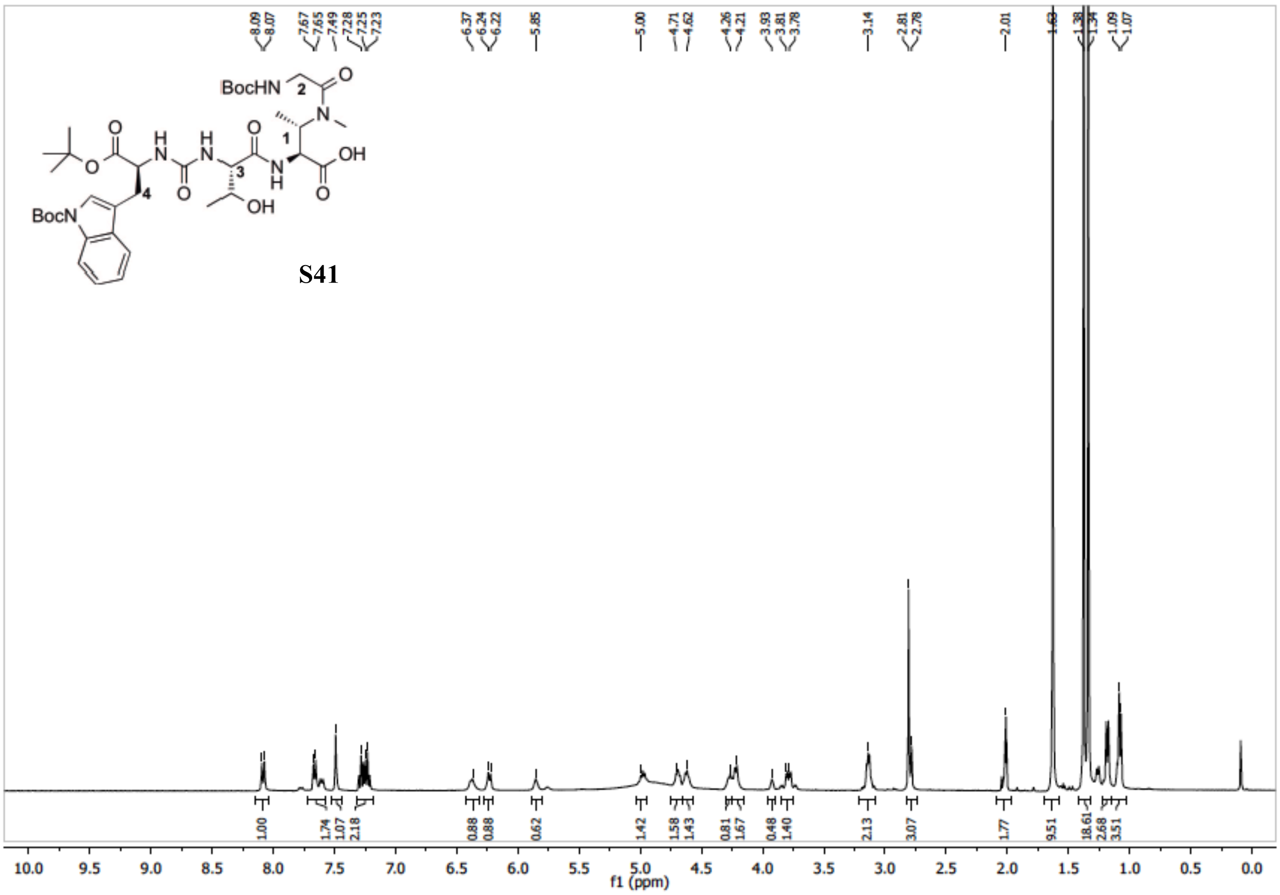

Supplementary Figure 79. ^{1}H NMR spectrum of compound **S41**.

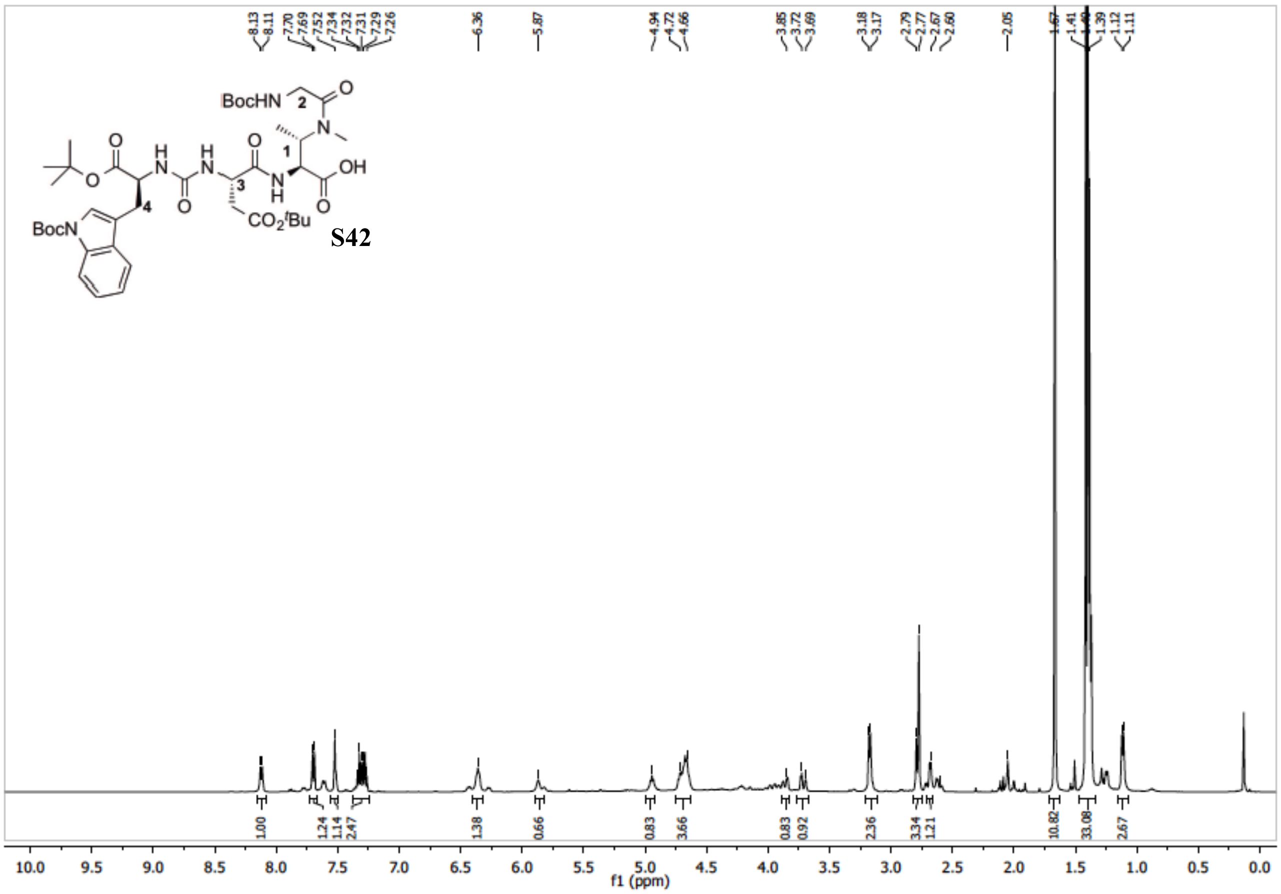

Supplementary Figure 80. ^{1}H NMR spectrum of compound **S42**.

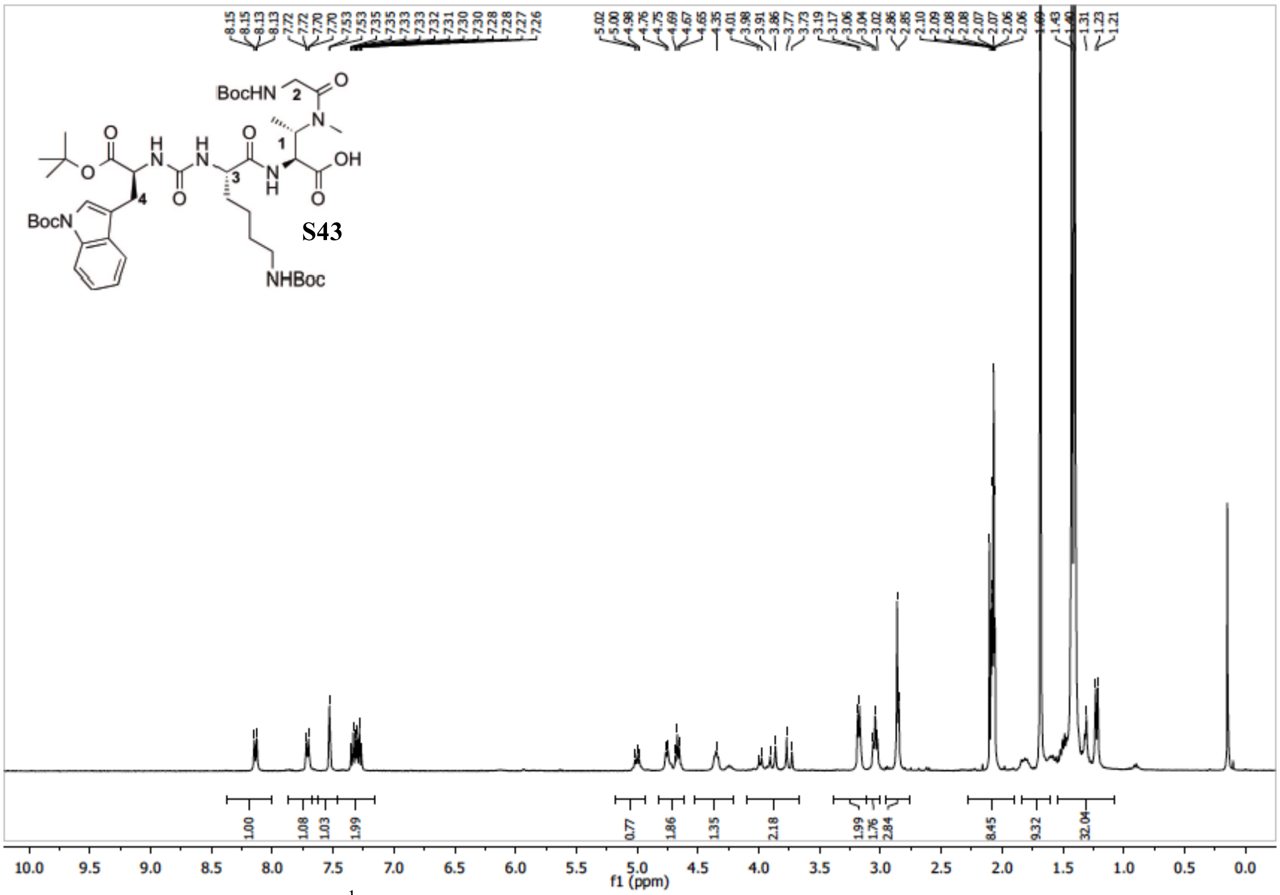

Supplementary Figure 81. ^{1}H NMR spectrum of compound **S43**.

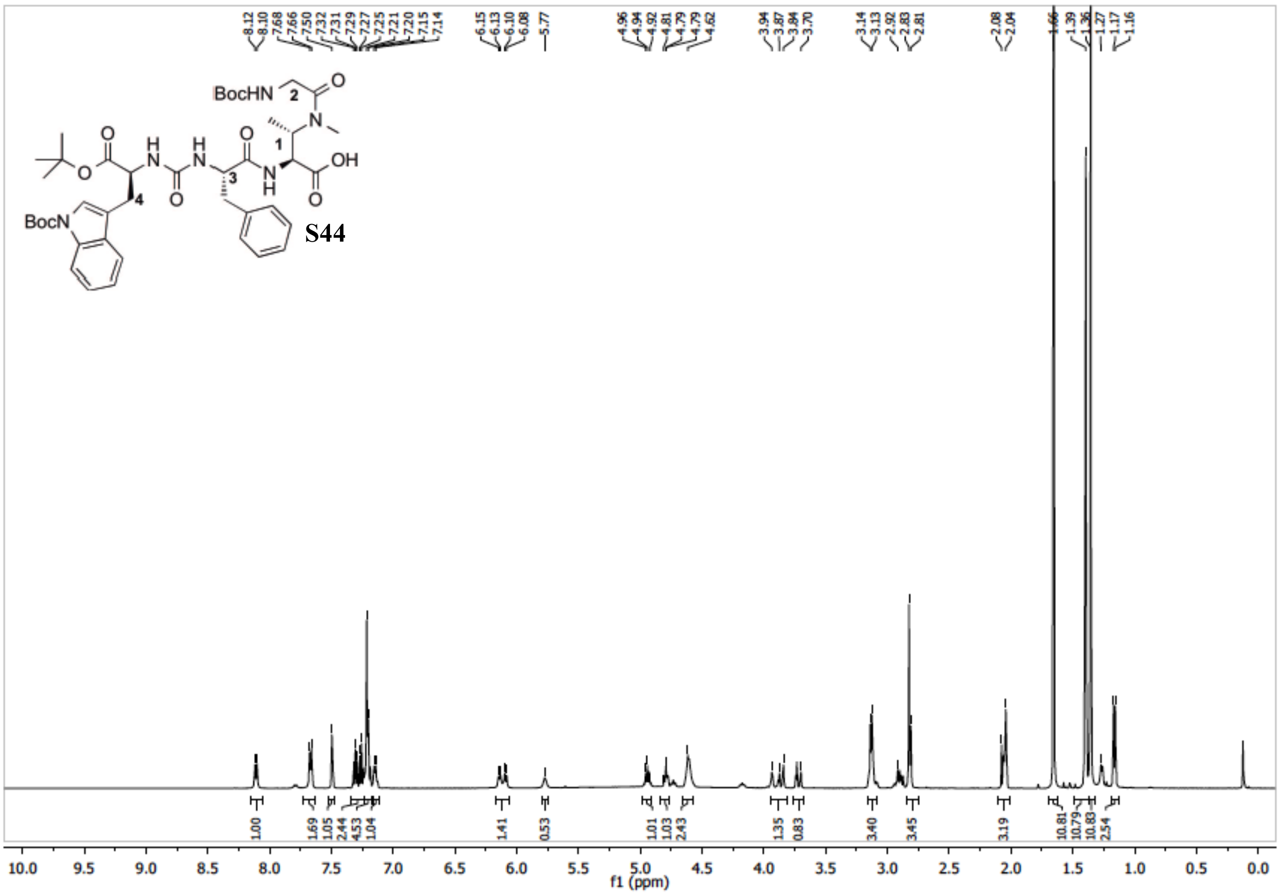

Supplementary Figure 82. ^{1}H NMR spectrum of compound **S44**.

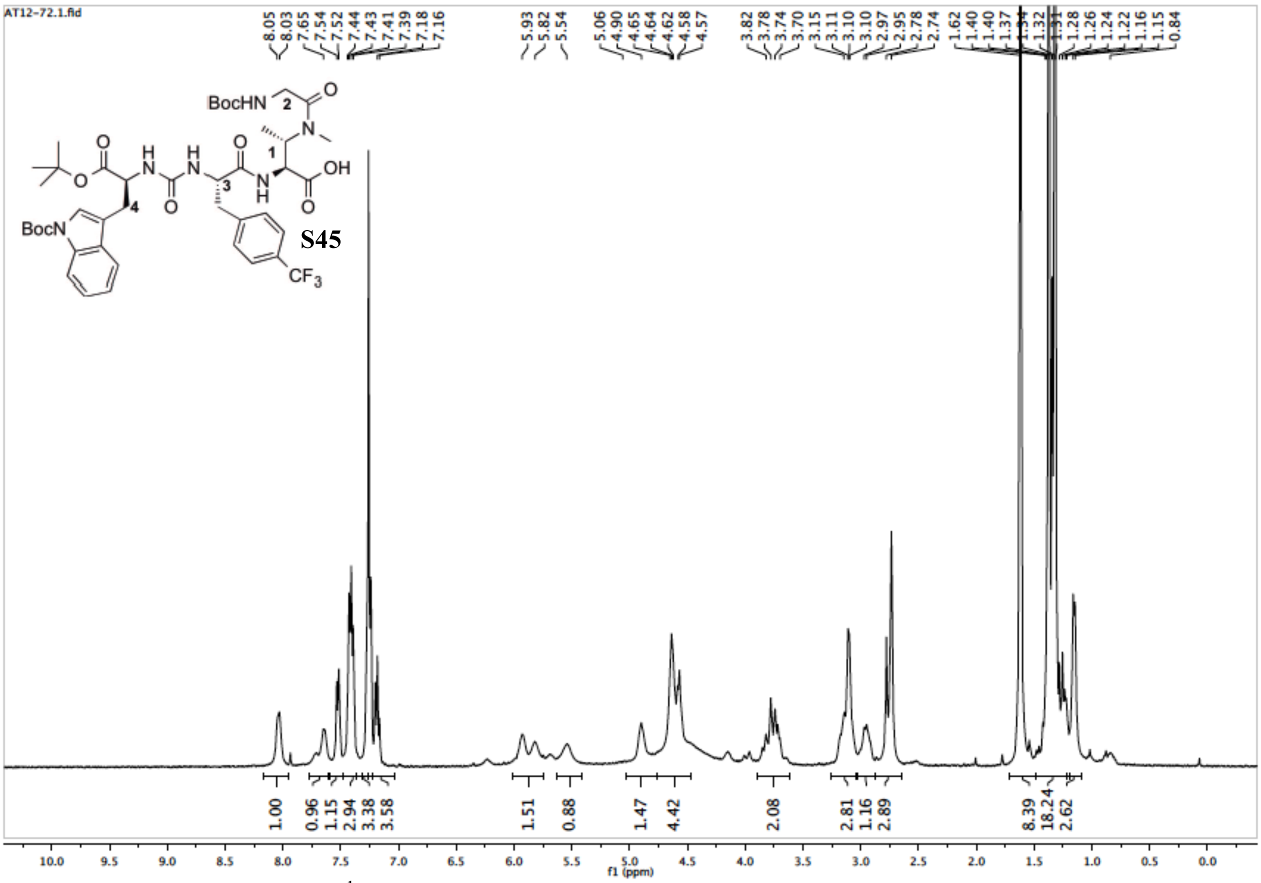

Supplementary Figure 83. ^{1}H NMR spectrum of compound **S45**.

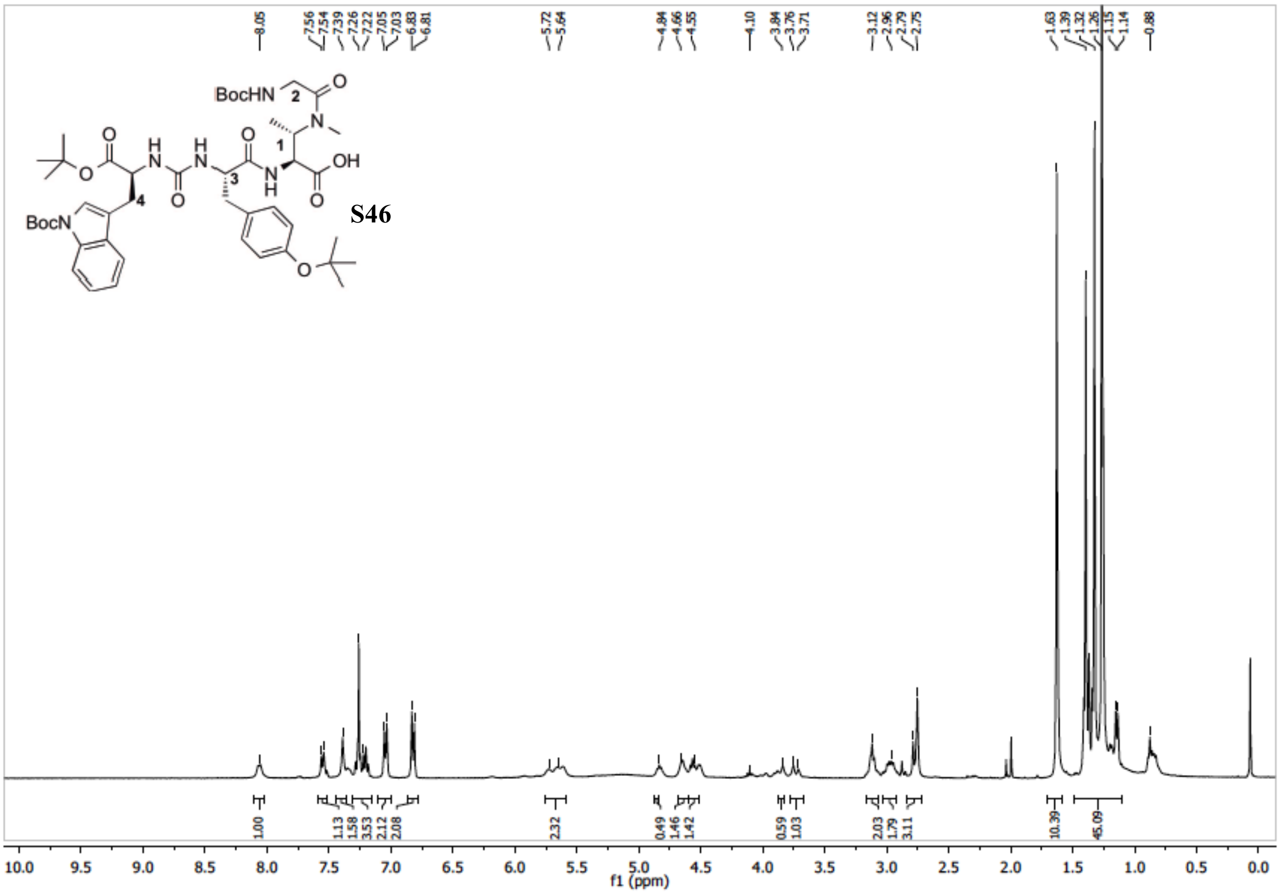

Supplementary Figure 84. ^{1}H NMR spectrum of compound **S46**.

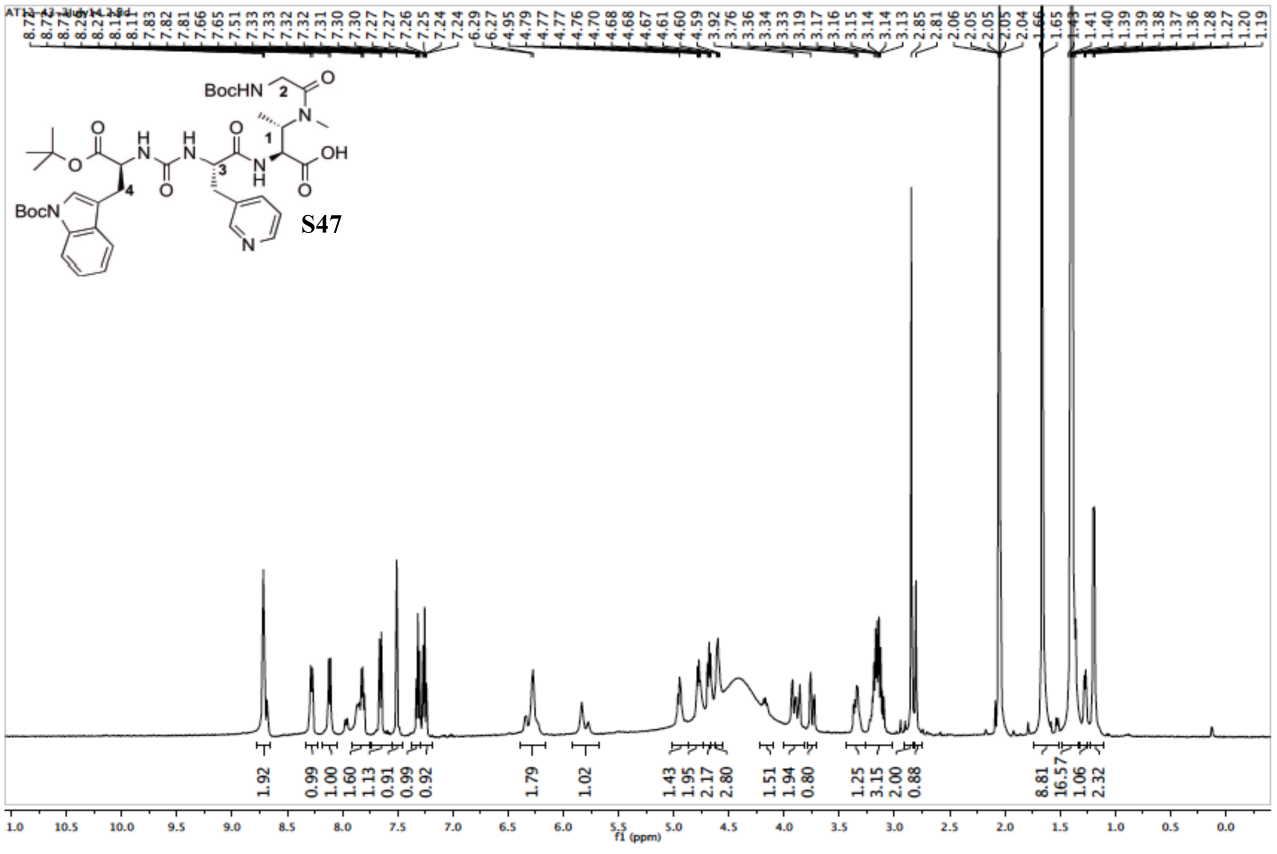

Supplementary Figure 85. ^{1}H NMR spectrum of compound **S47**.

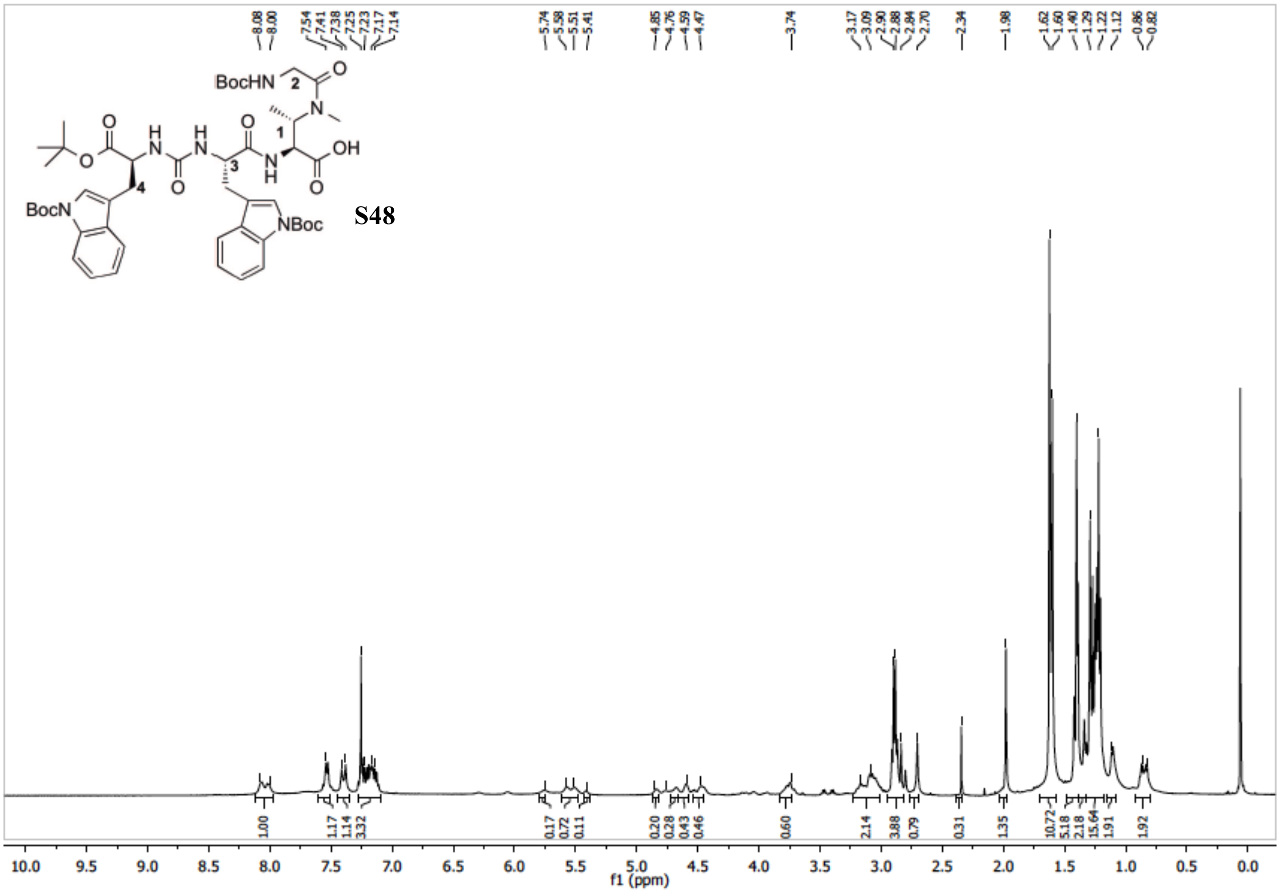

Supplementary Figure 86. ^{1}H NMR spectrum of compound **S48**.

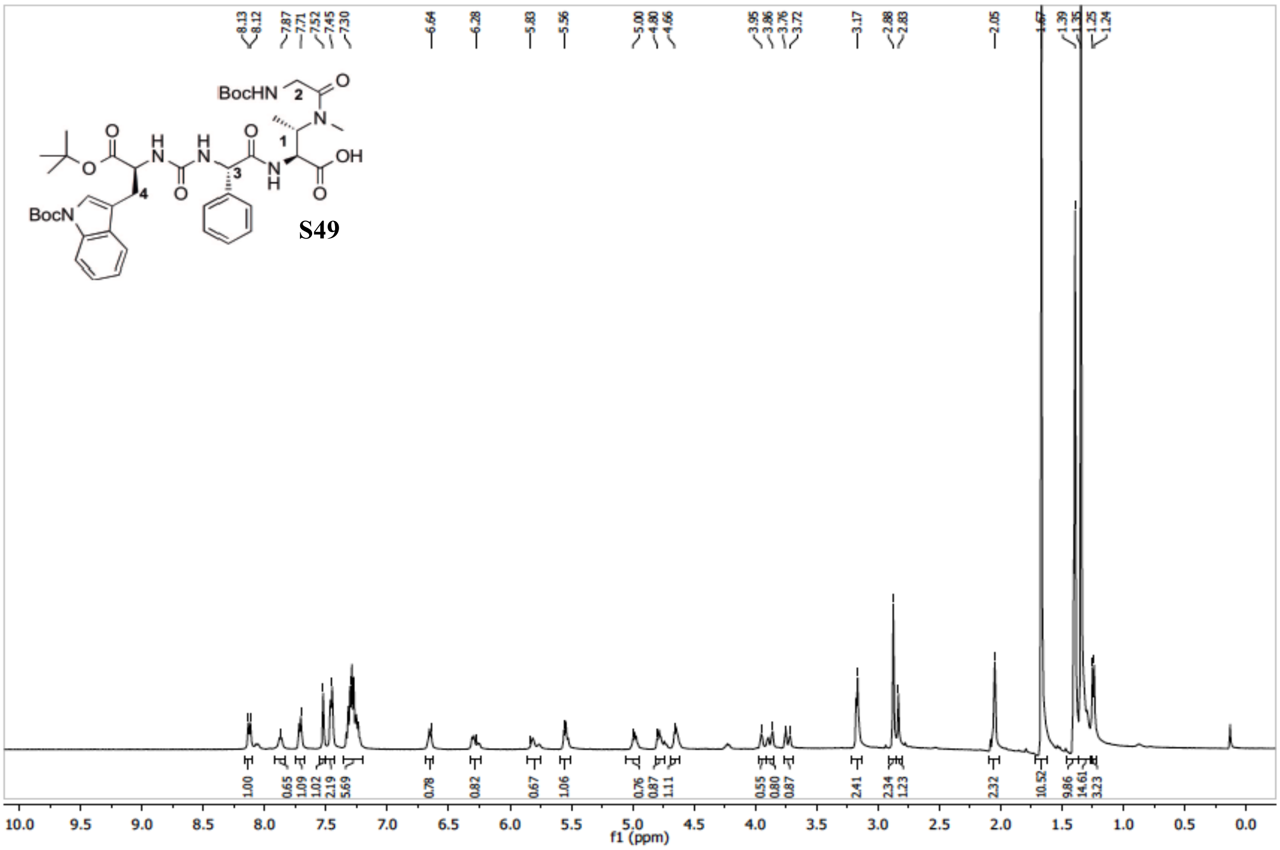

Supplementary Figure 87. ^{1}H NMR spectrum of compound **S49**.

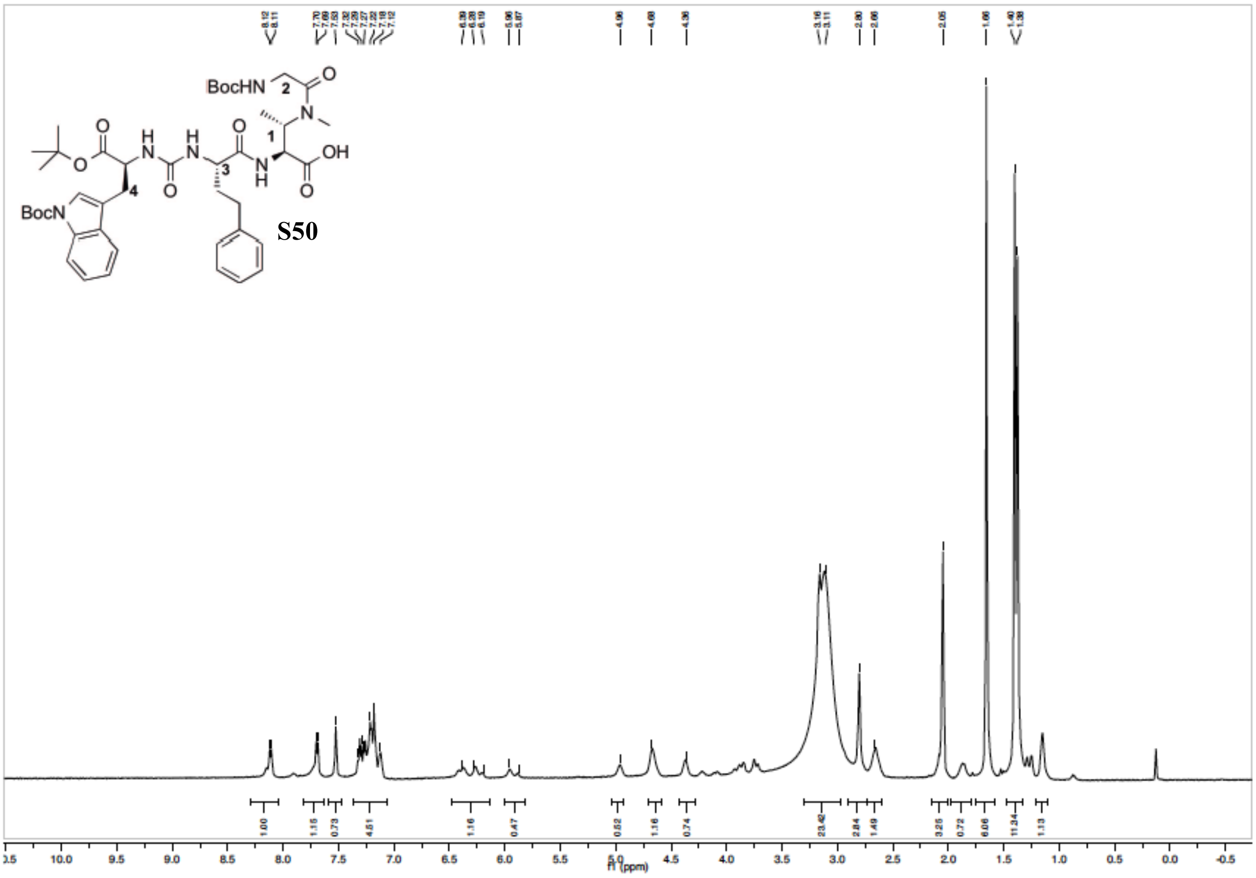

Supplementary Figure 88. ^{1}H NMR spectrum of compound **S50**.

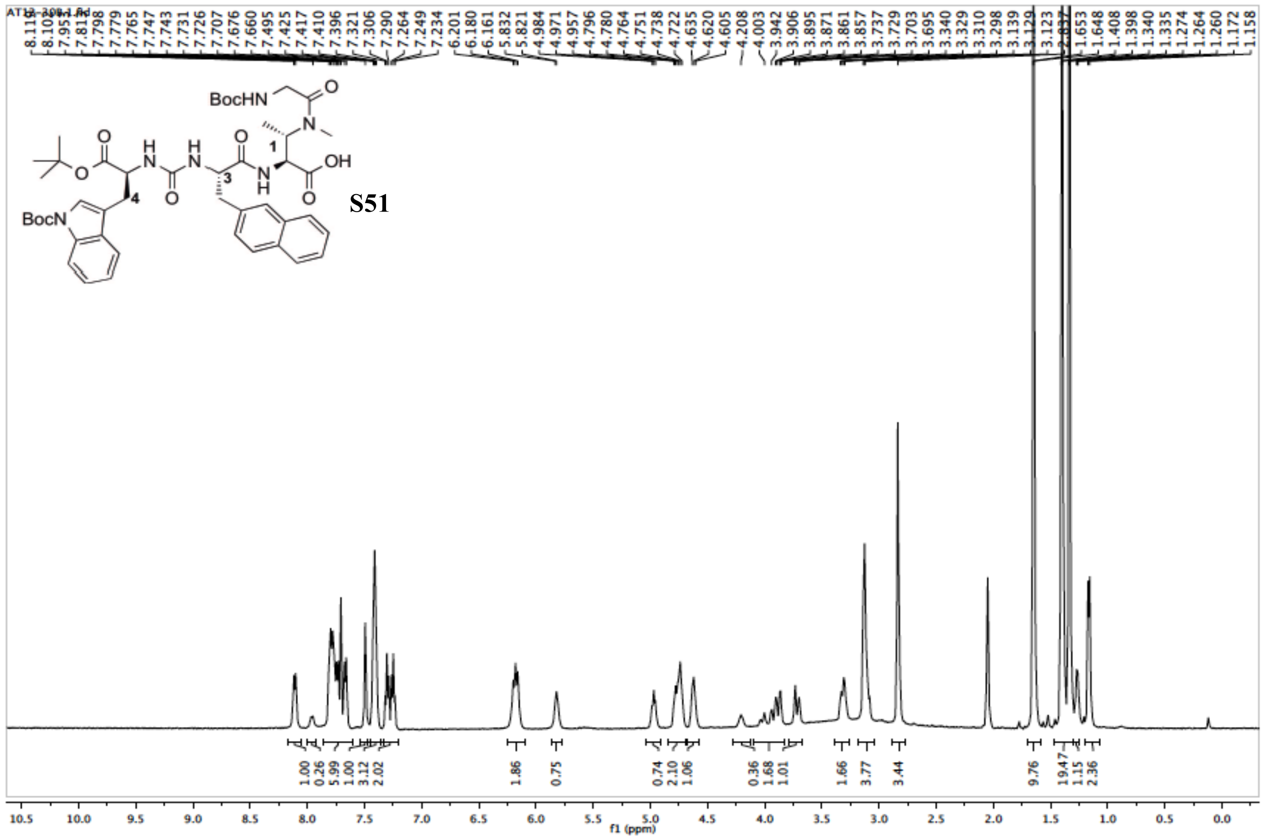

Supplementary Figure 89. ^{1}H NMR spectrum of compound **S51**.

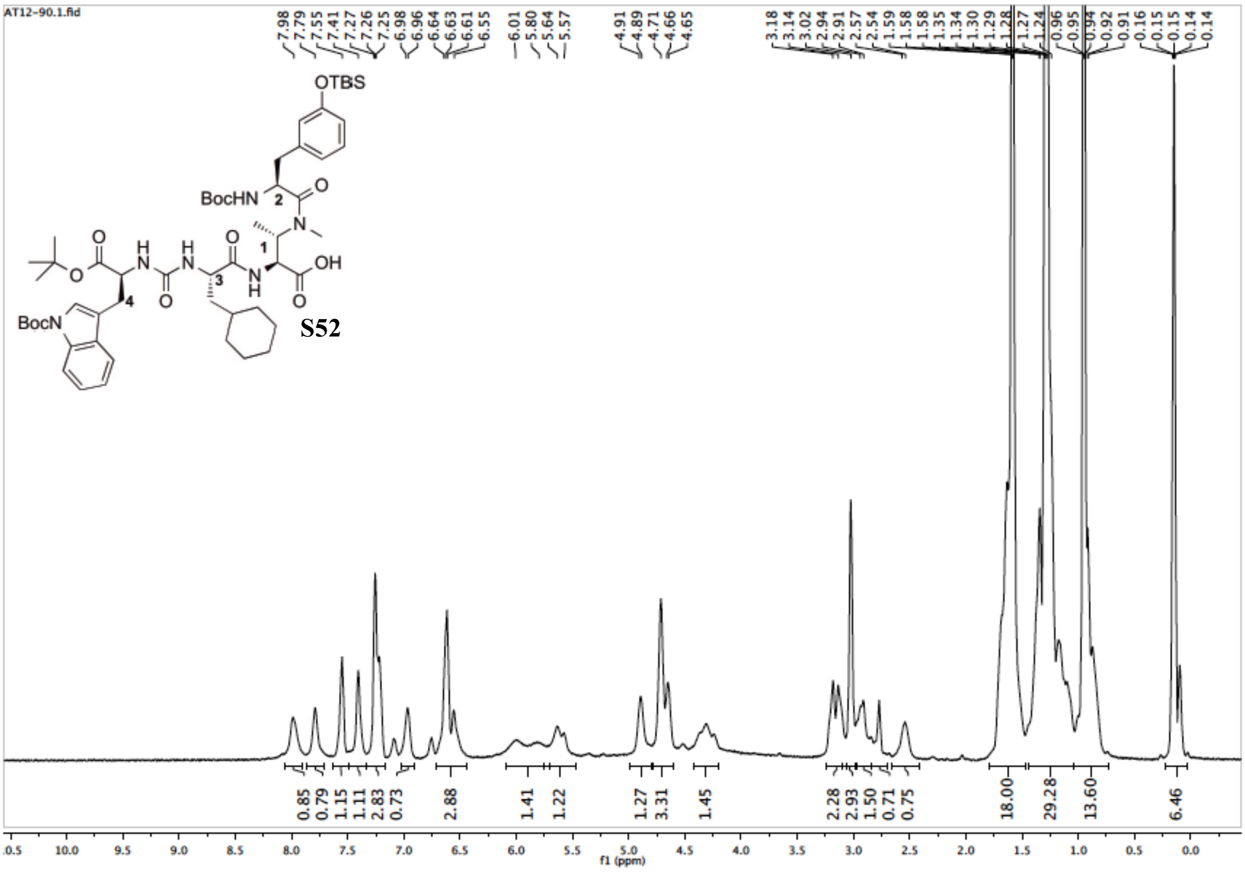

Supplementary Figure 90. ^{1}H NMR spectrum of compound **S52**.

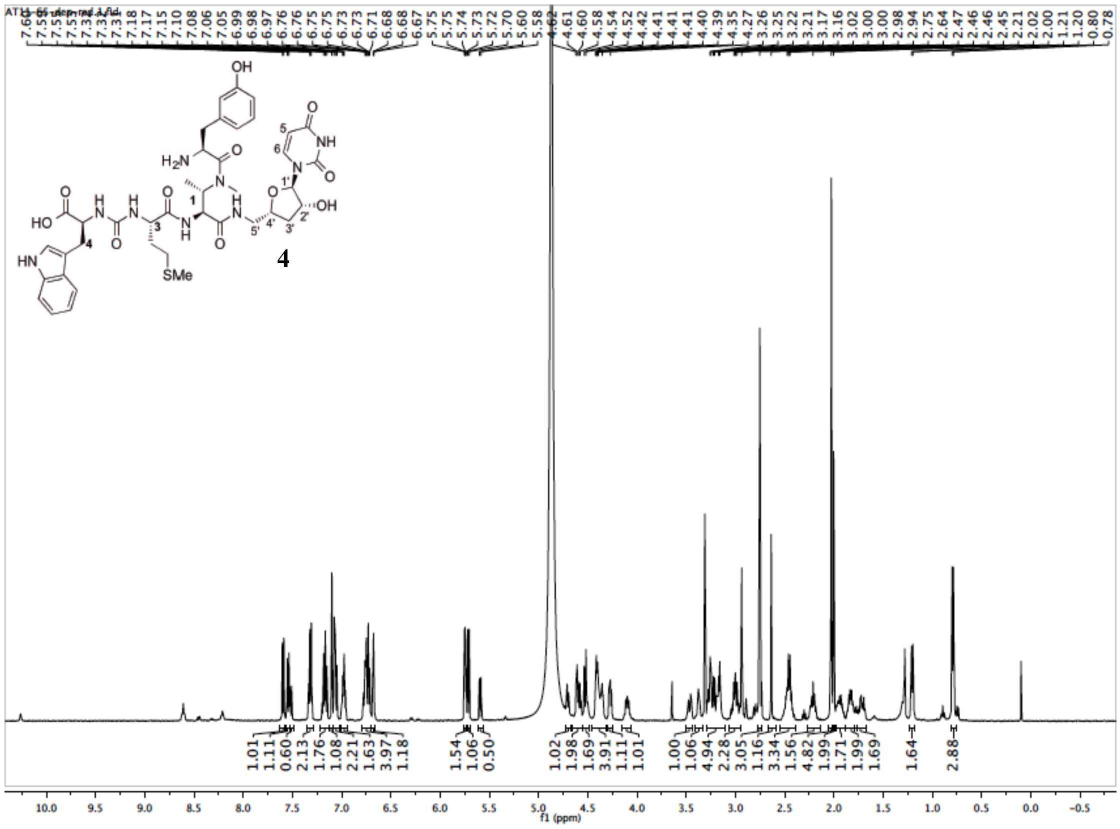

Supplementary Figure 91. ^{1}H NMR spectrum of compound **4**.

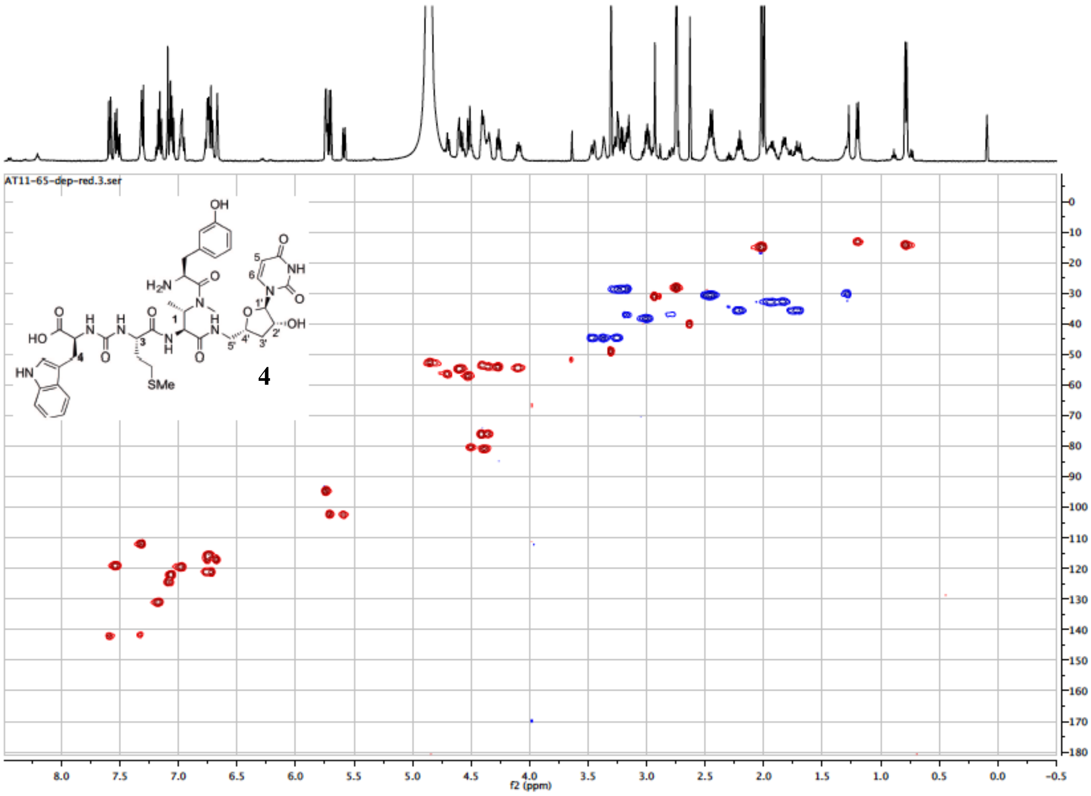

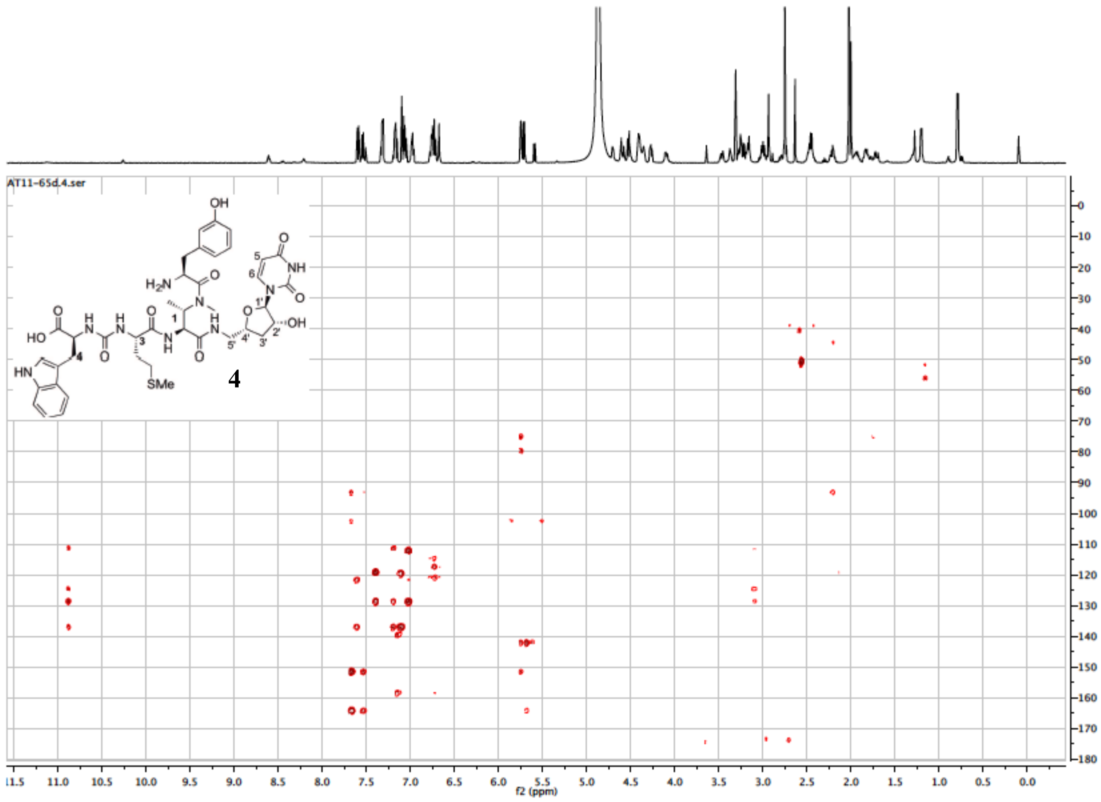

Supplementary Figure 92. HSQC (top) and HMBC (bottom) NMR spectra of compound **4**.

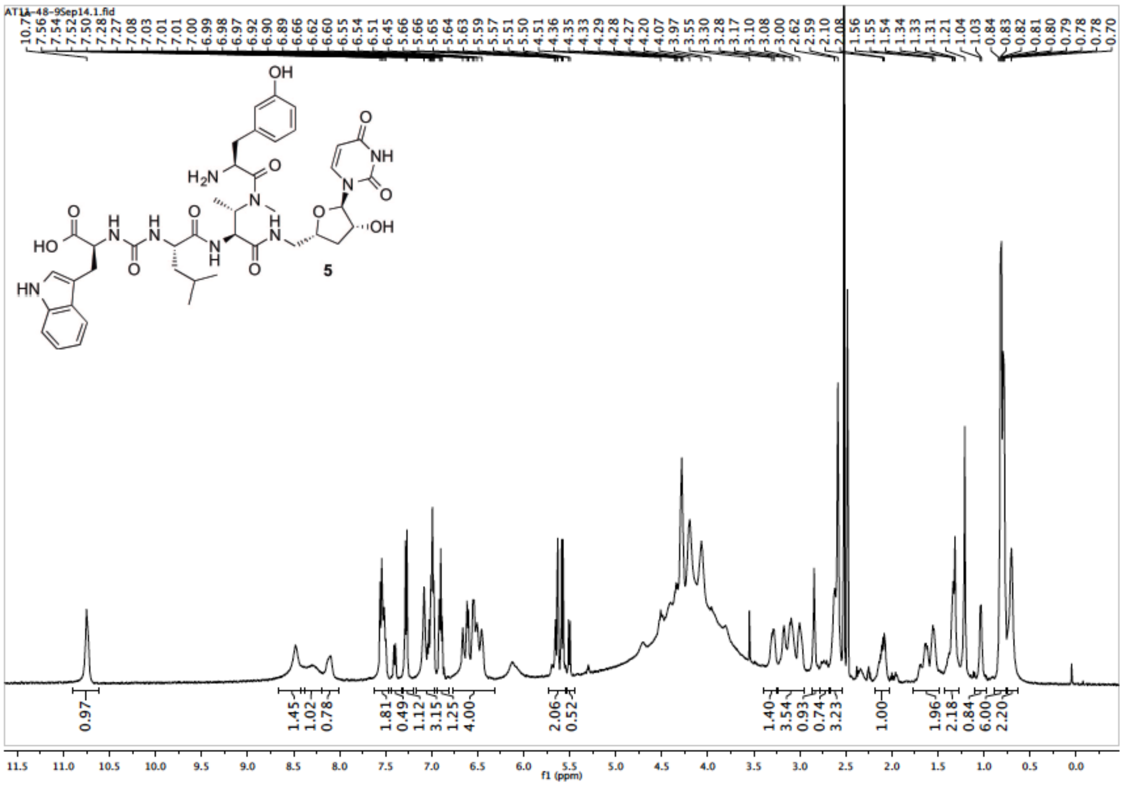

Supplementary Figure 93. ^{1}H NMR spectrum of compound **5**.

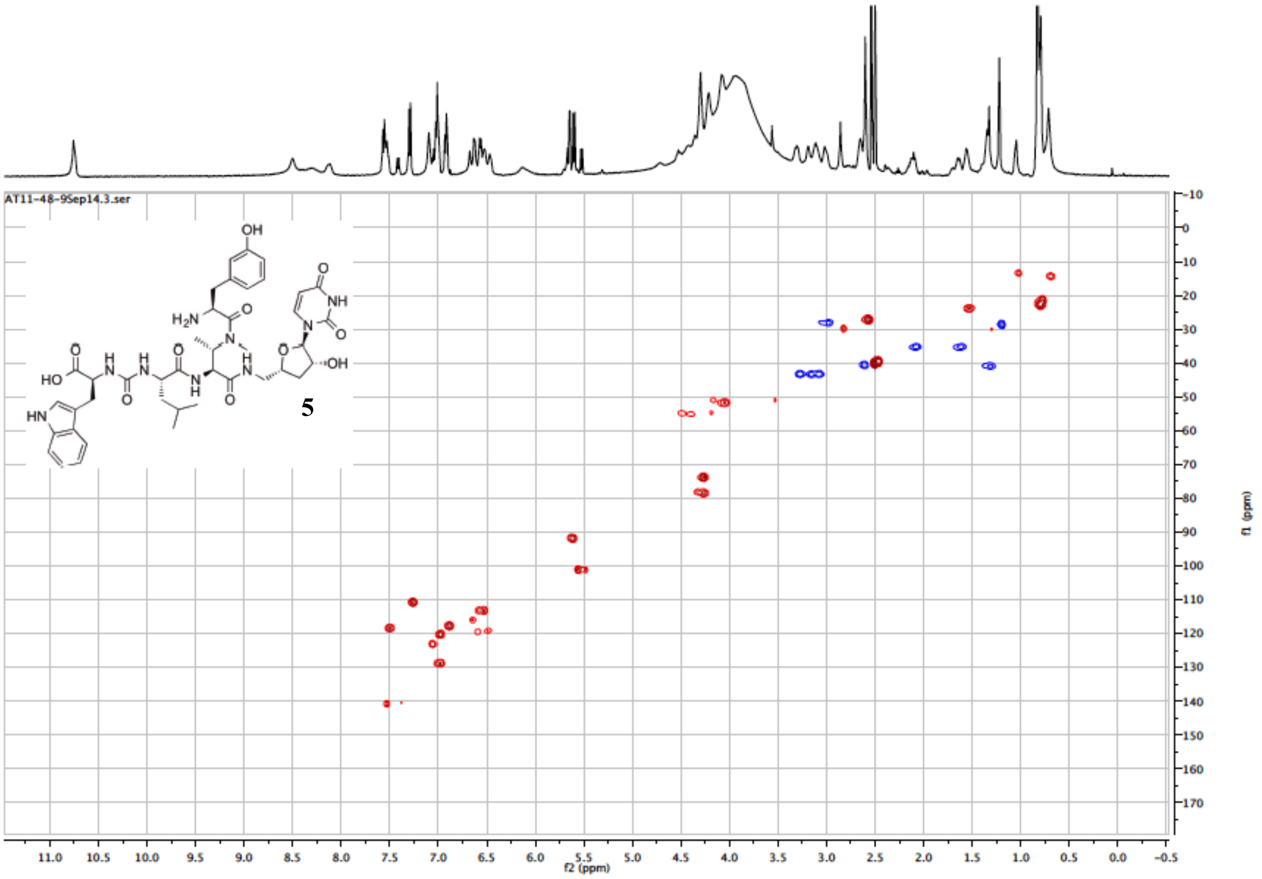

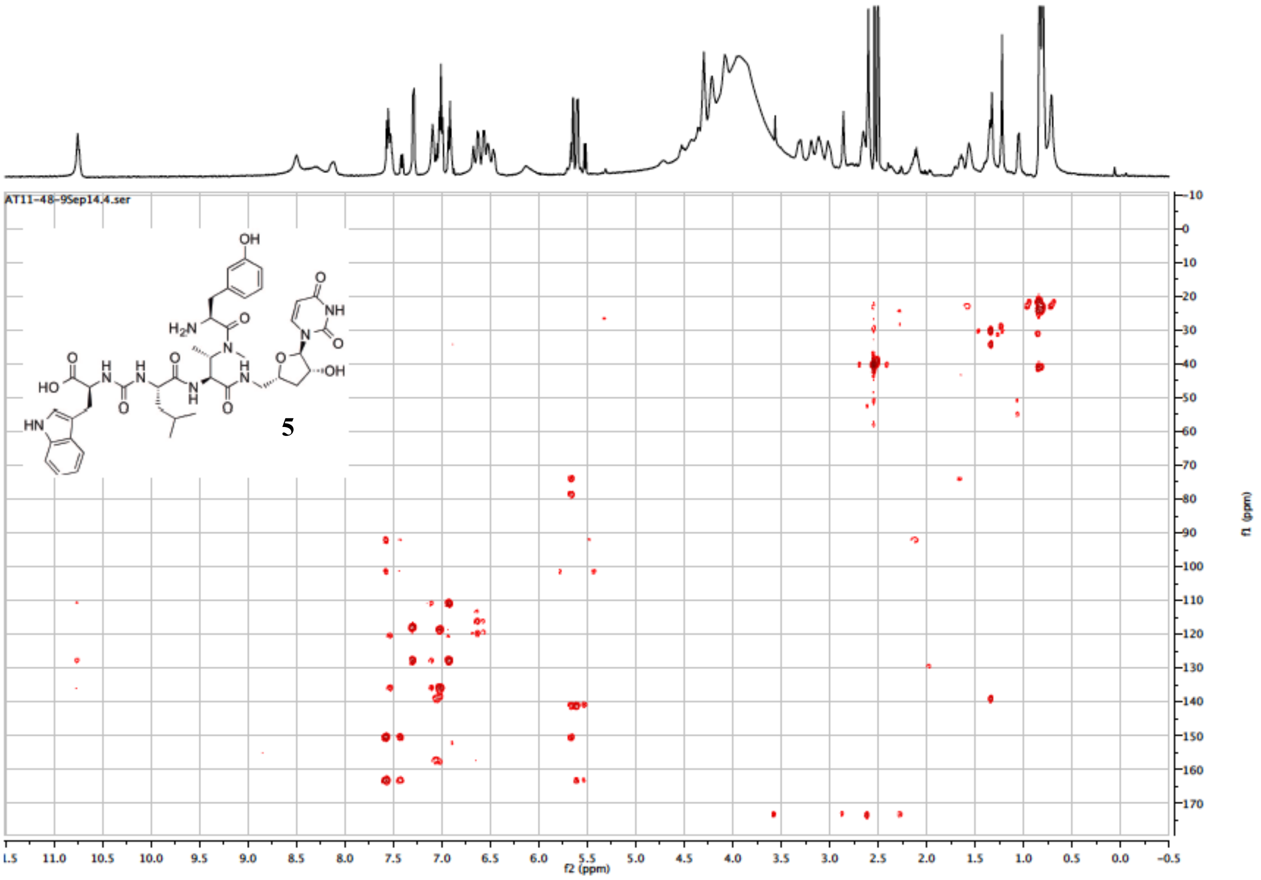

Supplementary Figure 94. HSQC (top) and HMBC (bottom) NMR spectra of compound **5**.

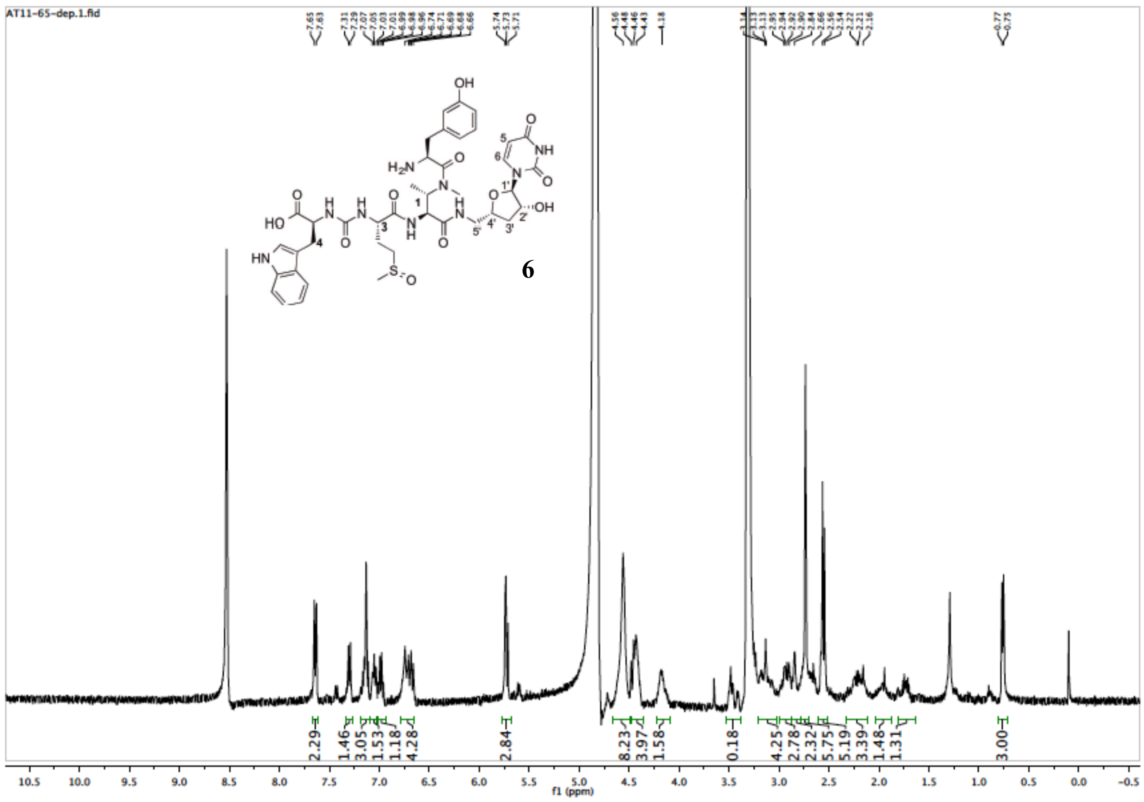

Supplementary Figure 95. ^{1}H NMR spectrum of compound **6**.

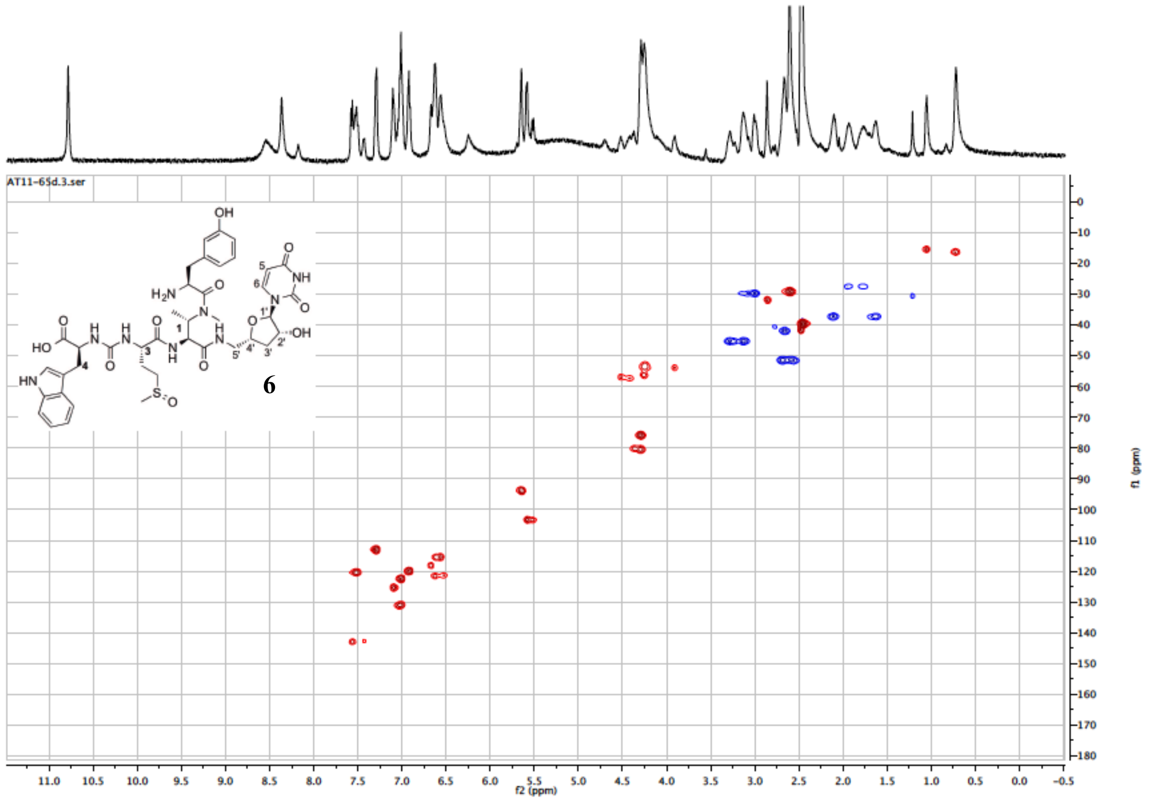

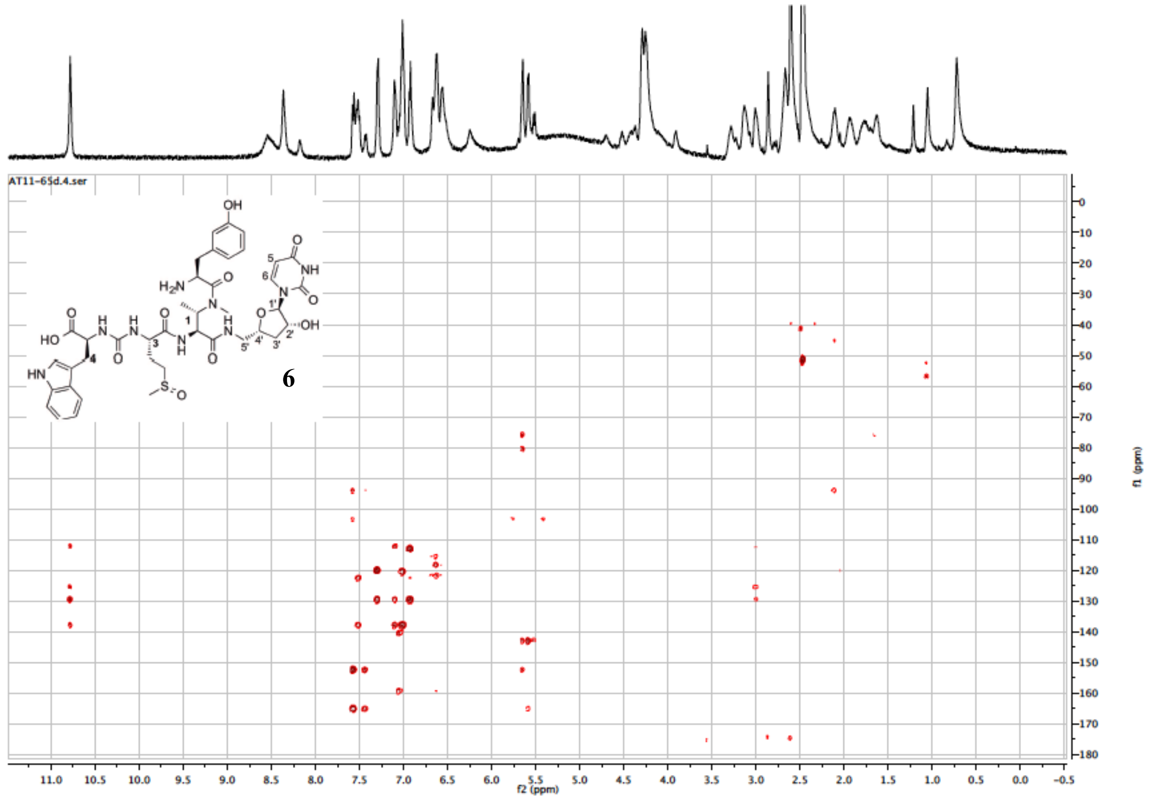

Supplementary Figure 96. HSQC (top) and HMBC (bottom) NMR spectra of compound **6**.

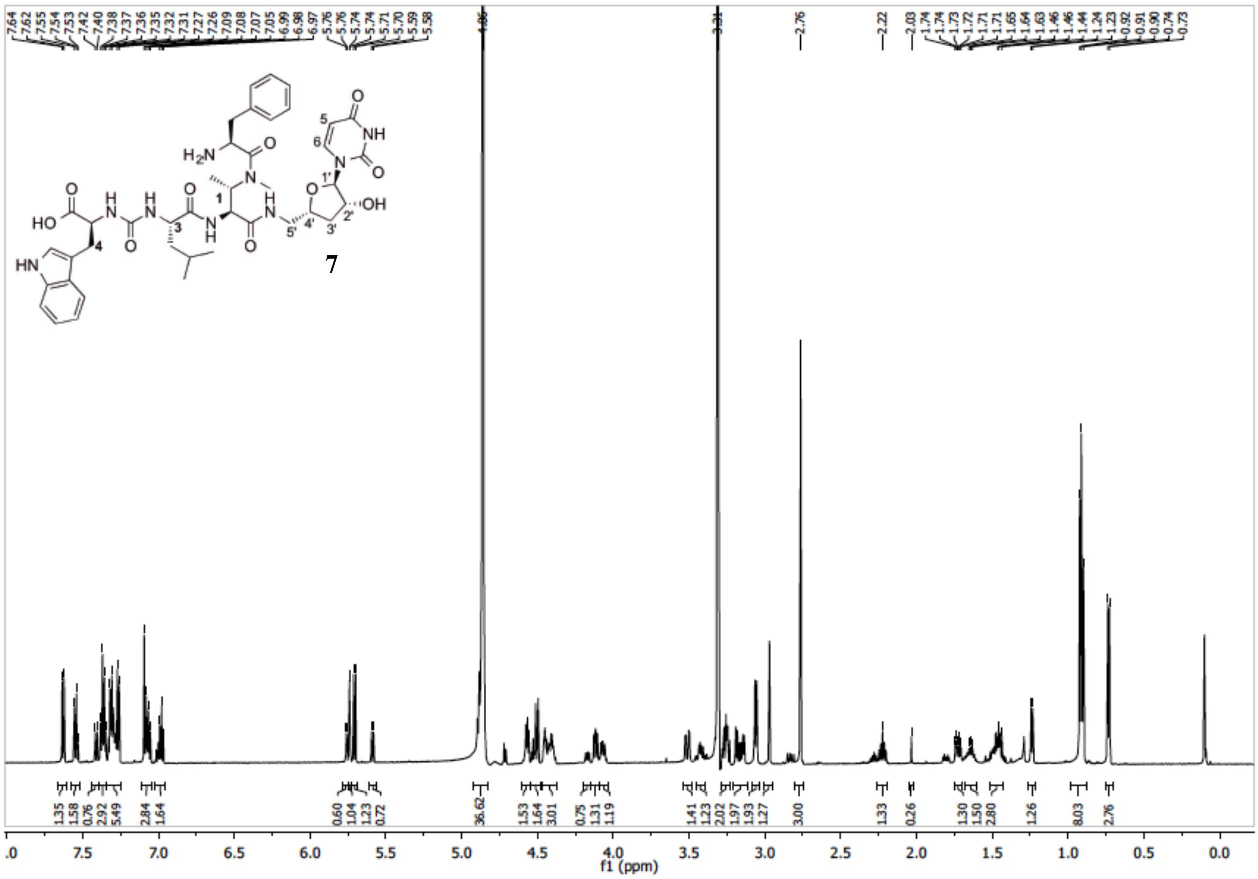

Supplementary Figure 97. ^{1}H NMR spectrum of compound **7**.

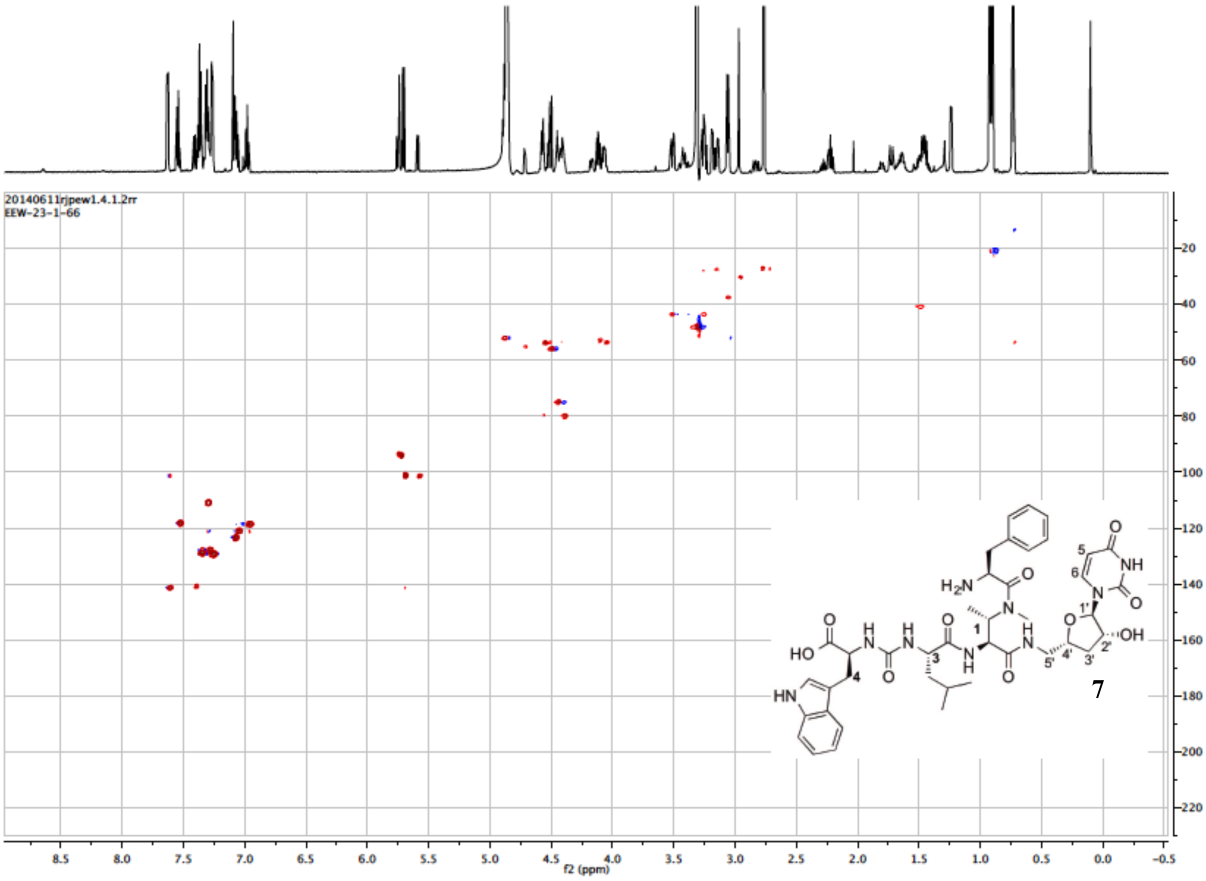

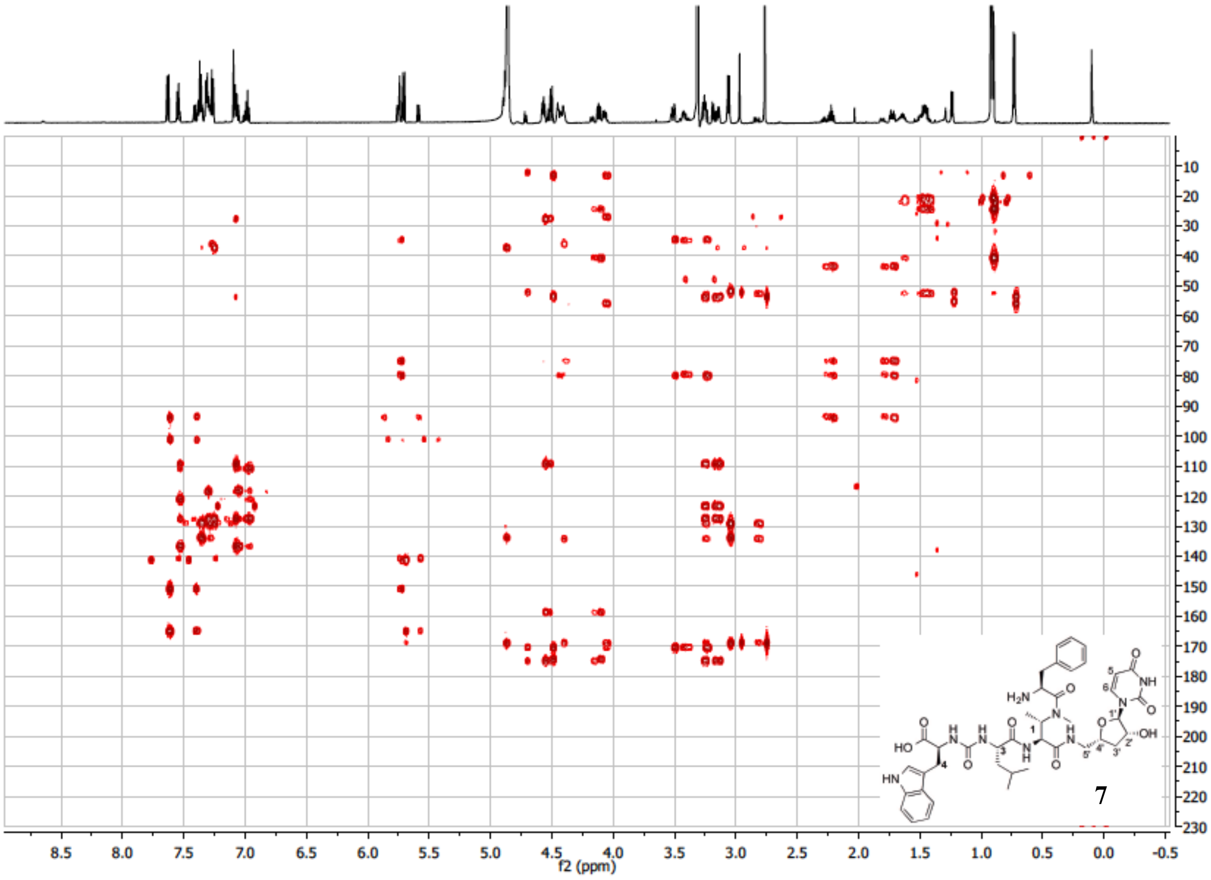

Supplementary Figure 98. HSQC (top) and HMBC (bottom) NMR spectra of compound **7**.

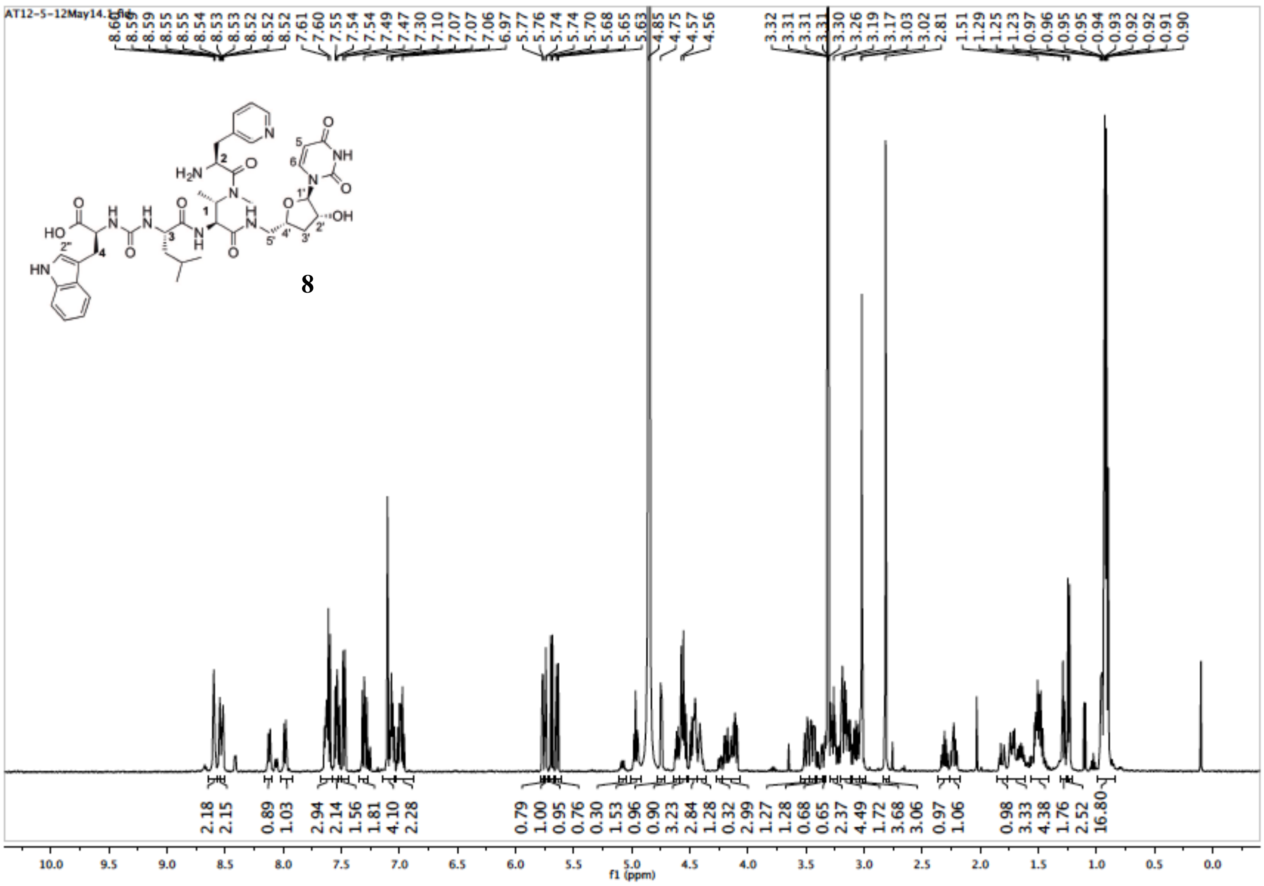

Supplementary Figure 99. ^{1}H NMR spectrum of compound **8**.

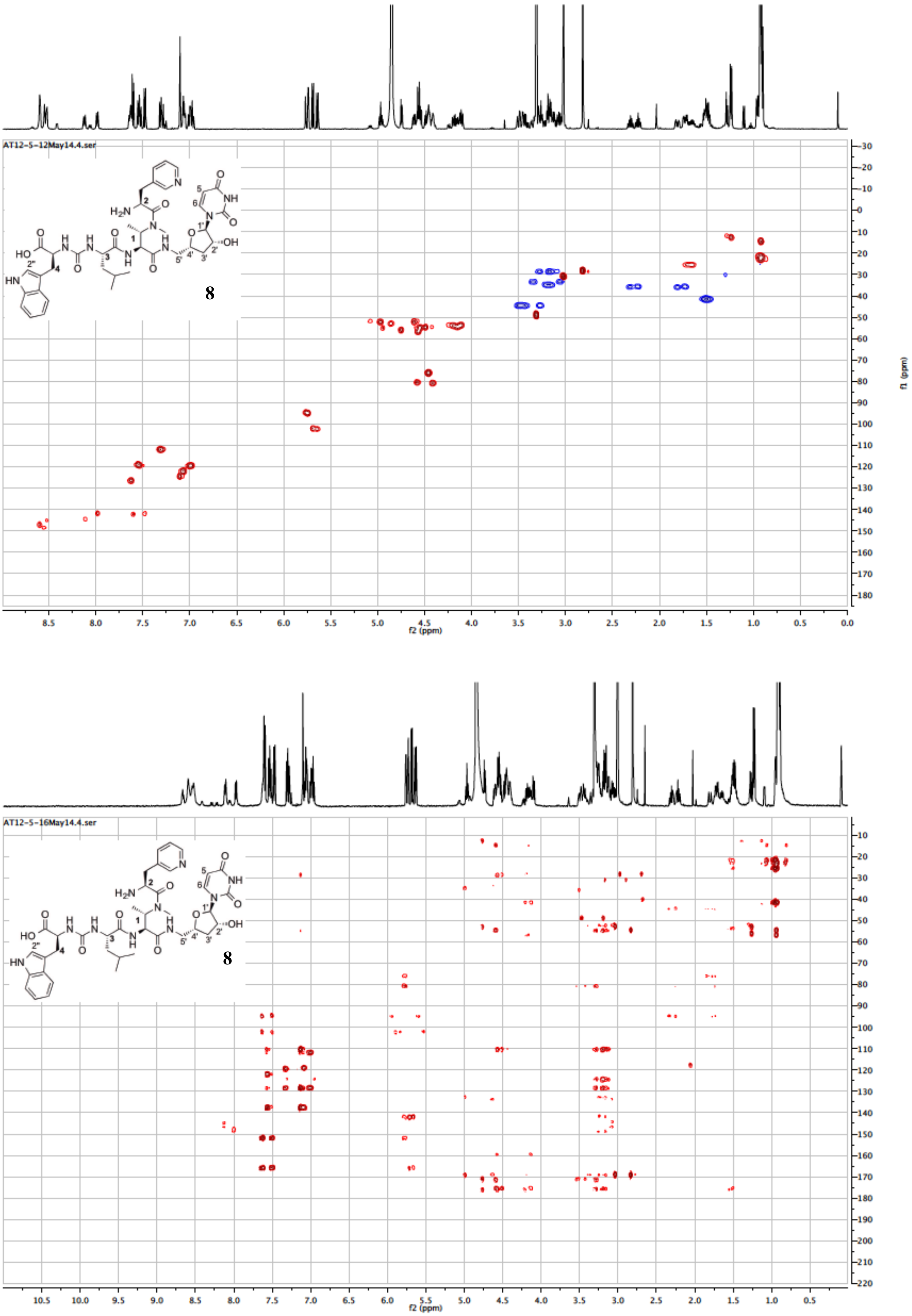

Supplementary Figure 100. HSQC (top) and HMBC (bottom) NMR spectra of compound **8**.

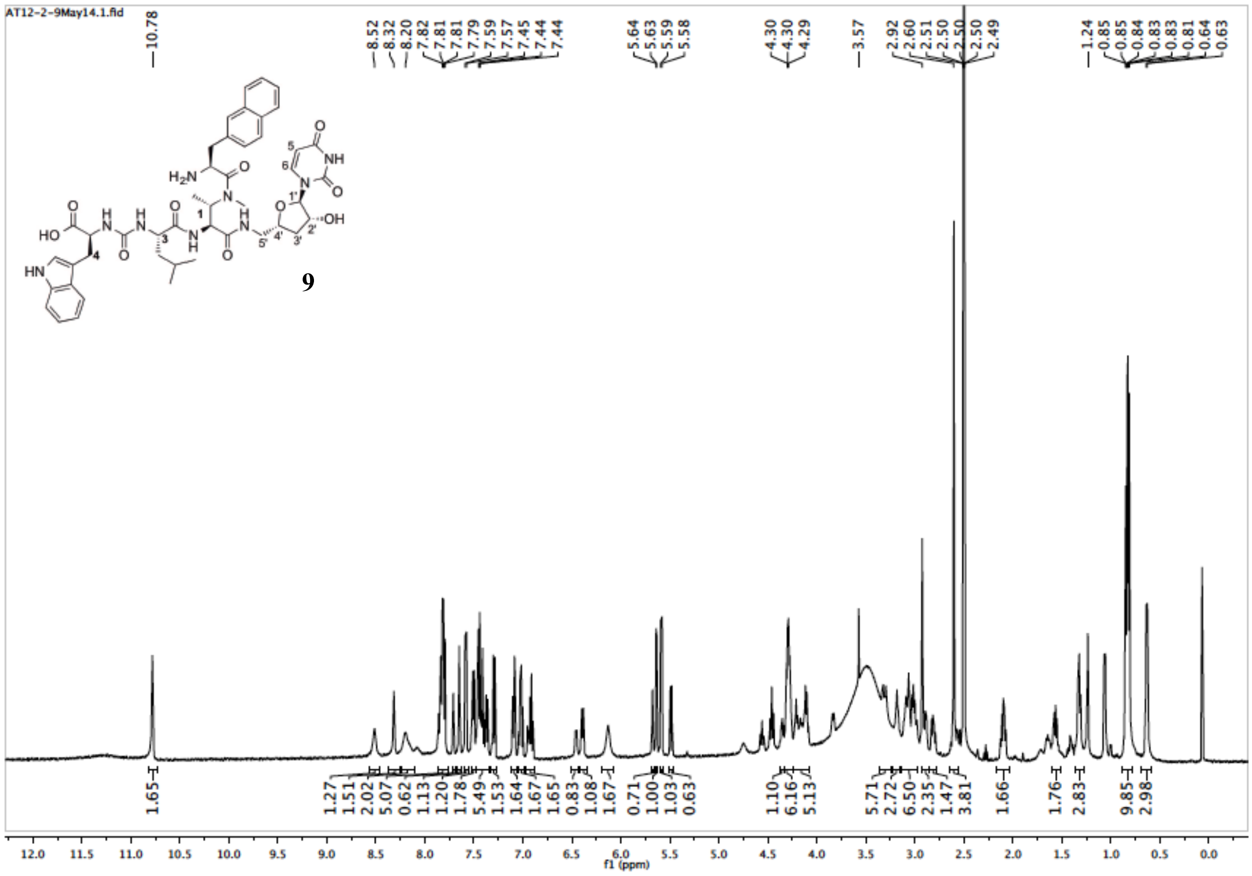

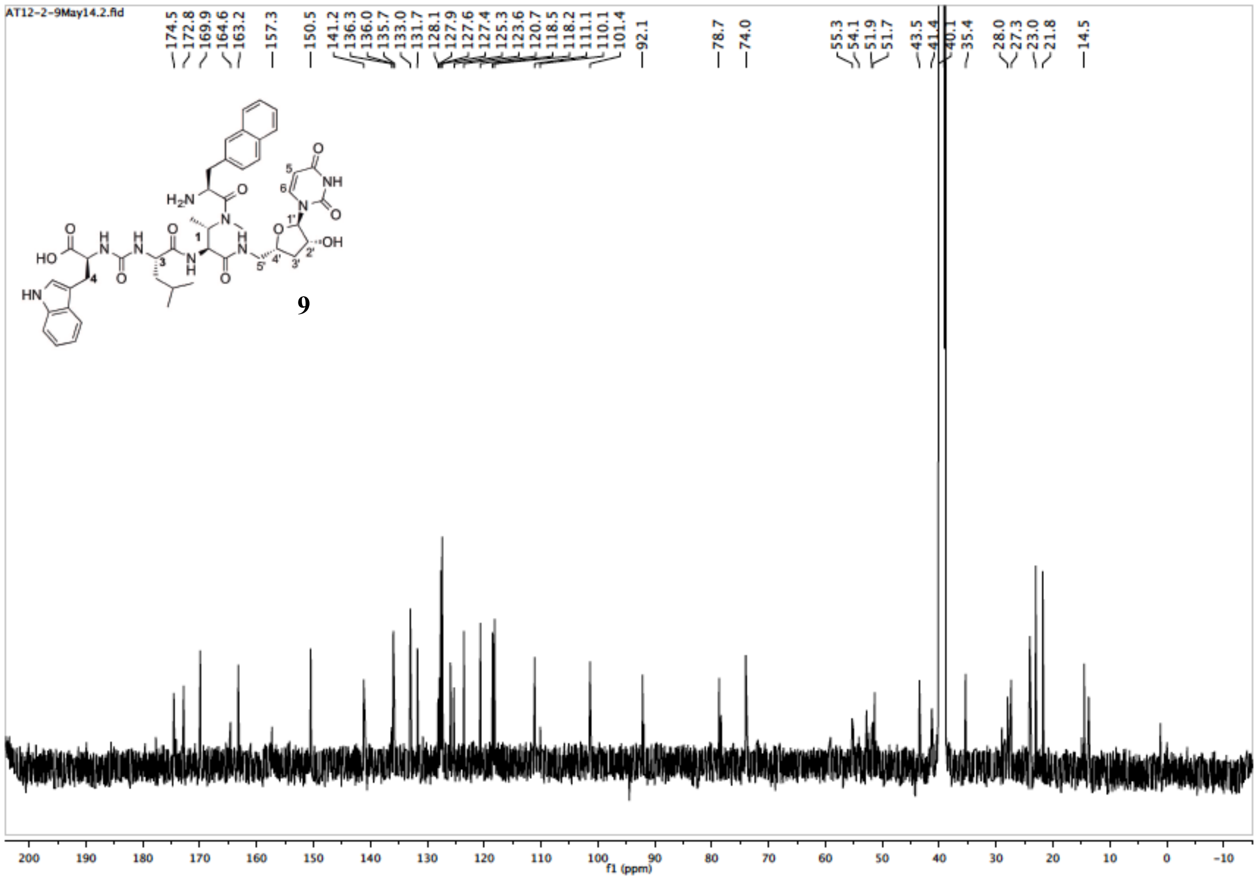

Supplementary Figure 101. ^{1}H (top) and $^{13}C\{^{1}H\}$ (bottom) NMR spectra of compound **9**.

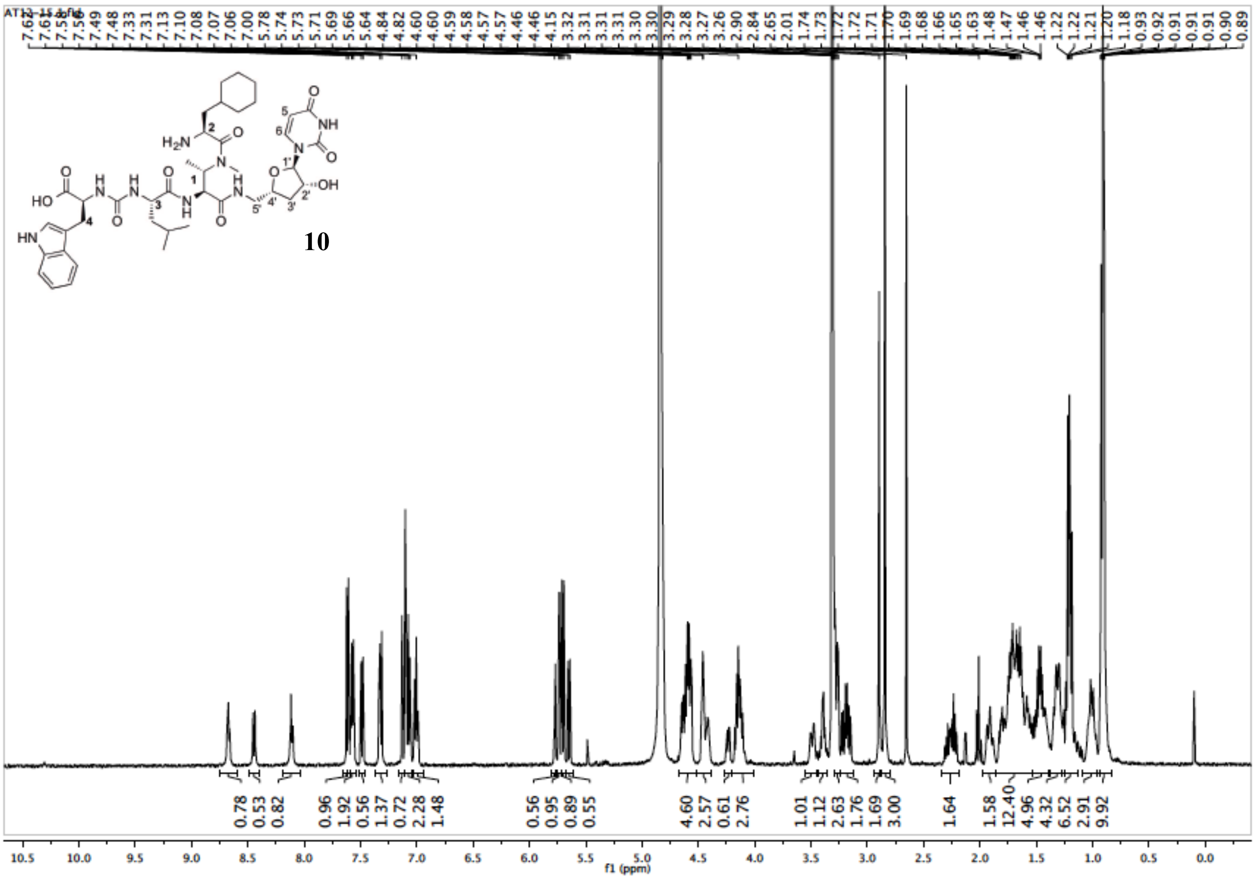

Supplementary Figure 102. ^{1}H NMR spectrum of compound **10**.

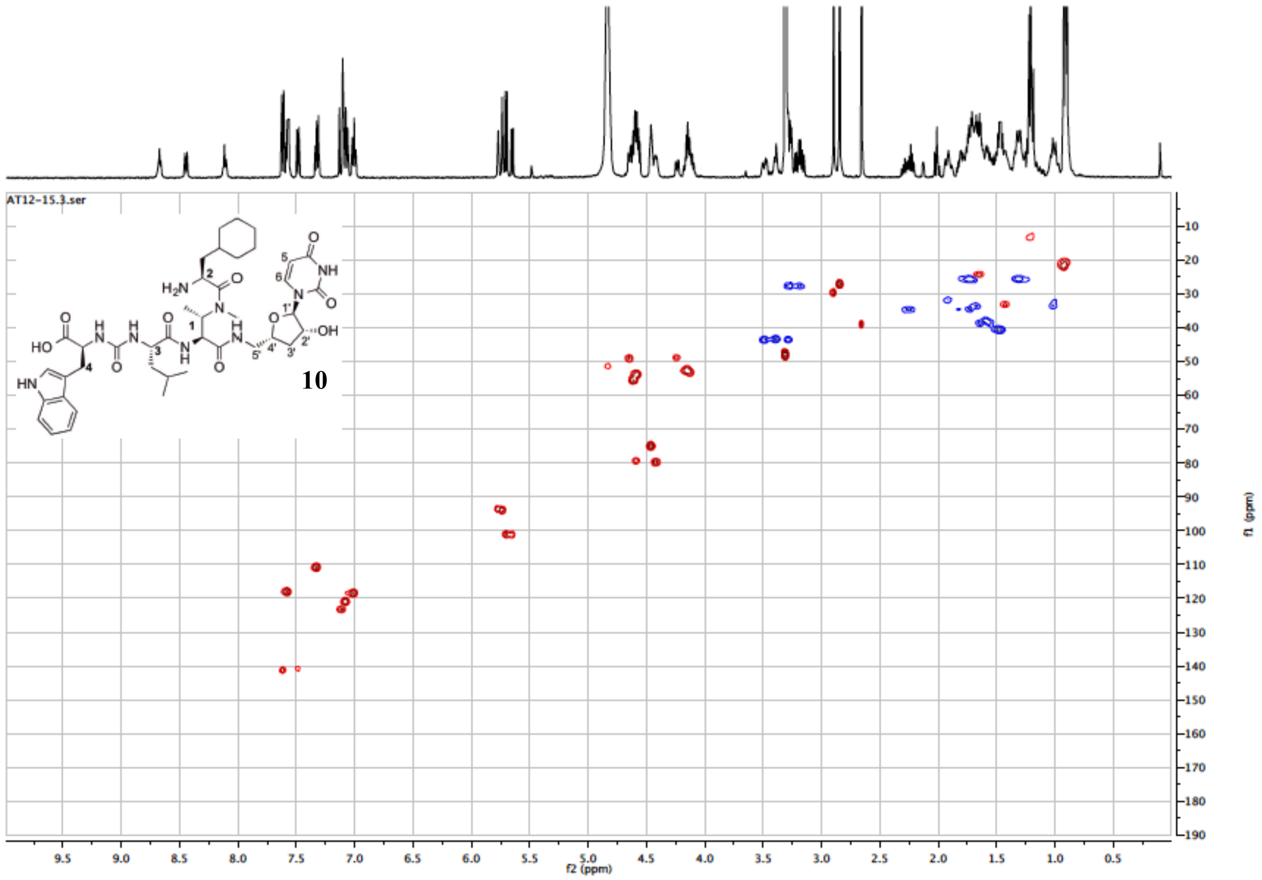

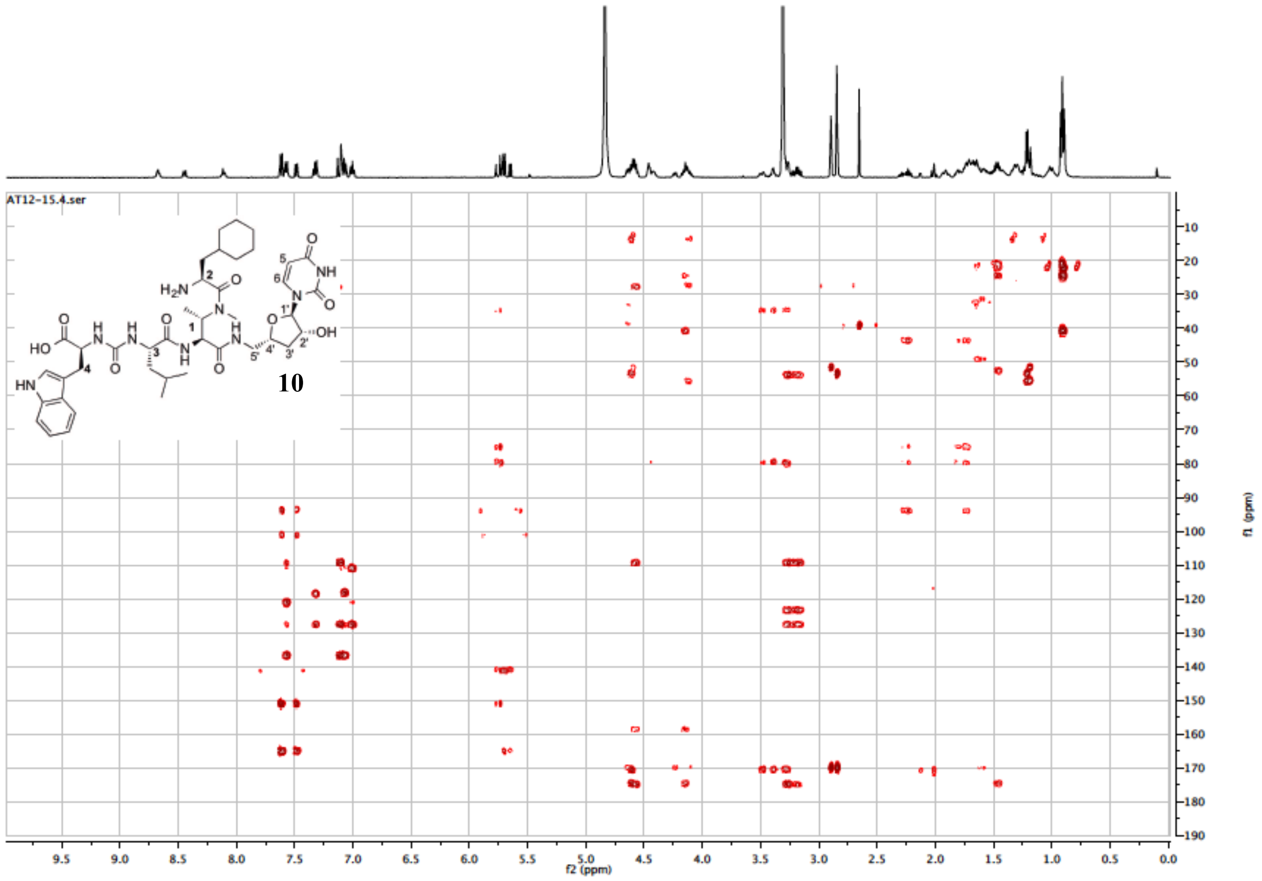

Supplementary Figure 103. HSQC (top) and HMBC (bottom) NMR spectra of compound **10**.

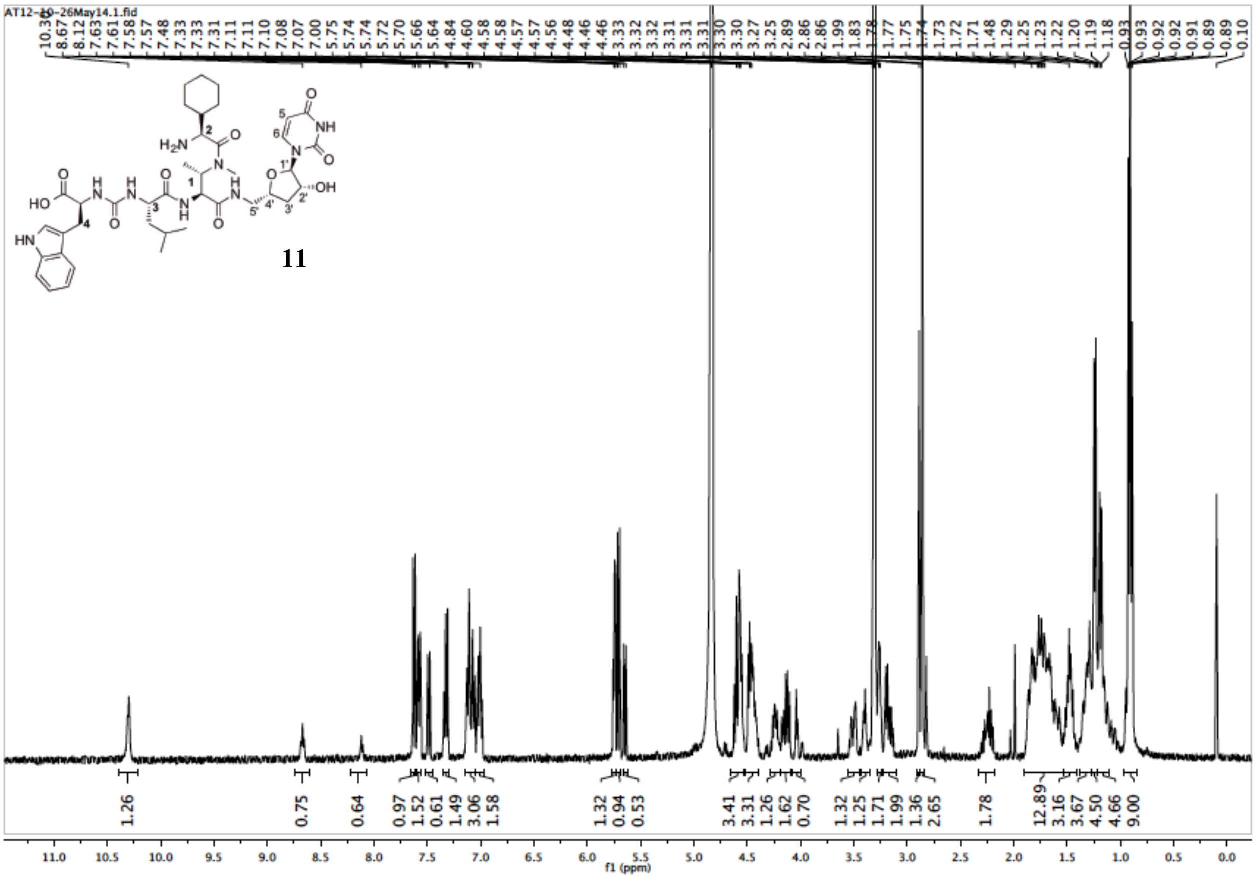

Supplementary Figure 104. ^{1}H NMR spectrum of compound **11**.

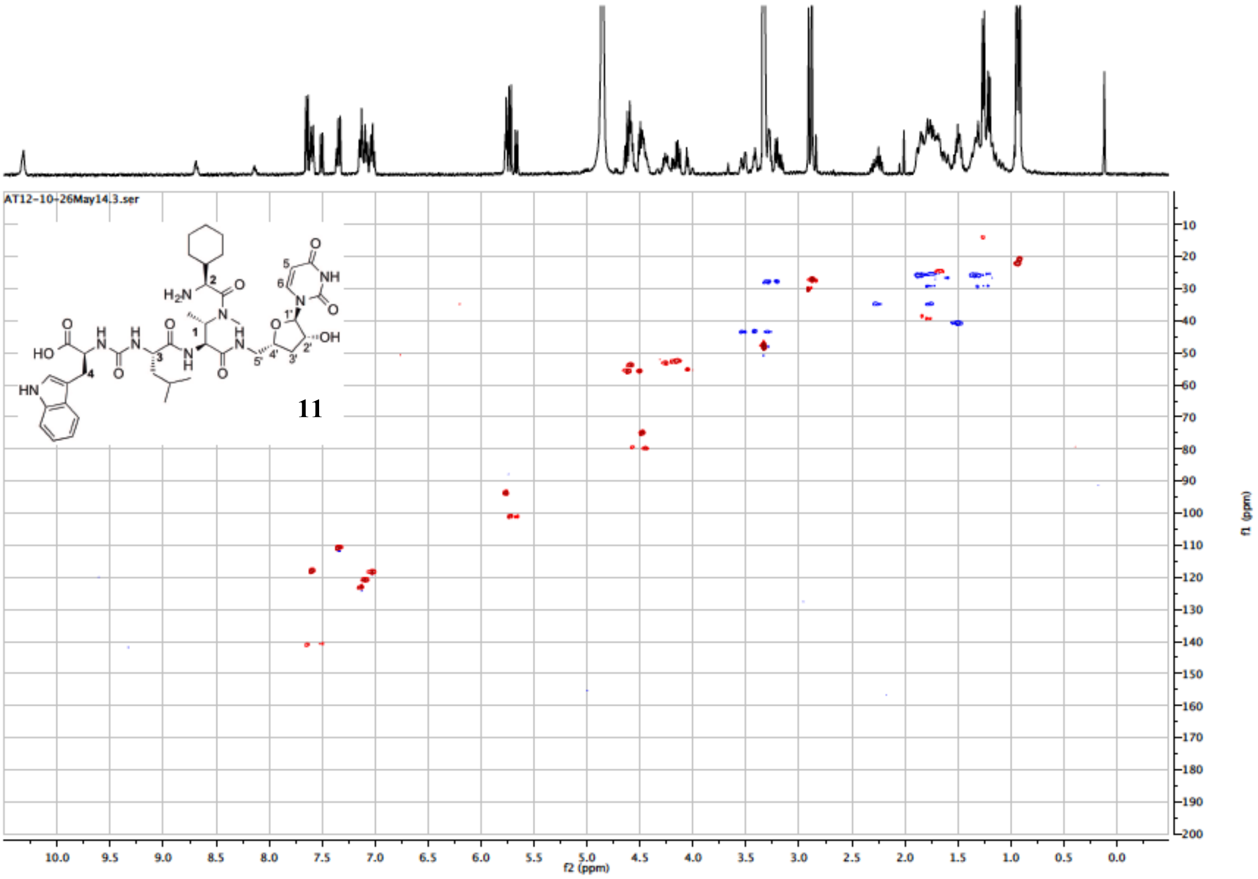

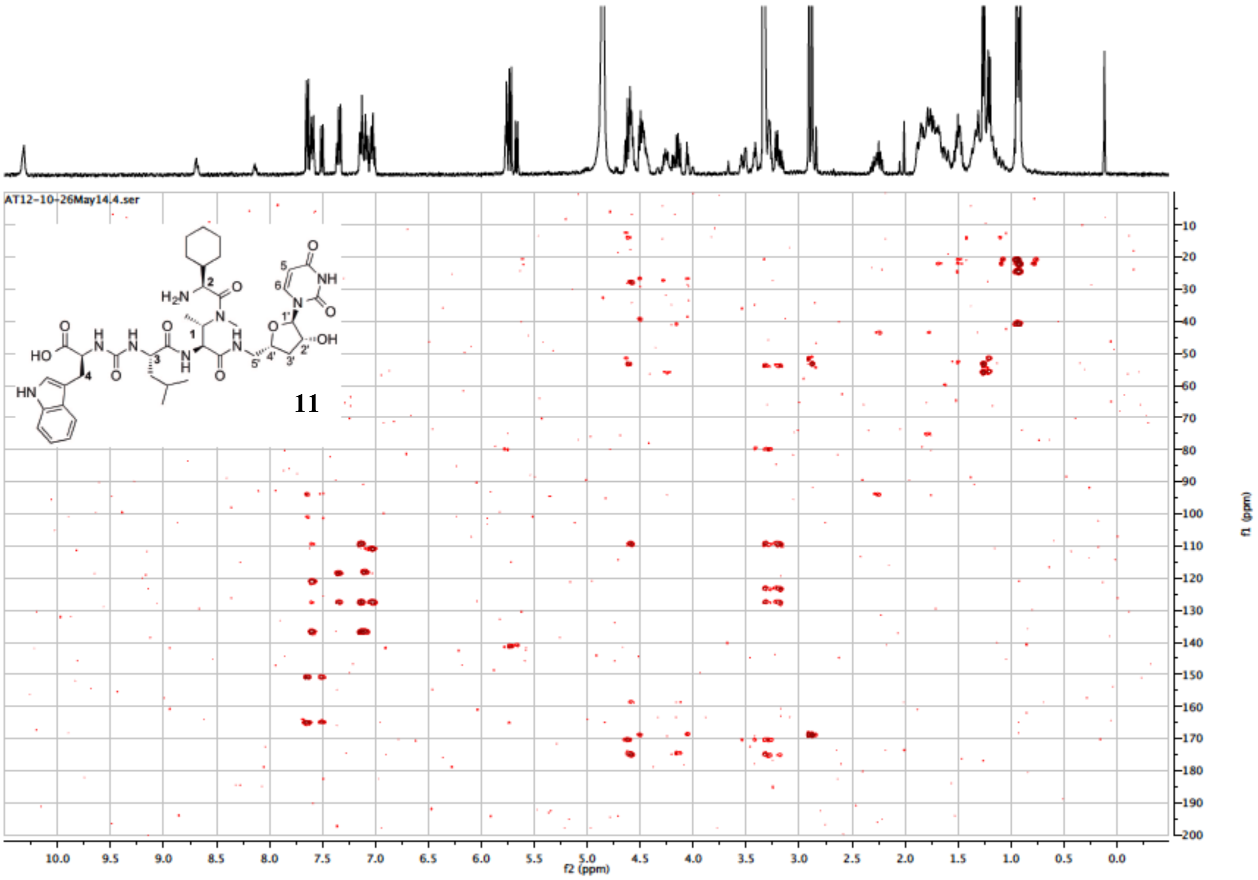

Supplementary Figure 105. HSQC (top) and HMBC (bottom) NMR spectra of compound **11**.

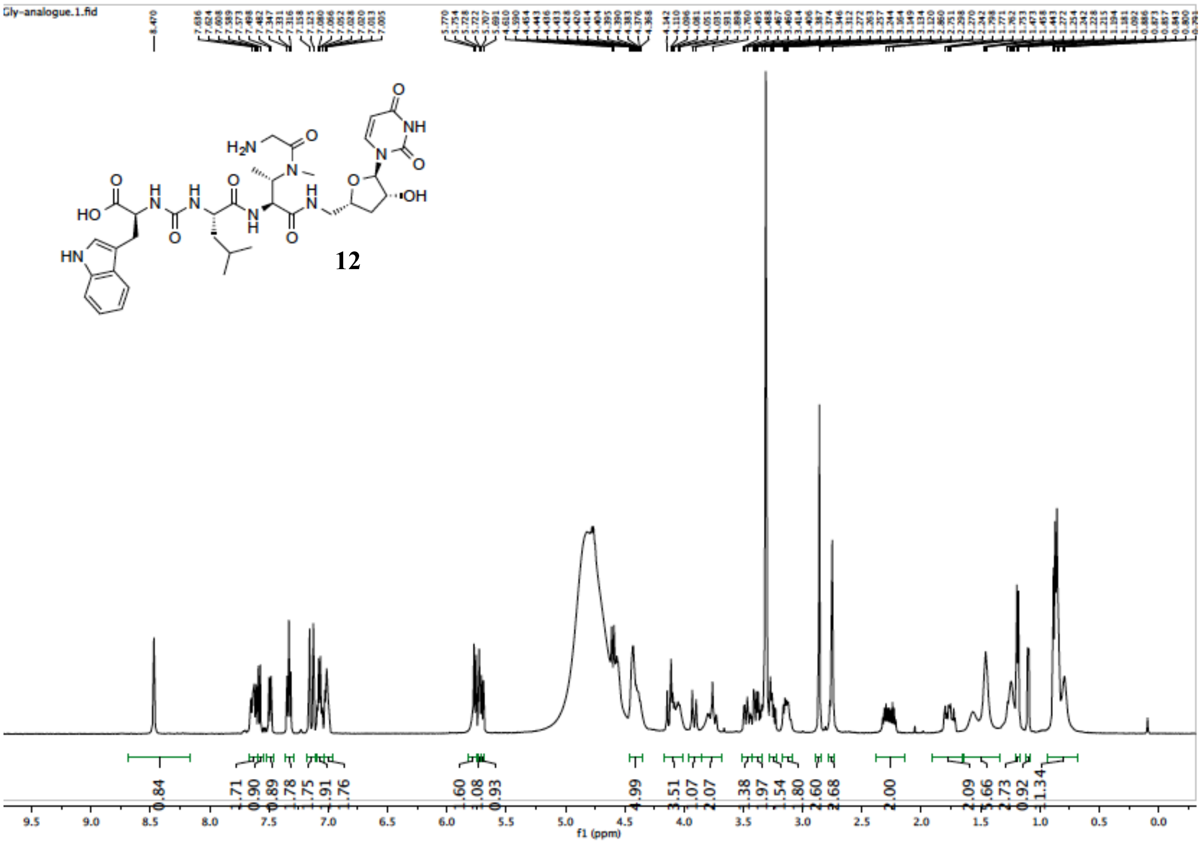

Supplementary Figure 106. ^{1}H NMR spectrum of compound **12**.

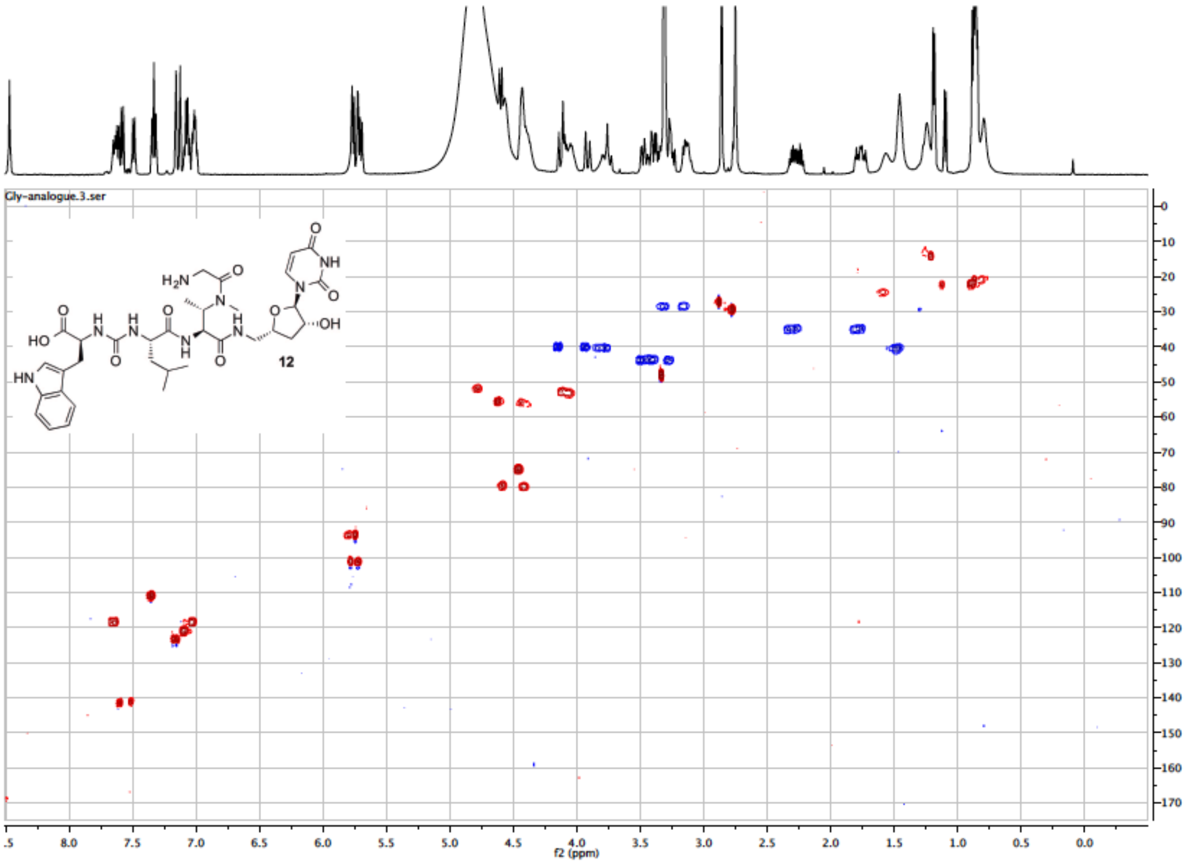

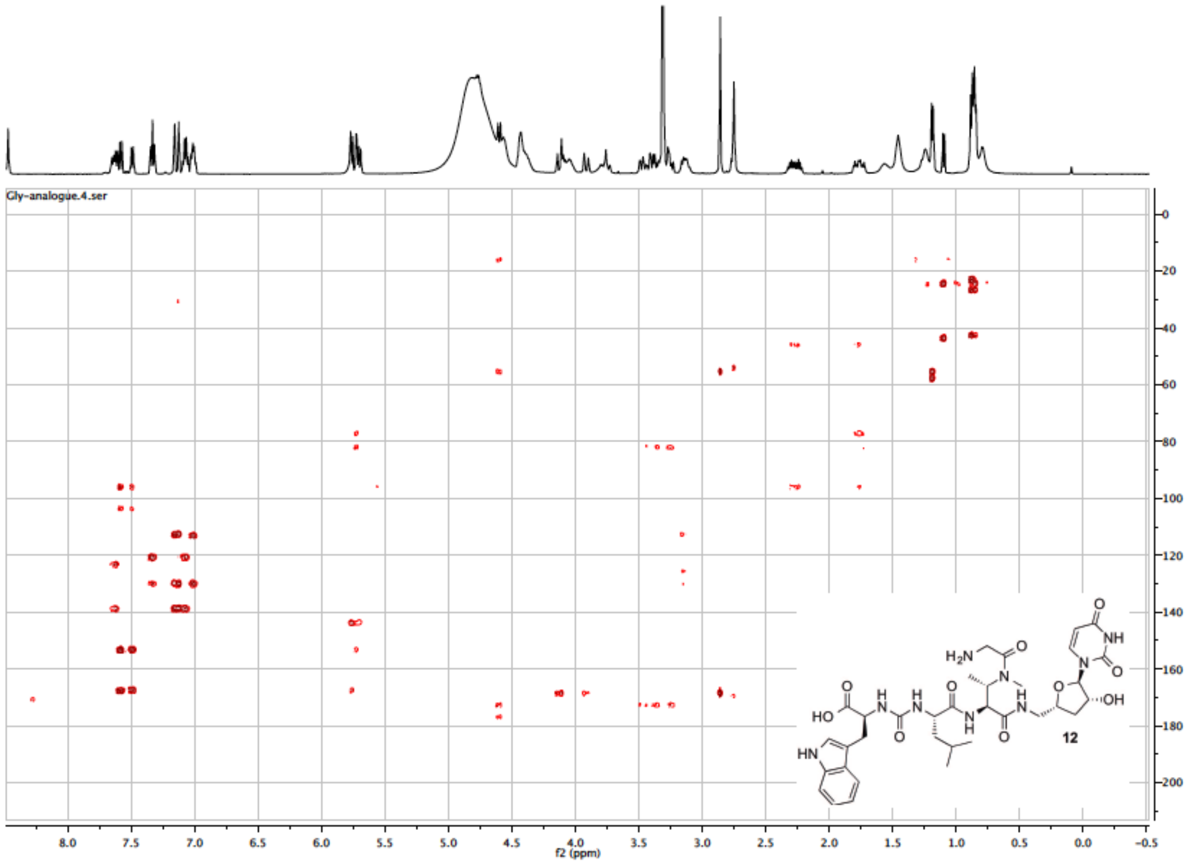

Supplementary Figure 107. HSQC (top) and HMBC (bottom) NMR spectra of compound **12**.

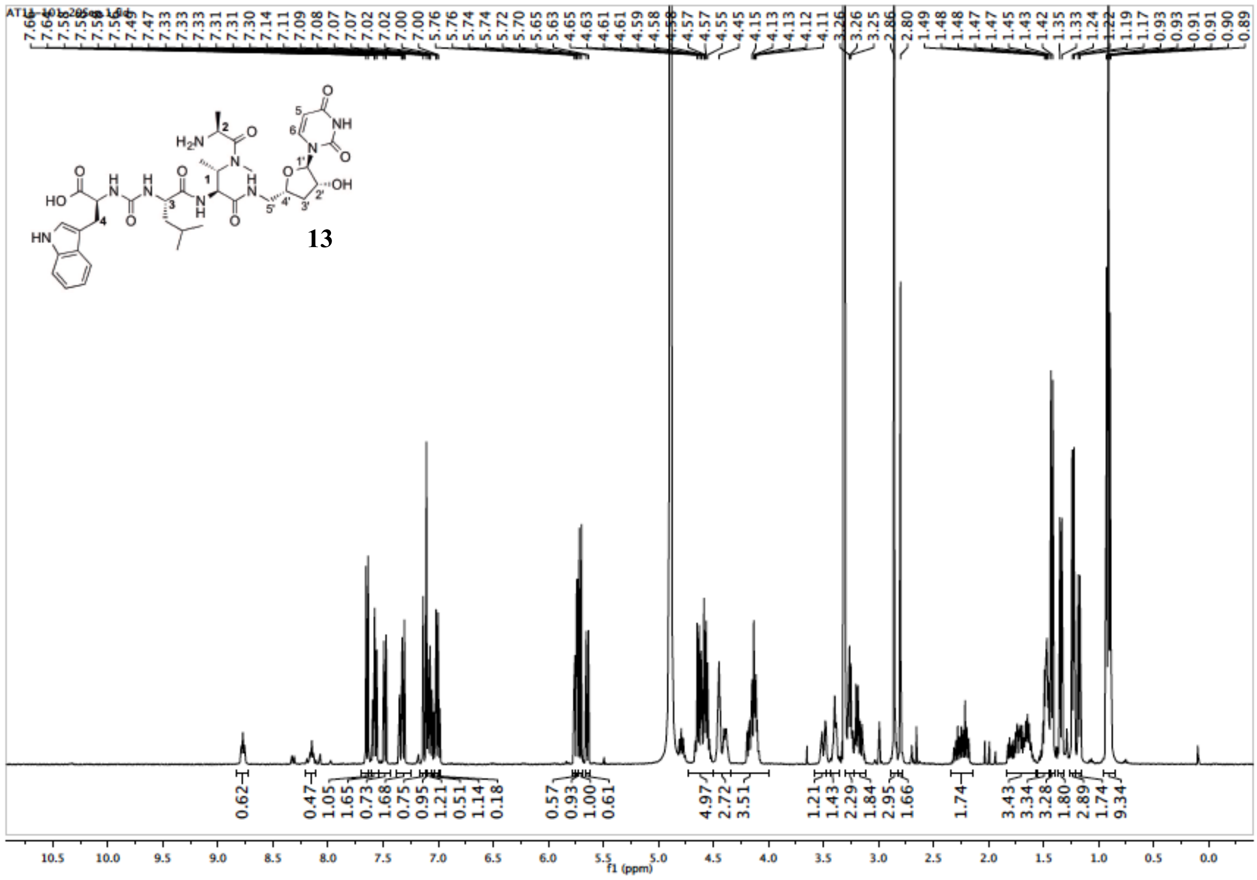

Supplementary Figure 108. ^{1}H NMR spectrum of compound **13**.

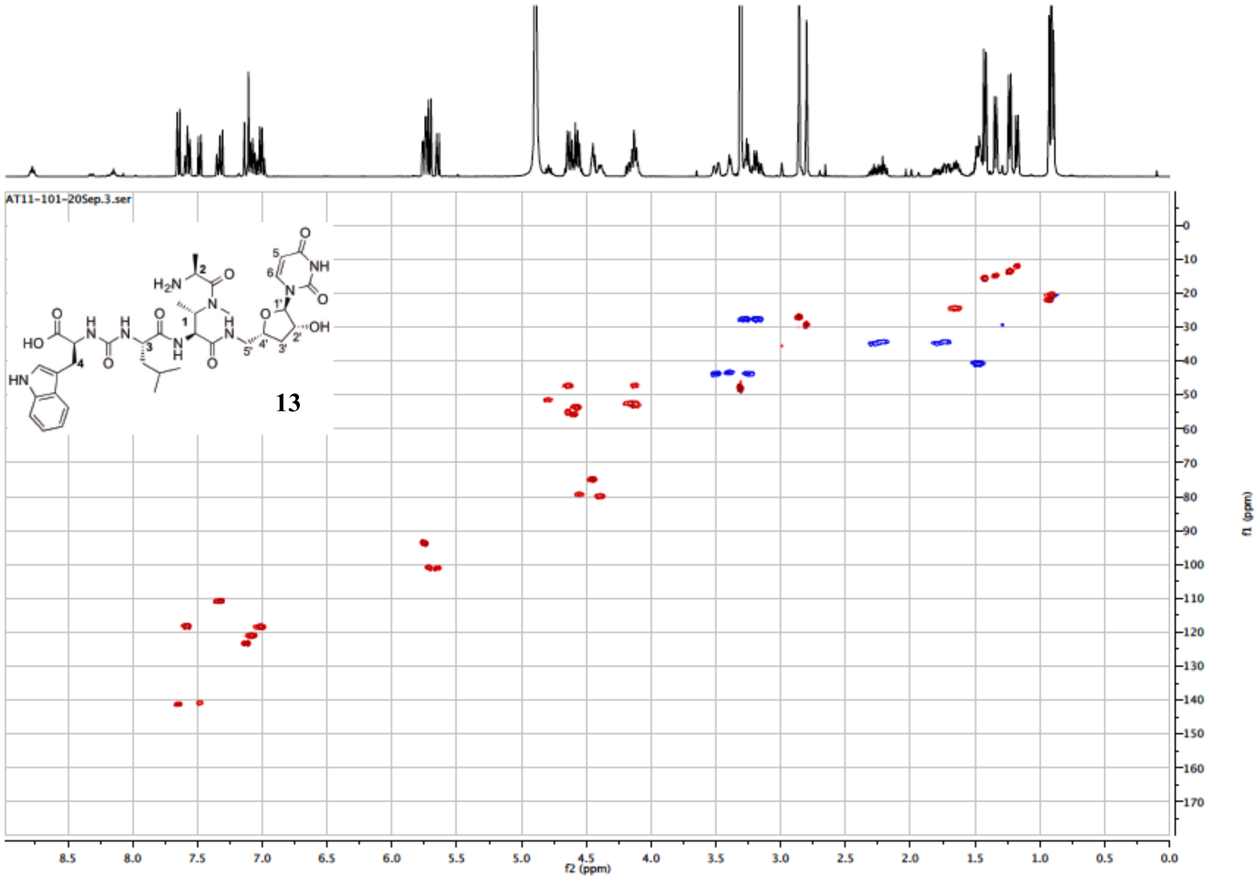

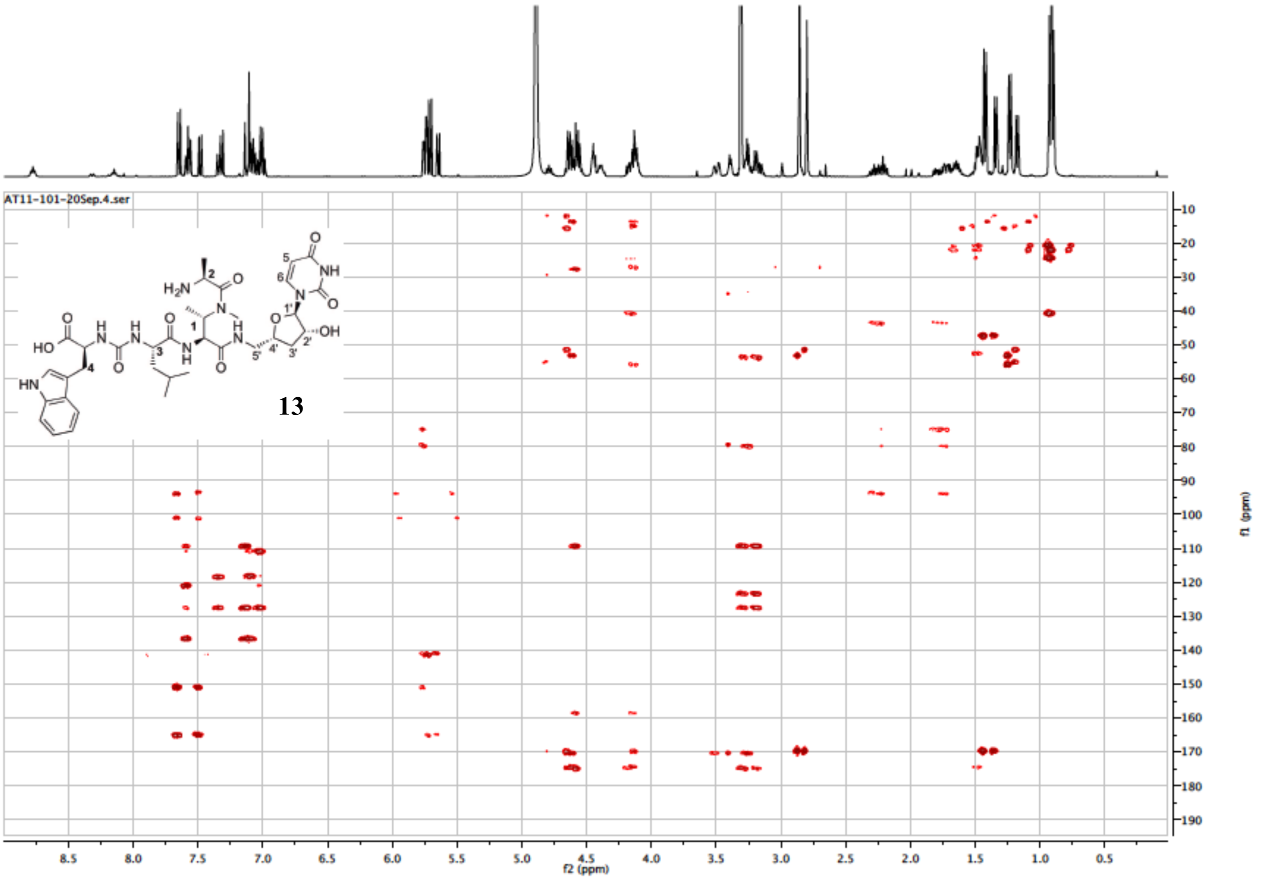

Supplementary Figure 109. HSQC (top) and HMBC (bottom) NMR spectra of compound **13**.

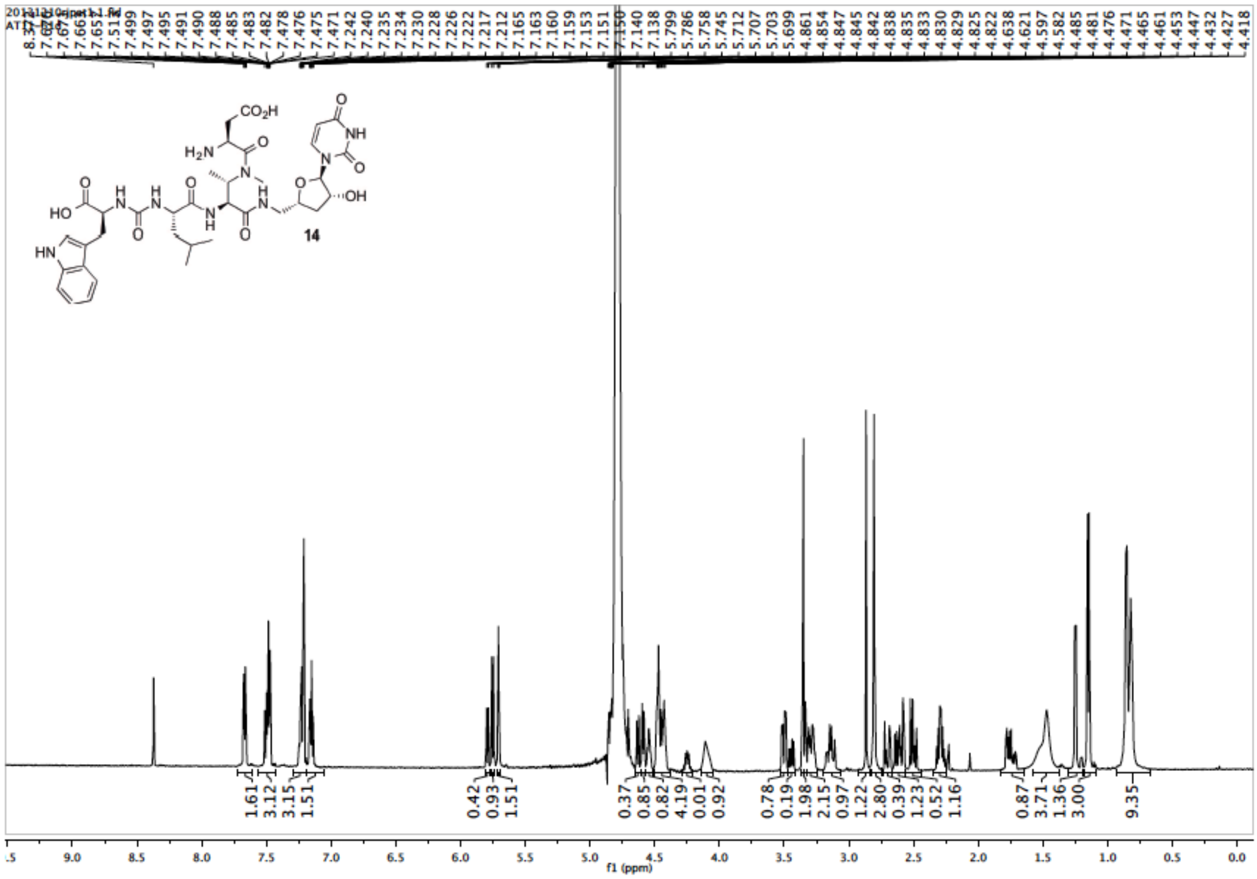

Supplementary Figure 110. ^{1}H NMR spectrum of compound **14**.

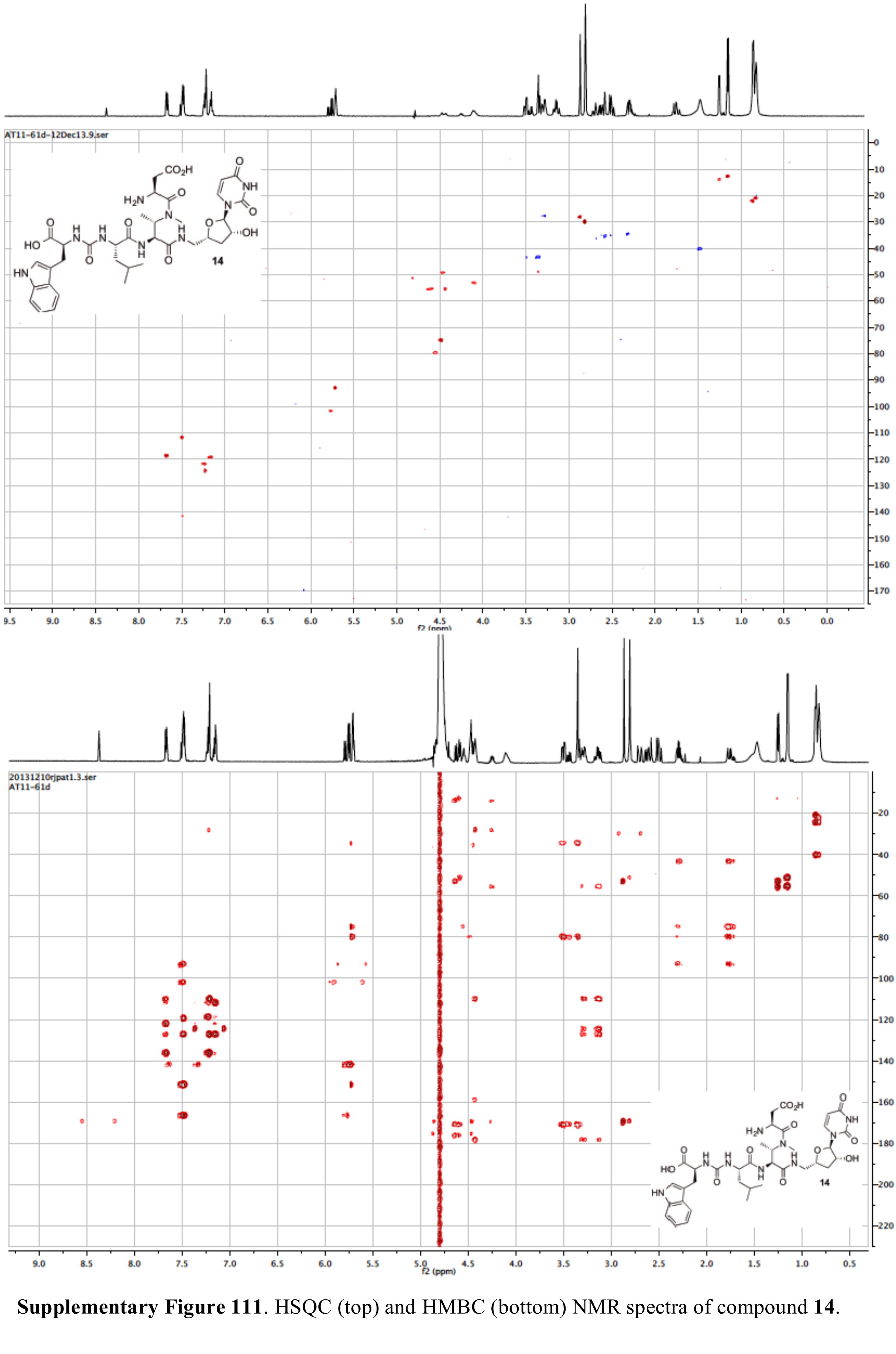

Supplementary Figure 111. HSQC (top) and HMBC (bottom) NMR spectra of compound **14**.

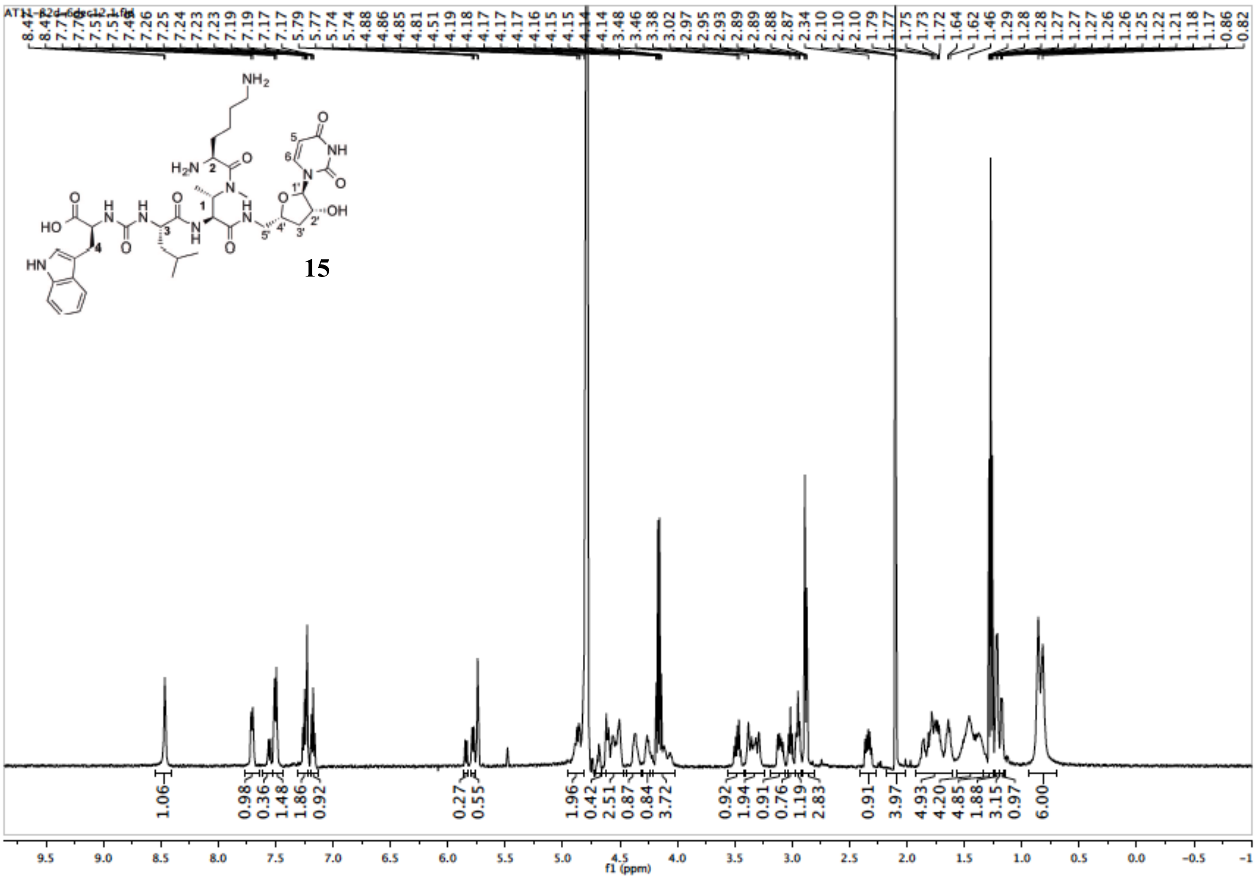

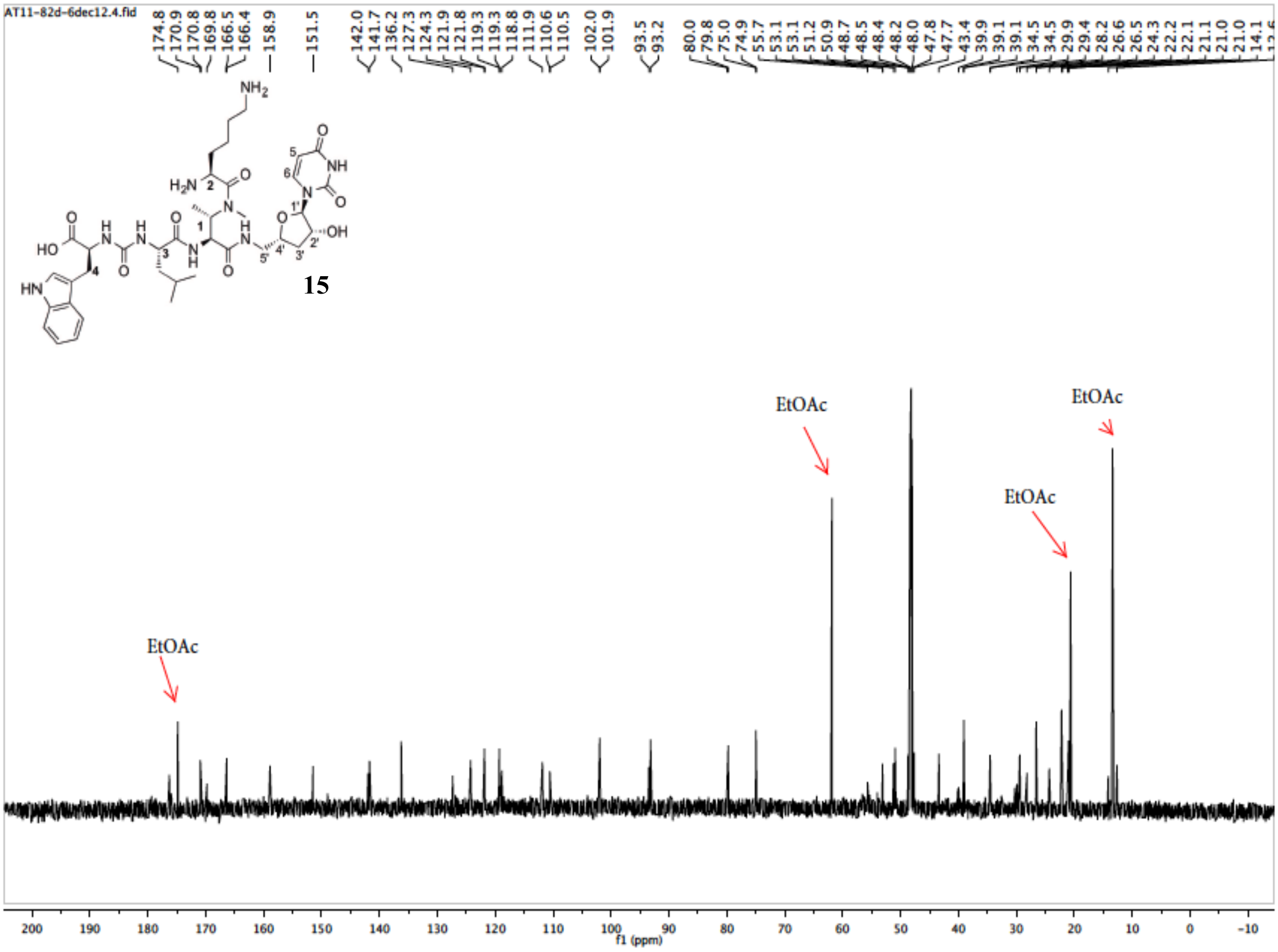

Supplementary Figure 112. ^{1}H (top) and ^{13}C{^{1}H} (bottom) NMR spectra of compound **15**.

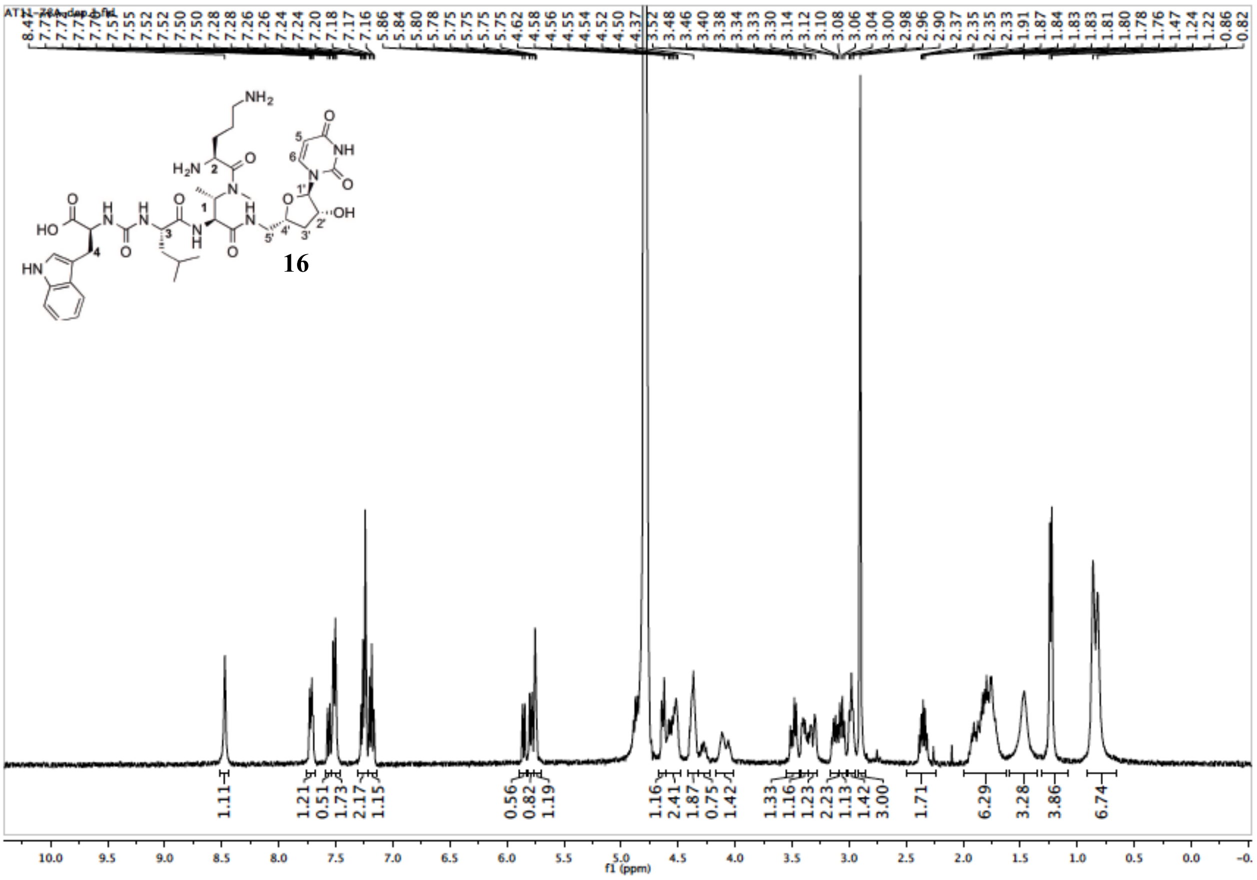

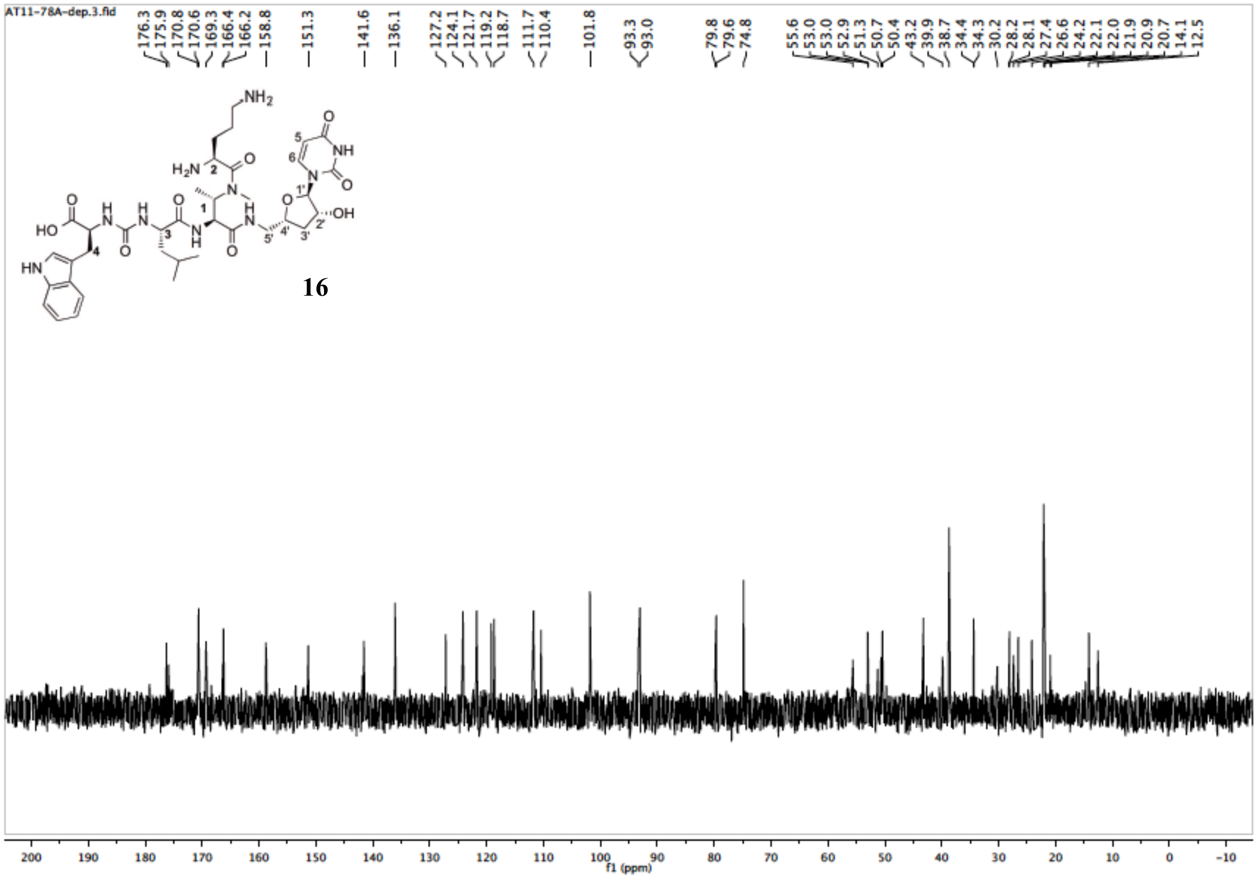

Supplementary Figure 113. ^{1}H (top) and $^{13}C\{^{1}H\}$ (bottom) NMR spectra of compound **16**.

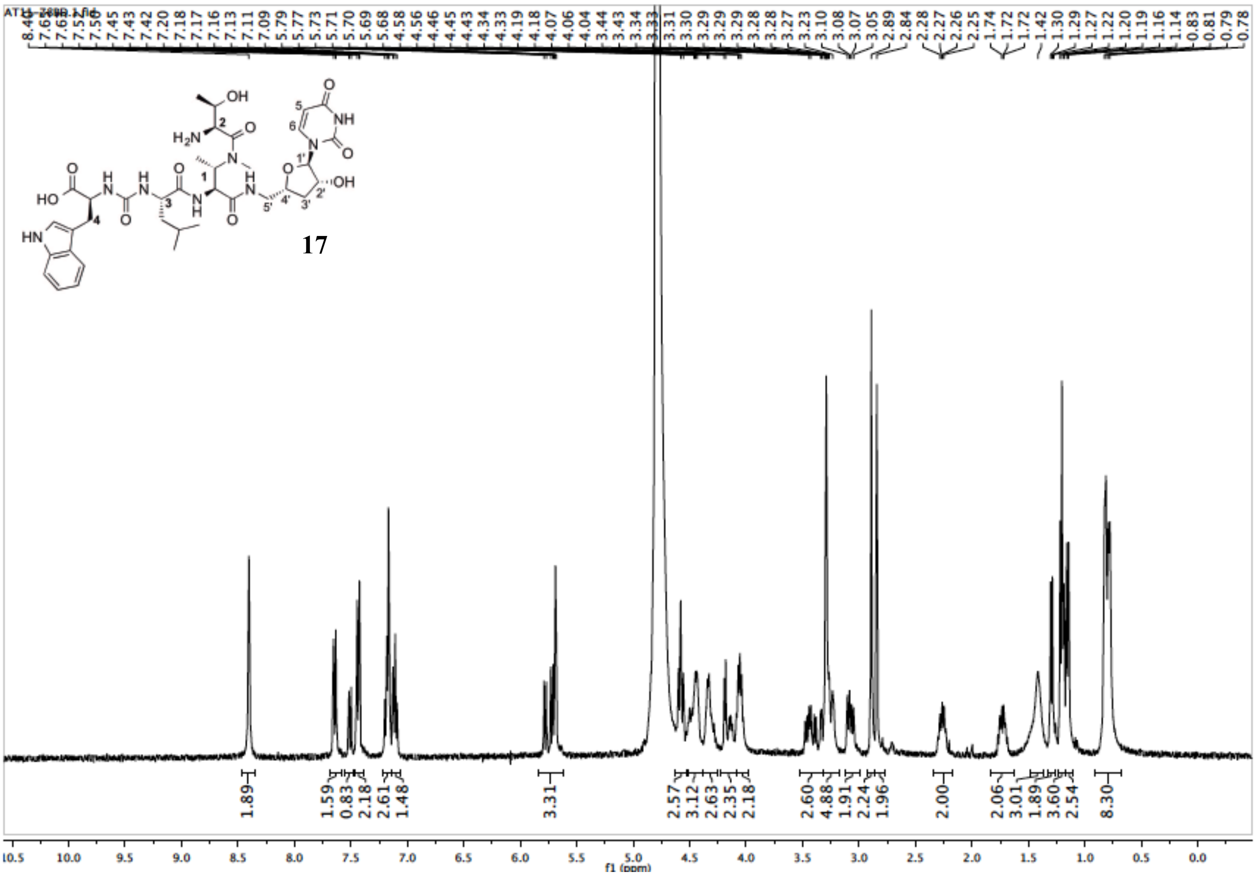

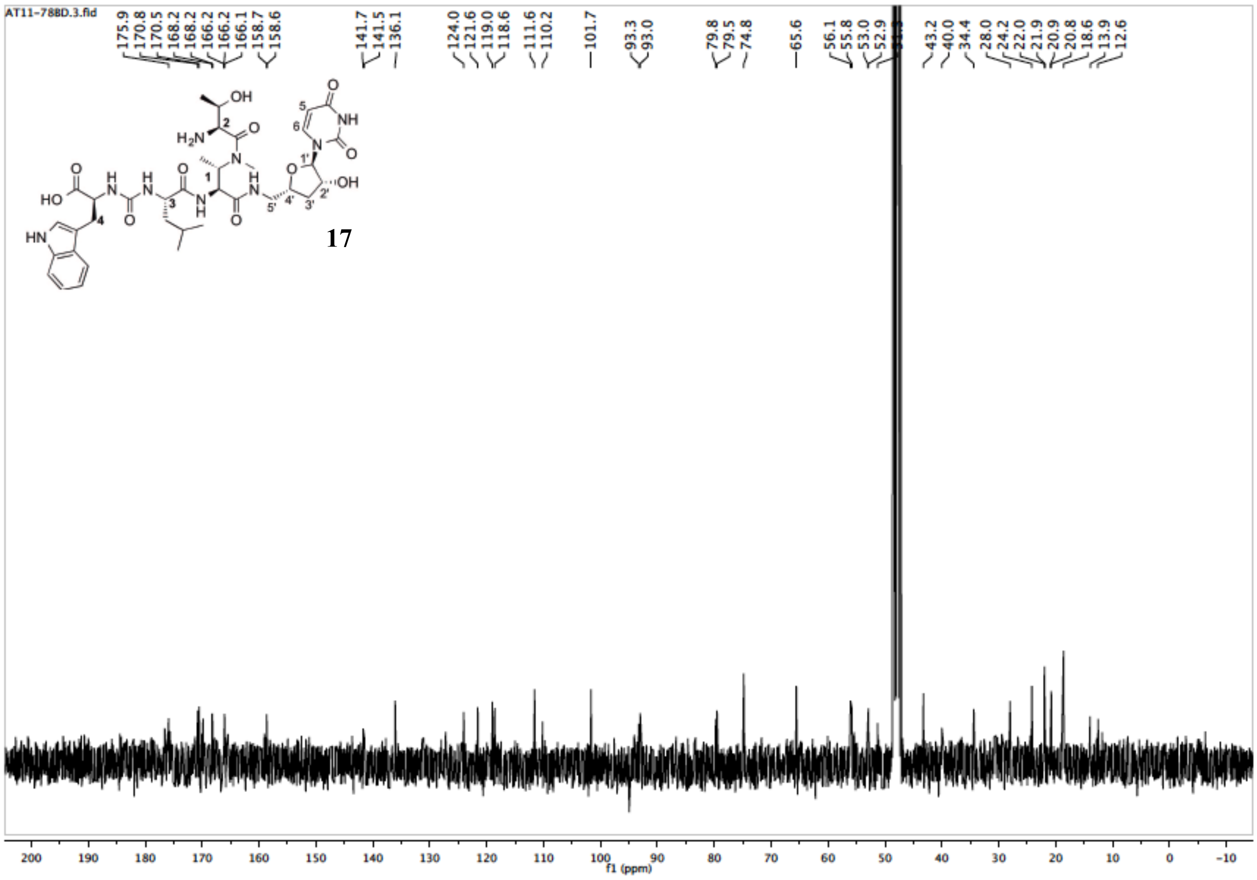

Supplementary Figure 114. ^{1}H (top) and ^{13}C{^{1}H} (bottom) NMR spectra of compound **17**.

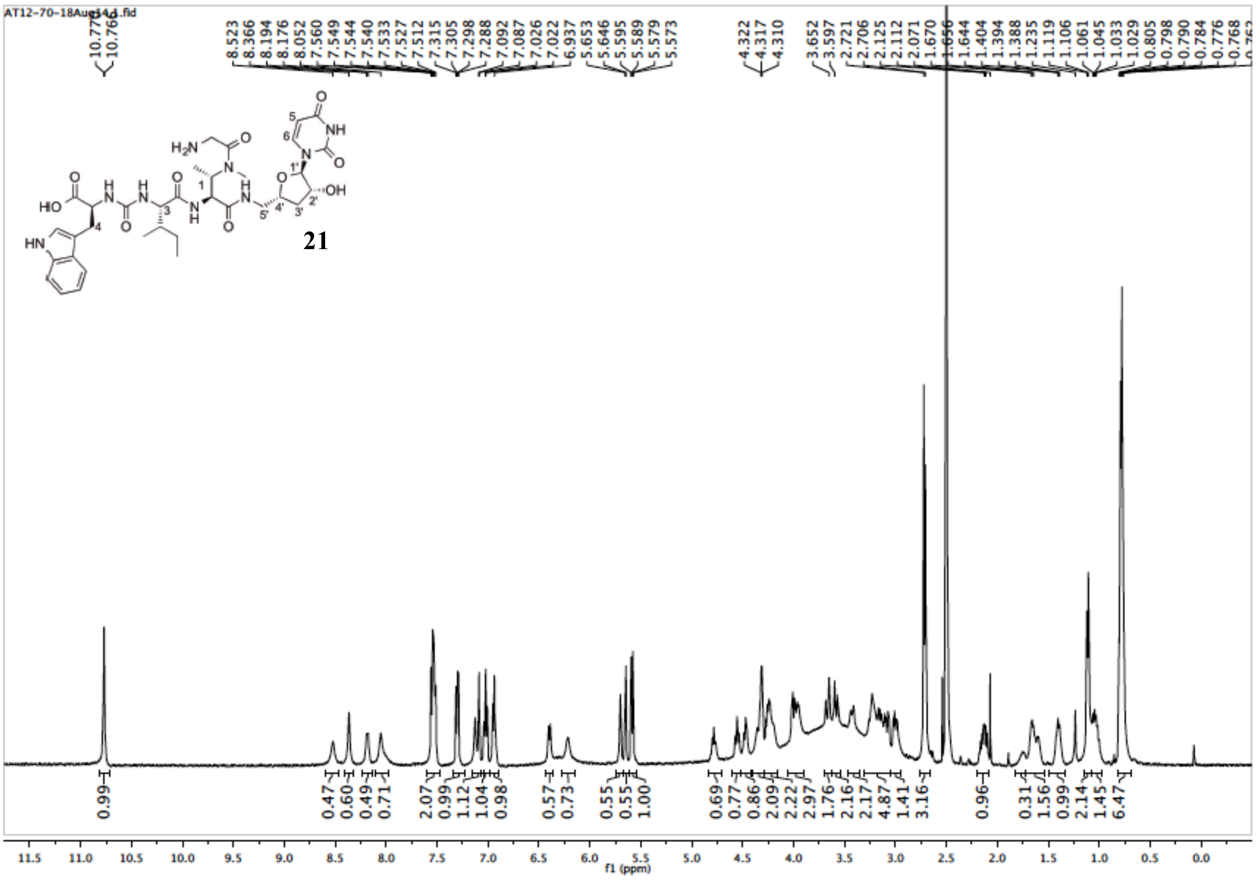

Supplementary Figure 115. ^{1}H NMR spectrum of compound **21**.

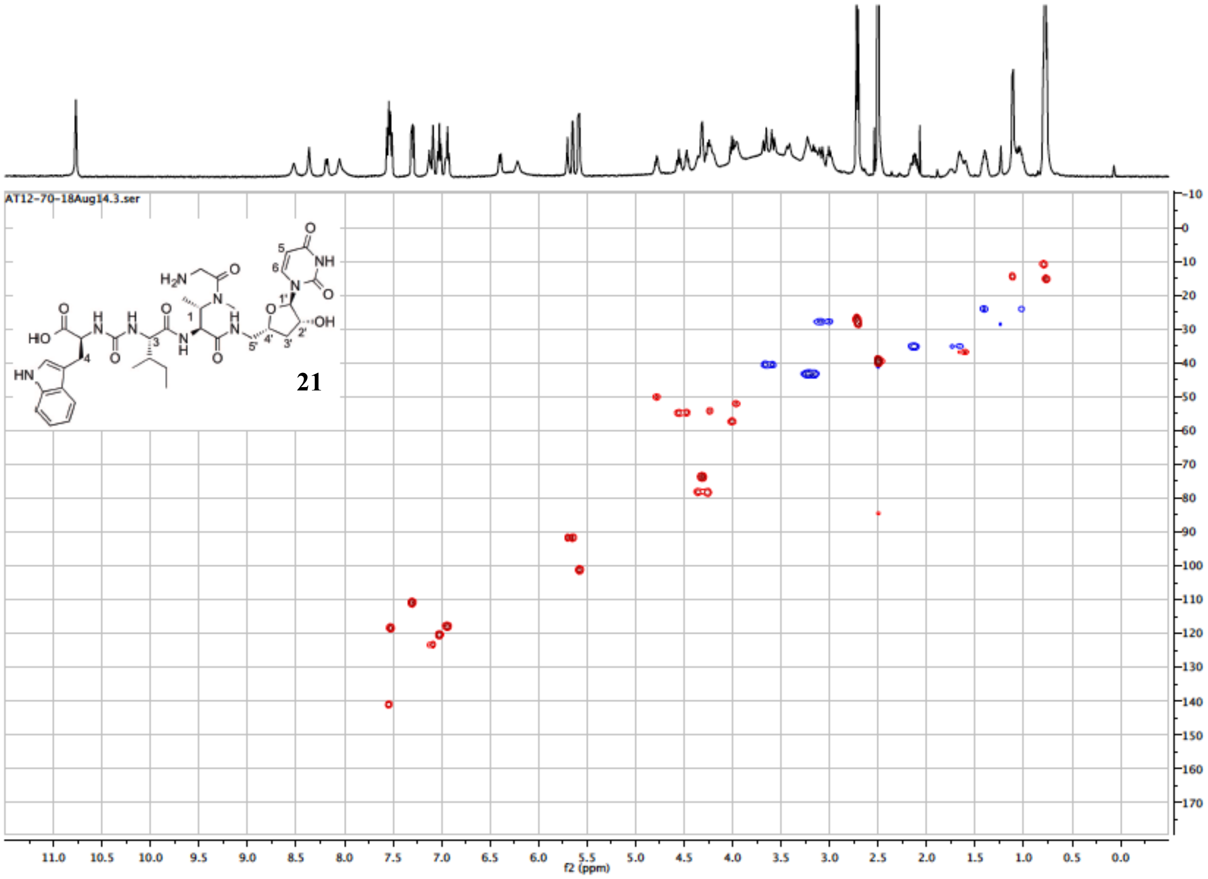

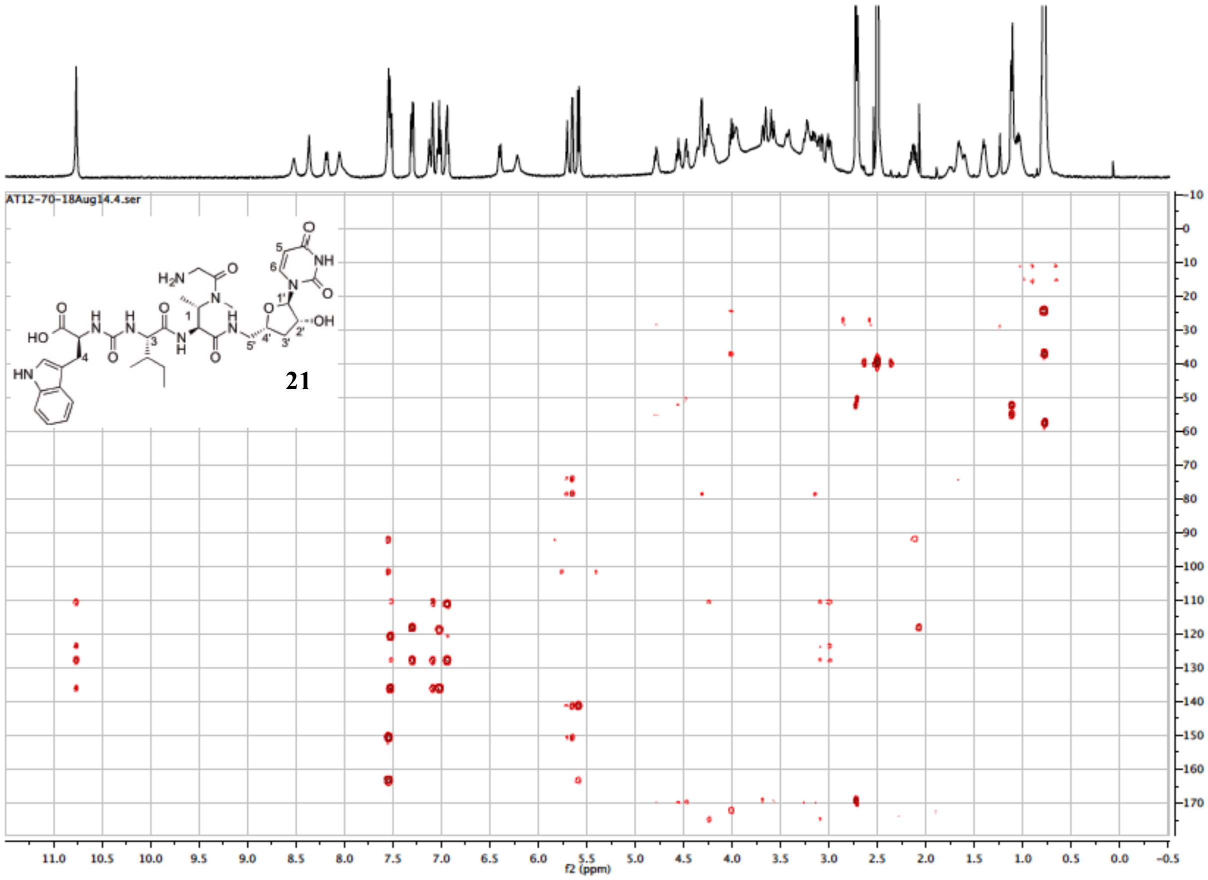

Supplementary Figure 116. HSQC (top) and HMBC (bottom) NMR spectra of compound **21**.

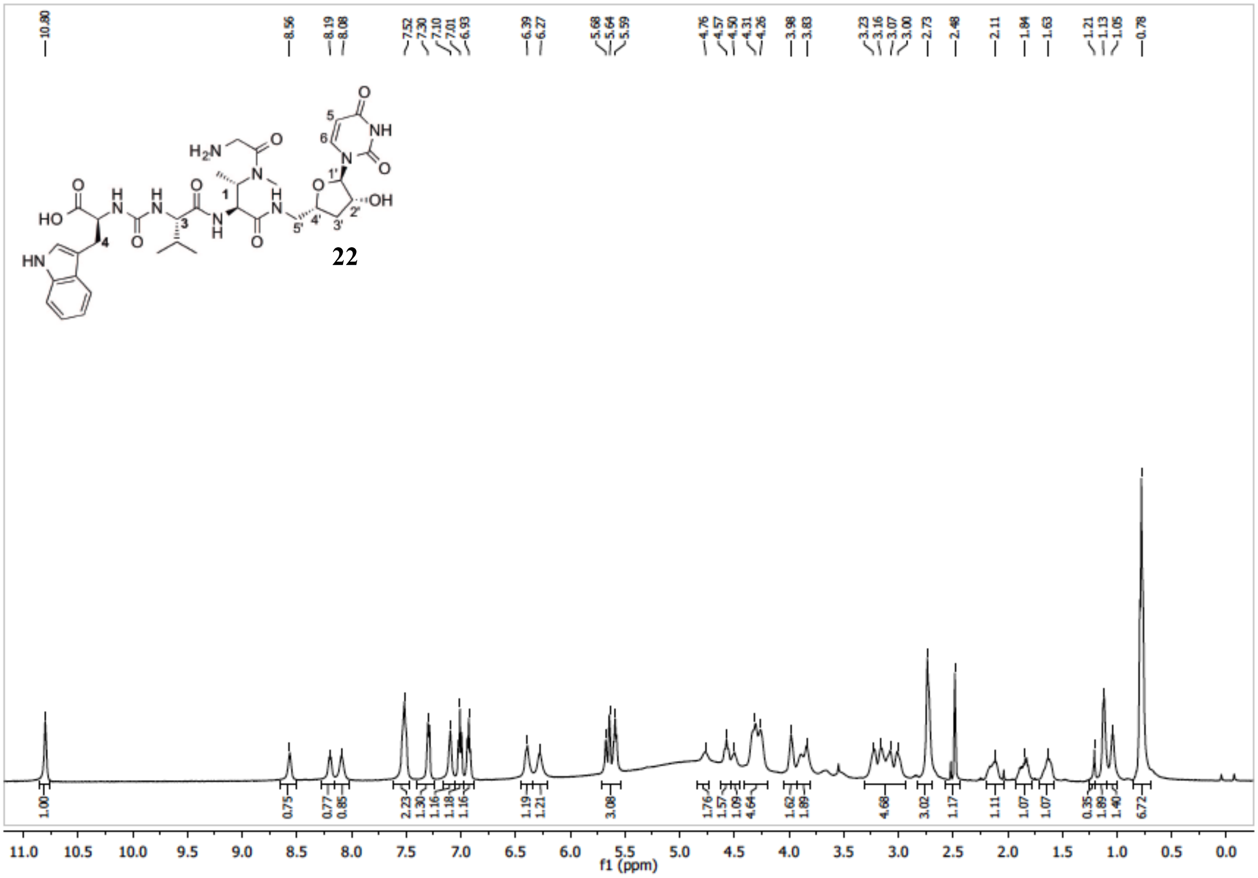

Supplementary Figure 117. ^{1}H NMR spectrum of compound **22**.

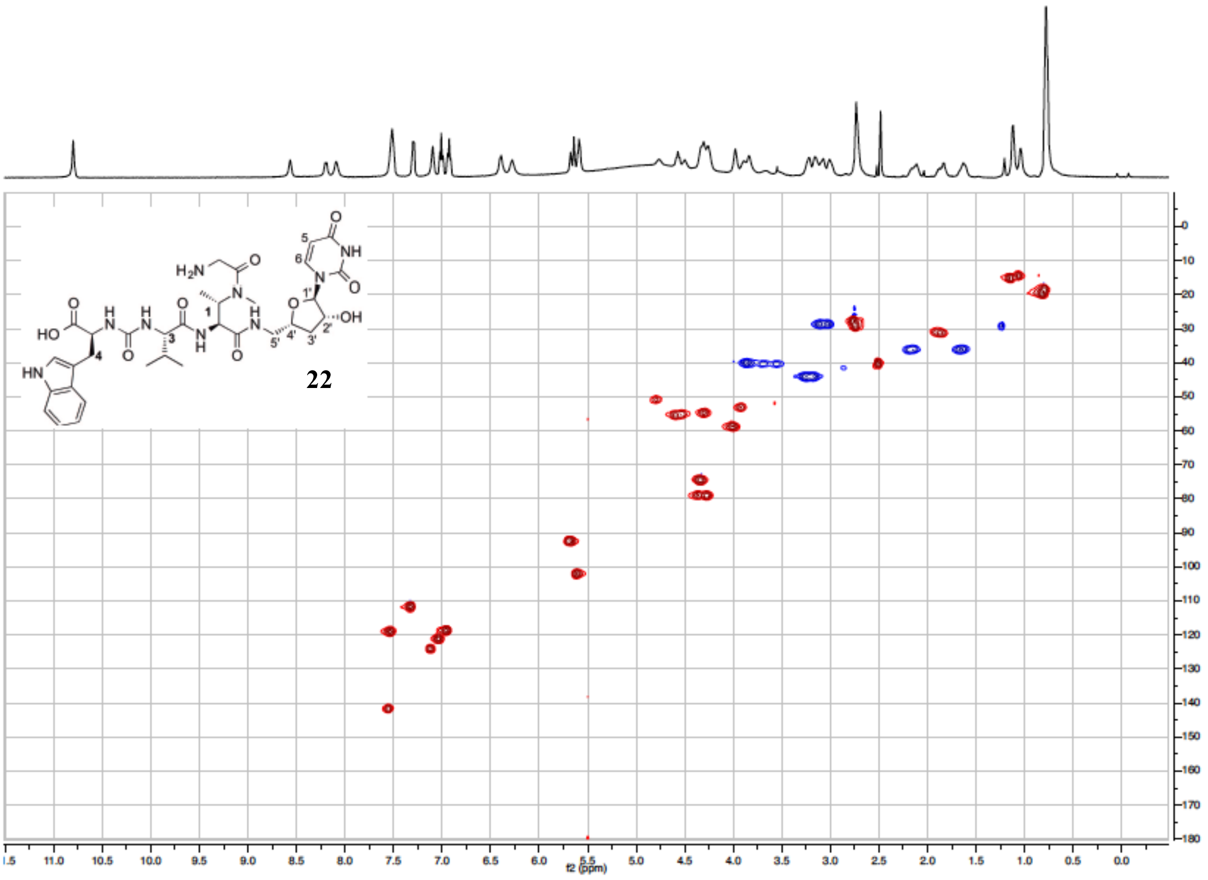

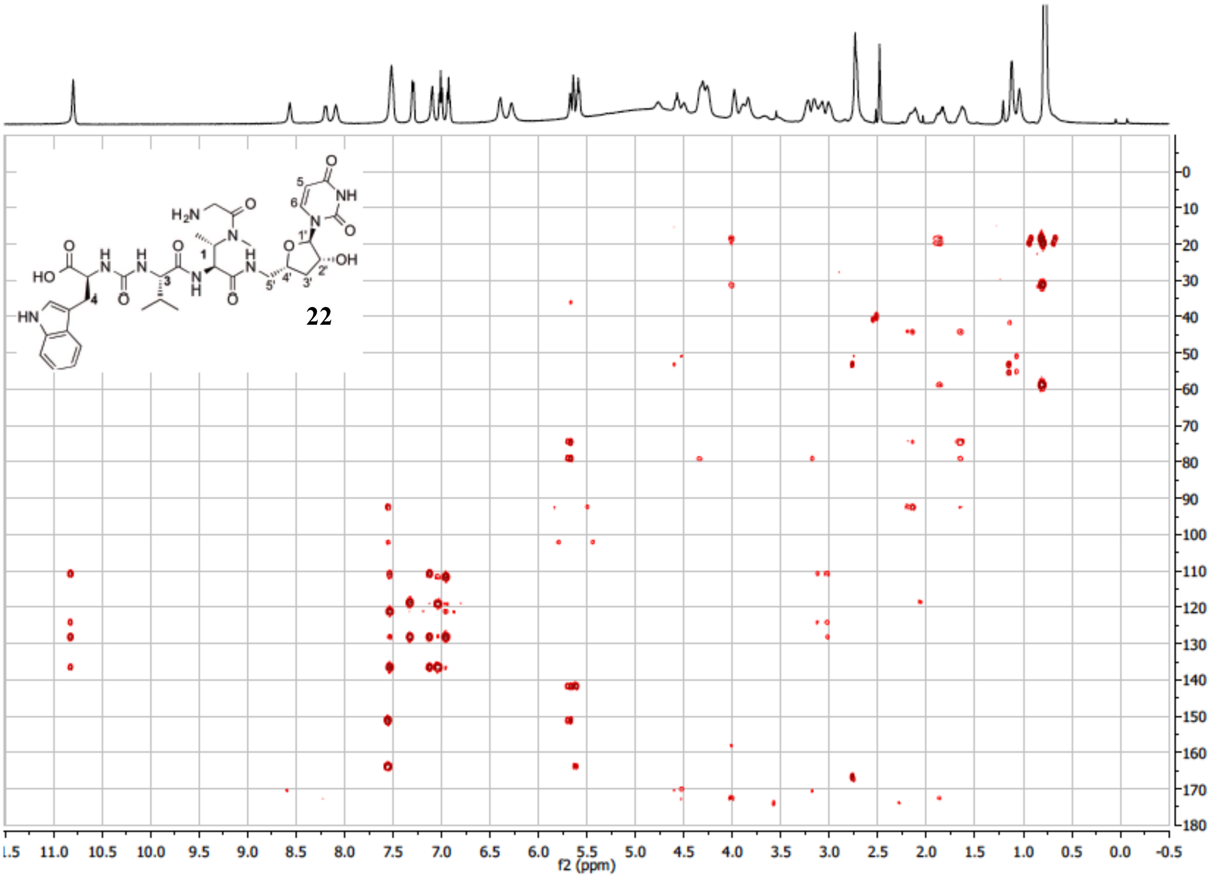

Supplementary Figure 118. HSQC (top) and HMBC (bottom) NMR spectra of compound **22**.

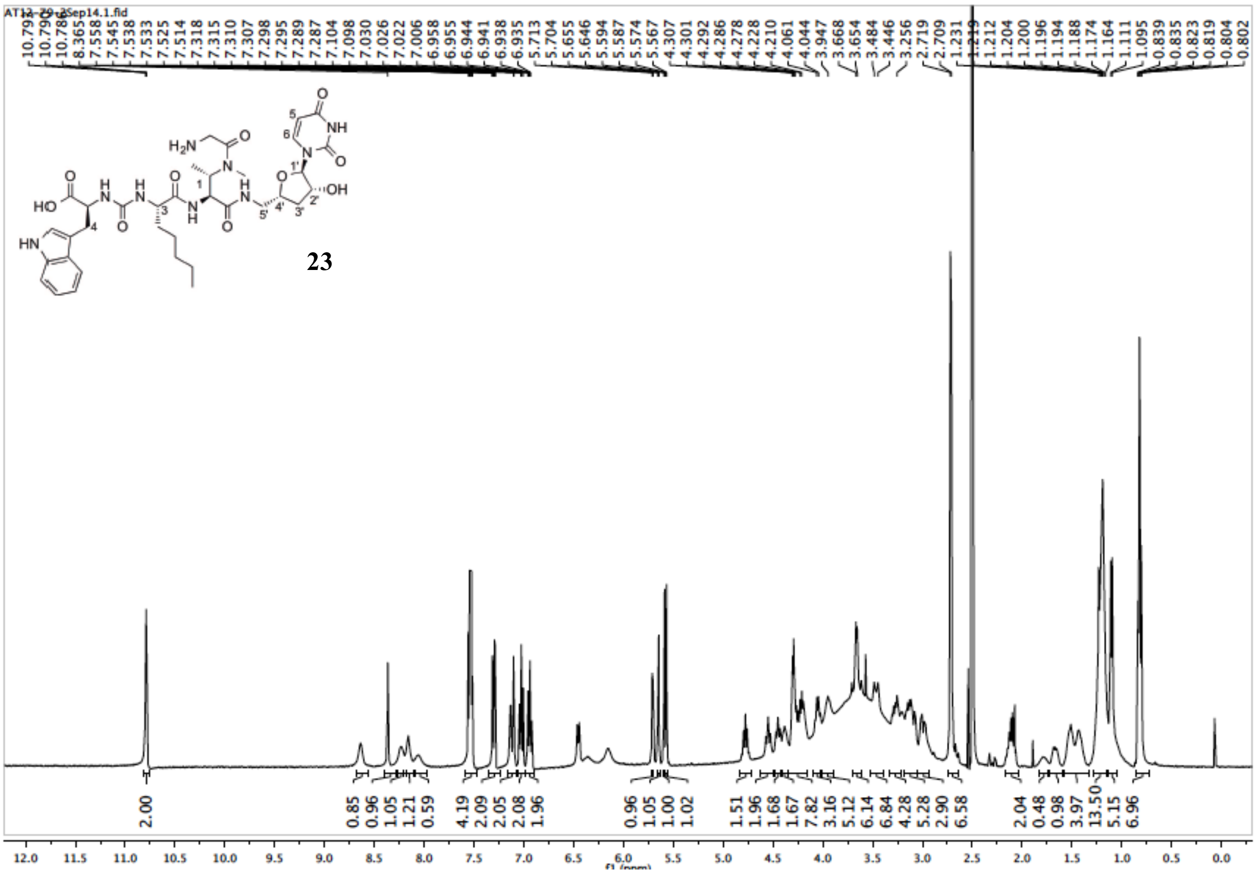

Supplementary Figure 119. ^{1}H NMR spectrum of compound **23**.

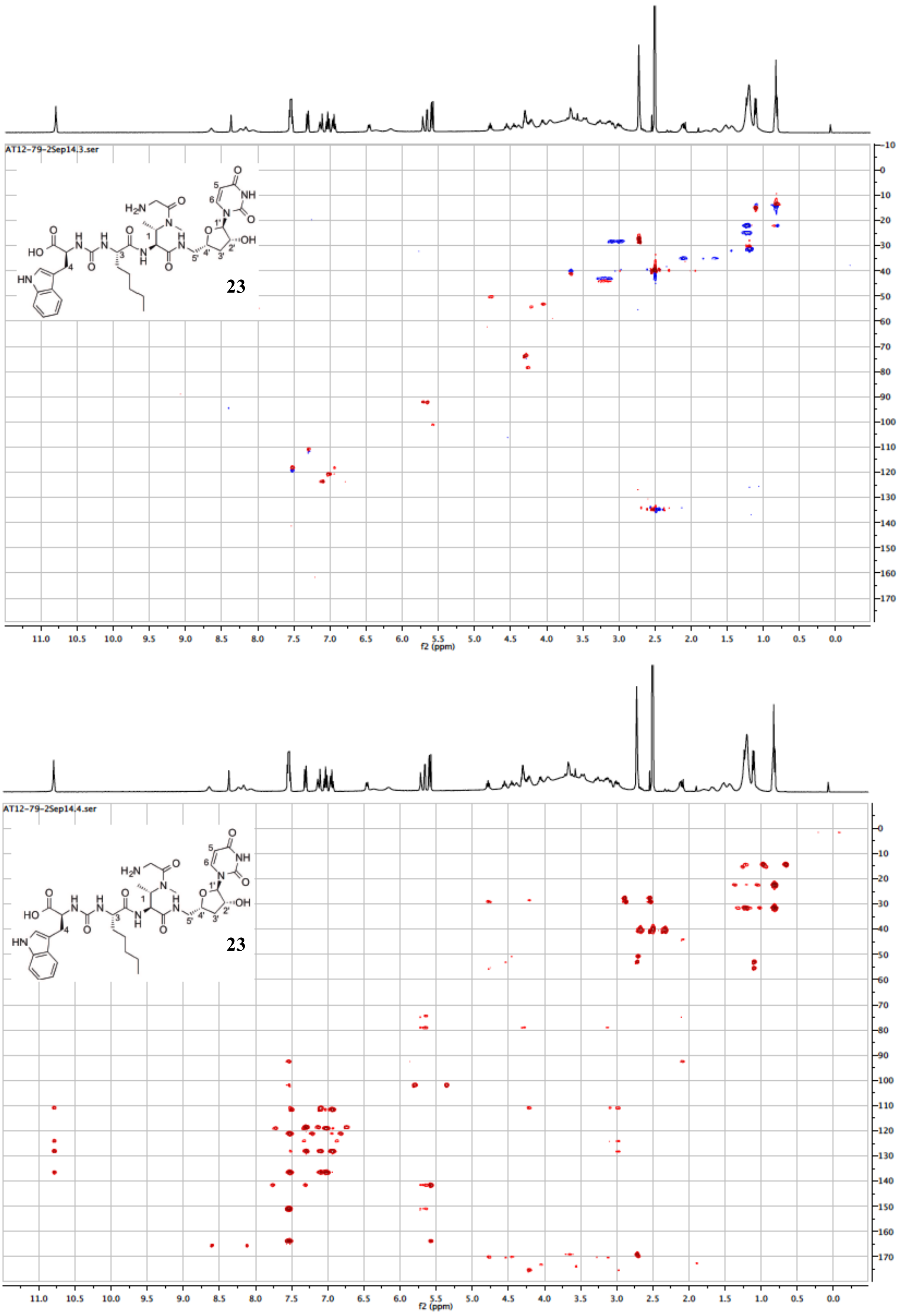

Supplementary Figure 120. HSQC (top) and HMBC (bottom) NMR spectra of compound **23**.

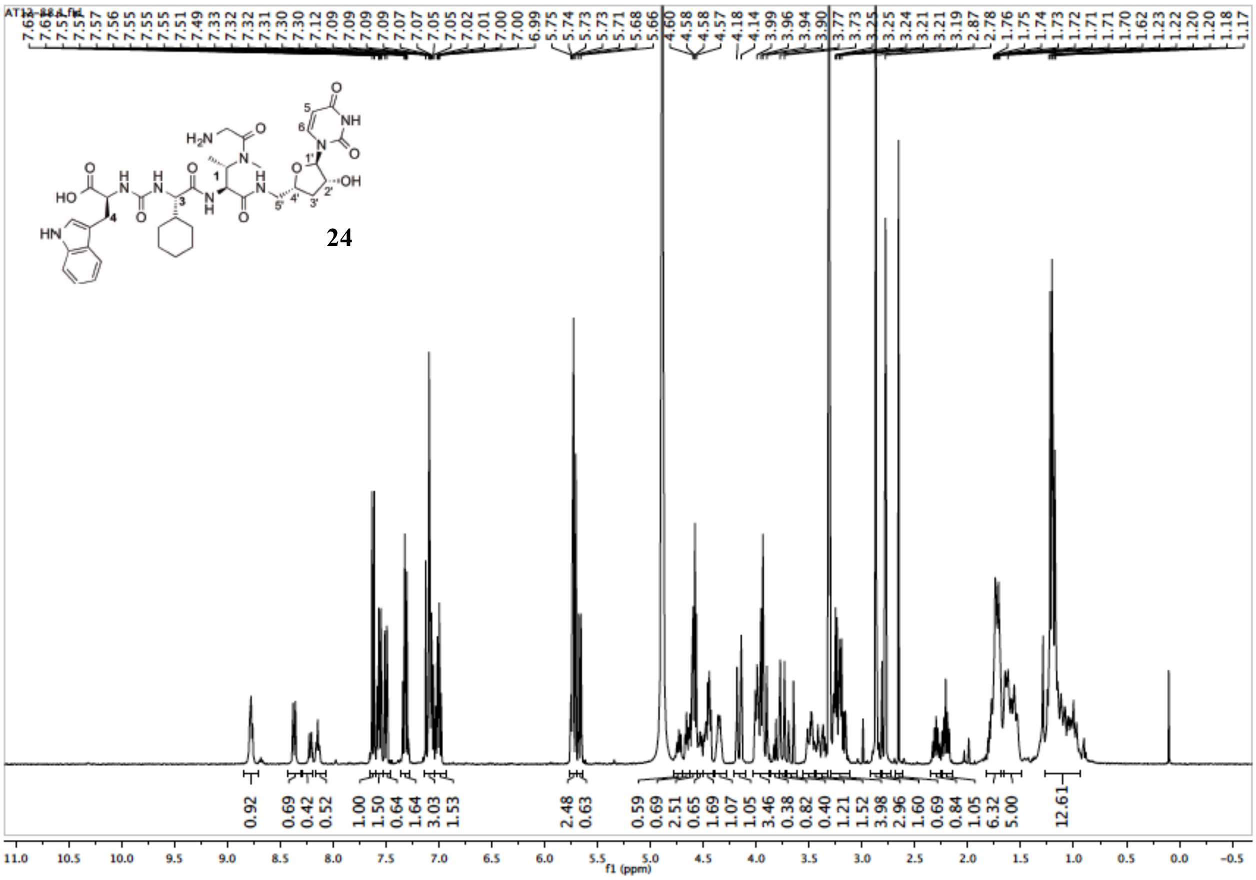

Supplementary Figure 121. ^{1}H NMR spectrum of compound **24**.

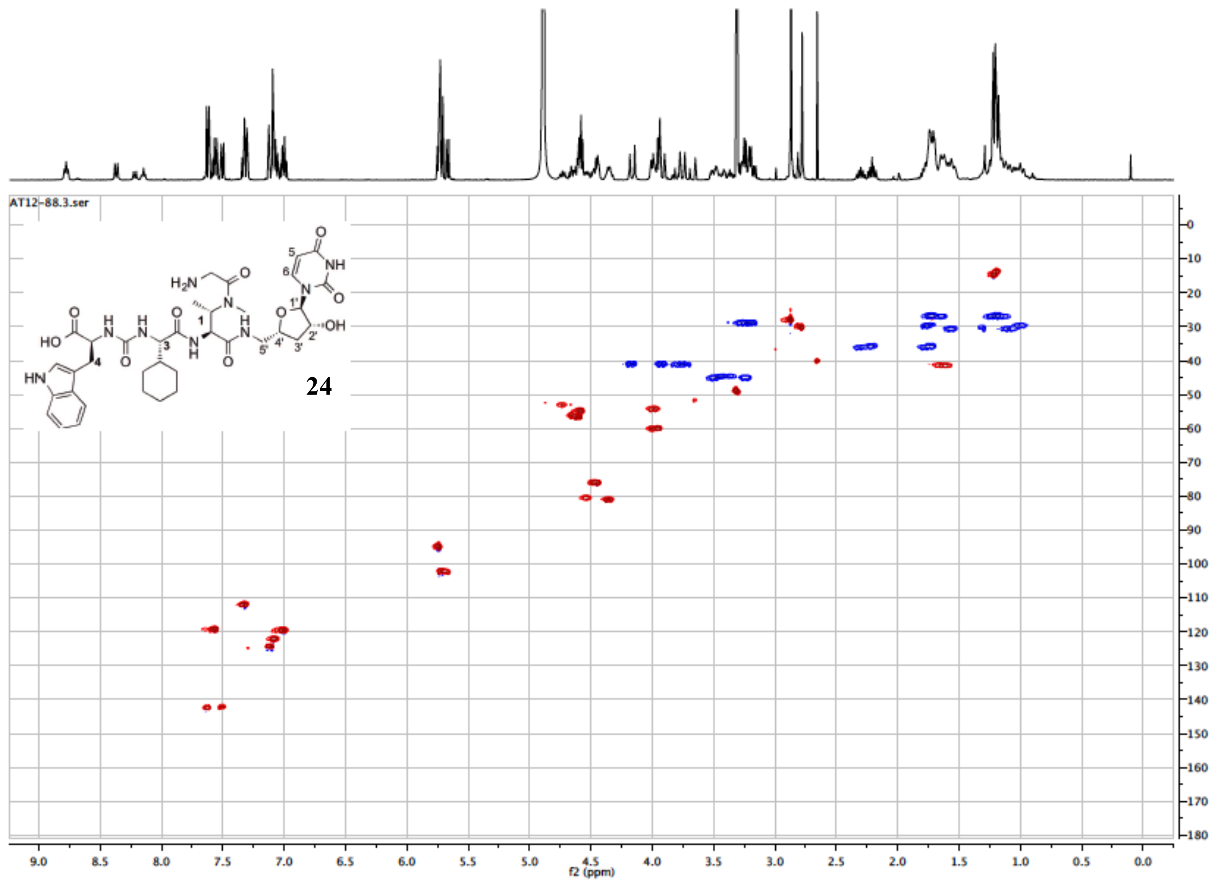

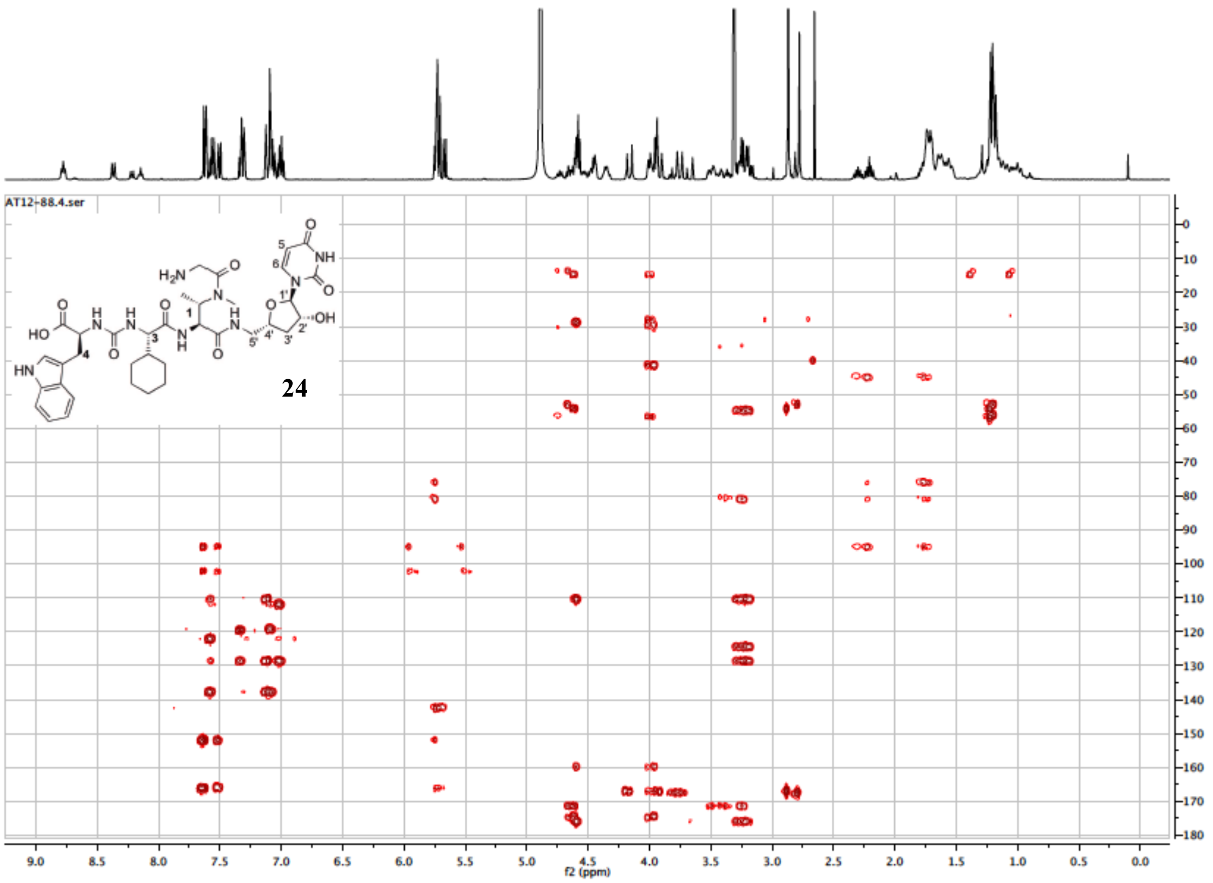

Supplementary Figure 122. HSQC (top) and HMBC (bottom) NMR spectra of compound **24**.

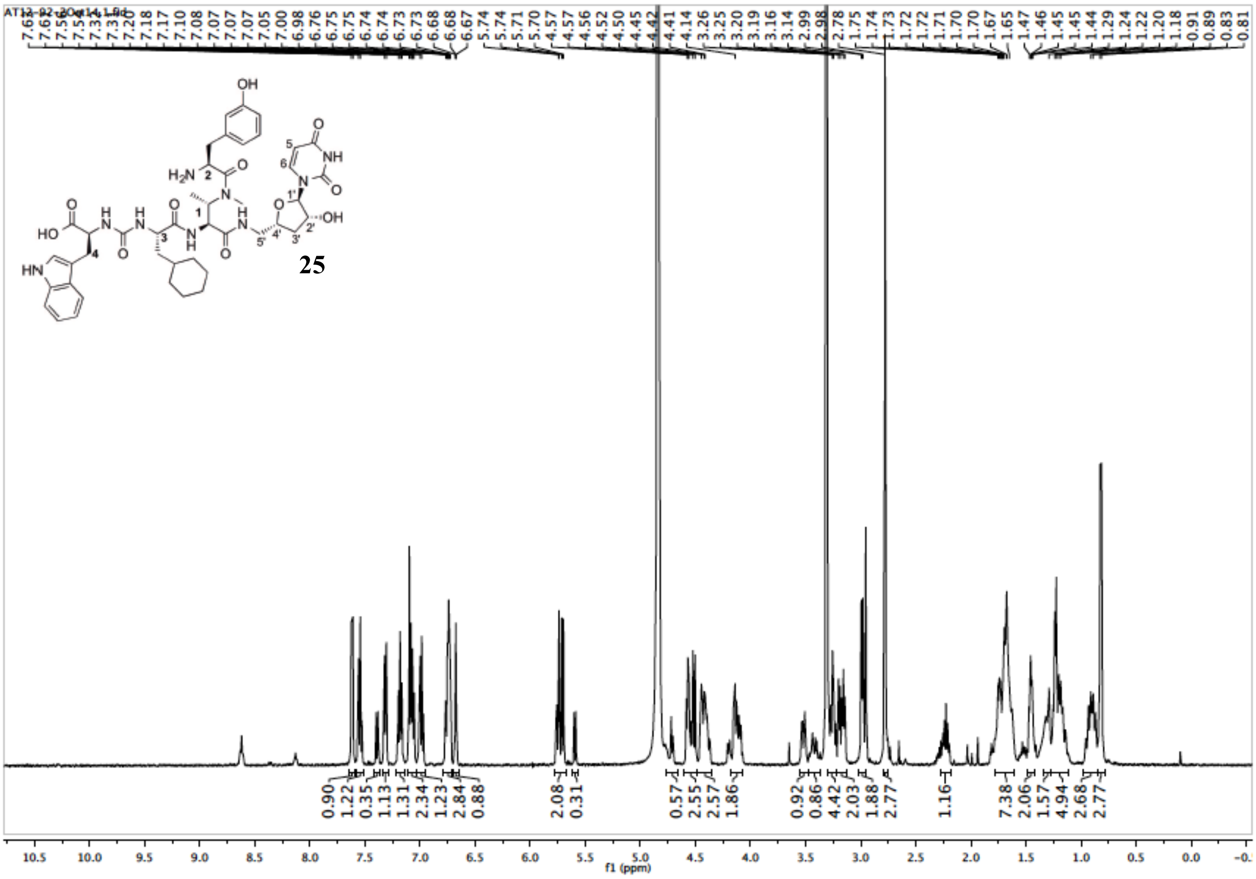

Supplementary Figure 123. ^{1}H NMR spectrum of compound **25**.

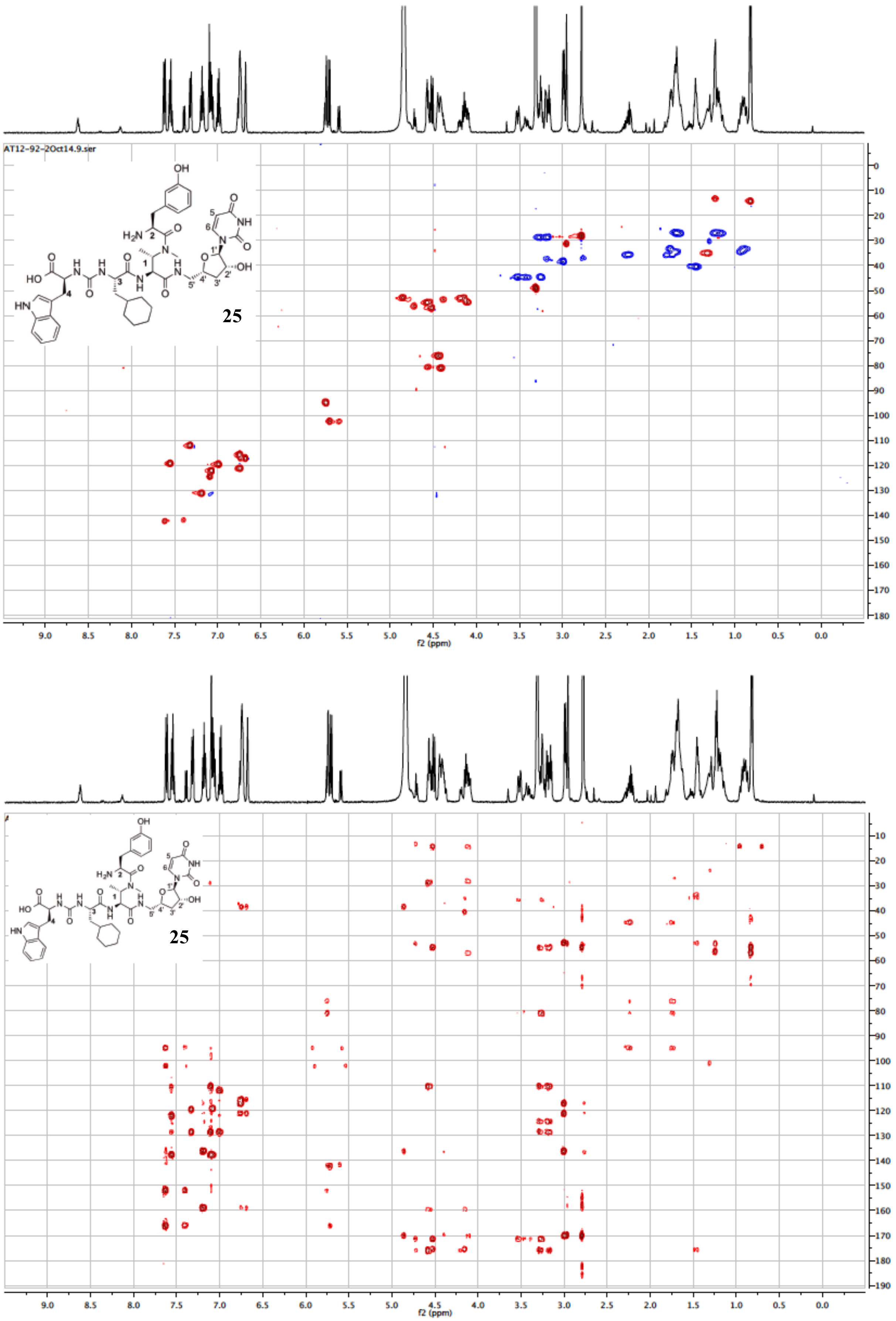

Supplementary Figure 124. HSQC (top) and HMBC (bottom) NMR spectra of compound **25**.

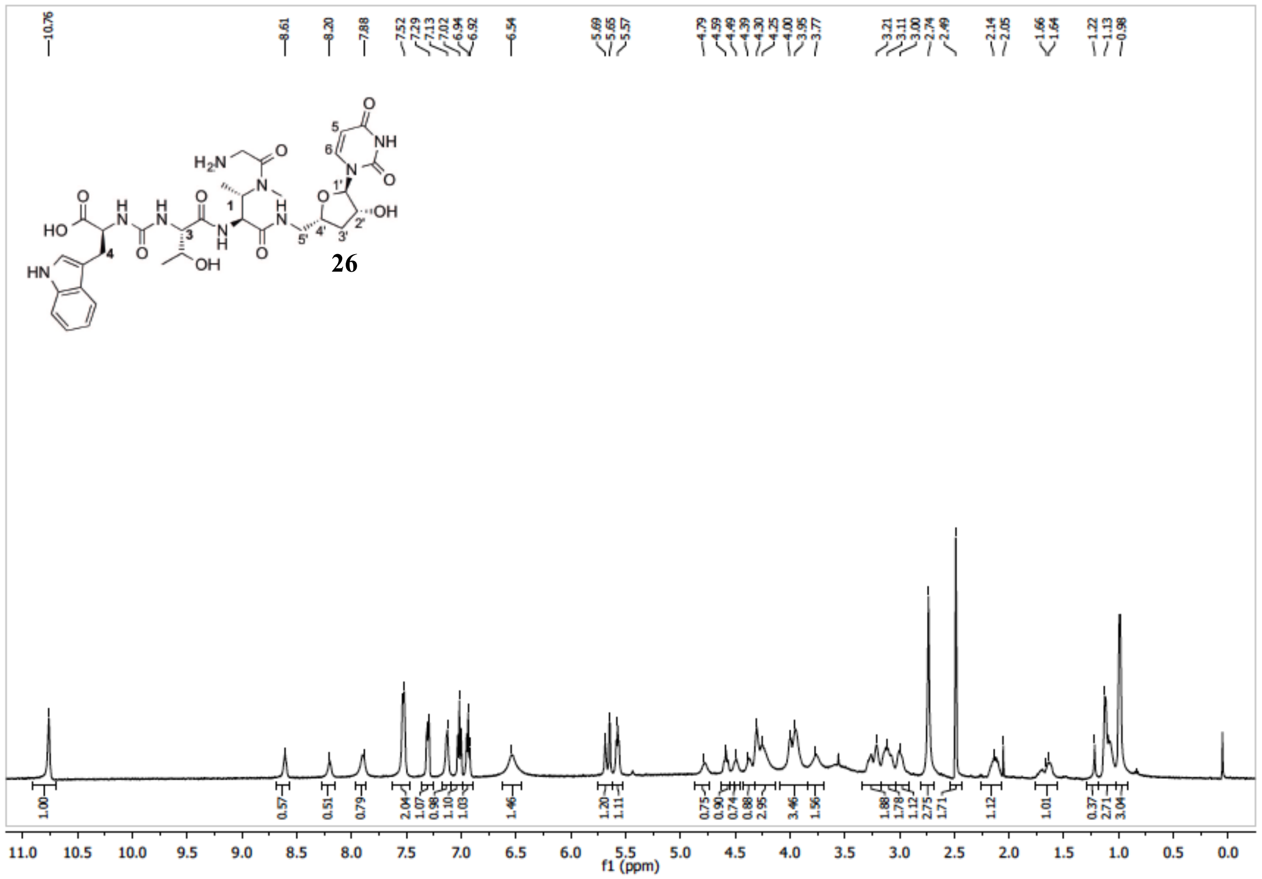

Supplementary Figure 125. ^{1}H NMR spectrum of compound **26**.

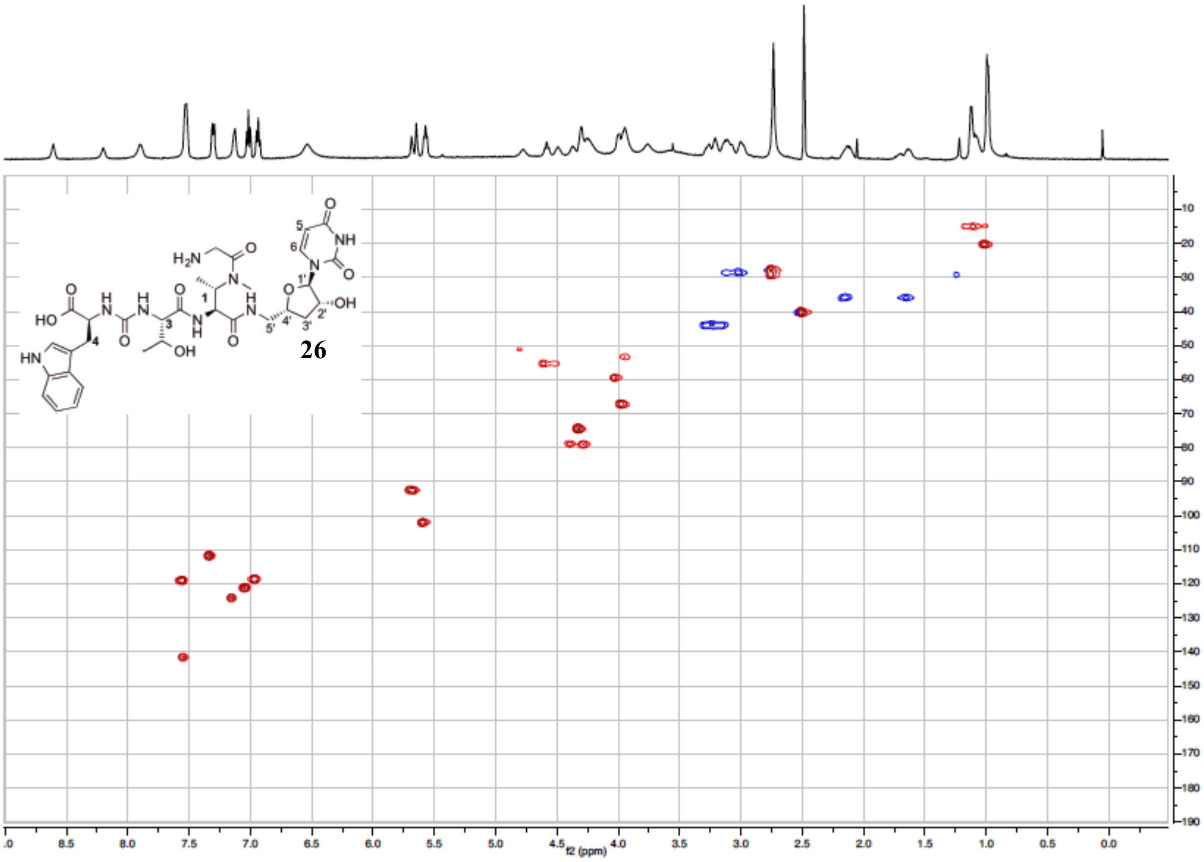

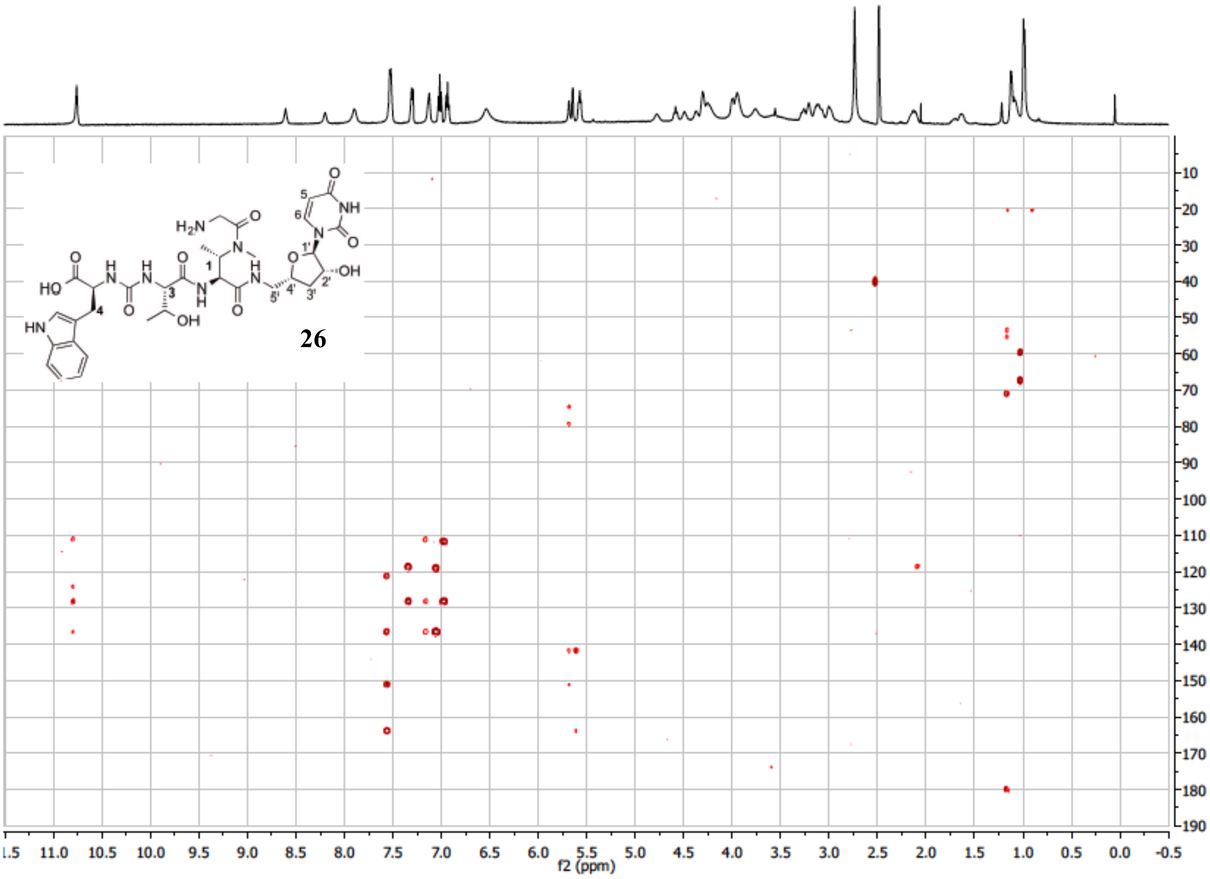

Supplementary Figure 126. HSQC (top) and HMBC (bottom) NMR spectra of compound **26**.

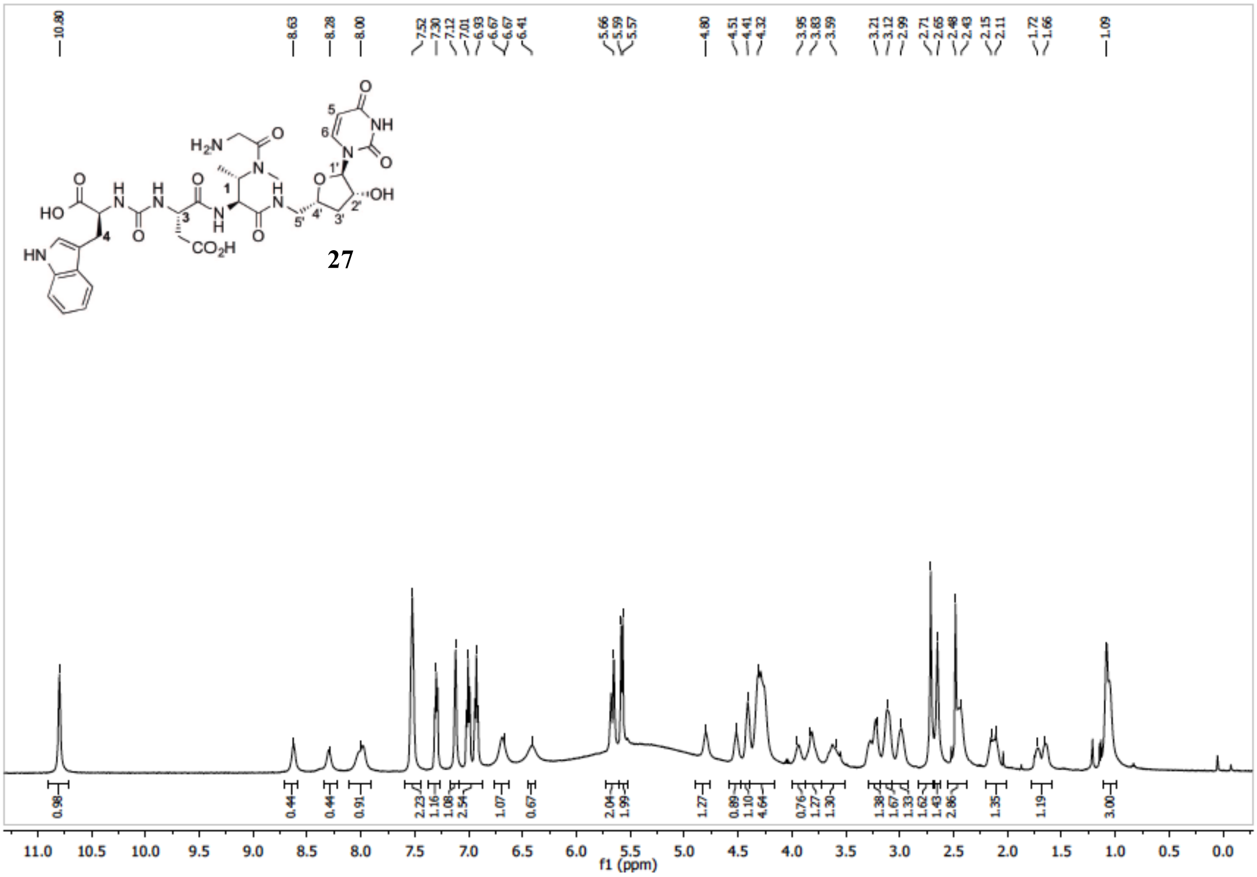

Supplementary Figure 127. ^{1}H NMR spectrum of compound **27**.

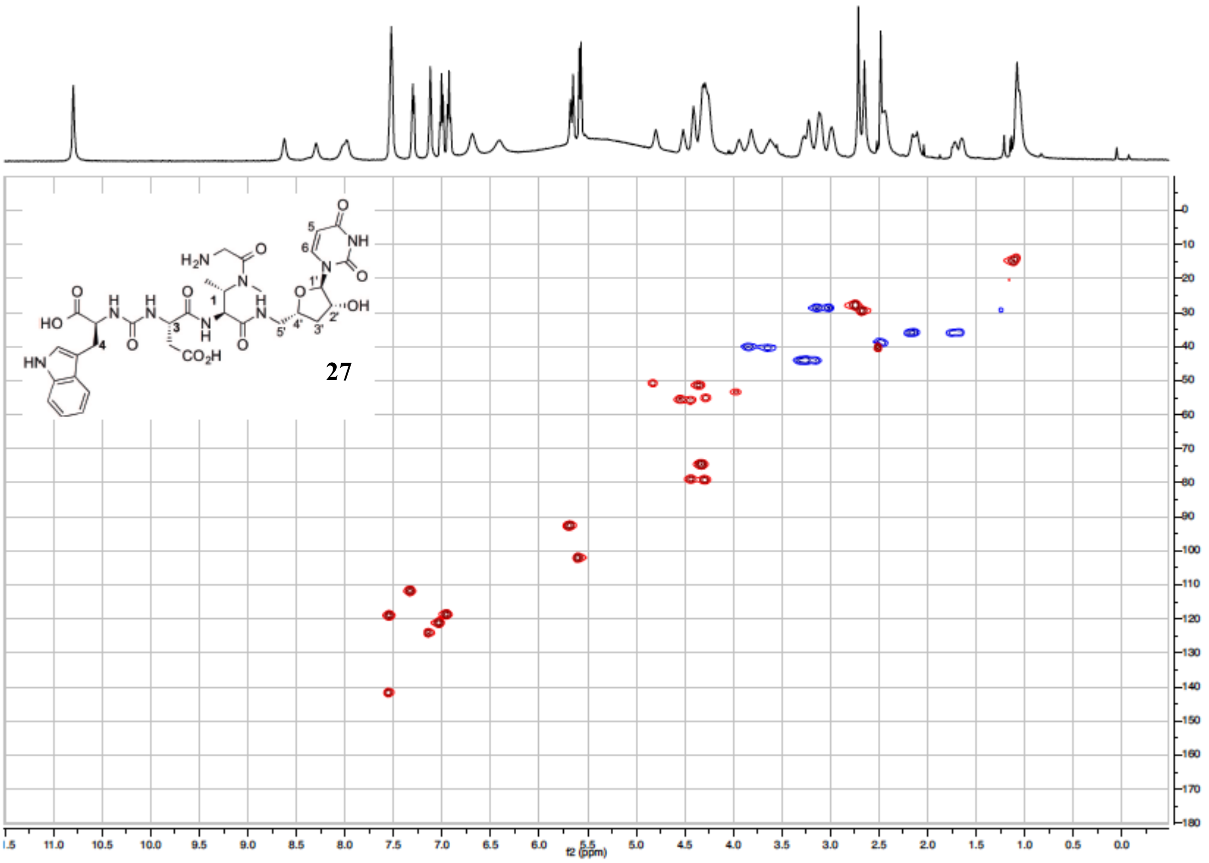

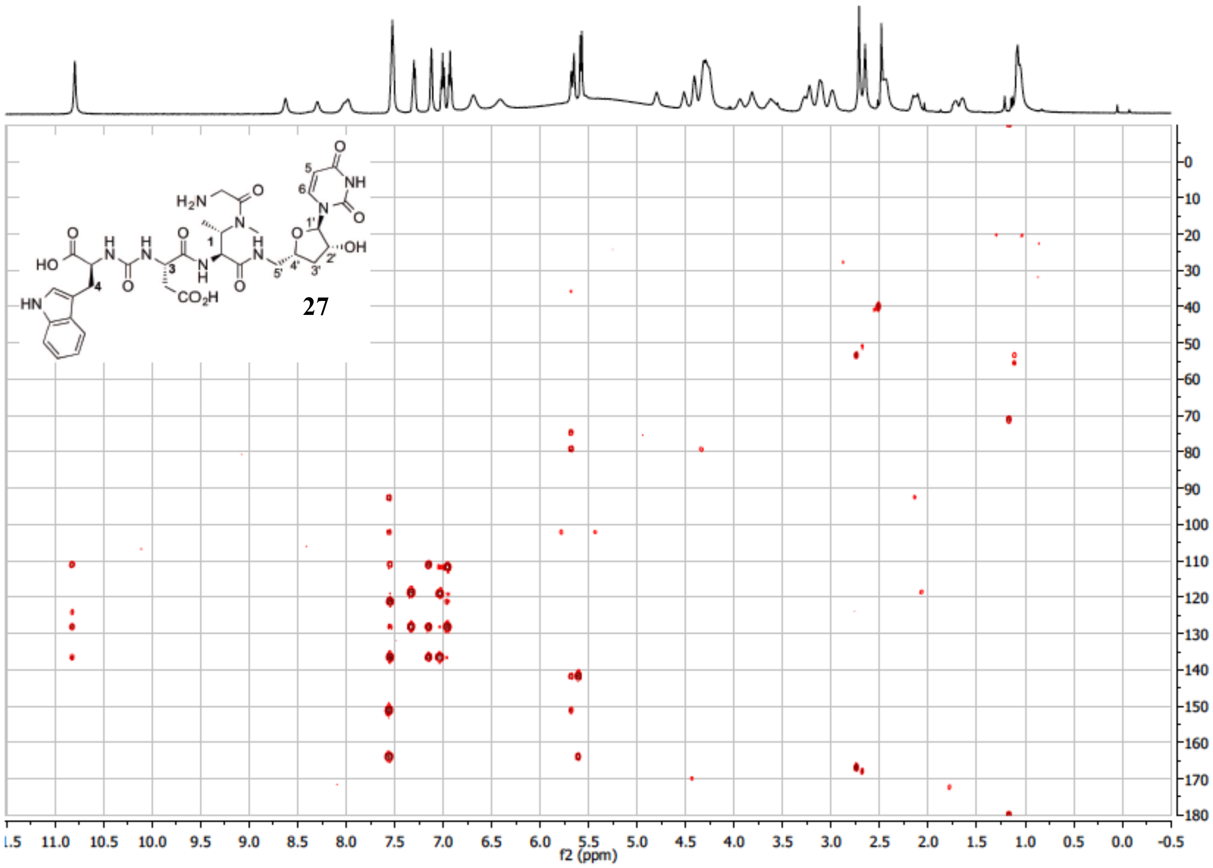

Supplementary Figure 128. HSQC (top) and HMBC (bottom) NMR spectra of compound **27**.

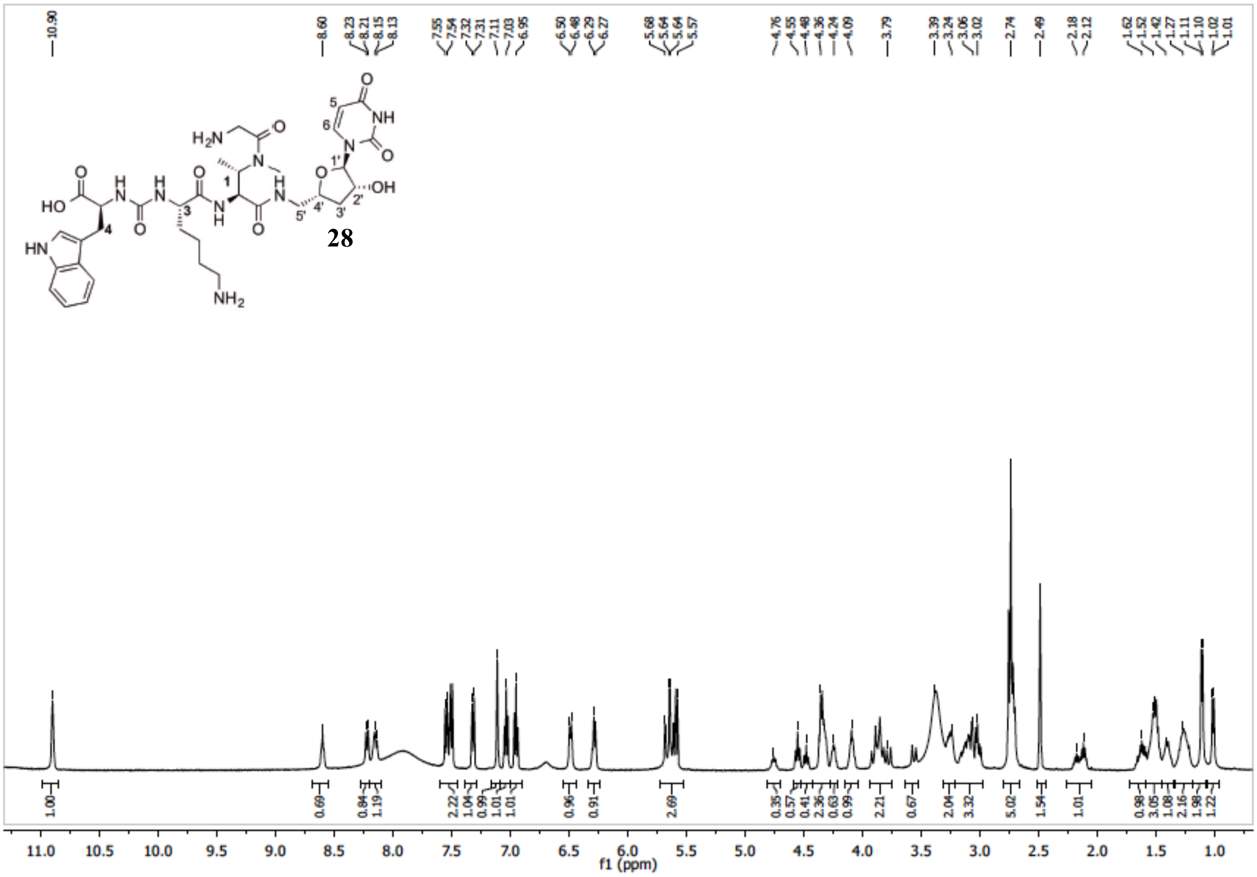

Supplementary Figure 129. ^{1}H NMR spectrum of compound **28**.

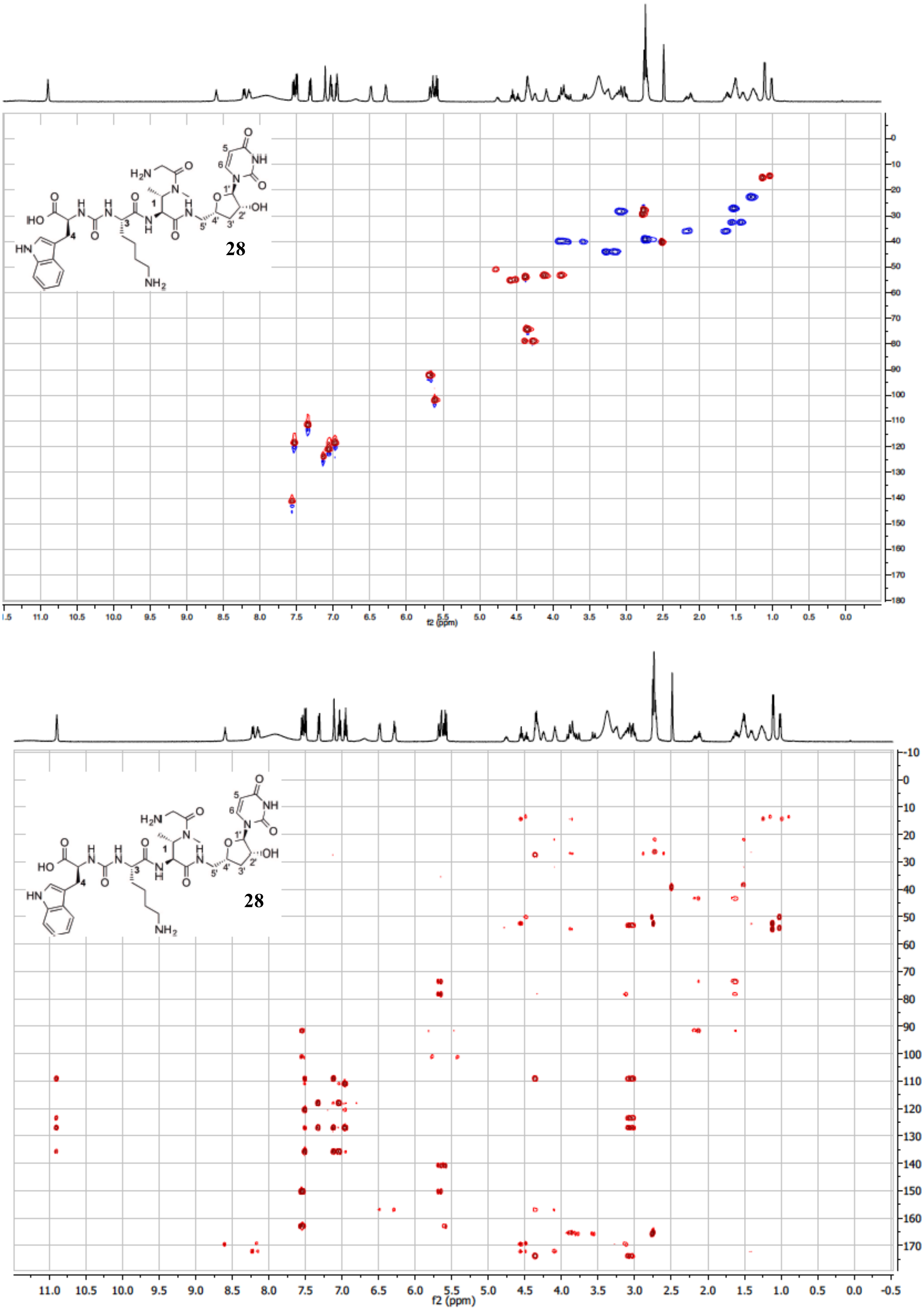

Supplementary Figure 130. HSQC (top) and HMBC (bottom) NMR spectra of compound **28**.

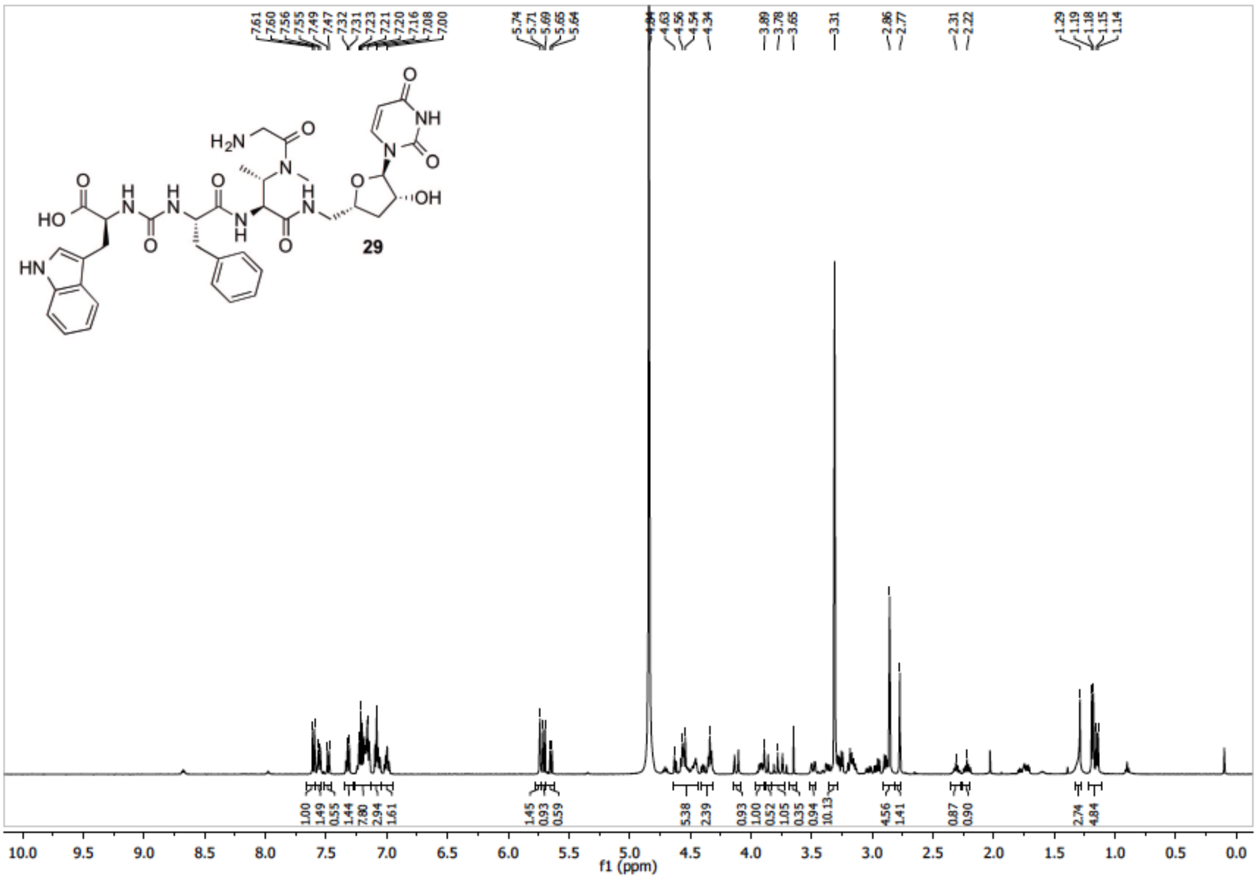

Supplementary Figure 131. ^{1}H NMR spectrum of compound **29**.

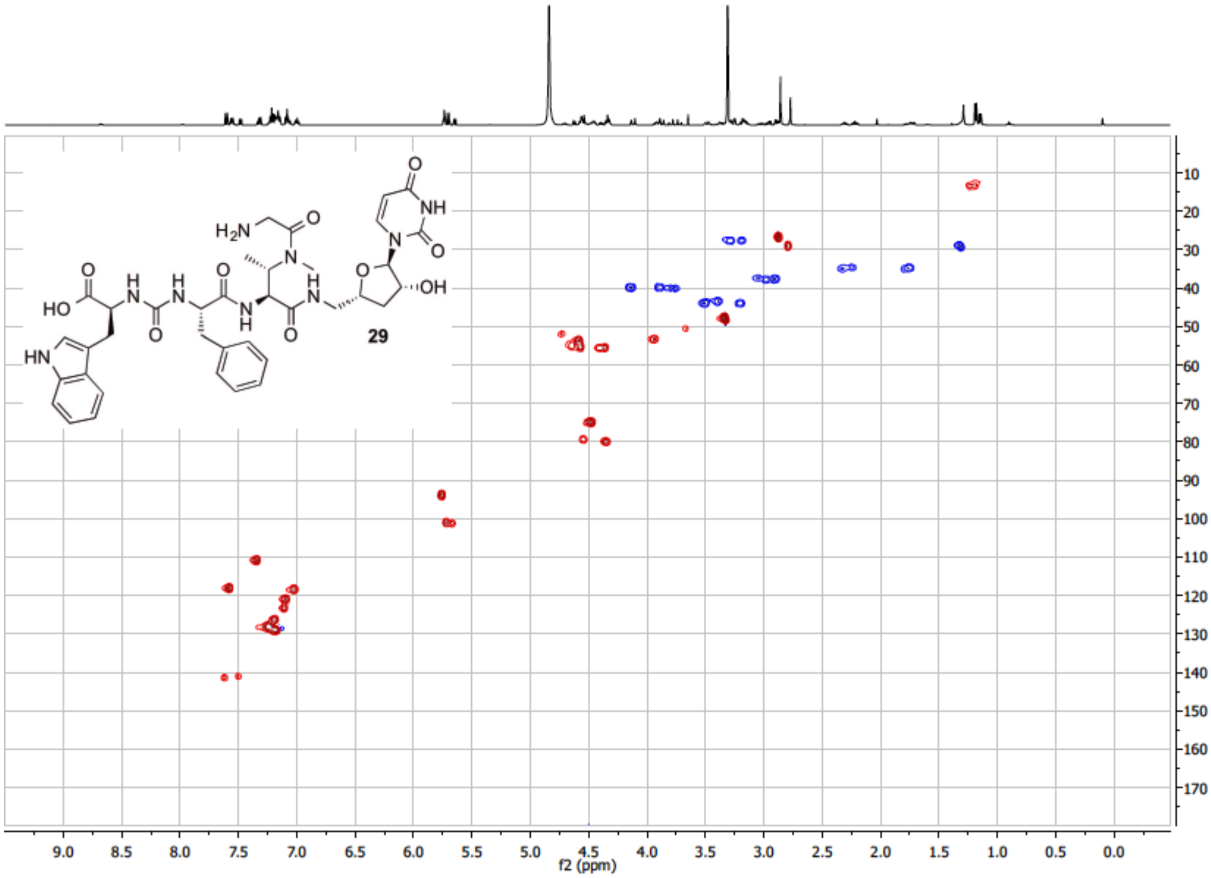

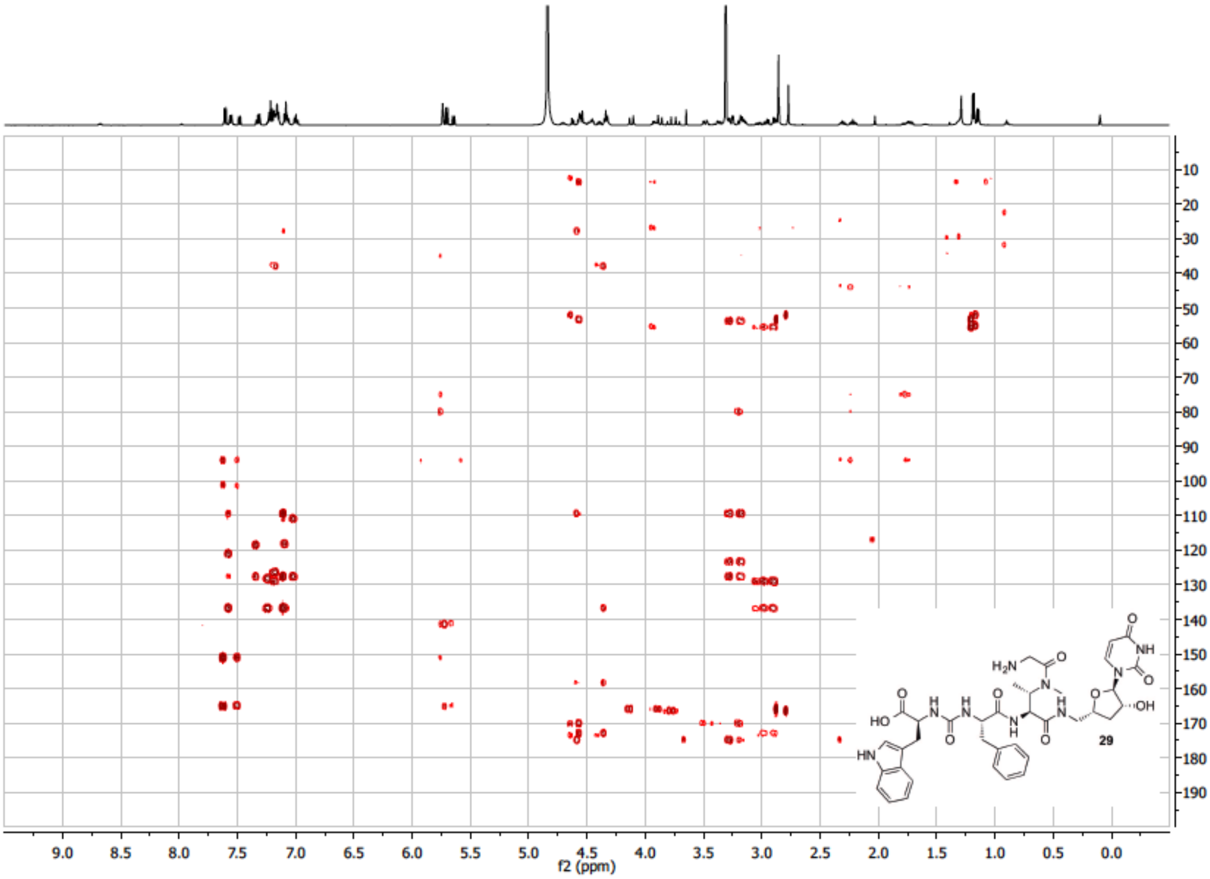

Supplementary Figure 132. HSQC (top) and HMBC (bottom) NMR spectra of compound **29**.

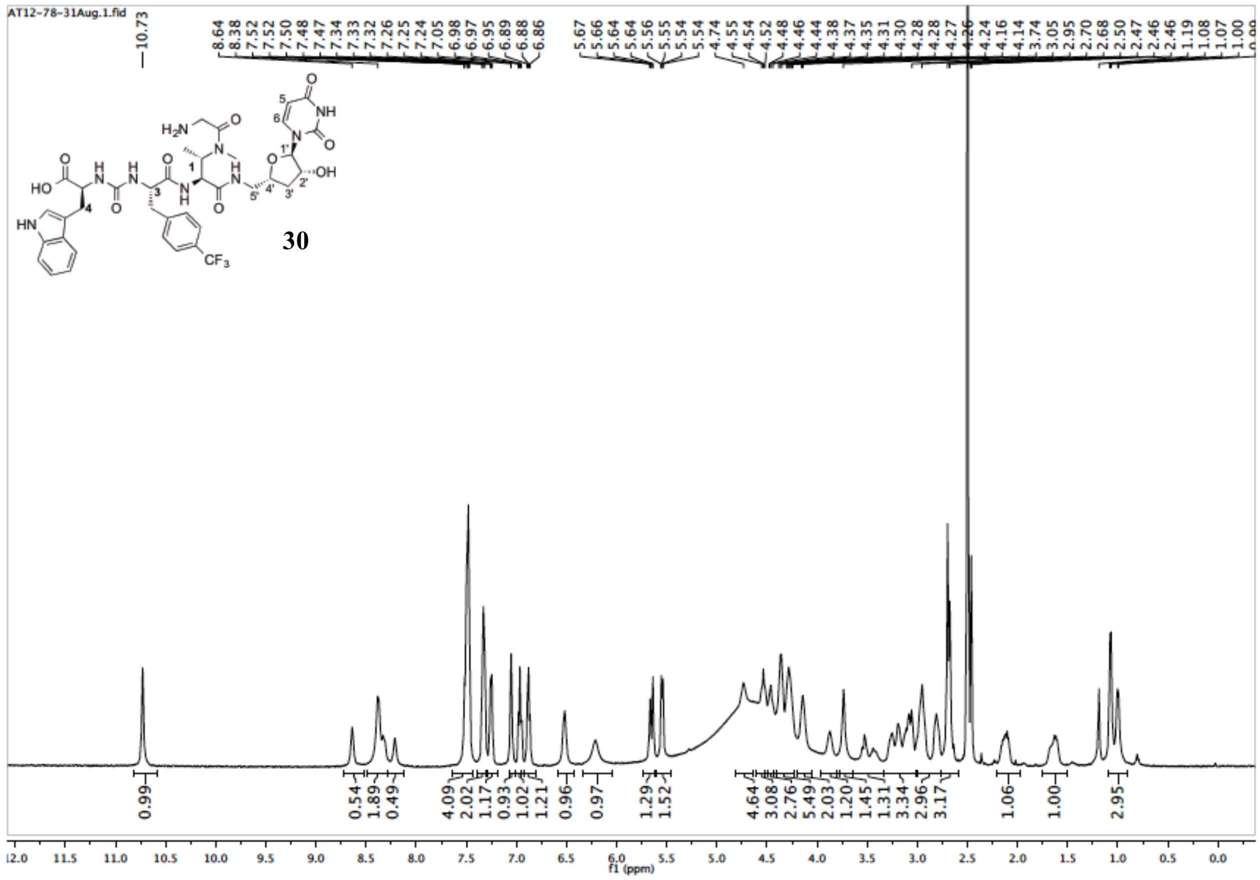

Supplementary Figure 133. ^{1}H NMR spectrum of compound **30**.

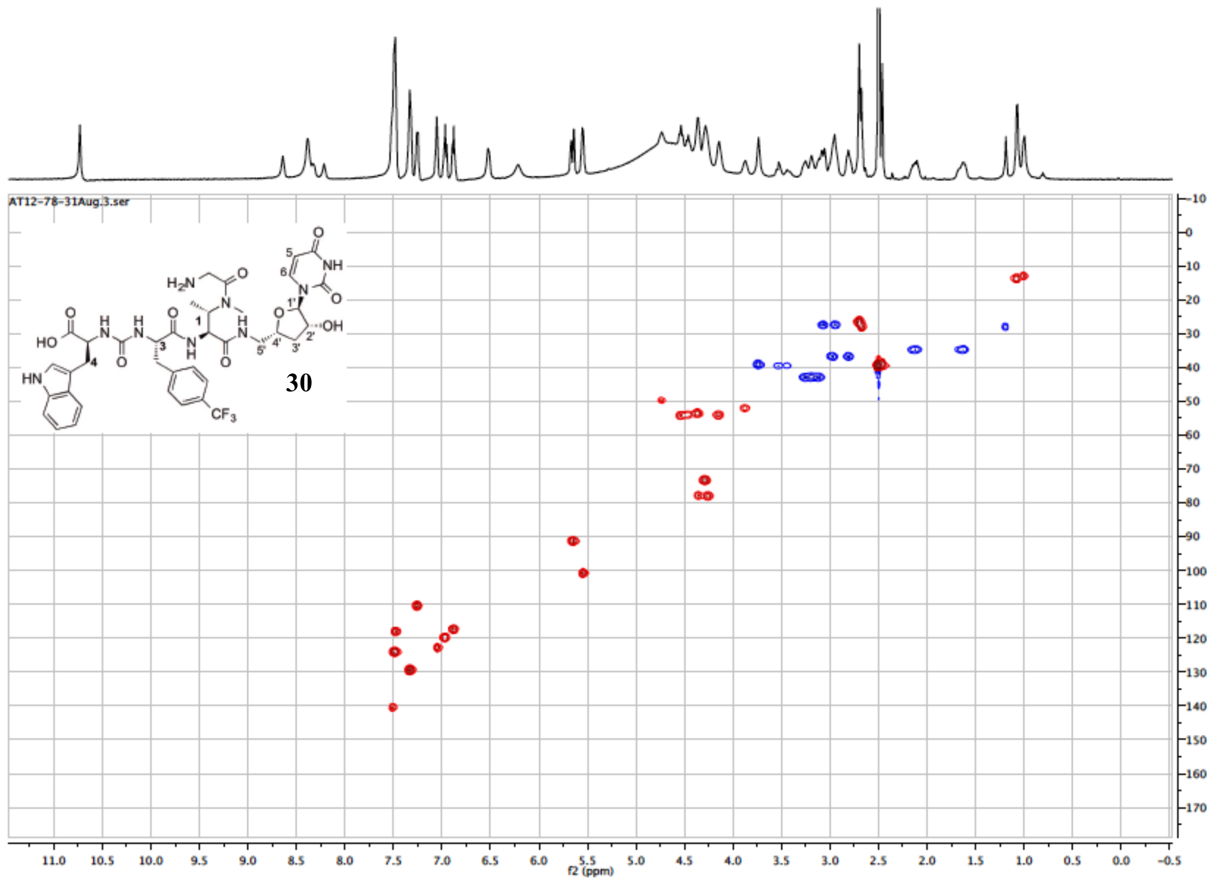

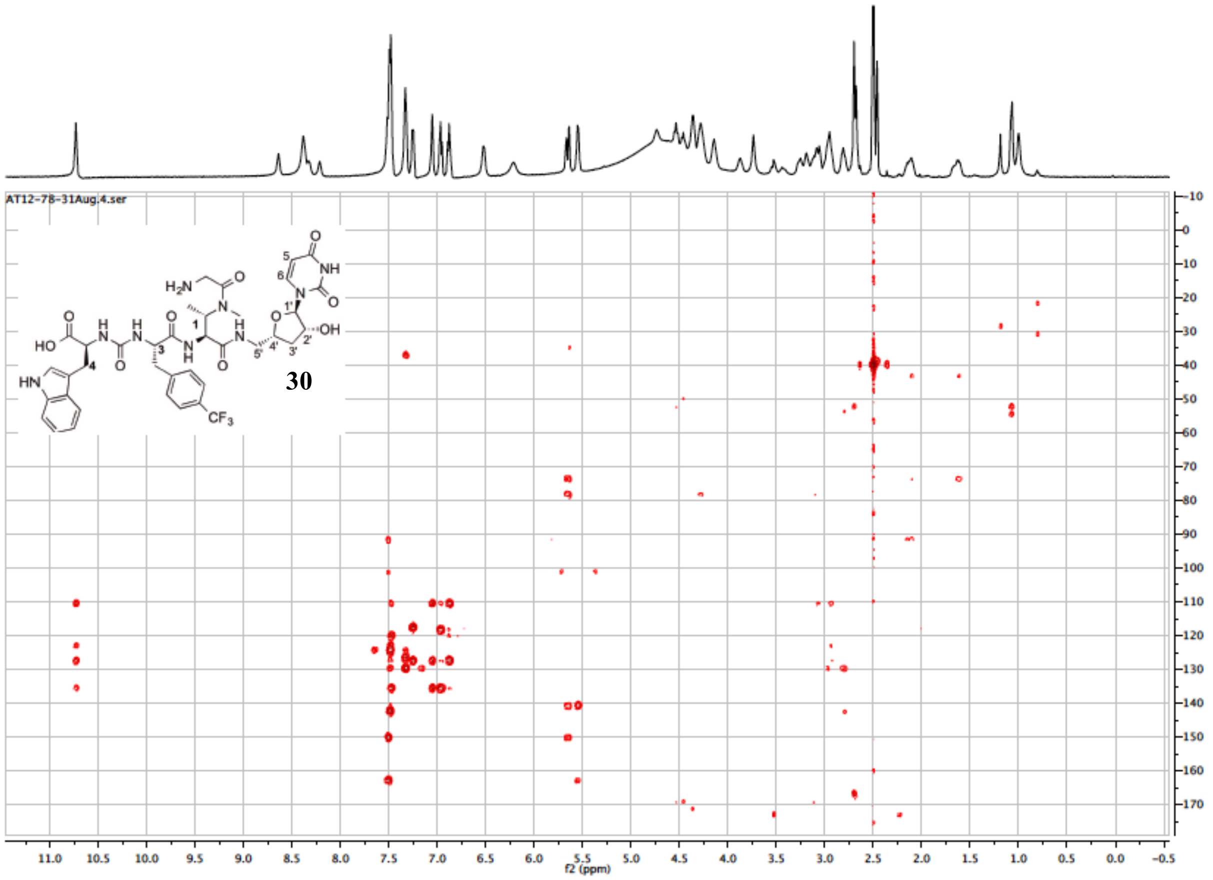

Supplementary Figure 134. HSQC (top) and HMBC (bottom) NMR spectra of compound **30**.

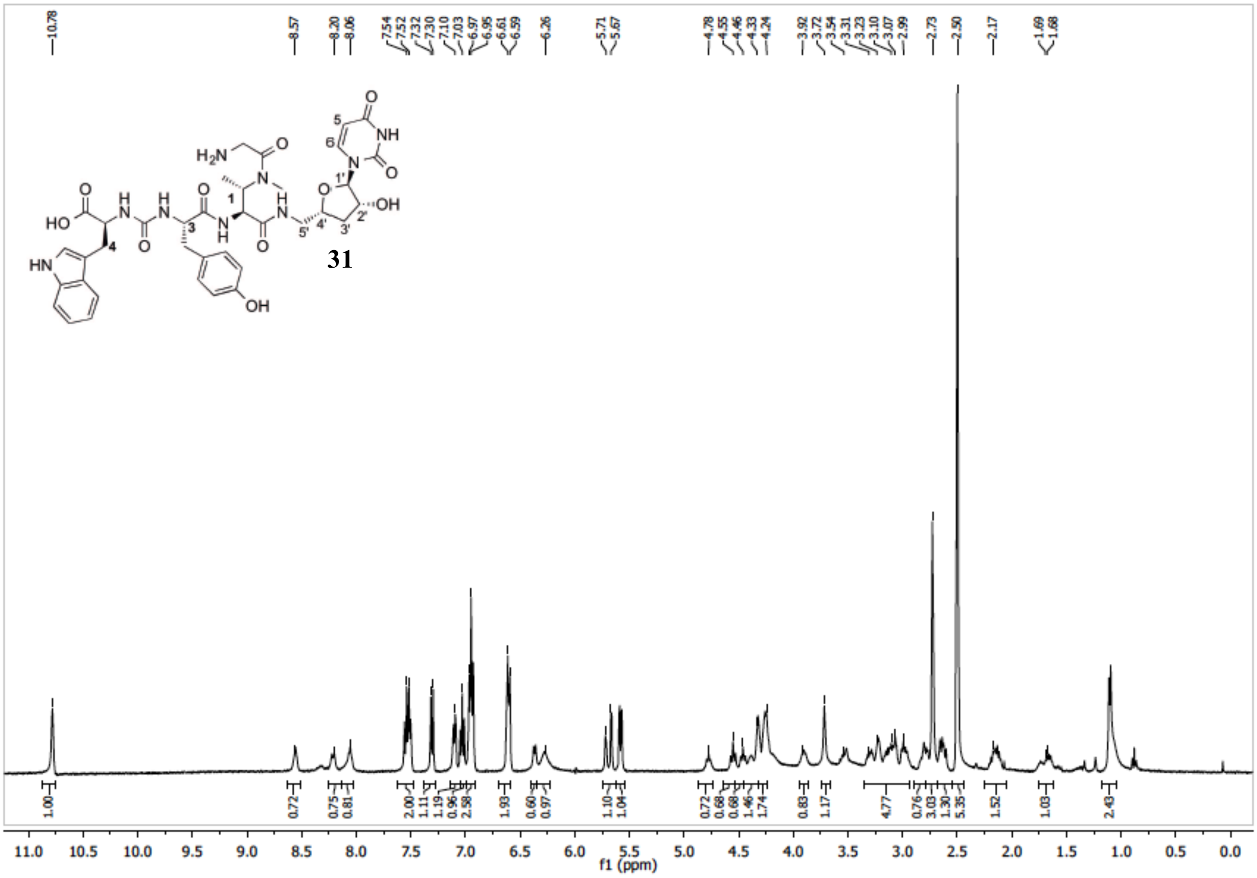

Supplementary Figure 135. ^{1}H NMR spectrum of compound **31**.

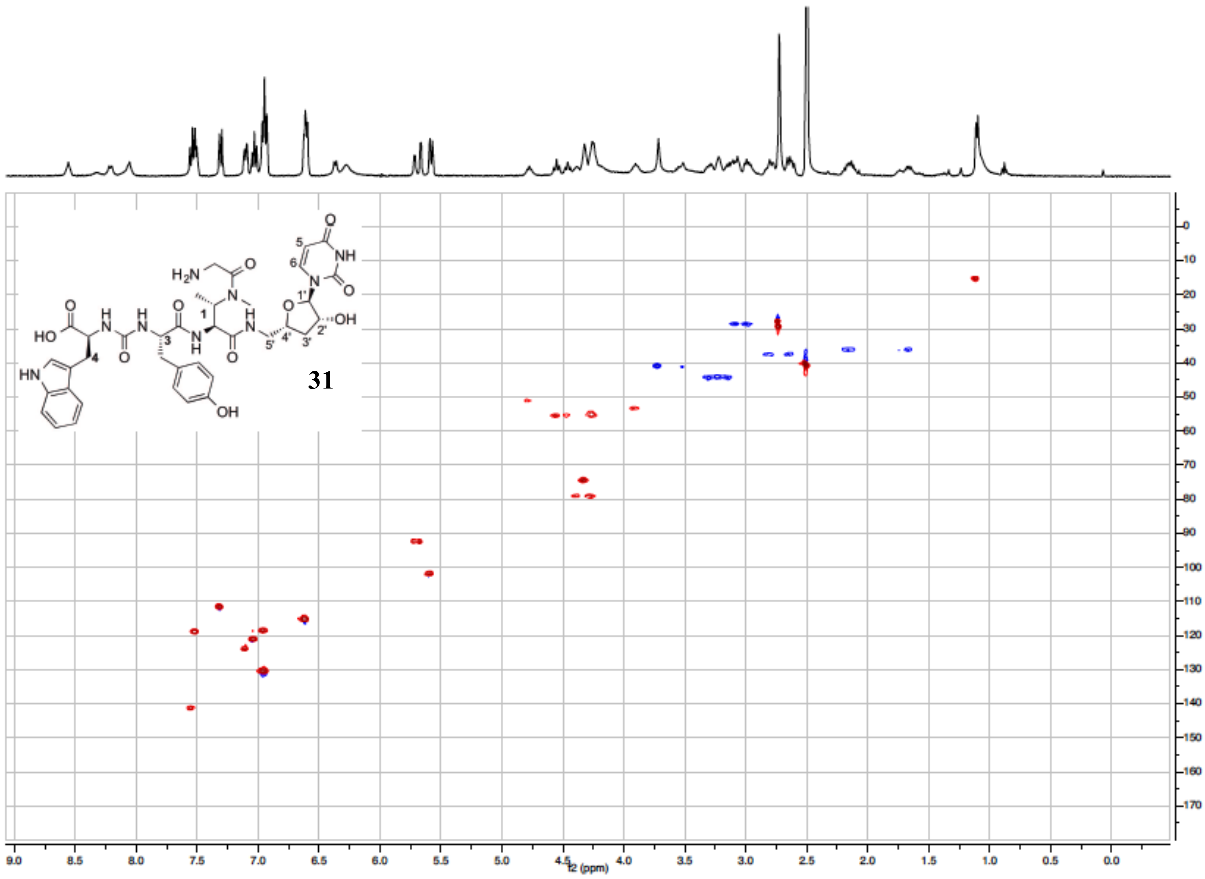

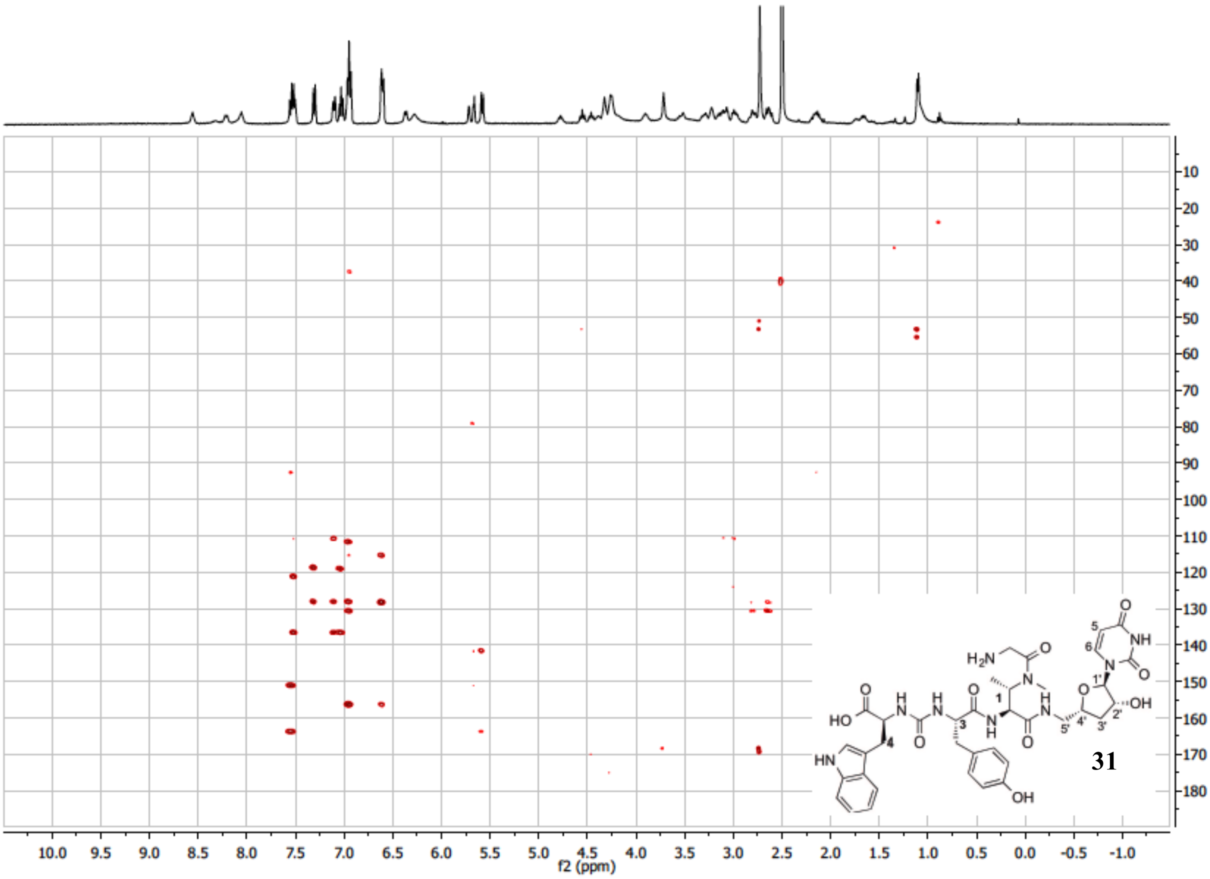

Supplementary Figure 136. HSQC (top) and HMBC (bottom) NMR spectra of compound **31**.

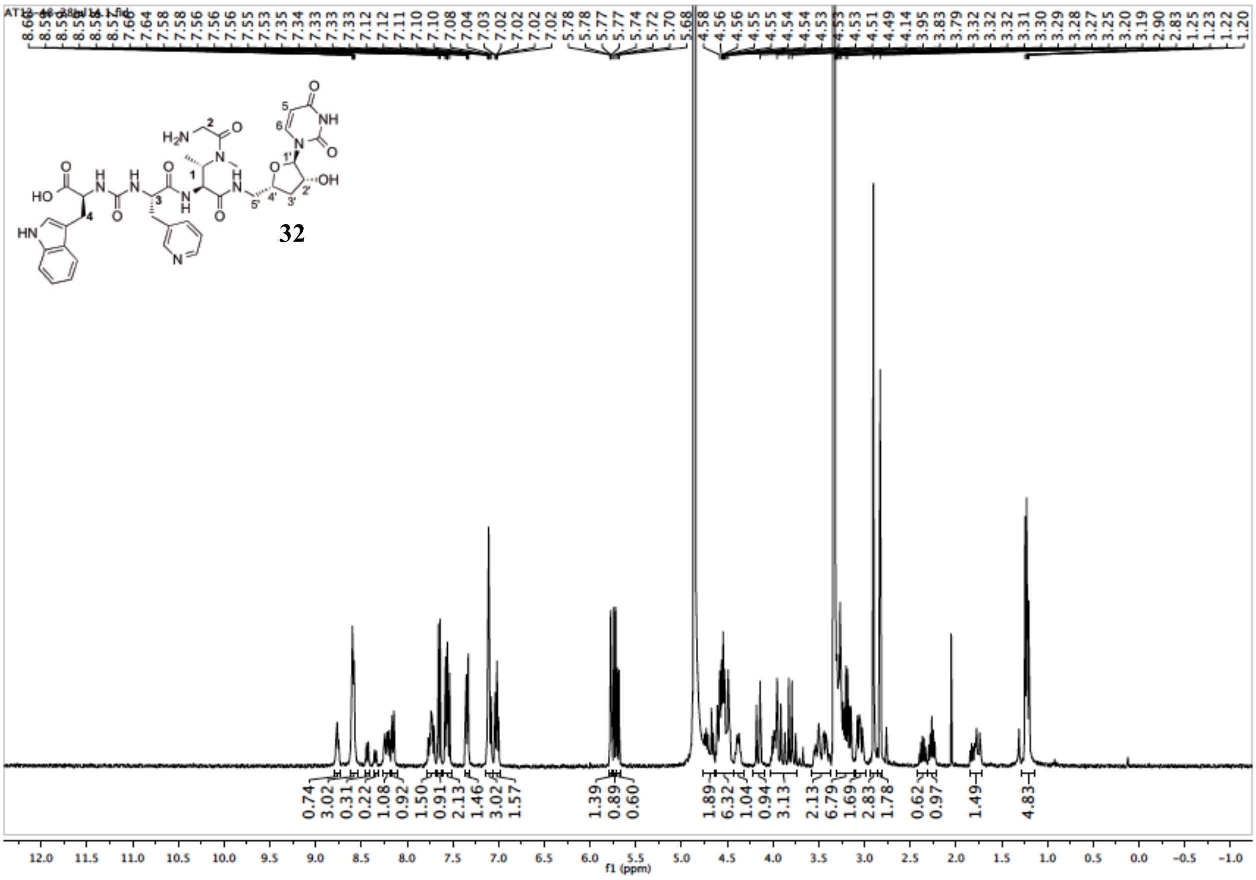

Supplementary Figure 137. ^{1}H NMR spectrum of compound **32**.

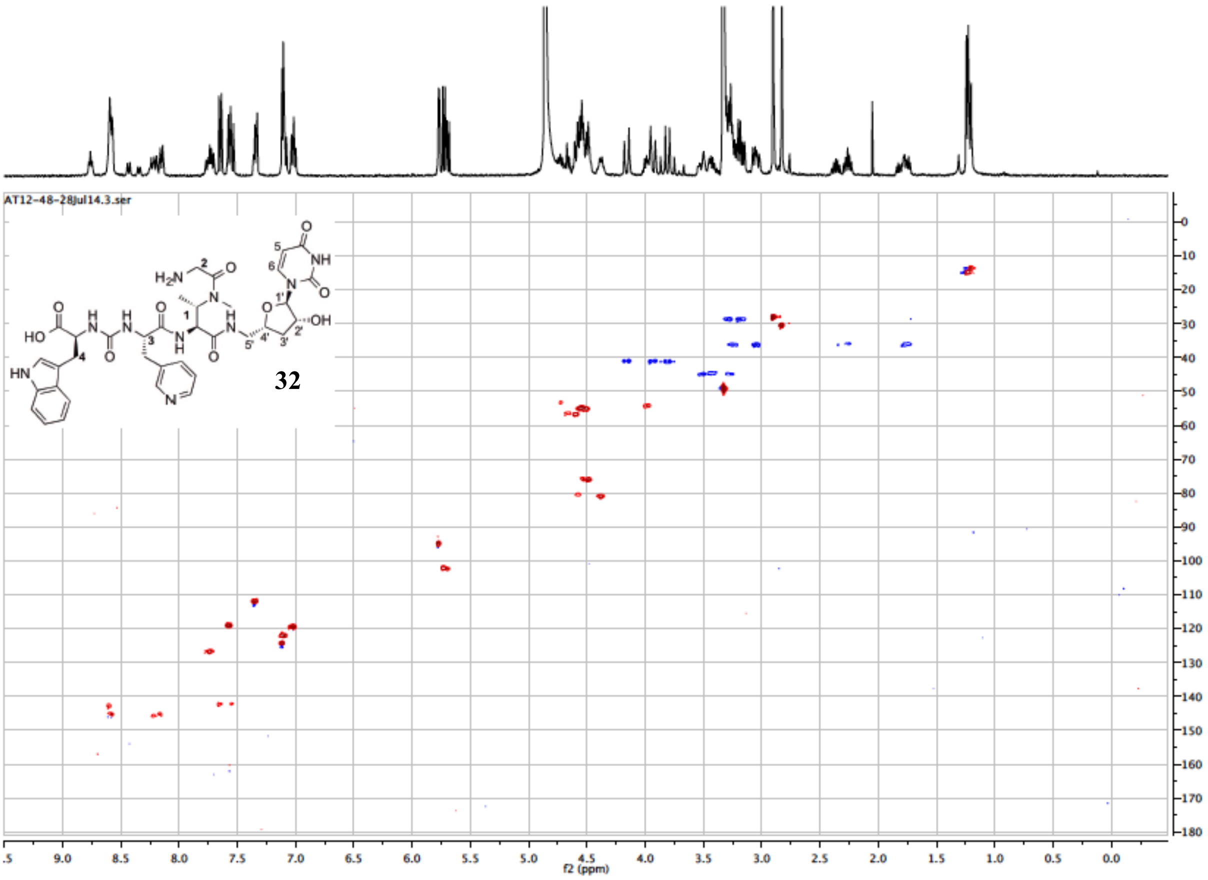

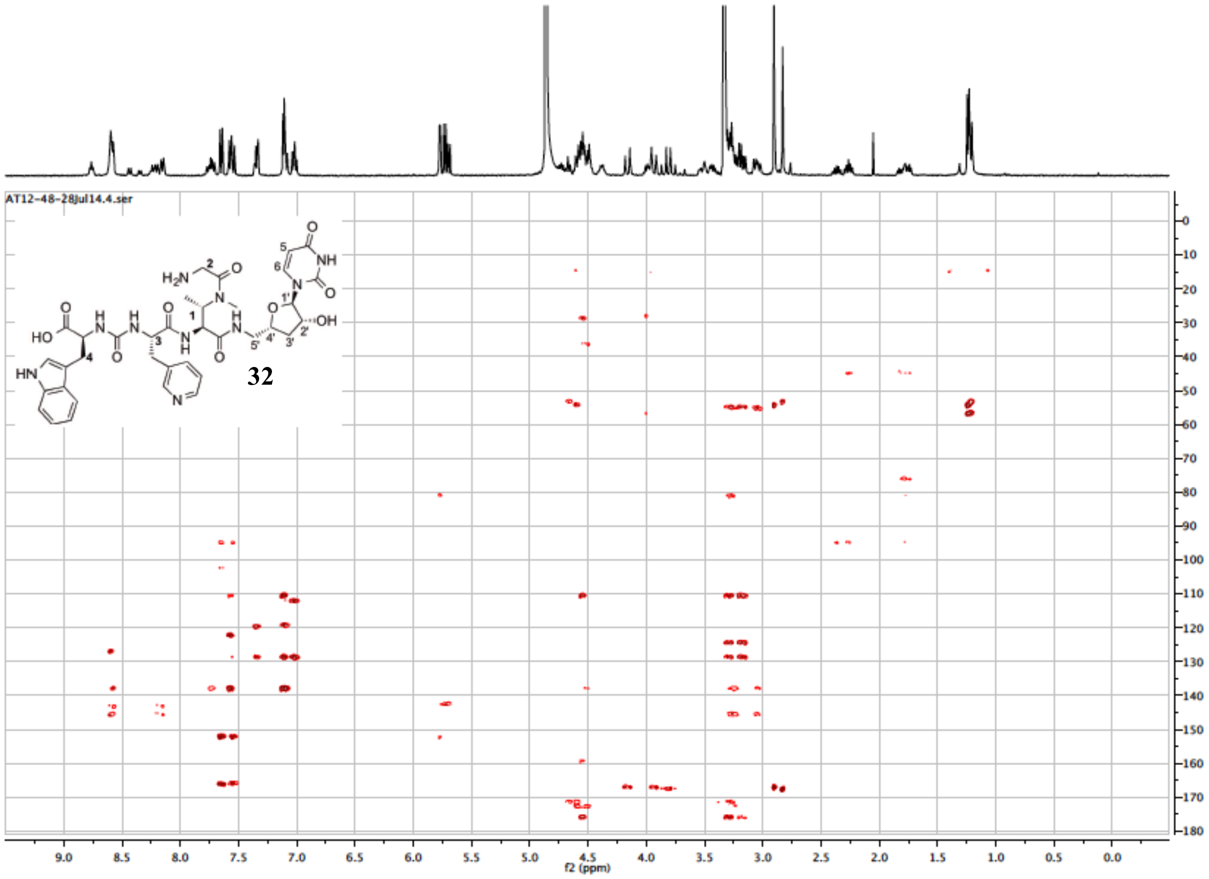

Supplementary Figure 138. HSQC (top) and HMBC (bottom) NMR spectra of compound **32**.

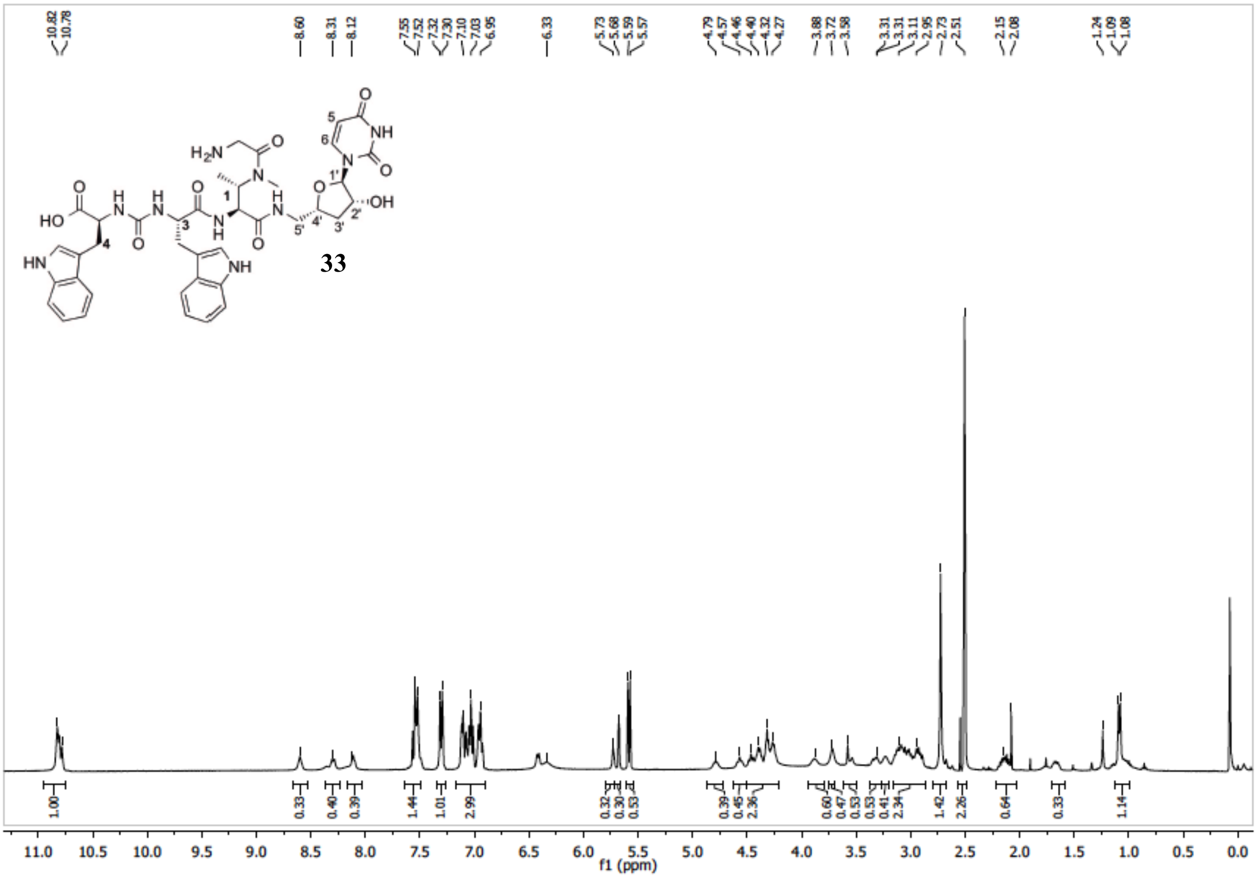

Supplementary Figure 139. ^{1}H NMR spectrum of compound **33**.

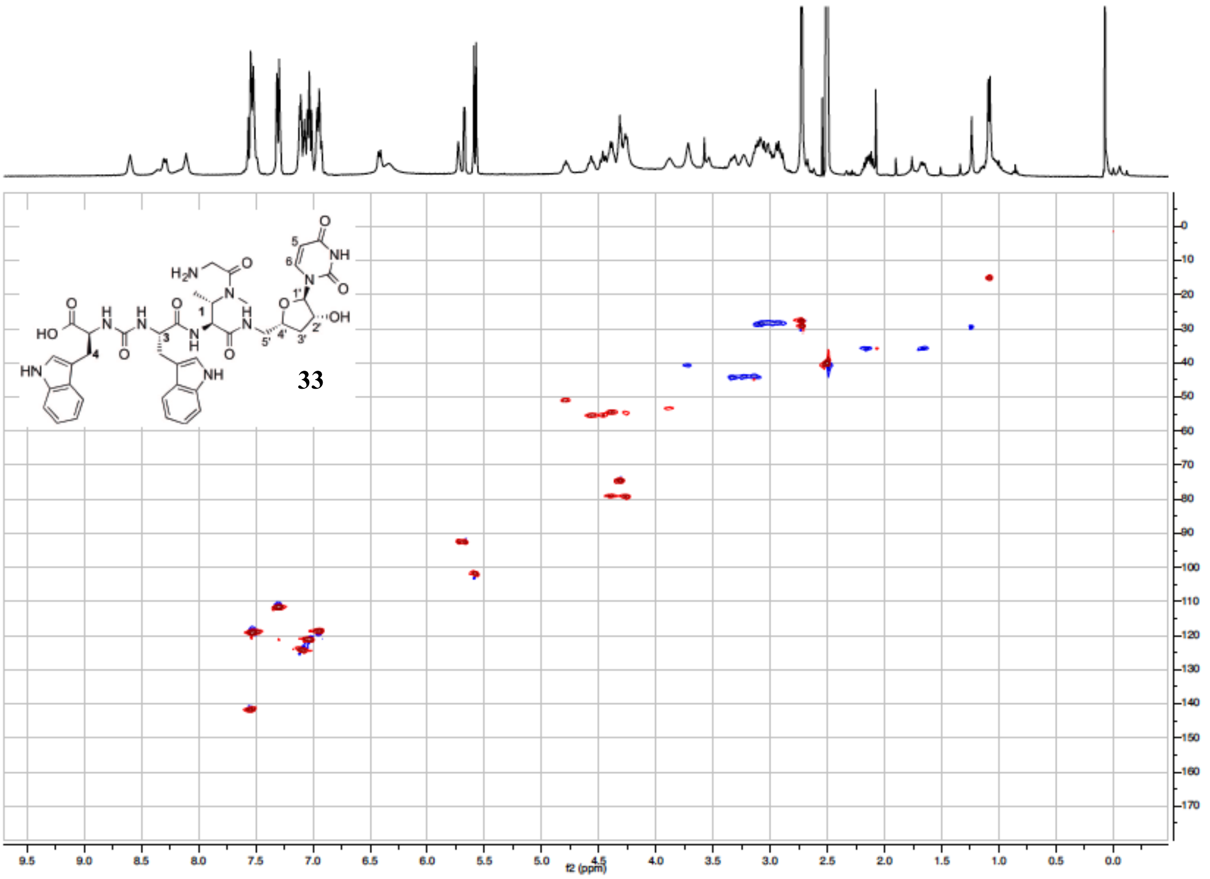

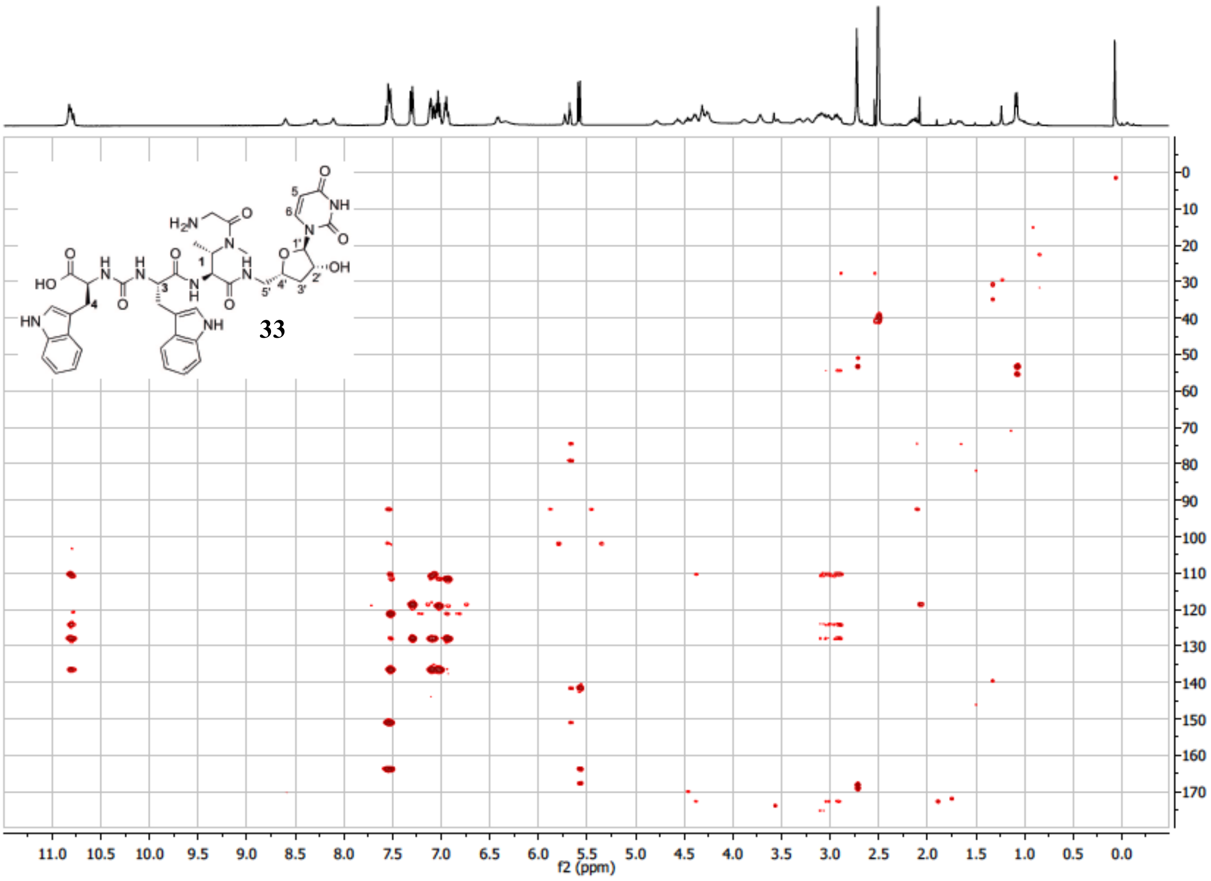

Supplementary Figure 140. HSQC (top) and HMBC (bottom) NMR spectra of compound **33**.

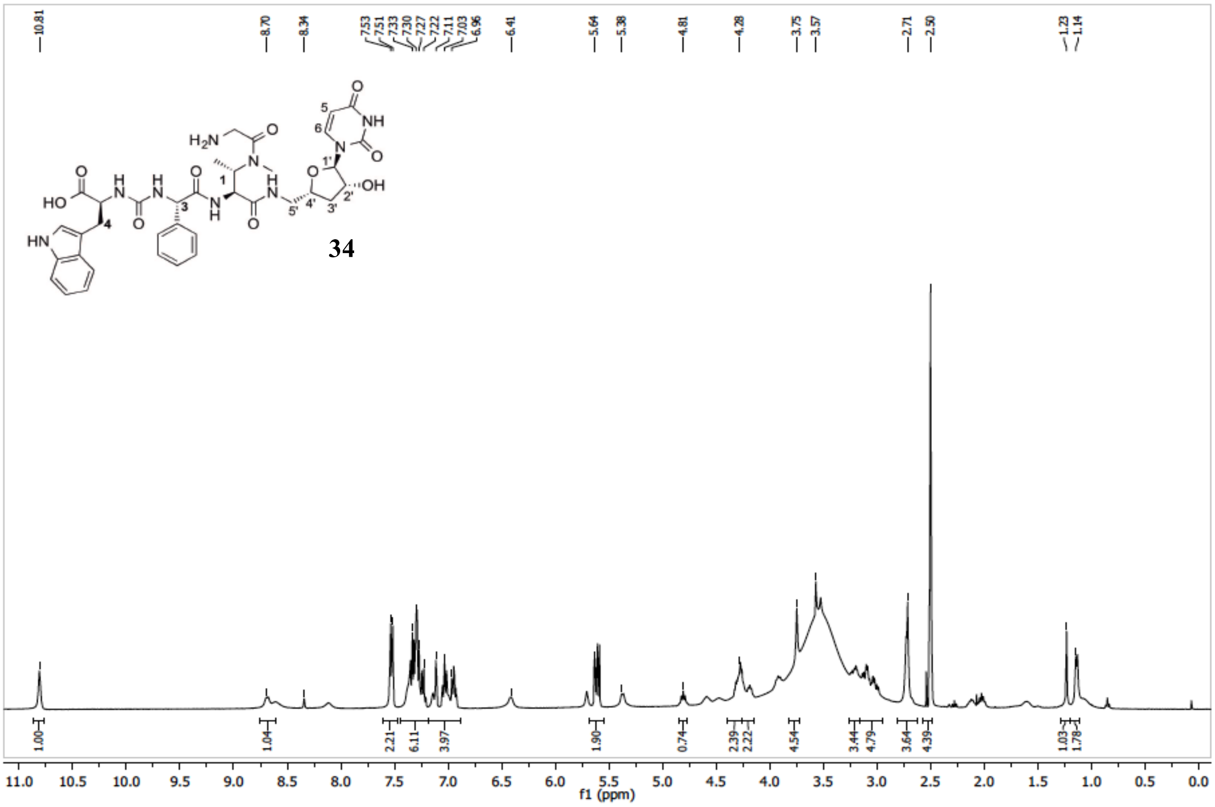

Supplementary Figure 141. ^{1}H NMR spectrum of compound **34**.

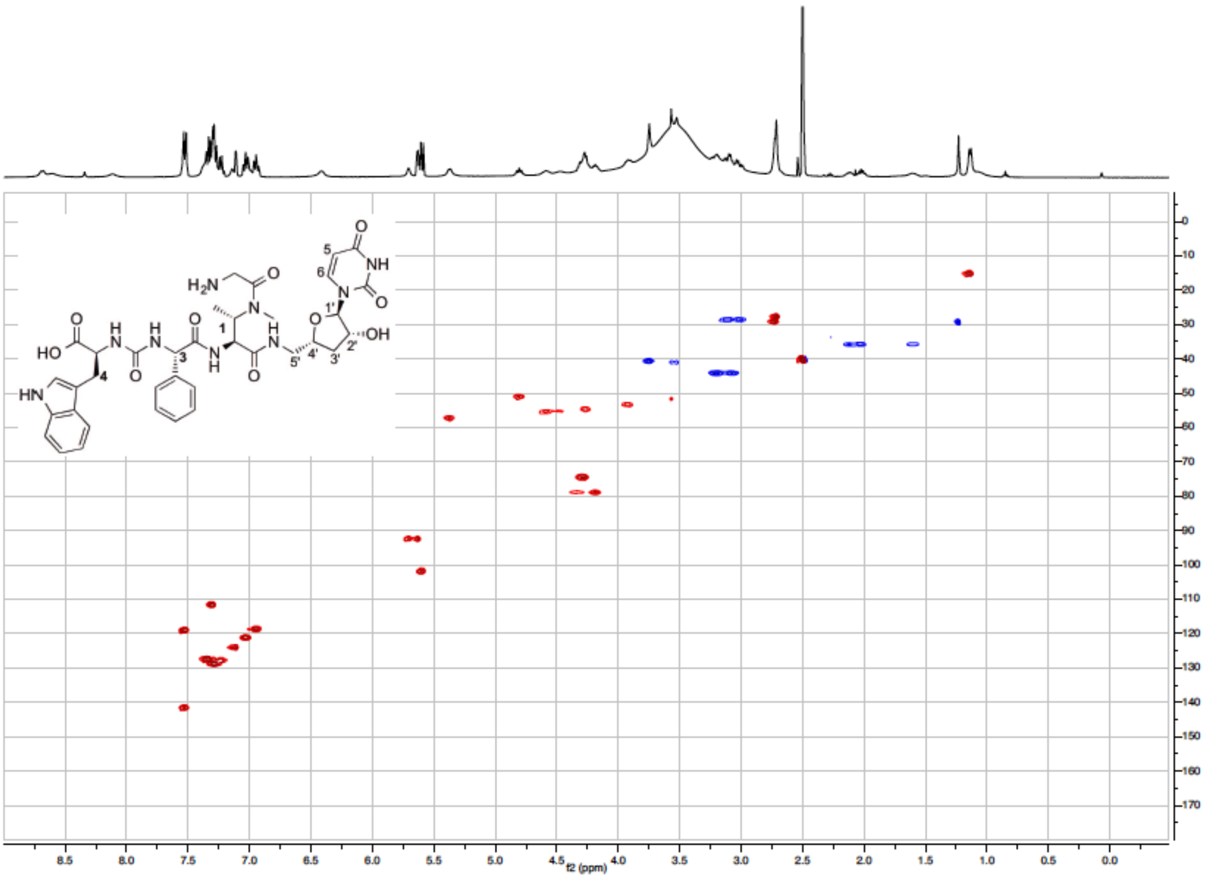

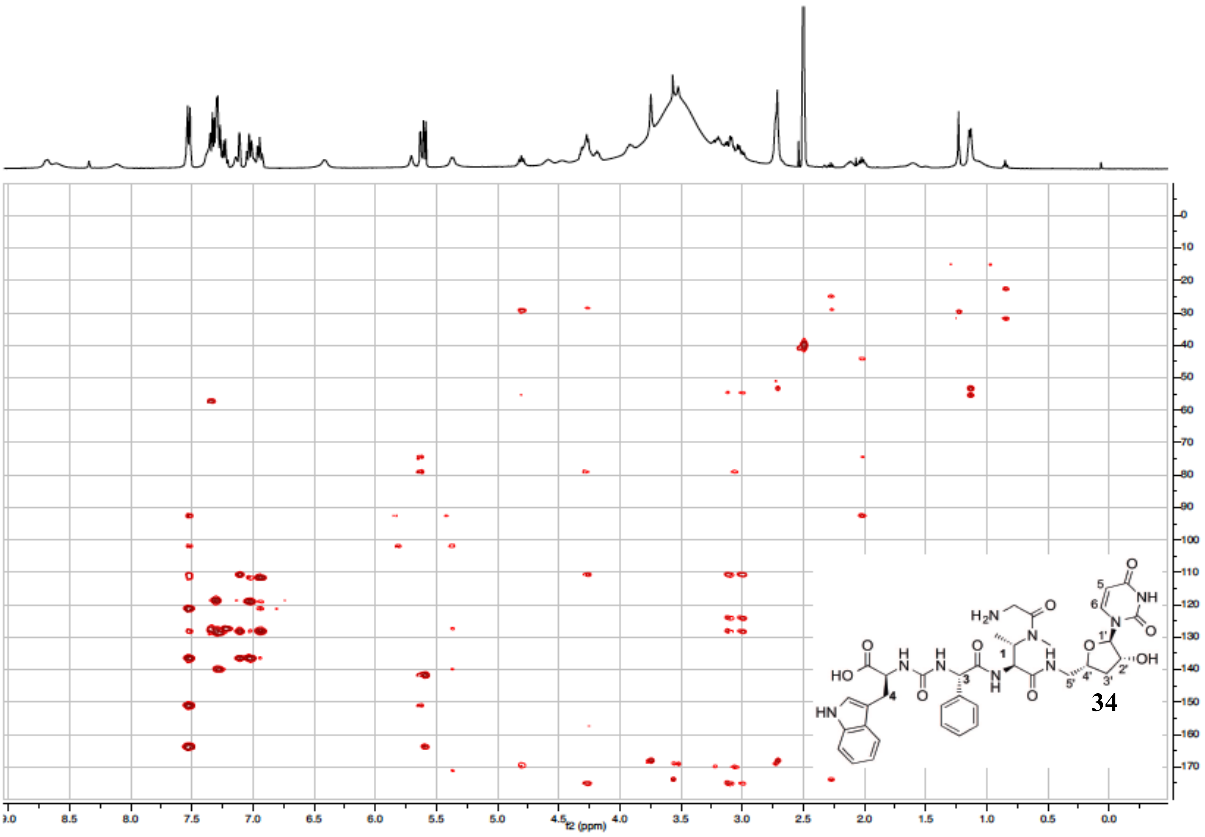

Supplementary Figure 142. HSQC (top) and HMBC (bottom) NMR spectra of compound **34**.

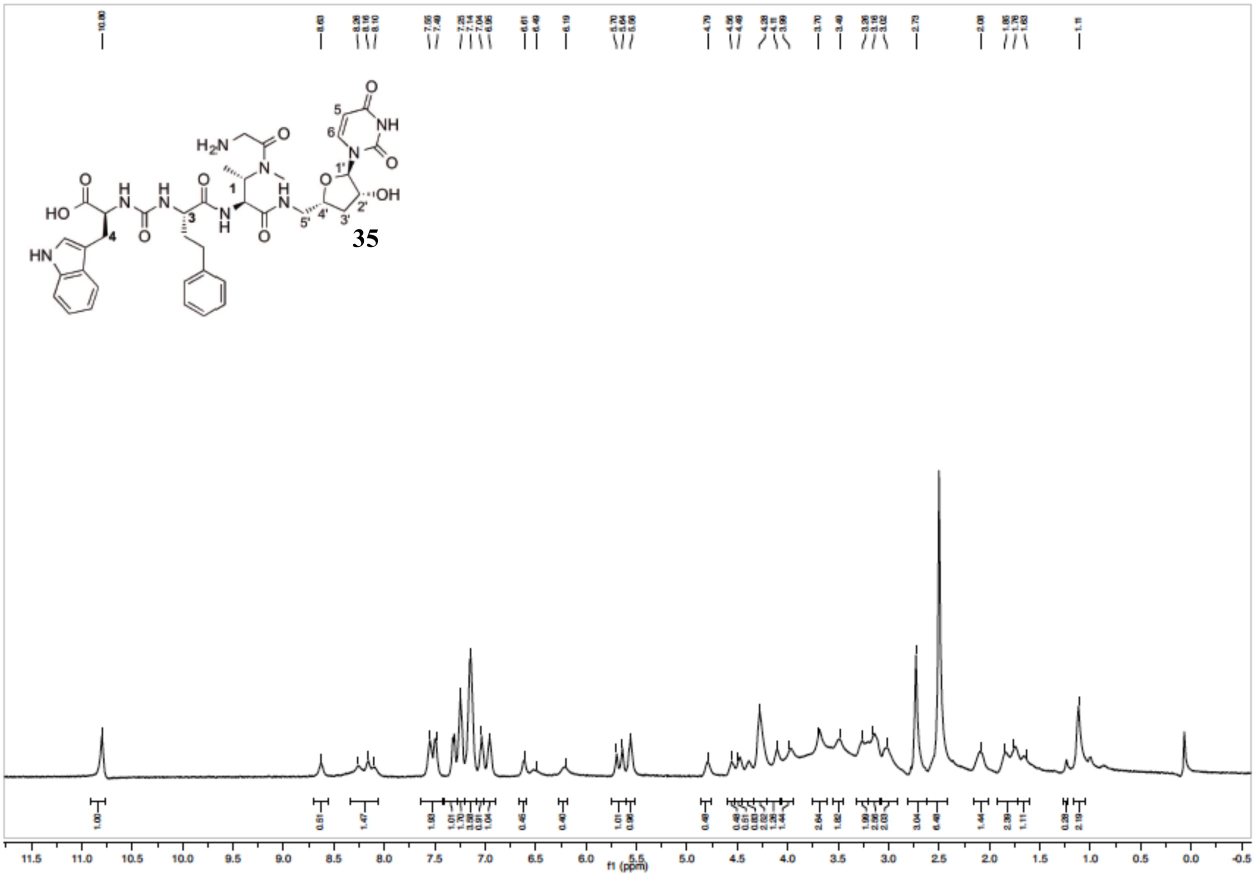

Supplementary Figure 143. ^{1}H NMR spectrum of compound **35**.

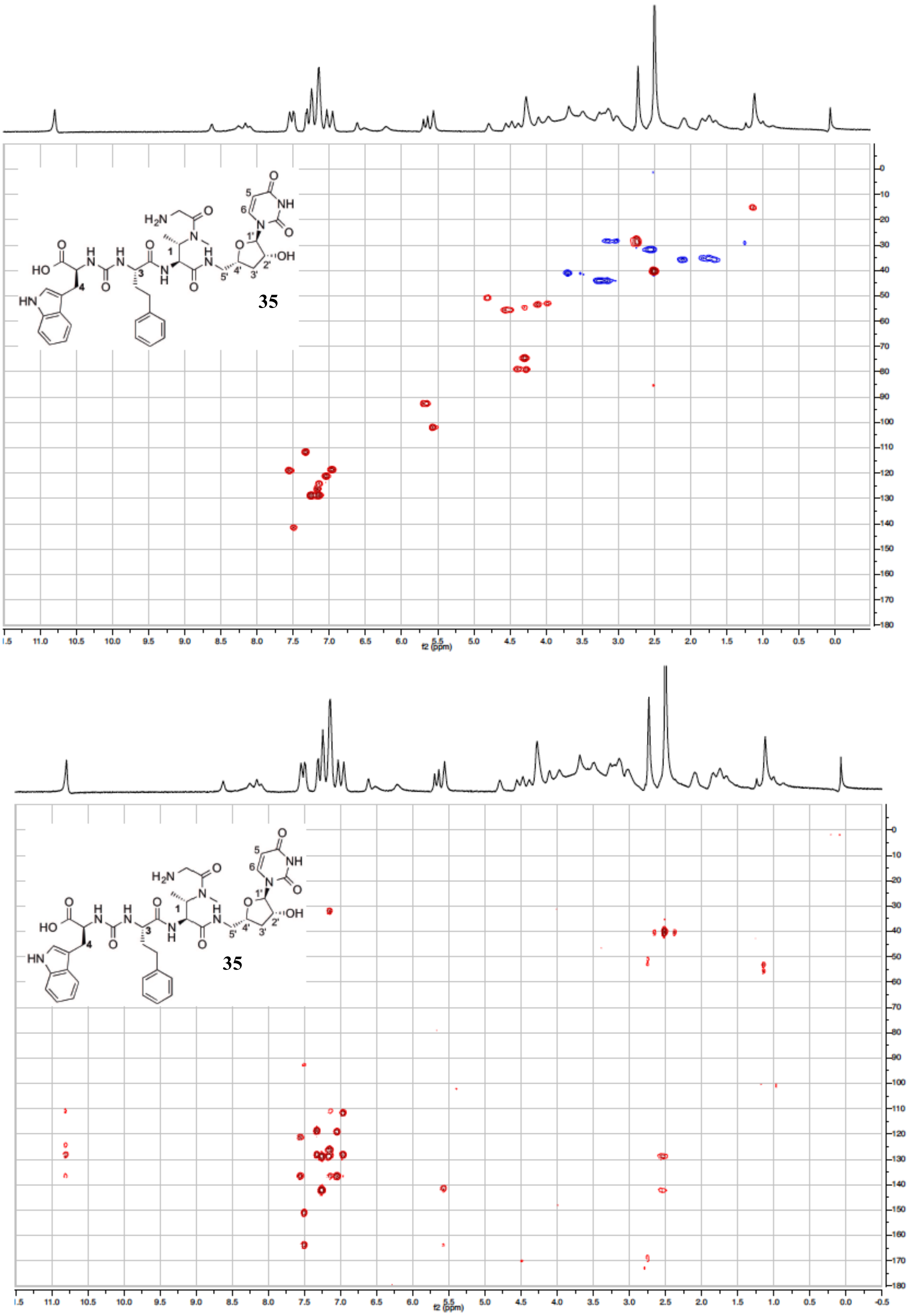

Supplementary Figure 144. HSQC (top) and HMBC (bottom) NMR spectra of compound **35**.

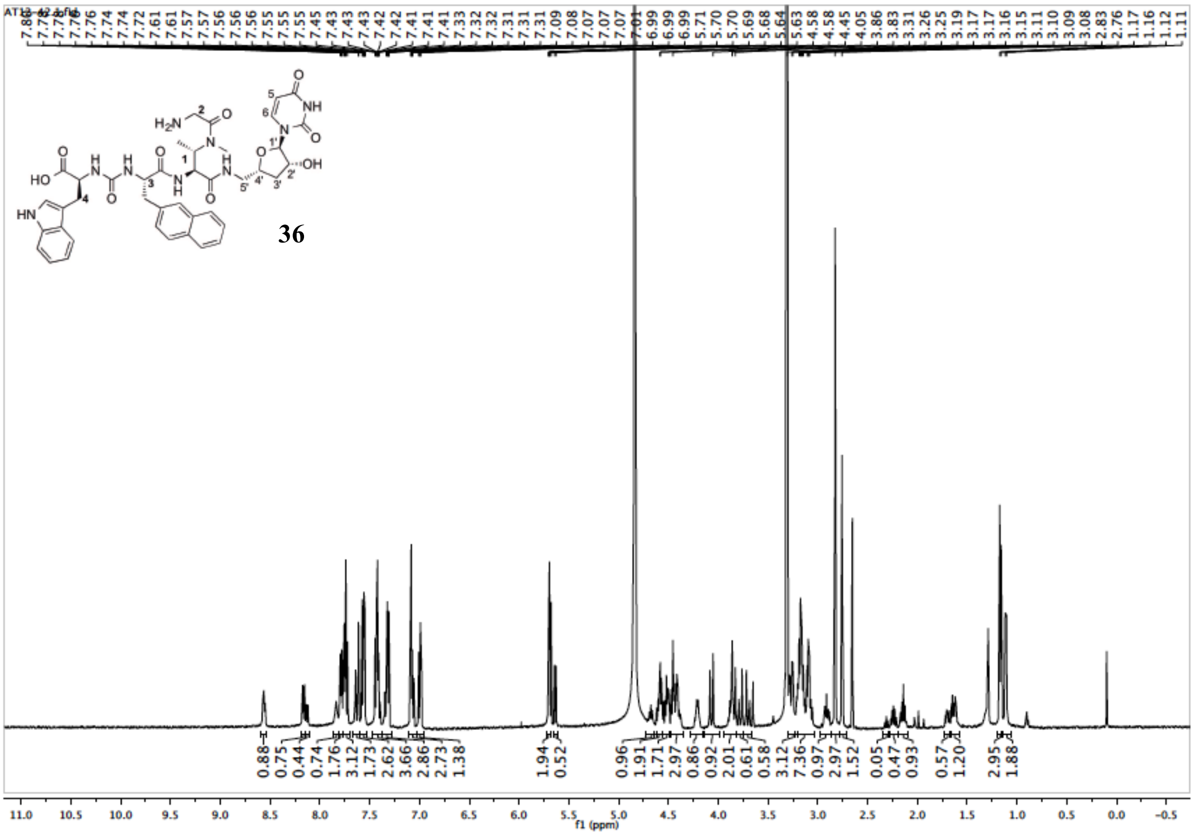

Supplementary Figure 145. ^{1}H NMR spectrum of compound **36**.

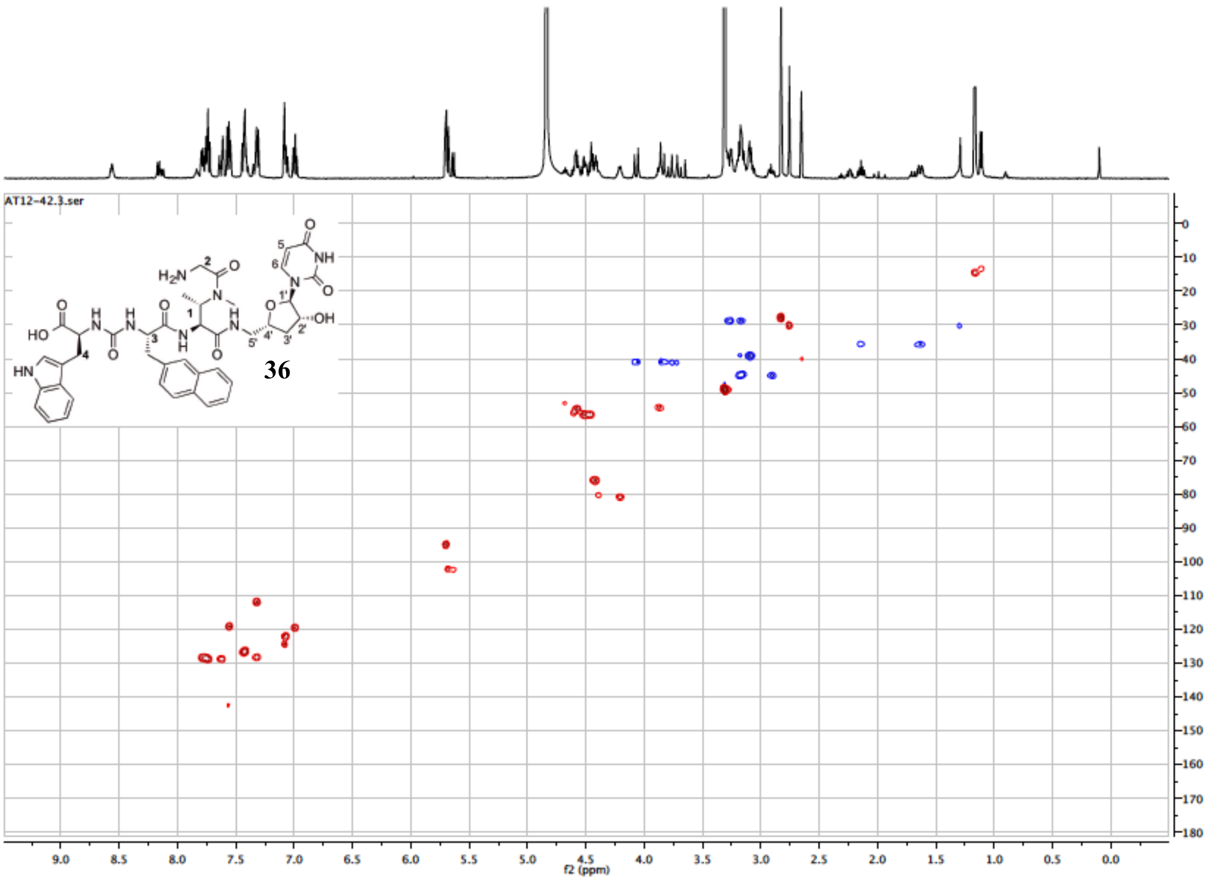

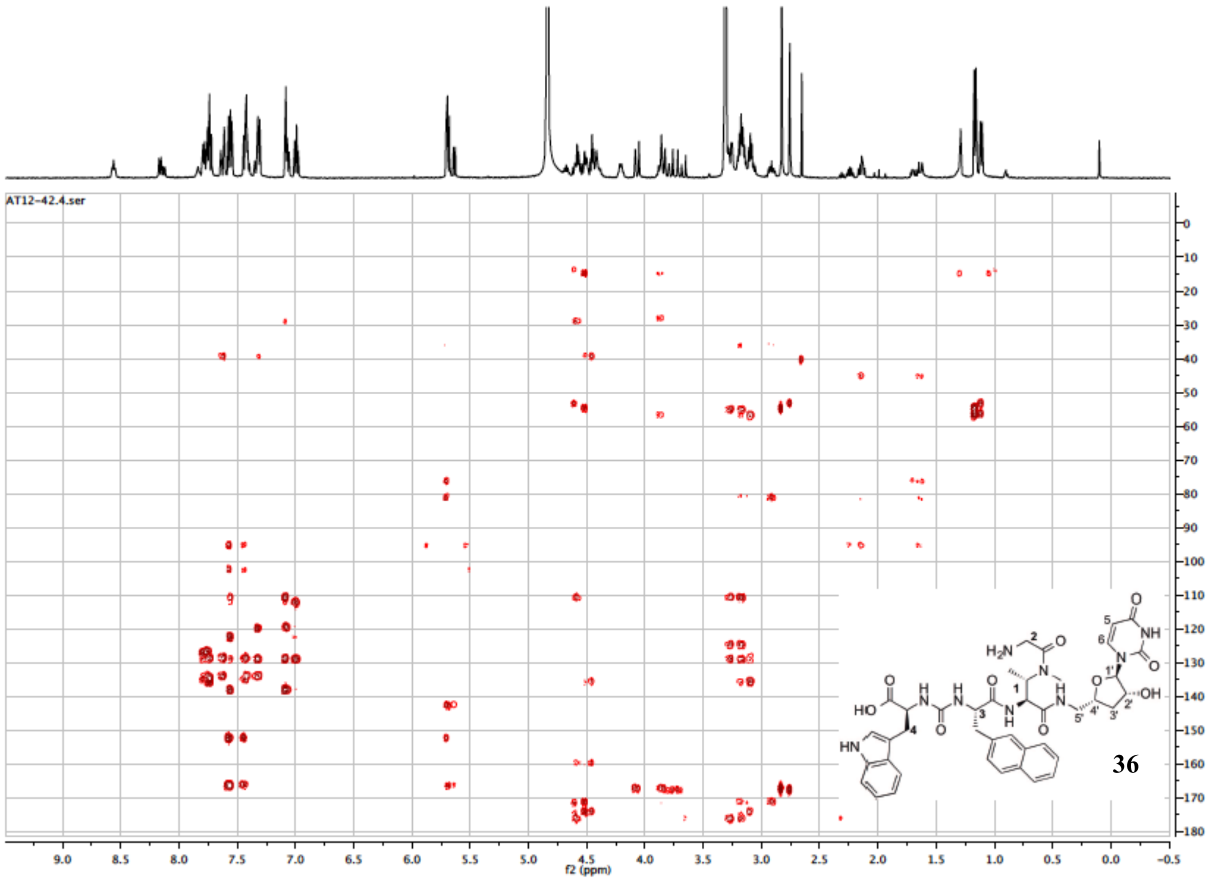

Supplementary Figure 146. HSQC (top) and HMBC (bottom) NMR spectra of compound **36**.

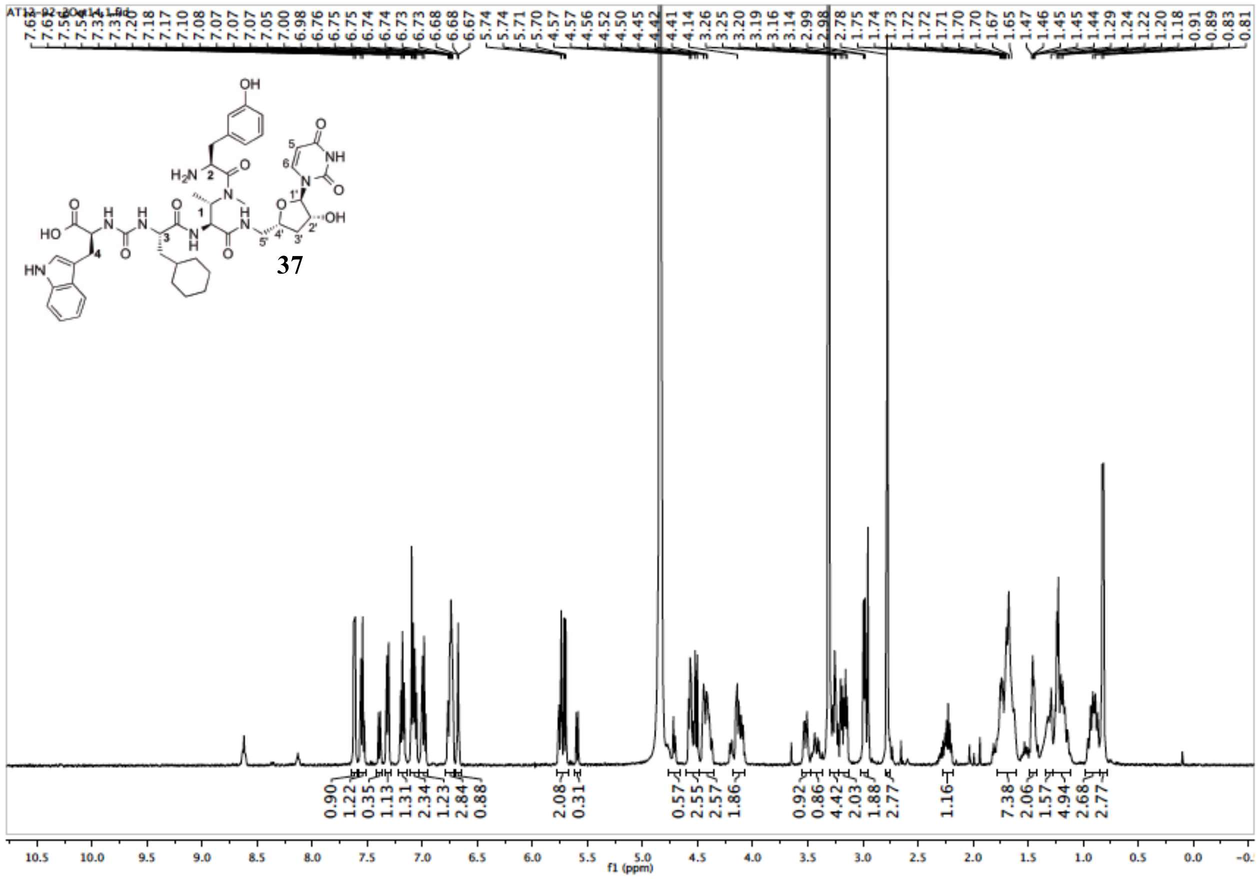

Supplementary Figure 147. ^{1}H NMR spectrum of compound **37**.

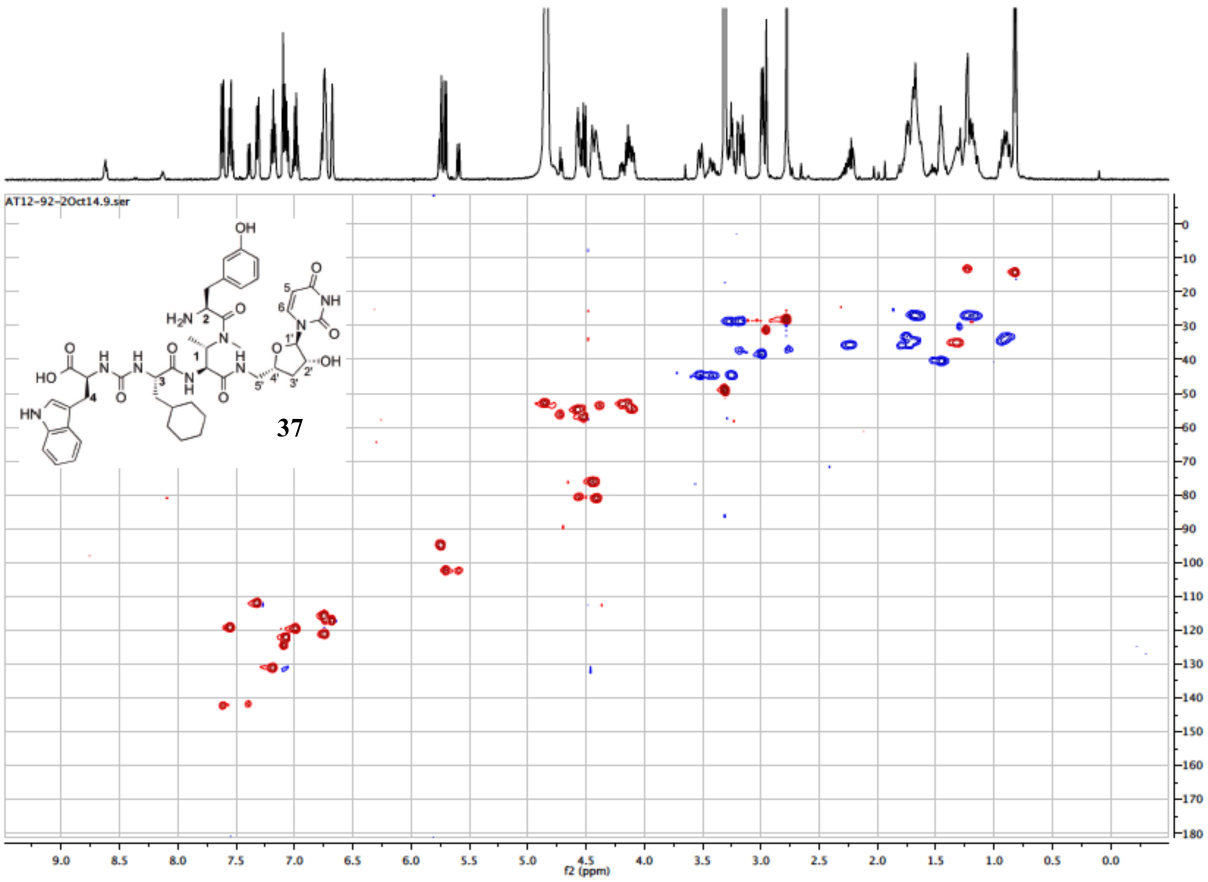

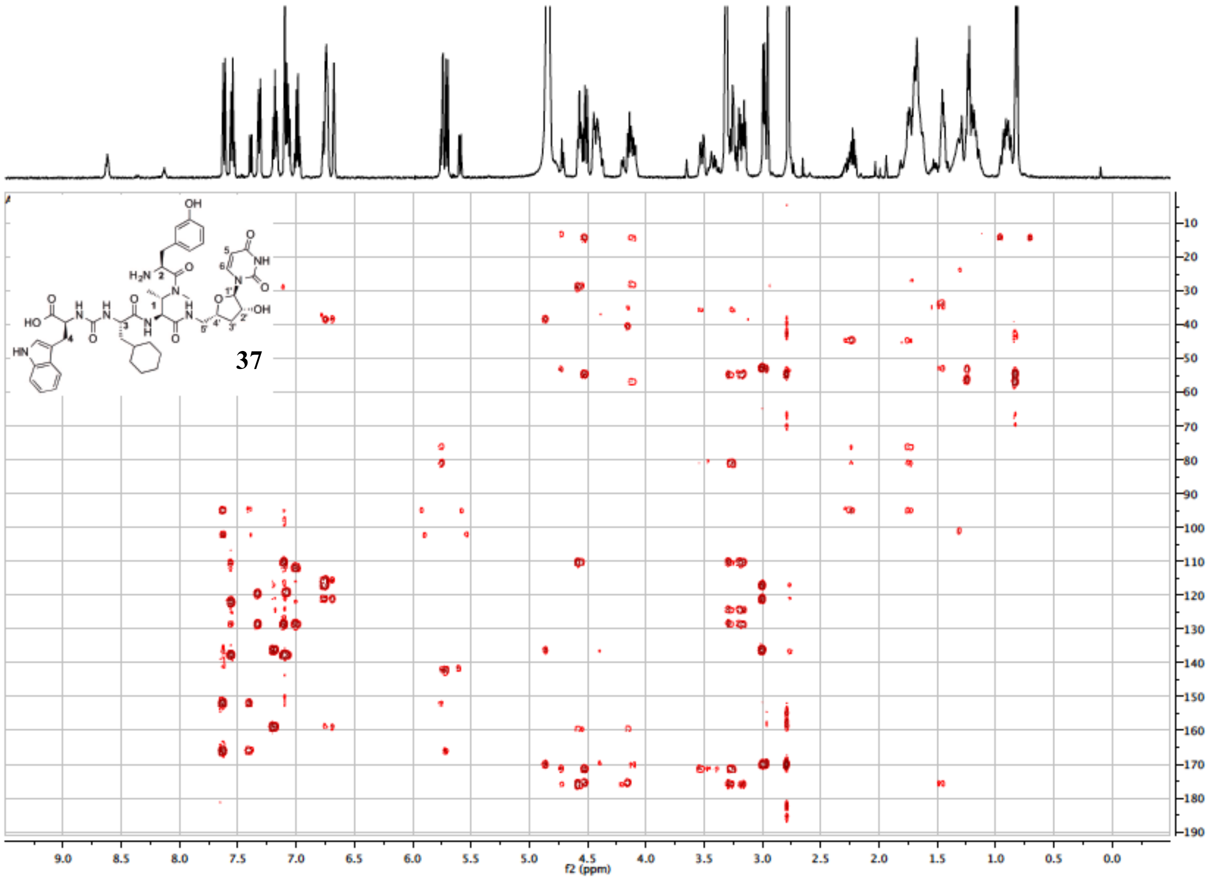

Supplementary Figure 148. HSQC (top) and HMBC (bottom) NMR spectra of compound **37**.

SUPPLEMENTARY TABLES

Growth Inhibition Screen of Dihydrosansanmycin Analogues 4-17 and 21-37 Against a Panel of Gram Positive and Gram Negative Bacterial Strains

Supplementary Table 1. Activity of dihydrosansanmycin analogues **4-17** against a panel of Gram-positive and Gram-negative bacteria. NB: NA = non-active (up to a concentration of 100 μM).

Bacteria strain	MIC (μM)													
	**4**	**5**	**6**	**7**	**8**	**9**	**10**	**11**	**12**	**13**	**14**	**15**	**16**	**17**
A. baumannii	NA	NA	NA	NA	NA	NA	NA	NA	NA	NA	NA	NA	NA	NA
E. coli	NA	50 μM	NA	NA	NA	NA	NA	NA	NA	NA	NA	NA	NA	NA
P. aeruginosa	25 μM	12.5 μM	25 μM	NA	NA	NA	NA	NA	NA	50 μM	NA	50 μM	50 μM	NA
S. aureus	NA	NA	NA	NA	NA	NA	NA	NA	NA	NA	NA	NA	NA	NA
MRSA	NA	NA	NA	NA	NA	NA	NA	NA	NA	NA	NA	NA	NA	NA
V. cholerae	NA	NA	NA	NA	NA	NA	NA	NA	NA	NA	NA	NA	NA	NA
E. aerogenes	NA	NA	NA	NA	NA	NA	NA	NA	NA	NA	NA	NA	NA	NA
O. anthropi	NA	NA	NA	NA	NA	NA	NA	NA	NA	NA	NA	NA	NA	NA
P. alcalifaciens	NA	NA	NA	NA	NA	NA	NA	NA	NA	NA	NA	NA	NA	NA
B. subtilis	NA	NA	NA	NA	NA	NA	NA	NA	NA	NA	NA	NA	NA	NA
E. faecium	NA	NA	NA	NA	NA	NA	NA	NA	NA	NA	NA	NA	NA	NA
L. ivanovii	NA	NA	NA	NA	NA	NA	NA	NA	NA	NA	NA	NA	NA	NA
S. epidermidis	NA	NA	NA	NA	NA	NA	NA	NA	NA	NA	NA	NA	NA	NA
S. typhimurium	NA	NA	NA	NA	NA	NA	NA	NA	NA	NA	NA	NA	NA	NA
Y. pseudotuberculosi	NA	NA	NA	NA	NA	NA	NA	NA	NA	NA	NA	NA	NA	NA

Supplementary Table 2. Activity of dihydrosansanmycin analogues **21**-**37** against a panel of Gram-positive and Gram-negative bacteria. NB: NA = non-active (up to a concentration of 100 μM).

Bacteria strain	MIC (μM)																
	**21**	**22**	**23**	**24**	**25**	**26**	**27**	**28**	**29**	**30**	**31**	**32**	**33**	**34**	**35**	**36**	**37**
A. baumannii	NA	NA	NA	NA	NA	NA	NA	NA	NA	NA	NA	NA	NA	NA	NA	NA	NA
E. coli	100 μM	50 μM	NA	NA	NA	100 μM	NA	NA	NA	NA	NA	NA	NA	NA	NA	NA	100 μM
P. aeruginosa	25 μM	12.5 μM	NA	100 μM	NA	50 μM	NA	NA	50 μM	NA	25 μM	100 μM	NA	NA	NA	NA	NA
S. aureus	NA	NA	NA	NA	NA	NA	NA	NA	NA	NA	NA	NA	NA	NA	NA	NA	NA
MRSA	NA	NA	NA	NA	NA	NA	NA	NA	NA	NA	NA	NA	NA	NA	NA	NA	50 μM
V. cholerae	NA	NA	NA	NA	NA	NA	NA	NA	NA	NA	NA	NA	NA	NA	NA	NA	NA
E. aerogenes	NA	NA	NA	NA	NA	NA	NA	NA	NA	NA	NA	NA	NA	NA	NA	NA	NA
O. anthropi	NA	NA	NA	NA	NA	NA	NA	NA	NA	NA	NA	NA	NA	NA	NA	NA	NA
P. alcalifaciens	NA	NA	NA	NA	NA	NA	NA	NA	NA	NA	NA	NA	NA	NA	NA	NA	NA
B. subtilis	NA	NA	NA	NA	NA	NA	NA	NA	NA	NA	NA	NA	NA	NA	NA	NA	NA
E. faecium	NA	NA	NA	NA	NA	NA	NA	NA	NA	NA	NA	NA	NA	NA	NA	NA	NA
L. ivanovii	NA	NA	NA	NA	NA	NA	NA	NA	NA	NA	NA	NA	NA	NA	NA	NA	NA
S. epidermidis	NA	NA	NA	NA	NA	NA	NA	NA	NA	NA	NA	NA	NA	NA	NA	NA	NA
S. typhimurium	NA	NA	NA	NA	NA	NA	NA	NA	NA	NA	NA	NA	NA	NA	NA	NA	NA
Y. pseudotuberculosi	NA	NA	NA	NA	NA	NA	NA	NA	NA	NA	NA	NA	NA	NA	NA	NA	NA

Inhibition of MurX activity by dihydrosansanmycin analogues

Supplementary Table 3. Inhibition of MurX by dihydrosansanmycins A-C (**4-6**)

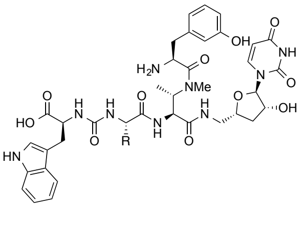

Compound	R	% MurX inhibition at 200 nM
4	CH_2CH_2SMe	68%
5	$CH_2CH(CH_3)_2$	>51%
6	$CH_2CH_2S(O)Me$	68%

Supplementary Table 4. Inhibition of MurX by dihydrosansanmycins **7-17**

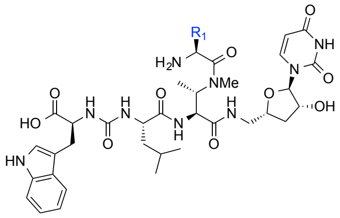

Compound	R_1	% MurX inhibition at 200 nM
7	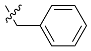	27%
8	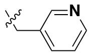	5%
9	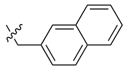	27%
10	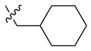	41%
11	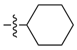	14%
12	H	9%
13	CH_3	50%
14	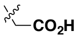	8%
15	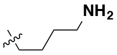	7%
16	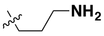	5%
17	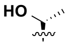	20%

Supplementary Table 5. Inhibition of MurX by dihydrosansanmycins **21-37**

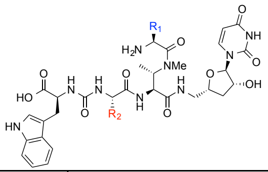

Compound	R_1	R_2	% MurX inhibition at 200 nM
21	H	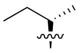	46%
22	H	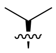	26%
23	H	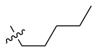	59%
24	H	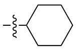	41%
25	H	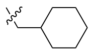	100%
26	H	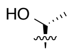	40%
27	H	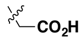	34%
28	H	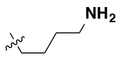	47%
29	H	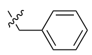	60%
30	H	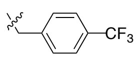	100%
31	H	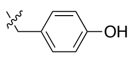	40%
32	H	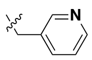	52%
33	H	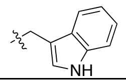	52%
34	H	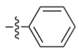	51%
36	H	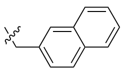	100%
37	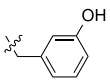	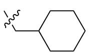	100%

Preliminary *in vitro* DMPK studies: Stability in human and mouse plasma

Supplementary Table 6. Measured concentrations of **25**, **36**, **37** in human and mouse plasma over a 6 hour incubation at 37°C

**Human Plasma**				
**Sampling time**	**Replicate**	**Measured conc. (ng/mL)**		
		**25**	**36**	**37**
2 min	1	826.7	808.7	964.3
	2	818.8	928.1	1092.6
0.5 h	1	943.7	937.9	1066.6
	2	853.3	908.9	1098.7
1 h	1	852.1	899.6	1014.9
	2	898.0	954.7	1058.1
2 h	1	803.9	916.8	1025.1
	2	735.1	890.9	1034.6
4 h	1	841.9	883.3	1024.2
	2	922.5	892.2	1117.3
6 h	1	871.0	893.7	1002.9
	2	904.5	900.1	1014.2
**Mouse Plasma**				
**Sampling time**	**Replicate**	**Measured conc. (ng/mL)**		
		**25**	**36**	**37**
2 min	1	849.7	960.5	948.7
	2	902.2	985.7	859.0
0.5 h	1	931.1	980.2	948.2
	2	867.8	1030.6	996.1
1 h	1	899.8	1043.2	933.2
	2	926.8	1016.3	938.7
2 h	1	871.1	1019.1	996.2
	2	880.9	962.5	958.0
4 h	1	886.1	957.5	918.2
	2	889.4	996.6	895.1
6 h	1	881.5	929.1	878.5
	2	878.0	928.4	905.1

Preliminary *in vitro* DMPK studies: Stability in human and mouse liver microsomes

Supplementary Table 7. Stability in human and mouse liver microsomes

Compound	Species	Average half-life (min)	Intrinsic Clearance (µL/min/mg protein)
**25**	Human	> 247	<7
	Mouse	> 247	<7
**36**	Human	> 247	<7
	Mouse	166	10
**37**	Human	174	10
	Mouse	233	7

SUPPLEMENTARY METHODS

General synthetic experimental

^{1}H NMR spectra were recorded at 300 K unless otherwise specified using a Bruker Avance DPX 300, DPX 400, DPX 500 and DPX 600 NMR spectrometer at a frequency of 300.2, 400.2, 500.2 and 600.2 MHz respectively. ^{1}H NMR chemical shifts are reported in parts per million (ppm) and are referenced to solvent residual signals: $CDCl_3$ δ 7.26, MeOD δ 3.31, acetone-d_6 δ 2.05, DMSO-d_6 δ 2.50 and D_2O δ 4.79. ^{1}H NMR data is reported as chemical shift (δ_H), relative integral, multiplicity (s = singlet, d = doublet, t = triplet, q = quartet, dd = doublet of doublets, ddd = doublet of doublet of doublets, dt = doublet of triplets, td = triplet of doublets, tt = triplet of triplets, qd = quartet of doublets), coupling constant (*J* Hz) and assignment where possible. In the presence of rotamers, ^{1}H and ^{13}C NMR data for both rotamers were reported when the ratio of the rotamers is smaller than 1.5:1. For cases in which the ratio is greater or equal to 1.5, only the major rotamer was reported.

Low resolution mass spectra were recorded on a Finnigan LCQ Deca ion trap mass spectrometer (ESI). High resolution mass spectra were recorded on a Bruker 7T Fourier Transform Ion Cyclotron Resonance Mass Spectrometer (FTICR).

Melting points were recorded using a Stanford Research Systems OptiMelt Automated Melting Point System. Infrared (IR) absorption spectra were recorded on a Bruker ALPHA Spectrometer with Attenuated Total Reflection (ATR) capability, using OPUS 6.5 software. Optical rotations were measured using a Perkin Elmer Model 341 polarimeter and $[\alpha]_D^{25}$ values are reported in 10^{-1} deg cm^2 g^{-1}.

Preparative reverse phase HPLC was performed using a Waters 600 Multisolvent Delivery System and Waters 500 pump with a Waters 2996 photodiode array detector or Waters 490E programmable wavelength detector operating at 254 and 280 nm using a Sunfire Prep C18 OBD, 19 x 50 mm column, operating at a flow rate of 7 mL min^{-1}. Compounds were eluted with 0.1% TFA or formic acid in water (solvent A), and 0.1% TFA or formic acid in CH_3CN (solvent B) using a linear gradient of 0-50% B over 40 min or 0-50% B over 45 min or 50-100% B over 40 min.

LC-MS was performed on a Shimadzu LC-MS 2020 instrument consisting of a LC-M20A pump and a SPD-20A UV/Vis detector coupled to a Shimadzu 2020 mass

spectrometer (ESI) operating in positive mode. Separations were performed on a Waters Sunfire 5 μm, 2.1 x 150 mm column (C18), operating at a flow rate of 0.2 mL min^{-1}. Separations were performed using a mobile phase of 0.1% formic acid in water (Solvent A) and 0.1% formic acid in CH_3CN (Solvent B) and a linear gradient of 0-50% B over 30 min or 50-100% B over 30 min.

Materials

Analytical thin layer chromatography (TLC) was performed on commercially prepared silica plates (Merck Kieselgel 60 0.25 mm F254). Flash column chromatography was performed using 230-400 mesh Kieselgel 60 silica eluting with distilled solvents as described. Ratios of solvents used for TLC and column chromatography are expressed in v/v as specified. Compounds were visualised by UV light at 254 nm or using vanillin or cerium molybdate stain. Commercial materials were used as received unless otherwise noted. DCM and MeOH were distilled from calcium hydride, and THF and diethyl ether were distilled over sodium/benzophenone. *tert*-butanol was dried over activated 3 Å molecular sieves at least 24 h before use. Anhydrous DMF was purchased from Sigma Aldrich.

Synthesis of uridylamine 18

The synthesis of uridylamine was carried out using modifications to the route previously published by Boojamra *et al.*[1]

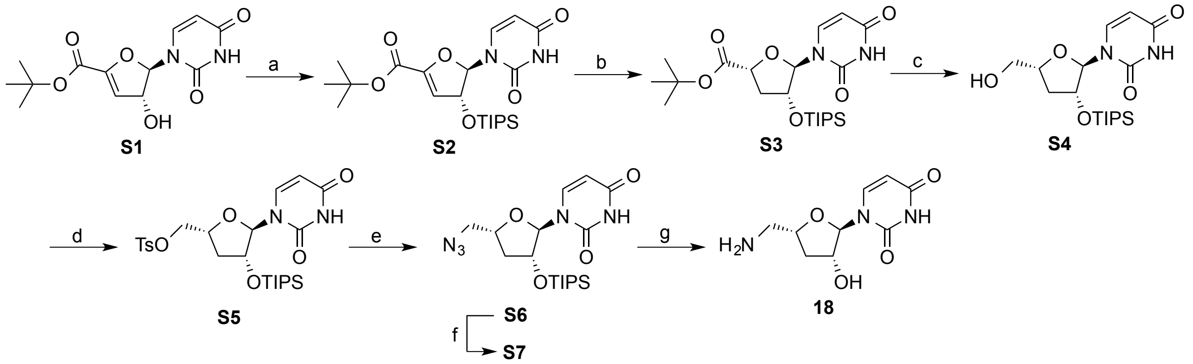

Synthesis of uridylamine **18**. Reagents and conditions: a) TIPSOTf, *i*Pr_2NEt, DMF, rt, 2.5 h, 82%; b) 10% Pd/C, H_2 (1 atm), MeOH, rt, 30 min, 80%; c) (i) TFA: CH_2Cl_2 (9:1 v/v), rt, 15 min; (ii) Isobutylchloroformate, *i*Pr_2NEt, THF, 0 °C to rt, 1.5 h; (iii) $NaBH_4$, H_2O (dropwise), 0 °C, 1 h, 69%; d) Tosyl chloride, pyridine, rt, 18 h, 82%; e) NaN_3, DMF, 75 °C, 4 h, 82%; f) (i) TBAF (1 M in THF), THF, rt, 1 h, (ii) Dowex 50WX8-400, $CaCO_3$, MeOH, rt, 1 h, quant.; g) 1,3-propanedithiol, Et_3N, MeOH, rt, 16 h, 68%.

***tert*-butyl (4*R*,5*R*)-5-(2,4-dioxo-3,4-dihydropyrimidin-1(2*H*)-yl)-4-((triisopropylsilyl)oxy)-4,5-dihydrofuran-2-carboxylate (S2)**

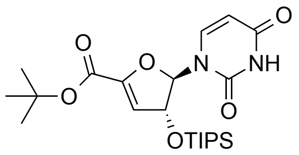

To a solution of ester **S1** (3.64 g, 12.3 mmol) and *N*,*N*-diisopropylethylamine (2.1 mL, 12.3 mmol) in DMF (37 mL) was added triisopropylsilyl trifluoromethanesulfonate (4.9 mL, 17.6 mmol) and the reaction was stirred at rt for 2.5 h. The reaction was quenched with saturated aqueous Na_2CO_3 (40 mL) and partitioned between Et_2O (200 mL) and H_2O (40 mL). The organic layer was washed with 0.2 M HCl (50 mL), H_2O (5× 50 mL), brine (50 mL) and dried over anhydrous Na_2SO_4. The organic layer was concentrated *in vacuo* to give a crude residue that was purified by column chromatography (2:1 v/v Hexane: EtOAc) to afford ester **S2** as a white foam (4.57 g, 82%).

$[\alpha]_D^{25}$ = -182° (c = 0.27 in CH_2Cl_2). **^{1}H NMR** (500 MHz, $CDCl_3$): δ 8.70 (s, 1H, NH), 7.03 (d, *J* = 8.1 Hz, 1H, H-6), 6.27 (d, *J* = 3.2 Hz, 1H, H-3'), 5.96 (d, *J* = 2.6 Hz, 1H, H-1'), 5.77 (d, *J* = 8.1 Hz, 1H, H-5), 5.23 (dd, *J* = 3.3, 2.6 Hz, 1H, H-2'), 1.54 (s, 9H, $CO_2{}^tBu$), 1.12-1.07 (m, 3H, $[CH(CH_3)_2]_3Si$), 1.06-1.02 (m, 18H, $[CH(CH_3)_2]_3Si$). **^{13}C NMR** (126 MHz, $CDCl_3$): δ 162.5 (C=O), 158.2, 151.3, 149.3, 140.0, 111.2, 103.7, 95.1, 83.5, 79.7, 28.0, 17.8, 17.8, 12.0. **IR (ATR):** 2944, 2868, 1725, 1698 cm^{-1}. **LRMS** [M+H^+] 453.0. **HRMS (ESI m/z)** [M+Na^+] calcd. for $C_{22}H_{36}N_2O_6SiNa$, 475.2240; found, 475.2238.

***tert*-butyl (2*R*,4*R*,5*R*)-5-(2,4-dioxo-3,4-dihydropyrimidin-1(2*H*)-yl)-4-((triisopropylsilyl)oxy)tetrahydrofuran-2-carboxylate (S3)**

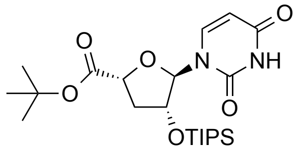

To a solution of ester **S2** (568 mg, 1.25 mmol) in MeOH (21 mL) was added 10% Pd/C (86 mg) and the reaction was degassed by passing through 3 cycles of alternating high vacuum and nitrogen. The reaction mixture was allowed to stir under an atmosphere of hydrogen for 30 min. At this point, the reaction was filtered over Celite®, and the filter cake was washed thoroughly with MeOH (100 mL). The

solvent was removed *in vacuo* to give a crude residue that was purified by column chromatography (1:1 v/v Hexane: EtOAc) to afford ester **S3** as a viscous colourless oil (480 mg, 80%).

$[\alpha]_D^{25}$ = -14° (c = 0.41 in CH_2Cl_2). **^{1}H NMR** (500 MHz, $CDCl_3$): δ 7.18 (d, *J* = 8.1 Hz, 1H, H-6), 5.72-5.68 (m, 2H, H-1' + H-5), 4.78-4.74 (m, 2H, H-2' + H-4'), 2.57 (ddd, *J* = 13.2, 8.7, 6.2 Hz, 1H, H-3'), 2.24 (ddd, *J* = 13.3, 5.2, 3.8 Hz, 1H, H-3'), 1.49 (s, 9H, $CO_2{}^tBu$), 1.15-1.05 (m, 3H, [*CH*(CH_3)$_2$]$_3$Si), 1.05-1.01 (m, 18H, [CH*(CH_3)$_2$*]$_3$Si). **^{13}C NMR** (126 MHz, $CDCl_3$): δ 170.0 (C=O), 163.0 (C=O), 149.7 (C=O), 141.4, 102.2, 97.2, 82.2, 78.3, 75.1, 37.8, 28.0, 17.8, 17.8, 11.9. **IR (ATR):** 2943, 2867, 1688 cm^{-1}. **LRMS** [M+H$^+$] 455.3. **HRMS (ESI m/z)** [M+Na$^+$] calcd. for $C_{22}H_{38}N_2O_6SiNa$, 477.2397; found, 477.2400.

1-((2*R*,3*R*,5*R*)-5-(hydroxymethyl)-3-((triisopropylsilyl)oxy)tetrahydrofuran-2-yl)pyrimidine-2,4(1*H*,3*H*)-dione (S4)

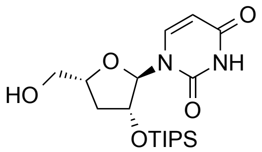

Ester **S3** (456 mg, 1.0 mmol) was dissolved in 9:1 v/v TFA: CH_2Cl_2 (2.2 mL) and the reaction was stirred at rt for 15 min. The solvent was removed *in vacuo* to give a residue that was subsequently dissolved in THF (13 mL) and cooled to 0 °C prior to the addition of isobutyl chloroformate (390 μL, 3 mmol) and *N*,*N*-diisopropylethylamine (260 μL, 1.5 mmol). The reaction mixture was allowed to warm up to rt and was stirred for a further 1.5 h before being cooled to 0 °C. Sodium borohydride (265 mg, 7 mmol) was added, followed by dropwise addition of water (2.3 mL) over 20 min to dissolve the sodium borohydride. The reaction was allowed to stir at 0 °C for 1 h before being concentrated *in vacuo*. The residue was partitioned between EtOAc (40 mL) and water (10 mL). The organic phase was separated, washed with 1 M HCl (10 mL), brine (10 mL) and dried over anhydrous $MgSO_4$. The solvent was removed *in vacuo* to give a crude residue that was purified by column chromatography (4:1 v/v EtOAc: Hexane → EtOAc) to afford alcohol **S4** as a white foam (265 mg, 69%).

$[\alpha]_D^{25}$ = -11° (c = 0.44 in CH_2Cl_2). **^{1}H NMR** (400 MHz, $CDCl_3$): δ 8.94 (s, 1H), 7.25 (d, *J* = 8.1 Hz, 1H, H-6), 5.72 (d, *J* = 8.1 Hz, 1H, H-5), 5.69 (d, *J* = 2.1 Hz, 1H, H-1'), 4.73 (ddd, *J* = 5.7, 3.5, 2.1 Hz, 1H, H-2'), 4.61 (*app.* dq, *J* = 9.0, 4.7 Hz, 1H, H-4'), 3.80-3.72 (m, 2H, H-5'), 2.60 (s, 1H, OH), 2.27 (ddd, *J* = 13.9, 8.3, 5.8 Hz, 1H, H-3'), 1.96 (ddd, *J* = 13.6, 5.0, 3.4 Hz, 1H, H-3'), 1.20-1.08 (m, 3H, $[CH(CH_3)_2]_3Si$), 1.06 (d, *J* = 7.0 Hz, 18H, $[CH(CH_3)_2]_3Si$). **^{13}C NMR** (101 MHz, $CDCl_3$): δ 163.2 (C=O), 150.0 (C=O), 140.2, 102.1, 95.3, 82.4, 76.1, 65.1, 34.8, 17.9, 17.8, 12.0. **IR (ATR):** 2943, 2866, 1686 cm^{-1}. **LRMS** [*M*+Na^+] 407.0. **HRMS (ESI m/z)** [*M*+Na^+] calcd. for $C_{18}H_{32}N_2O_5SiNa$, 407.1978; found, 407.1973.

((2*R*,4*R*,5*R*)-5-(2,4-dioxo-3,4-dihydropyrimidin-1(2*H*)-yl)-4-((triisopropylsilyl)oxy)tetrahydrofuran-2-yl)methyl 4-methylbenzenesulfonate (S5)

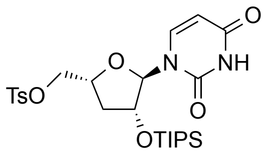

Alcohol **S4** (1.53 g, 3.97 mmol) and *p*-toluenesulfonyl chloride (3.70 g, 19.3 mmol) were dissolved in pyridine (33 mL) and the reaction was allowed to stir at rt for 18 h. At this point, the reaction was concentrated *in vacuo* to afford a residue that was dissolved in EtOAc (200 mL), washed with water (50 mL), 1 M HCl (50 mL), brine (50 mL) and dried over anhydrous $MgSO_4$. The solvent was removed *in vacuo* to give a crude residue that was purified by column chromatography (1:1 v/v Hexane: EtOAc) to afford tosylate **S5** as a colourless oil (1.73 g, 82%).

$[\alpha]_D^{25}$ = -7.3° (c = 0.31 in CH_2Cl_2). **^{1}H NMR** (500 MHz, $CDCl_3$): δ 8.86 (s, 1H, NH), 7.80 (*app.* d, *J* = 8.0 Hz, 2H, Ar-H), 7.34 (d, *J* = 8.0 Hz, 2H, Ar-H), 7.13 (d, *J* = 8.1 Hz, 1H, H-6), 5.70 (d, *J* = 8.1 Hz, 1H, H-5), 5.54 (d, *J* = 2.0 Hz, 1H, H-1'), 4.75-4.67 (m, 1H, H-4'), 4.65 (*app.* dt, *J* = 5.1, 2.0 Hz, 1H, H-2'), 4.21 (dd, *J* = 10.4, 7.0 Hz, 1H, H-5'), 4.12 (dd, *J* = 10.4, 4.5 Hz, 1H, H-5'), 2.45 (s, 3H, CH_3), 2.22 (ddd, *J* = 13.7, 8.3, 5.4 Hz, 1H, H-3'), 1.88 (*app.* dt, *J* = 13.7, 3.6 Hz, 1H, H-3'), 1.15-1.02 (m, 3H, $[CH(CH_3)_2]_3Si$), 1.00 (d, *J* = 6.7 Hz, 18H, $[CH(CH_3)_2]_3Si$). **^{13}C NMR** (126 MHz, $CDCl_3$): δ 163.0, 149.8, 145.0, 140.0, 132.7, 129.9, 128.0, 102.1, 95.7, 79.0, 75.8, 71.5, 35.2, 21.7, 17.9, 17.8, 11.9. **IR (ATR):** 2944, 2866, 1686 cm^{-1}. **LRMS**

[M+Na$^+$] 561.0. **HRMS (ESI m/z)** [M+Na$^+$] calcd. for $C_{25}H_{38}N_2O_7SSiNa$, 561.2067; found, 561.2061.

1-((2*R*,3*R*,5*R*)-5-(azidomethyl)-3-((triisopropylsilyl)oxy)tetrahydrofuran-2-yl)pyrimidine-2,4(1*H*,3*H*)-dione (S6)

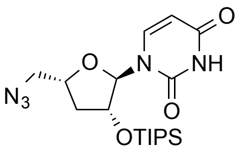

Tosylate **S5** (1.65 g, 3.07 mmol) was dissolved in DMF (34 mL). Sodium azide (2.0 g, 30.7 mmol) was added and the reaction was heated to 75 ^{o}C for 4 h. The reaction was diluted with EtOAc (200 mL), washed with water (5× 40 mL), brine (40 mL) and dried over anhydrous $MgSO_4$. The solvent was removed *in vacuo* to give a crude residue which was purified by column chromatography (1:1 v/v EtOAc: Hexane) to afford azide **S6** as a colourless oil (1.09 g, 87%).

$[\alpha]_D^{25}$ = -20° (c = 0.32 in CH_2Cl_2). **^{1}H NMR** (400 MHz, $CDCl_3$): δ 9.01 (s, 1H, NH), 7.21 (d, J = 8.1 Hz, 1H, H-6), 5.72 (d, J = 8.1 Hz, 1H, H-5), 5.66 (d, J = 2.1 Hz, 1H, H-1'), 4.75 (ddd, J = 5.7, 3.6, 2.1 Hz, 1H, H-2'), 4.62 (tt, J = 7.4, 4.7 Hz, 1H, H-4'), 3.60 (dd, J = 12.7, 7.1 Hz, 1H, H-5'), 3.39 (dd, J = 12.7, 4.6 Hz, 1H, H-5'), 2.28 (ddd, J = 13.5, 7.9, 5.7 Hz, 1H, H-3'), 1.93 (ddd, J = 13.5, 4.9, 3.5 Hz, 1H, H-3'), 1.19-1.08 (m, 3H, [$CH(CH_3)_2]_3$Si), 1.06 (d, J = 6.7 Hz, 18H, [$CH(CH_3)_2]_3$Si). **^{13}C NMR** (101 MHz, $CDCl_3$): δ 163.2 (C=O), 149.9 (C=O), 140.4, 102.1, 96.1, 80.6, 76.1, 55.1, 36.3, 17.9, 17.9, 12.0. **IR (ATR):** 2944, 2867, 2102, 1686 cm^{-1}. **LRMS** [M+H$^+$] 410.0. **HRMS (APCI m/z)** [M+Na$^+$] calcd. for $C_{18}H_{31}N_5O_4SiNa$, 432.2043; found, 432.2038.

1-((2*R*,3*R*,5*R*)-5-(azidomethyl)-3-hydroxytetrahydrofuran-2-yl)pyrimidine-2,4(1*H*,3*H*)-dione (S7)

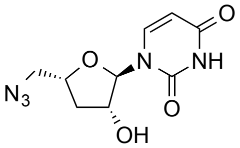

A 1 M solution of TBAF in THF (280 μL, 0.28 mmol, 1.15 eq.) was added dropwise to a solution of azide **S6** (0.10 g, 0.24 mmol, 1 eq.) in THF (1.8 mL) and allowed to stir for 1 h at rt. The reaction mixture was then diluted with MeOH (2.8 mL) and

stirred with calcium carbonate (0.39 g) and Dowex 50WX8-400 (1.2 g) for a further 1 h at rt. The reaction mixture was then filtered over Celite ® and concentrated *in vacuo* to afford the desired azide **S7** as a colourless oil (53.5 mg, 88%) which was used without further purification.

$[\alpha]_D^{25}$ = +25.8° (c = 0.21 in CH_2Cl_2). **^{1}H NMR** (300 MHz, $CDCl_3$): δ 10.53 (*br* s, 1H, NH), 7.35 (d, *J* = 7.5 Hz, 1H, H-6), 5.81 (m, 1H, H-5), 5.72 (d, *J* = 7.5 Hz, 1H, H-1'), 4.77–4.74 (m, 2H, H-2' + H-4'), 3.64 (m, 1H, H-5'), 3.42 (m, 1H, H-5'), 2.39 (m, 1H, H-3'), 1.99 (m, 1H, H-3'). **IR (ATR):** 3056, 2101, 1682, 1461 cm^{-1}. **LRMS** [M+H^+] 253.1. These data are in agreement with those reported by Boojamra *et al.*[1]

1-((2*R*,3*R*,5*R*)-5-(aminomethyl)-3-hydroxytetrahydrofuran-2-yl)pyrimidine-2,4(1*H*,3*H*)-dione (18)

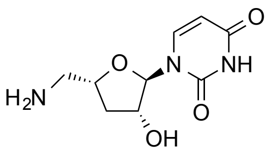

To a solution of azide **S7** (50.0 mg, 198 µmol, 1 eq.) in MeOH (1.0 mL) was added 1,3-propanedithiol (200 µL, 2.0 mmol, 10 eq.) and Et_3N (210 µL, 1.5 mmol, 7.6 eq.). The reaction was allowed to stir for 16 h at rt, before the solvent was removed *in vacuo*. The resulting residue was redissolved in H_2O (45 mL) and washed with CH_2Cl_2 (10x 6 mL). The aqueous layer was lyophilised to afford the desired amine **18** as a white fluffy solid (46.4 mg, 0.134 mmol, 68%), which was used without further purification.

$[\alpha]_D^{25}$ = -1.6° (c = 0.25 in CH_3OH). **^{1}H NMR** (500 MHz, CD_3OD): δ 7.56 (d, *J* = 8.0 Hz, 1H, H-6), 5.75 (d, *J* = 2.2 Hz, 1H, H-1'), 5.69 (d, *J* = 8.0 Hz, 1H, H-5), 4.68-4.48 (m, 1H, H-4'), 4.42 (ddd, *J* = 6.1, 3.6, 2.2 Hz, 1H, H-2'), 2.91 (dd, *J* = 13.6, 3.7 Hz, 1H, H-5'), 2.83 (dd, *J* = 13.5, 5.9 Hz, 1H, H-5'), 2.34 (ddd, *J* = 14.1, 8.1, 6.2 Hz, 1H, H-3'), 1.81 (ddd, *J* = 13.7, 4.8, 3.6 Hz, 1H, H-3'). **IR (ATR):** 3375, 1684, 1629 cm^{-1}. **LRMS** [M+H^+] 228.0. These data are in agreement with those previously reported by Boojamra *et al.*[1]

Synthesis of DABA fragments S8 and 20

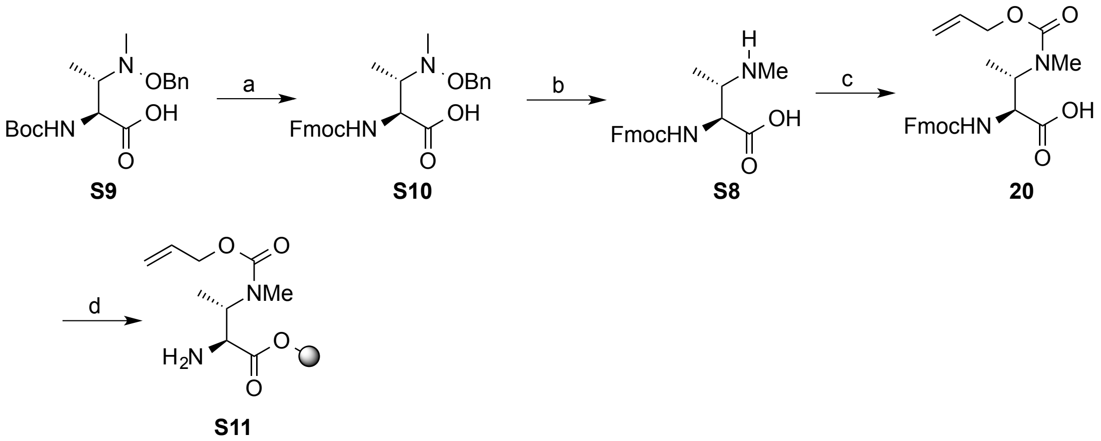

Synthesis of DABA fragments **S8** and **20**. Reagents and conditions: a) i. TFA: CH_2Cl_2 (1:1 v/v), rt, 30 min, ii. Fmoc-OSu, THF: H_2O (1.2:1 v/v), $NaHCO_3$, 70% over two steps; b) Nanoparticle Zn, 2 M HCl, MeOH, rt, 16 h, 78%; c) i. **20** (1 eq.), 10% Na_2CO_3, 0 °C, ii. Allyl chloroformate (1.1 eq.), dioxane, 0 °C to rt, 1.5 h, 83%; d) i. 2-chlorotrityl chloride resin, *i*Pr_2NEt, rt, 16 h; ii. 20 vol.% piperidine in DMF, rt, 2× 4 min.

(2*S*,3*S*)-2-((((9*H*-fluoren-9-yl)methoxy)carbonyl)amino)-3-((benzyloxy)(methyl)amino)butanoic acid (S10)

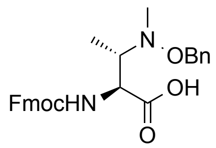

Acid **S9**[1] (4.42 g, 13 mmol) in a mixture of TFA and CH_2Cl_2 (1:1 v/v, 30 mL) was stirred at rt for 30 min. The solvent was removed *in vacuo* to give a residue which was dissolved in THF (70 mL). Saturated aqueous $NaHCO_3$ solution (70 mL) as well as solid $NaHCO_3$ (approx. 10 g) were added (until the reaction mixture was basic), followed by the addition of Fmoc-succinimide (4.62 g, 14 mmol). The reaction was allowed to stir at rt for 16 h. At this point, H_2O (100 mL) was added and the reaction mixture was washed with diethyl ether (3× 50 mL). The aqueous layer was acidified to pH 2 with 1 M HCl and extracted with EtOAc (3× 250 mL). The combined organic layers were dried over anhydrous $MgSO_4$ and the solvent was removed *in vacuo* to give a crude residue which was purified by

[1] Acid **S9** was synthesized following procedures previously reported by Boojamra *et al.*[1]

column chromatography (3:1 v/v Hexane: EtOAc → 1:1 v/v Hexane: EtOAc) to afford Fmoc-protected acid **S10** as a colourless oil (4.19 g, 70%).

$[\alpha]_D^{25}$ = +1.8° (c = 0.30 in CH_2Cl_2). **^{1}H NMR** (500 MHz, $CDCl_3$): δ 7.96-7.69 (m, 2H, Ar-H), 7.59 (dd, *J* = 7.7, 3.4 Hz, 2H, Ar-H), 7.41-7.27 (m, 9H, Ar-H), 5.60 (d, *J* = 8.3 Hz, 1H, NH), 4.75 (s, 2H, CH_2Ph), 4.43 (m, 3H, α-CH + Fmoc-CH_2), 4.23 (*app.* t, *J* = 7.2 Hz, 1H, Fmoc-CH), 3.30-3.05 (m, 1H, β-CH), 2.68 (s, 3H, NCH_3), 1.22 (d, *J* = 6.9 Hz, 3H, γ-CH_3). **^{13}C NMR** (126 MHz, $CDCl_3$): δ 173.5, 156.8, 143.8, 141.5, 136.0, 129.0, 128.8, 128.6, 127.3, 127.2, 125.3, 120.1, 74.7, 67.4, 64.2, 55.3, 47.3, 41.2, 10.1. **IR (ATR):** 2955, 1720 cm^{-1}. **LRMS** [*M*+H$^+$] 461.4. **HRMS (ESI m/z)** [*M*+Na$^+$] calcd. for $C_{27}H_{28}N_2O_5Na$, 483.1896; found, 483.1890.

(2*S*,3*S*)-2-((((9*H*-fluoren-9-yl)methoxy)carbonyl)amino)-3-(methylamino)butanoic acid (S8)

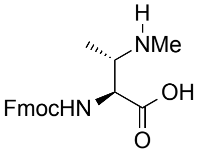

Nanoparticle zinc (2.15 g, 33 mmol) was added to a vigorously stirring solution of acid **S10** (3.04 g, 6.6 mmol) in MeOH (34 mL). A solution of 2 M HCl (34 mL) was added dropwise and the reaction was allowed to stir at rt for 2 h. A further portion of nanoparticle zinc (0.31 g, 4.8 mmol) and 2 M HCl (11 mL) were then added and the reaction was allowed to stir at rt for 16 h. The reaction was concentrated *in vacuo* to half of its volume, subsequently diluted with H_2O (400 mL) and washed with CH_2Cl_2 (80 mL). The aqueous layer was extracted with EtOAc (5× 150 mL) and EtOAc/MeOH mixture (9:1 v/v EtOAc: MeOH, 2× 150 mL). The combined organic layers were dried over anhydrous $MgSO_4$, the solvent was removed *in vacuo* to give acid **S8** as a white solid (1.82 g, 78%) that was used without further purification.

$[\alpha]_D^{25}$ = +2.4° (c = 0.40 in MeOH). **m.p.** 133-135 °C (decomp.). **^{1}H NMR** (500 MHz, CD_3OD): δ 7.86-7.76 (m, 2H, Ar-H), 7.70 (*app.* t, *J* = 7.4 Hz, 2H, Ar-H), 7.40 (dd, *J* = 8.1, 6.7 Hz, 2H, Ar-H), 7.32 (ddd, *J* = 7.7, 6.8, 1.6 Hz, 2H, Ar-H), 4.72-4.67 (m, 1H, DABA-α-CH), 4.55 (dd, *J* = 10.5, 6.7 Hz, 1H, Fmoc-CH_2), 4.41-4.32 (m, 1H, Fmoc-CH_2), 4.27 (*app.* t, *J* = 6.7 Hz, 1H, Fmoc-CH), 3.77-3.70 (m, 1H, DABA-β-CH), 2.75 (s, 3H, NCH_3), 1.26 (d, *J* = 6.5 Hz, 3H, DABA-γ-CH_3). **^{13}C**

NMR (100 MHz, $CDCl_3$): δ 174.2 (C=O), 156.7 (C=O), 144.0, 141.4, 136.2, 129.0, 128.6, 128.4, 127.8, 127.2, 125.2, 120.0, 74.5, 67.3, 64.1, 55.7, 47.2, 41.4, 14.3. **IR (ATR):** 3335, 3049, 1704 cm^{-1}. **LRMS** [*M*+H^+] 355.1. **HRMS (ESI m/z)** [*M*+Na^+] calcd. for $C_{27}H_{28}N_2O_5Na$, 483.1896; found, 483.1890.

(2*S*,3*S*)-2-((((9*H*-fluoren-9-yl)methoxy)carbonyl)amino)-3-(((allyloxy)carbonyl)amino)butanoic acid (20)

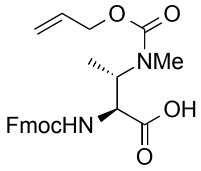

Acid **S8** (0.60 g, 1.7 mmols, 1 eq.) was dissolved in 10% aqueous sodium carbonate (14.3 mL) and cooled to 0 °C. A solution of allyl chloroformate (196 uL, 1.9 mmol, 1.1 eq.) in 1,4-dioxane (6.2 mL) was added dropwise to the above reaction mixture and the reaction was allowed to warm to rt and stirred for 1.5 h. The reaction was subsequently diluted with H_2O (120 mL), washed with diethyl ether (2 x 40 mL) and acidified to pH 2 with 1 M HCl. The aqueous layer was then extracted with EtOAc (3 x 200 mL), with the organic phases dried over $MgSO_4$, combined and concentrated *in vacuo* to a yellow oil which contained the desired acid **20** as a white foam (0.61 g, 83%).

^{1}H NMR (400 MHz, $CDCl_3$): δ 7.75 (d, *J* = 7.5 Hz, 2H, Ar-H), 7.61-7.55 (m, 2H, Ar-H), 7.39 (*app.* t, *J* = 7.5 Hz, 2H, Ar-H), 7.30 (*app.* t, *J* = 7.5 Hz, 2H, Ar-H), 5.97-5.81 (m, 2H, NH + Alloc-β-CH), 5.33-5.14 (m, 2H, Alloc-γ-CH_2), 4.73-4.32 (m, 6H, Alloc-α-CH_2 + Fmoc-CH_2 + DABA-α-CH + DABA-β-CH), 4.20 (t, *J* = 6.8 Hz, 1H, Fmoc-CH), 2.85 (s, 3H, NCH_3), 1.25 (d, *J* = 6.7 Hz, 3H, DABA-γ-CH_3). **^{13}C NMR** (101 MHz, $CDCl_3$): δ 173.0 (C=O), 156.1 (C=O), 143.8, 143.6, 141.3, 132.5, 127.7, 127.1, 125.0, 120.0, 117.6, 67.2, 66.6, 57.1, 53.4, 47.2, 29.5, 14.1. **IR (ATR):** 3312, 2984, 1688, 1683, 1528 cm^{-1}. **LRMS** [*M*+H^+] 439.3. **HRMS (ESI m/z)** [*M*+Na^+] calcd. for $C_{24}H_{26}N_2O_6Na$, 461.1688; found, 461.1683.

(2*S*,3*S*)-3-(((allyloxy)carbonyl)amino)-2-aminobutanoic acid on 2-chlorotrityl chloride resin (S11)

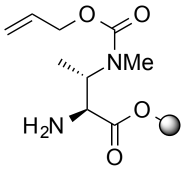

Amino acid **20** (515 mg, 1.1 mmol) was loaded onto 2-chlorotrityl chloride resin (745 mg, 1.05 mmol) in CH_2Cl_2 (6.5 mL) at rt for 16 h using *N*,*N*-diisopropylethylamine (550 µL, 3.1 mmol) and Fmoc-deprotected according to general procedure 2 (pg. 128) to afford resin-bound **S11** (65% loading as determined by spectroscopic measurement of the fulvene-piperidine adduct at λ= 301 nm).

Synthesis of tryptophan 4-nitrophenylcarbamate S12

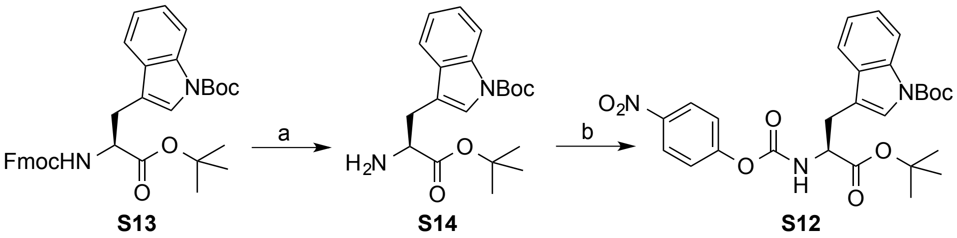

Synthesis of tryptophan 4-nitrophenyl carbamate **S12**. Reagents and conditions: a) 20 vol.% piperidine in MeCN, rt, 30 min, 75%; b) 4-nitrophenyl chloroformate, CH_2Cl_2, rt, 18 h, 80%.

***tert*-butyl (*S*)-3-(3-(*tert*-butoxy)-2-(((4-nitrophenoxy)carbonyl)amino)-3-oxopropyl)-1*H*-indole-1-carboxylate (S12)**

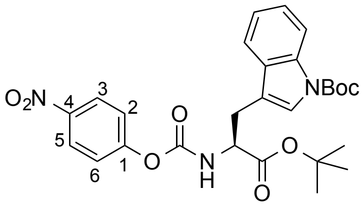

tert-Butyl ester **S13** (2.76 g, 4.74 mmol) was treated with 20 vol.% piperidine in acetonitrile (20 mL) and the reaction was allowed to stir at rt for 20 min. The solvent was removed *in vacuo* to give a crude residue that was purified by column chromatography (3:1 v/v Hexane: EtOAc, 0.1 vol.% Et_2NH) to afford the deprotected tryptophan **S14** as a yellow oil (1.28 g, 75%). To a solution of **S14** (1.28 g, 3.56 mmol) and *N*,*N*-diisopropylethylamine (620 µL, 3.56 mmol) in CH_2Cl_2 (26

mL) was added *p*-nitrophenyl chloroformate (860 mg, 4.27 mmol) and the reaction was allowed to stir at rt for 19 h. The solvent was removed *in vacuo* to give a crude residue that was purified by column chromatography (7:1 v/v Hexane: EtOAc → 5:1 v/v Hexane: EtOAc) to afford carbamate **S12** as a white foam (1.49 g, 80%).

$[\alpha]_D^{25}$ = +75° (c = 0.36 in CH_2Cl_2). **^{1}H NMR** (400 MHz, $CDCl_3$): δ 8.23 (d, *J* = 9.1 Hz, 2H, H-3 + H-5), 7.57 (*app.* dt, *J* = 7.9, 1.0 Hz, 1H, Ar-H), 7.46 (s, 1H, Ar-H), 7.34 (ddd, *J* = 8.4, 7.2, 1.3 Hz, 1H, Ar-H), 7.29-7.21 (m, 3H, Ar-H), 5.74 (d, *J* = 7.8 Hz, 1H, NH), 4.70-4.58 (m, 1H, α-CH), 3.35 (dd, *J* = 14.8, 5.8 Hz, 1H, β-CH_2), 3.25 (dd, *J* = 14.9, 5.5 Hz, 1H, β-CH_2), 1.66 (s, 9H, 3× CH_3), 1.46 (s, 9H, 3× CH_3). **^{13}C NMR** (101 MHz, $CDCl_3$): δ 170.0 (C=O), 155.7 (C=O), 152.5 (C=O), 144.9, 130.7, 125.5, 125.1, 124.7, 124.2, 122.7, 122.0, 121.6, 118.9, 115.4, 114.8, 83.8, 83.1, 54.9, 28.2, 28.0, 27.5. **IR (ATR):** 2988, 2973, 1724 cm^{-1}. **LRMS** [*M*(-NO_2Ph+Me)+Na^+] 441.0. **HRMS (ESI m/z)** [*M*(-NO_2Ph+Me)+Na]$^+$ calcd. for $C_{22}H_{30}N_2O_6Na$ 441.2002; found, 441.1995.

General procedure 1: Synthesis of isopeptides S15-S21

To a solution of a Boc-protected amino acid (1 eq.) in DMF (4-8 mL/mmol) was added HATU (1 eq.) and the solution was cooled to 0 °C after first stirring at rt for 10 min. **S8** (1 eq.) was added, followed by NMM or *N*,*N*-diisopropylethylamine (3 eq.) and the reaction was allowed to warm to rt and stirred for 3-6 h. Upon completion, the reaction was diluted with EtOAc (60-100 mL) and washed successively with 0.2 M HCl (15-20 mL), water (5× 15 mL) and brine (15 mL). The solvent was removed *in vacuo* to give a crude residue that was purified by column chromatography or reverse phase HPLC to afford isopeptides **S15-S21**.

(2*S*,3*S*)-2-((((9*H*-fluoren-9-yl)methoxy)carbonyl)amino)-3-((*S*)-2-((*tert*-butoxycarbonyl)amino)-3-(3-((*tert*-butyldimethylsilyl)oxy)phenyl)-*N*-methylpropanamido)butanoic acid (S15)

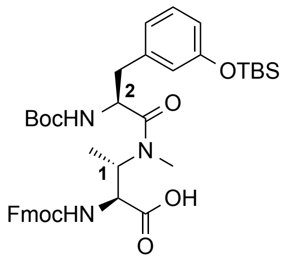

Boc-L-*m*-Tyr(O^{t}Bu)-OH (384 mg, 1.14 mmol) was reacted with **S8** (406 mg, 1.14 mmol) in the presence of HATU (433 mg, 1.14 mmol) and NMM (376 μL, 3.42 mmol) in DMF (8.9 mL) according to general procedure 1 for 6 h to obtain isopeptide **S15** after column chromatography (1:1 v/v EtOAc: Hexane → EtOAc) as a white amorphous solid (384 mg, 50%).

$[\alpha]_D^{25}$ = -3.8° (c = 0.39 in CH_2Cl_2). **^{1}H NMR** (400 MHz, $CDCl_3$): δ 7.81-7.68 (m, 2H, Ar-H), 7.64-7.53 (m, 2H, Ar-H), 7.43-7.34 (m, 2H, Ar-H), 7.33-7.27 (m, 2H, Ar-H), 7.08 (d, *J* = 7.8 Hz, 2H, Ar-H, H-2 + H-6), 6.94-6.83 (m, 2H, Ar-H, H-3 + H-5), 5.91 (s, 1H, NH), 5.58 (d, *J* = 8.6 Hz, 1H, NH), 4.92-4.83 (m, 1H, Tyr2-α-CH), 4.80-4.67 (m, 1H, DABA1-β-CH), 4.40-4.29 (m, 3H, Fmoc-CH_2 + DABA1-α-CH), 4.20 (*app.* t, *J* = 6.7 Hz, 1H, Fmoc-CH), 3.05-2.87 (m, 4H, NCH_3 + Tyr2-β-CH_2), 2.81-2.68 (m, 1H, Tyr2-β-CH_2), 1.56-1.00 (m, 21H, Boc + ^{t}Bu + DABA1-γ-CH_3). **IR (ATR):** 3335, 3045, 1704 cm^{-1}. **LRMS** [M+H$^+$] 673.3. **HRMS (ESI m/z)** [M+Na$^+$] calcd. for $C_{38}H_{47}N_3O_8Na$, 696.3261; found, 696.3266.

(2*S*,3*S*)-2-((((9*H*-fluoren-9-yl)methoxy)carbonyl)amino)-3-((*S*)-2-((*tert*-butoxycarbonyl)amino)-*N*-methyl-3-phenylpropanamido)butanoic acid (S16)

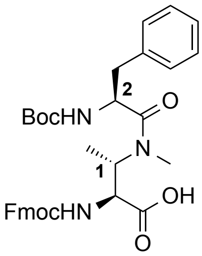

Boc-L-Phe-OH (70 mg, 0.26 mmol) was reacted with **S8** (100 mg, 0.28 mmol) in the presence of HATU (106 mg, 0.28 mmol) and NMM (9.2 μL, 0.84 mmol) in DMF (1.1 mL) according to general procedure 1 for 4 h to obtain isopeptide **S16** after column chromatography (95:5 v/v CH_2Cl_2: MeOH) as a white foam (73 mg, 46%).

$[\alpha]_D^{25}$ = -3.2° (c = 0.31 in CH_2Cl_2). **^{1}H NMR** (300 MHz, $CDCl_3$, *major rotamer*) δ 7.82 (d, *J* = 7.5 Hz, 2H, 2 x Ar-H), 7.69 (m, 2H, 2 x Ar-H), 7.39-7.34 (m, 2H, 2 x Ar-H), 7.30-6.96 (m, 7H, 4 x Ar-H + H-2 + H-3 + H-5), 5.93 (d, *J* = 8.4 Hz, 1H, N-H), 5.78 (d, *J* = 8.2 Hz, 1H, N-H), 5.03 (m, 1H, Phe2-α-CH), 4.68 (m, 1H, DABA1-β-CH), 4.53 (m, 1H, DABA1-α-CH), 4.40-4.27 (m, 3H, Fmoc-CH_2 + Fmoc-CH), 3.00-

2.77 (m, 4H, NCH_3 + Phe2-β-CH_2), 2.74 (m, 1H, Phe2-β-CH_2), 1.34-1.05 (m, 12H, Boc + DABA1-γ-CH_3). **IR (ATR):** 3316, 2979, 2919, 1712, 1640 cm^{-1}. **LRMS** [M^+H^+] 602.4. **HRMS (ESI m/z)** [M+Na^+] calcd. for $C_{34}H_{39}N_3O_7Na$, 624.2685; found, 624.2683.

(2*S*,3*S*)-2-((((9*H*-fluoren-9-yl)methoxy)carbonyl)amino)-3-(2-((*tert*-butoxycarbonyl)amino)-*N*-methylacetamido)butanoic acid (S17)

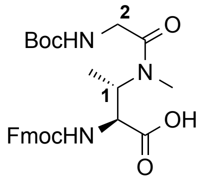

Boc-Gly-OH (100 mg, 0.56 mmol) was reacted with **S8** (200 mg, 0.56 mmol) in the presence of HATU (214 mg, 0.56 mmol) and *N*,*N*-diisopropylethylamine (294 μL, 1.69 mmol) in DMF (4 mL) according to general procedure **1** for 3 h to obtain isopeptide **S17** after column chromatography (98:2 v/v CH_2Cl_2: MeOH → 95:5 v/v CH_2Cl_2: MeOH → 9:1 v/v CH_2Cl_2: MeOH, 0.1 vol.% CH_3COOH) as a white foam (108 mg, 42%).

$[\alpha]_D^{25}$ = -20.7° (c = 0.58 in CH_2Cl_2). **^{1}H NMR** (400 MHz, $CDCl_3$, *major rotamer*): δ 7.83-7.69 (m, 2H, Ar-H), 7.61-7.54 (m, 2H, Ar-H), 7.38 (ddd, *J* = 8.2, 5.4, 2.2 Hz, 2H, Ar-H), 7.29 (*app.* t, *J* = 7.2 Hz, 2H, Ar-H), 5.87 (d, *J* = 8.1 Hz, 1H, NH), 5.80 (s, 1H, NH), 4.99-4.77 (m, 1H, DABA1-β-CH), 4.54-4.28 (m, 3H, DABA1-α-CH + Fmoc-CH_2), 4.20 (*app.* t, *J* = 6.8 Hz, 1H, Fmoc-CH), 4.12-3.88 (m, 1H, Gly2-α-CH_2), 3.80 (d, *J* = 17.1 Hz, 1H, Gly2-α-CH_2), 2.80 (s, 3H, NCH_3), 1.47-1.34 (m, 9H, 3× CH_3, Boc), 1.22 (d, *J* = 6.8 Hz, 3H, DABA1-γ-CH_3). **IR (ATR):** 3428, 3319, 2972, 1714, 1649 cm^{-1}. **LRMS** [M+H^+] 512.3. **HRMS (ESI m/z)** [M+Na^+] calcd. for $C_{27}H_{33}N_3O_7Na$, 534.2210; found, 534.2212.

(2*S*,3*S*)-2-((((9*H*-fluoren-9-yl)methoxy)carbonyl)amino)-3-((*S*)-4-(*tert*-butoxy)-2-((*tert*-butoxycarbonyl)amino)-*N*-methyl-4-oxobutanamido)butanoic acid (S18)

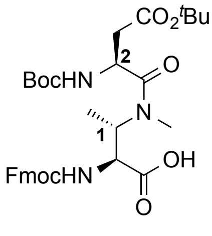

Boc-L-Asp(O^{t}Bu)-OH (162 mg, 0.56 mmol) was reacted with **S8** (200 mg, 0.56 mmol) in the presence of HATU (214 mg, 0.56 mmol) and *N*,*N*-diisopropylethylamine (294 μL, 1.69 mmol) in DMF (8.9 mL) according to general procedure 1 for 3.5 h to obtain isopeptide **S18** after column chromatography (98:2 v/v CH_2Cl_2: MeOH → 95:5 v/v CH_2Cl_2: MeOH → 9:1 v/v CH_2Cl_2: MeOH, 0.1 vol.% CH_3COOH) as a white foam (156 mg, 45%).

$[\alpha]_D^{25}$ = -17.7° (c = 0.3 in CH_2Cl_2). **IR (ATR):** 3307, 2976, 2929, 1710, 1641 cm^{-1}. **^{1}H NMR** (400 MHz, $CDCl_3$, rotamers in 2.5:1 ratio, *major rotamer*): δ 7.78-7.70 (m, 2H, Ar-H), 7.59 (*app.* t, *J* = 7.5 Hz, 2H, Ar-H), 7.42-7.35 (m, 2H, Ar-H), 7.33-7.27 (m, 2H, Ar-H), 5.93 (s, 1H, NH), 5.75 (d, *J* = 9.2 Hz, 1H, NH), 4.99-4.89 (m, 1H, Asp2-α-CH), 4.87-4.76 (m, 1H, DABA1-β-CH), 4.52-4.29 (m, 3H, DABA1-α-CH + Fmoc-CH_2), 4.24-4.17 (m, 1H, Fmoc-CH), 3.06 (s, 3H, NCH_3), 2.83-2.64 (m, 1H, Asp2-β-CH_2), 2.43 (dd, *J* = 15.3, 8.5 Hz, 1H, Asp2-β-CH_2), 1.52-1.32 (m, 18H, 6× CH_3, Boc + $CO_2{}^t$Bu), 1.32-1.18 (m, 3H, DABA1-γ-CH_3). **^{13}C NMR** (101 MHz, $CDCl_3$, *major rotamer*): δ 172.9 (C=O), 172.2 (C=O), 170.1 (C=O), 156.1 (C=O), 155.3 (C=O), 143.6, 141.3, 127.7, 127.1, 125.1, 120.0, 82.1, 80.8, 67.1, 56.5, 52.5, 48.6, 47.2, 38.4, 30.5, 28.3, 28.1, 14.1. **HRMS (ESI m/z)** [*M*+Na$^+$] calcd. for $C_{33}H_{43}N_3O_9Na$ 648.2891, found 648.2893.

(2*S*,3*S*)-2-((((9*H*-fluoren-9-yl)methoxy)carbonyl)amino)-3-((*S*)-2,6-bis((*tert*-butoxycarbonyl)amino)-*N*-methylhexanamido)butanoic acid (S19)

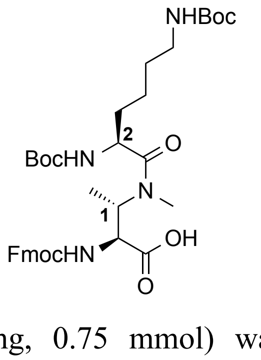

Boc-L-Lys(NHBoc)-OH (260 mg, 0.75 mmol) was reacted with **S8** (267 mg, 0.75 mmol) in the presence of HATU (287 mg, 0.75 mmol) and *N*,*N*-diisopropylethylamine (390 μL, 2.25 mmol) in DMF (12 mL) according to general procedure 1 for 3 h to obtain isopeptide **S19** after column chromatography (98:2 v/v CH_2Cl_2: MeOH → 95:5 v/v CH_2Cl_2: MeOH → 9:1 v/v CH_2Cl_2: MeOH, 0.1 vol.% CH_3COOH) as a white foam (279 mg, 55%).

$[\alpha]_D^{25}$ = -4.8° (c = 0.31 in CH_2Cl_2). **IR (ATR):** 3318, 2975, 2933, 1708, 1650 cm^{-1}. **^{1}H NMR** (400 MHz, $CDCl_3$, rotamers in 2:1 ratio, *major rotamer*): δ 7.79-7.70 (2H, m, Ar-H), 7.59 (2H, dd, *J* = 7.5, 4.3 Hz, Ar-H), 7.43-7.35 (2H, m, Ar-H), 7.31 (2H, *app.* t, *J* = 7.3 Hz, Ar-H), 5.87 (1H, s, NH), 5.62 (1H, s, NH), 5.53 (1H, d, *J* = 8.2 Hz, NH), 5.00-4.86 (1H, m, DABA1-β-CH), 4.72-4.52 (1H, m, Lys2-α-CH), 4.50-4.31 (3H, m, DABA1-α-CH + Fmoc-CH_2), 4.22 (1H, *app.* t, *J* = 6.9 Hz, Fmoc-CH), 3.25-3.06 (2H, m, Lys2-ε-CH_2), 2.80 (3H, s, NCH_3), 1.83-1.66 (1H, m, Lys2-β-CH_2), 1.64-1.27 (23H, m, 2× Boc + Lys2-β-CH_2 + Lys2-γ-CH_2 + Lys2-δ-CH_2), 1.21 (3H, d, *J* = 6.8 Hz, DABA1-γ-CH_3). **^{13}C NMR** (100 MHz, $CDCl_3$, 1 carbon signal obscure): δ 172.8 (C=O), 166.1 (C=O), 159.5 (C=O), 156.1 (C=O), 155.4 (C=O), 143.9, 141.4, 127.8, 127.3, 125.2, 120.1, 81.6, 79.9, 67.3, 56.6, 51.4, 50.5, 47.3, 40.4, 38.8, 29.8, 28.5, 28.4, 14.8. **LRMS** [M+H^+] 683.5. **HRMS (ESI m/z)** [M+Na^+] calcd. for $C_{36}H_{50}N_4O_9Na$ 705.3470, found 705.3477.

(2*S*,3*S*)-2-((((9*H*-fluoren-9-yl)methoxy)carbonyl)amino)-3-((*S*)-2,5-bis((*tert*-butoxycarbonyl)amino)-*N*-methylpentanamido)butanoic acid (S20)

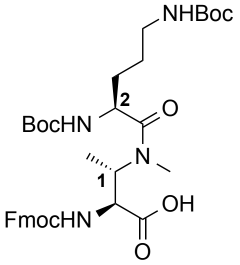

Boc-L-Orn(NHBoc)-OH (250 mg, 0.75 mmol) was reacted with **S8** (267 mg, 0.75 mmol) in the presence of HATU (287 mg, 0.75 mmol) and *N*,*N*-diisopropylethylamine (390 μL, 2.25 mmol) in DMF (12 mL) according to general procedure **1** for 3 h to obtain isopeptide **S20** after column chromatography (98:2 v/v CH_2Cl_2: MeOH → 95:5 v/v CH_2Cl_2: MeOH → 9:1 v/v CH_2Cl_2: MeOH, 0.1 vol.% CH_3COOH) as a white foam (267 mg, 52%).

$[\alpha]_D^{25}$ = +15.1° (c = 0.45 in CH_2Cl_2). **IR (ATR):** 3330, 2976, 2931, 1708, 1640 cm^{-1}. **^{1}H NMR** (400 MHz, $CDCl_3$, rotamers in 4:1 ratio, *major rotamer*): δ 7.87-7.73 (2H, m, Ar-H), 7.62 (2H, ddd, *J* = 7.6, 4.2, 1.9 Hz, Ar-H), 7.47-7.38 (2H, m, Ar-H), 7.36-7.30 (2H, m, Ar-H), 5.78-5.47 (2H, m, 2× NH), 5.11 (1H, s, NH), 5.03-4.91 (1H, m, DABA1-β-CH), 4.72-4.59 (1H, m, Orn2-α-CH), 4.54-4.34 (3H, m, DABA1-α-CH + Fmoc-CH_2), 4.24 (1H, *app.* t, *J* = 6.9 Hz, Fmoc-CH), 3.30-3.10 (1H, m, Orn2-δ-CH_2),

2.94-2.86 (1H, m, Orn2-δ-CH_2), 2.83 (3H, s, NCH_3), 1.96-1.66 (1H, m, Orn2-β-CH_2), 1.63-1.32 (21H, m, 2× Boc + Orn2-β-CH_2 + Orn2-γ-CH_2), 1.26-1.16 (3H, m, DABA1-γ-CH_3). **^{13}C NMR** (101 MHz, $CDCl_3$, *major rotamer*): δ 174.8 (C=O), 171.9 (C=O), 157.3 (C=O), 156.0 (C=O), 155.3 (C=O), 143.8, 141.3, 127.7, 127.1, 125.1, 120.0, 80.5, 79.7, 67.1, 56.4, 51.1, 50.4, 47.2, 40.7, 29.7, 29.2, 28.4, 24.8, 14.3 **LRMS** [*M*+H^+] 669.7. **HRMS (ESI m/z)** [*M*+Na^+] calcd. for $C_{35}H_{48}N_4O_9Na$ 691.3313, found 691.3314.

(2*S*,3*S*)-2-((((9*H*-fluoren-9-yl)methoxy)carbonyl)amino)-3-((2*S*,3*R*)-2-((*tert*-butoxycarbonyl)amino)-3-hydroxy-*N*-methylbutanamido)butanoic acid (S21)

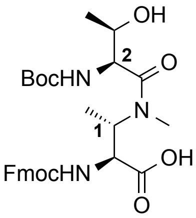

To a solution of Boc-L-Thr-OH (95 mg, 0.43 mmol) in DMF (4.5 mL) was added HATU (163 mg, 0.43 mmol), HOAt (658 mg, 4.3 mmol) and *N*,*N*-diisopropylethylamine (75 μL, 0.43 mmol) and the solution was cooled to 0 °C after stirring at rt for 5 min. **S8** (152 mg, 0.43 mmol) was added, followed by *N*,*N*-diisopropylethylamine (150 μL, 0.86 mmol) and the reaction was warmed to rt. The reaction was allowed to stir at ambient temperature for 4 h. Upon completion, the reaction was diluted with EtOAc (60-100 mL) and washed successively with 0.2 M HCl (15-20 mL), water (5× 15 mL) and brine (15 mL). The solvent was removed *in vacuo* to give a crude residue that was purified by column chromatography (98:2 v/v CH_2Cl_2: MeOH → 95:5 v/v CH_2Cl_2: MeOH → 9:1 v/v CH_2Cl_2: MeOH, 0.1 vol.% CH_3COOH) to afford isopeptide **S21** as a white foam (160 mg, 37%).

$[\alpha]_D^{25}$ = -16.3° (c = 0.46 in CH_2Cl_2). **IR (ATR):** 3307, 2976, 1710, 1635 cm^{-1}. **^{1}H NMR** (400 MHz, DMSO-d_6, rotamers in 3:1 ratio, *major rotamer*): δ 7.89 (2H, *app.* d, *J* = 7.6 Hz, Ar-H), 7.73 (2H, *app.* t, *J* = 6.1 Hz, Ar-H), 7.42 (2H, td, *J* = 7.6, 1.4 Hz, Ar-H), 7.37-7.29 (2H, m, Ar-H), 6.49 (0.5H, d, *J* = 8.1 Hz, NH), 4.94-4.81 (1H, m, DABA1-β-CH), 4.74 (0.7H, s), 4.47-4.13 (5H, m, DABA1-α-CH + Thr2-α-CH + Fmoc-CH + Fmoc-CH_2), 3.92-3.63 (1H, m, Thr2-β-CH), 2.69 (3H, s, NCH_3), 1.37 (9H, s, 3× CH_3, Boc), 1.10 (3H, d, *J* = 6.9 Hz, DABA1-γ-CH_3), 1.00 (3H, d, *J* = 6.3

Hz, Thr2-γ-CH_3). **LRMS** [M+H^+] 556.4. **HRMS (ESI m/z)** [M+Na^+] calcd. for $C_{29}H_{37}N_3O_8Na$ 578.2472, found 578.2478.

Synthesis of depsipeptides S22-S52

General procedure 2: Loading amino acids onto 2-chlorotrityl chloride resin

2-Chlorotrityl chloride resin (100-200 mesh) with 1% DVB (1.22-1.42 mmol/g, 50-730 µmol, 1 eq.) was allowed to swell in anhydrous CH_2Cl_2 (3-8 mL) for 30 min. Isopeptides **S15-S21** (100-320 µmol, 2 eq.) or amino acid **20** (1.1-2.5 mmol, 1.2 eq.) were dissolved in anhydrous CH_2Cl_2 (2-2.6 mL/100 µmol for isopeptides **S15-S21** and 0.6 mL/100 µmol for amino acid **20**). *N*,*N*-diisopropylethylamine (200-2920 µmol, 2-8 eq. for isopeptides **S15-S21** and 2.4-3 eq. for amino acid **20**) was added and the resin was shaken for 16 h at rt. The resin was subsequently washed with DMF (5× 5 mL), CH_2Cl_2 (5× 5 mL) and DMF (5× 5 mL). The resin was capped by treatment with 17:2:1 v/v/v CH_2Cl_2: MeOH: *i*Pr_2NEt (3-6 mL) for 40 min. The resin was then washed with DMF (5× 5 mL), CH_2Cl_2 (5× 5 mL) and DMF (5× 5 mL).

General procedure 3: Fmoc-strategy solid phase peptide synthesis

Fmoc deprotection: A solution of 10 vol.% piperidine/DMF (5 mL) was added to the resin and shaken for 4 min (× 2). The resin was subsequently washed with DMF (5× 3 mL), CH_2Cl_2 (5× 3 mL) and DMF (5× 3 mL). The efficiency of the previous amino acid coupling was determined by spectroscopic measurement of the resulting fulvene piperidine adduct at λ = 301 nm.

Amino Acid Coupling:

<u>Condition A</u>: A solution of protected amino acid (200-640 µmol, 4 eq.), PyBOP (200-640 µmol, 4 eq.) and NMM (0.4-1.28 mmol, 8 eq.) in DMF (0.1 M) was added to the resin (1 eq.) and shaken for 1 h at rt. The resin was then washed with DMF (5× 5 mL), CH_2Cl_2 (5× 5 mL) and DMF (5× 5 mL).

<u>Condition B:</u> A solution of Fmoc-protected amino acid (72-96 µmol, 1.2 eq.), HOAt (72-96 µmol, 1.2 eq.) and DIC (72-96 µmol, 1.2 eq.) in DMF (0.1 M) was added to the resin (1 eq.) and shaken for 16 h at rt. The resin was then washed with DMF (5× 3 mL), CH_2Cl_2 (5× 3 mL) and DMF (5× 3 mL).

Capping: A solution of 10 vol.% acetic anhydride/pyridine (5 mL) was added to the resin and shaken for 3 min. The resin was subsequently washed with DMF (5× 5 mL), CH_2Cl_2 (5× 5 mL) and DMF (5× 5 mL).

General procedure 4: Solid-phase urea formation

A solution of carbamate **S12** (90-320 μmol, 2 eq.) and *N*,*N*-diisopropylethylamine (180-640 μmol, 4 eq.) in DMF (17 μL/μmol) was added to the resin (45-160 μmol, 1 eq.). The resin was shaken at rt for 6 h. The resin was subsequently washed with DMF (5× 5 mL), CH_2Cl_2 (5× 5 mL) and DMF (5× 5 mL).

General Procedure 5: Solid Phase Deprotection of Allyl Carbamate

A solution of *tetrakis*(triphenylphosphine) palladium(0) (20 μmol, 0.2 eq.) and phenylsilane (2 mmol, 20 eq.) in CH_2Cl_2 (80 mM) was added to the resin (1 eq.) and shaken for 15 min at rt. The solvents were subsequently removed from the resin and the resin washed with CH_2Cl_2 (5 x 5 mL), DMF (5 x 5 mL) and CH_2Cl_2 (5 x 5 mL). The treatment was then repeated once.

General Procedure 6: Solid Phase Coupling to *N*-methylated Amino Acid Residues

Amino acid coupling: A solution of Boc-protected amino acid (80-580 μmol, 2 eq.), HATU (80-580 μmol, 2 eq.) and DIPEA (120-870 μmol, 2-3 eq.) in DMF (0.1 M) was added to the resin (1 eq.) and shaken for 2 h at rt. The resin was then washed with DMF (5 x 5 mL), CH_2Cl_2 (5 x 5 mL) and DMF (5 x 5 mL).

Capping: A solution of 10 vol.% acetic anhydride in pyridine (5 mL) was added to the resin and shaken for 3 min. The resin was then washed with DMF (5 x 3 mL), CH_2Cl_2 (5 x 3 mL) and DMF (5 x 3 mL).

General procedure 7: Cleavage from 2-chlorotrityl chloride resin and work-up

A solution of 30 vol.% hexafluoroisopropanol (HFIP) in CH_2Cl_2 (5-10 mL) was added to the resin and shaken for 30 min at rt. The resin was subsequently washed with CH_2Cl_2 (6× 10 mL) and the combined cleavage and washing solutions were concentrated *in vacuo*. The resulting residue was dissolved in 9:1 v/v MeCN: H_2O and purified by reverse phase HPLC.

(2*S*,5*S*,9*S*)-9-((1-(*tert*-butoxycarbonyl)-1*H*-indol-3-yl)methyl)-2-((*S*)-1-((*S*)-2-((*tert*-butoxycarbonyl)amino)-3-(3-((*tert*-butyldimethylsilyl)oxy)phenyl)-*N*-methylpropanamido)ethyl)-12,12-dimethyl-5-(2-(methylsulfinyl)ethyl)-4,7,10-trioxo-11-oxa-3,6,8-triazatridecanoic acid (S22)

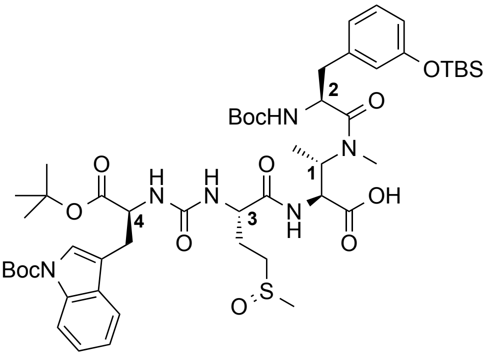

Isopeptide **S15** (146 mg, 200 μmol) was loaded onto 2-chlorotrityl chloride resin (83 mg, 100 μmol) in CH_2Cl_2 (2 mL) using *N*,*N*-diisopropylethylamine (40 μL, 220 μmol) and Fmoc-deprotected according to general procedure 2. Fmoc-L-Met(O)-OH (387 mg, 400 μmol) was subsequently coupled using PyBOP (208 mg, 400 μmol) and NMM (82 μL, 800 μmol) in DMF (1 mL) (general procedure 3). Carbamate **S12** (106 mg, 200 μmol) was subsequently coupled according to general procedure 4. Following cleavage from the resin (general procedure 7), the peptide was purified by reverse phase HPLC (50-100% MeCN over 40 min, 10 min at 50% MeCN) to afford depsipeptide **S22** as a fluffy white solid (26.2 mg, 26%).

IR (ATR): 3300, 2923, 2853, 1733, 1638 cm^{-1}. ^{1}**H NMR** (400 MHz, $CDCl_3$, *major rotamer*): δ 8.26 (1H, br. s), 8.21 (1H, br. s), 8.15-8.00 (1H, m, Ar-H), 7.55 (1H, dd, *J* = 8.0, 3.1 Hz, Ar-H), 7.39 (1H, s, Ar-H), 7.29-7.23 (1H, m, Ar-H), 7.17 (1H, *app.* q, *J* = 7.0 Hz, Ar-H), 7.09 (1H, *app.* td, *J* = 7.8, 3.1 Hz, Ar-H), 6.88 (1H, *app.* t, *J* = 9.8 Hz, Ar-H), 6.80 (1H, dd, *J* = 6.4, 4.4 Hz, Ar-H), 6.68 (1H, dt, *J* = 8.4, 2.6 Hz, Ar-H), 6.11 (1H, s, NH), 5.85 (1H, s, NH), 5.60 (1H, s, NH), 5.36 (1H, s, NH), 5.13-4.97 (1H, m, DABA1-β-CH), 4.73-4.59 (2H, m, *m*-Tyr2-α-CH, Trp4-α-CH), 4.50 (1H, *app.* t, *J* = 8.8 Hz, DABA1-α-CH), 3.35-3.03 (5H, m, Trp4-β-CH_2 + NCH_3), 3.02-2.92 (1H, m, *m*-Tyr2-β-CH_2), 2.93-2.70 (4H, m, *m*-Tyr2-β-CH_2, Met(O)3-α-CH + Met(O)3-γ-CH_2), 2.51 (3H, s, S(O)CH_3), 2.40-2.25 (1H, m, Met(O)3-β-CH_2), 2.09-1.91 (1H, m, Met(O)3-β-CH_2), 1.62 (9H, s, 3× CH_3), 1.36-1.19 (21H, m, 2× Boc + $CO_2{}^{t}Bu$ + DABA1-γ-CH_3), 0.97 (9H, s, $(CH_3)_3CSi$), 0.19 (6H, s, 2× CH_3Si). **LRMS**

[M+H$^+$] 1043.6. **HRMS (ESI m/z)** [M+Na$^+$] calcd. for $C_{51}H_{78}N_6O_{13}SSiNa$ 1065.5009, found 1065.5005.

(2*S*,5*S*,9*S*)-9-((1-(*tert*-butoxycarbonyl)-1*H*-indol-3-yl)methyl)-2-((*S*)-1-((*S*)-2-((*tert*-butoxycarbonyl)amino)-3-(3-((*tert*-butyldimethylsilyl)oxy)phenyl)-*N*-methylpropanamido)ethyl)-5-isobutyl-12,12-dimethyl-4,7,10-trioxo-11-oxa-3,6,8-triazatridecanoic acid (S23)

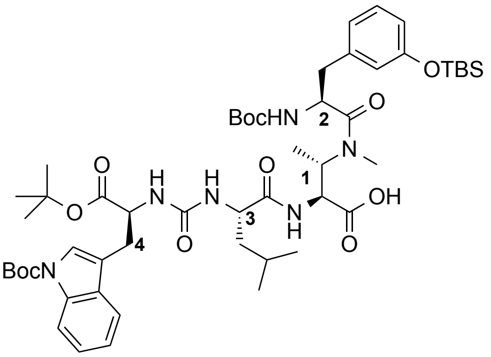

Isopeptide **S15** (73 mg, 100 μmol) was loaded onto 2-chlorotrityl chloride resin (41 mg, 50 μmol) in CH_2Cl_2 (1 mL) using *N*,*N*-diisopropylethylamine (20 μL, 110 μmol) and Fmoc-deprotected according to general procedure 2. Fmoc-L-Leu-OH (71 mg, 200 μmol) was subsequently coupled using PyBOP (104 mg, 200 μmol) and NMM (41 μL, 400 μmol) in DMF (0.5 mL) (general procedure 3). Carbamate **S12** (53 mg, 100 μmol) was subsequently coupled according to general procedure 4. Following cleavage from the resin (general procedure 7), the peptide was purified by reverse phase HPLC (50-100% MeCN over 40 min, 10 min at 50% MeCN) to afford depsipeptide **S23** as a fluffy white solid (17.6 mg, 35%).

IR (ATR): 3300, 2923, 2853, 1733, 1625 cm^{-1}. **^{1}H NMR** (400 MHz, $CDCl_3$, *major rotamer*): δ 8.04 (1H, *app.* d, J = 8.0 Hz, Ar-H), 7.64 (1H, d, J = 5.2 Hz, Ar-H), 7.54 (1H, *app.* d, J = 7.7 Hz, Ar-H), 7.41 (1H, s, Ar-H), 7.30-7.24 (1H, m, Ar-H), 7.24-7.18 (1H, m, Ar-H), 7.04 (1H, *app.* t, J = 7.7 Hz, Ar-H), 6.69-6.62 (3H, m, Ar-H), 5.73 (1H, s, NH), 5.47 (1H, s, NH), 4.91-4.53 (4H, m, DABA1-α-CH + DABA1-β-CH + *m*-Tyr2-α-CH + Trp4-α-CH), 4.33-4.15 (1H, m, Leu3-α-CH), 3.22-3.07 (2H, m, Trp4-β-CH_2), 3.03-2.90 (4H, m, NCH_3, *m*-Tyr2-β-CH_2), 2.72-2.54 (1H, m, *m*-Tyr2-β-CH_2), 1.72-1.45 (12H, m, 3× CH_3, Leu3-β-CH_2, Leu3-γ-CH), 1.44-1.25 (18H, m, 6× CH_3), 1.23 (3H, d, J = 6.9 Hz, DABA1-γ-CH_3), 0.96 (9H, m, $(CH_3)_3CSi$), 0.94-

0.81 (6H, m, 2× Leu3-δ-CH_3), 0.17 (6H, s, 2× *CH_3*Si). **LRMS** [*M*+H^+] 1009.7. **HRMS** (ESI m/z) [M+Na^+] calcd. for $C_{52}H_{80}N_6O_{12}Na$ 1031.5495, found 1031.5501.

(2*S*,5*S*,9*S*)-9-((1-(*tert*-butoxycarbonyl)-1*H*-indol-3-yl)methyl)-2-((*S*)-1-((*S*)-2-((*tert*-butoxycarbonyl)amino)-*N*-methyl-3-phenylpropanamido)ethyl)-5-isobutyl-12,12-dimethyl-4,7,10-trioxo-11-oxa-3,6,8-triazatridecan-1-oic acid (S24)

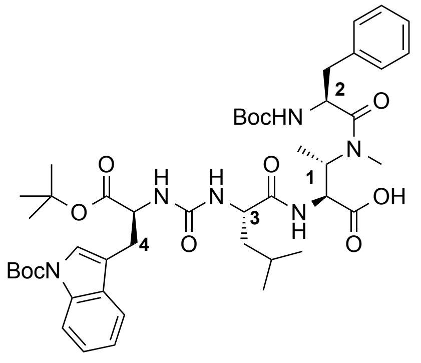

Isopeptide **S16** (105 mg, 175 μmol) was loaded onto 2-chlorotrityl chloride resin (62 mg, 88 μmol) in CH_2Cl_2 (2 mL) using *N*,*N*-diisopropylethylamine (61 μL, 350 μmol) and Fmoc-deprotected according to general procedure 2. Fmoc-L-Leu-OH (124 mg, 350 μmol) was subsequently coupled using PyBOP (182 mg, 350 μmol) and NMM (77 μL, 700 μmol) in DMF (1 mL) (general procedure 3). Carbamate **S12** (92 mg, 175 μmol) was subsequently coupled according to general procedure 4. Following cleavage from the resin (general procedure 7), the peptide was purified by reverse phase HPLC (50-100% MeCN over 40 min, 10 min at 50% MeCN) to afford depsipeptide **S24** as a fluffy white solid (12.4 mg, 39%).

IR (ATR): 3340, 2976, 2931, 1730, 1635 cm^{-1}. **^{1}H NMR** (400 MHz, $CDCl_3$): δ 8.12 (1H, d, *J* = 8.0 Hz, Ar-H), 7.83 (1H, d, *J* = 8.0 Hz, Ar-H), 7.69 (1H, d, *J* = 4.4 Hz, Ar-H), 7.52 (1H, s, Ar-H), 7.34-7.15 (7H, m, 7x Ar-H), 6.24-6.14 (1H, m, N-H), 6.07-5.87 (1H, m, N-H), 5.05 (1H, m, DABA1-β-CH), 4.80-4.66 (3H, m, DABA1-α-CH + Phe2-α-CH + Trp4-α-CH), 4.43-4.38 (1H, m, Leu3-α-CH), 3.20-3.06 (5H, m, Trp4-β-CH_2 + NCH_3), 2.82–2.76 (2H, m, Phe2-β-CH_2), 1.77-1.48 (12H, m, 3x CH_3 + Leu3-β-CH_2 + Leu3-γ-CH), 1.40-1.28 (18H, m, 6x CH_3), 1.23-1.18 (3H, m, DABA1-γ-CH_3), 0.93–0.89 (6H, m, 2 x Leu3-δ-CH_3). **LRMS** [M^+H^+] 879.5. **HRMS (ESI m/z)** [M+Na^+] calcd. for $C_{46}H_{66}N_6O_{11}$ 901.4687, found 901.4687.

(2*S*,5*S*,9*S*)-9-((1-(*tert*-butoxycarbonyl)-1*H*-indol-3-yl)methyl)-5-isobutyl-12,12-dimethyl-2-((*S*)-1-(methylamino)ethyl)-4,7,10-trioxo-11-oxa-3,6,8-triazatridecanoic acid (S25)

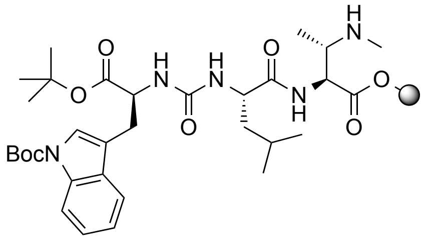

Fmoc-L-Leu-OH (1.12 g, 3.16 mmol) was coupled to resin-bound **S11** (0.79 mmol) using standard Fmoc-SPPS procedure using PyBOP (1.64 g, 3.16 mmol) and NMM (650 µL, 6.3 mmol) in DMF (7.9 mL) (general procedure 3, condition A). Following Fmoc deprotection, carbamate **S12** (840 mg, 1.58 mmol) was subsequently coupled using *N*,*N*-diisopropylethylamine (550 µL, 3.16 mmol) according to general procedure 4. Allyl carbamate was subsequently removed using *tetrakis*(triphenylphosphine) palladium(0) (2× 194 mg, 2× 158 µmol) and phenylsilane (2× 1.9 mL, 2× 15.8 mmol) according to general procedure 5 to afford resin bound **S25**.

(2*S*,5*S*,9*S*)-9-((1-(*tert*-butoxycarbonyl)-1*H*-indol-3-yl)methyl)-2-((*S*)-1-((*S*)-2-((*tert*-butoxycarbonyl)amino)-*N*-methyl-3-(pyridin-3-yl)propanamido)ethyl)-5-isobutyl-12,12-dimethyl-4,7,10-trioxo-11-oxa-3,6,8-triazatridecanoic acid (S26)

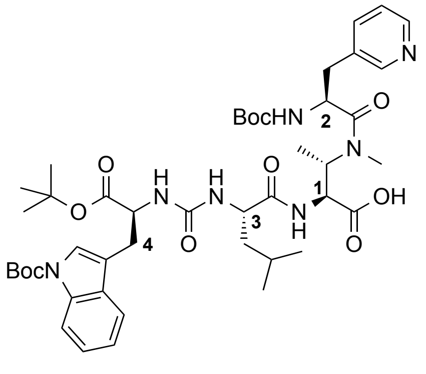

Resin-bound **S25** (60 µmol) was coupled to Boc-3-Pal-OH (32 mg, 120 µmol) using HATU (45 mg, 120 µmol) and *N*,*N*-diisopropylethylamine (33 µL, 180 µmol) in DMF (0.6 mL) according to general procedure 6. Following cleavage from the resin (general procedure 7), the peptide was purified by reverse phase HPLC (20-100% MeCN over 40 min, 10 min at 20% MeCN) to afford intermediate **S26** as a fluffy white solid (33 mg, 63%).

IR (ATR): 3361, 2977, 1730, 1639 cm^{-1}. **^{1}H NMR** (500 MHz, Acetone-d_6): δ 8.54-8.46 (1H, m), 8.46-8.33 (1H, m, Ar-H), 8.15-8.07 (1H, m, Ar-H), 7.81 (1H, *app.* d, *J* = 8.9 Hz), 7.73-7.62 (2H, m, Ar-H), 7.49 (1H, s), 7.36-7.15 (3H, m, Ar-H), 6.23 (1H, d, *J* = 8.0 Hz), 6.12 (1H, s), 6.03 (1H, d, *J* = 8.6 Hz), 4.81 (1H, *app.* t, *J* = 7.8 Hz, DABA1-α-CH), 4.73-4.62 (2H, m, Pal3-α-CH + Trp4-α-CH), 4.46-4.36 (2H, m, DABA1-β-CH + Leu3-α-CH), 3.22-2.97 (6H, m, NCH_3 + Pal3-β-CH_2 + Trp4-β-CH_2), 2.82-2.74 (1H, m, Pal3-β-CH_2), 1.78-1.69 (1H, m, Leu3-γ-CH), 1.65 (9H, s, 3× CH_3), 1.62-1.56 (1H, m, Leu3-β-CH_2), 1.50 (1H, m, Leu3-β-CH_2), 1.36 (9H, s, 3× CH_3), 1.28 (9H, s, 3× CH_3), 1.24-1.14 (3H, m, DABA1-γ-CH_3), 0.93-0.86 (6H, m, 2× Leu3-δ-CH_3). **LRMS** [*M*+H^+] 880.5. **HRMS (ESI m/z)** [*M*+H^+] calcd. for $C_{45}H_{66}N_7O_{11}$ 880.4818, found 880.4824.

(2*S*,5*S*,9*S*)-9-((1-(*tert*-butoxycarbonyl)-1*H*-indol-3-yl)methyl)-2-((*S*)-1-((*S*)-2-((*tert*-butoxycarbonyl)amino)-*N*-methyl-3-(naphthalen-2-yl)propanamido)ethyl)-5-isobutyl-12,12-dimethyl-4,7,10-trioxo-11-oxa-3,6,8-triazatridecanoic acid (S27)

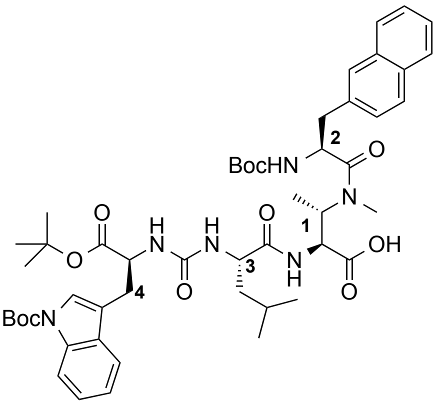

Resin-bound **S25** (60 μmol) was coupled to Boc-2-Nal-OH (38 mg, 120 μmol) using HATU (45 mg, 120 μmol) and *N*,*N*-diisopropylethylamine (33 μL, 180 μmol) in DMF (0.6 mL) according to general procedure 6. Following cleavage from the resin (general procedure 7), the peptide was purified by reverse phase HPLC (50-100% MeCN over 40 min, 10 min at 50% MeCN) to afford intermediate **S27** as a fluffy white solid (31.4 mg, 56%).

IR (ATR): 3334, 2979, 1708, 1660 cm^{-1}. **^{1}H NMR** (400 MHz, Acetone-d_6): δ 8.10 (1H, *app.* t, *J* = 7.6 Hz, Ar-H), 7.87-7.72 (4H, m, Ar-H), 7.67-7.58 (1H, m, Ar-H), 7.51-7.38 (4H, m, Ar-H), 7.29 (1H, ddd, *J* = 8.4, 7.3, 1.4 Hz, Ar-H), 7.23 (1H, *app.* td, *J* = 7.5, 1.2 Hz, Ar-H), 6.18 (1H, d, *J* = 8.1 Hz), 6.11 (1H, d, *J* = 12.7 Hz),

5.98 (1H, d, *J* = 8.8 Hz), 5.12-5.04 (1H, m, DABA1-β-CH), 4.94-4.84 (1H, m, DABA1-α-CH), 4.80 (1H, *app.* dt, *J* = 9.1, 4.6 Hz, Nal2-α-CH), 4.72-4.59 (1H, m, Trp4-α-CH), 4.50-4.32 (1H, m, Leu3-α-CH), 3.27 (1H, dd, *J* = 14.1, 4.0 Hz, Nal2-β-CH_2), 3.13 (3H, s, NCH_3), 3.11-3.01 (2H, m, Trp4-β-CH_2), 2.98-2.89 (1H, m, Nal2-β-CH_2), 1.81-1.69 (1H, m, Leu3-γ-CH), 1.68-1.58 (10H, m, 3× CH_3 + Leu3-β-CH_2), 1.57-1.45 (1H, m, Leu3-β-CH_2), 1.39-1.31 (9H, m, 3× CH_3), 1.27-1.08 (12H, m, 3× CH_3 + DABA1-γ-CH_3), 0.96-0.85 (6H, m, 2× Leu3-δ-CH_3). **LRMS** [M+H^+] 929.5. **HRMS (ESI m/z)** [M+Na^+] calcd. for $C_{50}H_{68}N_6O_{11}Na$ 951.4838, found 951.4846.

(2*S*,5*S*,9*S*)-9-((1-(*tert*-butoxycarbonyl)-1*H*-indol-3-yl)methyl)-2-((*S*)-1-((*S*)-2-((*tert*-butoxycarbonyl)amino)-3-cyclohexyl-*N*-methylpropanamido)ethyl)-5-isobutyl-12,12-dimethyl-4,7,10-trioxo-11-oxa-3,6,8-triazatridecanoic acid (S28)

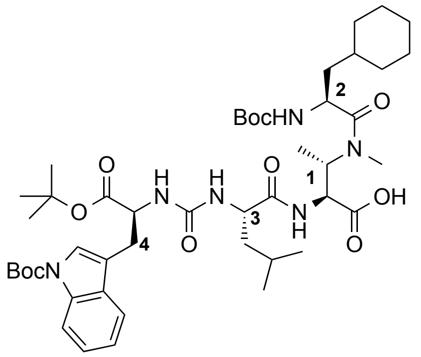

Resin-bound **S25** (60 μmol) was coupled to Boc-Cha-OH dicyclohexylamine salt (54 mg, 120 μmol) using HATU (45 mg, 120 μmol) and *N*,*N*-diisopropylethylamine (23 μL, 126 μmol) in DMF (0.6 mL) for 16 h. Following cleavage from the resin (general procedure 7), the peptide was purified by reverse phase HPLC (50-100% MeCN over 40 min, 10 min at 50% MeCN) to afford intermediate **S28** as a fluffy white solid (22 mg, 41%).

IR (ATR): 3346, 2924, 1728, 1627 cm^{-1}. **^{1}H NMR** (500 MHz, $CDCl_3$): δ 7.99 (1H, *app.* s, Ar-H), 7.86 (1H, s), 7.63-7.53 (1H, m, Ar-H), 7.41 (1H, s, Ar-H), 7.28-7.20 (2H, m, Ar-H), 6.23 (1H, s), 6.03 (1H, s), 5.64 (1H, s), 5.05-4.87 (1H, m, DABA1-β-CH), 4.79-4.66 (1H, m, Trp4-α-CH), 4.65-4.45 (2H, m, DABA1-α-CH + Leu3-α-CH), 4.45-4.15 (1H, m, Cha2-α-CH), 3.27-3.07 (2H, m, Trp4-β-CH_2), 2.98 (3H, s, NCH_3), 1.97-1.81 (1H, m, CH_2), 1.64 (12H, m, 3× CH_3 + 0.5× CH_2 + Leu-β-CH_2 + CH), 1.51-1.01 (23H, m, 6× CH_3, CH_2 + CH + DABA1-γ-CH_3), 0.95-0.65 (7H, m,

0.5× CH_2 + 2× Leu3-δ-CH_3). **LRMS** [M+H^+] 885.5. **HRMS (ESI m/z)** [M+Na^+] calcd. for $C_{45}H_{70}N_6O_{11}Na$ 907.5151, found 907.5150.

(2*S*,5*S*,9*S*)-9-((1-(*tert*-butoxycarbonyl)-1*H*-indol-3-yl)methyl)-2-((*S*)-1-((*S*)-2-((*tert*-butoxycarbonyl)amino)-2-cyclohexyl-*N*-methylacetamido)ethyl)-5-isobutyl-12,12-dimethyl-4,7,10-trioxo-11-oxa-3,6,8-triazatridecanoic acid (S29)

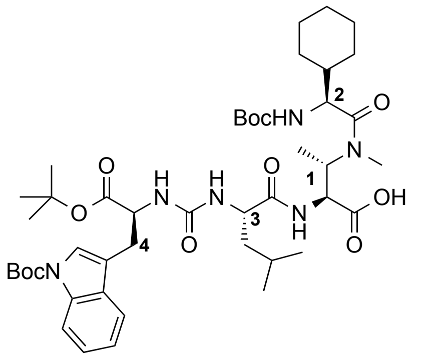

Resin-bound **S25** (60 μmol) was coupled to Boc-Chg-OH (122 mg, 480 μmol) using HATU (124 mg, 480 μmol), HOAt (324 mg, 2.4 mmol) and *N*,*N*-diisopropylethylamine (132 μL, 720 μmol) in DMF (0.6 mL) was added to resin-bound **S25** and the reaction vessel was shaken at rt for 4.5 h. Following cleavage from the resin (general procedure 7), the peptide was purified by reverse phase HPLC (50-100% MeCN over 40 min, 10 min at 50% MeCN) to afford intermediate **S29** as a fluffy white solid (19 mg, 36%).

IR (ATR): 3336, 2977, 1729, 1641, 1522 cm^{-1}. **^{1}H NMR** (500 MHz, Acetone-d_6): δ 8.12 (1H, *app.* d, J = 8.1 Hz, Ar-H), 7.72 (1H, d, J = 8.9 Hz, Ar-H), 7.71-7.65 (1H, m, Ar-H), 7.50 (1H, s, Ar-H), 7.31 (1H, ddd, J = 8.3, 7.3, 1.4 Hz, Ar-H), 7.26 (1H, *app.* td, J = 7.5, 1.2 Hz, Ar-H), 6.09 (1H, d, J = 8.1 Hz), 6.04 (1H, d, J = 9.8 Hz), 5.73 (1H, d, J = 9.3 Hz), 5.06-4.98 (1H, m, DABA1-β-CH), 4.73-4.69 (1H, m, DABA1-α-CH), 4.64 (1H, *app.* t, J = 6.5 Hz, Trp4-α-CH), 4.41-4.31 (2H, m, Chg2-α-CH + Leu3-α-CH), 3.23-3.06 (2H, m, Trp4-β-CH_2), 3.01 (3H, s, NCH_3), 1.75-1.70 (1H, m, Leu3-γ-CH), 1.67 (9H, s, 3× CH_3), 1.62-1.52 (2H, m, Leu3-β-CH_2 + CH_2), 1.52-1.44 (2H, m, Leu3-β-CH_2 + CH_2), 1.32-1.14 (7H, m, DABA1-γ-CH_3 + 2× CH_2), 1.14-1.02 (3H, m), 1.02-0.94 (1H, m), 0.95-0.87 (6H, m, 2× Leu3-δ-CH_3). **LRMS** [M+H^+] 871.5. **HRMS (ESI m/z)** [M+Na^+] calcd. for $C_{45}H_{70}N_6O_{11}Na$ 893.4994, found 893.5001.

(2*S*,5*S*,9*S*)-9-((1-(*tert*-butoxycarbonyl)-1*H*-indol-3-yl)methyl)-2-((*S*)-1-(2-((*tert*-butoxycarbonyl)amino)-*N*-methylacetamido)ethyl)-5-isobutyl-12,12-dimethyl-4,7,10-trioxo-11-oxa-3,6,8-triazatridecanoic acid (S30)

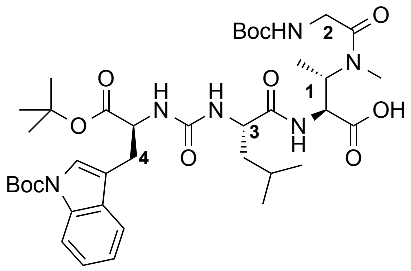

Isopeptide **S17** (77 mg, 139 μmol) was loaded onto 2-chlorotrityl chloride resin (58 mg, 70 μmol) in CH_2Cl_2 (1 mL) using *N*,*N*-diisopropylethylamine (49 μL, 280 μmol) and Fmoc-deprotected according to general procedure 2. Fmoc-L-Leu-OH (98 mg, 280 μmol) was subsequently coupled using standard Fmoc-SPPS procedure using PyBOP (145 mg, 280 μmol) and NMM (57 μL, 560 μmol) in DMF (1.1 mL) (general procedure 3). Carbamate **S12** (74 mg, 140 μmol) was subsequently coupled according to general procedure 4. Following cleavage from the resin (general procedure 7), the peptide was purified by reverse phase HPLC (50-100% MeCN over 40 min, 10 min at 50% MeCN) to afford depsipeptide **S30** as a fluffy white solid (14.5 mg, 27%).

IR (ATR): 3359, 2977, 2934, 1729, 1640 cm^{-1}. **^{1}H NMR** (400 MHz, Acetone-d_6, *major rotamer*): δ 8.13 (1H, *app.* d, *J* = 8.0 Hz, Ar-H), 7.90 (0.2 H, d, *J* = 8.0 Hz), 7.73-7.68 (1H, m, Ar-H), 7.53 (1H, s, Ar-H), 7.36-7.25 (2H, m, Ar-H), 6.10 (1H, d, *J* = 13.2 Hz), 5.98-5.81 (1H, m), 5.05-4.93 (1H, m, DABA1-β-CH), 4.78-4.60 (2H, m, DABA1-α-CH + Trp4-α-CH), 4.42-4.32 (1H, m, Leu3-α-CH), 3.94-3.82 (1H, m, Gly2-α-CH_2), 3.80-3.66 (1H, m, Gly2-α-CH_2), 3.24-3.09 (2H, m, Trp4-β-CH_2), 2.83 (3H, s, NCH_3), 1.78-1.55 (11H, m, 3× CH_3 + Leu3-β-CH_2 + Leu3-γ-CH), 1.53-1.34 (19H, m, 6× CH_3 + Leu3-β-CH_2), 1.20 (3H, d, *J* 7.1 Hz, DABA1-γ-CH_3), 0.97-0.83 (6H, m, 2× Leu3-δ-CH_3). **HRMS (ESI m/z)** [*M*+Na^+] calcd. for $C_{39}H_{60}N_6O_{11}Na$ 811.4212, found 811.4227.

(2*S*,5*S*,9*S*)-9-((1-(*tert*-butoxycarbonyl)-1*H*-indol-3-yl)methyl)-2-((*S*)-1-((*S*)-2-((*tert*-butoxycarbonyl)amino)-*N*-methylpropanamido)ethyl)-5-isobutyl-12,12-dimethyl-4,7,10-trioxo-11-oxa-3,6,8-triazatridecanoic acid (S31)

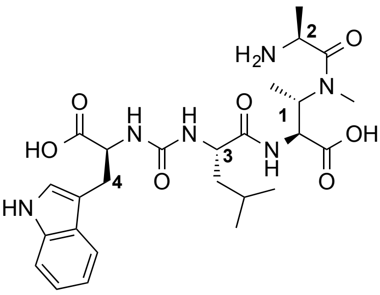

Resin-bound **S25** (60 μmol) was coupled to Boc-Ala-OH (23 mg, 120 μmol) using HATU (45 mg, 120 μmol) and *N*,*N*-diisopropylethylamine (22 μL, 120 μmol) in DMF (0.6 mL) according to general procedure 6. Following cleavage from the resin (general procedure 7), the peptide was purified by column chromatography (95:5 v/v CH_2Cl_2: MeOH → 9:1 v/v CH_2Cl_2: MeOH, 1% vol.% AcOH) to afford intermediate **S31** as a white foam (21 mg, 43%).

IR (ATR): 3331, 2978, 1644, 1534 cm^{-1}. **^{1}H NMR** (400 MHz, Acetone-d_6, *major rotamer*): δ 8.12 (1H, d, *J* = 8.0 Hz, Ar-H), 7.80-7.59 (1H, m, Ar-H), 7.51 (1H, s, Ar-H), 7.34-7.24 (2H, m, Ar-H), 6.16-6.01 (2H, m, 2× NH), 5.89 (1H, d, *J* = 8.3 Hz, NH), 4.99 (1H, *app.* t, *J* = 7.1 Hz, DABA1-β-CH), 4.74 (1H, *app.* dd, *J* = 9.0, 7.0 Hz, DABA1-α-CH), 4.70-4.58 (1H, m, Trp4-α-CH), 4.48-4.31 (2H, m, Ala2-α-CH + Leu3-α-CH), 3.23-3.07 (2H, m, Trp4-β-CH_2), 2.95 (3H, s, NCH_3), 1.78-1.68 (1H, m, Leu3-γ-CH), 1.66 (9H, s, 3× CH_3), 1.61-1.53 (1H, m, Leu3-β-CH_2), 1.51-1.44 (1H, m, Leu3-β-CH_2), 1.42-1.28 (18H, m, 6× CH_3), 1.23-1.13 (6H, m, DABA1-γ-CH_3 + Ala2-β-CH_3), 0.94-0.83 (6H, m, 2× Leu3-δ-CH_3). **LRMS** [M+H^+] 803.5. **HRMS (ESI m/z)** [M+Na^+] calcd. for $C_{40}H_{62}N_6O_{11}Na$ 825.4368, found 825.4379.

(2*S*,5*S*,9*S*)-2-((*S*)-1-((*S*)-4-(*tert*-butoxy)-2-((*tert*-butoxycarbonyl)amino)-*N*-methyl-4-oxobutanamido)ethyl)-9-((1-(*tert*-butoxycarbonyl)-1*H*-indol-3-yl)methyl)-5-isobutyl-12,12-dimethyl-4,7,10-trioxo-11-oxa-3,6,8-triazatridecanoic acid (S32)

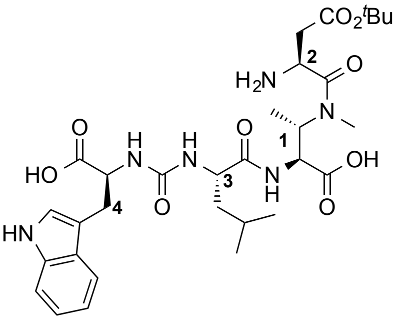

Isopeptide **S18** (90 mg, 144 μmol) was loaded onto 2-chlorotrityl chloride resin (59 mg, 72 μmol) in CH_2Cl_2 (1 mL) using *N*,*N*-diisopropylethylamine (50 μL, 288 μmol) and Fmoc-deprotected according to general procedure 2. Fmoc-Leu-OH (102 mg, 288 μmol) was subsequently coupled using PyBOP (150 mg, 288 μmol) and NMM (59 μL, 576 μmol) in DMF (0.7 mL) (general procedure 3). Carbamate **S12** (76 mg, 144 μmol) was subsequently coupled according to general procedure 4. Following cleavage from the resin (general procedure 7), the peptide was purified by reverse phase HPLC (50-100% MeCN over 40 min, 10 min at 50% MeCN) to afford depsipeptide **S32** as a fluffy white solid (18.4 mg, 28%).

IR (ATR): 3328, 2977, 2933, 1728, 1638 cm^{-1}. **^{1}H NMR** (400 MHz, $CDCl_3$): δ 8.06 (1H, *app.* d, *J* = 8.1 Hz, Ar-H), 7.63 (1H, br. s), 7.53 (1H, *app.* d, *J* = 7.7 Hz, Ar-H), 7.41 (1H, s, Ar-H), 7.33-7.16 (2H, m, Ar-H), 5.77 (1H, s, NH), 5.62 (1H, s, NH), 4.93-4.76 (2H, m, DABA1-α-CH + Asp2-α-CH), 4.69-4.48 (2H, m, DABA1-β-CH + Trp4-α-CH), 4.26-4.14 (1H, m, Leu3-α-CH), 3.19-3.08 (2H, m, Trp4-β-CH_2), 2.64 (1H, dd, *J* = 15.5, 5.0 Hz, Asp2-β-CH_2), 2.36 (1H, dd, *J* = 15.2, 7.9 Hz, Asp2-β-CH_2), 1.69-1.45 (12H, m, 3× CH_3 + Leu3-β-CH_2 + Leu3-γ-CH), 1.40 (9H, s, 3× CH_3), 1.33 (9H, s, 3× CH_3), 1.22 (3H, d, *J* = 6.9 Hz, DABA1-γ-CH_3), 0.93-0.79 (6H, m, 2× Leu3-δ-CH_3). **LRMS** [$M+H^+$] 903.6. **HRMS (ESI m/z)** [$M+Na^+$] calcd. for $C_{45}H_{70}N_6O_{13}Na$ 925.4893, found 925.4898.

(2*S*,5*S*,9*S*)-2-((*S*)-1-((*S*)-2,6-bis((*tert*-butoxycarbonyl)amino)-*N*-methylhexanamido)ethyl)-9-((1-(*tert*-butoxycarbonyl)-1*H*-indol-3-yl)methyl)-5-isobutyl-12,12-dimethyl-4,7,10-trioxo-11-oxa-3,6,8-triazatridecanoic acid (S33)

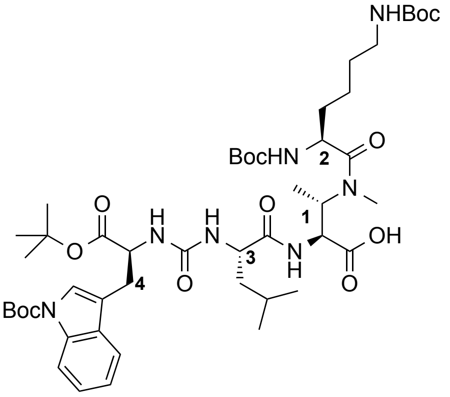

Isopeptide **S19** (279 mg, 410 μmol) was loaded onto 2-chlorotrityl chloride resin (168 mg, 205 μmol) in CH_2Cl_2 (2.8 mL) using *N*,*N*-diisopropylethylamine (142 μL, 820 μmol) and Fmoc-deprotected according to general procedure 2. Fmoc-L-Leu-OH (102 mg, 820 μmol) was subsequently coupled using PyBOP (426 mg, 820 μmol) and NMM (162 μL, 1.64 mmol) in DMF (2 mL) (general procedure 3). Carbamate **S12** (218 mg, 410 μmol) was subsequently coupled according to general procedure 4. Following cleavage from the resin (general procedure 7), the peptide was purified by reverse phase HPLC (50-100% MeCN over 40 min, 10 min at 50% MeCN) to afford depsipeptide **S33** as a fluffy white solid (44.2 mg, 22%).

IR (ATR): 3307, 2976, 1730, 1650 cm^{-1}. **^{1}H NMR** (400 MHz, $CDCl_3$): δ 8.09 (1H, *app.* d, *J* = 7.7 Hz, Ar-H), 7.78-7.50 (1H, m, Ar-H), 7.41 (1H, s, Ar-H), 7.33-7.22 (2H, m, Ar-H), 7.11 (1H, s), 6.65 (1H, s), 5.87-5.49 (3H, m, NH), 5.06-4.92 (1H, m, DABA1-β-CH), 4.87-4.42 (3H, m, DABA1-α-CH + Lys2-α-CH + Trp4-α-CH), 4.39-4.29 (1H, m, Leu3-α-CH), 3.27-2.99 (4H, m, Lys2-ε-CH_2 + Trp4-β-CH_2), 2.94 (3H, s, NCH_3), 1.91-1.24 (45H, m, 12× CH_3 + Leu3-β-CH_2 + Leu3-γ-CH + Lys2-β-CH_2 + Lys2-γ-CH_2 + Lys2-δ-CH_2), 1.24-1.14 (3H, m, DABA1-γ-CH_3), 0.97-0.85 (6H, m, 2× Leu3-δ-CH_3). **LRMS** [M+H^+] 960.1. **HRMS (ESI m/z)** [M+Na^+] calcd. for $C_{48}H_{77}N_7O_{13}Na$ 982.5472, found 982.5479.

(2*S*,5*S*,9*S*)-9-((1-(*tert*-butoxycarbonyl)-1*H*-indol-3-yl)methyl)-2-((*S*)-1-((2*S*,3*R*)-2-((*tert*-butoxycarbonyl)amino)-3-hydroxy-*N*-methylbutanamido)ethyl)-5-isobutyl-12,12-dimethyl-4,7,10-trioxo-11-oxa-3,6,8-triazatridecanoic acid (S34)

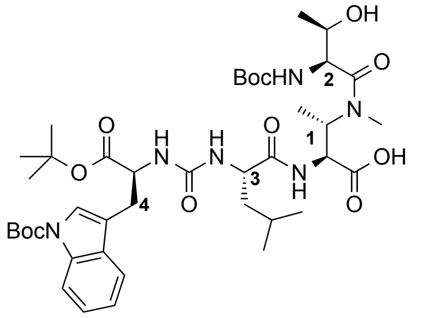

Isopeptide **S21** (89 mg, 160 μmol) was loaded onto 2-chlorotrityl chloride resin (66 mg, 80 μmol) in CH_2Cl_2 (1.1 mL) using *N*,*N*-diisopropylethylamine (55 μL, 320 μmol) and Fmoc-deprotected according to general procedure 2. Fmoc-L-Leu-OH (114 mg, 320 μmol) was subsequently coupled using PyBOP (167 mg, 320 μmol) and NMM (65 μL, 640 μmol) in DMF (0.8 mL) (general procedure 3). Carbamate **S12** (85 mg, 160 μmol) was subsequently coupled according to general procedure 4. Following cleavage from the resin (general procedure 7), the peptide was purified by reverse phase HPLC (50-100% MeCN over 40 min, 10 min at 50% MeCN) to afford depsipeptide **S34** as a fluffy white solid (14 mg, 21%).

IR (ATR): 3364, 2977, 1729, 1641 cm^{-1}. **^{1}H NMR** (400 MHz, $CDCl_3$): δ 8.09 (1H, *app.* d, *J* = 8.2 Hz, Ar-H), 7.69 (1H, *app.* d, *J* = 8.8 Hz), 7.56 (1H, *app.* d, *J* = 7.7 Hz, Ar-H), 7.40 (1H, s, Ar-H), 7.32-7.17 (2H, m, Ar-H), 5.90 (1H, s, NH), 5.70 (1H, s, NH), 5.61 (1H, s, NH), 5.20-4.91 (1H, m, DABA1-α-CH), 4.77-4.59 (2H, m, DABA1-β-CH + Trp4-α-CH), 4.54 (1H, *app.* d, *J* 9.4 Hz, Thr2-α-CH), 4.19-4.10 (2H, m, Thr2-β-CH + Leu3-α-CH), 3.21-3.12 (2H, m, Trp4-β-CH_2), 2.99 (3H, s, NCH_3), 1.71-1.46 (12H, m, 3× CH_3 + Leu3-β-CH_2 + Leu3-γ-CH), 1.41 (9H, s, 3× CH_3), 1.35 (9H, s, 3× CH_3), 1.26-1.14 (6H, m, DABA1-γ-CH_3 + Thr2-γ-CH_3), 0.90 (3H, d, *J* = 6.1 Hz, Leu3-γ-CH_3), 0.84 (3H, d, *J* = 6.0 Hz, Leu3-δ-CH_3). **LRMS** [M+H^+] 833.5. **HRMS (ESI m/z)** [M+Na^+] calcd. for $C_{41}H_{64}N_6O_{12}Na$ 855.8474, found 855.8470.

(2*S*,3*S*)-methyl 2-((((9*H*-fluoren-9-yl)methoxy)carbonyl)amino)-3-(2-((*tert*-butoxycarbonyl)amino)-*N*-methylacetamido)butanoate (S35)

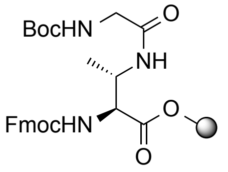

Amino acid **20** (219 mg, 805 μmol) was loaded onto 2-chlorotrityl chloride resin (514 mg, 730 μmol) in CH_2Cl_2 (2 mL) using *N*,*N*-diisopropylethylamine (500 μL, 400 μmol) according to general procedure 2. Allyl carbamate was subsequently removed using *tetrakis*(triphenylphosphine) palladium(0) (186 mg, 161 μmol) and phenylsilane (2.0 mL, 16.1 mmol) according to general procedure 5. Boc-Gly-OH (256 mg, 1.46 mmol) was coupled using solid-phase isopeptide formation (General procedure 6) with HATU (554 mg, 160 μmol) and *N*,*N*-diisopropylethylamine (381 μL, 2.1 mmol) in DMF (1.4 mL) to afford the resin-bound isopeptide **S35.**

(2*S*,5*S*,9*S*)-9-((1-(*tert*-butoxycarbonyl)-1*H*-indol-3-yl)methyl)-2-((*S*)-1-(2-((*tert*-butoxycarbonyl)amino)-*N*-methylacetamido)ethyl)-5-((*R*)-*sec*-butyl)-12,12-dimethyl-4,7,10-trioxo-11-oxa-3,6,8-triazatridecanoic acid (S36)

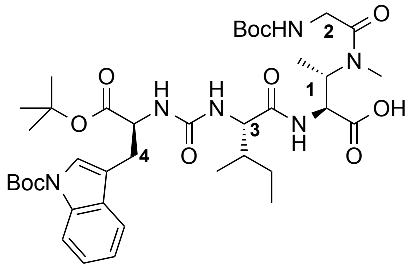

Resin-bound isopeptide **S35** (60 μmol) was Fmoc-deprotected (general procedure 2) and coupled to Fmoc-Ile-OH (85 mg, 240 μmol) using PyBOP (125 mg, 240 μmol) and NMM (50 μL, 480 μmol) in DMF (0.6 mL) according to general procedure 3. Carbamate **S12** (64 mg, 120 μmol) was subsequently coupled according to general procedure 4. Following cleavage from the resin (general procedure 7), the peptide was purified by reverse phase HPLC (50-100% MeCN over 40 min, 10 min at 50% MeCN) to afford depsipeptide **S36** as a fluffy white solid (17 mg, 36%).

IR (ATR): 3308, 2978, 2929, 1726, 1637 cm^{-1}. **^{1}H NMR** (500 MHz, Acetone-d_6, *major rotamer*): δ 8.12 (1H, d, *J* = 7.9 Hz), 7.80 (1H, *app.* d, *J* = 8.9 Hz, Ar-H), 7.69 (1H, d, *J* = 7.3 Hz), 7.59 (1H, *app.* d, *J* = 8.6 Hz, Ar-H), 7.51 (1H, s, Ar-H), 7.31 (1H, *app.* t, *J* = 7.7 Hz, Ar-H), 7.27 (1H, *app.* t, *J* = 7.5 Hz, Ar-H), 6.17-6.01 (2H, m),

5.85-5.75 (1H, m), 5.01-4.91 (1H, m, DABA1-β-CH), 4.75 (1H, *app.* t, J = 7.2 Hz, DABA1-α-CH), 4.64 (1H, *app.* t, J = 7.3 Hz, Trp4-α-CH), 4.31-4.19 (1H, m, Ile3-α-CH), 3.92-3.83 (1H, m, Gly2-α-CH_2), 3.75-3.68 (1H, m, Gly2-α-CH_2), 3.20-3.07 (2H, m, Trp4-β-CH_2), 2.85 (3H, s, NCH_3), 1.87-1.72 (1H, m, Ile3-β-CH), 1.67 (9H, s, 3× CH_3), 1.60-1.49 (1H, m, Ile3-γ-CH_2), 1.44-1.33 (18H, s, 6× CH_3), 1.21 (3H, d, J = 6.9 Hz, DABA1-γ-CH_3), 1.17-1.07 (1H, m, Ile3-γ-CH_2), 0.96-0.76 (6H, m, 2× CH_3). **LRMS** [M+H^+] 789.4. **HRMS (ESI m/z)** [M+Na^+] calcd. for $C_{39}H_{60}N_6O_{11}Na$ 811.4212, found 811.4227.

(2*S*,5*S*,9*S*)-9-((1-(*tert*-butoxycarbonyl)-1*H*-indol-3-yl)methyl)-2-((*S*)-1-(2-((*tert*-butoxycarbonyl)amino)-*N*-methylacetamido)ethyl)-5-isopropyl-12,12-dimethyl-4,7,10-trioxo-11-oxa-3,6,8-triazatridecan-1-oic acid (S37)

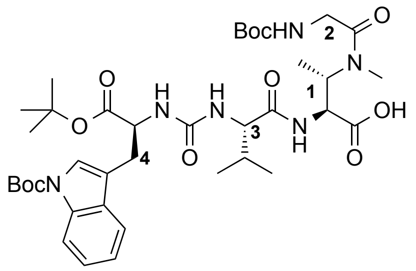

Resin-bound isopeptide **S35** (45 μmol) was Fmoc-deprotected (general procedure 2) and coupled to Fmoc-Val-OH (81.5 mg, 240 μmol) using PyBOP (125 mg, 240 μmol) and NMM (53 μL, 480 μmol) in DMF (0.45 mL) according to general procedure 6. Carbamate **S12** (63 mg, 120 μmol) was subsequently coupled according to general procedure 4. Following cleavage from the resin (general procedure 7), the peptide was purified by reverse-phase HPLC (50 to 100% MeCN over 40 min) to afford depsipeptide **S37** as a fluffy white solid (19.7 mg, 53%).

IR (ATR): 3034, 1735, 1639, 1384 cm^{-1}. **^{1}H NMR** (400 MHz, (Acetone-d_6, *major rotamer*): δ 8.14 (1H, d, J = 8.3 Hz, Ar-H), 7.70 (1H, d, J = 7.4 Hz, Ar-H), 7.52 (1H, s, Ar-H), 7.35-7.26 (2H, m, 2x Ar-H), 6.21–6.13 (2H, m, 2x N-H), 5.86 (1H, m, N-H), 5.04–4.97 (1H, m, DABA1-β-CH), 4.77 (1H, m, DABA1-α-CH), 4.69 (1H, m, Trp4-α-CH), 4.26 (1H, m, Val3-α-CH), 4.05–3.72 (2H, m, Gly2-α-CH_2), 3.17 (2H, d, J = 6.0 Hz, Trp4-β-CH_2), 2.88 (3H, s, NCH_3), 2.08 (1H, m, Val3-β-CH), 1.68 (9H, s, 3x CH_3), 1.42 (18H, m, 6x CH_3), 1.24 (3H, d, J = 7.0 Hz, DABA1-γ-CH_3), 0.95 (3H, d, J = 6.7 Hz, Val3-γ-CH_3), 0.91 (3H, d, J = 6.8 Hz, Val3-γ-CH_3). **LRMS** [M^+H^+]

775.5. **HRMS (ESI m/z)** [M+Na^+] calcd. for $C_{38}H_{58}N_6O_{11}Na$ 797.4061, found 797.4063.

(2*S*,5*S*,9*S*)-9-((1-(*tert*-butoxycarbonyl)-1*H*-indol-3-yl)methyl)-2-((*S*)-1-(2-((*tert*-butoxycarbonyl)amino)-*N*-methylacetamido)ethyl)-12,12-dimethyl-4,7,10-trioxo-5-pentyl-11-oxa-3,6,8-triazatridecanoic acid (S38)

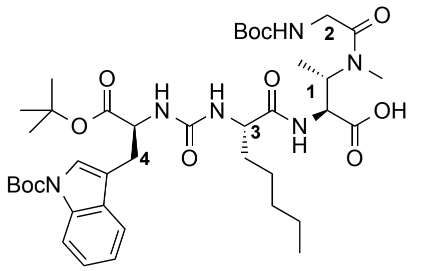

Resin-bound isopeptide **S35** (70 μmol) was Fmoc-deprotected (general procedure 2) and coupled to Fmoc-Aha-OH (103 mg, 280 μmol) using PyBOP (144 mg, 280 μmol) and NMM (58 μL, 560 μmol) in DMF (0.7 mL) according to general procedure 3. Carbamate **S12** (75 mg, 140 μmol) was subsequently coupled according to general procedure 4. Following cleavage from the resin (general procedure 7), the peptide was purified by column chromatography (98:2 v/v CH_2Cl_2: MeOH → 9:1 v/v CH_2Cl_2: MeOH, 0.5 vol.% AcOH) to afford intermediate **S38** as a white foam (30 mg, 53%).

IR (ATR): 3356, 2977, 2932, 1731, 1638 cm^{-1}. **^{1}H NMR** (500 MHz, Acetone-d_6, *major rotamer*): δ 8.12 (1H, d, J = 8.2 Hz, Ar-H), 7.69 (1H, d, J = 7.6 Hz, Ar-H), 7.60 (1H, d, J = 8.5 Hz), 7.52 (1H, s, Ar-H), 7.33-7.24 (2H, m, Ar-H), 6.19-6.01 (1H, m), 5.86-5.74 (1H, m), 5.04-4.91 (1H, m, DABA1-β-CH), 4.80-4.68 (1H, m, DABA1-α-CH), 4.64 (1H, *app.* t, J = 6.4 Hz, Trp4-α-CH), 4.30 (1H, *app.* t, J = 6.8 Hz, Aha3-α-CH), 3.90-3.80 (1H, m, Gly2-α-CH_2), 3.79-3.66 (1H, m, Gly2-α-CH_2), 3.19-3.10 (2H, m, Trp4-β-CH_2), 2.83 (3H, s, NCH_3), 1.76 (1H, dd, J = 16.3, 8.3 Hz, Aha3-β-CH_2), 1.67 (9H, s, 3× CH_3), 1.61-1.48 (1H, m, Aha3-β-CH_2), 1.45-1.33 (20H, s, 6× CH_3 + CH_2), 1.32-1.24 (4H, m, 2× CH_2), 1.20 (3H, d, J = 7.1 Hz, DABA1-γ-CH_3), 0.90-0.81 (3H, m). **LRMS** [M+H^+] 803.5. **HRMS (ESI m/z)** [M+Na^+] calcd. for $C_{40}H_{62}N_6O_{11}Na$ 825.4368, found 825.4364.

(2*S*,5*S*,9*S*)-9-((1-(*tert*-butoxycarbonyl)-1*H*-indol-3-yl)methyl)-2-((*S*)-1-(2-((*tert*-butoxycarbonyl)amino)-*N*-methylacetamido)ethyl)-5-cyclohexyl-12,12-dimethyl-4,7,10-trioxo-11-oxa-3,6,8-triazatridecanoic acid (S39)

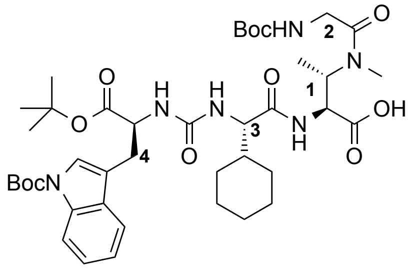

Resin-bound isopeptide **S35** (60 μmol) was Fmoc-deprotected (general procedure 2) and coupled to Fmoc-Chg-OH (91 mg, 240 μmol) using PyBOP (124 mg, 240 μmol) and NMM (50 μL, 480 μmol) in DMF (0.6 mL) according to general procedure 3. Carbamate **S12** (64 mg, 120 μmol) was subsequently coupled according to general procedure 4 in the presence of *N*,*N*-diisopropylethylamine (21 μL, 120 μmol) in DMF (1 mL). Following cleavage from the resin (general procedure 7), the peptide was purified by reverse phase HPLC (50-100% MeCN over 40 min, 10 min at 50% MeCN) to afford depsipeptide **S39** as a fluffy white solid (20 mg, 40%).

IR (ATR): 3308, 2979, 2929, 1726, 1637 cm^{-1}. **^{1}H NMR** (400 MHz, Acetone-d_6, *major rotamer*): δ 8.11 (1H, *app.* d, J = 8.1 Hz, Ar-H), 7.74-7.60 (1H, m, Ar-H), 7.50 (1H, s, Ar-H), 7.36-7.22 (2H, m, Ar-H), 6.25-6.12 (2H, m), 5.90-5.79 (1H, m), 5.06-4.91 (1H, m, DABA1-β-CH), 4.81-4.70 (1H, m, DABA1-α-CH), 4.70-4.58 (1H, m, Trp4-α-CH), 4.29-4.19 (1H, m, Chg3-α-CH), 3.90-3.81 (1H, m, Gly2-α-CH_2), 3.77-3.66 (1H, m, Gly2-α-CH_2), 3.17-3.06 (2H, m, Trp4-β-CH_2), 1.79-1.54 (15H, 3× CH_3 + 2.5× CH_2 + Chg3-β-CH), 1.43-1.32 (18H, m, 6× CH_3), 1.25-0.93 (8H, m, DABA1-γ-CH_3 + 2.5× CH_2). **LRMS** [M+H^+] 815.4. **HRMS (ESI m/z)** [M+H^+] calcd. for $C_{41}H_{62}N_6O_{11}$ 815.4549, found 815.4548.

(2*S*,5*S*,9*S*)-9-((1-(*tert*-butoxycarbonyl)-1*H*-indol-3-yl)methyl)-2-((*S*)-1-(2-((*tert*-butoxycarbonyl)amino)-*N*-methylacetamido)ethyl)-5-(cyclohexylmethyl)-12,12-dimethyl-4,7,10-trioxo-11-oxa-3,6,8-triazatridecanoic acid (S40)

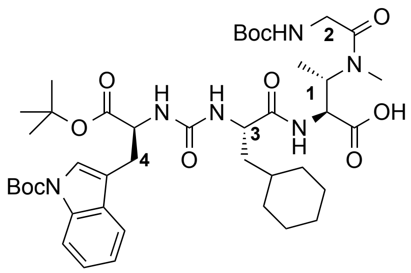

Resin-bound isopeptide **S35** (60 µmol) was Fmoc-deprotected (general procedure 2) and coupled to Fmoc-Cha-OH (47 mg, 120 µmol) using HATU (45 mg, 120 µmol) and *N*,*N*-diisopropylethylamine (33 µL, 180 µmol) in DMF (0.6 mL) according to general procedure 6. Carbamate **S12** (64 mg, 120 µmol) was subsequently coupled according to general procedure 4. Following cleavage from the resin (general procedure 7), the peptide was purified by reverse phase HPLC (50-100% MeCN over 60 min, 10 min at 50% MeCN) to afford depsipeptide **S40** as a fluffy white solid (17 mg, 34%).

IR (ATR): 3390, 2977, 2936, 1730, 1644 cm^{-1}. **^{1}H NMR** (500 MHz, Acetone-d_6, rotamers in 4:1 ratio, *major rotamer*): δ 8.12 (1H, *app.* d, *J* = 8.2 Hz, Ar-H), 7.72-7.66 (1H, m), 7.61 (1H, *app.* d, *J* = 8.9 Hz, Ar-H), 7.52 (1H, s, Ar-H), 7.32 (1H, ddd, *J* = 8.3, 7.1, 1.4 Hz, Ar-H), 7.27 (1H, *app.* td, *J* = 7.5, 1.2 Hz, Ar-H), 6.16-5.97 (2H, m), 5.89-5.69 (1H, m), 5.00-4.93 (1H, m, DABA1-β-CH), 4.79-4.70 (1H, m, DABA1-α-CH), 4.68-4.60 (1H, m, Trp4-α-CH), 4.42-4.35 (1H, m, Cha3-α-CH), 3.96 (1H, t, *J* 5.5 Hz), 3.87-3.81 (1H, m, Gly2-α-CH_2), 3.75-3.69 (1H, m, Gly2-α-CH_2), 3.19-3.11 (2H, m, Trp4-β-CH_2), 2.82 (3H, s, NCH_3), 1.84-1.75 (1H, m, CH_2), 1.67-1.56 (13H, m, 3× CH_3 + 1.5× CH_2 + Cha3-β-CH_2), 1.48-1.31 (22H, m, 6× CH_3 + Cha3-β-CH_2 + Cha3-γ-CH + CH_2), 1.24-1.08 (5H, m, DABA1-γ-CH_3 + CH_2), 1.00-0.77 (2H, m, CH_2). **LRMS** [M+H^+] 829.5. **HRMS (ESI m/z)** [M+Na^+] calcd. for $C_{42}H_{64}N_6O_{11}Na$ 851.5425, found 851.4526.

(2*S*,5*S*,9*S*)-9-((1-(*tert*-butoxycarbonyl)-1*H*-indol-3-yl)methyl)-2-((*S*)-1-(2-((*tert*-butoxycarbonyl)amino)-*N*-methylacetamido)ethyl)-5-((*S*)-1-hydroxyethyl)-12,12-dimethyl-4,7,10-trioxo-11-oxa-3,6,8-triazatridecan-1-oic acid (S41)

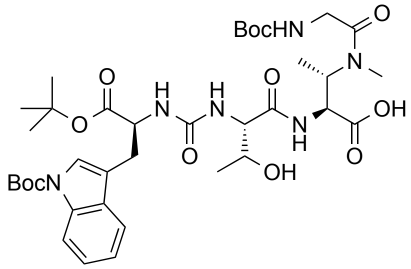

Resin-bound isopeptide **S35** (40 µmol) was Fmoc-deprotected (general procedure 2) and double coupled to Fmoc-Thr-OH (13.7 mg, 80 µmol) using HATU (30.4 mg, 80 µmol) and *N,N*-diisopropylethylamine (20 µL, 120 µmol) in DMF (0.4 mL) according to general procedure 6. Carbamate **S12** (46 mg, 90 µmol) was subsequently coupled according to general procedure. Following cleavage from the resin (general procedure 7), the peptide was purified by reverse-phase HPLC (50 to 100% MeCN over 40 min) to afford depsipeptide **S41** as a fluffy white solid (18.2 mg, 49%).

IR (ATR): 3004, 1709, 1422, 1359 cm^{-1}. **^{1}H NMR** (400 MHz, Acetone-d_6, *major rotamer*): δ 8.08 (1H, d, *J* = 8.1 Hz, Ar-H), 7.66 (1H, d, *J* = 8.6 Hz, Ar-H), 7.49 (1H, s, Ar-H), 7.30-7.21 (2H, m, 2x Ar-H), 6.37 (1H, m, N-H), 6.24 (1H, m, N-H), 5.85 (1H, m, N-H), 4.97 (1H, m, DABA1-β-CH), 4.69 (1H, m, DABA1-α-CH), 4.63 (1H, m, Trp4-α-CH), 4.24 (2H, m, Thr3-α-CH + Thr3-β-CH), 3.92–3.77 (2H, m, Gly2-α-CH_2), 3.14 (2H, m, Trp4-β-CH_2), 2.80 (3H, s, NCH_3), 1.63 (9H, s, 3x CH_3), 1.38–1.34 (18H, m, 6x CH_3), 1.18 (3H, d, *J* = 7.1 Hz, DABA1-γ-CH_3), 1.08 (3H, d, *J* = 6.4 Hz, Thr3-γ-CH_3). **LRMS** [*M*+H^+] 777.5. **HRMS (ESI m/z)** [*M*+Na^+] calcd. for $C_{37}H_{56}N_6O_{12}Na$ 799.3853, found 799.3850.

(2*S*,5*S*,9*S*)-5-(2-(*tert*-butoxy)-2-oxoethyl)-9-((1-(*tert*-butoxycarbonyl)-1*H*-indol-3-yl)methyl)-2-((*S*)-1-(2-((*tert*-butoxycarbonyl)amino)-*N*-methylacetamido)ethyl)-12,12-dimethyl-4,7,10-trioxo-11-oxa-3,6,8-triazatridecan-1-oic acid (S42)

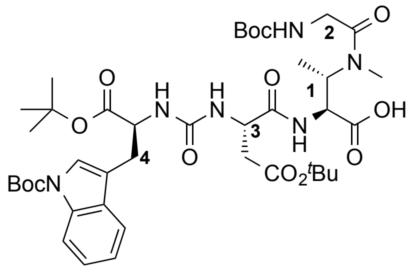

Resin-bound isopeptide **S35** (60 µmol) was Fmoc-deprotected (general procedure 2) and coupled to Fmoc-Asp(O^{t}Bu)-OH (99 mg, 240 µmol) using PyBOP (125 mg, 240 µmol) and NMM (53 µL, 480 µmol) in DMF (0.6 mL) according to general procedure 6. Carbamate **S12** (62 mg, 120 µmol) was subsequently coupled according to general procedure 4. Following cleavage from the resin (general procedure 7), the peptide was purified by column chromatography (eluent 95:5 v/v CH_2Cl_2: MeOH, 0.1 vol.% AcOH) to afford depsipeptide **S42** as a yellow oil (37.2 mg, 69%).

IR (ATR): 3359, 2978, 2933, 1726, 1640 cm^{-1}. **^{1}H NMR** (300 MHz, Acetone-d_6, *major rotamer*): δ 8.11 (1H, d, J = 7.8 Hz, Ar-H), 7.79 (1H, d, J = 6.9 Hz, Ar-H), 7.52 (1H, s, Ar-H), 7.34-7.25 (2H, m, 2x Ar-H), 6.36-6.24 (2H, m, 2x N-H), 5.83 (1H, m, N-H), 4.93 (1H, m, DABA1-β-CH), 4.74-4.63 (3H, m, DABA1-α-CH + Asp3-α-CH + Trp4-α-CH), 3.94–3.66 (2H, m, Gly2-α-CH_2), 3.17 (2H, d, J = 6.3 Hz, Trp4-β-CH_2), 2.75 (3H, s, NCH_3), 2.67–2.60 (2H, m, Asp3-β-CH_2), 1.66 (9H, m, 3x CH_3), 1.41–1.38 (27H, m, 9x CH_3), 1.10 (3H, d, J = 6.9 Hz, DABA1-γ-CH_3). **LRMS** [M+H$^+$] 847.6. **HRMS (ESI m/z)** [M+Na$^+$] calcd. for $C_{41}H_{62}N_6O_{13}Na$ 869.4272, found 869.4272.

(2*S*,5*S*,9*S*)-9-((1-(*tert*-butoxycarbonyl)-1*H*-indol-3-yl)methyl)-2-((*S*)-1-(2-((*tert*-butoxycarbonyl)amino)-*N*-methylacetamido)ethyl)-5-(4-((*tert*-butoxycarbonyl) amino)butyl)-12,12-dimethyl-4,7,10-trioxo-11-oxa-3,6,8-triazatridecan-1-oic acid (S43)

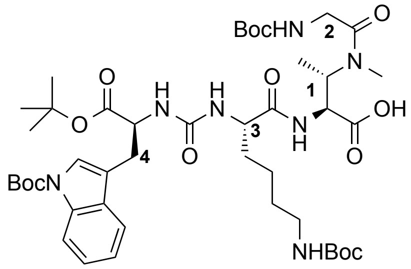

Resin-bound isopeptide **S35** (45 µmol) was Fmoc-deprotected (general procedure 2) and coupled to Fmoc-Lys(Boc)-OH (112 mg, 240 µmol) using PyBOP (125 mg, 240 µmol) and NMM (53 µL, 480 µmol) in DMF (0.6 mL) according to general procedure 6. Carbamate **S12** (63 mg, 126µmol) was subsequently coupled according to general procedure 4. Following cleavage from the resin (general procedure 6), the peptide was purified by column chromatography (97:3 v/v CH_2Cl_2: *i*-PrOH, 0.1 vol.% acetic acid - 9:1 v/v CH_2Cl_2: *i*-PrOH, 0.1% acetic acid) to afford depsipeptide **S43** as a white foam (15.4 mg, 38%).

IR (ATR): 3325, 2977, 1701, 1647 cm^{-1}. **^{1}H NMR** (400 MHz, Acetone-d_6, *major rotamer*): δ 8.08 (1H, d, *J* = 10.4 Hz, Ar-H), 7.66 (1H, d, *J* = 9.2 Hz, Ar-H), 7.47 (1H, s, Ar-H), 7.30-7.20 (2H, m, 2x Ar-H), 6.09 (2H, m, 2x N-H), 5.88 (1H, m, N-H), 4.96 (1H, m, DABA1-β-CH), 4.72-4.61 (2H, m, DABA1-α-CH + Trp4-α-CH), 4.30 (1H, m, Lys3-α-CH), 3.94–3.66 (2H, m, Gly2-α-CH_2), 3.12 (2H, d, *J* = 8.0 Hz, Trp4-β-CH_2), 2.99 (2H, d, *J* = 8.4 Hz, Lys3-β-CH_2), 2.80 (3H, s, NCH_3), 1.63 (6H, m, Lys-γ-CH_2 + Lys-δ-CH_2 + Lys-ε-CH_2), 1.37–1.24 (36H, m, 12 x CH_3), 1.16 (3H, d, *J* = 9.2 Hz, DABA1-γ-CH_3). **LRMS** [M+H$^+$] 904.6. **HRMS (ESI m/z)** [M+Na$^+$] calcd. for $C_{44}H_{69}N_7O_{13}Na$926.4851, found 926.4854.

(2*S*,5*S*,9*S*)-5-benzyl-9-((1-(*tert*-butoxycarbonyl)-1*H*-indol-3-yl)methyl)-2-((*S*)-1-(2-((*tert*-butoxycarbonyl)amino)-*N*-methylacetamido)ethyl)-12,12-dimethyl-4,7,10-trioxo-11-oxa-3,6,8-triazatridecan-1-oic acid (S44)

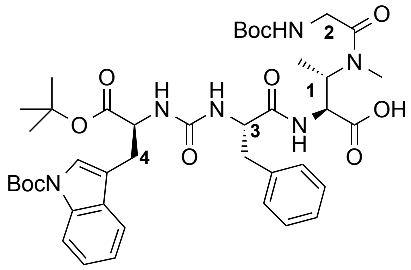

Resin-bound isopeptide **S35** (69 μmol) was Fmoc-deprotected (general procedure 2) and coupled to Fmoc-Phe-OH (107 mg, 276 μmol) using PyBOP (144 mg, 276 μmol) and NMM (61 μL, 553 μmol) in DMF (0.7 mL) according to general procedure 6. Carbamate **S12** (73 mg, 138 μmol) was subsequently coupled according to general procedure 4. Following cleavage from the resin (general procedure 7), the peptide was purified by reverse-phase HPLC (50 to 100% MeCN over 40 min,) to afford depsipeptide **S44** as a fluffy white solid. (48 mg, 55%).

IR (ATR): 3346, 2978, 2934, 1729, 1644 cm^{-1}. **^{1}H NMR** (300 MHz, (Acetone-d_6, *major rotamer*): δ 8.12 (1H, d, J = 7.8 Hz, Ar-H), 7.68 (1H, d, J = 6.9 Hz, Ar-H), 7.50 (1H, s, Ar-H), 7.34-7.14 (7H, m, 2x Ar-H + 5x Phe-Ar-H), 6.17-6.09 (2H, m, 2x N-H), 5.88 (1H, m, N-H), 4.94 (1H, m, DABA1-β-CH), 4.76-4.63 (1H, m, DABA1-α-CH), 4.62–4.60 (2H, m, Trp4-α-CH + Phe3-α-CH), 3.87–3.74 (2H, m, Gly2-α-CH_2), 3.15 (4H, m, Trp4-β-CH_2 + Phe-3-β-CH_2), 2.83 (3H, s, NCH_3), 1.66 (9H, m, 3x CH_3), 1.41–1.36 (18H, m, 6x CH_3), 1.16 (3H, d, J = 9.2 Hz, DABA1-γ-CH_3). **LRMS** [M+H^+] 823.5. **HRMS (ESI m/z)** [M+H^+] calcd. for $C_{42}H_{59}N_6O_{11}$ 823.4163, found 823.4239.

(2*S*,5*S*,9*S*)-9-((1-(*tert*-butoxycarbonyl)-1*H*-indol-3-yl)methyl)-2-((*S*)-1-(2-((*tert*-butoxycarbonyl)amino)-*N*-methylacetamido)ethyl)-12,12-dimethyl-4,7,10-trioxo-5-(4-(trifluoromethyl)benzyl)-11-oxa-3,6,8-triazatridecanoic acid (S45)

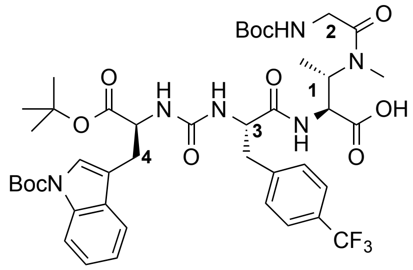

Resin-bound isopeptide **S35** (88 μmol) was Fmoc-deprotected (general procedure 2) and coupled to Fmoc-Phe(4-CF_3)-OH (160 mg, 352 μmol) using PyBOP (183 mg, 352 μmol) and NMM (73 μL, 704 μmol) in DMF (0.9 mL) according to general procedure 3. Carbamate **S12** (94 mg, 176 μmol) was subsequently coupled according to general procedure 4. Following cleavage from the resin (general procedure 7), the peptide was purified by reverse phase HPLC (50-100% MeCN over 40 min, 10 min at 50% MeCN) to afford depsipeptide **S45** as a fluffy white solid (29 mg, 36%).

IR (ATR): 3374, 2979, 2932, 1731, 1666 cm^{-1}. **^{1}H NMR** (400 MHz, $CDCl_3$, *major rotamer*): δ 8.04 (1H, *app.* d, *J* = 8.2 Hz, Ar-H), 7.76-7.60 (1H, m), 7.64 (1H, d, *J* = 6.8 Hz), 7.53 (1H, *app.* d, *J* = 7.5 Hz, Ar-H), 7.47-7.36 (4H, m, Ar-H), 7.30-7.14 (3H, m, Ar-H), 5.93 (1H, s), 5.82 (1H, s), 5.54 (1H, s), 4.98-4.82 (1H, m, DABA1-β-CH), 4.76-4.32 (3H, m, DABA1-α-CH + 4-CF_3-Phe3-α-CH + Trp4-α-CH), 3.85-3.67 (2H, m, Gly2-α-CH_2), 3.17-3.05 (3H, m, 4-CF_3-Phe3-β-CH_2 + Trp4-β-CH_2), 3.00-2.91 (1H, m, Trp4-β-CH_2), 2.73 (3H, s, NCH_3), 1.62 (9H, s, 3× CH_3), 1.42-1.19 (18H, m, 6× CH_3), 1.15 (3H, d, *J* = 6.9 Hz, DABA1-γ-CH_3). **LRMS** [M+H$^+$] 891.4. **HRMS (ESI m/z)** [M+H$^+$] calcd. for $C_{43}H_{57}F_3N_6O_{11}$ 891.4110, found 891.4103.

(2*S*,5*S*,9*S*)-5-(4-(*tert*-butoxy)benzyl)-9-((1-(*tert*-butoxycarbonyl)-1*H*-indol-3-yl)methyl)-2-((*S*)-1-(2-((*tert*-butoxycarbonyl)amino)-*N*-methylacetamido)ethyl)-12,12-dimethyl-4,7,10-trioxo-11-oxa-3,6,8-triazatridecan-1-oic acid (S46)

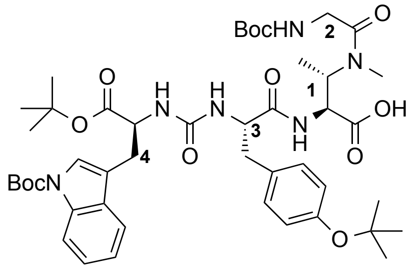

Resin-bound isopeptide **S35** (60 μmol) was Fmoc-deprotected (general procedure 2) and coupled to Fmoc-Tyr(O^{t}Bu)-OH (110 mg, 240 μmol) using PyBOP (125 mg, 240 μmol) and NMM (53 μL, 480 μmol) in DMF (0.6 mL) according to general procedure 6. Carbamate **S12** (63 mg, 120 μmol) was subsequently coupled according to general procedure 4. Following cleavage from the resin (general procedure 7), the peptide was purified by column chromatography (eluent: 95:5 v/v CH_2Cl_2: MeOH, 0.1 vol.% acetic acid) to afford depsipeptide **S46** as a yellow oil (34.8 mg, 56%).

IR (ATR): 3350, 2978, 2929, 1726, 1637 cm^{-1}. **^{1}H NMR** (400 MHz, $CDCl_3$, *major rotamer*): δ 8.06 (1H, d, *J* = 7.2 Hz, Ar-H), 7.55 (1H, d, *J* = 7.6 Hz, Ar-H), 7.39 (1H, s, Ar-H), 7.29-7.18 (2H, m, 2x Ar-H), 7.04 (2H, d, *J* = 8.4 Hz, 2x Ar-H), 6.82 (2H, d, *J* = 8.4 Hz, 2x Ar-H), 5.72–5.61 (2H, m, 2 x N-H), 4.84 (1H, m, DABA1-β-CH), 4.65 (1H, m, DABA1-α-CH), 4.57 (1H, m, Trp4-α-CH), 4.52 (2H, m, Tyr3-α-CH), 3.91–3.71 (2H, m, Gly2-α-CH_2), 3.12 (2H, m, Trp4-β-CH_2), 2.98 (2H, m, Trp4-β-CH_2), 2.79 (3H, s, NCH_3), 1.63 (9H, s, 3x CH_3), 1.40–1.25 (27H, m, 9x CH_3), 1.14 (3H, d, *J* = 6.8 Hz, DABA1-γ-CH_3). **LRMS** [*M*+H$^+$] 895.5. **HRMS (ESI m/z)** [*M*+Na$^+$] calcd. for $C_{46}H_{66}N_6O_{12}Na$ 895.4817, found 895.4810.

(2*S*,5*S*,9*S*)-9-((1-(*tert*-butoxycarbonyl)-1*H*-indol-3-yl)methyl)-2-((*S*)-1-(2-((*tert*-butoxycarbonyl)amino)-*N*-methylacetamido)ethyl)-12,12-dimethyl-4,7,10-trioxo-5-(pyridin-3-ylmethyl)-11-oxa-3,6,8-triazatridecanoic acid (S47)

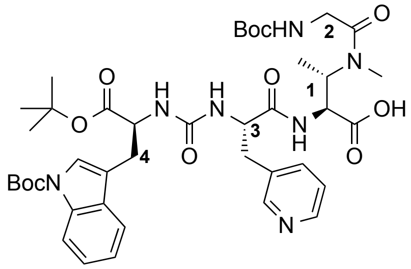

Resin-bound isopeptide **S35** (60 μmol) was Fmoc-deprotected (general procedure 2) and coupled to Fmoc-3-Pal-OH (92 mg, 240 μmol) using PyBOP (125 mg, 240 μmol) and NMM (50 μL, 480 μmol) in DMF (0.6 mL) according to general procedure 3. Carbamate **S12** (64 mg, 120 μmol) was subsequently coupled according to general procedure 4. Following cleavage from the resin (general procedure 7), the peptide was purified by reverse phase HPLC (50-100% MeCN over 60 min, 10 min at 50% MeCN) to afford depsipeptide **S47** as a fluffy white solid (14 mg, 29%).

IR (ATR): 3366, 2979, 2936, 1726, 1660 cm^{-1}. **^{1}H NMR** (500 MHz, Acetone-d_6, *major rotamer*): δ 8.70-8.61 (2H, m, NH + Ar-H), 8.21 (1H, *app.* d, *J* = 7.9 Hz, Ar-H), 8.11 (1H, *app.* d, *J* = 8.2 Hz, Ar-H), 7.98 (1H, d, *J* = 8.8 Hz, Ar-H), 7.83-7.81 (1H, m, Ar-H), 7.64 (1H, d, *J* = 7.0 Hz, Ar-H), 7.50 (1H, s, Ar-H), 7.33-7.28 (1H, m, Ar-H), 7.25 (1H, *app.* td, *J* = 7.5, 1.2 Hz, Ar-H), 6.37-6.21 (2H, m, 2× NH), 5.86-5.73 (1H, m), 4.97-4.89 (1H, m, DABA1-β-CH), 4.81-4.73 (1H, m, *J* = 7.0 Hz, 3-Pal3-α-CH), 4.71-4.62 (1H, m, DABA1-α-CH), 4.61-4.52 (1H, m, Trp4-α-CH), 3.94-3.81 (1H, m, Gly2-α-CH_2), 3.78-3.69 (1H, m, Gly2-α-CH_2), 3.31 (1H, *app.* dt, *J* = 15.6, 7.6 Hz, 3-Pal3-β-CH_2), 3.20-3.06 (3H, m, 3-Pal3-β-CH_2 + Trp4-β-CH_2), 2.83 (3H, s, NCH_3), 1.65 (9H, s, 3× CH_3), 1.43-1.31 (18H, s, 6× CH_3), 1.18 (3H, d, *J* = 7.1 Hz, DABA1-γ-CH_3). **LRMS** [M+H^+] 829.5. **HRMS (ESI m/z)** [M+H^+] calcd. for $C_{41}H_{57}N_7O_{11}$ 824.4188, found 824.4182.

(2*S*,5*S*,9*S*)-5,9-bis((1-(*tert*-butoxycarbonyl)-1*H*-indol-3-yl)methyl)-2-((*S*)-1-(2-((*tert*-butoxycarbonyl)amino)-*N*-methylacetamido)ethyl)-12,12-dimethyl-4,7,10-trioxo-11-oxa-3,6,8-triazatridecan-1-oic acid (S48)

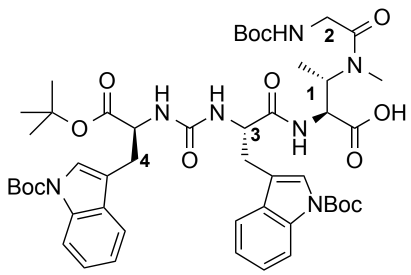

Resin-bound isopeptide **S35** (60 μmol) was Fmoc-deprotected (general procedure 2) and coupled to Fmoc-Trp(Boc)-OH (126.4 mg, 240 μmol) using PyBOP (125 mg, 240 μmol) and NMM (53 μL, 480 μmol) in DMF (0.6 mL) according to general procedure 7. Carbamate **S12** (90.4 mg, 172 μmol) was subsequently coupled according to general procedure 4. Following cleavage from the resin (general procedure 7), the peptide was purified by column chromatography (eluent: 95:5 v/v CH_2Cl_2: MeOH, 0.1 vol.% acetic acid on deactivated silica) to afford depsipeptide **S48** as a yellow oil (30.2 mg, 52%).

IR (ATR): 3352, 2980, 2928, 1731, 1646, 1553 cm^{-1}. **^{1}H NMR** (500 MHz, $CDCl_3$, *major rotamer*): δ 8.04 (1H, m, 2x Ar-H), 7.53 (1H, d, *J* = 7.6 Hz, 2x Ar-H), 7.40 (2H, m, Ar-H), 7.28-7.11 (4H, m, 4x Ar-H), 6.29 (1H, m, N-H), 6.06 (1H, m, N-H), 5.57 (1H, m, N-H), 4.84 (1H, m, DABA1-β-CH), 4.68-4.52 (2H, m, Trp3-α-CH + Trp4-α-CH), 4.47 (1H, m, DABA1-α-CH), 3.75–3.48 (2H, m, Gly2-α-CH_2), 3.16-2.91 (4H, m, Trp3-β-CH_2 + Trp4-β-CH_2), 2.88 (3H, s, NCH_3), 1.61 (18H, s, 6x CH_3), 1.42–1.22 (18H, m, 6x CH_3), 1.11 (3H, d, *J* = 6.6 Hz, DABA1-γ-CH_3). **LRMS** [M+H^+] 962.6. **HRMS (ESI m/z)** [M+H^+] calcd. for $C_{49}H_{68}N_7O_{11}$ 962.4876, found 962.4869.

(2*S*,5*S*,9*S*)-9-((1-(*tert*-butoxycarbonyl)-1*H*-indol-3-yl)methyl)-2-((*S*)-1-(2-((*tert*-butoxycarbonyl)amino)-*N*-methylacetamido)ethyl)-12,12-dimethyl-4,7,10-trioxo-5-phenyl-11-oxa-3,6,8-triazatridecan-1-oic acid (S49)

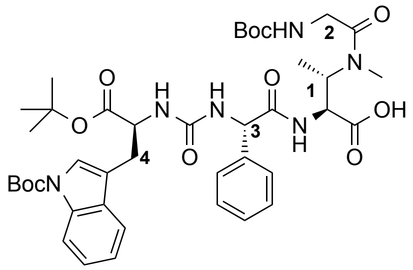

Resin-bound isopeptide **S35** (70 μmol) was Fmoc-deprotected (general procedure 2) and coupled to Fmoc-Phg-OH (31.5 mg, 84 μmol) using HOAt (11.2 mg, 84 μmol,) and DIC (13.3 μL, 84 μmol) in DMF (0.7 mL) according to general procedure 6. Carbamate **S12** (73.6 mg, 140 μmol) was subsequently coupled according to general procedure 4. Following cleavage from the resin (general procedure 7), the peptide was provided as a 9:1 mixture of diastereomers, which were readily separable by reverse-phase HPLC (50 to 100% MeCN over 40 min) to afford depsipeptide **S49** as a single diastereomer as a fluffy white solid (14.5 mg, 25%).

IR (ATR): 3343, 2978, 2933, 1727, 1700, 1638 cm^{-1}. **^{1}H NMR** (400 MHz, Acetone-d_6, *major rotamer*): δ 8.13 (1H, d, *J* = 7.6 Hz, Ar-H), 7.72 (1H, d, *J* = 6.8 Hz, Ar-H), 7.52 (1H, s, Ar-H), 7.46 (2H, d, *J* 7.2 Hz, 2x Ar-H), 7.47-7.24 (5H, m, 5x Ar-H), 6.66 (1H, m, N-H), 6.27 (1H, m, N-H), 5.80 (1H, m, N-H), 5.57 (1H, m, Phg3-α-CH), 4.99 (1H, m, DABA1-β-CH), 4.79 (1H, m, DABA1-α-CH), 4.65 (1H, m, Trp4-α-CH), 3.95-3.71 (2H, m, Gly2-α-CH_2), 3.17 (2H, m, Trp4-β-CH_2), 2.88 (3H, s, NCH_3), 1.67 (9H, s, 3x CH_3), 1.40 (9H, m, 3x CH_3), 1.35 (9H, m, 3x CH_3), 1.25 (3H, d, *J* = 6.8 Hz, DABA1-γ-CH_3). **LRMS** [*M*+H^+] 809.5. **HRMS (ESI m/z)** [*M*+Na^+] calcd. for $C_{41}H_{56}N_6O_{11}Na$ 809.4085, found 809.4080.

(2*S*,5*S*,9*S*)-9-((1-(*tert*-butoxycarbonyl)-1*H*-indol-3-yl)methyl)-2-((*S*)-1-(2-((*tert*-butoxycarbonyl)amino)-*N*-methylacetamido)ethyl)-12,12-dimethyl-4,7,10-trioxo-5-phenethyl-11-oxa-3,6,8-triazatridecan-1-oic acid (S50)

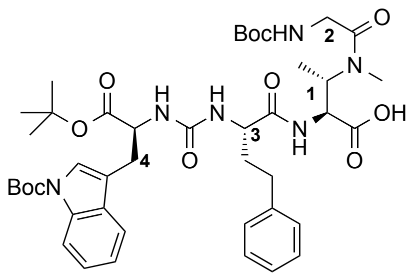

Resin-bound isopeptide **S35** (70 µmol) was Fmoc-deprotected (general procedure 2) and coupled to Fmoc-HPhe-OH (33.7 mg, 84 µmol) using HOAt (11.2 mg, 84 µmol) and DIC (13.3 µL, 84 µmol) in DMF (0.7 mL) according to general procedure 6. Carbamate **S12** (73.6 mg, 140 µmol) was subsequently coupled according to general procedure 4. Following cleavage from the resin (general procedure 7), the peptide was provided as a 9:1 mixture of diastereomers which were readily separable by reverse-phase HPLC (50 to 100% MeCN over 40 min) to afford depsipeptide **S50** as a single diastereomer as a fluffy white solid (18.8 mg, 27%).

IR (ATR): 3342, 2977, 2931, 1730 cm^{-1}. ^{1}**H NMR** (500 MHz, $CDCl_3$, *major rotamer*): δ 8.11 (1H, d, *J* = 8.0 Hz, Ar-H), 7.69 (1H, d, *J* = 7.5 Hz, Ar-H), 7.53 (1H, s, Ar-H), 7.32-7.12 (7H, m, 7x Ar-H), 6.39 (1H, m, N-H), 6.25 (1H, m, N-H), 5.95 (1H, m, N-H), 4.96 (1H, m, DABA1-β-CH), 4.71 (1H, m, DABA1-α-CH), 4.64 (1H, m, Trp4-α-CH), 4.36 (1H, m, HPhe3-α-CH), 3.96–3.69 (2H, m, Gly2-α-CH_2), 3.15 (2H, m, Trp4-β-CH_2), 2.80 (3H, s, NCH_3), 2.64 (2H, m, HPhe3-β-CH_2), 2.02 (2H, m, HPhe3-γ-CH_2), 1.63 (9H, s, 3x CH_3), 1.36–1.34 (18H, m, 6x CH_3), 1.16 (3H, d, *J* = 7.1 Hz, DABA1-γ-CH_3). **LRMS** [*M*+H$^+$] 837.5. **HRMS (ESI m/z)** [*M*+Na$^+$] calcd. for $C_{43}H_{60}N_6O_{11}Na$ 859.4218, found 859.4205.

(2*S*,5*S*,9*S*)-9-((1-(*tert*-butoxycarbonyl)-1*H*-indol-3-yl)methyl)-2-((*S*)-1-(2-((*tert*-butoxycarbonyl)amino)-*N*-methylacetamido)ethyl)-12,12-dimethyl-5-(naphthalen-2-ylmethyl)-4,7,10-trioxo-11-oxa-3,6,8-triazatridecanoic acid (S51)

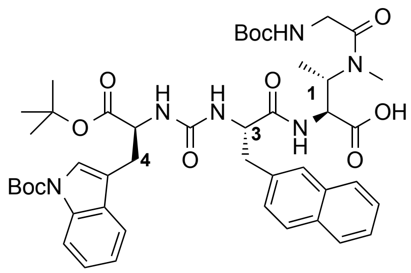

Resin-bound isopeptide **S35** (60 μmol) was Fmoc-deprotected (general procedure 2) and coupled to Fmoc-2-Nal-OH (104 mg, 240 μmol) using PyBOP (124 mg, 240 μmol) and NMM (50 μL, 480 μmol) in DMF (0.6 mL) according to general procedure 3. Carbamate **S12** (64 mg, 120 μmol) was subsequently coupled according to general procedure 4 in the presence of *N,N*-diisopropylethylamine (21 μL, 120 μmol) in DMF (1 mL). Following cleavage from the resin (general procedure 7), the peptide was purified by reverse phase HPLC (50-100% MeCN over 40 min, 10 min at 50% MeCN) to afford depsipeptide **S51** as a fluffy white solid (22 mg, 42%).

IR (ATR): 3390, 2978, 2936, 1730, 1644 cm^{-1}. **^{1}H NMR** (500 MHz, Acetone-d_6, *major rotamer*): δ 8.11 (1H, d, J = 8.2 Hz, Ar-H), 7.86-7.61 (6H, m, 5× Ar-H + NH), 7.50 (1H, s, Ar-H), 7.45-7.37 (3H, m, Ar-H), 7.34-7.21 (2H, m, Ar-H), 6.22-6.12 (2H, m, NH), 5.82 (1H, s, NH), 5.01-4.92 (1H, m, DABA1-β-CH), 4.82-4.69 (2H, m, DABA1-α-CH + 2-Nal3-α-CH), 4.66-4.58 (1H, m, Trp4-α-CH), 3.93-3.83 (1H, m, Gly2-α-CH_2), 3.72 (1H, dd, J = 17.0, 4.2 Hz, Gly2-α-CH_2), 3.39-3.25 (1H, m, 2-Nal3-β-CH_2), 3.19-3.05 (3H, m, 2-Nal3-β-CH_2 + Trp4-β-CH_2), 2.84 (3H, s, NCH_3), 1.65 (9H, s, 3× CH_3), 1.46-1.30 (18H, m, 6× CH_3), 1.17 (3H, d, J = 7.2 Hz, DABA1-γ-CH_3). **LRMS** [M+H^+] 873.4. **HRMS (ESI m/z)** [M+Na^+] calcd. for $C_{46}H_{60}N_6O_{11}Na$ 895.4212, found 895.4212.

(2*S*,5*S*,9*S*)-9-((1-(*tert*-butoxycarbonyl)-1*H*-indol-3-yl)methyl)-2-((*S*)-1-((*S*)-2-((*tert*-butoxycarbonyl)amino)-3-(3-((*tert*-butyldimethylsilyl)oxy)phenyl)-*N*-methylpropanamido)ethyl)-5-(cyclohexylmethyl)-12,12-dimethyl-4,7,10-trioxo-11-oxa-3,6,8-triazatridecanoic acid (S52)

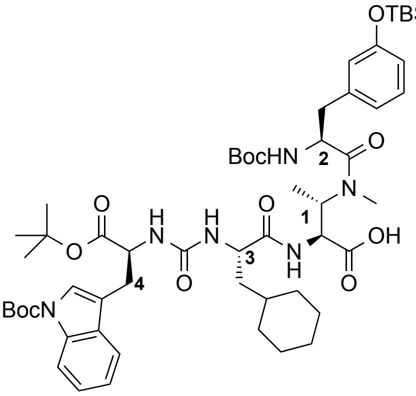

Resin-bound amino acid **S25** (87 μmol) was coupled to Fmoc-Cha-OH (68 mg, 174 μmol) using HATU (65 mg, 174 μmol) and *N*,*N*-diisopropylethylamine (48 μL, 260 μmol) in DMF (0.9 mL) according to general procedure 6. Carbamate **S12** (93 mg, 174 μmol) was subsequently coupled according to general procedure 4. Allyl carbamate was subsequently removed using *tetrakis*(triphenylphosphine) palladium(0) (20 mg, 17 μmol) and phenylsilane (20 μL, 170 μmol) in CH_2Cl_2 (1 mL) according to general procedure 5. Boc-*m*-Tyr(OTBS)-OH (51 mg, 130 μmol) was subsequently coupled using HATU (49 mg, 130 μmol), HOAt (25 mg, 130 μmol) and *N*,*N*-Diisopropylethylamine (23 μL, 130 μmol) in DMF (0.87 mL). Following cleavage from the resin (general procedure 7), the peptide was purified by reverse phase HPLC (50-100% MeCN over 40 min, 10 min at 50% MeCN) to afford depsipeptide **S52** as a fluffy white solid (18 mg, 20%).

IR (ATR): 3314, 2928, 2856, 1730, 1630 cm^{-1}. ^{1}**H NMR** (500 MHz, $CDCl_3$, *major rotamer*): δ 8.08-7.92 (1H, m, Ar-H), 7.79 (1H, s), 7.61-7.52 (1H, m, Ar-H), 7.45-7.36 (1H, m, Ar-H), 7.31-7.15 (2H, m, Ar-H), 7.01-6.87 (1H, m, Ar-H), 6.72-6.46 (3H, m, Ar-H), 6.11-5.73 (1H, m, NH), 5.69-5.53 (1H, m, NH), 4.94-4.84 (1H, m, DABA1-β-CH), 4.76-4.60 (3H, m, DABA1-α-CH + *m*-Tyr2-α-CH + Trp4-α-CH), 4.46-4.19 (1H, m, Cha3-α-CH), 3.30-3.07 (2H, m, Trp4-β-CH_2), 3.02 (3H, s, NCH_3), 2.98-2.87 (1H, m, *m*-Tyr2-β-CH_2), 2.61-2.46 (1H, m, *m*-Tyr2-β-CH_2), 1.78-0.74 (58H, m). **LRMS** [*M*+H$^+$] 1049.4. **HRMS (ESI m/z)** [*M*+H$^+$] calcd. for $C_{55}H_{84}N_6O_{12}Si$ 1049.5989, found 1049.5981.

Synthesis of dihydrosansanmycin analogues 7-17 and 21-37

General procedure 8: synthesis of dihydrosansanmycin analogues 7-17, 21-37 *via* fragment condensation strategy

Condition A:

To a solution of amine **18** (15-25 µmol, 1.2 eq.) in CH_2Cl_2 and DMF (1:1 v/v CH_2Cl_2: DMF, 0.1 M) was added depsipeptide **S24**, **S26**, **S27**, **S29**, **S30-S34** and **S53** (13-21 µmol, 1 eq.) and HOAt (5-5.5 eq.), immediately followed by DIC (1 eq.). The reaction was allowed to stir at rt for 1.5-2 h, at which point, the reaction mixture was diluted with H_2O (5 mL) and saturated aqueous $NaHCO_3$ solution (5 mL) and partitioned with EtOAc (30 mL). The organic phase was washed with a further portion of saturated aqueous $NaHCO_3$ solution (5 mL), dried over anhydrous $MgSO_4$ and concentrated *in vacuo* to afford a crude residue which was then resuspended in a mixture of TFA and *i*Pr_3SiH in CH_2Cl_2 (1:1 v/v TFA: CH_2Cl_2, 2.5 vol.% *i*Pr_3SiH, 0.1 mL/µmol) and the reaction stirred at rt (1.5-16 h). The solvent was removed *in vacuo* to afford a crude residue which was purified *via* reverse phase HPLC.

Condition B:

To a solution of EDC.HCl (12-30 µmol, 1.3–3.3 eq.) in 1:1 v/v CH_2Cl_2: DMF (180-240 µL) was added NMM (19-30 µmol, 1.3 eq.) and the solution was shaken for 15 min at rt. This solution was then added dropwise to a solution of depsipeptide **S28**, **S36-S52** (15-25 µmol, 1 eq.), amine **18** (28 -50 µmol, 1.2–3.3 eq.) and HOAt (70 – 120 µmol, 5 eq.) and the reaction stirred at rt for 3-4 h. At this point, the reaction was diluted with EtOAc (15-25 mL), and washed with HCl (0.5 M, 4-7mL), saturated aqueous $NaHCO_3$ solution (5 x 4-7mL), H_2O (4-7 mL) and brine (4-7 mL) and dried over $MgSO_4$. The solvent was then removed *in vacuo* to afford a crude residue which was redissolved in a mixture of CH_2Cl_2, TFA and *i*Pr_3SiH (1:1 CH_2Cl_2: TFA, 2.5 vol.% *i*Pr_3SiH, 0.1 mL/µmol) and the reaction stirred at rt (5-16 h). The solvent was removed *in vacuo* to afford a crude residue which was purified *via* reverse phase HPLC.

(((*S*)-1-(((2*S*,3*S*)-3-((*S*)-2-amino-3-(3-hydroxyphenyl)-*N*-methylpropanamido)-1-((((2*R*,4*R*,5*R*)-5-(2,4-dioxo-3,4-dihydropyrimidin-1(2*H*)-yl)-4-hydroxytetrahydrofuran-2-yl)methyl)amino)-1-oxobutan-2-yl)amino)-4-(methylthio)-1-oxobutan-2-yl)carbamoyl)-*L*-tryptophan (4)

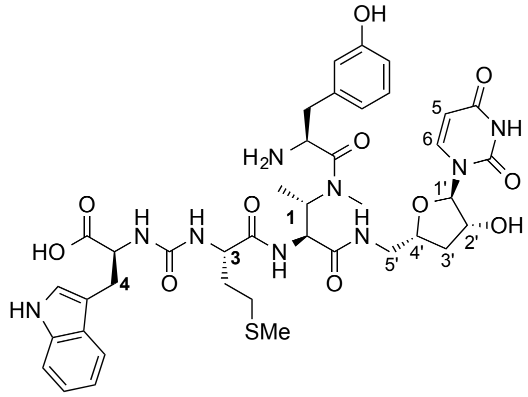

Depsipeptide **S22** (16 mg, 16 μmol) was reacted with amine **18** (9 mg, 40 μmol) in CH_2Cl_2: DMF (1:1 v/v, 160 μL) in the presence of HOAt (11 mg, 80 μmol) and EDC.HCl (3.9 mg, 20 μmol) and NMM (2.1 μL, 20 μmol) for 4 h at rt according to general procedure 8 condition B to obtain the fully protected dihydrosansanmycin analogue. This compound was suspended in a mixture of TFA in H_2O (9:1 v/v TFA: H_2O, 6.4 mL) and the reaction was cooled to 0 °C. Dimethyl sulfide (23 μL, 320 μmol) and ammonium iodide (47 mg, 320 μmol) were then added and the reaction was stirred at 0 °C for 30 min. At this point, the solvent was removed under a stream of nitrogen to give a crude a residue that was purified by reverse phase HPLC (0 to 50% MeCN over 40 min, 10 min at 100% H_2O) to afford dihydrosansanmycin **4** (as a TFA salt) as an amorphous white solid (5.8 mg, 37% over 2 steps).

IR (ATR): 3303, 3078, 2925, 1670, 1553 cm^{-1}. **^{1}H NMR** (500 MHz, CD_3OD, rotamers in 1.8:1 ratio, *major rotamer*): δ 7.59 (1H, d, *J* = 8.1 Hz, H-6), 7.54 (1H, d, *J* = 8.0 Hz, Ar-H), 7.34-7.30 (1H, m, Ar-H), 7.20-7.14 (1H, m, Ar-H), 7.10 (1H, s, Ar-H), 7.08-7.04 (1H, m, Ar-H), 6.98 (1H, *app.* td, *J* = 7.5, 3.5 Hz, Ar-H), 6.80-6.71 (2H, m, Ar-H), 6.68 (1H, *app.* t, *J* = 2.1 Hz, Ar-H), 5.75 (1H, d, *J* = 2.5 Hz, H-1'), 5.71 (1H, d, *J* = 8.1 Hz, H-5), 4.63-4.55 (1H, m, Trp4-α-CH), 4.54-4.48 (1H, m, DABA1-α-CH), 4.43-4.33 (3H, m, H-2' + H-4' + *m*-Tyr2-α-CH), 4.27 (1H, dd, *J* = 8.4, 5.5 Hz, Met3-α-CH), 4.13-4.06 (1H, m, DABA1-β-CH), 3.47 (1H, dd, *J* = 14.0, 3.9 Hz, H-5'), 3.29-3.13 (3H, m, H-5' + Trp4-β-CH_2), 3.05-2.96 (2H, m, *m*-

Tyr2-β-CH_2), 2.75 (3H, s, NCH_3), 2.64 (s, 1H), 2.52-2.40 (2H, m, Met3-γ-CH_2), 2.21 (1H, *app.* dt, *J* = 13.5, 7.3 Hz, H-3'), 2.02 (3H, s, Met3-SMe), 1.98-1.88 (1H, m, Met3-β-CH_2), 1.87-1.77 (1H, m, Met3-β-CH_2), 1.75-1.68 (1H, m, H-3'), 0.79 (3H, d, *J* = 6.4 Hz, DABA1-γ-CH_3). **^{13}C NMR** (125 MHz, CD_3OD, rotamers in 1.8:1 ratio, *major rotamer*): δ 174.5 (C=O), 174.2 (C=O), 173.4 (C=O), 171.0 (C=O), 164.3 (C=O), 158.6, 151.5 (C=O), 142.1, 140.0, 139.7, 136.9, 128.6, 124.4, 122.2, 121.2, 119.4, 119.0, 117.0, 115.8, 111.4, 112.0, 102.2, 94.4, 81.0, 76.1, 56.9, 54.6, 54.4, 54.1, 53.6, 44.5, 38.0, 35.7, 32.6, 30.6, 28.7, 28.0, 14.8, 14.0. (^{13}C signal which corresponds to the carbonyl of the urea linker is obscured). **LRMS** [M+H$^+$] 866.4. **HRMS (ESI m/z)** [*M*+H$^+$] calcd. for $C_{40}H_{52}N_9O_{11}S$ 866.3502, found 866.3502.

(((*S*)-1-(((2*S*,3*S*)-3-((*S*)-2-amino-3-(3-hydroxyphenyl)-*N*-methylpropanamido)1-((((2*R*,4*R*,5*R*)-5-(2,4-dioxo-3,4-dihydropyrimidin-1(2*H*)-yl)-4-hydroxytetrahydrofuran-2-yl)methyl)amino)-1-oxobutan-2-yl)amino)-4-methyl-1-oxopentan-2-yl)carbamoyl)-*L*-tryptophan (5)

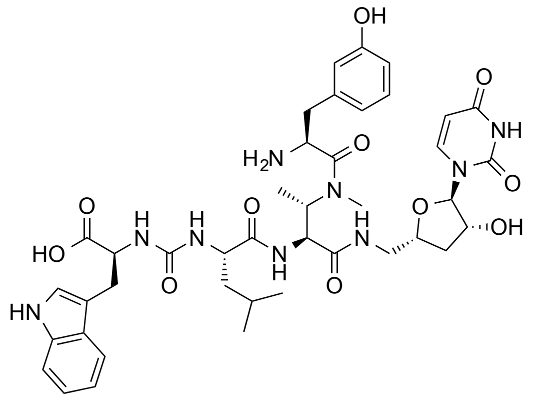

Depsipeptide **S23** (13 mg, 13 μmol) was reacted with amine **18** (4.5 mg, 19 μmol) in CH_2Cl_2: DMF (1:1 v/v, 130 μL) in the presence of HOAt (8.8 mg, 65 μmol) and EDC.HCl (3.2 mg, 17 μmol) and NMM (1.8 μL, 17 μmol) for 4 h at rt according to general procedure 8 condition B to obtain the fully protected dihydrosansanmycin analogue. This compound was treated with a mixture of TFA and *i*Pr_3SiH in CH_2Cl_2 (1:1 v/v TFA: CH_2Cl_2, 2.5 vol.% *i*Pr_3SiH, 1.3 mL) for 16 h according to general procedure 8 to afford dihydrosansanmycin **5** (as a formate salt) after reverse phase HPLC purification (0 to 50% MeCN over 40 min, 10 min at 100% H_2O) as an amorphous white solid (3.5 mg, 30% over 2 steps).

IR (ATR): 3257, 3063, 2956, 1650, 1587 cm^{-1}. **^{1}H NMR** (500 MHz, DMSO-*d*$_6$, rotamers in 2:1 ratio, *major rotamer*): δ 10.75 (1H, d, *J* = 9.8 Hz), 8.50 (1H, s), 8.32

(1H, s), 8.13 (1H, s), 7.69-7.47 (2H, m, H-6 + Ar-H), 7.29 (1H, *app.* d, J = 8.1 Hz, Ar-H), 7.10 (1H, s, Ar-H), 7.07-6.98 (3H, m, Ar-H), 6.71-6.62 (1H, m, Ar-H), 6.59-6.42 (2H, m, Ar-H), 5.67 (1H, d, J = 3.5 Hz, H-1'), 5.65 (1H, d, J = 3.3 Hz, H-1'), 5.60 (1H, d, J = 8.0 Hz, H-5), 4.57-4.02 (7H, m, H-2' + H-4' + DABA1-α-CH + DABA1-β-CH + *m*-Tyr2-α-CH + Leu3-α-CH + Trp4-α-CH), 3.35-3.26 (1H, m, H-5'), 3.22-2.96 (3H, m, H-5' + Trp4-β-CH_2), 2.69-2.57 (5H, m, *m*-Tyr2-β-CH_2 + NCH_3), 2.18-2.06 (1H, m, H-3'), 1.67-1.50 (2H, m, H-3' + Leu3-γ-CH), 1.42-1.29 (2H, m, Leu3-β-CH_2), 0.92-0.59 (9H, m, DABA1-γ-CH_3 + 2× Leu3-δ-CH_3). **^{13}C NMR** (125 MHz, DMSO-d_6, rotamers in 2:1 ratio): δ 174.7 (C=O), 173.6 (C=O), 173.3 (C=O), 170.0 (C=O), 163.7 (C=O), 160.4 (C=O), 157.6, 150.7 (C=O), 140.8, 138.9, 135.6, 128.7, 127.7, 123.1, 120.4, 119.6, 118.8, 117.6, 116.3, 113.0, 110.8, 110.7, 101.2, 91.8, 78.4, 74.0, 54.8 (×2), 54.6, 51.7, 50.6, 43.3, 40.8, 40.5, 35.1, 28.2, 27.0, 23.9, 22.3, 14.1. **LRMS** [M+H$^+$] 848.4. **HRMS (ESI m/z)** [*M*+H$^+$] calcd. for $C_{41}H_{54}N_9O_{11}$ 848.3937, found 848.3937.

(((2*S*)-1-(((2*S*,3*S*)-3-((*S*)-2-amino-3-(3-hydroxyphenyl)-*N*-methylpropanamido)-1-((((2*R*,4*R*,5*R*)-5-(2,4-dioxo-3,4-dihydropyrimidin-1(2*H*)-yl)-4-hydroxytetrahydrofuran-2-yl)methyl)amino)-1-oxobutan-2-yl)amino)-4-(methylsulfinyl)-1-oxobutan-2-yl)carbamoyl)-*L*-tryptophan (6)

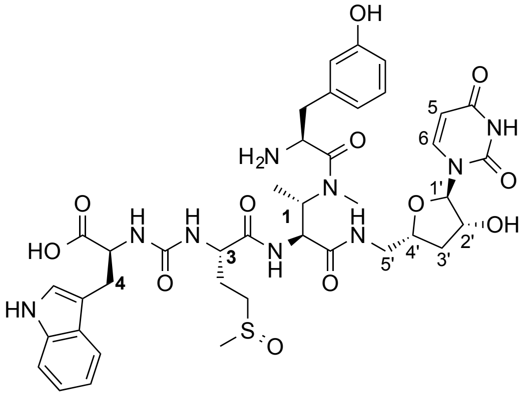

Depsipeptide **S22** (16 mg, 16 μmol) was reacted with amine **18** (9 mg, 40 μmol) in CH_2Cl_2: DMF (1:1 v/v, 160 μL) in the presence of HOAt (11 mg, 80 μmol) and EDC.HCl (3.9 mg, 20 μmol) and NMM (2.1 μL, 20 μmol) for 4 h at rt according to general procedure 8 condition B to obtain the fully protected dihydrosansanmycin analogue. This compound was treated with a mixture of TFA and *i*Pr_3SiH in H_2O (90:5:5 v/v/v TFA: *i*Pr_3SiH: H_2O, 1.6 mL) for 5 h at rt to afford dihydrosansanmycin

6 (as a TFA salt) after reverse phase HPLC purification (0 to 50% MeCN over 40 min, 10 min at 100% H_2O) as an amorphous white solid (4.2 mg, 26% over 2 steps).

IR (ATR): 3302, 2927, 1674, 1590, 1554 cm^{-1}. **^{1}H NMR** (500 MHz, DMSO-d_6, rotamers in 2:1 ratio, *major rotamer*): δ 10.78 (1H, s), 8.54 (1H, s), 8.36 (1H, s), 8.18 (1H, s), 7.63-7.47 (2H, m, H-6 + Ar-H), 7.29 (1H, *app.* d, J = 8.1 Hz, Ar-H), 7.14-6.89 (4H, m, Ar-H), 6.71-6.49 (3H, m, Ar-H), 5.68-5.62 (1H, m, H-1' major), 5.59 (1H, d, J = 8.0 Hz, H-5), 4.75-4.66 (1H, m, DABA1-β-CH), 4.57-4.47 (1H, m, DABA1-α-CH), 4.46-4.04 (4H, m, H-2' + H-4' + Met(O)3-α-CH + Trp4-α-CH), 3.95-3.86 (1H, m, *m*-Tyr2-α-CH), 3.38-2.93 (4H, m, H-5' + Trp4-β-CH_2), 2.81-2.53 (7H, m, NCH_3 + *m*-Tyr2-β-CH_2 + Met(O)3-γ-CH_2), 2.51-2.36 (3H, s, Met(O)3-S(O)CH_3), 2.18-2.04 (1H, m, H-3'), 2.01-1.58 (3H, m, H-3' + Met(O)3-β-CH_2), 0.78-0.64 (3H, m, DABA1-γ-CH_3). **^{13}C NMR** (125 MHz, DMSO-d_6, rotamers in 2:1 ratio, *major rotamer*): δ 177.1 (C=O), 175.4 (C=O), 174.4 (C=O), 171.7 (C=O), 165.3 (C=O), 159.4, 152.5 (C=O), 142.9, 140.4, 138.0, 131.2, 129.8, 125.2, 122.5, 121.5, 120.6, 120.2, 118.4, 115.6, 112.8, 112.0, 103.3, 93.8, 80.5, 75.9, 57.4, 56.4, 53.7, 53.3, 52.8, 51.1, 41.8, 41.2, 40.6, 37.2, 29.5, 28.9, 27.3, 15.9 (^{13}C signal which corresponds to the carbonyl of the urea linker is obscured). **LRMS** [M+H^+] 882.4. **HRMS (ESI m/z)** [M+H^+] calcd. for $C_{40}H_{52}N_9O_{12}S$ 882.3451, found 882.3450.

(2*S*,6*S*,9*S*,10*S*,13*S*)-2-((1*H*-indol-3-yl)methyl)-13-amino-9-((((2*R*,4*R*,5*R*)-5-(2,4-dioxo-3,4-dihydropyrimidin-1(2*H*)-yl)-4-hydroxytetrahydrofuran-2-yl)methyl)carbamoyl)-6-isobutyl-10,11-dimethyl-4,7,12-trioxo-14-phenyl-3,5,8,11-tetraazatetradecan-1-oic acid (7)

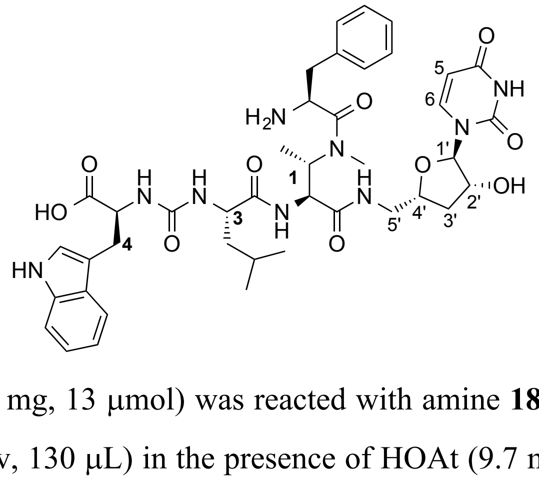

Depsipeptide **S24** (11 mg, 13 μmol) was reacted with amine **18** (3.5 mg, 16 μmol) in CH_2Cl_2: DMF (1:1 v/v, 130 μL) in the presence of HOAt (9.7 mg, 72 μmol) and DIC

(1.9 μL, 12 μmol) for 3 h at rt according to general procedure 8 condition A to obtain the fully protected dihydrosansanmycin analogue. This compound was treated with a mixture of TFA and *i*Pr_3SiH in CH_2Cl_2 (1:1 v/v TFA: CH_2Cl_2, 2.5 vol.% *i*Pr_3SiH, 1.2 mL) according to general procedure 8 to afford dihydrosansanmycin **7** (as a TFA salt) after reverse phase HPLC purification (0 to 50% MeCN over 40 min, 10 min at 100% H_2O) as an amorphous white solid (5.6 mg, 50% over 2 steps).

IR (ATR): 3315, 2975, 2871, 1436 cm^{-1}. ^{1}**H NMR** (600 MHz, MeOD, rotamers in 2.4:1 ratio, *major rotamer*): δ 7.63 (1H, d, *J* = 8.4 Hz, H-6), 7.55 (1H, d, *J* = 9.2 Hz, Ar-H), 7.38-7.32 (2H, m, 2x Ar-H), 7.31-7.26 (4H, m, 4x Ar-H), 7.09-7.05 (1H, m, Ar-H), 6.99-6.97 (1H, m, Ar-H), 5.74 (1H, d, *J* = 2.4 Hz, H-5), 5.70 (1H, d, *J* = 7.8 Hz, H-1'), 4.57-4.50 (2H, m, Phe2-α-CH + Trp4-αCH), 4.46-4.44 (1H, m, DABA1-α-CH), 4.43-4.40 (2H, m, H-2' + H-4'), 4.11 (1H, dd, *J* = 6.0, 9.0 Hz, Leu3-α-CH), 4.08–4.07 (1H, m, DABA1-β-CH), 3.51 (1H, dd, *J* = 3.6, 14.4 Hz, H-5'), 3.28-3.23 (2H, m, Phe2-β-CH_2 + Trp4-β-CH_2), 3.19-3.13 (2H, m, Phe2-β-CH_2 + Trp4-β-CH_2), 3.06 (1H, d, *J* = 7.2 Hz, H-5'), 2.76 (3H, s, NCH_3), 2.25-2.20 (1H, m, H-3'), 1.74-1.71 (1H, m, H-3'), 1.51-1.44 (1H, m, Leu3-β-CH_2), 0.91 (1H, m, 2x Leu3-γ-CH), 0.73 (3H, d, *J* = 6.6 Hz, DABA1-γ-CH_3). ^{13}**C NMR** (151 MHz, MeOD, *major rotamer)*: δ 174.8 (C=O), 174.2 (C=O), 170.2 (C=O), 168.9 (C=O), 164.8 (C=O), 158.3 (C=O), 150.6 (C=O), 141.3, 136.3**,** 134.1, 133.8**,** 129.0, 127.4**,** 127.2, 123.2, 120.7, 118.3, 118.1, 111.6, 110.7, 109.0**,** 101.1, 93.6, 79.6, 74.8, 55.7, 54.9, 53.5, 53.3, 52.0, 51.5, 43.3, 40.5, 37.2, 34.4, 27.6, 27.3, 26.9, 24.3, 21.8, 20.7, 13.0. **LRMS** [*M*+H^+] 832.5; **HRMS (ESI m/z) [***M*+Na^+] calcd. for $C_{41}H_{53}N_9O_{10}$ 832.3993, found 832.3992.

(((*S*)-1-(((2*S*,3*S*)-3-((*S*)-2-amino-*N*-methyl-3-(pyridin-3-yl)propanamido)-1-((((2*R*,4*R*,5*R*)-5-(2,4-dioxo-3,4-dihydropyrimidin-1(2*H*)-yl)-4-hydroxytetrahydrofuran-2-yl)methyl)amino)-1-oxobutan-2-yl)amino)-4-methyl-1-oxopentan-2-yl)carbamoyl)-*L*-tryptophan (8)

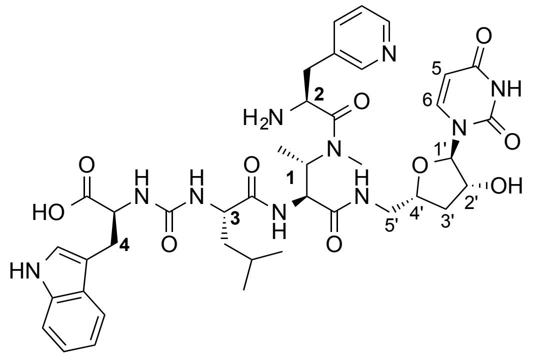

Depsipeptide **S26** (24 mg, 27 μmol) was reacted with amine **18** (7.2 mg, 32 μmol) in CH_2Cl_2: DMF (1:1 v/v, 260 μL) in the presence of HOAt (18 mg, 134 μmol) and DIC (4.3 μL, 27 μmol) for 1.5 h at rt according to general procedure 8 condition A to obtain the fully protected dihydrosansanmycin analogue. This compound was treated with a mixture of TFA and *i*Pr_3SiH in CH_2Cl_2 (1:1 v/v TFA: CH_2Cl_2, 2.5 vol.% *i*Pr_3SiH, 2.7 mL) according to general procedure 8 to afford dihydrosansanmycin **8** (as a formate salt) after reverse phase HPLC purification (0 to 50% MeCN over 40 min, 10 min at 100% H_2O) as an amorphous white solid (11 mg, 46% over 2 steps).

IR (ATR): 2951, 2836, 1662 cm^{-1}. **^{1}H NMR** (500 MHz, CD_3OD, rotamers in 1.3:1 ratio, *rotamer 1*): δ 8.69-8.64 (1H, m, NH), 8.63-8.57 (1H, m, NH), 8.57-8.49 (1H, m, NH), 8.12 (1H, d, *J* = 7.5 Hz, Ar-H), 7.98 (1H, d, *J* = 8.0 Hz, Ar-H), 7.66-7.59 (2H, m, Ar-H + H-6), 7.58-7.51 (1H, m, Ar-H), 7.35-7.23 (1H, m, Ar-H), 7.11 (1H, s, Ar-H), 7.10-7.04 (1H, m, Ar-H), 7.03-6.93 (1H, m, Ar-H), 5.74 (1H, d, *J* = 2.7 Hz, H-1'), 5.69 (1H, d, *J* = 8.0 Hz, H-5), 5.00-4.91 (1H, m, Pal2-α-CH), 4.89-4.81 (1H, m, DABA1-β-CH), 4.76-4.72 (1H, m, DABA1-α-CH), 4.64-4.37 (3H, m, H-2' + H-4' + Trp4-α-CH), 4.30-4.04 (2H, m, Leu3-α-CH), 3.52-3.41 (1H, m, H-5'), 3.30-3.24 (2H, m, H-5' + Trp4-β-CH_2), 3.23-3.11 (3H, m, Pal2-β-CH_2 + Trp4-β-CH_2), 2.81 (3H, s, NCH_3), 2.31 (1H, dt, *J* = 13.8, 6.9 Hz, H-2'), 2.23 (1H, ddd, *J* = 13.7, 7.5, 6.2 Hz, H-2'), 1.86-1.77 (1H, m, H-2'), 1.78-1.61 (3H, m, H-2' + Leu3-γ-CH), 1.60-1.40 (2H, m, Leu3-β-CH_2), 1.28 (3H, d, *J* = 7.1 Hz, DABA1-γ-CH_3), 1.24 (3H, d, *J* = 7.1

Hz, DABA1-γ-CH_3), 0.98-0.86 (6H, m, 2× Leu3-δ-CH_3). **^{13}C NMR** (125 MHz, CD_3OD, ratio 1.3:1, *rotamer 1*): δ 176.0 (C=O), 175.6 (C=O), 171.3 (C=O), 169.2 (C=O), 166.1 (C=O), 159.8 (C=O), 151.9 (C=O), 147.6, 144.4, 142.0, 141.6, 137.9, 128.9, 126.5, 124.6 (×2), 122.2, 119.6, 119.1, 111.9, 110.6, 102.2, 94.9, 80.6, 76.2, 56.4, 54.7, 54.3, 53.0, 52.3, 44.6, 41.6, 35.6, 34.8, 28.8, 28.4, 25.4, 22.4, 12.5. **^{1}H NMR** (500 MHz, CD_3OD, rotamers in 1.3:1 ratio, *rotamer 2*): δ 8.06 (1H, d, *J* = 8.0 Hz, Ar-H), 7.48 (1H, d, *J* = 8.1 Hz, H-6), 5.77 (1H, d, *J* = 3.0 Hz, H-1'), 5.64 (1H, d, *J* = 8.1 Hz, H-5), 5.11-5.04 (1H, m, DABA1-β-CH), 4.64-4.37 (2H, m, DABA1-α-CH + Pal2-α-CH), 3.40-3.32 (1H, m, Pal2-β-CH_2), 3.11-3.05 (1H, m, Pal2-β-CH_2), 3.01 (3H, s, NCH_3); **^{13}C NMR** (125 MHz, CD_3OD, ratio 1.3:1, *rotamer 2*): δ 165.7 (C=O), 146.7, 141.7, 51.9, 33.4, 30.6, 12.2. **LRMS** [*M*+H$^+$] 833.4. **HRMS (ESI m/z)** [*M*+H$^+$] calcd. for $C_{40}H_{52}N_{10}O_{10}$ 833.3940, found 833.3938.

(((*S*)-1-(((2*S*,3*S*)-3-((*S*)-2-amino-*N*-methyl-3-(naphthalen-2-yl)propanamido)-1-((((2*R*,4*R*,5*R*)-5-(2,4-dioxo-3,4-dihydropyrimidin-1(2*H*)-yl)-4-hydroxytetrahydrofuran-2-yl)methyl)amino)-1-oxobutan-2-yl)amino)-4-methyl-1-oxopentan-2-yl)carbamoyl)-*L*-tryptophan (9)

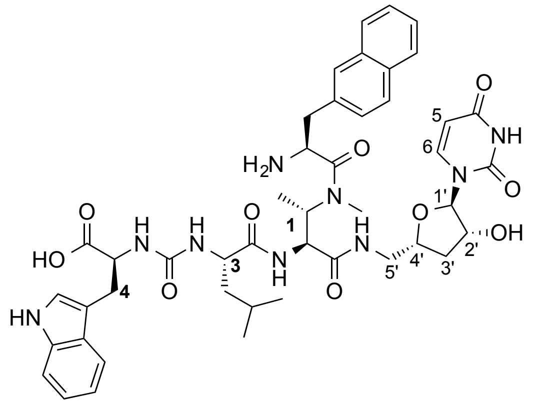

Depsipeptide **S27** (30 mg, 33 μmol) was reacted with amine **18** (9.8 mg, 40 μmol) in CH_2Cl_2: DMF (1:1 v/v, 320 μL) in the presence of HOAt (23 mg, 165 μmol) and DIC (5.3 μL, 33 μmol) for 2.5 h at rt according to general procedure 8 condition A to obtain the fully protected dihydrosansanmycin analogue. This compound was treated with a mixture of TFA and *i*Pr_3SiH in CH_2Cl_2 (1:1 v/v TFA: CH_2Cl_2, 2.5 vol.% *i*Pr_3SiH, 3.3 mL) according to general procedure 8 to afford dihydrosansanmycin **9** (as a formate salt) after reverse phase HPLC purification (0 to 50% MeCN over 40 min, 10 min at 100% H_2O) as an amorphous white solid (10 mg, 32% over 2 steps).

IR (ATR): 3264, 3056, 2954, 2918, 2851, 1672, 1636 cm^{-1}. **^{1}H NMR** (500 MHz, DMSO-d_6, rotamers in 1.8:1 ratio, *major rotamer*): 10.78 (1H, s), 8.52 (1H, s), 8.32 (1H, s), 8.20 (2H, s), 7.88-7.77 (3H, m, Ar-H), 7.65 (1H, *app.* s, Ar-H), 7.58 (1H, d, *J* = 8.1 Hz, H-6), 7.54-7.39 (1H, m, Ar-H), 7.47-7.35 (3H, m, Ar-H), 7.32-7.27 (1H, m, Ar-H), 7.12-7.07 (1H, m, Ar-H), 7.05-6.99 (1H, m, Ar-H), 6.97-6.88 (1H, m, Ar-H), 6.46 (1H, d, *J* = 8.0 Hz), 6.39 (1H, d, *J* = 8.0 Hz), 6.13 (1H, s), 5.64 (1H, d, H-1'), 5.58 (1H, d, H-5), 4.46 (1H, *app.* t, *J* = 9.2 Hz, DABA1-α-CH), 4.39-4.24 (3H, m, H-2' + H-4' + Trp4-α-CH), 4.24-4.08 (3H, m, DABA1-β-CH + Nal2-α-CH + Leu3-α-CH), 3.34-3.28 (1H, m, H-5'), 3.12-2.96 (3H, H-5' + Trp4-β-CH_2), 2.91-2.86 (1H, m, Nal2-β-CH_2), 2.81 (1H, dd, *J* = 13.2, 6.7 Hz, Nal2-β-CH_2), 2.60 (3H, s, NCH_3), 2.58-2.52 (1H, m, Nal2-β-CH_2), 2.10 (1H, *app.* dt, *J* = 13.4, 6.8 Hz, H-3'), 1.68-1.50 (2H, m, H-3' + Leu3-γ-CH), 1.36-1.28 (2H, m, Leu3-β-CH_2), 0.89-0.76 (6H, m, 2× Leu3-δ-CH_3), 0.63 (3H, d, *J* = 6.5 Hz, DABA1-γ-CH_3). **^{13}C NMR** (125 MHz, DMSO-d_6, rotamers in 1.8:1 ratio, *major rotamer*): δ 174.3 (C=O), 172.8 (C=O), 169.9 (C=O), 164.6 (C=O), 163.2 (C=O), 157.3 (C=O), 150.4 (C=O), 141.8, 136.2, 135.9, 135.7, 132.0, 131.7, 128.0, 127.8, 127.6, 127.5, 127.4 (×2), 127.3, 125.4, 123.4, 120.4, 118.4, 118.0, 111.0, 110.0, 101.4, 92.1, 78.8, 74.0, 55.2, 54.1, 51.8, 51.6, 43.4, 41.2, 40.1, 35.2, 27.9, 27.3, 23.7, 21.8, 14.5. **LRMS** [M+H^+] 882.4. **HRMS (ESI m/z)** [M+H^+] calcd. for $C_{45}H_{55}N_9O_{10}$ 882.4144, found 882.4140.

(((*S*)-1-(((2*S*,3*S*)-3-((*S*)-2-amino-3-cyclohexyl-*N*-methylpropanamido)-1-((((2*R*,4*R*,5*R*)-5-(2,4-dioxo-3,4-dihydropyrimidin-1(2*H*)-yl)-4-hydroxytetrahydrofuran-2-yl)methyl)amino)-1-oxobutan-2-yl)amino)-4-methyl-1-oxopentan-2-yl)carbamoyl)-*L*-tryptophan (10)

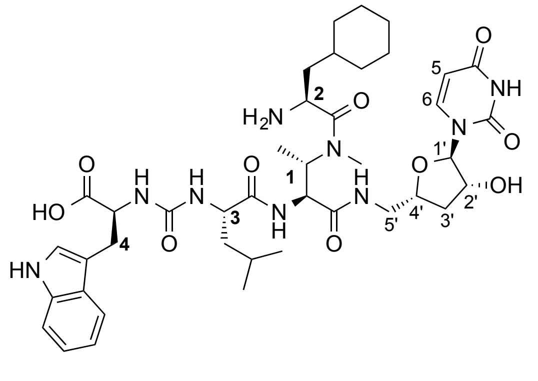

Depsipeptide **S28** (22 mg, 25 μmol) was reacted with amine **18** (6.8 mg, 30 μmol) in CH_2Cl_2: DMF (1:1 v/v, 240 μL) in the presence of HOAt (17 mg, 123 μmol) and EDC.HCl (5.2 mg, 25 μmol) and NMM (3 μL, 25 μmol) for 3 h at rt according to

general procedure 8 condition B to obtain the fully protected dihydrosansanmycin analogue. This compound was treated with a mixture of TFA and *i*Pr_3SiH in CH_2Cl_2 (1:1 v/v TFA: CH_2Cl_2, 2.5 vol.% *i*Pr_3SiH, 2.5 mL) according to general procedure 8 to afford dihydrosansanmycin **10** (as a TFA salt) after reverse phase HPLC purification (0 to 50% MeCN over 40 min, 10 min at 100% H_2O) as an amorphous white solid (10.4 mg, 44% over 2 steps).

IR (ATR): 3346, 2924, 1728, 1627 cm^{-1}. ^{1}**H NMR** (500 MHz, CD_3OD, rotamers in 1.6:1 ratio, *major rotamer*): δ 8.67 (1H, t, *J* = 6.0 Hz), 8.45 (1H, d, *J* = 9.0 Hz), 8.16-8.05 (1H, m), 7.62 (1H, d, *J* = 8.1 Hz, H-6), 7.58 (1H, dd, *J* = 7.9, 4.0 Hz, Ar-H), 7.33 (1H, dd, *J* = 8.1, 5.6 Hz, Ar-H), 7.14-7.05 (2H, m, Ar-H), 7.03-6.98 (1H, m, Ar-H), 5.74 (1H, d, *J* = 2.7 Hz, H-1'), 5.70 (1H, d, *J* = 8.0 Hz, H-5), 4.88-4.78 (1H, m, DABA1-β-CH), 4.67-4.54 (3H, m, DABA1-α-CH + Cha2-α-CH + Trp4-α-CH), 4.49-4.38 (2H, m, H-2' + H-4'), 4.19-4.08 (1H, m, Leu3-α-CH), 3.52-3.46 (1H, m, H-5'), 3.29-3.23 (2H, m, H-5' major + Trp4-β-CH_2), 3.24-3.14 (1H, m, Trp4-β-CH_2), 2.85 (3H, s, NCH_3), 2.30-2.18 (1H, m, H-3'), 1.84-1.55 (10H, m, 4× CH_2 + H-3' + Leu3-γ-CH), 1.52-1.40 (3H, CH_2 + Cha2-γ-CH), 1.36-1.26 (2H, m, Leu3-β-CH_2), 1.24-1.10 (3H, m, DABA1-γ-CH_3), 1.07-0.95 (2H, m, CH_2), 0.94-0.86 (6H, m, 2× Leu3-δ-CH_3). ^{13}**C NMR** (125 MHz, CD_3OD, rotamers in 1.6:1 ratio, *major rotamer*): δ 176.2 (C=O), 175.6 (C=O), 171.8 (C=O), 171.0 (C=O), 166.3 (C=O), 159.8 (C=O), 152.3 (C=O), 142.2, 137.9, 128.6, 124.4, 122.0, 119.4, 119.2, 111.9, 110.6, 102.2, 95.0, 80.9, 76.1, 54.9, 54.0, 52.6, 50.3, 44.8, 41.8, 39.6, 36.0, 34.8, 34.2, 29.0, 28.3, 26.9, 25.6, 22.9, 14.6. **LRMS** [$M+H^+$] 838.4. **HRMS (ESI m/z)** [$M+Na^+$] calcd. for $C_{46}H_{72}N_6O_{11}Na$ 907.5151, found 907.5150.

(((*S*)-1-(((2*S*,3*S*)-3-((*S*)-2-amino-2-cyclohexyl-*N*-methylacetamido)-1-((((2*R*,4*R*,5*R*)-5-(2,4-dioxo-3,4-dihydropyrimidin-1(2*H*)-yl)-4-hydroxytetrahydrofuran-2-yl)methyl)amino)-1-oxobutan-2-yl)amino)-4-methyl-1-oxopentan-2-yl)carbamoyl)-*L*-tryptophan (11)

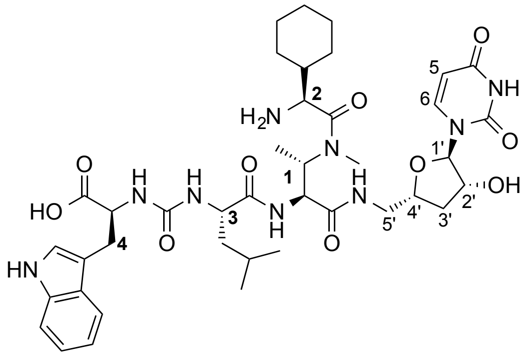

Depsipeptide **S29** (19 mg, 22 μmol) was reacted with amine **18** (5.8 mg, 26 μmol) in CH_2Cl_2: DMF (1:1 v/v, 200 μL) in the presence of HOAt (14 mg, 107 μmol) and DIC (5.2 μL, 33 μmol) for 18 h at rt according to general procedure 8 condition A to obtain the fully protected dihydrosansanmycin analogue. This compound was treated with a mixture of TFA and *i*Pr_3SiH in CH_2Cl_2 (1:1 v/v TFA: CH_2Cl_2, 2.5 vol.% *i*Pr_3SiH, 2.2 mL) according to general procedure 8 to afford dihydrosansanmycin **11** (as a TFA salt) after reverse phase HPLC purification (0 to 50% MeCN over 40 min, 10 min at 100% H_2O) as an amorphous white solid (5.4 mg, 26% over 2 steps).

IR (ATR): 3261, 2931, 2856, 1665 cm^{-1}. **^{1}H NMR** (400 MHz, CD_3OD, rotamers in 1.6:1 ratio, *major rotamer*): δ 10.30 (1H, s, NH), 8.67 (1H, s, NH), 8.12 (1H, s, NH), 7.62 (1H, d, *J* = 8.1 Hz, H-6), 7.58 (1H, dd, *J* = 8.0, 3.0 Hz, Ar-H), 7.39-7.24 (1H, m, Ar-H), 7.18-7.03 (2H, m, Ar-H), 7.04-6.98 (1H, m, Ar-H), 5.74 (1H, d, *J* = 2.7 Hz, H-1'), 5.71 (1H, d, *J* = 8.0 Hz, H-5), 4.91-4.78 (1H, m, DABA1-β-CH), 4.71-4.53 (1H, Trp4-α-CH), 4.51-4.37 (2H, m, H-2' + H-4'), 4.30-4.22 (1H, m, DABA1-α-CH), 4.20-4.08 (1H, m, Leu3-α-CH), 4.03 (1H, d, *J* = 4.6 Hz, Chg2-α-CH), 3.55-3.45 (1H, m, H-5'), 3.29-3.25 (2H, m, H-5' major + Trp4-β-CH_2), 3.22-3.09 (1H, m, Trp4-β-CH_2), 2.86 (3H, s, NCH_3), 2.35-2.16 (1H, m, H-2'), 1.87-1.54 (6H, m, H-2' + Chg2-β-CH + 1.5 × Chg2-CH_2 + Leu3-γ-CH), 1.53-1.42 (2H, m, Leu3-β-CH_2), 1.35-1.02 (6H, m, DABA1-γ-CH_3 + 1.5 × Chg2-CH_2, major), 0.91 (6H, m, 2× Leu3-δ-CH_3 major). **^{13}C NMR** (100 MHz, CD_3OD, *major rotamer*): δ 176.1 (C=O), 175.1 (C=O),

171.4 (C=O), 169.7 (C=O), 166.0 (C=O), 159.5 (C=O), 151.9 (C=O), 142.3, 137.8, 128.6, 124.7, 122.0, 119.6, 119.2, 111.9, 110.5, 109.1, 102.3, 95.0, 80.8, 76.2, 56.4, 55.0, 54.4, 53.8, 52.8, 44.6, 43.6, 42.0, 40.5, 35.8, 30.2, 29.0, 28.3, 26.7, 25.7, 23.4, 15.2. **LRMS** [*M*+H$^+$] 824.4. **HRMS (ESI m/z)** [*M*+H$^+$] calcd. for $C_{40}H_{57}N_9O_{10}$ 824.4301, found 824.4294.

(((*S*)-1-(((2*S*,3*S*)-3-(2-amino-*N*-methylacetamido)-1-((((2*R*,4*R*,5*R*)-5-(2,4-dioxo-3,4-dihydropyrimidin-1(2*H*)-yl)-4-hydroxytetrahydrofuran-2-yl)methyl)amino)-1-oxobutan-2-yl)amino)-4-methyl-1-oxopentan-2-yl)carbamoyl)-*L*-tryptophan (12)

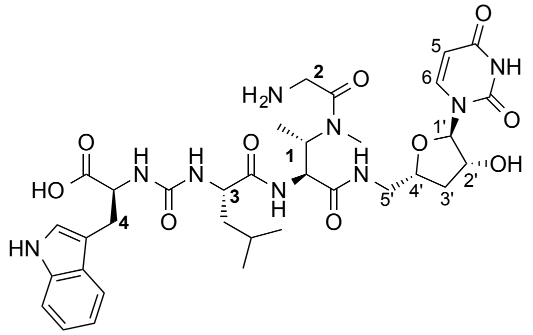

Depsipeptide **S30** (14.5 mg, 18 μmol) was reacted with amine **18** (5.0 mg, 22 μmol) in CH_2Cl_2: DMF (1:1 v/v, 180 μL) in the presence of HOAt (13 mg, 95 μmol) and DIC (2.8 μL) for 1.5 h at rt according to general procedure 8 condition A to obtain fully protected dihydrosansanmycin analogue. This compound was treated with a mixture of TFA and *i*Pr_3SiH in CH_2Cl_2 (1:1 v/v TFA: CH_2Cl_2, 2.5 vol.% *i*Pr_3SiH, 1.5 mL) according to general procedure 8 to afford dihydrosansanmycin **12** (as formate salt) after reverse phase HPLC purification (0 to 50% MeCN over 40 min, 10 min at 100% H_2O) as a fluffy white solid (10 mg, 69% over 2 steps).

IR (ATR): 3300, 2923, 1688, 1548 cm^{-1}. **^{1}H NMR** (500 MHz, D_2O: CD_3OD, 2:1 v/v, rotamers in 1:1 ratio, *rotamer 1*): δ 7.68-7.60 (1H, m, Ar-H), 7.58 (1H, d, *J* = 8.1 Hz, H-6), 7.33 (1H, *app.* t, *J* = 7.7 Hz, Ar-H), 7.16 (1H, s, Ar-H), 7.10-7.04 (1H, m, Ar-H), 7.02 (1H, dd, *J* = 7.6, 4.1 Hz, Ar-H), 5.76 (1H, d, *J* = 8.0 Hz, H-5), 5.72 (1H, d, *J* = 2.8 Hz, H-1'), 4.78-4.74 (1H, m, DABA1-β-CH), 4.58-4.53 (2H, m, H-4' + DABA1-α-CH), 4.48-4.26 (2H, m, H-2' + Trp4-α-CH), 4.19-3.99 (2H, m, Gly2-α-CH_2, Leu3-α-CH), 3.91 (1H, d, *J* = 16.2 Hz, Gly2-α-CH_2), 3.45-3.33 (2H, m, H-5'), 3.33-3.28 (1H, m, Trp4-β-CH_2), 3.14 (1H, dd, *J* = 14.5, 7.1 Hz, Trp4-β-CH_2), 2.86 (3H, s, NCH_3), 2.36-2.12 (1H, m, H-3'), 1.88-1.68 (1H, m, H-3'), 1.61-1.39 (3H, m,

Leu3-β-CH_2 + Leu3-γ-CH), 1.30-1.07 (3H, m, DABA1-γ-CH_3), 0.90-0.75 (6H, m, 2× Leu3-δ-CH_3). **^{13}C NMR** (125 MHz, D_2O: CD_3OD, 2:1 v/v, mixture of rotamers with 1:1 ratio, *rotamer 1*): δ 176.4 (C=O), 171.8 (C=O), 169.9 (C=O), 167.6 (C=O), 166.6 (C=O), 159.8 (C=O), 152.4 (C=O), 142.9, 137.8, 129.1, 124.7, 122.3, 119.8, 119.7, 112.3, 111.9, 102.7, 95.1, 80.9, 76.3, 57.2, 56.8, 54.3, 53.2, 44.9, 41.7, 41.3, 35.9, 29.6, 28.4, 25.7, 23.4, 22.2, 15.1. **^{1}H NMR** (500 MHz, D_2O: CD_3OD, 2:1 v/v, rotamers in 1:1 ratio, *rotamer 2*): δ 7.68-7.60 (1H, m, Ar-H), 7.49 (1H, d, *J* = 8.0 Hz, H-6), 7.33 (1H, *app.* t, *J* = 7.7 Hz), 7.12 (1H, s, Ar-H), 7.10-7.04 (1H, m, Ar-H), 7.02 (1H, dd, *J* = 7.6, 4.1 Hz, Ar-H), 5.72 (1H, d, *J* = 2.8 Hz, H-1'), 5.70 (1H, d, *J* = 8.1 Hz, H-5), 4.62-4.58 (1H, m, DABA1-α-CH), 4.48-4.26 (3H, m, H-2' + H-4' + Trp4-α-CH), 4.19-3.99 (1H, m, DABA1-β-CH + Leu3-α-CH), 3.84-3.70 (2H, m, Gly2-α-CH_2), 3.57-3.41 (1H, m, H-5'), 3.33-3.28 (1H, m, Trp4-β-CH_2), 3.28-3.19 (1H, m, H-5'), 3.14 (1H, dd, *J* 14.5, 7.1 Hz, Trp4-β-CH_2), 2.75 (3H, s, NCH_3), 2.36-2.12 (1H, m, H-3'), 1.88-1.68 (1H, m, H-3'), 1.61-1.39 (3H, m, Leu3-β-CH_2 + Leu3-γ-CH), 1.30-1.07 (3H, m, DABA1-γ-CH_3), 0.90-0.75 (6H, m, 2× Leu3-δ-CH_3). **^{13}C NMR** (125 MHz, D_2O: CD_3OD, 2:1 v/v, mixture of rotamers with 1:1 ratio, *rotamer 2*): δ 176.0 (C=O), 171.8 (C=O), 169.9 (C=O), 167.6 (C=O), 166.5 (C=O), 159.8 (C=O), 152.3 (C=O), 142.6, 137.8, 129.1, 124.7, 122.2, 119.7, 119.6, 111.7, 112.2, 102.6, 95.0, 81.1, 76.2, 57.2, 56.8, 54.5, 54.0, 45.0, 42.7, 41.5, 36.2, 30.6, 29.6, 25.7, 23.5, 23.3, 15.1. **LRMS** [M+H^+] 742.5. **HRMS (ESI m/z)** [M+H^+] calcd. for $C_{34}H_{48}N_9O_{10}$ 742.3518, found 742.3519.

(((*S*)-1-(((2*S*,3*S*)-3-((*S*)-2-amino-*N*-methylpropanamido)-1-((((2*R*,4*R*,5*R*)-5-(2,4-dioxo-3,4-dihydropyrimidin-1(2*H*)-yl)-4-hydroxytetrahydrofuran-2-yl)methyl)amino)-1-oxobutan-2-yl)amino)-4-methyl-1-oxopentan-2-yl)carbamoyl)-*L*-tryptophan (13)

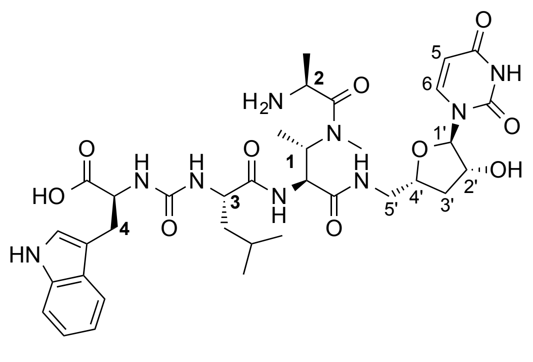

Depsipeptide **S31** (21 mg, 26 μmol) was reacted with amine **18** (6.8 mg, 30 μmol) in CH_2Cl_2: DMF (1:1 v/v, 260 μL) in the presence of HOAt (17 mg, 130 μmol) and DIC (6.1 μL, 39 μmol) for 3 h at rt according to general procedure 8 condition A to obtain the fully protected dihydrosansanmycin analogue. This compound was treated with a mixture of TFA and *i*Pr_3SiH in CH_2Cl_2 (1:1 v/v TFA: CH_2Cl_2, 2.5 vol.% *i*Pr_3SiH, 2.0 mL) according to general procedure 8 to afford dihydrosansanmycin **13** (as a formate salt) after reverse phase HPLC purification (0 to 50% MeCN over 40 min, 10 min at 100% H_2O) as an amorphous white solid (12.4 mg, 58% over 2 steps).

IR (ATR): 2957, 1654, 1547 cm^{-1}. **^{1}H NMR** (400 MHz, CD_3OD, rotamers in 1.7:1 ratio, *major rotamer*): δ 8.79 (0.6 H, m), 8.16-8.13 (0.5 H, m), 7.66 (1H, *app.* d, *J* = 8.1 Hz, H-6), 7.63-7.57 (1H, m, Ar-H), 7.38-7.31 (1H, m, Ar-H), 7.13 (1H, s, Ar-H), 7.12-7.07 (1H, m, Ar-H), 7.06-6.99 (1H, m, Ar-H), 5.76 (1H, d, *J* = 2.6 Hz, H-1'), 5.73 (1H, d, *J* = 8.1 Hz, H-5), 4.68-4.54 (3H, DABA1-α-CH + Ala2-α-CH + Trp4-α-CH), 4.49-4.44 (1H, m, H-4'), 4.41 (1H, *app.* tdd, *J* = 8.6, 5.5, 3.5 Hz, H-2'), 4.24-4.03 (2H, m, DABA1-β-CH + Leu3-α-CH), 3.44-3.38 (1H, m, H-5'), 3.31-3.25 (2H, m, H-5' + Trp4-β-CH_2), 3.24-3.12 (1H, m, Trp4-β-CH_2), 2.88 (3H, s, NCH_3), 2.36-2.18 (1H, m, H-3'), 1.85-1.59 (2H, m, H-3' + Leu3-γ-CH), 1.54-1.47 (2H, m, Leu3-γ-CH), 1.44 (3H, d, *J* = 7.0 Hz, Ala2-β-CH_3), 1.25 (3H, d, *J* = 6.5 Hz, DABA1-γ-CH_3), 1.20 (3H, d, *J* = 7.1 Hz, DABA1-γ-CH_3), 0.97-0.89 (6H, m, 2× Leu3-δ-CH_3). **^{13}C NMR** (100 MHz, CD_3OD, rotamers in 1.7:1 ratio, *major rotamer*): δ 176.5 (C=O), 175.6 (C=O), 171.5 (C=O), 170.4 (C=O), 166.2 (C=O), 159.8 (C=O), 152.2 (C=O), 142.6 (C-6), 137.8, 128.7, 124.6, 122.4, 119.7, 119.5, 115.2, 110.5, 102.3, 95.0, 81.2, 76.2, 57.3, 55.0, 54.0, 48.7, 48.7, 44.9, 41.9, 35.8, 29.4, 28.6, 25.8, 17.1, 23.4, 14.7. **LRMS** [*M*+H^+] 756.4. **HRMS (ESI m/z)** [*M*+Na^+] calcd. for $C_{35}H_{49}N_9O_{10}Na$ 778.3494, found 778.3498.

(2*S*,6*S*,9*S*,10*S*,13*S*)-2-((1*H*-indol-3-yl)methyl)-13-amino-9-((((2*R*,4*R*,5*R*)-5-(2,4-dioxo-3,4-dihydropyrimidin-1(2*H*)-yl)-4-hydroxytetrahydrofuran-2-yl)methyl)carbamoyl)-6-isobutyl-10,11-dimethyl-4,7,12-trioxo-3,5,8,11-tetraazapentadecanedioic acid (14)

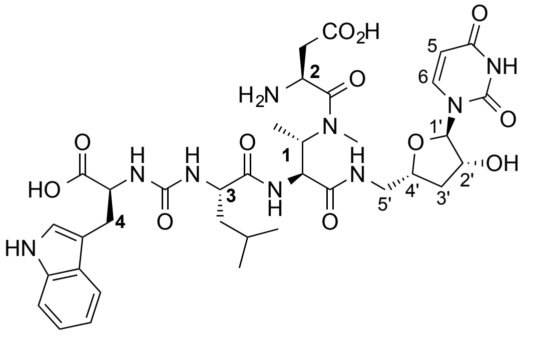

Depsipeptide **S32** (19 mg, 21 μmol) was reacted with amine **18** (5.5 mg, 25 μmol) in CH_2Cl_2: DMF (1:1 v/v, 200 μL) in the presence of HOAt (15 mg, 110 μmol) and DIC (3.3 μL) for 1.5 h at rt according to general procedure 8 condition A to obtain fully protected dihydrosansanmycin analogue. This compound was treated with a mixture of TFA and *i*Pr_3SiH in CH_2Cl_2 (1:1 v/v TFA: CH_2Cl_2, 2.5 vol.% *i*Pr_3SiH, 1.8 mL) according to general procedure 8 to afford dihydrosansanmycin **14** (as a formate salt) after reverse phase HPLC purification (0 to 50% MeCN over 40 min, 10 min at 100% H_2O) as a fluffy white solid (6.5 mg, 37% over 2 steps).

IR (ATR): 3675, 2957, 1675, 1587 cm^{-1}. **^{1}H NMR** (600 MHz, D_2O, rotamers in 2:1 ratio, *major rotamer*): δ 7.67 (1H, *app.* d, *J* = 8.4 Hz, Ar-H), 7.57-7.38 (2H, m, H-6 + Ar-H), 7.29-7.19 (2H, m, Ar-H), 7.16 (1H, *app.* dt, *J* = 8.1, 5.6 Hz, Ar-H), 5.76 (1H, d, *J* = 8.6 Hz, H-5), 5.72-5.70 (1H, m, H-1'), 4.86-4.83 (1H, m, DABA1-β-CH), 4.59 (1H, d, *J* = 9.0 Hz, DABA1-α-CH), 4.57-4.52 (1H, m, H-4'), 4.50-4.41 (3H, m, H-2' + Asp2-α-CH + Trp4-α-CH), 4.15-4.05 (1H, m, Leu3-α-CH), 3.52 (1H, dd, *J* = 14.7, 6.7 Hz, H-5'), 3.38-3.35 (1H, m, H-5'), 3.31-3.28 (1H, m, Trp4-β-CH_2), 3.15 (1H, td, *J* 16.1, 7.5 Hz, Trp4-β-CH_2), 2.81 (3H, s, NCH_3), 2.60-2.57 (1H, m, Asp2-β-CH_2), 2.51 (1H, dd, *J* = 17.6, 10.8 Hz, Asp2-β-CH_2), 2.33-2.24 (1H, m, H-3'), 1.87-1.65 (1H, m, H-3'), 1.59-1.40 (3H, m, Leu3-β-CH_2+ Leu3-γ-CH), 1.15 (3H, d, *J* = 6.9 Hz, DABA1-γ-CH_3), 0.86 (6H, d, *J* = 7.2 Hz, 2× Leu3-δ-CH_3). **^{13}C NMR** (150 MHz, D_2O, rotamers in 2:1 ratio, *major rotamer*): δ 178.1 (C=O), 176.0 (C=O), 175.0 (C=O), 170.6 (C=O), 169.1 (C=O), 166.0 (C=O), 158.7 (C=O), 151.2 (C=O), 141.6, 136.0, 126.8, 124.3, 121.7, 118.9, 118.4, 111.6, 109.9, 101.9, 92.9, 79.8, 74.8, 55.5,

55.3, 55.2, 51.1, 43.0, 40.0, 35.2, 34.3, 29.7, 27.8, 24.2, 22.0, 20.8, 12.6. **LRMS** [M+H$^+$] 800.5. **HRMS (ESI m/z)** [M+Na$^+$] calcd. for $C_{36}H_{49}N_9O_{12}Na$ 822.3400, found 822.3402.

(((*S*)-1-(((2*S*,3*S*)-3-((*S*)-2,6-diamino-*N*-methylhexanamido)-1-((((2*R*,4*R*,5*R*)-5-(2,4-dioxo-3,4-dihydropyrimidin-1(2*H*)-yl)-4-hydroxytetrahydrofuran-2-yl)methyl)amino)-1-oxobutan-2-yl)amino)-4-methyl-1-oxopentan-2-yl)carbamoyl)-*L*-tryptophan (15)

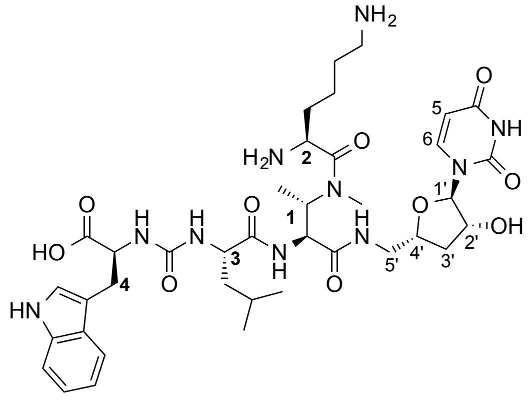

Depsipeptide **S33** (15 mg, 16 μmol) was reacted with amine **18** (4.4 mg, 19 μmol) in CH_2Cl_2: DMF (1:1 v/v, 160 μL) in the presence of HOAt (12 mg, 88 μmol) and DIC (5.3 μL, 16 μmol) for 1.5 h at rt according to general procedure 8 condition A to obtain the fully protected dihydrosansanmycin analogue. This compound was treated with a mixture of TFA and *i*Pr_3SiH in CH_2Cl_2 (1:1 v/v TFA: CH_2Cl_2, 2.5 vol.% *i*Pr_3SiH, 1.5 mL) according to general procedure 8 to afford dihydrosansanmycin **15** (as a formate salt) after reverse phase HPLC purification (0 to 50% MeCN over 40 min, 10 min at 100% H_2O) as an amorphous white solid (9.2 mg, 65% over 2 steps).

IR (ATR): 3392, 1572 cm^{-1}. ^{1}**H NMR** (400 MHz, CD_3OD, rotamers in 1.2:1 ratio, *rotamer 1*): δ 7.64 (1H, d, J = 8.1 Hz, H-6), 7.61 (1H, *app.* d, J = 7.9 Hz, Ar-H), 7.37-7.30 (1H, m, Ar-H), 7.15-6.99 (3H, m, Ar-H), 5.74 (1H, d, J = 2.5 Hz, H-1'), 5.70 (1H, d, J = 8.1 Hz, H-5), 4.63-4.38 (4H, m, H-2' + H-4' + DABA1-α-CH + Lys2-α-CH), 4.27-4.20 (1H, m, DABA1-β-CH), 4.16-4.02 (1H, m, Leu3-α-CH), 3.52-3.37 (2H, m, H-5'), 3.26-3.12 (2H, m, Trp4-β-CH_2), 2.93 (2H, t, J = 7.9 Hz, Lys2-ε-CH_2), 2.86 (3H, s, NH_3), 2.34-2.18 (1H, m, H-3'), 1.84-1.28 (10H, m), 1.21 (3H, d, J = 6.7 Hz, DABA1-γ-CH_3), 0.98-0.86 (6H, m, 2× Leu3-δ-CH_3). ^{13}**C NMR** (101 MHz, D_2O, rotamers in 1.5:1 ratio, *rotamer 1*): δ 176.3 (C=O), 170.8 (C=O), 169.3 (C=O), 166.4

(C=O), 166.2 (C=O), 158.8 (C=O), 151.3 (C=O), 141.6, 136.1, 127.2, 124.2, 121.7, 119.2, 118.7, 111.7, 110.4, 101.8, 93.0, 79.6, 74.8, 56.4, 55.6, 53.0, 51.3, 50.4, 43.2, 39.9, 38.7, 34.4, 28.2. 28.1, 26.6, 24.2, 21.1, 21.0, 12.5. **^{1}H NMR** (400 MHz, CD_3OD, rotamers in 1.2:1 ratio, *rotamer 2*): δ 7.57 (1H, *app.* d, *J* = 7.6, Ar-H), 7.49 (1H, d, *J* = 8.1 Hz, H-6), 5.76 (1H, d, *J* = 3.0 Hz, H-1'), 5.65 (1H, d, *J* = 8.1 Hz, H-5), 4.68-4.63 (1H, m, DABA1-β-CH), 2.87 (3H, s, NH_3), 2.85-2.76 (2H, m, Lys2-ε-CH_2). **^{13}C NMR** (101 MHz, D_2O, rotamers in 1.5:1 ratio, *rotamer 2*): δ 175.9 (C=O), 170.6 (C=O), 30.2, 27.4, 21.0, 14.1. **LRMS** [*M*+H$^+$] 799.5. **HRMS (ESI m/z)** [*M*+H$^+$] calcd. for $C_{37}H_{55}N_{10}O_{10}$ 799.4097, found 799.4102.

(((*S*)-1-(((2*S*,3*S*)-3-((*S*)-2,5-diamino-*N*-methylpentanamido)-1-((((2*R*,4*R*,5*R*)-5 -(2,4-dioxo-3,4-dihydropyrimidin-1(2*H*)-yl)-4-hydroxytetrahydrofuran-2-yl)methyl)amino)-1-oxobutan-2-yl)amino)-4-methyl-1-oxopentan-2-yl)carbamoyl)-*L*-tryptophan (16)

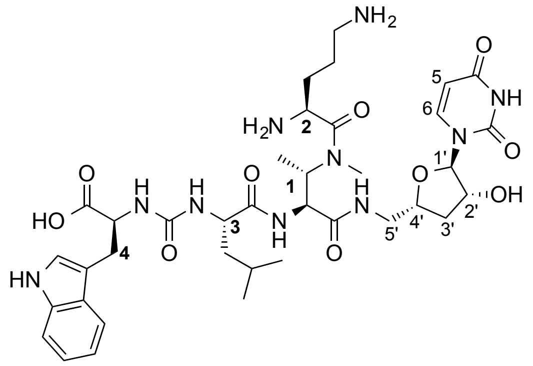

Isopeptide **S20** (133 mg, 195 μmol) was loaded onto 2-chlorotrityl chloride resin (80.0 mg, 98.0 μmol) in CH_2Cl_2 (3 mL) using *N*,*N*-diisopropylethylamine (68.0 μL, 390 μmol) and Fmoc-deprotected according to general procedure 2. Fmoc-Leu-OH (137 mg, 392 μmol) was subsequently coupled with PyBOP (203 mg, 392 μmol) and NMM (80.0 μL, 784 μmol) in DMF (0.95 mL) (general procedure 3). Carbamate **S12** (104 mg, 195 μmol) was subsequently coupled in the presence of *N*,*N*-diisopropylethylamine (70 μL, 390 μmol) in DMF (3.3 mL) according to general procedure 4. Following cleavage from the resin (general procedure 7), the peptide was purified by reverse phase HPLC (50-100% MeCN over 40 min, 10 min at 50% MeCN) to afford depsipeptide **S53** as a fluffy white solid (15.2 mg, 17%).

Depsipeptide **S53** (15.2 mg, 16.0 μmol) was reacted with amine **18** (4.40 mg, 19.0 μmol) in CH_2Cl_2: DMF (1:1 v/v, 160 μL) in the presence of HOAt (12 mg,

88 μmol) and DIC (2.5 μL) for 1.5 h at rt according to general procedure 8 condition A to obtain fully protected dihydrosansanmycin analogue. This compound was treated with a mixture of TFA and iPr$_3$SiH in CH_2Cl_2 (1:1 v/v TFA: CH_2Cl_2, 2.5 vol.% iPr$_3$SiH, 1.5 mL) according to general procedure 8 to afford dihydrosansanmycin **16** (as a formate salt) after reverse phase HPLC purification (0 to 50% MeCN over 40 min, 10 min at 100% H_2O) as an amorphous white solid (9.2 mg, 65% over 2 steps).

IR (ATR): 3292, 1572 cm^{-1}. **^{1}H NMR** (400 MHz, D_2O, rotamers in 1.5:1 ratio, *rotamer 1*): δ 7.71 (1H, dd, J = 8.0, 4.5 Hz, Ar-H), 7.53-7.48 (2H, m, H-6 + Ar-H), 7.29-7.23 (2H, m, Ar-H), 7.19 (1H, *app.* t, J = 7.4 Hz, Ar-H), 5.79 (1H, d, J = 8.0 Hz, H-5), 5.75 (1H, d, J = 2.4 Hz, H-1'), 4.89-4.85 (1H, m, DABA1-β-CH), 4.66-4.60 (1H, m, DABA1-α-CH), 4.60-4.49 (2H, m, H-2' + H-4'), 4.41-4.33 (2H, m, Orn2-α-CH + Trp4-α-CH), 4.16-4.02 (1H, m, Leu3-α-CH), 3.49 (1H, dd, J = 14.4, 6.7 Hz, H-5'), 3.43-3.36 (1H, m, H-5'), 3.35-3.27 (1H, m, Trp4-β-CH_2), 3.16-3.09 (1H, m, Trp4-β-CH_2), 3.08-3.03 (1H, m, Orn2-δ-CH_2), 2.98 (1H, *app.* t, J = 7.0 Hz, Orn2-δ-CH_2), 2.90 (3H, s, NCH_3), 2.35 (1H, *app.* dt, J = 13.8, 7.0 Hz, H-3'), 1.96-1.68 (5H, m, H-3' + Orn2-β-CH_2 + Orn2-γ-CH_2), 1.56-1.40 (3H, m, Leu3-β-CH_2 + Leu3-γ-CH), 1.23 (3H, d, J = 6.8 Hz, DABA1-γ-CH_3), 0.84 (6H, d, J = 16.1 Hz, 2× Leu3-δ-CH_3). **^{13}C NMR** (101 MHz, D_2O, rotamers in 1.5:1 ratio, *rotamer 1*): δ 176.3 (C=O), 170.8 (C=O), 169.3 (C=O), 166.4 (C=O), 166.2 (C=O), 158.8 (C=O), 151.3 (C=O), 141.6, 136.1, 127.2, 124.2, 121.7, 119.2, 118.7, 111.7, 110.4, 101.8, 93.0, 79.6, 74.8, 56.4, 55.6, 53.0, 51.3, 50.4, 43.2, 39.9, 38.7, 34.4, 28.2. 28.1, 26.6, 21.9, 24.2, 20.7, 12.5. **^{1}H NMR** (400 MHz, D_2O, rotamers in 1.5:1 ratio, *rotamer 2*): 7.56 (1H, d, J = 8.0 Hz, H-6), 5.85 (1H, d, J = 8.0 Hz, H-5), 4.68 (1H, m, Ornithine2-α-CH), 4.31-4.22 (1H, m, DABA1-β-CH). **^{13}C NMR** (101 MHz, D_2O, rotamers in 1.5:1 ratio, *rotamer 2*): δ 175.9 (C=O), 170.6 (C=O), 93.3, 79.8, 50.6, 30.2, 27.4, 24.2, 20.9, 14.1. **LRMS** [M+H$^+$] 799.5. **HRMS (ESI m/z)** [M+H$^+$] calcd. for $C_{37}H_{55}N_{10}O_{10}$ 799.4097, found 799.4102.

(((*S*)-1-(((2*S*,3*S*)-3-((2*S*,3*R*)-2-amino-3-hydroxy-*N*-methylbutanamido)-1-((((2*R*,4*R*,5*R*)-5-(2,4-dioxo-3,4-dihydropyrimidin-1(2*H*)-yl)-4-hydroxytetrahydrofuran-2-yl)methyl)amino)-1-oxobutan-2-yl)amino)-4-methyl-1-oxopentan-2-yl)carbamoyl)-*L*-tryptophan (17)

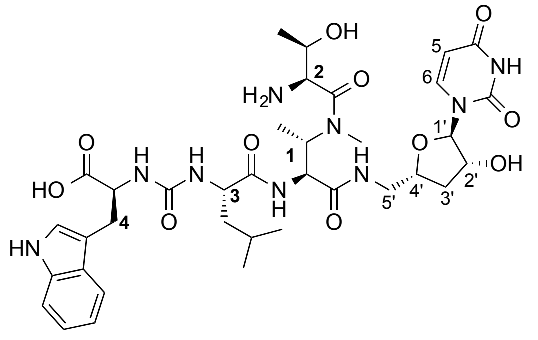

Depsipeptide **S34** (11 mg, 13 μmol) was reacted with amine **18** (3.6 mg, 15 μmol) in CH_2Cl_2: DMF (1:1 v/v, 120 μL) in the presence of HOAt (8.6 mg, 66 μmol) and DIC (2.0 μL) for 1.5 h at rt according to general procedure 8 condition A to obtain fully protected dihydrosansanmycin analogue. This compound was treated with a mixture of TFA and *i*Pr_3SiH in CH_2Cl_2 (1:1 v/v TFA: CH_2Cl_2, 2.5 vol.% *i*Pr_3SiH, 2 mL) according to general procedure 8 to afford dihydrosansanmycin **17** (as a formate salt) after reverse phase HPLC purification (0 to 50% MeCN over 40 min, 10 min at 100% H_2O) as a fluffy white solid (5.5 mg, 52% over 2 steps).

IR (ATR): 3296, 2954, 2928, 1679, 1589 cm^{-1}. **^{1}H NMR** (400 MHz, D_2O: CD_3OD, 4:1 v/v, rotamers in 1:1 ratio, *rotamer 1*): δ 7.64 (1H, *app.* d, *J* = 7.9 Hz, Ar-H), 7.45-7.43 (2H, m, H-6 + Ar-H), 7.23-7.15 (2H, m, Ar-H), 7.11 (1H, *app.* t, *J* = 7.5 Hz, Ar-H), 5.72 (1H, d, *J* = 8.0 Hz, H-5), 5.68 (1H, d, *J* = 3.0 Hz, H-1'), 4.84-4.81 (1H, m, DABA1-β-CH), 4.62-4.54 (1H, m, DABA1-α-CH), 4.52-4.40 (2H, m, H-2' + H-4'), 4.38-4.27 (1H, m, Trp4-α-CH), 4.20-4.16 (1H, m, Thr2-α-CH), 4.09-4.01 (2H, m, Thr2-β-CH + Leu3-α-CH), 3.45 (1H, dd, *J* = 14.2, 6.7 Hz, H-5'), 3.36 (1H, m, H-5'), 3.25-3.20 (1H, m, Trp4-β-CH_2), 3.08 (1H, dd, *J* = 14.7, 7.4 Hz, Trp4-β-CH_2), 2.89 (3H, s, NCH_3), 2.39-2.15 (1H, m, H-3'), 1.85-1.62 (1H, m, H-3'), 1.51-1.35 (2H, m, Leu3-β-CH_2), 1.29 (3H, d, *J* = 7.2 Hz, Thr2-γ-CH_3), 1.24-1.18 (4H, m, DABA1-γ-CH_3 + Leu3-γ-CH), 0.86-0.73 (6H, m, 2× Leu3-δ-CH_3). **^{13}C NMR** (101 MHz, D_2O: CD_3OD, 4:1 v/v, rotamers in 1:1 ratio, *rotamer 1*): δ 175.8 (C=O), 170.7 (C=O), 168.2 (C=O), 166.2 (C=O), 166.1 (C=O), 158.6 (C=O), 153.9 (C=O), 141.7,

136.1, 127.2, 124.0, 121.6, 119.0, 118.6, 111.6, 110.2, 101.7, 93.3, 79.7, 74.8, 65.6, 56.1, 55.8, 53.0, 52.9, 51.3, 43.2, 40.0, 34.4, 28.0, 24.2, 21.9, 20.9, 18.6, 13.9. **^{1}H NMR** (400 MHz, D_2O: CD_3OD, 4:1 v/v, rotamers in 1:1 ratio, *rotamer 2*): δ 7.51 (1H, d, *J* = 8.0 Hz, H-6), 5.78 (1H, d, *J* = 8.0 Hz, H-5), 2.84 (3H, s, NCH_3), 1.15 (3H, d, *J* = 7.0 Hz, DABA1-γ-CH_3). **^{13}C NMR** (101 MHz, D_2O: CD_3OD, 4:1 v/v, rotamers in 1:1 ratio, *rotamer 2*): δ 170.5 (C=O), 141.5, 93.0, 79.5, 20.7, 12.6. **LRMS** [M+H$^+$] 786.5. **HRMS (ESI m/z)** [M+H$^+$] calcd. for $C_{36}H_{52}N_9O_{11}$ 786.3781, found 786.3782.

(((2*S*,3*R*)-1-(((2*S*,3*S*)-3-(2-amino-*N*-methylacetamido)-1-((((2*R*,4*R*,5*R*)-5-(2,4-dioxo-3,4-dihydropyrimidin-1(2*H*)-yl)-4-hydroxytetrahydrofuran-2-yl)methyl)amino)-1-oxobutan-2-yl)amino)-3-methyl-1-oxopentan-2-yl)carbamoyl)-*L*-tryptophan (21)

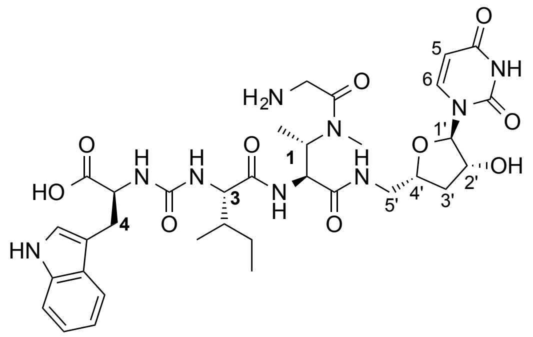

Depsipeptide **S36** (17 mg, 22 μmol) was reacted with amine **18** (7.4 mg, 32 μmol) in CH_2Cl_2: DMF (1:1 v/v, 210 μL) in the presence of HOAt (15 mg, 108 μmol) and EDC.HCl (5.4 mg, 28 μmol) and NMM (3.0 μL, 28 μmol) for 4 h at rt according to general procedure 8 condition B to obtain the fully protected dihydrosansanmycin analogue. This compound was treated with a mixture of TFA and *i*Pr_3SiH in CH_2Cl_2 (1:1 v/v TFA: CH_2Cl_2, 2.5 vol.% *i*Pr_3SiH, 2.2 mL) for 4 h according to general procedure 8 to afford dihydrosansanmycin **21** (as a formate salt) after reverse phase HPLC purification (0 to 50% MeCN over 45 min, 10 min at 100% H_2O, flow rate 9 mL/min) as an amorphous white solid (7.4 mg, 44% over 2 steps).

IR (ATR): 3374, 2979, 2932, 1731, 1666 cm^{-1}. **^{1}H NMR** (500 MHz, DMSO-d_6, rotamers in 1:1 ratio, *rotamer 1*): δ 10.77 (s, 1H), 8.37 (s, 1H), 8.05 (s, 1H), 7.62-7.47 (2H, m, Ar-H + H-6), 7.30 (1H, dd, *J* = 8.1, 5.0 Hz, Ar-H), 7.13 (1H, s, Ar-H), 7.09 (1H, s, Ar-H), 7.04-6.99 (1H, m, Ar-H), 6.94 (1H, *app.* td, *J* = 7.2, 3.0 Hz, Ar-H),

6.40 (1H, d, J = 8.6 Hz), 6.22 (1H, s), 5.70 (1H, d, J = 3.6 Hz, H-1'), 5.60-5.56 (1H, m, H-5), 4.59-4.52 (1H, m, DABA1-α-CH), 4.40-4.16 (3H, m, H-2' + H-4' + Trp4-α-CH), 4.04-3.89 (2H, m, DABA1-β-CH + Ile3-α-CH), 3.67 (1H, d, J = 16.2 Hz, Gly2-α-CH_2), 3.62-3.54 (1H, m, Gly2-α-CH_2), 3.41 (1H, J = 14.5 Hz), 3.28-3.04 (3H, m, H-5' + Trp4-β-CH_2), 3.00 (1H, dd, J = 14.5, 6.3 Hz, Trp4-β-CH_2), 2.70 (3H, s, NCH_3), 2.20-2.07 (1H, m, H-3'), 1.80-1.56 (2H, m, H-3' + Ile3-β-CH), 1.47-1.34 (1H, m, Ile3-γ-CH_2), 1.11 (3H, d, J = 6.5 Hz, DABA1-γ-CH_3), 1.08-0.98 (1H, m, Ile3-γ-CH_2), 0.78 (6H, m, 2× CH_3). **^{13}C NMR** (125 MHz, DMSO-d_6, rotamers in 1:1 ratio, *rotamer 1*, one ^{13}C signal is obscure): δ 174.8 (C=O), 172.2 (C=O), 169.8 (C=O), 169.3 (C=O), 163.2 (C=O), 150.6 (C=O), 141.1, 136.0, 127.5, 123.4, 120.6, 118.4, 117.8, 111.5, 110.3, 101.5, 91.9, 78.2, 72.7, 57.4, 55.3, 54.5, 52.3, 43.2, 40.4, 39.6, 36.8, 35.2, 27.7, 24.2, 14.9, 14.5, 10.9. **^{1}H NMR** (500 MHz, DMSO-d_6, rotamers in 1:1 ratio, *rotamer 2*): δ 8.52 (s, 1H), 8.18 (d, J = 9.0 Hz, 1H), 5.65 (1H, d, J = 3.6 Hz, H-1'), 4.82-4.74 (1H, m, DABA1-β-CH), 4.51-4.44 (1H, m, DABA1-α-CH), 2.72 (3H, s, NCH_3). **^{13}C NMR** (125 MHz, DMSO-d_6, rotamers in 1:1 ratio, *rotamer 2*): δ 50.0. **LRMS** [M+H$^+$] 742.4. **HRMS (ESI m/z)** [M+H$^+$] calcd. for $C_{34}H_{48}N_9O_{10}$ 742.3518, found 742.3516.

(2*S*,6*S*,9*S*,10*S*)-2-((1*H*-indol-3-yl)methyl)-13-amino-9-((((2*R*,4*R*,5*R*)-5-(2,4-dioxo-3,4-dihydropyrimidin-1(2*H*)-yl)-4-hydroxytetrahydrofuran-2-yl)methyl)carbamoyl)-6-isopropyl-10,11-dimethyl-4,7,12-trioxo-3,5,8,11-tetraazatridecan-1-oic acid (22)

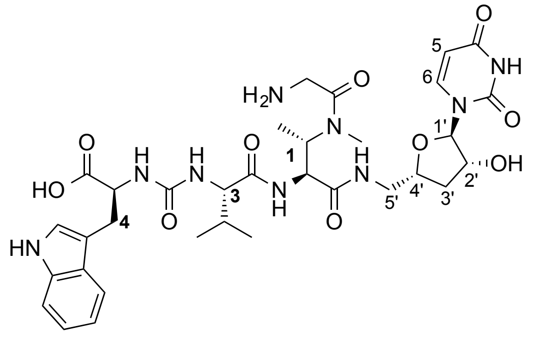

Depsipeptide **S37** (17.8 mg, 23.4 μmol.) was reacted with amine **18** (10.5 mg, 46 μmol) in CH_2Cl_2: DMF (1:1 v/v, 240 μL) in the presence of HOAt (15.7 mg, 115 μmol), EDC.HCl (5.8 mg, 30 μmol) and NMM (3.3 μL, 30 μmol) for 4 h at rt according to general procedure 8 condition B to obtain the fully protected dihydrosansanmycin analogue. This compound was then treated with a mixture of

TFA and *i*-Pr_3SiH in CH_2Cl_2 (1:1 v/v TFA: CH_2Cl_2, 2.5 vol.% *i*-Pr_3SiH, 1.2 mL) for 16 h according to general procedure 8 to afford dihydrosansanmycin **22** as a 9:1 mixture of diastereomers which were inseparable* by reverse-phase HPLC purification (0 to 50% MeCN over 40 min, 0.1 vol.% formic acid) which yielded **22** as a fluffy white solid (8.5 mg, 62%, yield calculated based on both diastereomers as formate salts after HPLC purification). * The inseparable 9:1 mixture of diastereomers was thus submitted for characterisation and biological assays.

IR (ATR): 3316, 2925, 2872, 1672, 1558 cm^{-1}. **^{1}H NMR** (500 MHz, DMSO-d_6, rotamers in 1.4:1 ratio, *rotamer 1*): δ 7.52 (2H, m, H-6 + Ar-H), 7.30 (1H, m, Ar-H), 7.09 (1H, m, Ar-H), 7.02 (1H, m, Ar-H), 6.92 (1H, m, Ar-H), 5.64 (1H, m, H-1'), 5.59 (1H, m, H-5), 4.57 (1H, m, DABA1-α-CH), 4.30 (2H, m, H-2' + H-4' + Val3-α-CH + Trp4-α-CH), 3.98 (1H, m, DABA1-β-CH), 3.89 (2H, m, Gly2-α-CH_2), 3.22-3.00 (4H, m, Trp4-β-CH_2 + 2x H-5'), 2.73 (3H, s, NCH_3), 2.14–2.11 (1H, m, H-3'), 1.63-1.60 (1H, m, H-3'), 1.11 (3H, m, DABA1-γ-CH_3), 1.04 (1H, m, Val3-β-CH), 0.79 (6H, m, 2 x Val-γ-CH_3). **^{13}C NMR** (126 MHz, DMSO-d_6, rotamers in 1.4:1 ratio, *rotamer 1*): δ 173.7 (C=O), 172.4 (C=O), 170.1 (C=O), 167.2 (C=O), 163.3 (C=O), 157.9 (C=O), 151.0 (C=O), 141.4, 136.5, 128.0, 123.6, 120.9, 118.9, 111.7, 110.7, 101.4, 91.9, 78.6, 74.1, 58.4, 55.0, 54.6, 52.9, 43.7, 40.0, 35.9, 31.4, 29.1, 27.6, 19.8, 18.1, 15.1, 14.5. **^{1}H NMR** (500 MHz, DMSO-d_6, rotamers in 1.4:1 ratio, *rotamer 2*): δ 5.67 (1H, m, H-1'). **^{13}C NMR** (126 MHz, DMSO-d_6, rotamers in 1.4:1 ratio, *rotamer 2*): δ 92.3. **LRMS** [M+H^+] 728.4. **HRMS (ESI m/z)** [M+H^+] calcd. for $C_{33}H_{45}N_9O_{10}$ 728.3362, found: 728.3359.

(((*S*)-1-(((2*S*,3*S*)-3-(2-amino-*N*-methylacetamido)-1-((((2*R*,4*R*,5*R*)-5-(2,4-dioxo-3,4-dihydropyrimidin-1(2*H*)-yl)-4-hydroxytetrahydrofuran-2-yl)methyl)amino)-1-oxobutan-2-yl)amino)-1-oxoheptan-2-yl)carbamoyl)-*L*-tryptophan (23)

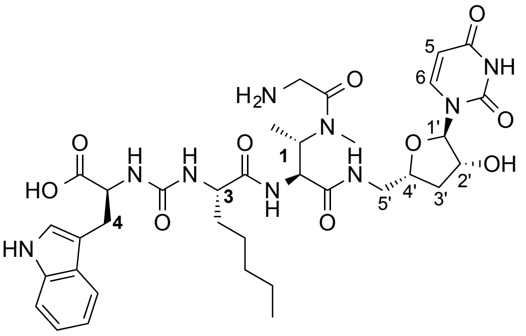

Depsipeptide **S38** (30 mg, 37 μmol) was reacted with amine **18** (20 mg, 92 μmol) in CH_2Cl_2: DMF (1:1 v/v, 370 μL) in the presence of HOAt (25 mg, 185 μmol) and EDC.HCl (9.2 mg, 48 μmol) and NMM (5.0 μL, 48 μmol) for 4 h at rt according to general procedure 8 condition B to obtain the fully protected dihydrosansanmycin analogue. This compound was treated with a mixture of TFA and iPr$_3$SiH in CH_2Cl_2 (1:1 v/v TFA: CH_2Cl_2, 2.5 vol.% iPr$_3$SiH, 3.7 mL) for 4 h according to general procedure 8 to afford dihydrosansanmycin **23** (as a formate salt) after reverse phase HPLC purification (0 to 50% MeCN over 40 min, 10 min at 100% H_2O) as an amorphous white solid (8.1 mg, 27% over 2 steps).

IR (ATR): 3356, 2977, 2932, 1731, 1638 cm^{-1}. **^{1}H NMR** (400 MHz, DMSO-d_6, rotamers in 1:1 ratio, *rotamer 1*): δ 10.79 (1H, s), 8.64 (s, 1H), 8.37 (s, 1H), 8.23 (s, 1H), 8.16 (s, 1H), 7.54 (2H, m, H-6 + Ar-H), 7.33-7.28 (1H, m, Ar-H), 7.13 (1H, s, Ar-H), 6.97-6.91 (1H, m, Ar-H), 6.45 (1H, d, J = 7.8 Hz), 6.15 (1H, s), 5.71 (1H, d, J = 3.3 Hz, H-1'), 5.58 (1H, *app.* dd, J = 8.0, 2.5 Hz, H-5), 4.55 (1H, *app.* t, J = 9.4 Hz, DABA1-α-CH), 4.24-4.17 (3H, m, H-2' + H-4' + Trp4-α-CH), 4.09-4.00 (1H, m, Aha3-α-CH), 3.99-3.88 (1H, m, DABA1-β-CH), 3.32-3.00 (3H, m, H-5' + Trp4-β-CH_2), 3.70-3.61 (1H, m, Gly2-α-CH_2), 3.46 (1H, d, J = 14.9 Hz, Gly2-α-CH_2), 2.99 (1H, dd, J =14.6, 6.5 Hz, Trp4-β-CH_2), 2.72 (3H, s, NCH_3), 2.71 (3H, s, NCH_3), 2.15-2.05 (1H, m, H-3'), 1.71-1.63 (1H, m, H-3'), 1.56-1.37 (2H, m, Aha3-β-CH_2), 1.30-1.14 (6H, m), 1.10 (3H, d, J = 6.5 Hz, DABA1-γ-CH_3), 0.86-0.77 (3H, m). **^{13}C NMR** (100 MHz, DMSO-d_6, rotamers in 1:1 ratio, *rotamer 1*): δ 175.2 (C=O), 173.9 (C=O), 170.1 (C=O), 169.8 (C=O), 168.8 (C=O), 163.3 (C=O), 150.9 (C=O), 141.5, 136.4, 128.0, 124.2, 123.8, 120.7, 117.8, 111.6, 110.6, 101.2, 92.0, 78.4, 73.8, 54.3, 53.4, 50.7, 42.9, 40.2, 34.7, 31.2, 28.6, 28.4, 24.9, 21.8, 15.3, 13.1. **^{1}H NMR** (400 MHz, DMSO-d_6, rotamers in 1:1 ratio, *rotamer 2*): δ 8.06 (s, 1H), 7.06-6.99 (1H, m, Ar-H), 7.10 (1H, *app.* d, J = 2.3 Hz, Ar-H), 5.65 (1H, d, J = 3.4 Hz, H-1'), 4.82-4.73 (1H, m, DABA1-β-CH), 4.49-4.42 (1H, m, DABA1-α-CH), 4.41-4.34 (1H, m, H-4'), 2.15-2.05 (1H, m, H-3'), H-3' minor), 1.77 (1H, *app.* d, J = 13.0 Hz, H-3'). **^{13}C NMR** (100 MHz, DMSO-d_6, rotamers in 1:1 ratio, *rotamer 2*): δ 27.0. **LRMS** [M+H$^+$] 756.4. **HRMS (ESI m/z)** [M+H$^+$] calcd. for $C_{35}H_{50}N_9O_{10}$ 756.3675, found 756.3674.

(((*S*)-2-(((2*S*,3*S*)-3-(2-amino-*N*-methylacetamido)-1-((((2*R*,4*R*,5*R*)-5-(2,4-dioxo-3,4-dihydropyrimidin-1(2*H*)-yl)-4-hydroxytetrahydrofuran-2-yl)methyl)amino)-1-oxobutan-2-yl)amino)-1-cyclohexyl-2-oxoethyl)carbamoyl)-*L*-tryptophan (24)

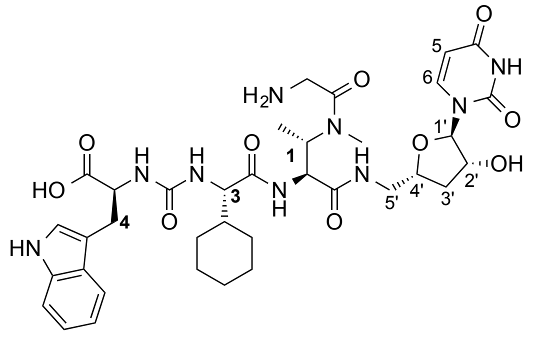

Depsipeptide **S39** (24 mg, 30 μmol) was reacted with amine **18** (14 mg, 60 μmol) in CH_2Cl_2: DMF (1:1 v/v, 300 μL) in the presence of HOAt (20 mg, 150 μmol) and EDC.HCl (7.4 mg, 39 μmol) and NMM (4.0 μL, 39 μmol) for 4 h at rt according to general procedure 8 condition B to obtain the fully protected dihydrosansanmycin analogue. This compound was treated with a mixture of TFA and *i*Pr_3SiH in CH_2Cl_2 (1:1 v/v TFA: CH_2Cl_2, 2.5 vol.% *i*Pr_3SiH, 3.0 mL) for 4 h according to general procedure 8 to afford dihydrosansanmycin **24** (as a TFA salt) after reverse phase HPLC purification (0 to 50% MeCN over 40 min, 10 min at 100% H_2O) as an amorphous white solid (13.7 mg, 60% over 2 steps).

IR (ATR): 3323, 2979, 2932, 1626 cm^{-1}. **^{1}H NMR** (400 MHz, CD_3OD, rotamers in 1.6:1 ratio, *major rotamer*): δ 8.78 (1H, t, *J* = 5.9 Hz), 8.37 (1H, d, *J* = 8.9 Hz), 8.22 (1H, d, *J* = 8.5 Hz), 8.15 (1H, t, *J* = 5.8 Hz), 7.62 (1H, d, *J* = 8.1 Hz, H-6), 7.58-7.54 (1H, m, Ar-H), 7.38-7.26 (1H, m, Ar-H), 7.16-7.04 (2H, m, Ar-H), 7.04-6.95 (1H, m, Ar-H), 5.77-5.70 (2H, m, H-1' + H-5), 4.62-4.56 (2H, m, DABA1-α-CH + Trp4-α-CH), 4.50-4.42 (2H, m, H-2'), 4.39-4.31 (1H, m, H-4'), 4.16 (1H, d, *J* = 16.0 Hz, Gly2-α-CH_2), 4.03-3.88 (3H, m, DABA1-β-CH + Gly2-α-CH_2 + Chg3-α-CH), 3.54-3.46 (1H, m, H-5'), 3.29-3.13 (3H, m, H-5' + Trp4-β-CH_2), 2.87 (3H, s, NCH_3), 2.78 (3H, s, NCH_3), 2.21 (1H, ddd, *J* = 13.6, 7.6, 6.1 Hz, H-3'), 1.84-1.48 (6H, m, H-3' + 2.5× CH_2 + Chg3-β-CH), 1.25-0.95 (8H, m, DABA1-γ-CH_3 + 2.5× CH_2). **^{13}C NMR** (100 MHz, CD_3OD, rotamers in 1.6:1 ratio, *major rotamer*): δ 176.2 (C=O), 175.0 (C=O), 171.3 (C=O), 167.6 (C=O), 167.2 (C=O), 160.0 (C=O), 152.2 (C=O), 142.4, 137.9, 128.8, 124.2, 122.1, 119.6, 119.4, 111.8, 110.4, 102.0, 94.7, 81.2, 75.8, 60.0,

56.6, 54.6, 54.1, 45.0, 41.5, 40.8, 35.6, 30.6, 29.6, 29.0, 27.8, 26.8, 14.9. **LRMS** [M+H$^+$] 768.3. **HRMS (ESI m/z)** [*M*+H$^+$] calcd. for $C_{36}H_{50}N_9O_{10}$ 768.3875, found 768.3867.

(((*S*)-1-(((2*S*,3*S*)-3-(2-amino-*N*-methylacetamido)-1-((((2*R*,4*R*,5*R*)-5-(2,4-dioxo-3,4-dihydropyrimidin-1(2*H*)-yl)-4-hydroxytetrahydrofuran-2-yl)methyl)amino)-1-oxobutan-2-yl)amino)-3-cyclohexyl-1-oxopropan-2-yl)carbamoyl)-*L*-tryptophan (25)

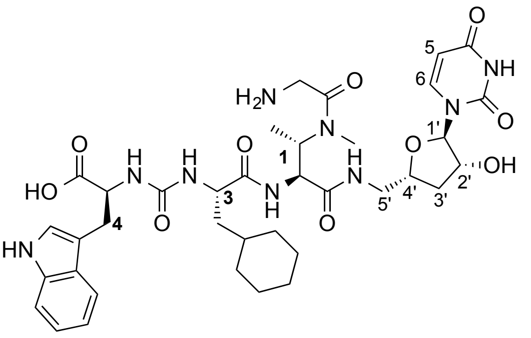

Depsipeptide **S40** (17 mg, 20 μmol) was reacted with amine **18** (9.5 mg, 42 μmol) in CH_2Cl_2: DMF (1:1 v/v, 200 μL) in the presence of HOAt (14 mg, 103 μmol) and EDC.HCl (5.1 mg, 27 μmol) and NMM (3.0 μL, 27 μmol) for 4 h at rt according to general procedure 8 condition B to obtain the fully protected dihydrosansanmycin analogue. This compound was treated with a mixture of TFA and *i*Pr_3SiH in CH_2Cl_2 (1:1 v/v TFA: CH_2Cl_2, 2.5 vol.% *i*Pr_3SiH, 4 mL) for 4 h according to general procedure 8 to afford dihydrosansanmycin **25** (as a formate salt) after reverse phase HPLC purification (0 to 50% MeCN over 45 min, 10 min at 100% H_2O) as an amorphous white solid (6.0 mg, 35% over 2 steps).

IR (ATR): 3368, 2926, 1647, 1549 cm^{-1}. **^{1}H NMR** (600 MHz, CD_3OD, rotamers in 1.5:1 ratio, *major rotamer*): δ 7.62 (1H, d, *J* = 8.1 Hz, H-6), 7.60-7.54 (1H, m, Ar-H), 7.33 (1H, dd, *J* = 9.1, 8.2 Hz, Ar-H), 7.11 (1H, s, Ar-H), 7.10-7.05 (1H, m, Ar-H), 7.04-6.98 (1H, m, Ar-H), 5.73 (1H, d, *J* = 2.6 Hz, H-1'), 5.71 (1H, d, *J* = 8.0 Hz, H-5), 4.65 (1H, d, *J* = 7.6 Hz, DABA1-α-CH), 4.60-4.52 (2H, m, H-4' + Trp4-α-CH), 4.49-4.44 (1H, m, H-2'), 4.21-4.12 (2H, m, Gly2-CH_2 + Cha3-α-CH), 3.99 (1H, dq, *J* = 10.1, 6.6 Hz, DABA1-β-CH), 3.91 (1H, d, *J* = 16.0 Hz, Gly2-CH_2), 3.55-3.49 (1H, dd, m, H-5'), 3.29-3.16 (2H, m, Trp4-β-CH_2), 2.88 (3H, s, NCH_3), 2.22 (1H, ddd, *J* = 13.8, 7.7, 6.2 Hz, H-3'), 1.84-1.60 (5H, m, 2× Cha3-CH_2 + H-3'), 1.49-1.41 (2H, m, Cha3-β-CH_2), 1.36-1.31 (1H, m, Cha3-γ-CH), 1.26-1.09 (7H, m, 2× Cha3-

CH_2 + DABA1-γ-CH_3), 1.03-0.79 (2H, m, Cha3-CH_2). **^{13}C NMR** (150 MHz, CD_3OD, rotamers in 1.5:1 ratio, *major rotamer*): δ 176.3 (C=O), 175.9 (C=O), 171.9 (C=O), 167.4 (C=O), 167.1 (C=O), 160.2 (C=O), 152.5 (C=O), 142.6, 138.1, 129.1, 124.5, 122.0, 119.3, 119.0, 112.0, 110.4, 102.3, 95.1, 80.6, 76.2, 56.6, 54.8, 54.4, 53.0, 44.8, 40.9, 40.6, 36.2, 34.9, 34.4, 28.5, 28.4, 27.5, 26.8, 13.9. **LRMS** [M+H$^+$] 782.4. **HRMS (ESI m/z)** [M+H$^+$] calcd. for $C_{37}H_{52}N_9O_{10}$ 782.3831, found 782.3824.

(((2*S*,3*S*)-1-(((2*S*,3*S*)-3-(2-amino-*N*-methylacetamido)-1-((((2*R*,4*R*,5*R*)-5-(2,4-dioxo-3,4-dihydropyrimidin-1(2*H*)-yl)-4-hydroxytetrahydrofuran-2-yl)methyl)amino)-1-oxobutan-2-yl)amino)-3-hydroxy-1-oxobutan-2-yl)carbamoyl)-*L*-tryptophan (26)

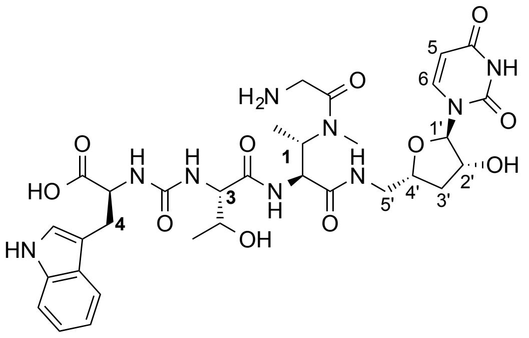

Depsipeptide **S41** (18.0 mg, 23.2 μmol.) was reacted with amine **18** (10.5 mg, 46.4 μmol) in CH_2Cl_2: DMF (1:1 v/v, 240 μL) in the presence of HOAt (15.8 mg, 116.0 μmol), EDC.HCl (5.8 mg, 30.1 μmol) and NMM (3.3 μL, 30.1 μmol) for 4 h at rt according to general procedure 8 to obtain the fully protected dihydrosansanmycin analogue. This compound was then treated with a mixture of TFA and *i*-Pr_3SiH in CH_2Cl_2 (1:1 v/v TFA: CH_2Cl_2, 2.5 vol.% *i*-Pr_3SiH, 1.2 mL) for 16 h according to general procedure 8 to afford dihydrosansanmycin **26** (as a formate salt) after reverse phase HPLC purification (0 to 30% MeCN over 40 min) as a fluffy white solid (8.3 mg, 46% over two steps).

IR (ATR): 3293, 2922, 1677, 1594, 1436 cm^{-1}. **^{1}H NMR** (500 MHz, DMSO-d_6, rotamers in 1.3:1 ratio, *rotamer 1*): δ 7.53 (2H, m, H-6 + Ar-H), 7.30 (1H, m, Ar-H), 7.12 (1H, m, Ar-H), 7.03 (1H, m, Ar-H), 6.94 (1H, m, Ar-H), 6.55 (1H, br s, Thr3-γ-OH), 5.64 (1H, d, J = 3.1 Hz, H-1’), 5.57 (1H, d, J = 6.4 Hz, H-5), 4.60 (1H, m, DABA1-α-CH), 4.30 (1H, m, Trp4-α-CH), 4.27–4.23 (2H, m, H-2’ + H-4’), 4.00–

3.94 (3H, m, Thr3-α-CH + Thr3-β-CH + DABA1-β-CH), 3.36–3.00 (6H, m, Gly2-α-CH_2 + 2x H-5' + Trp4-β-CH_2), 2.49 (3H, s, NCH_3), 2.14–2.11 (1H, m, H-3'), 1.71–1.62 (1H, m, H-3'), 1.15 (3H, d, J = 4.7 Hz,Thr3-γ-CH_3), 0.99 (3H, d, J = 5.5 Hz, DABA1-γ-CH_3). **^{13}C NMR** (126 MHz, DMSO-d_6, rotamers in 1.3:1 ratio, *rotamer 1*): δ 179.3 (C=O), 173.7 (C=O), 171.8 (C=O), 170.0 (C=O), 164.5 (C=O), 163.6 (C=O), 150.8 (C=O), 141.7, 136.4, 127.9, 124.3, 121.2, 119.2, 118.5, 111.7, 110.0, 102.1, 92.6, 79.0, 78.9, 74.1, 66.9, 59.4, 55.3, 55.2, 44.1, 40.3, 35.8, 29.2, 27.6, 20.1, 15.1. **^{1}H NMR** (500 MHz, DMSO-d_6; rotamers in 1.3:1 ration, *rotamer 1*): δ 5.68 (1H, m, H-1'), 5.58 (1H, m, H-5). **LRMS** [M+H$^+$] 730.4. **HRMS (ESI m/z)** [M+H$^+$] calcd. for $C_{32}H_{44}N_9O_{11}$ 730.3154, found 730.3150.

(2*S*,6*S*,9*S*,10*S*)-2-((1*H*-indol-3-yl)methyl)-13-amino-6-(carboxymethyl)-9-((((2*R*,4*R*,5*R*)-5-(2,4-dioxo-3,4-dihydropyrimidin-1(2*H*)-yl)-4-hydroxytetrahydrofuran-2-yl)methyl)carbamoyl)-10,11-dimethyl-4,7,12-trioxo-3,5,8,11-tetraazatridecanoic acid (27)

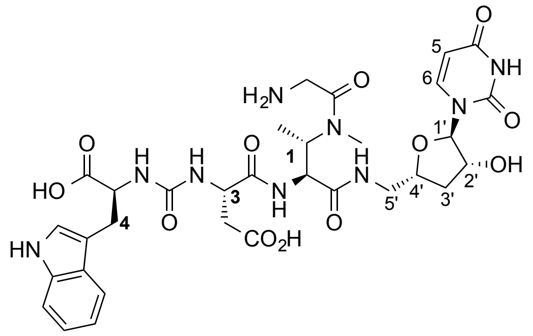

Depsipeptide **S42** (19.2 mg, 21.6 μmol) was reacted with amine **18** (12.2 mg, 53.9 μmol) in CH_2Cl_2: DMF (1:1 v/v, 220 μL) in the presence of HOAt (14.7 mg, 107.8 μmol), EDC·HCl (5.4 mg, 28.0 μmol) and NMM (3.1 μL, 28.0 μmol) for 4 h according to general procedure 8 condition B to obtain the fully protected dihydrosansanmycin analogue. This compound was then treated with a mixture of TFA and *i*-Pr_3SiH in CH_2Cl_2 (1:1 v/v TFA: CH_2Cl_2, 2.5 vol.% *i*-Pr_3SiH, 1.2 mL) for 16 h according to general procedure 8 to afford dihydrosansanmycin **27** (as a formate salt) after reverse phase HPLC (0 to 50% MeCN over 40 min) as a white solid (10.0 mg, 62% over two steps).

IR (ATR): 3304, 2952, 2844, 1643, 1450, 1411 cm^{-1}. **^{1}H NMR** (500 MHz, DMSO-d_6, rotamers in 1.1:1 ratio, *rotamer 1*): *δ* 7.52 (2H, m, H-6 + Ar-H), 7.31 (1H, m, Ar-H), 7.12 (1H, s, Ar-H), 6.99 (1H, m, Ar-H), 6.93 (1H, m, Ar-H), 5.65 (1H, m, H-5), 5.57 (1H, m, H-1'), 4.41 (1H, m, Asp3-α-CH), 4.33-4.28 (4H, m, H-2' + H-4' +

DABA1-α-CH + Trp4-αCH), 3.94–3.55 (2H, m, Gly2-α-CH_2), 3.27 (1H, m, DABA1-β-CH), 3.12 (1H, m, H-5'), 2.99 (1H, m, H-5'), 2.71 (3H, m, NMe), 2.44 (4H, m, Asp3-β-CH_2 + Trp4-β-CH_2), 2.15–2.11 (1H, m, H-3'), 1.68–1.65 (1H, m, H-3'), 1.08 (3H, m, DABA1-γ-CH_3). **^{1}H NMR** (500 MHz, DMSO-d_6, rotamers in 1.1:1 ratio, *rotamer 2*): *δ* 5.59 (1H, m, H-1'), 5.66 (1H, m, H-5), 2.65 (3H, m, NMe), 1.75 - 1.68 (1H, m, H-3'). **^{13}C NMR:** (126 MHz, DMSO-d_6)*:* δ 179.5 (C=O), 172.1 (C=O), 167.8 (C=O), 166.8 (C=O), 166.1 (C=O), 163.8 (C=O), 163.7 (C=O), 150.9 (C=O), 141.5, 136.3, 128.0, 124.0, 123.9, 121.0, 118.9, 111.6, 110.7, 110.9, 101.9, 92.5, 78.8, 74.4, 55.6, 55.0, 51.2, 43.9, 40.2, 40.0, 35.9, 29.4, 28.5, 27.8, 15.0. *Rotamer 2:* not distinguishable. **LRMS** [M+H$^+$] 744.4. **HRMS (ESI m/z)** [M+H]$^+$ calcd. for $C_{32}H_{42}N_9O_{12}$ 744.2947, found 744.2943.

(((*S*)-6-amino-1-(((2*S*,3*S*)-3-(2-amino-*N*-methylacetamido)-1-((((2*R*,4*R*,5*R*)-5-(2,4-dioxo-3,4-dihydropyrimidin-1(2*H*)-yl)-4-hydroxytetrahydrofuran-2-yl)methyl)amino)-1-oxobutan-2-yl)amino)-1-oxohexan-2-yl)carbamoyl)-*L*-tryptophan (28)

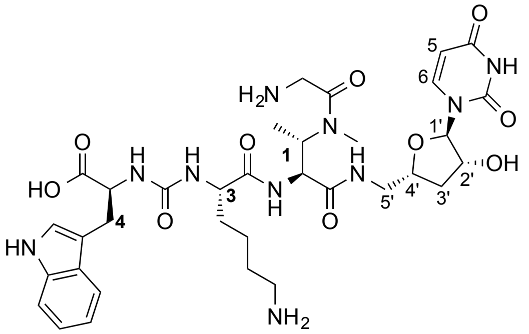

Depsipeptide **S43** (13.4 mg, 14.8 μmol.) was reacted with amine **18** (11 mg, 48.7 μmol) in CH_2Cl_2: DMF (1:1 v/v, 180 μL) in the presence of HOAt (10 mg, 74.0 μmol), EDC.HCl (3.7 mg, 19.2 μmol) and NMM (2.1 μL, 19.2 μmol) for 4 h according to general procedure 8 condition B to obtain the corresponding protected dihydrosansanmycin analogue. This compound was then treated with a mixture of TFA and *i*-Pr_3SiH in CH_2Cl_2 (1:1 v/v TFA: CH_2Cl_2, 2.5 vol.% *i*-Pr_3SiH, 1.2 mL) for 16 h according to general procedure 8 to afford dihydrosansanmycin **28** (as a TFA salt) after reverse phase HPLC (0 to 30% MeCN over 40 min) as a fluffy white solid (7.2 mg, 62% over two steps).

IR (ATR): 3285, 3083, 2941, 1672, 1555 cm^{-1}. **^{1}H NMR** (500 MHz, DMSO-d_6, rotamers in 2.2:1 ratio, *major rotamer*): *δ* 7.55-7.49 (2H, m, H-6 + Ar-H), 7.32 (1H,

d, J = 8.0 Hz, Ar-H), 7.11 (1H, s, Ar-H), 7.04 (1H, m, Ar-H), 6.95 (1H, m, Ar-H), 5.68–5.57 (2H, m, H-5 + H-1'), 4.79–4.73 (1H, m, Lys3-α-CH), 4.36-4.32 (3H, m, H-2' + DABA1-α-CH + H-4'), 4.11–4.09 (1H, m, Trp4-α-CH), 3.89–3.86 (1H, m, DABA1-β-CH), 3.86–3.65 (2H, m, Gly2-α-CH_2), 3.25–3.24 (1H, m, H-5'), 3.19–2.98 (3H, m, H-5'+ 2 x Trp4-β-CH_2), 2.74 (5H, m, NMe + Lys3-ε-CH_2), 2.21–2.08 (1H, m, H-3'), 1.51-1.48 (3H, m, H-3'+ Lys3-δ-CH_2), 1.27–1.24 (2H, m, Lys3-γ-CH_2), 1.11 (3H, d, J = 6.4 Hz, DABA1-γ-CH_3), 1.02 (2H, d, J = 6.7 Hz, Lys3-β-CH_2). **^{13}C NMR** (126 MHz, DMSO-d_6, rotamers in 2.2:1 ratio, *major rotamer*): δ 173.7 (C=O), 172.0 (C=O), 169.8 (C=O), 169.0 (C=O), 165.6 (C=O), 162.8 (C=O), 150.0 (C=O), 141.7, 135.6, 126.9, 124.0, 121.2, 118.7, 111.7, 109.0, 79.0, 74.3, 55.4, 55.1, 54.8, 53.7, 53.1, 44.0, 40.1, 39.1, 36.0, 32.2, 29.0, 28.1, 27.7, 27.0, 22.7, 15.0, 14.3. **LRMS** [M+H$^+$] 757.4. **HRMS (ESI m/z)** [M+H$^+$] calcd. for $C_{34}H_{49}N_{10}O_{10}$ 757.3627, found 757.3620.

(2*S*,6*S*,9*S*,10*S*)-2-((1*H*-indol-3-yl)methyl)-13-amino-6-benzyl-9-((((2*R*,4*R*,5*R*)-5-(2,4-dioxo-3,4-dihydropyrimidin-1(2*H*)-yl)-4-hydroxytetrahydrofuran-2-yl)methyl) carbamoyl)-10,11-dimethyl-4,7,12-trioxo-3,5,8,11-tetraazatridecan-1-oic acid (29)

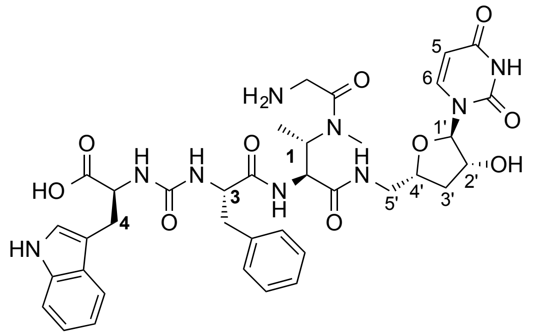

Depsipeptide **S44** (16.7 mg, 19.3 μmol) was reacted with amine **18** (10.3 mg, 48.1 μmol) in CH_2Cl_2: DMF (1:1 v/v, 220 μL) in the presence of HOAt (13.1 mg, 96.3 μmol), EDC·HCl (4.8 mg, 25.0 μmol) and NMM (2.7 μL, 25.0 μmol) for 4 h according to general procedure 8 to obtain the fully protected dihydrosansanmycin analogue. This compound was then treated with a mixture of TFA and *i*-Pr_3SiH in CH_2Cl_2 (1:1 v/v TFA: CH_2Cl_2, 2.5 vol.% *i*-Pr_3SiH, 1.2 mL) for 16 h according to general procedure 8 to afford dihydrosansanmycin **29** (as a TFA salt) after reverse phase HPLC (0 to 50% MeCN over 40 min as a fluffy white solid (8.7 mg, 59% over two steps).

IR (ATR): 3304, 2977, 2742, 1435, 1080 cm^{-1}. **^{1}H NMR** (500 MHz, CD_3OD, rotamers in 1.2:1 ratio, *rotamer 1*): δ 7.61 (1H, m, H-6), 7.56 (1H, m, Ar-H), 7.32 (1H, m, Ar-H), 7.23-7.14 (5H, m, 5x Ar-H), 7.10-7.05 (2H, m, 2x Ar-H), 7.01-6.98 (1H, m, Ar-H), 5.73 (1H, m, H-5), 5.70 (1H, m, H-1'), 4.57-4.54 (3H, m, H-2' + DABA1-α-CH + Trp4-αCH), 4.47-4.45 (1H, m, Phe3-α-CH), 4.35-4.32 (1H, m, H-4'), 3.92-3.86 (1H, m, DABA1-β-CH), 3.86-3.65 (2H, m, Gly2-α-CH_2), 3.49 (1H, m, H-5'), 3.38-3.24 (5H, m, H-5'+ 2x Phe3-β-CH_2 + 2 x Trp4-β-CH_2), 2.86 (3H, s, NCH_3), 2.25-2.19 (1H, m, H-3'), 1.75-1.70 (1H, m, H-3'), 1.19 (3H, d, J = 6.7 Hz, DABA1-γ-CH_3. **^{13}C NMR:** (126 MHz, CD_3OD, rotamers in 1.2:1 ratio, *rotamer 1*): δ 174.8 (C=O), 172.7 (C=O), 169.9 (C=O), 166.3 (C=O), 165.6 (C=O), 164.9 (C=O), 150.9 (C=O), 141.1, 136.7, 128.8, 127.9, 127.3, 126.3, 126.2, 123.0, 120.4, 118.4, 118.0, 110.7, 109.0, 101.1, 94.0, 79.8, 79.2, 74.8, 55.4, 55.2, 54.9, 53.6, 47.7, 43.8, 43.2, 37.8, 29.0, 27.5, 26.5, 13.3. **^{1}H NMR** (500 MHz, CD_3OD, rotamers in 1.2:1 ratio, *rotamer 2*): δ 2.33-2.29 (1H, m, H-3'). **^{13}C NMR:** (126 MHz, CD_3OD, rotamers in 1.2:1 ratio, *rotamer 2*): δ 34.8. **LRMS** [M^+H^+] 776.4. **HRMS (ESI m/z)** [$M+H^+$] calcd. for $C_{37}H_{46}N_9O_{10}$ 776.3362, found 776.3358.

(((*S*)-1-(((2*S*,3*S*)-3-(2-amino-*N*-methylacetamido)-1-((((2*R*,4*R*,5*R*)-5-(2,4-dioxo-3,4-dihydropyrimidin-1(2*H*)-yl)-4-hydroxytetrahydrofuran-2-yl)methyl)amino)-1-oxobutan-2-yl)amino)-1-oxo-3-(4-(trifluoromethyl)phenyl)propan-2-yl)carbamoyl)-*L*-tryptophan (30)

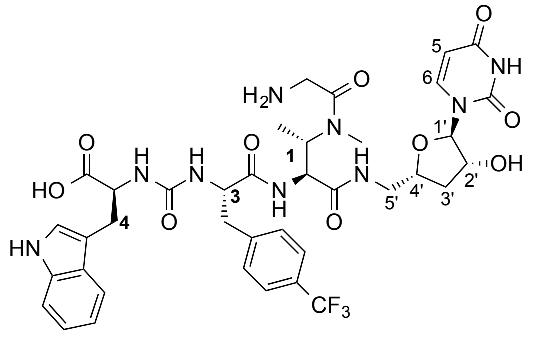

Depsipeptide **S45** (28 mg, 32 μmol) was reacted with amine **18** (15 mg, 64 μmol) in CH_2Cl_2: DMF (1:1 v/v, 320 μL) in the presence of HOAt (22 mg, 161 μmol) and EDC.HCl (8.0 mg, 42 μmol) and NMM (4.4 μL, 42 μmol) for 4 h at rt according to general procedure 8 condition B to obtain the fully protected dihydrosansanmycin analogue. This compound was treated with a mixture of TFA and iPr$_3$SiH in CH_2Cl_2 (1:1 v/v TFA: CH_2Cl_2, 2.5 vol.% iPr$_3$SiH, 3.2 mL) for 4 h according to general

procedure 8 to afford dihydrosansanmycin **30** (as a formate salt) after reverse phase HPLC purification (0 to 50% MeCN over 45 min, 10 min at 100% H_2O) as an amorphous white solid (5.6 mg, 20% over 2 steps).

IR (ATR): 3331, 2948, 2837, 1668 cm^{-1}. **^{1}H NMR** (500 MHz, DMSO-d_6, rotamers in 1:1 ratio, *rotamer 1*): δ 10.73 (1H, s), 8.64 (1H, s), 8.45-8.29 (1H, m), 8.21 (1H, s), 7.66-7.42 (4H, H-6 + Ar-H), 7.37-7.29 (2H, m, Ar-H), 7.30-7.16 (1H, m, Ar-H), 7.05 (1H, s, Ar-H), 6.97 (1H, *app.* t, *J* = 7.5 Hz, Ar-H), 6.88 (1H, *app.* t, *J* = 7.5 Hz, Ar-H), 6.52 (1H, s), 6.22 (1H, s), 5.76-5.59 (1H, m, H-1'), 5.55 (1H, d, *J* = 8.0, H-5), 4.54 (1H, *app.* t, *J* = 9.1 Hz, DABA1-α-CH), 4.43-4.20 (3H, m, H-2' + H-4' + *p*-CF_3-Pal3-α-CH), 4.19-4.08 (1H, m, Trp4-α-CH), 3.94-3.82 (1H, m, DABA1-β-CH), 3.79-3.86 (1H, m, Gly2-α-CH_2), 3.60-3.34 (1H, m, Gly2-α-CH_2), 3.61-3.39 (m, 1H), 3.34-2.89 (5H, m, H-5' + *p*-CF_3-Pal3-β-CH_2 + Trp4-β-CH_2), 2.88-2.78 (1H, m, *p*-CF_3-Pal3-β-CH_2), 2.70 (3H, s, NCH_3), 2.19-2.04 (1H, m, H-3'), 1.73-1.53 (1H, m, H-3'), 1.03 (3H, d, *J* = 6.0 Hz, DABA1-γ-CH_3). **^{13}C NMR** (125 MHz, DMSO-d_6, rotamers in 1:1 ratio, *rotamer 1*): δ 175.1 (C=O), 173.0 (C=O), 170.9 (C=O), 169.0 (C=O), 167.0 (C=O), 163.1 (C=O), 150.4 (C=O), 142.1, 140.6, 135.3, 129.4, 127.4, 126.6, 123.9, 123.6, 119.7, 118.0, 117.4, 117.1, 110.4, 110.3, 100.9, 91.3, 78.6, 73.4, 54.4, 54.1, 53.5, 52.1, 42.8, 39.3, 36.8, 34.6, 27.2, 26.5, 13.8. **^{1}H NMR** (500 MHz, DMSO-d_6, rotamers in 1:1 ratio, *rotamer 2*): δ 4.77-4.70 (1H, m, DABA1-β-CH), 4.46 (1H, *app.* t, *J* = 8.7 Hz, DABA1-α-CH), 2.68 (3H, s, NCH_3). **^{13}C NMR** (125 MHz, DMSO-d_6, rotamers in 1:1 ratio, *rotamer 2*): δ 53.5, 49.6, 28.0, 13.2. **LRMS** [M+H^+] 844.3. **HRMS (ESI m/z)** [M+H^+] calcd. for $C_{38}H_{45}F_3N_9O_{10}$ 844.3260, found 844.3234.

(2*S*,6*S*,9*S*,10*S*)-2-((1*H*-indol-3-yl)methyl)-13-amino-9-((((2*R*,4*R*,5*R*)-5-(2,4-dioxo-3,4-dihydropyrimidin-1(2*H*)-yl)-4-hydroxytetrahydrofuran-2-yl)methyl)carbamoyl)-6-(4-hydroxybenzyl)-10,11-dimethyl-4,7,12-trioxo-3,5,8,11-tetraazatridecan-1-oic acid (31)

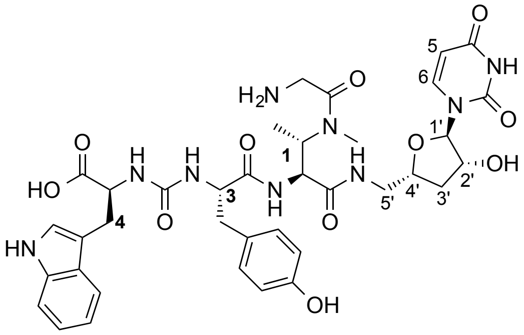

Depsipeptide **S46** (29.5 mg, 33.0 μmol.) was reacted with amine **18** (18.7 mg, 82.4 μmol) in CH_2Cl_2: DMF (1:1 v/v, 340 μL) in the presence of HOAt (22.5 mg, 165.0 μmol), EDC·HCl (8.2 mg, 42.9 μmol) and NMM (4.7 μL, 42.9 μmol) for 4 h according to general procedure 8 to obtain the fully protected dihydrosansanmycin analogue. This compound was then treated with a mixture of TFA and *i*-Pr_3SiH in CH_2Cl_2 (1:1 v/v TFA: CH_2Cl_2, 2.5 vol.% *i*-Pr_3SiH, 1.2 mL) for 16 h according to general procedure 8 to afford dihydrosansanmycin **31 (**as a formate salt) after reverse-phase HPLC purification (0 to 50% MeCN over 40 min) as a fluffy white solid (15.7 mg, 50%, yield over two steps).

IR (ATR): 3325, 2928, 1676, 1541, 1498 cm^{-1}. **^{1}H NMR** (400 MHz, DMSO-d_6, rotamers in 1.5:1 ratio, *major rotamer*): δ 7.53 (2H, m, H-6 + Ar-H), 7.30 (1H, d, *J* = 8.0 Hz Ar-H), 7.10 (1H, app. dd, *J* = 6.2 Hz, Ar-H), 7.03 (1H, m, Ar-H), 6.95 (3H, m, 3x Ar-H), 6.61 (2H, m, 2x Ar-H), 5.67 (1H, d, *J* = 3.2 Hz, H-1'), 5.59 (1H, d, *J* = 2.3 Hz, H-5), 4.55 (1H, m, DABA1-α-CH), 4.32-4.24 (4H, m, Trp4-α-CH + Tyr3-α-CH, H-2' + H-4'), 3.91 (1H, m, DABA1-β-CH), 3.33–2.96 (6H, m, Trp4-β-CH_2 + Tyr3-β-CH_2 + 2x H-5'), 2.82-2.60 (5H, m, Gly2-α-CH_2 + NCH_3), 2.14–2.11 (1H, m, H-3'), 1.70-1.62 (1H, m, H-3'), 1.10 (3H, d, *J* 6.5 Hz, DABA1-γ-CH_3). **^{13}C NMR** (101 MHz, DMSO-d_6, rotamers in 1.5:1 ratio, *major rotamer*): δ 175.0 (C=O), 172.3 (C=O), 169.1 (C=O), 168.3 (C=O), 163.6 (C=O), 159.9, 156.2 (C=O), 156.0 (C=O), 141.3, 135.6, 130.4, 127.3, 127.1, 124.0, 121.1, 118.8, 118.5, 116.2, 115.2, 109.9, 108.8, 92.3, 79.0, 74.4, 55.3, 54.2, 50.8, 43.9, 40.6, 35.8, 29.3, 28.3, 27.6, 15.0. **LRMS** [M+H$^+$] 792.5. **HRMS (ESI m/z)** [M+H$^+$] calcd. for $C_{37}H_{46}N_9O_{11}$ 792.3311, found 792.3304.

(((*S*)-1-(((2*S*,3*S*)-3-(2-amino-*N*-methylacetamido)-1-((((2*R*,4*R*,5*R*)-5-(2,4-dioxo-3,4-dihydropyrimidin-1(2*H*)-yl)-4-hydroxytetrahydrofuran-2-yl)methyl)amino)-1-oxobutan-2-yl)amino)-1-oxo-3-(pyridin-3-yl)propan-2-yl)carbamoyl)-*L*-tryptophan (32)

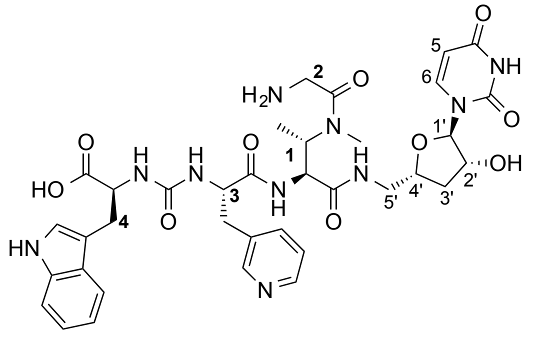

Depsipeptide **S47** (14 mg, 17 μmol) was reacted with amine **18** (10 mg, 44 μmol) in CH_2Cl_2: DMF (1:1 v/v, 170 μL) in the presence of HOAt (12 mg, 86 μmol) and EDC.HCl (4.1 mg, 22 μmol) and NMM (2.3 μL, 22 μmol) for 3.5 h at rt according to general procedure 8 condition B to obtain the fully protected dihydrosansanmycin analogue. This compound was treated with a mixture of TFA and iPr$_3$SiH in CH_2Cl_2 (1:1 v/v TFA: CH_2Cl_2, 2.5 vol.% iPr$_3$SiH, 1.7 mL) for 4 h according to general procedure 8 to afford dihydrosansanmycin **32** (as a TFA salt) after reverse phase HPLC purification (0 to 50% MeCN over 40 min, 10 min at 100% H_2O) as an amorphous white solid (8.2 mg, 54% over 2 steps).

IR (ATR): 3367, 3063, 1666 cm^{-1}. ^{1}**H NMR** (400 MHz, CD_3OD, rotamers in 1.4:1 ratio, *rotamer* 1): δ 8.74 (1H, t, *J* = 6.0 Hz), 8.60-8.54 (1H, m, Ar-H), 8.41 (1H, d, *J* = 8.8 Hz), 8.25-8.16 (1H, m, Ar-H), 7.77-7.67 (1H, m, Ar-H), 7.63 (1H, d, *J* = 8.1 Hz, H-6), 7.57-7.47 (1H, m, Ar-H), 7.35-7.30 (1H, m, Ar-H), 7.11-7.04 (2H, m, Ar-H), 7.03-6.97 (1H, m, Ar-H), 5.76-5.74 (1H, m, H-1’), 5.71 (1H, d, *J* = 8.0 Hz, H-5), 4.74-4.62 (1H, m, DABA1-α-CH), 4.61-4.43 (4H, m, H-2’ + DABA1-α-CH + 3-Pal3-α-CH + Trp4-α-CH), 4.41-4.32 (1H, m, H-4’), 4.14 (1H, d, *J* = 16.0 Hz, Gly2-α-CH_2), 4.01-3.88 (2H, m, DABA1-β-CH + Gly2-α-CH_2), 3.55-3.46 (1H, m, H-5’), 3.30-3.10 (4H, m, H-5’, 3-Pal3-β-CH_2 + Trp4-β-CH_2), 3.07-2.97 (1H, m, 3-Pal3-β-CH_2 major), 2.88 (3H, s, NCH_3), 2.28-2.18 (1H, m, H-3’), 1.84-1.69 (1H, m, H-3’), 1.25-1.17 (3H, m, DABA1-γ-CH_3). ^{13}**C NMR** (100 MHz, CD_3OD, rotamers in 1.4:1 ratio, *rotamer* 1): δ 176.3 (C=O), 172.8 (C=O), 171.4 (C=O), 167.3 (C=O), 166.8 (C=O), 165.9 (C=O), 152.2 (C=O), 145.8, 145.4, 142.8, 142.2, 137.9 (×2), 128.8, 126.6, 124.3, 122.1, 119.6, 119.2, 111.8, 110.5, 102.2, 94.8, 81.0, 76.1, 56.8, 55.0, 54.8, 54.2, 44.9, 41.0, 36.1, 36.0,

28.6, 27.8, 14.8. **^{1}H NMR** (400 MHz, CD_3OD, rotamers in 1.4:1 ratio, *rotamer* 2): δ 8.33 (1H, d, J = 8.2 Hz), 8.15-8.10 (1H, m, Ar-H), 7.57-7.47 (1H, m, Ar-H, H-6), 5.67 (1H, d, J = 8.1 Hz, H-5), 4.74-4.62 (1H, m, DABA1-β-CH), 4.61-4.43 (1H, m, H-4'), 3.83 (1H, d, J = 16.2 Hz, Gly2-α-CH_2), 3.75 (1H, d, J = 16.2 Hz, Gly2-α-CH_2), 3.46-3.35 (1H, m, H-5'), 3.30-3.10 (4H, m, H-5', 3-Pal3-β-CH_2 + Trp4-β-CH_2), 3.07-2.97 (1H, m, 3-Pal3-β-CH_2), 2.81 (3H, s, NCH_3), 2.35 (1H, *app.* dt, J = 13.6, 7.0 Hz, H-3'), 1.84-1.69 (1H, m, H-3'), 1.25-1.17 (3H, m, DABA1-γ-CH_3). **^{13}C NMR** (100 MHz, CD_3OD, rotamers in 1.4:1 ratio, *rotamer* 2): δ 145.2, 102.5, 80.4, 75.9, 56.4, 53.3, 44.6, 41.2, 36.4, 30.4, 13.6. **LRMS** [M+H^+] 777.4. **HRMS (ESI m/z)** [M+H^+] calcd. for $C_{36}H_{45}N_{10}O_{10}$ 777.3314, found 777.3311.

(2*S*,6*S*,9*S*,10*S*)-2,6-bis((1*H*-indol-3-yl)methyl)-13-amino-9-((((2*R*,4*R*,5*R*)-5-(2,4-dioxo-3,4-dihydropyrimidin-1(2*H*)-yl)-4-hydroxytetrahydrofuran-2-yl)methyl)carbamoyl)-10,11-dimethyl-4,7,12-trioxo-3,5,8,11-tetraazatridecan-1-oic acid (33)

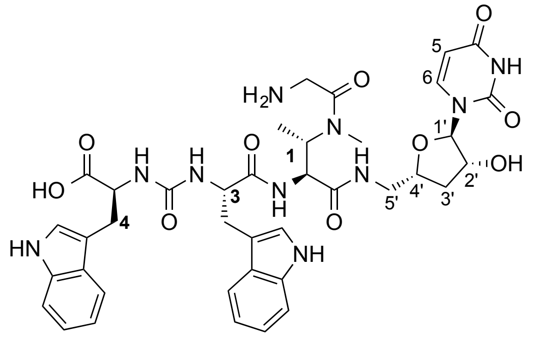

Depsipeptide **S48** (36.0 mg, 37.4 μmol.) was reacted with amine **18** (21.3 mg, 93.5 μmol) in CH_2Cl_2: DMF (1:1 v/v, 380 μL) in the presence of HOAt (25.5 mg, 187.0 μmol), EDC.HCl (9.3 mg, 48.6 μmol) and NMM (5.4 μL, 48.6 μmol) for 4 h according to general procedure 8 to obtain the fully protected dihydrosansanmycin analogue. This compound was then treated with a mixture of TFA and *i*-Pr_3SiH in CH_2Cl_2 (1:1 v/v TFA: CH_2Cl_2, 2.5 vol.% *i*-Pr_3SiH, 1.2 mL) for 16 h according to general procedure 8 to afford dihydrosansanmycin **33** (as a formate salt) after reverse phase HPLC (0 to 50% MeCN over 40 min) as a fluffy white solid (9.1 mg, 32% over two steps).

IR (ATR): 3327, 2964, 2928, 1680, 1554 cm^{-1}. **^{1}H NMR** (400 MHz, DMSO-d_6, rotamers in 1.1:1 ratio, *rotamer 1*): δ 7.54 (2H, m, H-6 + 2x Ar-H), 7.30 (2H, d, J = 8.0 Hz, 2x Ar-H), 7.11-6.92 (6H, m, 6x Ar-H), 7.02 (1H, m, Ar-H), 6.92 (1H, m,

Ar-H), 5.67 (1H, d, $J = 3.4$ Hz, H-1'), 5.59 (1H, app. d, H-5), 4.56 (1H, m, DABA1-α-CH), 4.48-4.25 (4H, m, H-2' + H-4' + Trp3-α-CH + Trp4-α-CH), 3.88 (1H, m, DABA1-β-CH), 3.71 (2H, m, Gly2-α-CH_2), 3.34-2.88 (6H, m, Trp3-β-CH_2 + Trp4-β-CH_2 + 2x H-5'), 2.73 (3H, s, NCH_3), 2.14-2.11 (1H, m, H-3'), 1.76-1.65 (1H, m, H-3'), 1.11 (3H, m, DABA1-γ-CH_3). **^{13}C NMR** (101 MHz, DMSO-d_6, rotamers in 1.1:1 ratio, *rotamer 1*): δ 173.7 (C=O), 169.0 (C=O), 168.0 (C=O), 167.3 (C=O), 163.7 (C=O), 163.6 (C=O), 150.8 (C=O), 141.2, 136.4, 136.3, 127.9, 127.8, 124.3, 123.7, 121.9, 120.8, 119.7, 119.5, 118.6, 118.5, 112.2, 111.5, 111.4, 110.2, 101.8, 92.4, 78.8, 74.3, 55.2, 54.5, 51.0, 44.0, 40.4, 39.4, 35.8, 29.3, 28.3, 27.2, 14.9. **^{1}H NMR** (400 MHz, DMSO-d_6, rotamers in 1.1:1 ratio, *rotamer 2*): δ 5.72 (1H, m, H-1'). **^{13}C NMR** (101 MHz, DMSO-d_6, rotamers in 1.1:1 ratio, *rotamer 2*): 93.4, 102.8. **LRMS** [M^+H^+] 815.4. **HRMS (ESI m/z)** [$M+H^+$] calcd. for $C_{39}H_{47}N_{10}O_{10}$ 815.3471, found 815.3465.

(2*S*,6*S*,9*S*,10*S*)-2-((1*H*-indol-3-yl)methyl)-13-amino-9-((((2*R*,4*R*,5*R*)-5-(2,4-dioxo-3,4-dihydropyrimidin-1(2*H*)-yl)-4-hydroxytetrahydrofuran-2-yl)methyl)carbamoyl)-10,11-dimethyl-4,7,12-trioxo-6-phenyl-3,5,8,11-tetraazatridecan-1-oic acid (34)

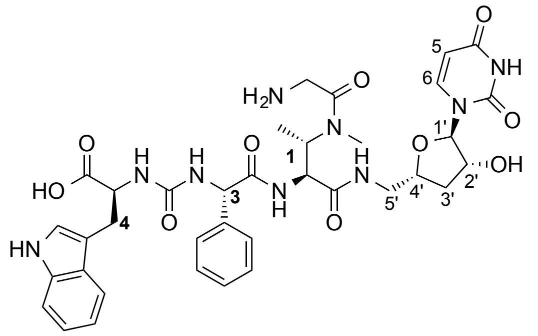

Depsipeptide **S49** (25.3 mg, 31.3 μmol.) was reacted with amine **18** (17.8 mg, 78.2 μmol) in CH_2Cl_2: DMF (1:1 v/v, 320 μL) in the presence of HOAt (21.3 mg, 156.5 μmol), EDC.HCl (7.8 mg, 40.7 μmol) and NMM (4.5 μL, 40.7 μmol) for 4 h according to general procedure 8 to obtain the fully protected dihydrosansanmycin analogue. This compound was then treated with a mixture of TFA and *i*-Pr_3SiH in CH_2Cl_2 (1:1 v/v TFA: CH_2Cl_2, 2.5 vol.% *i*-Pr_3SiH, 1.2 mL) for 16 h according to general procedure 8 to afford dihydrosansanmycin **34** (as a formate salt) after reverse phase HPLC purification (0 to 50% MeCN over 40 min) as a fluffy white solid (12.0 mg, 47% over two steps).

IR (ATR): 3407, 2923, 2853, 1679, 1437 cm^{-1}. **^{1}H NMR** (400 MHz, DMSO-d_6, rotamers in 1.3:1 ratio, *rotamer 1*): δ 7.52 (2H, m, H-6 + Ar-H), 7.35–7.29 (6H, m, 6x Ar-H), 7.11–6.93 (3H, m, 3x Ar-H), 5.64–5.59 (2H, m, H-1' + H-5), 4.81 (1H, m, Phg3-α-CH), 4.34 (1H, m, DABA1-α-CH), 4.32–4.25 (3H, m, H-2' + H-4' + Trp4-α-CH), 3.90 (1H, m, DABA1-β-CH), 3.71-3.50 (4H, m, Gly2-α-CH_2 + Trp4-β-CH_2), 3.24–2.99 (4H, m, Trp4-β-CH_2 + 2x H-5'), 2.73 (3H, s, NCH_3), 2.06–1.98 (1H, m, H-3'), 1.76 – 1.65 (1H, m, H-3'), 1.13 (3H, m, DABA1-γ-CH_3). **^{1}H NMR** (400 MHz, DMSO-d_6, rotamers in 1.3:1 ratio, *rotamer 2*): δ 2.17-2.08 (1H, m, H-3'). **^{13}C NMR** (101 MHz, DMSO-d_6; rotamers in 1.3:1 ratio, *rotamer 1*): δ 175.1 (C=O), 174.8 (C=O), 173.0 (C=O), 169.9 (C=O), 168.7 (C=O), 167.9 (C=O), 163.6, 150.8 (C=O), 141.5, 136.2, 128.5, 128.0, 127.7, 123.8, 121.0, 118.9, 118.5, 111.5, 110.6, 101.9, 92.4, 78.7, 74.4, 57.0, 55.4, 54.6, 53.3, 50.8, 44.0, 40.5, 36.5, 29.1, 28.3, 27.3, 14.9. **^{13}C NMR** (101 MHz, DMSO-d_6, rotamers in 1.3:1 ratio, *rotamer 2*): δ 35.7. **LRMS** [M+H$^+$] 762.4. **HRMS (ESI m/z)** [M+H$^+$] calcd. for $C_{36}H_{44}N_9O_{10}$ 762.3206, found 762.3200.

(2*S*,6*S*,9*S*,10*S*)-2-((1*H*-indol-3-yl)methyl)-13-amino-9-((((2*R*,4*R*,5*R*)-5-(2,4-dioxo-3,4-dihydropyrimidin-1(2*H*)-yl)-4-hydroxytetrahydrofuran-2-yl)methyl)carbamoyl)-10,11-dimethyl-4,7,12-trioxo-6-phenethyl-3,5,8,11-tetraazatridecan-1-oic acid (35)

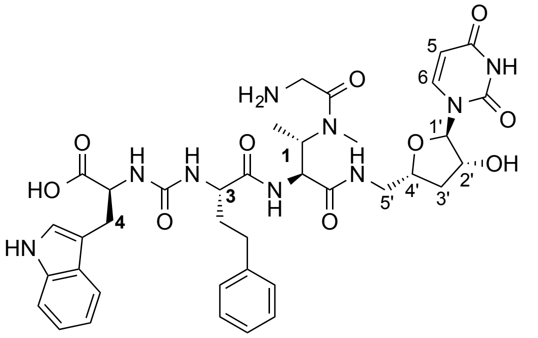

Depsipeptide **S50** (29.3 mg, 35.1 µmol.) was reacted with amine **18** (19.9 mg, 87.7 µmol) in CH_2Cl_2: DMF (1:1 v/v, 360 µL) in the presence of HOAt (23.9 mg, 175.5 µmol), EDC.HCl (8.7 mg, 45.6 µmol) and NMM (5.0 µL, 45.6 µmol) for 4 h according to general procedure 8 to obtain the fully protected dihydrosansanmycin analogue. This compound was then treated with a mixture of TFA and *i*-Pr_3SiH in CH_2Cl_2 (1:1 v/v TFA: CH_2Cl_2, 2.5 vol.% *i*-Pr_3SiH, 1.2 mL) for 16 h according to general procedure 8 to afford dihydrosansanmycin **35** (as a formate

salt) after reverse phase HPLC purification (0 to 50% MeCN over 40 min) as a fluffy white solid (7.0 mg, 24% over two steps).

IR (ATR): 3377, 2960, 2929, 1680, 1435 cm^{-1}. **^{1}H NMR** (500 MHz, DMSO-d_6, rotamers in 1.5:1 ratio, *major rotamer*): δ 7.53 (2H, m, H-6 + Ar-H), 7.30 (7H, m, 7x Ar-H), 7.01 (2H, m, 2x Ar-H), 5.64 (1H, m, H-1'), 5.55 (1H, m, H-5), 4.56 (1H, m, DABA1-α-CH), 4.28 (3H, m, Trp4-α-CH + H-2' + H-4'), 4.10–4.04 (2H, m, HPhe3-α-CH + DABA1-β-CH), 3.69 (2H, m, Gly2-α-CH_2), 3.26-3.14 (4H, m, Trp4-β-CH_2 + 2x H-5'), 2.73 (3H, s, NCH_3), 2.50 (2H, obscure, HPhe3-γ-CH_2), 2.09 (1H, m, H-3'), 1.83-1.66 (3H, m, H-3'+ HPhe3-β-CH_2), 1.12 (3H, m, DABA1-γ-CH_3). **^{13}C NMR** (126 MHz, DMSO-d_6, rotamers in 1.5:1 ratio, *major rotamer*): δ 177.7 (C=O), 172.7 (C=O), 171.4 (C=O), 169.9 (C=O), 169.0 (C=O), 163.7 (C=O), 150.8 (C=O), 141.4, 136.3, 128.8, 128.7, 126.2, 126.0, 125.1, 124.1, 121.0, 118.9, 111.4, 110.3, 101.6, 92.5, 79.0, 78.9, 74.2, 55.5, 53.4, 52.9, 50.8, 43.8, 40.9, 40.3, 35.7, 31.4, 29.2, 27.5, 15.1. **LRMS** [M+H^+] 790.5. **HRMS (ESI m/z)** [M+H^+] calcd. for $C_{38}H_{48}N_9O_{10}$ 790.3518, found 790.3509.

(((*S*)-1-(((2*S*,3*S*)-3-(2-amino-*N*-methylacetamido)-1-((((2*R*,4*R*,5*R*)-5-(2,4-dioxo-3,4-dihydropyrimidin-1(2*H*)-yl)-4-hydroxytetrahydrofuran-2-yl)methyl)amino)-1-oxobutan-2-yl)amino)-3-(naphthalen-2-yl)-1-oxopropan-2-yl)carbamoyl)-*L*-tryptophan (36)

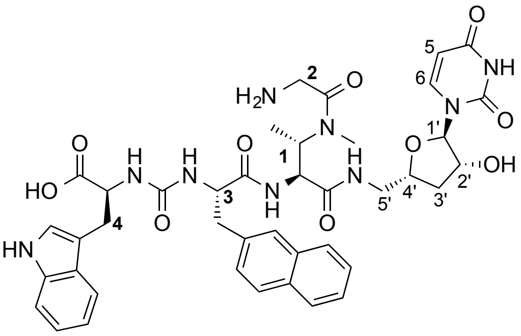

Depsipeptide **S51** (21 mg, 25 μmol) was reacted with amine **18** (14 mg, 63 μmol) in CH_2Cl_2: DMF (1:1 v/v, 250 μL) in the presence of HOAt (17 mg, 123 μmol) and EDC.HCl (6.1 mg, 32 μmol) and NMM (3.5 μL, 32 μmol) for 4 h at rt according to general procedure 8 condition B to obtain the fully protected dihydrosansanmycin analogue. This compound was treated with a mixture of TFA and *i*Pr_3SiH in CH_2Cl_2 (1:1 v/v TFA: CH_2Cl_2, 2.5 vol.% *i*Pr_3SiH, 2.5 mL) for 4 h according to general

procedure 8 to afford dihydrosansanmycin **36** (as a formate salt) after reverse phase HPLC purification (0 to 50% MeCN over 40 min, 10 min at 100% H_2O, flow rate 9 mL/min) as an amorphous white solid (6.0 mg, 28% over 2 steps).

IR (ATR): 3390, 2977, 2936, 1730, 1644 cm^{-1}. **^{1}H NMR** (500 MHz, CD_3OD, *major rotamer*): δ 8.56 (1H, t, *J* = 6.0 Hz, 1H), 8.16 (1H, d, *J* = 9.1 Hz), 8.13 (0.4H, d, *J* = 8.6 Hz), 7.84 (1H, t, *J* = 5.9 Hz), 7.81-7.69 (2H, m, Ar-H), 7.65-7.60 (1H, m, Ar-H), 7.58-7.54 (2H, m, Ar-H + H-6), 7.45-7.40 (3H, m, Ar-H), 7.36-7.29 (2H, m, Ar-H), 7.11-7.04 (2H, m, Ar-H), 7.03-6.92 (1H, m, Ar-H), 5.72-5.66 (2H, m, H-1' + H-5), 4.64-4.53 (1H, m, Trp-α-CH), 4.53-4.48 (1H, m, DABA1-α-CH), 4.48-4.34 (2H, H-2' + Nal3-α-CH), 4.25-4.16 (1H, m, H-4'), 4.07 (1H d, *J* = 15.9 Hz, Gly2-α-CH_2), 3.88-3.81 (2H, m, DABA1-β-CH + Gly2-α-CH_2), 3.30-3.23 (1H, m, Trp4-β-CH_2), 3.22-3.04 (4H, m, H-5' + Trp4-β-CH_2 + Nal3-β-CH_2), 3.13-3.03 (1H, m, H-5'), 2.91 (1H, ddd, *J* = 13.6, 8.6, 4.8 Hz, H-5'), 2.83 (3H, s, NCH_3), 2.14 (1H, ddd, *J* = 13.6, 7.5, 6.0 Hz, H-3'), 1.66-1.59 (1H, m, H-3'), 1.17 (3H, d, *J* = 6.6 Hz, DABA1-γ-CH_3). **^{13}C NMR** (125 MHz, CD_3OD, *major rotamer*, two ^{13}C signals are obscure): δ 176.0 (C=O), 174.4 (C=O), 171.0 (C=O), 167.6 (C=O), 166.3 (C=O), 159.5 (C=O), 152.4 (C=O), 142.6, 142.4, 137.6, 135.8, 134.7, 134.0, 128.8 (×2), 128.6, 128.2, 126.6, 124.4, 122.0, 119.6, 119.2, 112.0, 110.6, 102.3, 94.8, 81.1, 76.0, 56.6 (×2), 55.0, 54.8, 45.0, 40.9, 39.2, 35.9, 29.1, 27.7, 14.5. **LRMS** [M+H$^+$] 826.4. **HRMS (ESI m/z)** [*M*+H$^+$] calcd. for $C_{41}H_{48}N_9O_{10}$ 826.3518, found 826.3515.

(((*S*)-1-(((2*S*,3*S*)-3-((*S*)-2-amino-3-(3-hydroxyphenyl)-*N*-methylpropanamido)-1-((((2*R*,4*R*,5*R*)-5-(2,4-dioxo-3,4-dihydropyrimidin-1(2*H*)-yl)-4-hydroxytetrahydrofuran-2-yl)methyl)amino)-1-oxobutan-2-yl)amino)-3-cyclohexyl-1-oxopropan-2-yl)carbamoyl)-*L*-tryptophan (37)

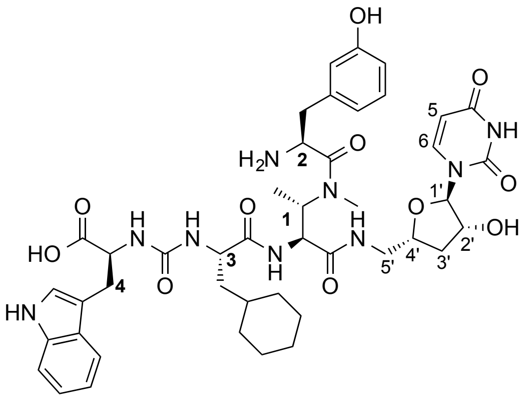

Depsipeptide **S52** (18 mg, 17 μmol) was reacted with amine **18** (12 mg, 52 μmol) in CH_2Cl_2: DMF (1:1 v/v, 180 μL) in the presence of HOAt (11 mg, 150 μmol) and EDC.HCl (4.4 mg, 22 μmol) and NMM (2.6 μL, 22 μmol) for 2.5 h at rt according to general procedure 8 condition B to obtain the fully protected dihydrosansanmycin analogue. This compound was treated with a mixture of TFA and *i*Pr_3SiH in H_2O (9:1 v/v TFA: CH_2Cl_2, 2 vol.% *i*Pr_3SiH, 1.7 mL) for 45 min at 0 ^oC and 2 h at rt to afford dihydrosansanmycin **37** (as a TFA salt) after reverse phase HPLC purification (0 to 50% MeCN over 40 min, 10 min at 100% H_2O) as an amorphous white solid (8.0 mg, 53% over 2 steps).

IR (ATR): 3388, 2926, 1647 cm^{-1}. **^{1}H NMR** (500 MHz, CD_3OD, rotamers in 2.6:1 ratio, *major rotamer*): δ 7.62 (1H, d, *J* = 8.1 Hz, H-6), 7.54 (1H, *app.* t, *J* = 7.8 Hz, Ar-H), 7.32 (1H, *app.* d, *J* = 8.2 Hz, Ar-H), 7.18 (1H, *app.* t, *J* = 7.9 Hz, Ar-H), 7.12-7.04 (2H, m, Ar-H), 7.02-6.96 (1H, m, Ar-H), 6.78-6.71 (2H, m, Ar-H), 6.69-6.66 (1H, m, Ar-H), 5.74 (1H, d, *J* = 2.5 Hz, H-1'), 5.71 (1H, d, *J* = 8.1 Hz, H-5), 4.91-4.75 (1H, m, *m*-Tyr2-α-CH), 4.59-4.49 (2H, m, DABA1-α-CH + Trp4-α-CH), 4.48-4.35 (2H, H-2' + H-4'), 4.23-4.07 (2H, m, DABA1-β-CH + Cha3-α-CH), 3.52 (1H, dd, *J* = 13.9, 3.9 Hz, H-5'), 3.29-3.23 (2H, m, H-5' + Trp4-β-CH_2), 3.23-3.11 (1H, m, Trp4-β-CH_2), 3.02-2.97 (2H, m, *m*-Tyr2-β-CH_2), 2.78 (3H, s, NCH_3), 2.23 (1H, *app.* dt, *J* = 13.7, 7.0 Hz, H-3'), 1.82-1.58 (5H, m, H-3' + 2× CH_2), 1.48-1.40 (2H, m, Cha3-β-CH_2), 1.38-1.11 (6H, m, Cha3-γ-CH + 2.5× CH_2), 0.98-0.85 (1H, m, 0.5× CH_2), 0.82 (3H, d, *J* = 6.5 Hz, DABA1-γ-CH_3). **^{13}C NMR** (125 MHz, CD_3OD, rotamers in 2.6:1 ratio, *major rotamer*): δ 175.8 (C=O), 175.4 (C=O), 171.3 (C=O), 170.2 (C=O), 165.8 (C=O), 159.8 (C=O), 159.0, 151.6 (C=O), 142.1, 137.6, 136.1, 131.2, 128.6, 124.4, 122.2, 121.7, 119.7, 119.3, 116.9, 115.7, 112.1, 110.5, 102.3, 94.9, 80.9, 76.2, 56.9, 54.6, 54.5, 52.9, 52.8, 44.8, 40.4, 38.3, 35.7, 35.0, 33.8, 28.8, 28.3, 27.1, 14.2. **LRMS** [M+H^+] 888.3. **HRMS (ESI m/z)** [*M*+H^+] calcd. for $C_{44}H_{58}N_9O_{11}$ 888.4250, found 888.4244.

Cytotoxicity Screening of Dihydrosansanmycin Analogues

Screening of sansanmycin analogues against HEK293 cells (ATCC 11268). Potential cytotoxic effects of dihydrosansanmycin analogues against Human Embryonic Kidney cells (HEK293) was determined *via* a resazurin reduction microplate assay. HEK293 cells were plated at 1 x 10^4 cells/well in a 96-well micrometer plate, in complete media DMEM and incubated at 37 °C for 48 h in a humidified incubator with 5% CO_2. After this time, serially diluted aliquots of analogues were added to the wells and the plates incubated for a further 24 h as above. Resazurin was then added to a final concentration of 0.0065% to each well and the plates incubated for a further 3 h before sample fluorescence (ex: 530 nm; em: 590 nm) was measured on a fluorescent plate reader (BMG Labtech).

SUPPLEMENTARY REFERENCES

(1) Boojamra, C. G. *et al.* Stereochemical elucidation and total synthesis of dihydropacidamycin D, a semisynthetic pacidamycin. *J. Am. Chem. Soc.* **123**, 870-874 (2001)
